# PNPLA3-I148M genetic variant rewires lipid metabolism to drive programmed cell death in human hepatocytes

**DOI:** 10.1172/jci.insight.193805

**Published:** 2025-10-21

**Authors:** Rodrigo M. Florentino, Olamide Animasahun, Nils Haep, Minal Nenwani, Kehinde Omoloja, Leyla Nurcihan Altay, Abhinav Achreja, Kazutoyo Morita, Takashi Motomura, Ricardo Diaz-Aragon, Lanuza A.P. Faccioli, Yiyue Sun, Zhenghao Liu, Zhiping Hu, Bo Yang, Fulei Wuchu, Ajay Shankaran, Miya Paserba, Annalisa M. Baratta, Shohrat Arazov, Zehra N. Kocas-Kilicarslan, Noah Meurs, Jaideep Behari, Edgar N. Tafaleng, Jonathan Franks, Alina Ostrowska, Takahiro Tomiyama, Kyohei Yugawa, Akinari Morinaga, Zi Wang, Kazuki Takeishi, Dillon C. Gavlock, Mark Miedel, D. Lansing Taylor, Ira J. Fox, Tomoharu Yoshizumi, Deepak Nagrath, Alejandro Soto-Gutierrez

**Affiliations:** 1 Department of Pathology,; 2 Center for Transcriptional Medicine, and; 3 Pittsburgh Liver Research Center, University of Pittsburgh, Pittsburgh, Pennsylvania, USA.; 4 Department of Chemical Engineering,; 5 Biointerfaces Institute,; 6 Laboratory for Systems Biology of Human Diseases, and; 7 Department of Biomedical Engineering, University of Michigan, Ann Arbor, Michigan, USA.; 8 Department of Medicine, Division of Gastroenterology, Hepatology, and Nutrition, University of Pittsburgh Medical Center (UPMC), Pittsburgh, Pennsylvania, USA.; 9 Center for Biologic Imaging, University of Pittsburgh, Pittsburgh, Pennsylvania, USA.; 10 Department of Surgery and Science, Graduate School of Medical Sciences, Kyushu University, Fukuoka, Japan.; 11 Department of Statistics,; 12 University of Pittsburgh Drug Discovery Institute,; 13 Department of Computational and Systems Biology, and; 14 Department of Surgery, Children’s Hospital of Pittsburgh of UPMC, University of Pittsburgh, Pittsburgh, Pennsylvania, USA.

**Keywords:** Gastroenterology, Hepatology, Cell stress, Fatty acid oxidation, Lipidomics

## Abstract

Genetic variants in lipid metabolism influence the risk of developing metabolic dysfunction–associated steatotic liver disease (MASLD), cirrhosis, and end-stage liver disease (ESLD). The mechanisms by which these variants drive disease are poorly understood. Because of the PNPLA3-I148M variant’s strong correlation with all stages of the MASLD spectrum and the lack of tractable therapeutic targets, we sought to understand its impact on cellular function and liver metabolism. Primary human hepatocytes (HAHs) and induced pluripotent stem cell–derived (iPSC-derived) hepatocytes (iHeps) from healthy individuals possessing the PNPLA3-I148M mutation were characterized for changes in lipid metabolism, cellular stress, and survival. Using lipidomics, metabolomics, stable isotope tracing, and flux propensity analysis, we created a comprehensive metabolic profile of the changes associated with the PNPLA3-I148M variant. Functional analysis showed that the presence of the PNPLA3-I148M variant increased endoplasmic reticulum stress, mitochondrial dysfunction, and peroxisomal β-oxidation, ultimately leading to cell death via ferroptosis. Nutritional interventions, ferroptosis-specific inhibitors, and genetic approaches modulating GPX4 activity in PNPLA3-I148M HAHs and iHeps decreased programmed cell death. Our findings indicate that therapies targeting ferroptosis in patients carrying the PNPLA3-I148M variant could affect the development of MASLD and ESLD and highlight the utility of iPSC-based models for the study of genetic contributions to hepatic disorders.

## Abstract 2

Using an integrative multi-omics analysis, we mapped the metabolic rewiring induced by the PNPLA3-I148M variant in human hepatocytes. ER stress, mitochondrial dysfunction, and peroxisomal β-oxidation that ultimately led to cell death via ferroptosis were observed in the samples carrying the PNPLA3 mutation.

## Introduction

Globally, end-stage liver disease (ESLD) claims over 2 million lives each year (1). Moreover, metabolic dysfunction–associated steatotic liver disease (MASLD) is the most rapidly increasing cause of ESLD in the United States (2). In addition, there is a paucity of FDA-approved therapies for MASLD. Resmetirom, a thyromimetic engineered for selective activation of thyroid hormone receptor β in the liver, is the only approved drug therapy for the treatment of the disease (3). MASLD is affected by genetic and environmental factors, inflammatory cytokines, adipokines, bacterial products, and metabolites originating from the intestine and adipose tissue (4). Aggressive clinical management of ESLD can extend life, but the only definitive therapy is liver transplantation. Widespread use of liver transplantation, however, is limited by a shortage of donor organs, primary graft dysfunction, the need for long-term immune suppression, and high cost; thus, understanding disease mechanism and progression is imperative for the development of new therapeutics.

GWAS (5–7) have identified several variants or combinations of variants, including in patatin-like phospholipase domain-containing protein 3 (*PNPLA3*; rs738409 C>G p.Ie148Met), that are associated with predisposed susceptibility to MASLD and progression to fibrosis and ESLD, especially when synergized in individuals with a body mass index greater than 35 kg/m^2^. Here, we have focused on the PNPLA3-I148M variant because of its ubiquitous association with every step on the MASLD spectrum (including metabolic dysfunction–associated steatohepatitis [MASH] and hepatocellular carcinoma) and lack of tractable mechanistic targets (8). Most of the gene variant associations examined, including PNPLA3-I148M, highlight the role of lipid droplet biology, intracellular lipid synthesis and degradation, and secretion of very low-density lipoproteins as potential mechanisms behind the development of MASLD. Recently, it was reported that PNPLA3-I148M sequesters the hydrolase 1-acylglycerol-3-phosphate O-acyltransferase, causing a decrease of adipose triglyceride lipase (ATGL) lipolytic activity, which resulted in intracellular fat accumulation (9). Functional analyses of these variants in human livers are relatively underexplored, and the mechanisms by which the variants lead to ESLD, metabolic adaptations, and cellular death are poorly understood.

To elucidate the impact of the genetic variant in healthy donors, we obtained primary human adult hepatocytes (HAHs) and induced pluripotent stem cells (iPSCs) with the PNPLA3-I148M variant from healthy individuals and, after creating hepatocytes from the iPSCs (iHeps) and corresponding isogeneic Cas9-based gene-edited controls, dissected the molecular and metabolic consequences of the PNPLA3-I148M variant using an integrated multiomics approach encompassing semi-targeted metabolomics and transcriptomics. Additionally, we investigated the effect of this variant on mitochondrial morphology, peroxisomal fatty acid β-oxidation, lipid peroxidation, and transferrin receptor 1 (TFRC) mobilization, which can contribute to cell death via ferroptosis in both HAHs and iHeps. To increase survival of HAHs and iHeps, ferroptosis-preventive approaches were used. Collectively, our results have elucidated a potential mechanism of metabolic regulation by the PNPLA3-I148M variant and revealed that personalized therapies that deter ferroptosis can reverse programmed cell death by reducing lipid peroxidation. Importantly, our study highlights the utility of iPSCs in modeling and investigating the contribution of genetic variants to hepatic disorders for the development of new therapeutic strategies.

## Results

### The PNPLA3-I148M variant in primary human hepatocytes modulates lipid accumulation, cellular stress, mitochondrial function, and metabolism.

**Figure 1:**
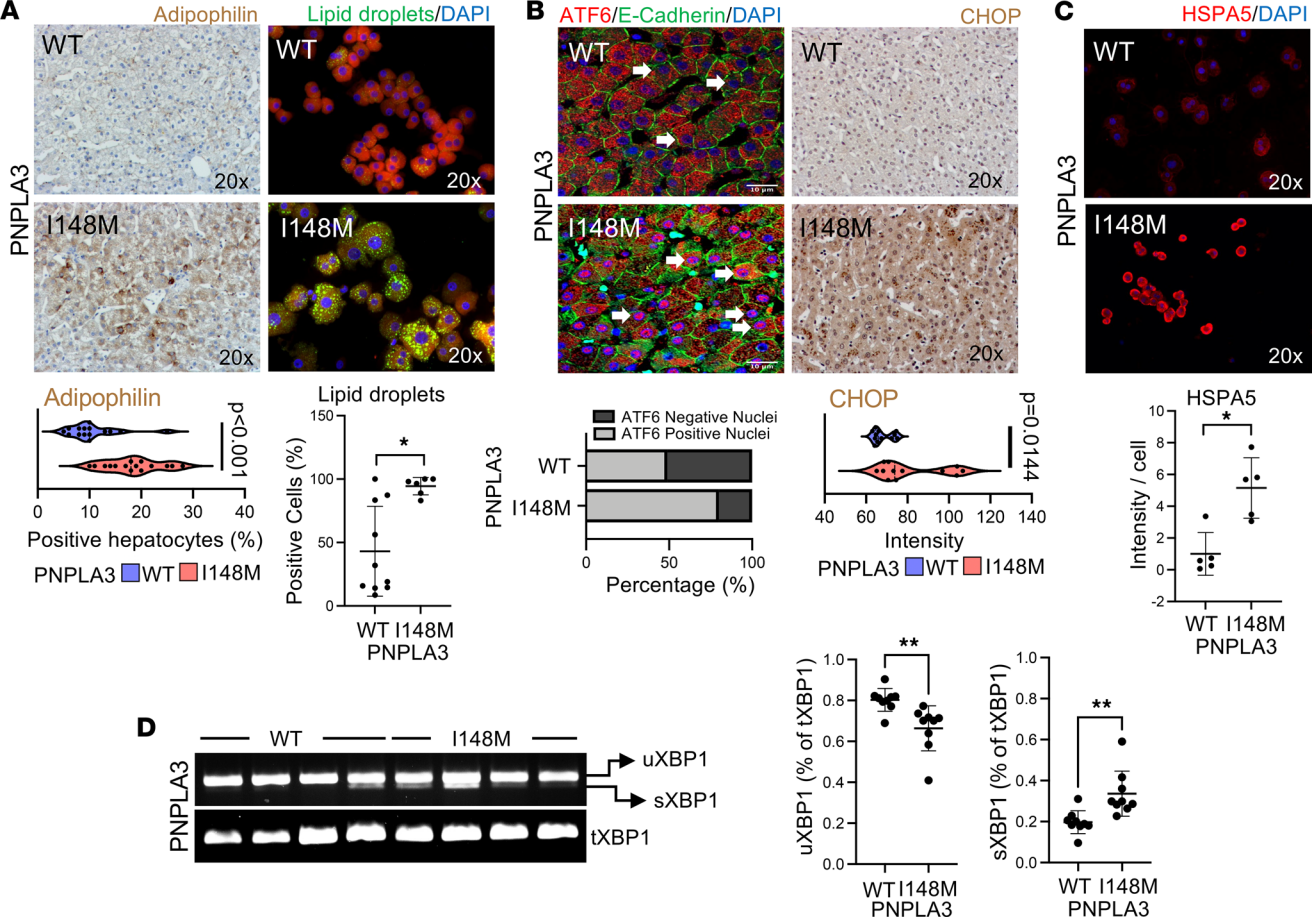
The PNPLA3-I148M mutation leads to intracellular lipid accumulation and ER stress in primary human hepatocytes. (**A**) Histological micrographs of donor livers. Adipophilin 2 immunohistochemistry-positive hepatocyte quantification (*P* < 0.001, Welch’s *t* test, WT: *n* = 3 normal livers/5 different fields each, I418M: *n* = 3 normal livers/5 fields each). Nile Red staining micrographs and quantification of lipid droplet-positive Hep-PNPLA3-I148M and Hep-PNPLA3-WT (*P = 0.01, Mann-Whitney test, WT: *n* = 10, I148M: *n* = 6). (**B**) Histological micrographs (ATF6 and CHOP) of donor livers. Quantification of immunofluorescence signal in the cell nucleus (*P* < 0.01, Mann-Whitney test, WT: *n* = 6, I148M: *n* = 6). CHOP immunohistochemistry signal quantification (*P* = 0.0144, Mann-Whitney test, WT: *n* = 6, I148M: *n* = 6). (**C**) Immunofluorescence micrographs of HSPA5 in primary human hepatocytes. HSPA5 immunofluorescence intensity quantification (*P = 0.02, Mann-Whitney test, WT: *n* = 6, I148M: *n* = 6). (**D**) XBP1 splice detection. Quantification of unspliced XBP1 (**P = 0.005, Welch’s *t* test, WT: *n* = 9, I148M: *n* = 9) and spliced XBP1 as percentage of total XBP1 (**P = 0.005, Welch’s *t* test, WT: *n* = 9, I148M: *n* = 9).

To determine how the PNPLA3-I148M variant affects cellular fitness, we obtained liver tissue and primary hepatocytes from individuals with the PNPLA3-I148M variant without previous diagnosis of hepatic disorders. Staining for adipophilin, a key player in the formation of lipid droplets and intracellular lipid uptake (10), revealed a significant increase in PNPLA3-I148M compared with PNPLA3-WT tissue (Figure 1A). Similarly, primary WT (Hep-PNPLA3-WT) and mutant (Hep-PNPLA3-I148M) hepatocytes were analyzed for the presence of lipid droplets, and Hep-PNPLA3-I148M showed a significant increase compared with controls, indicating intracellular accumulation of lipids in the presence of the variant (Figure 1A).

ER stress is a critical consequence of the PNPLA3-I148M mutation (11). Beginning with liver tissue, we measured expression of activating transcription factor 6 (ATF6), an ER-bound protein that translocates to the nucleus in response to stress (12), and the C/EBP homologous protein (CHOP), a transcription factor involved in the ER stress response (13). We observed a significant increase in both ATF6 and CHOP in PNPLA3-I148M liver tissue compared with control (Figure 1B). We next assessed levels of heat shock protein family A member 5 (HSPA5), another known indicator of ER stress, in Hep-PNPLA3-WT and Hep-PNPLA3-I148M. Immunofluorescence revealed an increase in HSPA5 expression in Hep-PNPLA3-I148M cells compared with WT (Figure 1C). Finally, elevations in ER stress are known to impact alternative splicing of X-box-binding protein 1 (XBP1), such that elevated stress results in the removal of a 26-nucleotide intron (14). To evaluate this phenomenon, we quantified expression of the unspliced (uXBP1) and spliced (sXBP1) proteins as a percentage of total XBP1 (tXBP1) expression (14). Consistent with previous findings, Hep-PNPLA3-I148M presented with significantly increased XBP1 mRNA splicing when compared with Hep-PNPLA3-WT (Figure 1D).

**Figure 2:**
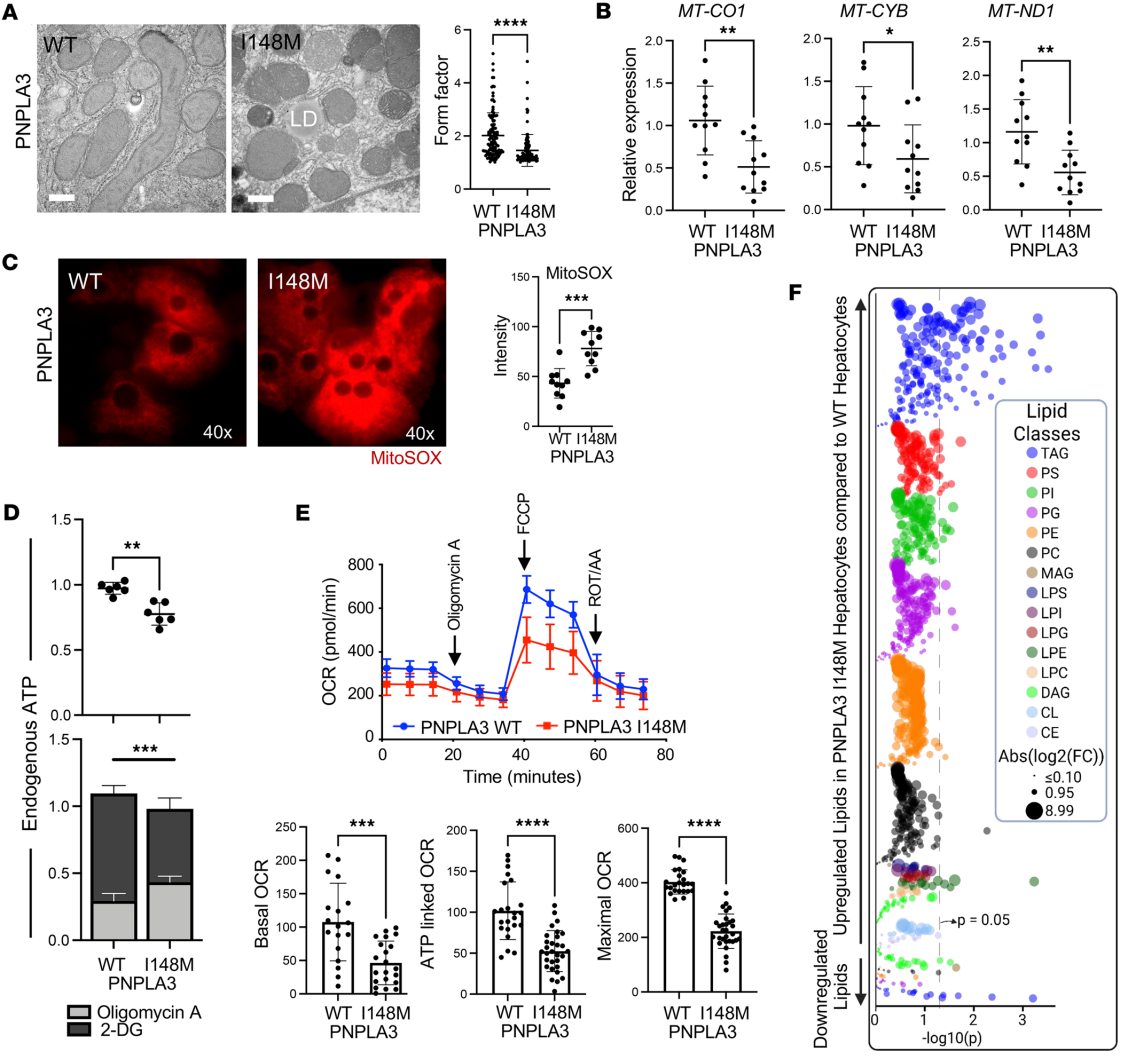
The PNPLA3-I148M mutation leads to mitochondrial dysfunction in primary human hepatocytes. (**A**) Transmission electron microscopy images of mitochondria in hepatocytes (scale bar: 400 nm; LD, lipid droplet) and mitochondria form factor (****P < 0.0001, Mann-Whitney test, WT: *n* = 122, I148M: *n* = 104). (**B**) Relative expression of mitochondrial genes: *MT-CO1* (**P = 0.0021, Mann-Whitney test, WT: *n* = 11, I148M: *n* = 11), *MT-CYB* (*P = 0.0461, Mann-Whitney test, WT: *n* = 11, I148M: *n* = 11) and *MT-ND1* (**P = 0.0029, Mann-Whitney test, WT: *n* = 11, I148M: *n* = 11). (**C**) MitoSOX micrographs and quantification of mitochondrial stress in Hep-PNPLA3*-*I148M and Hep-PNPLA3-WT (***P = 0.0001, Mann-Whitney test, WT: *n* = 10, I148M: *n* = 10). (**D**) Total endogenous ATP quantification in primary hepatocytes (**P = 0.0012, Welch’s *t* test, WT: *n* = 6, I148M: *n* = 6) and relative ATP contribution of the glycolytic and oxidative phosphorylation systems in energy production (***P < 0.001). (**E**) Mitochondria function was assessed by quantification of the oxygen consumption rate (OCR) in Hep-PNPLA3-WT and Hep-PNPLA3-I148M. The basal OCR, ATP-linked OCR, and maximal OCR were calculated (***P < 0.001, ****P < 0.0001, Welch’s *t* test). (**F**) Bubble plot comparing the lipid profiles of Hep-PNPLA3-I148M and Hep-PNPLA3-WT. The size of the bubbles indicates the absolute log_2_ fold change of the lipid species upregulated/downregulated. The color of the bubbles corresponds to the different lipid classes.

An additional consequence of the PNPLA3-I148M variant is abnormality in mitochondrial structure and function (15). Cellular ultrastructure analysis of Hep-PNPLA3-WT and Hep-PNPLA3-I148M revealed an increased number of spherical mitochondria among Hep-PNPLA3-I148M cells rather than the rod shape commonly found in human hepatocytes (Figure 2A). Expression of the mitochondrial genes mitochondrially encoded cytochrome c oxidase I (*MT-CO1*), mitochondrially encoded cytochrome b (*MT-CYB*), and mitochondrially encoded NADH dehydrogenase I (*MT-ND1*), essential components of the oxidative phosphorylation system, was significantly lower in Hep-PNPLA3-I148M compared with Hep-PNPLA3-WT (Figure 2B). Treatment of cells with an indicator of mitochondrial superoxide demonstrated a significant increase in mitochondrial oxidation in Hep-PNPLA3-I148M (Figure 2C). To further characterize changes in energy production in Hep-PNPLA3-I148M cells, we assessed the levels of ATP. We found that Hep-PNPLA3-I148M had significantly reduced ATP levels when compared with Hep-PNPLA3-WT controls (Figure 2D). To determine how Hep-PNPLA3-WT and Hep-PNPLA3-I148M maintain ATP production, we examined whether energy was derived from oxidative phosphorylation or glycolysis. We measured ATP levels in the presence of either an inhibitor of mitochondrial oxidative phosphorylation, oligomycin A, or an inhibitor of glycolysis, 2-deoxyglucose. In Hep-PNPLA3-I148M, a shift in energy production from oxidative phosphorylation to glycolysis occurred (Figure 2D), which may compensate for decreased oxidative phosphorylation. To further validate mitochondrial dysfunction in the Hep-PNPLA3-I148M, we measured oxygen consumption rate (OCR). After exposing the cells to oligomycin A, carbonyl cyanide-4-(trifluoromethoxy) phenylhydrazone (FCCP), and rotenone and antimycin A (ROT/AA), we observed reduced basal, ATP-linked, and maximal OCR in Hep-PNPLA3-I148M (Figure 2E).

One of the well-documented hallmarks of hepatocytes carrying MASLD-associated genetic variants is alteration in the prevailing lipid profile (16). Corroboratively, Hep-PNPLA3-I148M showed upregulated levels of several lipid classes, including lipid droplet–associated triglycerides (TG), phosphatidylcholine (PC), and phosphatidylethanolamine (PE) (Figure 2F and Supplemental Figure 1A; supplemental material available online with this article; https://doi.org/10.1172/jci.insight.193805DS1). In the context of MASLD, alterations in hepatic mitochondrial cardiolipin (CL) can impair structural integrity and the electron transfer necessary for optimal ATP production (17). The presence of multiple double bonds in CLs’ fatty acyl groups makes them vulnerable targets for lipid peroxidation, leading to dysfunctional mitochondrial activities (18). Compared with Hep-PNPLA3-WT, we observed upregulated levels of multi–double bond CLs in Hep-PNPLA3-I148M, corroborating the previously observed abnormalities in mitochondrial structure and function (Supplemental Figure 1B). Taken together, the PNPLA3-I148M variant leads to lipid accumulation, ER stress, mitochondrial dysfunction, and lipid metabolism alterations that may predispose individuals to developing hepatic disorders.

### Transcriptomic changes in Hep-PNPLA3-I148M cells reveal alterations associated with cellular metabolism.

**Figure 3:**
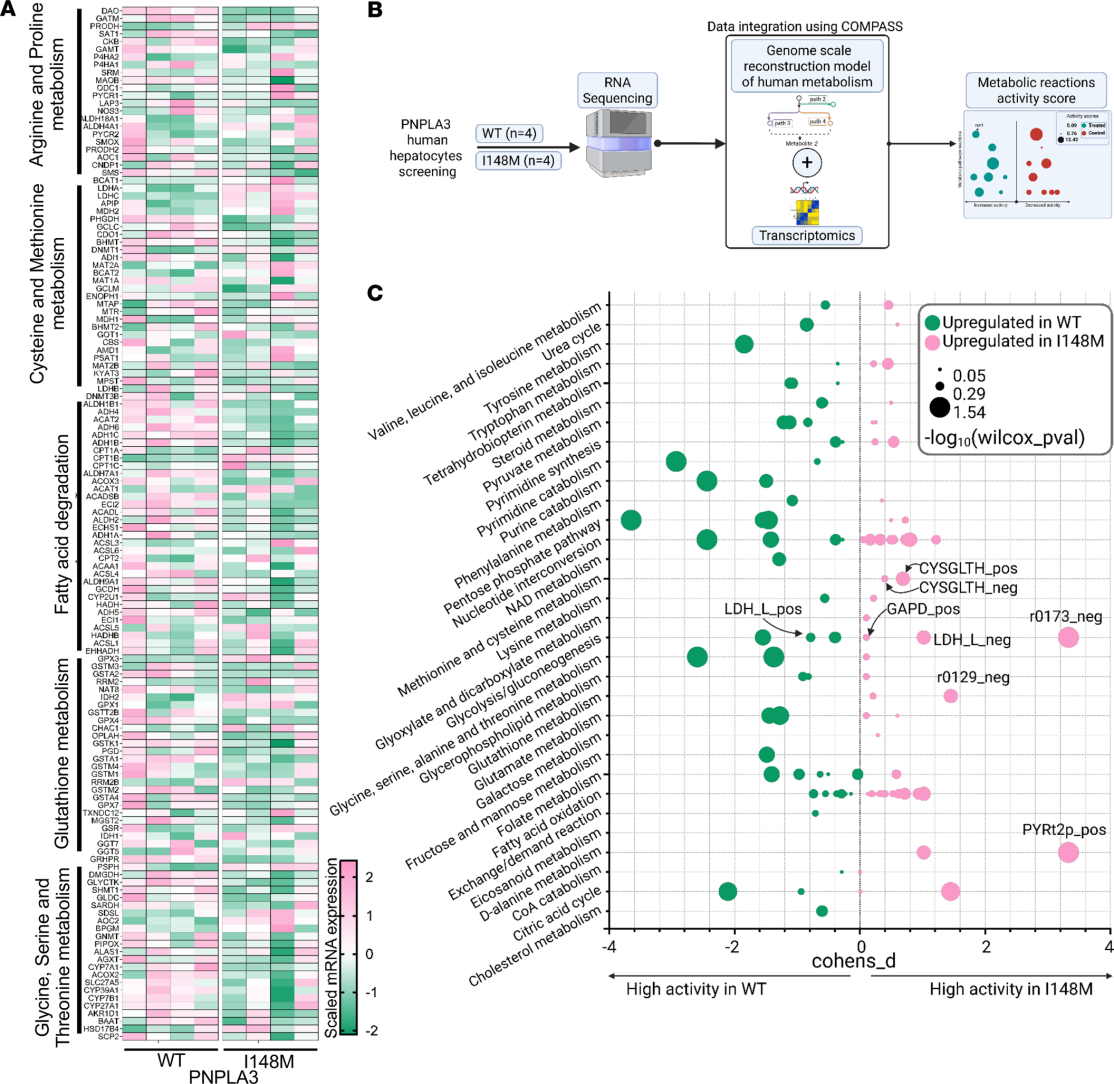
Integration of transcriptomic data with a genome-scale model of human metabolism unravels the rewired metabolism in human Hep-PNPLA3-I148M. (**A**) Heatmap showing the *z*-scored mRNA expression level of metabolic genes in Hep-PNPLA3-I148M (*n* = 4) and Hep-PNPLA3-WT (*n* = 4). The genes are grouped according to their affiliated metabolic pathways. (**B**) Schematic of the workflow for integrating RNA-Seq data with Recon 2.2, a genome scale reconstruction model of human metabolism. The pipeline generates reaction activity scores that correspond to the degree of alterations in metabolic pathway activities in PNPLA3-WT and PNPLA3-I148M hepatocytes. (**C**) Bubble plot showing the flux propensity analysis of reactions in metabolic pathways of human hepatocytes. Cohen’s D statistic was used to compute the fold change difference between the means of 2 groups. The test statistic of the Cohen’s D fold change difference was computed using Wilcoxon’s *P* value adjusted based on Benjamini-Hochberg method. A positive Cohen’s D value indicates a reaction that is relatively more active in Hep-PNPLA3-I148M while a negative value indicates relatively higher activity in Hep-PNPLA3-WT. The size of the bubbles corresponds to the –log10 (Wilcoxon’s *P* value).

To elucidate the genetic and metabolic alterations in human Hep-PNPLA3-I148M, we performed transcriptomic analysis. As compared with Hep-PNPLA3-WT, our results show alterations in the expression of genes involved in several metabolic pathways, including arginine and proline metabolism; cysteine and methionine metabolism; fatty acid degradation; glutathione metabolism; and glycine, serine, and threonine metabolism (Figure 3A). To pinpoint the prevailing metabolic rewiring in Hep-PNPLA3-I148M, we utilized the COMPASS algorithm to integrate transcriptomic data with Recon 2.2, a genome-scale reconstruction model of human metabolism (19) (Figure 3B). The resulting reaction activity scores suggest increased activity in reactions involved in glycolysis/gluconeogenesis (GAPD, r0173, and LDH_L) and metabolic activity that produces glutathione (CYSGLTH) in Hep-PNPLA3-I148M. More importantly, we found an increase in the metabolic activity involved in pyruvate flux into the peroxisome (PYRt2p) (Figure 3C), suggesting that the PNPLA3-I148M variant may be preferentially utilizing the peroxisome over the mitochondria for certain metabolic activities.

### Human Hep-PNPLA3-I148M display increases in peroxisomal β-oxidation.

**Figure 4:**
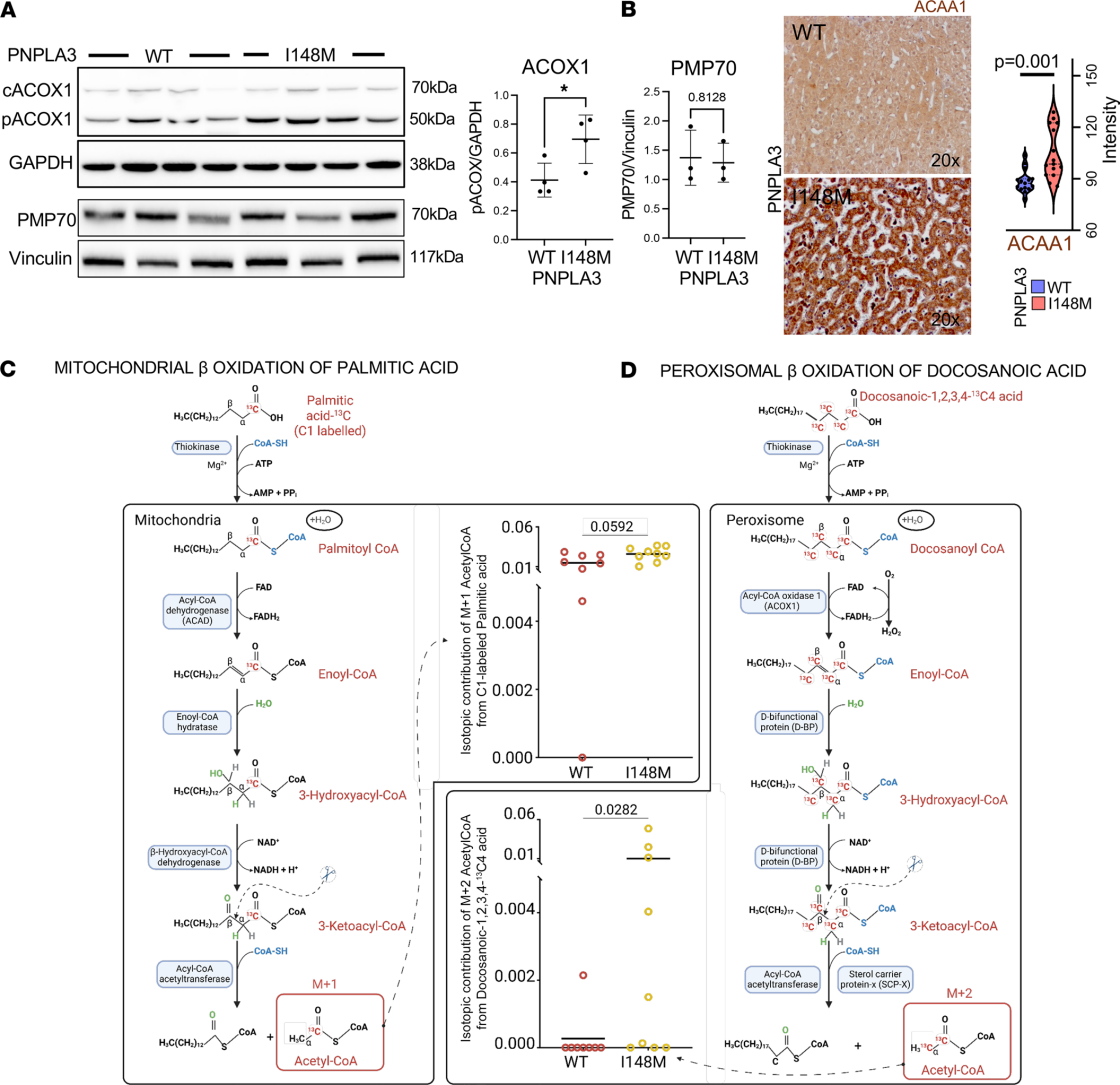
Peroxisomal β-oxidation is increased in human PNPLA3-I148M hepatocytes. (**A**) Western blot analysis of ACOX1 intensity in Hep-PNPLA3-I148M and Hep-PNPLA3-WT. Intensity ratio of the peroxisomal 50 kDA ACOX1 and precursor 70 kDa ACOX1 (*P = 0.05, Welch’s *t* test, WT: *n* = 3, I148M: *n* = 3). PMP70 intensity (^#^*P* = 0.8128, Welch’s *t* test, WT: *n* = 3, I148M: *n* = 3). (**B**) ACAA1 immunohistochemistry signal quantification (*P* = 0.001, Mann-Whitney *U* test, WT: *n* = 3 normal livers/5 different fields each, I418M: *n* = 3 normal livers/5 fields each). (**C**) Schematic of the generation of M+1 Acetyl-CoA from C1-labeled palmitic acid via mitochondrial β-oxidation. Inserted is the scatterplot indicating the enrichment of M+1 Acetyl-CoA in Hep-PNPLA3-I481M extracts (*n* = 9) compared with Hep-PNPLA3-WT (*n* = 8). Horizontal bar indicates the mean and data are analyzed using 2-tailed Mann-Whitney *U* test. (**D**) Schematic of the generation of M+2 Acetyl-CoA from docosanoic-1,2,3,4- ^13^C4 acid via peroxisomal β-oxidation. Inserted is the scatterplot indicating the enrichment of M+2 Acetyl-CoA in Hep-PNPLA3-I481M extracts (*n* = 9) compared with Hep-PNPLA3-WT (*n* = 8). Horizontal bar indicates the mean and data are analyzed using 2-tailed Mann-Whitney *U* test.

One mechanism by which the PNPLA3-I148M variant may be driving alterations in cellular stress is through increased lipid oxidation in either mitochondria or peroxisomes. To corroborate our hypothesis, we examined the expression of peroxisomal acyl-coenzyme A oxidase 1 (ACOX1), an enzyme that catalyzes the rate-limiting step in peroxisomal β-oxidation (20). ACOX1 is synthesized as a 70 kDa precursor protein; within peroxisomes, however, it is processed into a 50 kDa protein (21). We found a significant increase in the ratio of the protein levels of peroxisomal ACOX1 (50 kDa) to precursor ACOX1 (70 kDa) in Hep-PNPLA3-I148M (Figure 4A), indicating increased peroxisomal β-oxidation in Heps-PNPLA3-I148M. Changes in ACOX1 were observed without notable expression changes in peroxisomal membrane protein 70 (PMP70), which transports long-chain fatty acids across the peroxisomal membrane (Figure 4A). In liver tissue, we measured expression of acetyl-coenzyme A acyltransferase 1 (ACAA1), an enzyme involved in fatty acid β-oxidation in peroxisomes, and, like primary hepatocytes, observed increased expression in PNPLA3-I148M tissue when compared with PNPLA3-WT (Figure 4B), indicating increased peroxisomal β-oxidation.

To separately investigate mitochondrial and peroxisomal β-oxidation activities in Hep-PNPLA3-I148M and Hep-PNPLA3-WT cells, we performed stable isotope tracing experiments using C1-labeled palmitic acid, a long-chain fatty acid (LCFA) oxidized primarily via mitochondrial β-oxidation, and docosanoic-1,2,3,4-^13^C4 acid, a very-long-chain fatty acid (VLCFA) preferred by peroxisomal β-oxidation, and measured the resulting labeled acetyl-CoA (22). Our results showed a similar enrichment of M+1 acetyl-CoA originating from C1-labeled palmitic acid in Hep-PNPLA3-I148M and Hep-PNPLA3-WT (Figure 4C). It has been reported that when mitochondrial β-oxidation is impaired, peroxisomes can oxidize medium-chain fatty acids (MCFAs) and LCFAs, including palmitic acid (23). Hence, we posited that, for C1-labeled palmitic acid, peroxisomal fatty acid oxidation compensated for the impaired mitochondrial fatty acid oxidation in Hep-PNPLA3-I148M, which explains why there is no difference in the enrichment of M+1 acetyl-CoA in the 2 groups. Compared with Hep-PNPLA3-WT, our results showed a significant enrichment of M+2 acetyl-CoA from the docosanoic-1,2,3,4-^13^C4 acid tracing experiment (Figure 4D) in Hep-PNPLA3-I148M, further corroborating our hypothesis that human Hep-PNPLA3-I148M have upregulated peroxisomal β-oxidation.

**Figure 5:**
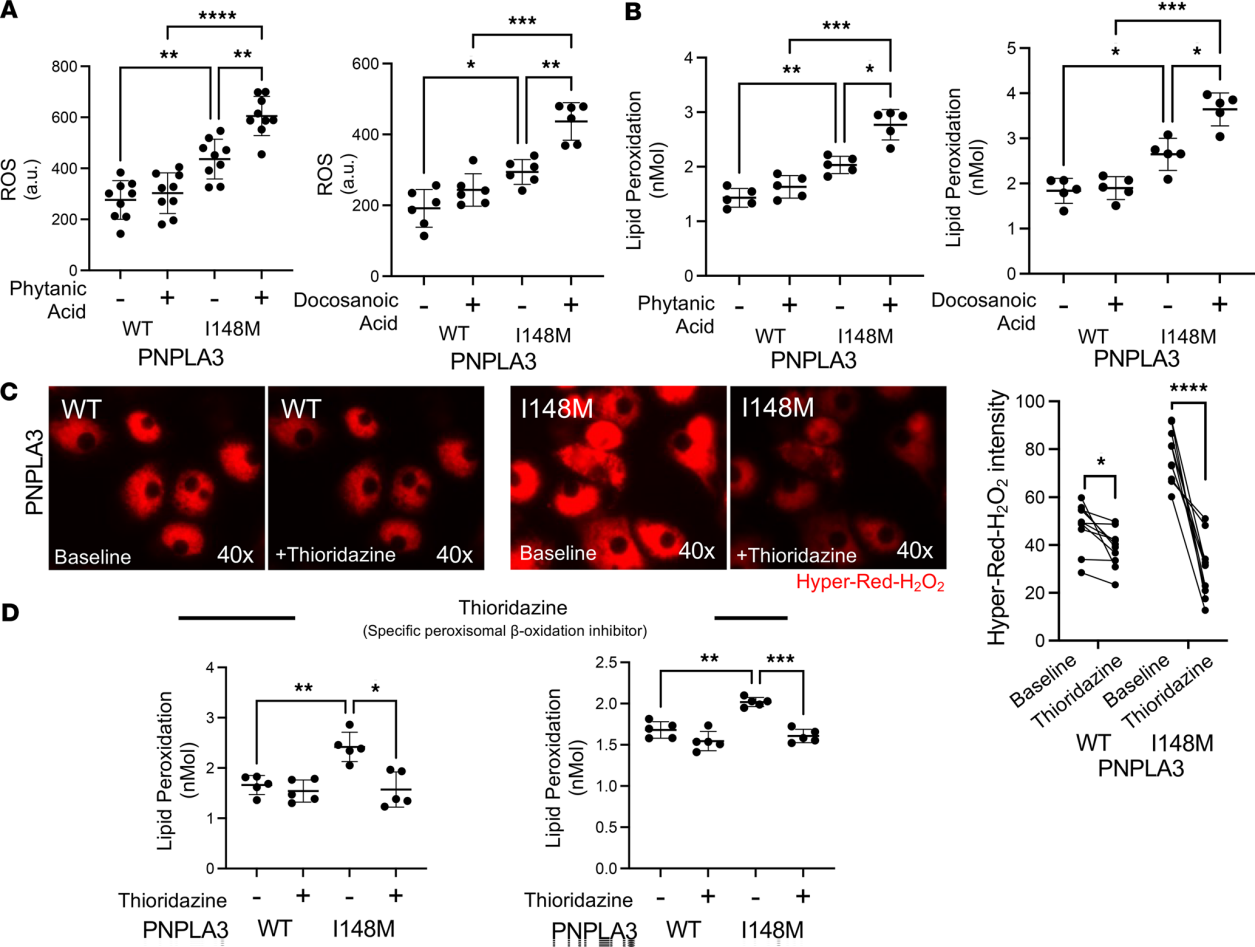
Functional assessment of peroxisomal β-oxidation in human PNPLA3-I148M hepatocytes. (**A**) Total ROS measurement in primary human hepatocytes resulting from peroxisomal β-oxidation following phytanic acid (*n* = 9) and docosanoic acid exposure (*n* = 6, *P < 0.05, **P < 0.01, ***P < 0.001, ****P < 0.0001, 1-way ANOVA). (**B**) Lipid peroxidation measurement in primary hepatocytes resulting from peroxisomal β-oxidation following phytanic acid (*n* = 5) and docosanoic acid exposure (*n* = 5, *P < 0.05, **P < 0.01, ***P < 0.001, 1-way ANOVA). (**C**) Fluorescent micrographs of HyPer-Red in transiently transfected primary hepatocytes exposed to thioridazine for 1 hour between micrographs. Quantification of H_2_O_2_ by HyPer-Red intensity (*P < 0.05, ****P < 0.001, Welch’s *t* test, WT: *n* = 15 [5 cells per case], I148M: 15 [5 cells per case]). (**D**) Lipid peroxidation suppression in primary hepatocytes exposed to thioridazine and treated with phytanic acid or docosanoic acid (*P < 0.05, **P < 0.01, ***P < 0.001, 1-way ANOVA, WT: *n* = 5, I148M: *n* = 5).

To validate these observations, we predicted that increased peroxisomal β-oxidation would result in an increase in ROS production and cellular stress in Hep-PNPLA3-I148M. Given that peroxisomes are highly abundant in hepatocytes and involved in β-oxidation of fatty acids (24), we hypothesized that the observed decrease in mitochondrial β-oxidation leads to increased peroxisomal β-oxidation and ROS production (24). We treated Hep-PNPLA3-I148M and Hep-PNPLA3-WT cells with docosanoic acid and phytanic acid, an odd-chained fatty acid that undergoes α-oxidation exclusively in peroxisomes (25), and measured ROS production and lipid peroxidation. As expected, ROS production (Figure 5A) and lipid peroxidation (Figure 5B) were significantly increased in Hep-PNPLA3-I148M when compared with Hep-PNPLA3-WT controls. These findings support our hypothesis that Hep-PNPLA3-I148M cells rely on increased peroxisomal β-oxidation and that impairment of this process results in accumulation of intracellular VLCFAs, thereby increasing cellular stress (26).

We then assessed the role of peroxisomal β-oxidation in the production of ROS by measuring H_2_O_2_ production following treatment with thioridazine, an inhibitor of peroxisomal β-oxidation (27, 28). We found a significant decrease in H_2_O_2_ in the presence of thioridazine in Hep-PNPLA3-I148M when compared with untreated cells (Figure 5C and Supplemental Figure 2). To dissect the individual contributions of peroxisomes and mitochondria to the ROS pool in Hep-PNPLA3-I148M, we treated the cells with thioridazine or etomoxir, a well-known inhibitor of β-oxidation in mitochondria. Our results showed that approximately 20% of intracellular ROS originated from peroxisomal β-oxidation, while about 9% was derived from mitochondria (Supplemental Figure 3). We then repeated our phytanic or docosanoic acid exposure in the presence of thioridazine and observed a significant suppression of lipid peroxidation in both treatment conditions in Hep-PNPLA3-I148M cells (Figure 5D). Finally, using CRISPR/Cas9 in a human hepatoma cell line (HepG2), we generated HepG2-PNPLA3-I148M and isogenic gene-edited control HepG2-PNPLA3-WT^Cas9^ (Supplemental Figure 2) and assessed ROS production and lipid peroxidation in the presence of thioridazine. Like our previous results, thioridazine treatment reduced H_2_O_2_ in both genotypes and suppressed lipid peroxidation, with a greater magnitude of change observed in HepG2-PNPLA3-I148M than control HepG2-PNPLA3-WT^Cas9^ (Supplemental Figure 2). Taken together, we conclude that the PNPLA3-I148M variant increases levels of β-oxidation in peroxisomes.

### PNPLA3-I148M increases ferroptosis-associated processes in primary human hepatocytes.

**Figure 6:**
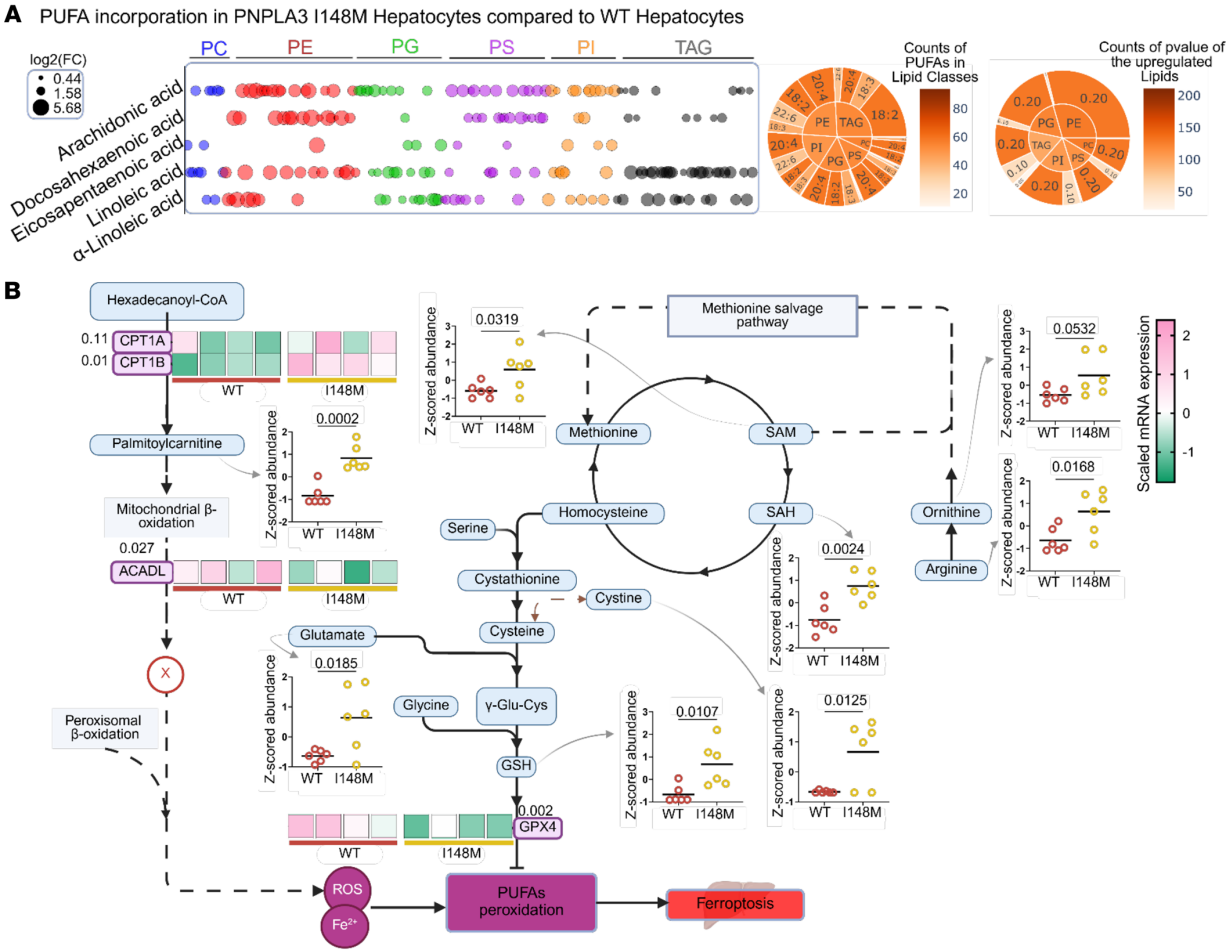
PUFA-enriched lipid reprogramming links PNPLA3-I148M to ferroptosis. (**A**) Bubble plot of the cell membrane and lipid droplet-associated lipid classes that are upregulated in Hep-PNPLA3-I148M compared with the Hep-PNPLA3-WT. The plot also shows the polyunsaturated fatty acids (PUFAs) attached to the backbone of individual lipids. Only species with *P* values less than 0.2 are included. Bubble size indicates the log_2_ fold change of upregulated lipid species. Bubble color corresponds to the different lipid classes. The inserted sunburst plots show the counts of each PUFA in the lipid classes and the distribution of the statistical significance of the upregulation. (**B**) Untargeted metabolomic profiles comparing Hep-PNPLA3*-*I148M and Hep-PNPLA3-WT (WT: *n* = 6, I148M: *n* = 6) coupling transcriptomic analysis of related gene expression (WT: *n* = 4, I148M: *n* = 4). For transcriptomic data, the test statistics were computed based on Wald test statistics while for the metabolomics data the test statistics were computed using MetaboAnalyst (ver. 4.0).

It has been reported that susceptibility of cells to lipid peroxidation is directly proportional to the amount of polyunsaturated fatty acids (PUFAs) contained within membrane-bound phospholipids and lipid droplet-associated TGs (29). Moreover, it is well known that hepatic TGs are enriched in PUFAs in carriers of the PNPLA3-I148M variant (30). Given our earlier findings, we investigated the mechanisms contributing to a unique cell death modality that results from the interaction between cellular amino acids, lipids, iron, and ROS (31) known as ferroptosis. Using lipidomic analysis, we assessed the cell membrane and lipid droplet-associated lipid classes that were upregulated in Hep-PNPLA3-I148M cells and observed increased PUFA incorporation in Hep-PNPLA3-I148M compared with WT controls (Figure 6A).

Our integrated approach encompassing transcriptomics and metabolomics revealed substantially rewired metabolic pathways that culminated in increased reduced glutathione (GSH) in primary Hep-PNPLA3-I148M (Figure 6B and Supplemental Figure 4). This increase in GSH results from upregulation of metabolites in the methionine cycle, supplemented by arginine metabolism in the methionine salvage pathway (Figure 6B). Our transcriptomic analysis also shows a substantial reduction in the expression of glutathione peroxidase 4 (*GPX4*) in Hep-PNPLA3-I148M (Figure 6B and Supplemental Figure 5), which has been previously associated with increased ferroptosis susceptibility (32). Furthermore, we found altered expression of genes related to iron metabolism that can increase intracellular iron in Hep-PNPLA3-I148M (Supplemental Figure 5).

**Figure 7:**
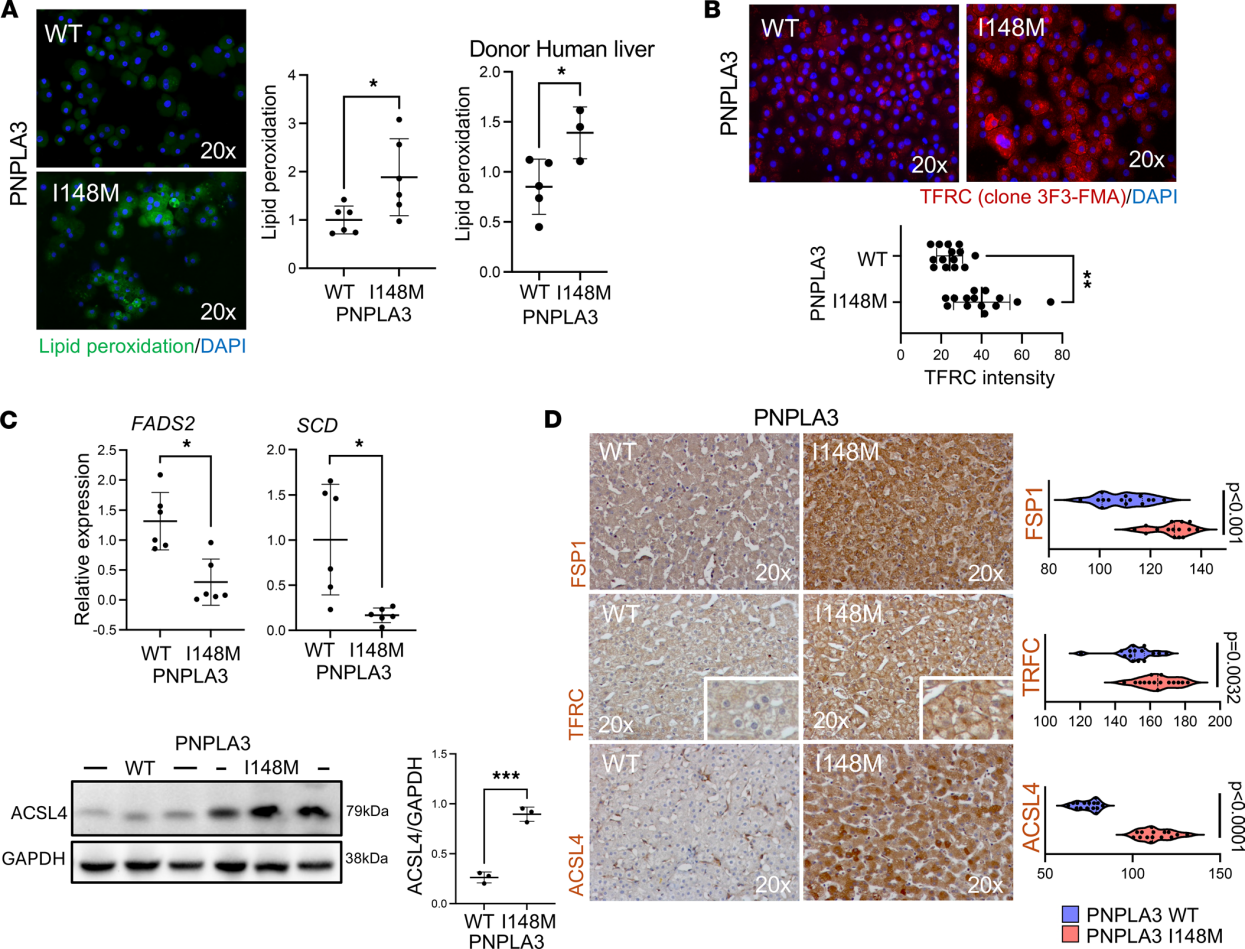
Functional characterization of ferroptosis in primary human hepatocytes. (**A**) Lipid peroxidation in human hepatocytes shown by representative C11-BODIPY (581/591) micrographs and malondialdehyde quantification (*P = 0.04, Welch’s *t* test, WT: *n* = 6, I148M: *n* = 6). Lipid peroxidation measurement in donor liver tissue (*P = 0.0427, Welch’s *t* test, WT: *n* = 5, I148M: *n* = 3). (**B**) TFRC staining micrographs and intensity quantification in Hep-PNPLA3-WT and Hep-PNPLA3-I148M (**P = 0.0011, Mann-Whitney test, WT: *n* = 14, I148M: *n* = 14). (**C**) Quantitative gene expression analysis of *FADS2* and *SCD* in hepatocytes normalized to *ACTB* (*P = 0.02, *P* = 0.28, *P = 0.02, Welch’s *t* test, WT: *n* = 6, I148M: *n* = 6). Western blot analysis of ACSL4 intensity normalized to GAPDH (***P = 0.0004, Welch’s *t* test, WT: *n* = 3, I148M: *n* = 3). (**D**) Histological micrographs (FSP1, TFRC, and ACSL4) of donor livers. Immunohistochemistry signal quantification (FSP1: *P* < 0.001, TFRC: *P* = 0.0032, ACSL4: *P* < 0.0001, Welch’s *t* test, WT: *n* = 3 normal livers/5 different fields each, I418M: *n* = 3 normal livers/5 fields each).

To further assess ferroptosis activity, we measured lipid peroxidation in primary human hepatocytes and liver tissue and observed a significant increase in lipid peroxidation in Hep-PNPLA3-I148M when compared with Hep-PNPLA3-WT, as well as in PNPLA3-I148M liver tissue when compared with control (Figure 7A). Similarly, expression of TFRC, which performs a critical role in cellular iron uptake, was greater in Hep-PNPLA3-I148M cells (Figure 7B). To examine potential alterations in lipid synthesis that could contribute to ferroptosis, we investigated the expression of metabolic enzymes associated with susceptibility to ferroptosis. Notably, we found significantly decreased expression of stearoyl CoA desaturase (*SCD*) and fatty acid desaturase 2 (*FADS2*), along with a significant upregulation of acyl-CoA synthetase long-chain family member 4 (ACSL4) in Hep-PNPLA3-I148M (Figure 7C). Finally, we assessed expression of TFRC and ACSL4, as well as ferroptosis suppressor protein 1 (FSP1), in liver tissue and observed a significant increase in expression of all 3 proteins in PNPLA3-I148M (Figure 7D). Treatment with thioridazine reduced lipid peroxidation, TFRC expression, and mitochondrial oxidation by 20%–25%, indicating that peroxisomal β-oxidation is a contributor, but not the sole driver, of ferroptosis (Supplemental Figure 6). Taken together, these data demonstrate that alterations in the prevailing lipid homeostasis driven by the PNPLA3-I148M variant can increase the incidence of lipid peroxidation partially through peroxisomal fatty acid β-oxidation, drive mitochondrial and transcriptomic alterations, and induce changes in mobilization of TFRC.

### Pharmacological inhibition of ferroptosis suppresses cellular stress and cell death in human Hep-PNPLA3 I148M.

**Figure 8:**
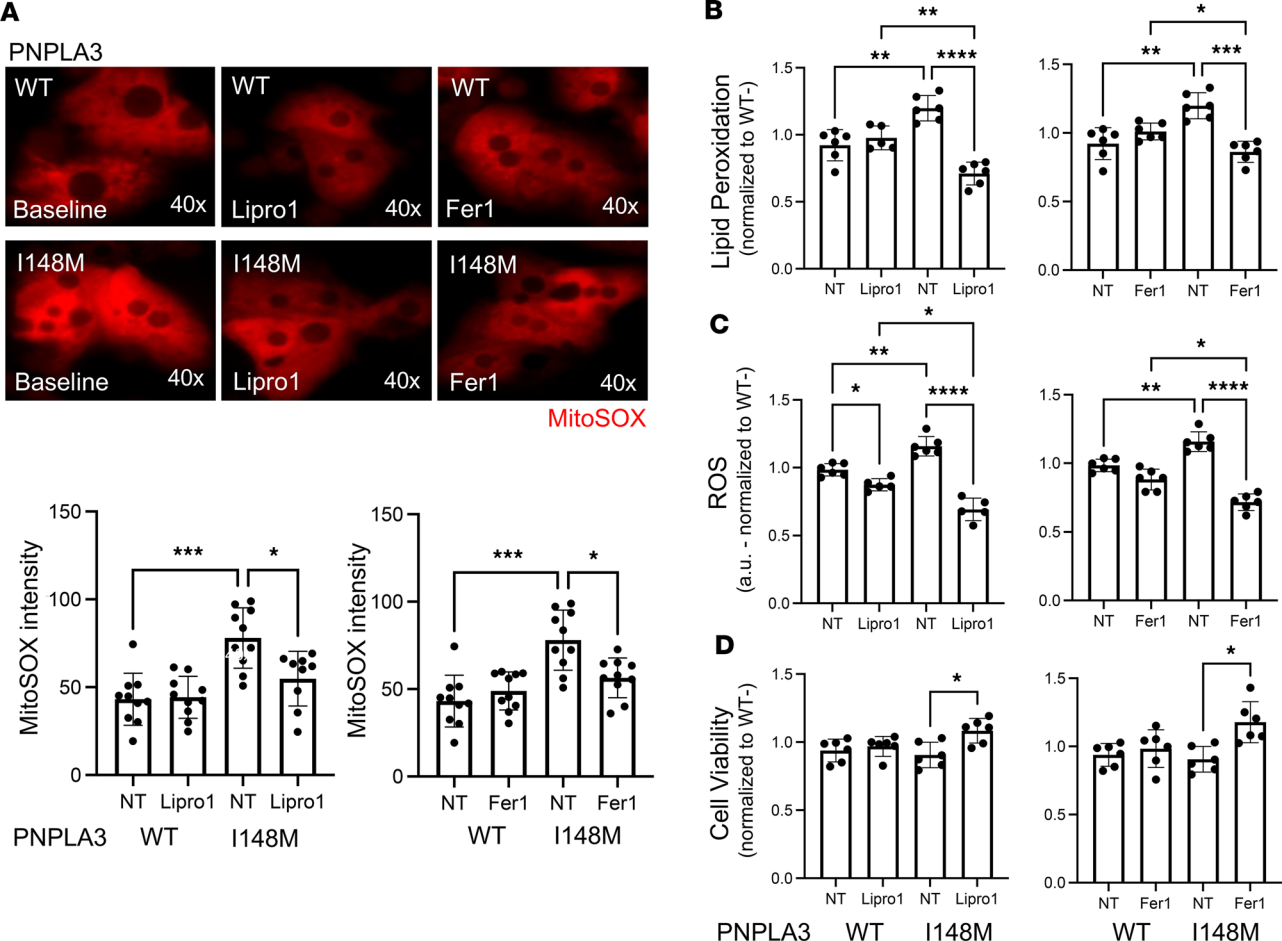
The ferroptosis inhibitors Lipro1 and Fer1 suppress cellular stress and cell death in human PNPLA3-I148M hepatocytes. MitoSOX micrographs and quantification, lipid peroxidation, ROS measurement, and viability of human hepatocytes in the presence of Lipro1 or Fer1. (**A**) MitoSOX (*P < 0.05, ***P < 0.001, ****P < 0.0001, 1-way ANOVA, WT: *n* = 10, I148M: *n* = 10), (**B**) lipid peroxidation (*P < 0.05, **P < 0.01, ***P < 0.001, ****P < 0.0001, 1-way ANOVA, WT: *n* = 6, I148M: *n* = 6), (**C**) ROS (*P < 0.05, **P < 0.01, ****P < 0.0001, 1-way ANOVA, WT: *n* = 6, I148M: *n* = 6), and (**D**) cell viability (*P < 0.05, 1-way ANOVA, WT: *n* = 6, I148M: *n* = 6).

To determine how blockage of ferroptosis affects cell viability in the presence of the PNPLA3-I148M variant, we treated human Hep-PNPLA3-WT and Hep-PNPLA3-I148M cells with the specific ferroptosis inhibitors liproxstatin-1 (Lipro1) and ferrostatin-1 (Fer1) and assessed mitochondrial oxidation, lipid peroxidation, ROS production, and cell death. Treatment of Hep-PNPLA3-I148M cells with Lipro1 or Fer1 led to a significant reduction in mitochondrial superoxide (Figure 8A). Additionally, treatment with the ferroptosis inhibitors led to a significantly greater suppression of lipid peroxidation (Figure 8B), ROS production (Figure 8C), and cell death (Figure 8D) in Hep-PNPLA3-I148M compared with Hep-PNPLA3-WT cells, suggesting that inhibition of ferroptosis overcomes the consequences associated with the PNPLA3-I148M variant. Interestingly, exposure of Hep-PNPLA3-I148M cells to ferroptosis inhibitors did not reduce ER stress (Supplemental Figure 7). We hypothesize that targeting lipid peroxidation alone is insufficient to decrease the intracellular lipid accumulation driven by the PNPLA3 mutation, which is the underlying source of ER stress.

**Figure 9:**
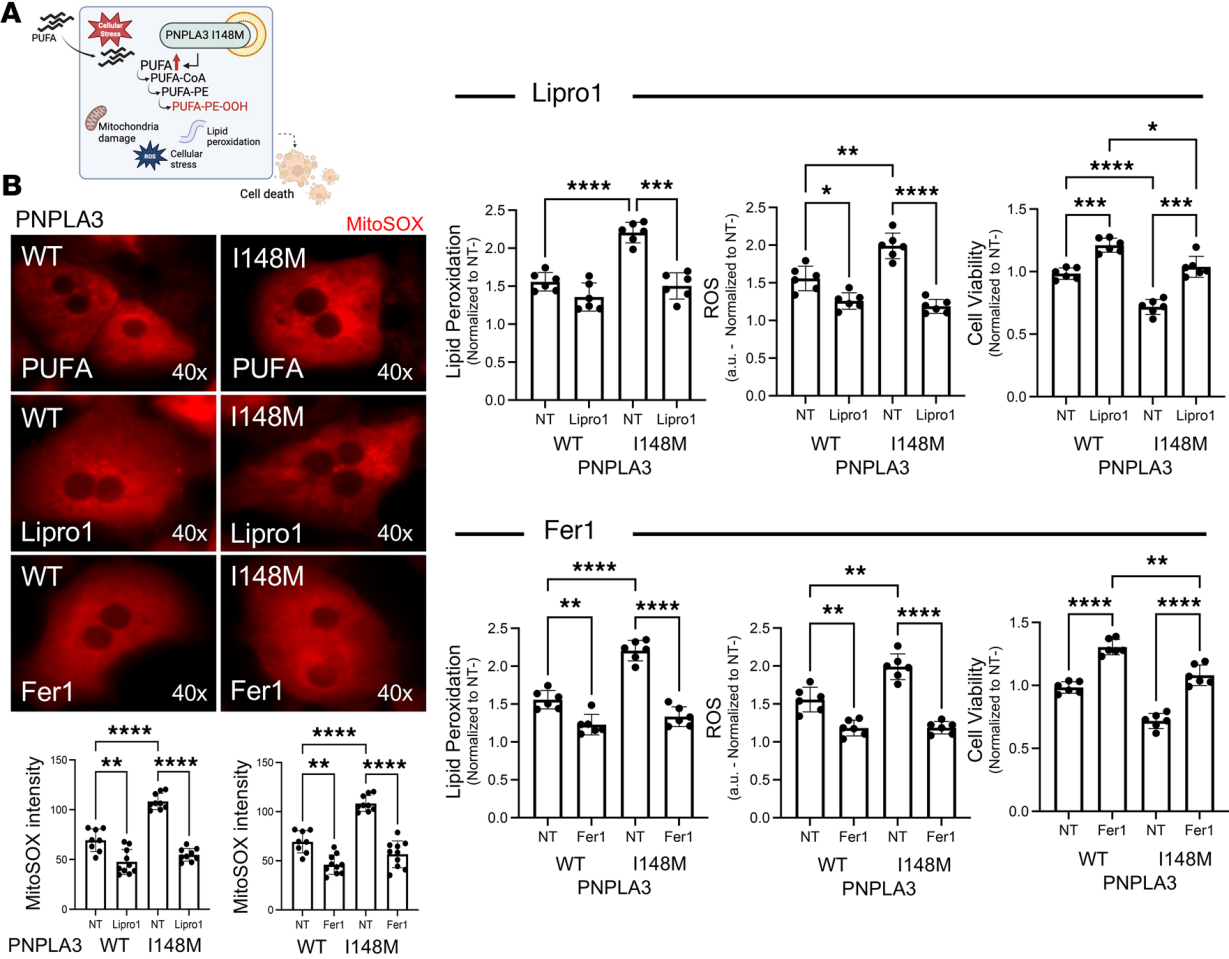
Lipro1 and Fer1 suppress cellular stress and cell death in human PNPLA3-I148M hepatocytes under PUFA stress. (**A**) PNPLA3-I148M leads to intracellular accumulation of PUFAs that are more susceptible to peroxidation, increasing ferroptosis and cell death. (**B**) MitoSOX micrographs and quantification, lipid peroxidation, ROS measurement, and viability of hepatocytes in the presence of Lipro1 or Fer1 after exposing hepatocytes to PUFAs. (MitoSOX: *n* = 9, lipid peroxidation: *n* = 6, ROS: *n* = 6 and cell viability: *n* = 6, *P < 0.05, **P < 0.01, ***P < 0.001, ****P < 0.0001, 1-way ANOVA.)

As noted previously, we observed a greater presence of PUFAs in Hep-PNPLA3-I148M cells compared with Hep-PNPLA3-WT, and this intracellular accumulation increases susceptibility to ferroptosis (Figure 9A). We, therefore, exposed WT and variant cells to elevated levels of PUFAs and assessed whether treatment with Lipro1 or Fer1 would attenuate the effects. In the presence of PUFAs alone, we observed elevated levels of stress in Hep-PNPLA3-I148M compared with Hep-PNPLA3-WT cells, indicating that the PNPLA3-I148M variant increases susceptibility to stress-related outcomes (Figure 9B). When exposed to PUFAs in conjunction with a ferroptosis inhibitor, both WT and variant Heps displayed significant attenuations of mitochondrial oxidation, lipid peroxidation, ROS production, and cell death (Figure 9B). This suppression was significantly greater in Hep-PNPLA3-I148M than Hep-PNPLA3-WT cells. These results indicate that PNPLA3-I148M is related to susceptibility to ferroptosis, and treatment with ferroptosis inhibitors can diminish the negative effects on cellular fitness associated with the PNPLA3-I148M variant.

### Abrogation of ferroptosis-driven cellular stress in human Hep-PNPLA3-I148M increases cell survival.

**Figure 10:**
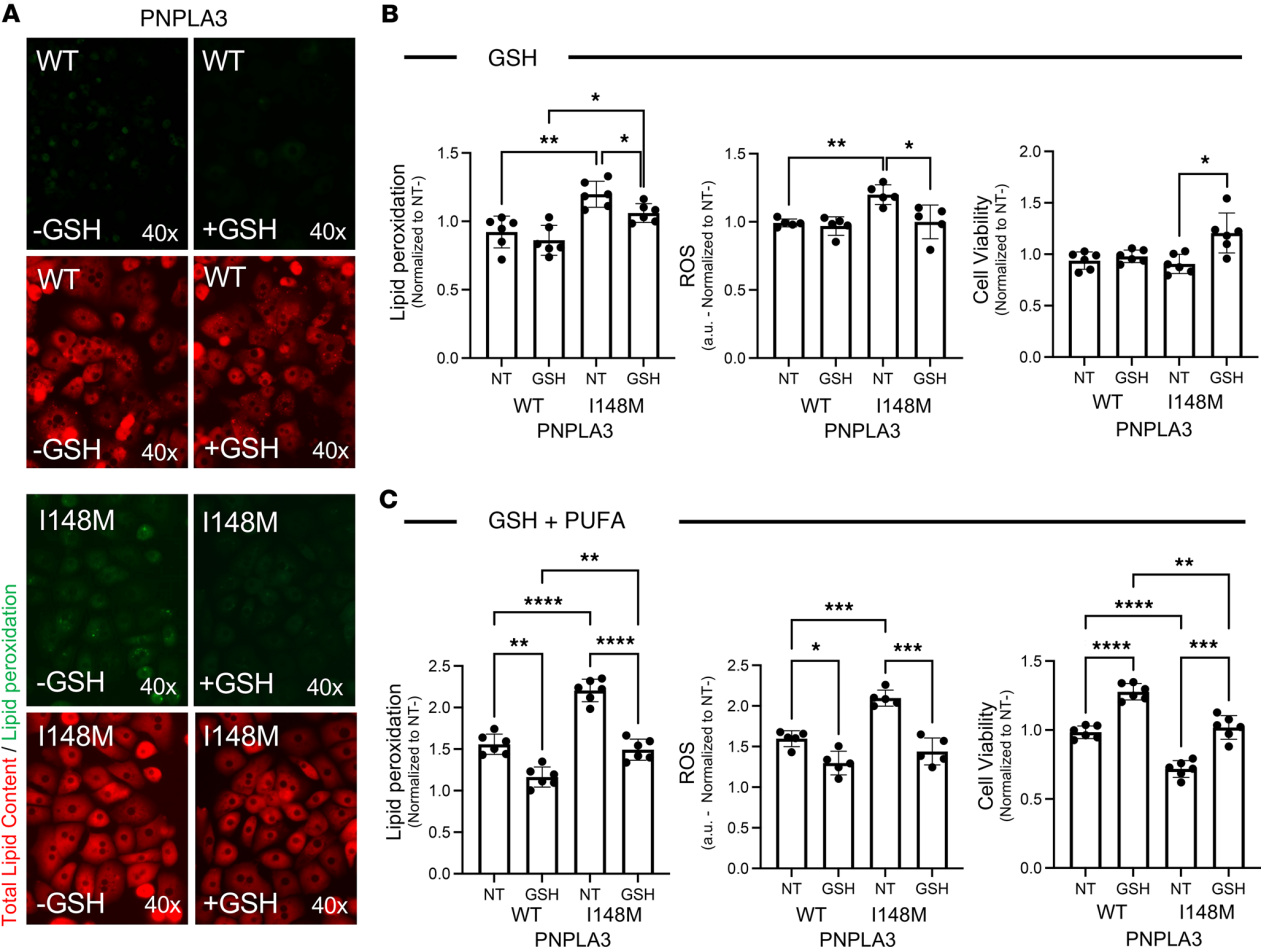
Programmed cell death is attenuated in primary human hepatocytes carrying the PNPLA3-I148M variant by reducing lipid peroxidation and oxidative stress. (**A**) C11-BODIPY micrographs of lipid peroxidation in hepatocytes cultured in the presence of GSH for 24 hours. (**B**) Suppression of lipid peroxidation, ROS, and cell death in primary hepatocytes exposed to GSH for 24 hours. (**C**) Evaluation of lipid peroxidation, ROS, and cell death suppression after GSH supplementation and PUFA exposure (lipid peroxidation: *n* = 6, ROS: *n* = 5, and cell viability: *n* = 6, *P < 0.05, **P < 0.01, ***P < 0.001, ****P < 0.0001, 1-way ANOVA).

**Figure 11:**
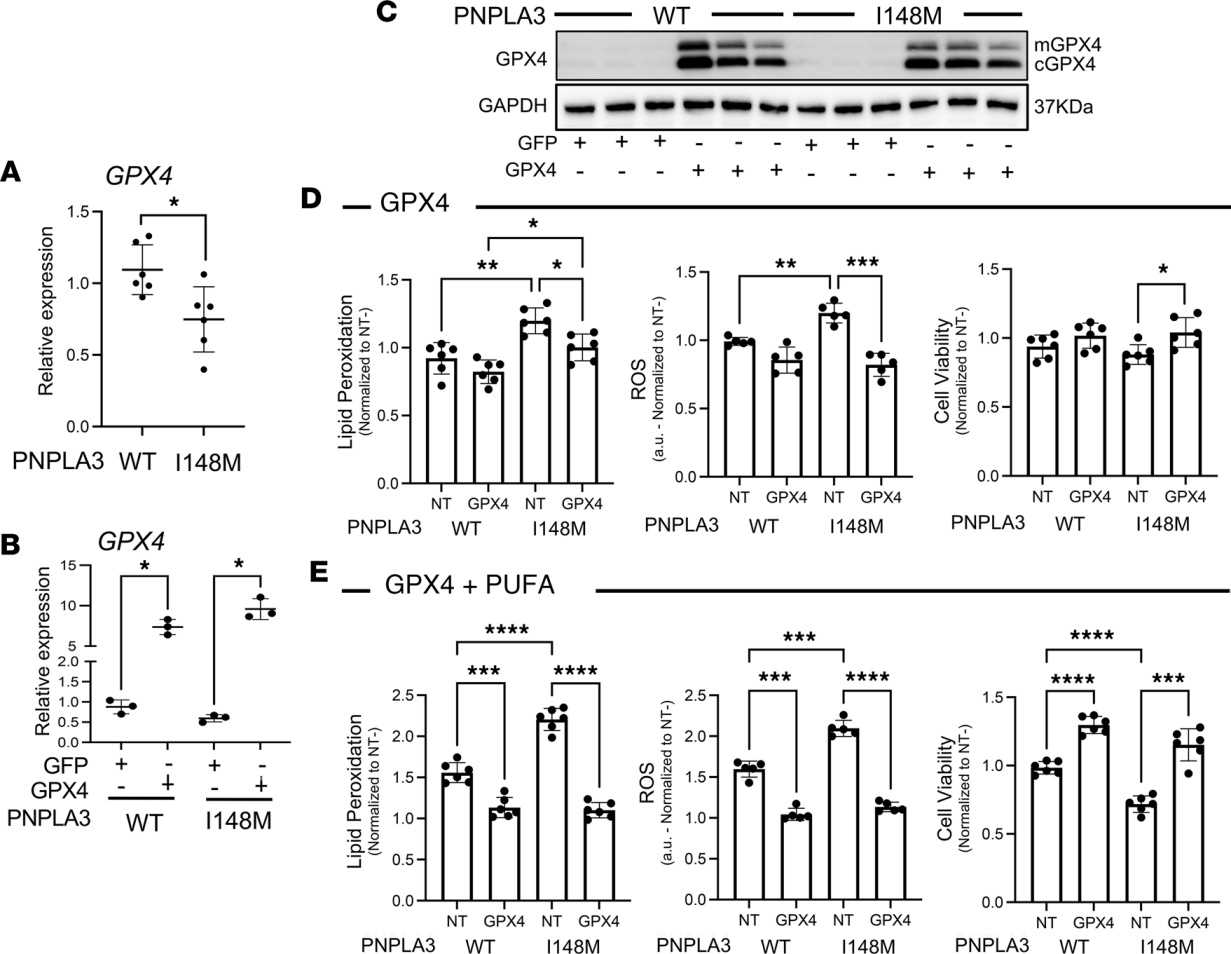
Restoration of GPX4 expression attenuates programmed cell death in primary human hepatocytes carrying the PNPLA3-I148M variant by reducing lipid peroxidation and oxidative stress. (**A**) Quantitative gene expression analysis of *GPX4* in hepatocytes normalized to *ACTB* (*P = 0.02, Welch’s *t* test, WT: *n* = 6, I148M: *n* = 6). (**B**) Quantitative gene expression of *GPX4* 48 hours after viral transduction (*P < 0.03, Welch’s ANOVA, WT: *n* = 3, I148M: *n* = 3). (**C**) GPX4 protein expression after lentivirus transduction. (**D**) Suppression of lipid peroxidation, ROS, and cell death in primary hepatocytes after GPX4 overexpression. (**E**) Lipid peroxidation, ROS, and cell death suppression in hepatocytes exposed to PUFAs. (Lipid peroxidation: *n* = 6, ROS: *n* = 5, and cell viability: *n* = 6, *P < 0.05, **P < 0.01, ***P < 0.001, ****P < 0.0001, 1-way ANOVA.)

While our current studies were performed in human hepatocytes from patients with no history of hepatic disorders, marked metabolomic alterations were observed between WT or PNPLA3-I148M MASH patients, highlighting the need for personalized therapeutic strategies (Supplemental Figure 8). Our findings suggest that strategies targeting ferroptosis susceptibility may normalize hepatocellular fitness. To test this hypothesis, we evaluated 2 approaches: i) nutritional supplementation using GSH and ii) a gene modulation approach designed to increase GPX4 expression (Figures 10 and 11).

First, we treated Hep-PNPLA3-WT and Hep-PNPLA3-I148M cells with GSH, which reduces ROS and lipid peroxidation by binding to free cytosolic iron (Fe^2+^) (33). Treatment with GSH led to a suppression of lipid peroxidation, ROS production, and cell death in both WT and variant cells; however, the extent of suppression was greater in Hep-PNPLA3-I148M than Hep-PNPLA3-WT (Figure 10, A and B). Additionally, cells were exposed to PUFAs to induce cellular stress, a phenomenon that we observed to be exacerbated by the presence of the PNPLA3-I148M variant. In both Hep-PNPLA3-WT and Hep-PNPLA3-I148M cells, concurrent administration of GSH and PUFAs led to an improvement of all measures of cellular fitness (Figure 10C). The magnitude of the improvement in PUFA-treated Hep-PNPLA3-I148M cells was far greater than that observed in Hep-PNPLA3-WT cells, indicating that GSH supplementation can abrogate cellular stress-induced processes.

Next, we employed a gene modulation approach to attenuate PNPLA3-I148M–induced metabolic changes. GPX4 is an enzyme that reduces lipid peroxides at the expense of GSH. It has been previously shown that lower expression of GPX4 leads to increased ferroptosis susceptibility (32). As previously shown, Hep-PNPLA3-I148M cells had significantly reduced expression of *GPX4* compared with Hep-PNPLA3-WT (Figure 11A). Consequently, we treated Hep-PNPLA3-I148M and Hep-PNPLA3-WT with a lentiviral vector encoding *GPX4* to evaluate its role in cell viability and hypothesized that overexpression would enable these cells to utilize endogenous GSH and prevent ferroptosis. We observed a significant increase in GPX4 expression in both groups following lentiviral transfection (Figure 11, B and C, and Supplemental Figure 9). As with the findings following GSH supplementation, GPX4 overexpression led to a significant suppression of lipid peroxidation, ROS production, and cell death in both groups, with a greater extent of suppression observed in Hep-PNPLA3-I148M cells (Figure 11D). Similarly, GPX4 overexpression was able to combat the cellular stress induced by PUFA exposure in Hep-PNPLA3-I148M cells (Figure 11E). Taken together, our data demonstrate that ferroptosis is a viable therapeutic target in human Hep-PNPLA3-I148M, and personalized therapies inhibiting ferroptosis may serve to relieve cellular stress and ferroptosis sensitivity through a reduction in lipid peroxidation.

### iHeps possessing the PNPLA3-I148M mutation replicate ferroptosis alterations.

**Figure 12:**
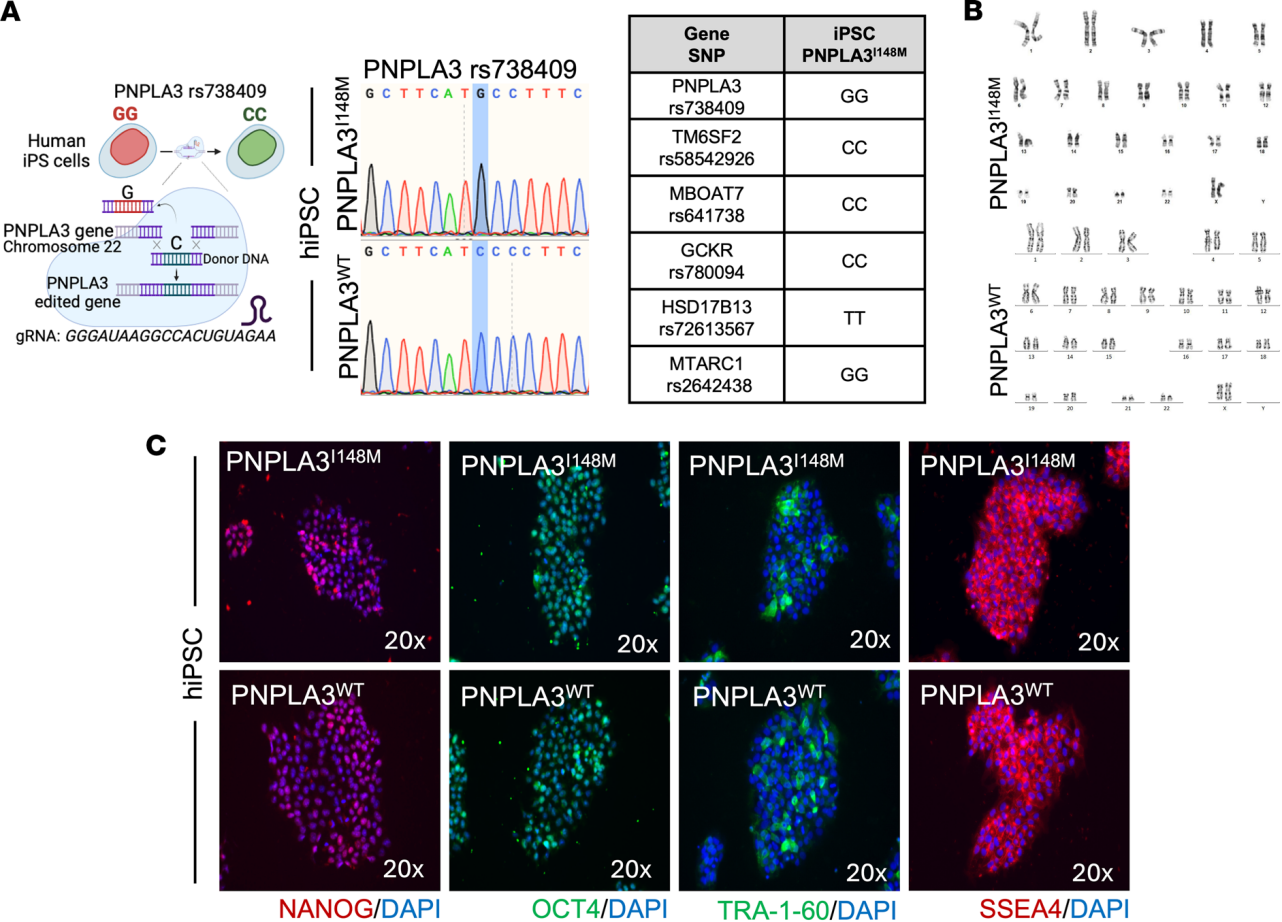
Generation and characterization of iPSC-PNPLA3-I148M and iPSC-PNPLA3-WT. (**A**) Schematic design of CRISPR/Cas9 gene editing to generate human iPSC-PNPLA3-WT from an iPSC carrying the PNPLA3-I148M mutation (iPSC-PNPLA3-I148M). The Sanger sequence for the *PNPLA3* gene confirmed that iPSC-PNPLA3-I148M cells are minor homozygous (GG) and the iPSC-PNPLA3-WT cells are major homozygous (CC) after gene editing, as highlighted in blue. iPSC-PNPLA3-I148M do not carry any additional well-known gene mutations linked with the development of fatty liver disease. (**B**) G-banding analysis for karyotype in both iPSC-PNPLA3-WT and iPSC-PNPLA3-I148M shows no abnormalities. (**C**) Immunofluorescence micrographs of the pluripotency markers NANOG, SSEA4, OCT, and TRA-1-60 showing their stemness status.

**Figure 13:**
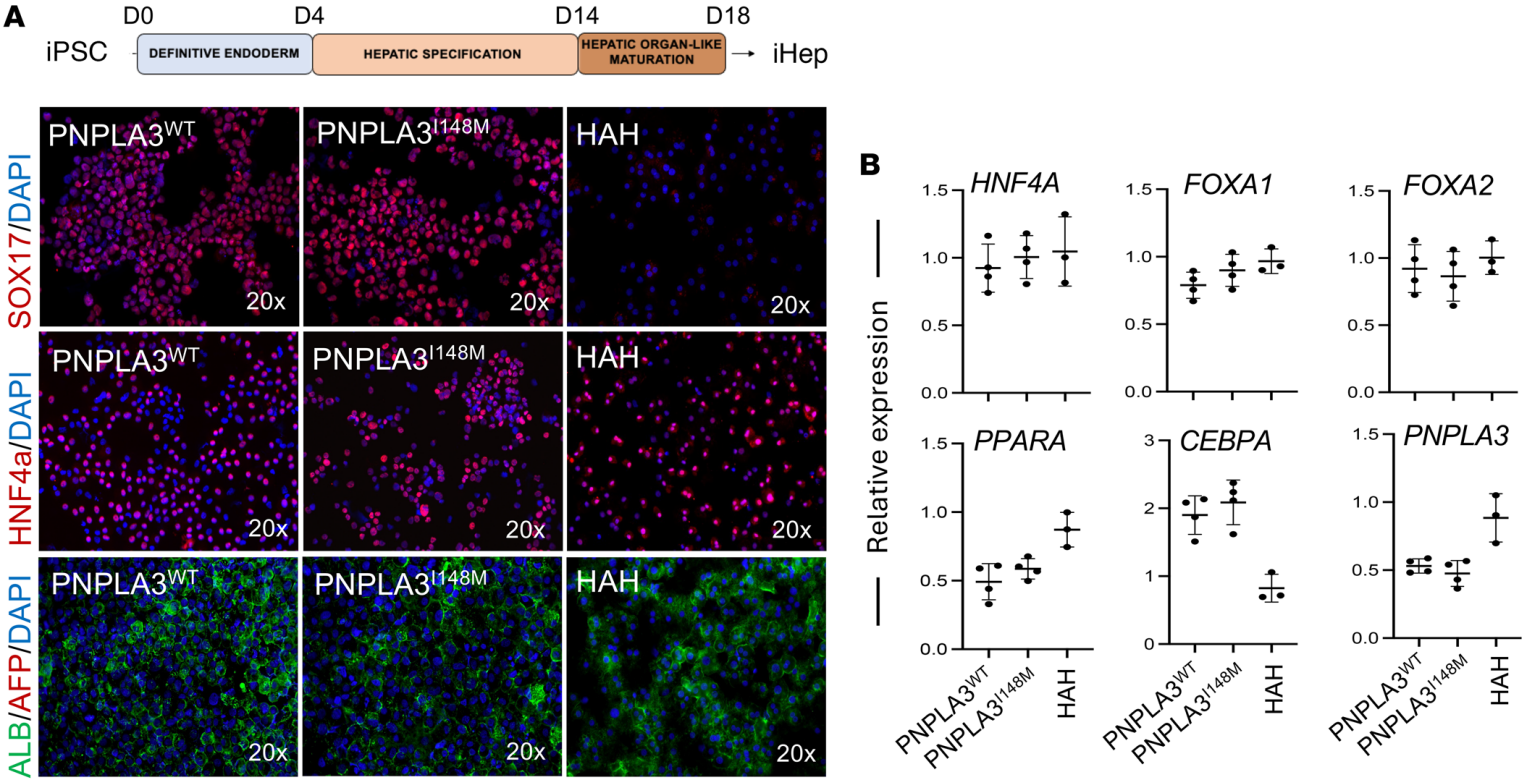
Hepatic differentiation and characterization of iHep-PNPLA3-WT and iHep-PNPLA3-I148M. (**A**) Schematic illustration of the hepatocyte differentiation protocol. Immunofluorescence micrographs of endoderm marker SOX17 in iDE-PNPLA3^WT^ and iDE-PNPLA3^I148M^ at D4, and immunofluorescence micrographs of hepatocyte markers, adult isoform HNF4A, AFP, and albumin in iHep-PNPLA3-WT and iHep-PNPLA3-I148M. (**B**) Quantitative gene expression for hepatocyte markers: *HNF4A*, *FOXA1*, *FOXA2*, *PPARA*, *CEBPA*, and *PNPLA3* in iHep-PNPLA3-WT (*n* = 4) and iHep-PNPLA3-I148M (*n* = 4). Human adult hepatocytes (HAH) were used as positive control.

To validate our findings in a second model, we generated iHeps from iPSCs obtained from healthy individuals possessing the PNPLA3-I148M variant (iHep-PNPLA3-I148M). This model presents many advantages over primary human cells. These nascent cells have not been impacted by an individual’s lifestyle, and they present a platform for the study of cellular development. After obtaining iPSCs with the I148M variant, we performed CRISPR/Cas9 genome editing to correct the PNPLA3 sequence, allowing us to evaluate WT and mutant cells on the same genetic background (Figure 12A). The pluripotency markers NANOG, SSEA4, OCT4, and TRA-1-60 were used to determine stemness status, and karyotype was analyzed using G-banding. No abnormalities were detected in either iPSC-PNPLA3-I148M or isogeneic gene-edited control iPSC-PNPLA3-WT cells (Figure 12, B and C). iPSCs then underwent hepatocyte-directed differentiation as previously described (34). The endoderm marker SOX17, adult isoform HNF4A, and albumin were present, without notable expression of alpha-fetoprotein (AFP), in both WT and variant cells during the differentiation process, and no differences were observed between iHep-PNPLA3-WT, iHep-PNPLA3-I148M, or HAHs (Figure 13A). The resulting iHep-PNPLA3-WT and iHep-PNPLA3-I148M cells displayed similar expression of hepatocyte-specific genes (*HNF4A*, *FOXA1*, *FOXA2*, *PPARA*, and *CEBPA*) and *PNPLA3*, with no differences observed between the genotypes or HAHs (Figure 13B).

**Figure 14:**
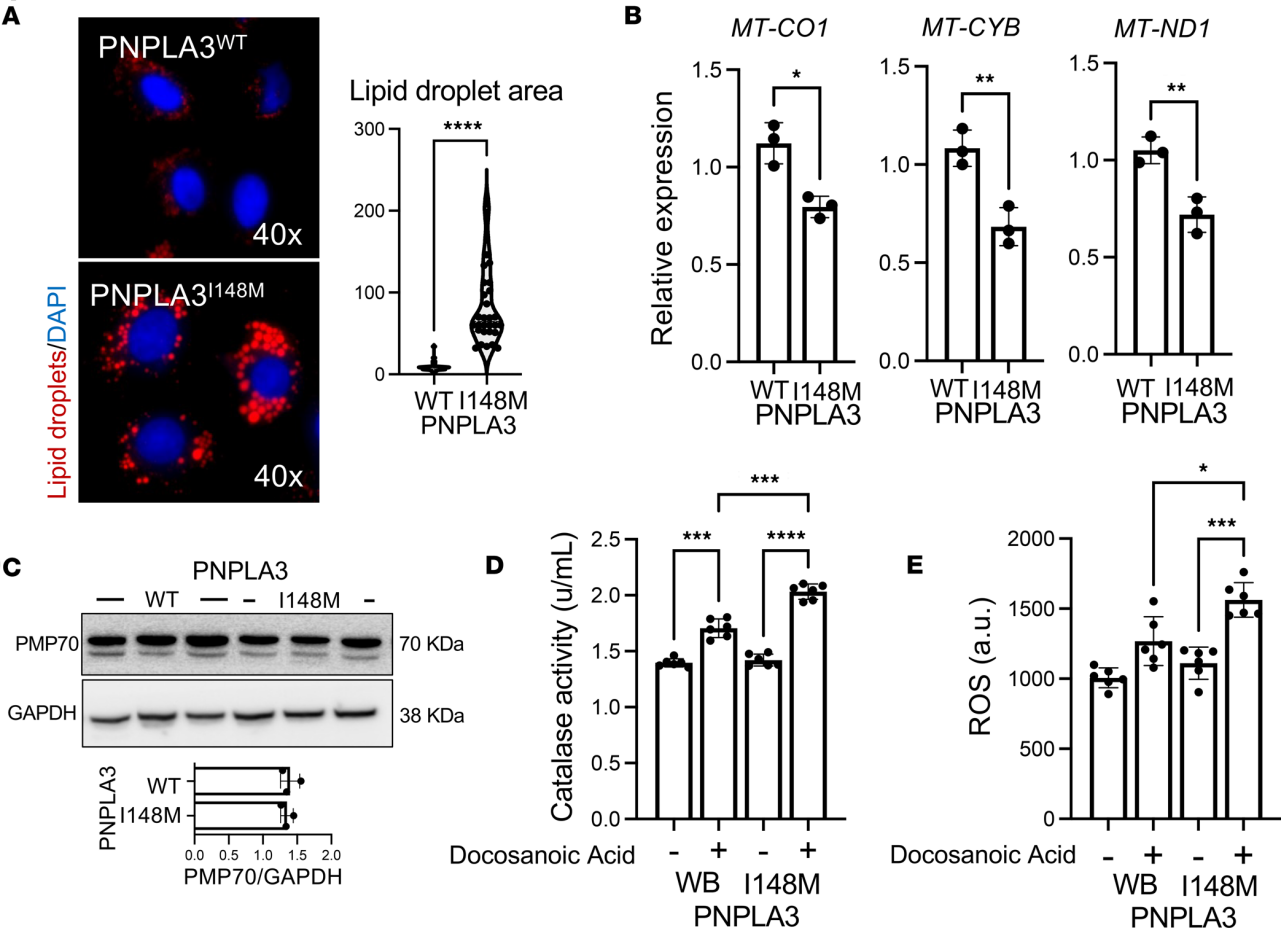
iHeps recapitulate the PNPLA3-I148M–induced alterations in lipid accumulation, mitochondrial stress, and peroxisomal activity. (**A**) LipidTOX staining micrographs and quantification of lipid droplet area of iHep-PNPLA3-WT and iHep-PNPLA3-I148M (****P < 0.0001, Welch’s *t* test, WT: *n* = 30, I148M: *n* = 30). (**B**) Mitochondrial content is decreased in iHep-PNPLA3-I148M in comparison with iHep-PNPLA3-WT. *MT-CO1* (*P < 0.05, Welch’s *t* test, WT: *n* = 3, I148M: *n* = 3), *MT-CYB* (**P < 0.01, Welch’s *t* test, WT: *n* = 3, I148M: *n* = 3), *MT-ND1* (**P < 0.01, Welch’s *t* test, WT: *n* = 3, I148M: *n* = 3). (**C**) PMP70 Western blot showing that the amount of peroxisomes is not different between iHep-PNPLA3-WT and iHep-PNPLA3-I148M (*P* = 0.6647, Welch’s *t* test, WT: *n* = 3, I148M: *n* = 3). (**D**) Peroxisomal activity is higher in iHeps with the PNPLA3 variant. After exposing the cells to docosanoic acid, higher catalase activity (***P < 0.001, ****P < 0.0001, 1-way ANOVA, WT: *n* = 6, I148M: *n* = 6) and (**E**) intracellular ROS (*P < 0.05, ***P < 0.001, 1-way ANOVA, WT: *n* = 6, I148M: *n* = 6) were observed in the mutant iHeps.

**Figure 15:**
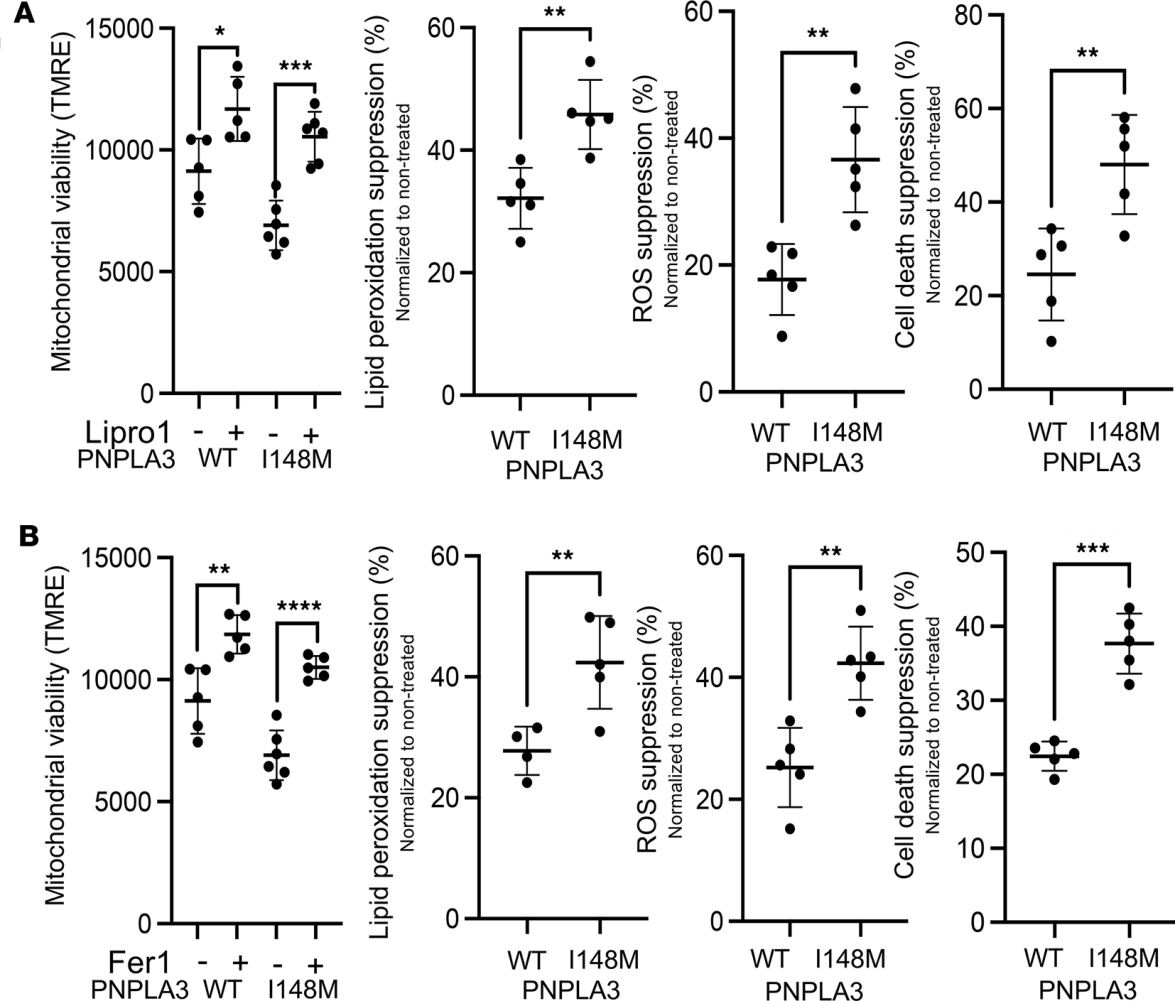
iHeps reproduce increased ferroptosis susceptibility associated with the PNPLA3-I148M mutation. Mitochondria viability, lipid peroxidation, ROS measurement, and cell death suppression in iHep-PNPLA3-WT and iHep-PNPLA3-I148M in the presence of Lipro1 or Fer1. (**A**) Lipro1: TMRE (*P = 0.0163, ***P = 0.0001, Welch’s *t* test, WT: *n* = 6, I148M: *n* = 6), lipid peroxidation (**P = 0.0037, Welch’s *t* test, WT: *n* = 5, I148M: *n* = 5), ROS (**P = 0.0039, Welch’s *t* test, WT: *n* = 5, I148M: *n* = 5), and cell death (**P = 0.0067, Welch’s *t* test, WT: *n* = 6, I148M: *n* = 6). (**B**) Fer1: TMRE (**P = 0.0069, ****P < 0.0001, Welch’s *t* test, WT: *n* = 6, I148M: *n* = 5), lipid peroxidation (**P = 0.0097, Welch’s *t* test, WT: *n* = 4, I148M: *n* = 5), ROS (**P = 0.0026, Welch’s *t* test, WT: *n* = 5, I148M: *n* = 5), and cell death (***P = 0.0003, Welch’s *t* test, WT: *n* = 5, I148M: *n* = 5).

To determine if iHep-PNPLA3-I148M cells recapitulate the phenotype observed in Hep-PNPLA3-I148M, intracellular lipid accumulation was assessed by immunofluorescence. iHep-PNPLA3-I148M displayed a significantly greater lipid droplet area than iHep-PNPLA3-WT cells, replicating our previous findings (Figure 14A). The iHep-PNPLA3-I148M cells also exhibited reduced mitochondrial content, as determined by mitochondrial DNA quantification (Figure 14B). No difference in peroxisome number was detected between iHep-PNPLA3-WT and iHep-PNPLA3-I148M cells, based on PMP70 expression (Figure 14C). However, peroxisomal activity was elevated in the mutant cells, as evidenced by higher catalase activity and increased intracellular ROS levels following exposure to docosanoic acid (Figure 14, D and E). Through exposure to the ferroptosis inducers Erastin and RSL3, we concluded that iHep-PNPLA3-I148M are more susceptible to ferroptosis than iHep-PNPLA3-WT (Supplemental Figure 10). To counter this effect, we treated cells with the ferroptosis inhibitors Lipro1 and Fer1. As previously observed, administration of either inhibitor led to an increase in mitochondrial viability and suppression of lipid peroxidation, ROS production, and cell death in both WT and mutant cells (Figure 15, A and B). These changes, however, occurred to a much greater magnitude in iHep-PNPLA3-I148M than iHep-PNPLA3-WT cells. The PNPLA3-I148M variant has been reported to contribute to liver inflammation and fibrosis (35, 36). To investigate the potential role of ferroptosis in these processes, we differentiated iPSC-PNPLA3-WT and iPSC-PNPLA3-I148M lines into hepatic stellate cells (iHSCs) (37) and established a coculture system with iHeps. The cocultures were then exposed to Fer1, a ferroptosis inhibitor. Under basal conditions, cocultures carrying the PNPLA3-I148M variant displayed higher levels of IL-6 and TGFB1, along with increased α-SMA expression, indicative of a pro-inflammatory and pro-fibrotic milieu. Notably, inhibition of ferroptosis led to a reduction in these markers (Supplemental Figure 11). Overall, these data replicate results observed in primary human hepatocytes, positioning iHeps as a valuable tool for the study of genetic variants and disease mechanisms.

## Discussion

In this study, the PNPLA3-I148M variant was characterized in primary human hepatocytes, liver tissue, and iHeps derived from patients with no history of hepatic disorders. We found that the presence of the *PNPLA3* rs738409:G variant increases lipid peroxidation, induces mitochondrial shrinkage and substantial metabolic and transcriptomic alterations, and upregulates peroxisomal β-oxidation and ferroptosis-associated cell death. Using ferroptosis inhibitors, nutritional supplementation, and genetic approaches, we were able to counter many of these negative effects. Our studies highlight the potential mechanisms driving cell death in PNPLA3-I148M mutant hepatocytes and the importance of utilizing iPSC-based models to study clinically relevant genetic variants and precision therapeutics.

GWAS have identified genomic variants associated with the development of ESLD; however, the precise mechanisms by which these variants lead to ESLD and cellular death in humans have not been determined. Animal studies have revealed that endogenous PNPLA3 expression is exceedingly low in livers and highly expressed in adipose tissue (38). As a result, PNPLA3 must be overexpressed in mouse liver tissue to produce a human-like phenotype (39). Moreover, the mouse and human PNPLA3 proteins are only approximately 60% identical, and when the human PNPLA3 protein is overexpressed in mouse livers, it is resistant to dietary regulation, indicating that the protein may have different functions in different species (40). Thus, it remains unclear to what extent rodent models can fully recapitulate the phenotype of the PNPLA3-I148M variant in humans.

To understand the human biology of the PNPLA3-I148M variant, we obtained primary human hepatocytes from individuals possessing the mutation without the diagnosis of a related hepatic disorder. We then employed integrated omics approaches to perform flux propensity analysis based on the COMPASS algorithm to uncover precise alterations in human PNPLA3-I148M hepatocyte function. Using this approach, we identified that increased β-oxidation in peroxisomes contributed to increased lipid peroxidation levels and 20%–25% of the ferroptosis process. Importantly, recent studies (41) demonstrated that impaired hepatic mitochondrial function is a feature of MASLD progression when the PNPLA3-I148M variant is present. In line with these reports, we identified abnormalities in mitochondrial morphology, reduced ATP levels, and increased glycolysis contributing to energy production among Hep-PNPLA3-I148M. Due to the structural and functional proximity of ER and mitochondria, mitochondria may take up excess ER stress–induced calcium ions through mitochondria-associated membrane contact sites (42–44). This excess calcium may contribute to the opening of the mitochondrial permeability transition pore, disrupting mitochondrial morphology and function (45–47). MCFAs and LCFAs are generally processed by the mitochondria (23). However, impaired mitochondrial functionality routes MCFAs and LCFAs to the peroxisome, enhancing peroxisomal β-oxidation. Our findings also demonstrated a role for the PNPLA3-I148M variant in regulating levels of *GPX4* expression and modulating iron metabolism and lipid peroxidation under conditions of cellular stress. These forms of cellular stress may have tissue-cumulative effects on metabolism and increase the risk of recurrent liver disease in transplanted patients.

Our studies also identified the importance of the programmed cell death mechanism, ferroptosis, in patients with the PNPLA3 variant. This type of cell death is caused by iron-dependent lipid peroxidation and is associated with mitochondrial shrinkage and reduced expression of *GPX4*. Hep-PNPLA3-I148M expressed the ferroptosis signature of lower *GPX4* expression, mitochondrial shrinkage, higher content of lipid droplets, and lipid peroxidation. Furthermore, we found altered expression of genes and proteins related to ferroptosis, including *FADS2*, *SCD*, and TFRC. Consistent with the recent finding of iron accumulation in individuals with metabolic dysfunction (48), our results demonstrate that expression of the PNPLA3-I148M variant leads to ferroptosis in healthy human hepatocytes. Further research is needed to determine the extent to which ferroptosis and expression of the PNPLA3-I148M variant contribute to the development of MASH in humans, as well their role on the inflammation and fibrosis process. By increasing available GSH to bind free Fe^2+^ (33) or inducing GPX4 expression, we were able to reduce lipid peroxidation, thus limiting ferroptosis and rescuing human hepatocytes from cell death, suggesting a path for future potential precision therapies.

In addition to the primary human hepatocytes used throughout this study, we generated and characterized an iHep cell model derived from patient iPSCs to understand the impact of the PNPLA3-I148M mutation. Our previously published differentiation protocol allows us to produce mature iHeps with hepatocyte-specific gene signatures comparable to those of primary hepatocytes. This cellular model holds many advantages over primary cells. As these cells are newly differentiated, they are not subjected to changes associated with the patient’s lifestyle, such as drug or alcohol use, environmental exposures, or disease history, which may alter the cellular transcriptome or function. Additionally, the ease with which these cells can be gene-edited using CRISPR/Cas9 allows for the creation of isogenic variant and control cell lines. Finally, as our iHeps undergo a maturation process during differentiation, they provide an important platform for the study of development. Previous studies utilizing iPSCs to model the PNPLA3-I148M variant found that presence of the mutation increased fat accumulation, altered the stress response to lipid exposure, and reduced metabolism (49). Our iHeps displayed similar phenotypic differences while recapitulating the ferroptosis response observed in primary human hepatocytes. Overall, iPSC-derived cell lines have proven to be an accurate and valuable disease model for the study of genetic factors influencing hepatic disorders.

In conclusion, we found that the use of molecular genotyping and phenotyping in combination with sensitive metabolomic and transcriptomic analysis was effective at identifying molecular and metabolic processes associated with ESLD in humans. Further investigation into other MASLD-related polymorphic sites’ effects on metabolism and cellular adaptation to stressors in physiological and disease conditions will also be of high importance. The results presented here have the potential to transform our understanding of the effect of the PNPLA3-I148M variant and elucidate important implications for the treatment of liver diseases. Further studies should be conducted on targeting ferroptosis as a therapeutic strategy for the treatment of hepatic disorders.

## Methods

### Sex as a biological variable.

Due to the limited availability of liver samples from healthy individuals carrying the PNPLA3-I148M variant, reported to have a prevalence of approximately 6% in the healthy US population, we made every effort to collect and include all available samples in our analysis. The liver tissue samples we were able to obtain were all male.

### Cell culture and treatments.

Primary human hepatocytes (cell sources included in Supplemental Methods) were cultured in Hepatocyte Culture Medium (Lonza) on type I rat tail collagen-coated plates (Corning) and kept at 37°C in 5% CO_2_ (Supplemental Table 1). Cell preparations with post-thaw viability greater than 80% were used for cell culture. A hepatocellular cell line was obtained from ATCC (HepG2, ATCC), and it and its isogenic control (HepG2 CC^Cas9^) were maintained in EMEM medium (ATCC) supplemented with 10% HyClone fetal bovine serum (Thermo Fisher Scientific) and 1% Penicillin/Streptomycin (Thermo Fisher Scientific) and kept at 37°C in 5% CO_2_. Transfection of HyPer-Red plasmid was performed by Lipofectamine 3000 (Invitrogen). To evaluate peroxisomal β-oxidation, hepatocytes were exposed to 0.1 mM phytanic acid (Cayman Chemical) or 0.1 mM docosanoic acid (Cayman Chemical) conjugated with 5% BSA-free fatty acid before being added to the medium. Thioridazine (Cayman Chemical) dissolved in DMSO was added to the medium at a final concentration of 1 μM 48 hours before collecting the cells. 10 μM of 3-amino-4-(cyclohexylamino)-benzoic acid, ethyl ester (Ferrostatin-1, Cayman Chemical) and 5 μM N-[(3-chlorophenyl)methyl]-spiro[piperidine-4,2′(1′H)-quinoxalin]-3′-amine (Liproxstatin-1, Cayman Chemical) were used as ferroptosis inhibitors. 20 μg/mL of (2R)-2-[6-(4-chlorophenoxy)hexyl]-2-oxiranecarboxylic acid monosodium salt was used to inhibit mitochondrial β-oxidation (Etomoxir, Cayman Chemical). Briefly, primary human hepatocytes were plated in a collagen-coated plate and kept overnight at 37°C in 5% CO_2_. The following day, the ferroptosis inhibitors were added, and 48 hours later, the analysis was performed. To induce stress in the cells, a mix of linoleic acid (Sigma-Aldrich) and α-linoleic acid (Cayman Chemical) at 400 μM (1:1) was used. To evaluate the effect of GSH, primary human hepatocytes on collagen-coated plates were treated with 10 μM reduced l-glutathione (GSH, Sigma-Aldrich) for 48 hours.

### Statistics.

Descriptive data were expressed as mean ± SD. Variables were tested for normal distribution prior to statistical testing. Comparisons of 2 groups were performed using a nonparametric Mann-Whitney *U* test or a parametric 2-tailed *t* test (indicated in the corresponding figure legends). For groups of 3 or more, the test for significance was performed using either a parametric 1-way ANOVA if all samples were normally distributed or a nonparametric Kruskal-Wallis test for non-normal distributions. Both tests were accompanied with a posttest analysis for multiple-comparison correction (indicated in the corresponding figure legends). All *P* values less than 0.05 were considered statistically significant. Statistical analyses were performed using SPSS 25.0 (IBM), DESeq2 in R (CRAN 4.04), MetaboAnalyst (ver. 6.0), and Prism 10.4.0 (GraphPad Software).

### Study approval.

Collection of deidentified liver resections and use as healthy control isolated hepatocytes were performed through the Human Liver Tissue & Hepatocytes Research Resource (HLTHRR), an NIH-funded (R24 DK139775) cell resource with an approved IRB# STUDY20040276 by the Human Research Review Committee of the University of Pittsburgh.

### Data availability.

Further information and requests for resources and reagents should be directed to the lead contact. RNA-Seq data have been deposited at EMBL-EBI database under accession code *E-MTAB-15689* (https://www.ebi.ac.uk/biostudies/arrayexpress/studies/E-MTAB-15689?key=6910b918-0ab6-4048-b42f-9aa0db9f7d62). Values for all data points in graphs are reported in the Supporting Data Values file. Please refer to Supplemental Methods for additional information.

## Author contributions

RMF, NH, TM, RDA, LAPF, YS, ZL, ZH, BY, SA, ZNKK, ENT, JF, OA, LNA, AA, MN, KO, AS, FW, NM, KM, AS, MP, TT, KY, AM, DCG, MM, and KT performed data acquisition. RMF, NH, OA, AA, KM, KT, IJF, DLT, JB, TY, DN, and ASG analyzed and interpreted data. NH and ZW performed statistical analysis. AO and LAPF isolated human hepatocytes. RMF, NH, OA, LNA, AA, and DN analyzed functional and metabolic data. KM, TT, KY, AM, KT, and TY performed acquisition of clinical data and specimens for genotyping. RMF, OA, DN, AMB, IJF, and ASG wrote the manuscript. RMF, NH, AA, OA, KM, KT, DLT, JB, DN, IJF, and ASG participated in critical revision of the manuscript for intellectual content. DN and ASG conceived and designed the study and obtained funding. All authors contributed to the preparation of the manuscript.

## Funding support

This work, in whole or in part, is the result of NIH funding and is subject to the NIH Public Access Policy. Through acceptance of this funding, the NIH has been given a right to make the work publicly available in PubMed Central.

NIH grant DK099257 to ASG and DN.

NIH TR003289 to ASG, DLT, JB.

NIH DK096990 to IJF and ASG.

NIH DK117881, DK119973, TR002383 to DLT and ASG.

NIH DK135606 to ASG and MTM.

NIH grant S10 OD028450 to DLT.

JSPS KAKENHI Grant Number 21H03004 to KT.

NIH grant R24 DK139775 to the HLTHRR.

Internal funds from the Center for Transcriptional Medicine at the University of Pittsburgh.

## Supplementary Material

Supplemental data

Unedited blot and gel images

Supporting data values

## Version 1 10/21/2025

In-Press Preview

## Version 2 12/08/2025

Electronic publication
