# Supplementary material for: PNPLA3-I148M genetic variant rewires lipid metabolism to drive programmed cell death in human hepatocytes
Source: JCI Insight. 2025 Oct 21;10(23):e193805. doi: 10.1172/jci.insight.193805 (PMC12890524; doi:10.1172/jci.insight.193805)
Supplement: Supplemental data [file jciinsight-10-193805-s057.pdf]

SUPPLEMENTARY METHODS

Human hepatocytes

Primary human hepatocytes were isolated using a three-step collagenase digestion technique as previously described (1). Briefly, cell isolation was initiated by perfusion with pre-warmed to 37°C calcium-free HBSS supplemented with 0.5 mM EGTA and collagenase solution (VitaCyte, Indianapolis, IN) until the tissue was fully digested. The digested liver was cooled with ice-cold Leibovitz's L-15 medium and strained through a series of progressively smaller stainless-steel sieves with a final filtration through a 100 µm mesh. The final crude cell suspension was centrifuged twice, and the post-digest medium was aspirated. Cell viability was assessed after isolation using trypan blue exclusion, and only cell preparations with viability greater than 80% were cryopreserved. Single vials of cryopreserved hepatocytes were obtained from In Vitro ADMET Laboratories (Malden, MA), Novabiosis (Durham, NC), and Anabios (San Diego, CA). Specific information on age, gender, and cell viability of human liver tissue and hepatocytes used in this study is described in Supplementary Table S1**.**

Genotyping

DNA was extracted with the DNeasy Blood & Tissue Kit (QIAGEN, Hilden, Germany), and samples were genotyped using TaqMan SNP genotyping assays for *PNPLA3* (rs738409) (Thermo Fisher Scientific, San Jose, CA)(2) and listed in the Supplementary Table S2. Amplification and genotype clustering were performed using a StepOnePlus system (Applied Biosystems, Foster City, CA).

Immunostaining

Human liver tissue was fixed in 4% paraformaldehyde for 12 hours and 70% ethanol overnight at 4°C and then embedded in paraffin. Cut sections (5-7 microns) were mounted on glass slides for immunofluorescence and immunohistochemistry. Each sample was first stained with hematoxylin and eosin for histological examination. Slides were deparaffinized with xylenes and dehydrated with ethanol. Antigen unmasking was performed by boiling in 10 mM citrate buffer, pH 6.0. For immunofluorescence, the slides were then blocked for 1 hour with 10% goat serum, left incubating overnight at 4°C with primary antibodies listed in the Supplementary Table S2, and then incubated with secondary antibody for 1 hour at room temperature. Sections were covered with DAPI-containing mounting media. For immunohistochemistry staining after the antigen unmasking, the slides were exposed to 3% hydrogen peroxide and incubated overnight at 4°C with primary antibodies listed in the Supplementary Table S2. Tissue sections were then incubated with the

secondary biotinylated antibody corresponding to the animal species of the primary antibody (BA-1000; Vector Laboratories, Burlingame, CA) and exposed to 3,3'-diaminobenzidine (SK-4105; Vector Laboratories) to visualize the peroxidase activity. Counterstaining was performed with Richard-Allan Scientific Signature Series Hematoxylin (Thermo Scientific, Waltham, MA). All procedures followed the kit instructions. Images were captured with a Nikon Eclipse Ti microscope. Following that, images were analyzed using ImageJ software. RGB stacks were generated, pre-processed to equalize the illumination within the stack, thresholded, and measured.

ATF6 and E-Cadherin immunohistochemistry images were acquired using the PE Opera Phenix using the Harmony 5.1 software. Images were acquired with a 40X/1.1 NA Water Immersion objective. Fluorescence images were acquired with the following channels: Hoechst Ex. 405/Em. 435-480, ATF6 640 Ex./Em. 650-760, and E-Cadherin Ex. 488/Em. 500-550. To gather maximum intensity projections, a z-stack was acquired in confocal mode with 14 planes and a distance between each plane of 1.2 um. To determine the number of ATF6-positive nuclei, we used Harmony 5.1 to create an analysis protocol that identifies nuclei based on Hoechst signal. Then, to determine ATF6 signal, we sampled a ring region of 1.5X the outer border of the nuclear region. ATF6-positive cells were then quantified by thresholding signal above the ATF6 negative control well average signal. The overall percent of ATF6-positive cells was determined by taking the total count of ATF6-positive cells and expressing it as a percent of the total number of nuclei (Hoechst-positive cells) in 49 fields per condition.

Intracellular Lipid Staining

Samples were fixed with 4% PFA for 15 minutes and washed three times with phosphate-buffered saline (PBS). After that, the samples were incubated with a 0.3 mM Nile Red (Sigma Aldrich, ON, Canada) or 1x HCS LipidTOX™ Red neutral lipid stain (Thermo Scientific, Waltham, MA) solution for 30 minutes at room temperature. Then, they were washed twice with PBS and counterstained with 1 µg/mL of DAPI (Sigma Aldrich, ON, Canada) for 1 minute. Samples were imaged using an Eclipse Ti inverted microscope (Nikon) and the NIS-Elements software platform (Nikon, NY, USA). Following that, images were analyzed using ImageJ software.

XBP1 splicing quantification

Total cellular RNA was isolated using the RNeasy Mini kit (QIAGEN, Hilden, Germany) and reverse transcribed using SuperScript III (Invitrogen, Carlsbad, CA) following the manufacturers'

instructions. Complementary DNA was amplified using two sets of primers: total XBP1 (tXBP1) and a sequence that distinguished between the unspliced (uXBP1) and spliced XBP1 (sXBP1) (3).The primer sequences are listed in Supplementary Table S2. The amplification products were resolved in a 2% agarose gel, and the quantification was performed using ImageJ software.

Transmission electron microscopy

Human hepatocytes in suspension were briefly centrifuged and washed with PBS solution. Samples were then fixed with 2.5% glutaraldehyde overnight at 4°C. Fixed samples were processed by the Center for Biologic Imaging at the University of Pittsburgh and treated with 1% osmium tetroxide and 1% potassium ferricyanide for 1 hour at room temperature. Samples were washed with PBS and dehydrated in a graded series of ethanol solutions (30%, 50%, 70%, and 90% - 10 minutes each) and three 15-minute changes in fresh 100% ethanol. Infiltration was done with four 1-hour changes of EPON embedding plastic. The last change of EPON was allowed to polymerize overnight at 37°C and then for 48 hours at 60°C. Resin blocks were removed from the Eppendorf tubes, and 70 nm sections were taken and placed onto copper TEM grids. Image acquisition was made using either the JEM-1011 or the JEM-1400Plus transmission electron microscopes (Jeol, Peabody, MA) at 80 kV fitted with a side mount AMT 2k digital camera (Advanced Microscopy Techniques, Danvers, MA). The mitochondrial shape was evaluated as published previously using ImageJ (4). Briefly, individual mitochondria were analyzed for circularity (4π area/perimeter^2) and its reciprocal value used as the form factor. The form factor has a minimum value of 1 where mitochondria are a perfect circle, increasing in mitochondria with a rod shape.

Mitochondrial DNA (mtDNA)

Genomic DNA was extracted with the DNeasy Blood & Tissue Kit (QIAGEN, Hilden, Germany) following the manufacturers' instructions. mtDNA content was analyzed by SYBER green qPCR using primers amplifying mitochondrial cytochrome C oxidase subunit 1 (*MT-CO1*), mitochondrial cytochrome B (*MT-CYB*), and mitochondrial NADH-Ubiquinone Oxidoreductase Chain 1 (*MT-ND1*). Relative gene expression was normalized to β-actin (*ACTB*) mRNA and was calculated using the ΔΔCT method. Primers are listed in the Supplementary Table S2.

Mitochondria stress

MitoSOX Red mitochondrial superoxide indicator (Thermo Fisher Scientific) was used to detect mitochondrial superoxide in hepatocytes. A 5 mM stock solution was prepared in DMSO and

diluted in PBS to a working concentration of 5 μM immediately before use. Hepatocytes were washed twice with warm PBS before staining. MitoSOX Red working solution was added to the cells and incubated at 37°C for 30 minutes, protected from light. After incubation, cells were washed twice with PBS to remove unbound dye. Tetramethylrhodamine ethyl ester (TMRE, Thermo Fisher Scientific) was used to assess mitochondrial membrane potential in the iHeps. A 2 mM stock solution was prepared in DMSO and diluted in cell culture medium to a working concentration of 100 nM immediately before use. TMRE working solution was added to the cells, and they were incubated at 37°C for 60 minutes, protected from light. After incubation, cells were washed twice with warm PBS to remove unbound dye. Fluorescence was analyzed using a fluorescence microscope, and images were analyzed using ImageJ software.

ATP measurement

The intracellular ATP content was measured in hepatocytes by using the ATP Determination Kit (Thermo Fisher Scientific, Waltham, MA) following the manufacturer's instructions. The cells were incubated at 37°C for 1 hour in the absence or presence of 10 μg/ml oligomycin A (Cayman Chemical, Ann Arbor, MI) or 10 mM 2-deoxyglucose (Cayman Chemical, Ann Arbor, MI). The ATP content was measured by luminescence with an integration time of 1 second per well.

Mitochondria Respiration

Oxygen consumption rate (OCR) was measured with an XF96 Extracellular Flux Analyzer (Seahorse Bioscience). Hepatocytes were seeded in Seahorse 96-well collagen-coated microplates, and the respiration was assayed the following day. The culture media was replaced with 100 μL of assay medium (DMEM 10 mM glucose, 1 mM pyruvate, 2 mM glutamine, and 1% penicillin-streptomycin without serum and bicarbonate) and incubated at 37°C without CO_2 for 1 hour. The cells were then exposed to 1.5 μM of oligomycin A, 1 μM of carbonyl cyanide-p-trifluoromethoxyphenylhydrazone (FCCP), and 0.5 μM of rotenone/antimycin A (ROT/AA). Basal, ATP-linked, and maximal OCR were measured and compared between Hep-PNPLA3-WT and Hep-PNPLA3-I148M.

Lipidomics

Hepatocytes originally stored at -80°C were retrieved and transferred into a 15 mL centrifuge glass tube. 1 mL of water was added to lyse the cells, and the mixture was allowed to rest on ice for 10 minutes. Next, 2 mL of methanol and 0.9 mL of methylene chloride were added to the mixture. The mixture was vortexed to ensure a monophasic solution; otherwise, we stepwise

added 50 µL of methanol and vortexed until a monophasic solution was achieved. Next, we added 5 µL of the SPLASH™ LIPIDOMIX® internal standard mixture to each sample. Next, we added 1 mL of water and 0.9 mL of methylene chloride and vortexed. Next, we centrifuged at 1200 rpm for 10 minutes at 4°C. The lower lipid layer was collected and transferred to a fresh glass tube. Next, we added 2 mL of methylene chloride to the remains in the extraction tube and mixed, centrifuged, and collected the lower lipid layer and added it to the first extract. The extract was evaporated under nitrogen and resuspended in 500 µL of mobile phase A. Mobile phase A (MPA) was 2 mM ammonium acetate in 7/93 methylene chloride/acetonitrile. Mobile phase B (MPB) was 2 mM ammonium acetate in 50/50 water/acetonitrile. Samples were run on our SCIEX Triple Quad™ LC-MS/MS system coupled with ExionLC™. 5 µL of each sample was injected per run and eluted based on the following gradient: 0min: 0% MPB, 2min: 0% MPB, and at flowrate of 0.2 ml/min.; 2.01min: 0% MPB, at flowrate of 0.7 ml/min; 11min: 50% MPB; 11.5min: 70% MPB; 12.5min: 100% MPB; 15min: 100% MPB, 15.1min: 0% MPB at flowrate of 0.2 ml/min; 17min: 0% MPB. Lipids were detected based on an optimized scheduled multiple reaction monitoring (MRM) MS method that ran concurrently in both positive and negative modes based on our in-house MRM list of over 1100 lipids. The MS method was set up using the following parameters: GS1 = 45 psi, GS2 = 70 psi, spray voltage = 1600 V, temperature = 450°C, settling time = 15 ms, and pause time = 3 ms. Analytes were separated using Phenomenex's Luna® 3 µm NH2, 2 x 100 mm column (00D-4377-B0) fitted with NH2 Standard Security Guard column (AJ0-4301) kept at 35°C. Peaks were extracted using SCIEX OS (version 3.1.6.44), and our in-house algorithm, implemented in R (CRAN 4.1.1), was utilized to filter out analytes with poor quality. Only unique metabolites with good quality across all samples were considered in the downstream analysis. Peaks were further normalized to the internal standards, and, finally, metabolite abundance was normalized to the cell number. Further downstream analyses were performed in Metaboanalyst® (V. 6.0), GraphPad Prism (Version 10.4.0), Python (ver. 3.11.4), and BioRender.com. Lipid classes legend; TAG: Triacylglycerols, PS: Phosphatidylserine, PI: Phosphatidylinositol, PG: Phosphatidylglycerol, PE: Phosphatidylethanolamine, PC: Phosphatidylcholine, MAG: Monoacylglycerol, LPS: Lipopolysaccharide, LPI: Lysophosphatidylinositol, LPG: Lysophosphatidylglycerol, LPC: Lysophosphatidylcholine, DAG: Diacylglycerol, CL: Cardiolipin, CE: Cholesteryl Ester, PG: Phosphatidylglycerol, PS: Phosphatidylserine. PUFAs legend: Arachidonic acid (20:4), Docosahexaenoic acid (DHA, 22:6), Eicosapentaenoic acid (EPA, 20:5), Linoleic acid (18:2), Alpha-Linolenic Acid (18:3).

Western Blot

Cells were trypsinized, pelleted, and washed with PBS. Lysis was performed with RIPA buffer (Sigma-Aldrich, Saint Louis, Missouri) and 1x Halt™ Protease and Phosphatase Inhibitor Cocktail (Thermo Fisher Scientific, Waltham, MA) for 30 minutes at 4°C, followed by centrifuging at 13,000 x g for 10 min at 4°C. Protein concentrations of the supernatant were determined by comparison with a known concentration of bovine serum albumin using a Pierce BCA Protein Assay Kit (ThermoFisher Scientific, Waltham, MA). 30 μg of lysate were loaded per well into 10% Mini-PROTEAN TGX™ gel (BioRad, Hercules, CA). Proteins were transferred onto a PVDF Transfer Membrane (Thermo Fisher Scientific, Waltham, MA). Membranes were incubated with a primary antibody solution overnight and then washed before incubation in a secondary antibody solution for 1 hour. Target antigens were finally detected using SuperSignal™ West Pico PLUS Chemiluminescent Substrate (Thermo Fisher Scientific, Waltham, MA). Images were scanned and analyzed using ImageJ software. The antibodies used are listed in the Supplementary Table S2.

Stable Isotopic Tracing Experiment

Primary human hepatocytes were cultured in Hepatocyte Culture Medium (Lonza, Walkersville, MD) on type I rat tail collagen-coated plates (Corning, Corning, NY) and kept at 37°C in 5% CO_2 overnight for attachment. The following day, cells were cultured with docosanoic-1,2,3,4-^{13}C4 acid (#57527, Sigma-Aldrich, Saint Louis, MO) and ^{13}C1-labelled palmitic acid (#28749, Cayman Chemical, Ann Arbor, MI) conjugated in 5% BSA free-fatty acid. After 48 hours, the cell pellets were stored at -80°C until we were ready for Acetyl-CoA measurement based on a protocol adopted from well-established methods from the literature (5-7). Briefly, on the day of analysis, the cell pellets were transferred to the pre-chilled 2 mL Precellys homogenizer tubes preloaded with 300-350 mg of the small ceramic beads (1.4 mm OD). Next, 200 µL of pre-chilled extraction solvent (10% (wt/vol) trichloroacetic acid (TCA) in optimal grade water) was added to each sample. Subsequently, samples were homogenized to disintegrate the cellular organelles using the Precellys + Cyrolys Evolution Homogenizer with the following settings: speed = 6000 rpm; cycle = 4 x 20 seconds; pause = 120 seconds; Cyolys = On; temperature = 4°C; and Mode Auto. After complete homogenization, 300 µL of the extraction solvent was added to achieve a final volume of 500 µL, and the sample tubes were incubated on ice for 10 minutes, followed by centrifugation for 10 minutes at 1700 x g at 4^{0}C. The supernatant was collected for solid phase extraction (SPE). SPE was performed using the Oasis PRiME HLB 96-well µElution Plate with 3 mg Sorbent per well. Briefly, the vacuum pressure was set at 5 in Hg and the columns were conditioned with 200 µL of methanol, followed by equilibration with 200 µL of water. Next, 500 µL

of the metabolite extract was loaded, and the column was desalted with 200 µL of water. Finally, the samples were eluted three times with 50 µL of 25 mM ammonium acetate, resuspended in 5% salicylic acid, and analyzed using Sciex 7500 LC-MS/MS system. Samples were run on the Kinetex 2.6um F5 100 A, LC Column 150 x 2.1 mm C18 column (Phenomenex). The flow rate was set to 0.1 mL/min with 5 mM ammonium acetate and 2.5 mM DMBA (N,N-Dimethylbutylamine) as mobile phase A and 100% methanol as mobile phase B. The gradient for the 22 min method was set as follows- 0 min: 2% B; 5 min: 25% B; 5.5 min: 100% B; 16 min: 2% B. The MRMs for isotopologue measurements are in Table S4. The data was corrected for natural abundance using IsoCorrectorR (v3.18) (8).

Analysis of reactive oxygen species

Total ROS in live cells was measured using the Cellular ROS Assay Kit (Abcam, Cambridge, UK). Following the manufacturer's instructions, the fluorescence (Ex/Em = 520/605 nm) was quantitatively measured on a Synergy HTX microplate reader (Biotek, Winooski, VT). ROS unit is arbitrary unity, and the values were normalized to the mean of the PNPLA3 WT non-treated group. To detect hydrogen peroxide (H_2O_2), a HyPerRed vector (Addgene 48249, Watertown, MA) was transfected, and live cells were imaged with a Nikon Inverted Research Fluorescence Microscope ECLIPSE Ti(9). Images were scanned and analyzed using ImageJ software.

Analysis of Lipid peroxidation

Malondialdehyde (MDA) is a natural byproduct of lipid peroxidation, and its quantification is generally used as a marker for lipid peroxidation. The MDA content in primary human hepatocytes was quantified using *Lipid Peroxidation (MDA) Assay Kit*, a commercially available kit from Sigma-Aldrich (Sigma-Aldrich, Saint Louis, MO). Cryopreserved primary human hepatocytes were pelleted and analyzed directly or cultured for 3 hours in suspension before the analysis. Cell lysates were deproteinized, and thiobarbituric acid (TBA) was added to generate an MDA-TBA adduct. The MDA-TBA adduct was quantified by fluorometric detection (Ex/Em = 430 nm/590 nm). The oxidation-sensitive probe C11-BODIPY 581/591 (Invitrogen, Carlsbad, CA) was used to evaluate lipid peroxidation under a fluorescence microscope. Untreated hepatocytes were prepared with a cytocentrifuge, incubated for 30 minutes with 10 µM C11-BODIPY, and washed with PBS. Cultured cells were incubated with 10 µM C11-BODIPY dissolved in culture media for 30 minutes at 37°C and washed with PBS.

Pex2 knockdown

The following siRNAs were ordered from GE Dharmacon: Accell Human Pex2 siRNA SMARTpool (E-006548-00-0050) and Accell Non-targeting Pool (D-001910-10-50). siRNAs were reconstituted in siRNA Buffer (B-002000-UB-100, Dharmacon, CO) at 100 μM. Hepatocytes were plated into collagen-coated plates. The following day, cells were washed once with PBS and siRNAs in media were added at a final concentration of 1 μM. After 24 hours, fresh media was added. Hepatocytes were cultured for an additional 24 hours after which samples were collected for analysis.

PNPLA3 gene edition

The single-guide RNA (sgRNA) sequence was designed to cut the human *PNPLA3* gene at the position chr22:43,928,854 to replace the minor allele (G) with the major allele (C). The sgRNA sequence can be found in Supplementary Figure 3 and Figure 12A. The sgRNA was cloned into a plasmid vector and nucleofected into a hepatocellular cell line or human iPSC line together with the donor DNA (*ACTTCTCTCTCCTTTGCTTTCACAGGCCTTGGTATGTTCCTGCTTCATCCCCTTTTACAGTG GCCTTATCCCTCCTTCCTTCAGAGGCGTGGTAAGT*). Antibiotic selection was performed, and DNA from selected clonal cells was extracted, amplified, and purified before sequencing as previously described (Synthego, Redwood City, CA). Major homozygous clones were identified, expanded, and cryopreserved, and one was used to perform the experiments.

Sanger sequencing

DNA extraction was performed with the KAPA Express Extract DNA Extraction Kit (Kapa Biosystems, London, UK). Polymerase chain reaction (PCR) amplification was conducted with the KOD ONE PCR Master Mix (Toyobo, Osaka, Japan) using the forward primer: 5′-CCA ACA ACC CTT GGT CCT GT-3′ and reverse primer: 5′-GGG TAG CCT GGA AAT AGG GC-3′ for *PNPLA3*. PCR products were then purified using the ExoSAP-IT Express PCR cleanup kit (Applied Biosystems, Foster City, CA) and sequenced at the Genomics Research Core at the University of Pittsburgh (Pittsburgh, PA). Sequencing buffer and a 1:4 dilution of BigDye 3.1 (ThermoFisher Scientific, Waltham, MA) were added, and thermocycling was performed according to ABI recommendations. According to manufacturer instructions, removing unincorporated sequencing reagents was performed using CleanSeq magnetic beads (Agencourt, Beckman Coulter, Brea, CA). Two control samples were included with every sequencing to ensure the proper performance of reagents and equipment.

RNA sequencing

RNA extraction, library preparation, and sequencing were performed at the University of Pittsburgh HSCRF Genomics Research Core. Briefly, total RNA was extracted from isolated primary human hepatocytes using the RNeasy Plus Micro Kit (Qiagen). RNA integrity was assessed using the High Sensitivity RNA ScreenTape system on an Agilent 2200 TapeStation (Agilent). The SMART-Seq HT Kit (Takara Bio) was used to generate cDNA from 10 ng of total RNA, and the cDNA product was checked by an Agilent Fragment Analyzer system (Agilent) for quality control. The sequencing library was constructed by following the Illumina Nextera XT Sample Preparation Guide. One nanogram of input cDNA was tagmented and amplified using the Illumina Nextera XT kit. Equimolar amounts of each sample were finally pooled and sequenced on an Illumina Nextseq 500 system using a paired-end 75-bp strategy.

RNA-seq data analysis

All memory-intensive computations were performed on clusters from the University of Michigan Great Lakes Advanced Research Computing resources. The quality of the raw fastq files was checked using FASTQC (version 0.11.9). In summary, the FastQC reports show that the guanine-cytosine content of the samples ranged from 47% to 54%. As a reference, a guanine-cytosine content between 30-40% is considered too low, as the DNA will be unstable, while a guanine-cytosine content between 70-80% is considered too high because it makes PCR amplification more difficult. So, a guanine-cytosine content of about 50 to 60% is desirable (10). Another important metric is the mean quality scores. All the samples have a score that falls within the acceptable threshold of 30. The QC-passed raw reads were then aligned to the genome with STAR (version 2.7.5a) (11). The quality of the aligned reads was assessed using QoRTs (version 1.3.6) (12). The quality of the aligned reads was assessed based on the percentage of novel splice events. For all of the samples, the percentage of novel splice events is about 1%, within acceptable limits. All samples passed the strandedness test. For all the samples, about 97% of the reads are mapped to the first strand. This attests to the quality of the sequenced RNA fragments. Downstream analyses were performed using DESeq2 in R (CRAN 4.04), BEAVR (a Browser-based tool for the Exploration And Visualization of RNAseq data) (13), GSEA (14, 15), and GraphPad Prism version 8. The p-value was computed based on Wald test statistics. Genes with a fold change greater than 1.5 and p-value less than 0.05 are considered significant.

Flux propensity analysis

The flux propensity of hepatocytes was computed by integrating RNA-Seq data with the reconstructed genome-scale model of human metabolism (also known as Recon 2.2) (16) based

on the COMPASS algorithm detailed in Wagner, Wang (17). First, the vector of maximum flux, x_r^{opt}, through each and every metabolic reaction in Recon 2.2 was computed. This was achieved by solving the following optimization problem:

$$\text{Maximize: } \|x\|, \; x \in \mathbb{R}^m$$

$$\text{Subjected to: } \quad A.x = 0$$
$$\omega \le x \le \lambda$$
$$x_{rev} = 0$$

Where x is a vector of metabolic flux through each metabolic pathway in Recon 2.2, m is the number of metabolic reactions in Recon 2.2, A is the stoichiometry matrix of all metabolites with respect to their metabolic reactions, and ω and λ are the lower and upper bounds of the flux, x. The bounds are set to a default value of ±1000. x_{rev} denotes the reverse fluxes of reversible reactions, which was set to zero to ensure only forward reactions were used in computing the maximum fluxes.

Next, gene expression data from samples was utilized to design a penalty variable for each metabolic reaction such that the numerical value of the penalty variable is inversely proportional to the level of expression of genes that participate in each reaction. The implication is that genes with low expression will impose stricter penalties on their corresponding reaction. Subsequently, a linear optimization problem was designed to determine the set of penalty variables that minimize the penalty imposed on the metabolic pathways. This was achieved by solving the following optimization problem:

$$\text{Minimize: } \|P.x\|, \; x \in \mathbb{R}^m$$

$$\text{Subjected to: } \quad A.x = 0$$
$$\omega \le x \le \lambda$$
$$x_{rev} = 0$$
$$x_r \ge 0.95 x_r^{opt}$$

The resulting penalty values were aggregated to generate a reaction propensity score that is inversely proportional to the aggregated penalty value for each reaction in Recon 2.2.

Downstream analyses were performed in Python on the Google Colab platform, R (CRAN 4.04), and GraphPad Prism version 8. To compare the relative activity of each metabolic reaction between *PNPLA3* rs738409:G and wild-type hepatocytes, Cohen's D statistics were used to compute the fold change difference between the means of two groups:

$$\text{Cohen's D} = \frac{M_1 - M_2}{SD_{pooled}}$$

where,

$$SD_{pooled} = \sqrt{(\Sigma(s_1^2 + s_2^2)/2}$$

Where M_1 and M_2 are the means of propensity score for group 1 (*PNPLA3* rs738409:G hepatocytes) and group 2 (wild-type hepatocytes), respectively. s_1 and s_2 are the standard deviations of group 1 and group 2, respectively.

A positive Cohen's D value indicates a reaction that is relatively more active in *PNPLA3* rs738409:G hepatocytes, while a negative value indicates relatively higher activity in wild-type hepatocytes. The higher the magnitude of Cohen's D, the higher the relative difference in activity between the two groups. We also computed the statistical significance of these Cohen's D using Wilcoxon's p-value adjusted based on the Benjamini-Hochberg (BH) method. Because of the small sample size (n=3), an absolute Cohen's D value greater than 1.5 and a Wilcoxon's p-value less than 0.1 are considered significant. Table S3 contains detailed information of the results from our flux propensity analysis. Below, we defined some of the metabolic reactions mentioned in the manuscript.

**metadata_r_id**	**reaction_name**	**subsystem**
PYRt2p	pyruvate peroxisomal transport via proton symport	D-alanine metabolism
r0173	(S)-Lactate:NAD+ oxidoreductase Glycolysis / Gluconeogenesis / Pyruvate metabolism EC:1.1.1.27	Glycolysis/gluconeogenesis
LDH_L	L-lactate dehydrogenase	Glycolysis/gluconeogenesis
CYSGLTH	Glutathione:cystine oxidoreductase	Methionine and cysteine metabolism
GAPD	glyceraldehyde-3-phosphate dehydrogenase	Glycolysis/gluconeogenesis

Untargeted metabolomics

Global metabolomics was carried out based on our previously reported protocol (18, 19). Briefly, hepatocytes stored at -80˚C were retrieved and transferred into a 15 mL centrifuge tube. After adding 4 mL of 80% (v/v) MEOH/water to each sample, they were briefly vortexed and deprotenated by incubating at -80˚C for 20 minutes. Samples were then vortexed at 4^0C for 5 minutes, followed by centrifugation at 4˚C, 4696 x g (maximum speed) for 10 minutes. Supernatants were transferred to a fresh tube. 500 µL of 80% (v/v) MEOH/water was added to the remaining pellets, vortexed for 5 minutes at 4˚C, and then centrifuged at 4˚C, 4696 x g

(maximum speed) for 10 minutes. Supernatant was added to the previously collected supernatant. Next, the supernatant was completely dried in a vacuum concentrator. Dried pellets were stored at -80°C until they were processed the day of analysis. On the day of the analysis, the dried extracts were reconstituted in 200 µL of methanol/water (50/50 v/v), sonicated for 10 minutes, vortexed for a few seconds, and then filtered. 100 µL of reconstituted samples were transferred to the LC vials, and 10 µL of each sample was pooled to the QC samples. The samples were analyzed on both HILIC and C18 columns with both positive and negative ion modes.

For the C18 column in positive mode, 10 µL (8 µL for negative mode) of the samples were injected for analysis on an Agilent 6520 QTOF LC/MS machine using an ACQUITY UPLC BEH C18 Column (130 Å, 1.7 µm, 2.1 mm X 150 mm) coupled with 5 mm Van-Guard Pre-Columns. The column compartment was set at 40°C, and the analysis was performed in both positive and negative modes. Mobile phase A was water with 0.1% formic acid, while mobile phase B contained acetonitrile with 0.1% formic acid. The gradient method is as follows: 0 min: 1% B; 1 min: 1% B; 8 min: 99% B; 13 min: 85% B; 13.1 min: 1% B; 16 min: 1% B.

For the HILIC column in positive mode, 5 µL (2 µL for negative mode) of the samples were injected for analysis on an Agilent 6520 QTOF LC/MS machine using an XBridgeBEH Amide XP Column (130 Å, 2.5 µm, 4.6 mm X 150 mm) coupled with a 5 mm VanGuard Cartridge. The column compartment was set at 40°C, and the analysis was performed in both positive and negative modes. Mobile phase A was 10 mM ammonium formate in water with 0.1% formic acid, while mobile phase B contained 10 mM ammonium formate in acetonitrile with 0.1% formic acid. The gradient method is as follows: 0 min: 1% B; 1 min: 1% B; 11.8 min: 80% B; 12.5 min: 1% B; 14.7 min: 1% B; 16 min: 1% B.

The metabolite peaks were extracted using Agilent Masshunter Profinder based on our in-house library. Any metabolites whose RSD is greater than 30% in the QC measurements were removed from further analysis. The peak areas were normalized with cell numbers. Other downstream analysis was performed with Metaboanalyst® (ver. 6.0) and GraphPad Prism version 8. Figures were created with Biorender.com. Metabolites with more than a 1.5-fold change and with a p-value less than 0.05 were considered to be significantly different between the two groups.

Quantitative Real-Time PCR

Total cellular RNA was isolated using the RNeasy Mini kit (QIAGEN, Hilden, Germany) and reverse transcribed using SuperScript III (Invitrogen, Carlsbad, CA) following the manufacturers' instructions. We performed qPCR with a StepOnePlus system (Applied Biosystems, Foster City, CA) using TaqMan Fast Advanced Master Mix (Life Technologies, Waltham, MA). Relative gene

expression was normalized to β-actin (*ACTB*) mRNA. TaqMan probes are listed in Supplementary Table S2. Relative expression was calculated using the ΔΔCT method.

Cell Viability Measurements

Cell viability was assessed in a 96-well format by Alamar Blue (Invitrogen, Carlsbad, CA) absorbance (570 nm/600 nm) measured on a Synergy HTX microplate reader (Biotek, Winooski, VT). Post-isolation and post-thaw viability of primary human hepatocytes was evaluated by trypan blue dye exclusion counting using a Countess 3 automated cell counter (Invitrogen, Carlsbad, CA).

Production of High Titer Lentivirus

Vector cloning, vector sequencing, and production of high-titer lentivirus were performed by Vectorbuilder (Chicago, IL). Briefly, lentiviruses were produced by co-transfecting 293T cells with the expression vectors pLV[Exp]-CMV>EGFP or pLV[Exp]-CMV>hGPX4 (transcript variant NM_001039848.4 containing a selenocysteine insertion sequence (SECIS) element to ensure proper incorporation of selenocysteine), envelope plasmid (pMD2. G), and packaging plasmids (pMDLg/pRRE and pRSV-Rev) using FuGENE 6 transfection reagent (Promega Corporation, Madison, WI, USA). After cell debris and macromolecular contaminants were filtered by a 0.45 um filter, the filtrate was collected and concentrated with PEG6000 (Sigma-Aldrich, Saint-Louis, MO) to increase the virus titer to yield > 1x10^9 viral particles/mL before being stored at −80°C.

Generation and Culture of Human iPSC

Human fibroblasts were derived from fetal hepatocytes as previously described. Briefly, human fetal hepatocytes were cultured in DMEM medium (GIBCO, Life Technologies, Carlsbad, CA, USA) containing 1X Penicillin/Streptomycin (ThermoFisher Scientific, Waltham, MA), 10^{-7} M of insulin (Sigma-Aldrich, Saint-Louis, MO), and 5% bovine serum albumin (GIBCO, Life Technologies, Carlsbad, CA, USA) for 2-3 weeks.

hiPSC-PNPLA3^{GG} were generated from fibroblasts. Reprogramming of fibroblasts was performed using episomal plasmid vectors adapted from a previously described method (20). Briefly, for each nucleofection, 1 million cells were resuspended in 100 mL of the AmaxaTM NHDF Nucleofector kit (Lonza, Walkersville, MD) containing 1 mg of each of the four episomal plasmid vectors encoding OCT3/4 and p53 shRNA, SOX2 and KLF4, L-MYC and LIN28, and enhanced green fluorescent protein (eGFP) (Addgene, Boston, MA, USA). Cells were nucleofected using

the Amaxa 4D-Nucleofector (Lonza, Walkersville, MD) and plated in mTeSR on human embryonic stem cell-qualified Matrigel (Corning, New York, NW)-coated plates. Colonies were isolated around 60 days after induction based on morphology. The cell line underwent karyotyping, and its pluripotency was validated by the expression of NANOG, OCT4, and membrane markers SSEA and TRA-1-60 at different passages. Additionally, the cell line was routinely tested and found to be negative for mycoplasma contamination. After 10 passages, a stable hiPSC-PNPLA3^{GG} colony was selected to be gene edited as previously mentioned.

Differentiation of human iPSC into induced hepatocytes (iHeps)

Our hepatocyte differentiation protocol was based on previously published methods(21-25). Briefly, hiPSC-PNPLA3^{CC} and hiPSC-PNPLA3^{GG} were passaged with Accutase (Stem Cell Technologies, Vancouver, Canada) and re-plated at a density of 1 to 2x10^5 per cm^2 in growth factor reduced Matrigel (Corning Incorporated, Corning, NY)-coated plates in mTeSR. The day after the hiPSC passage, cells were exposed to a defined differentiation medium containing RPMI (Invitrogen, Carlsbad, CA), 1x B-27 without insulin supplement (Invitrogen, Carlsbad, CA), 0.5% penicillin/streptomycin (Millipore, Billerica, MA), 0.5% of Non-Essential Amino Acids (Millipore, Billerica, MA), 100 ng/mL Activin A (R&D Systems, Minneapolis, MN), 10 ng/mL BMP4 (R&D Systems, Minneapolis, MN), and 20 ng/mL FGF2 (BD, Franklin Lakes, NJ) for two days and placed in a normal O_2 incubator (endoderm induction). Cells were subsequently maintained in a similar medium without FGF2 and BMP4 for two days in ambient O_2 (definitive endoderm). Cells were then grown for 10 days in a defined medium containing 45% DMEM low glucose (ThermoFisher Scientific, Waltham, MA), 45% F-12 (ThermoFisher Scientific, Waltham, MA), 10% CTS KnockOut SR XenoFree Medium (ThermoFisher Scientific, Waltham, MA), 0.5% Non-Essential Amino Acids (ThermoFisher Scientific, Waltham, MA), 0.5% L-glutamine (ThermoFisher Scientific, Waltham, MA), 50 ng/mL HGF (R&D Systems, Minneapolis, MN), and 1% DMSO (Sigma-Aldrich, Saint Louis, MO). Medium was changed every other day (hepatic specification). At the end of Stage 3, cells were detached and re-plated at 30%–40% confluence in thin type 1 collagen layer (Corning Incorporated, Corning, NY) for further maturation. Cells were grown for 4 days in a defined medium containing 45% DMEM low glucose (ThermoFisher Scientific, Waltham, MA), 45% F-12 (ThermoFisher Scientific, Waltham, MA), 10% CTS KnockOut SR XenoFree Medium, 0.5% Non-Essential Amino Acids (ThermoFisher Scientific, Waltham, MA), 0.5% L-glutamine (ThermoFisher Scientific, Waltham, MA), 0.1% of Gentamicin/Amphotericin-B (ThermoFisher Scientific, Waltham, MA), 1% of penicillin/streptomycin (ThermoFisher Scientific, Waltham, MA), 50 ng/mL HGF (R&D Systems, Minneapolis, MN), 1% DMSO, 0.5uM

Dexamethasone (Sigma-Aldrich, Saint Louis, MO), 0.1% of Ascorbic Acid (Sigma-Aldrich, Saint Louis, MO), 0.1% of Bovine Serum Albumin Free of Fatty Acids, 0.1% of Hydrocortisone, 0.1% of Transferrin, 0.1% of Insulin (HCM Bullet Kit, ThermoFisher Scientific, Waltham, MA), 100 μM of Ursodeoxycolic acid (Sigma-Aldrich, Saint Louis, MO), 20 μM of palmitic acid (Sigma-Aldrich, Saint Louis, MO), 30 μM of oleic acid (Sigma-Aldrich, Saint Louis, MO), 400 μM of linoleic acid (Sigma-Aldrich, Saint Louis, Missouri), 400 μM of a-linoleic acid (Cayman Chemical, Ann Arbor, MI), 20 μM of rifampicin (Sigma-Aldrich, Saint Louis, Missouri), and 1x of cholesterol (ThermoFisher Scientific, Waltham, MA) (Stage 4, hepatic maturation).

Catalase Activity

Catalase activity in live cells was measured using the Catalase Colorimetric Activity Kit (Invitrogen, Carlsbad, CA) after exposing the cells to docosanoic for 48 hours. Following the manufacturer's instructions, the absorbance (560 nm) was quantitatively measured on a Synergy HTX microplate reader (Biotek, Winooski, VT).

Differentiation of human iPSCs into induced hepatic stellate cells (iHSC)

The iHSC differentiation protocol was based on a previously published method(26). Briefly, hiPSC-PNPLA3^{CC} and hiPSC-PNPLA3^{GG} were passaged with Accutase (Stem Cell Technologies, Vancouver, Canada) and re-plated at a density of 1 to 2x10^{5} per cm^{2} in growth factor reduced Matrigel (Corning Incorporated, Corning, NY)-coated plates in mTeSR. One day after passage, the cells were exposed to a defined differentiation medium containing 10 μg/mL BMP4 to induce mesoderm formation. After 4 days, the medium was replaced with fresh medium containing 10 μg/mL BMP4 along with 100 μg/mL FGF1 and FGF3 for 48 hours. This was followed by a medium change to a defined medium supplemented with 100 μg/mL FGF1 and FGF3, 30 mM retinol, and 200 μM palmitic acid for another 48 hours. In the final step of the differentiation protocol, the cells were cultured in a defined medium containing 30 mM retinol and 200 μM palmitic acid.

REFERENCES

1. Faccioli LAP, Kocas-Kilicarslan ZN, Diaz-Aragon R, Motomura T, Amirneni S, Malizio MR, Coard MC, et al. Human Hepatocytes Isolated from Explanted Livers: A Powerful Tool to Understand End-stage Liver Disease and Drug Screening. Organogenesis 2021;17:117-125.
2. Yang J, Trepo E, Nahon P, Cao Q, Moreno C, Letouze E, Imbeaud S, et al. A 17-Beta-Hydroxysteroid Dehydrogenase 13 Variant Protects From Hepatocellular Carcinoma Development in Alcoholic Liver Disease. Hepatology 2019;70:231-240.

3. Yoon SB, Park YH, Choi SA, Yang HJ, Jeong PS, Cha JJ, Lee S, et al. Real-time PCR quantification of spliced X-box binding protein 1 (XBP1) using a universal primer method. PLoS One 2019;14:e0219978.
4. De Vos KJ, Allan VJ, Grierson AJ, Sheetz MP. Mitochondrial function and actin regulate dynamin-related protein 1-dependent mitochondrial fission. Curr Biol 2005;15:678-683.
5. Basu SS, Blair IA. SILEC: a protocol for generating and using isotopically labeled coenzyme A mass spectrometry standards. Nature protocols 2012;7:1-11.
6. Izzo LT, Trefely S, Demetriadou C, Drummond JM, Mizukami T, Kuprasertkul N, Farria AT, et al. Acetylcarnitine shuttling links mitochondrial metabolism to histone acetylation and lipogenesis. Science Advances 2023;9:eadf0115.
7. Jones AE, Arias NJ, Acevedo A, Reddy ST, Divakaruni AS, Meriwether D. A single LC-MS/MS analysis to quantify CoA biosynthetic intermediates and short-chain acyl CoAs. Metabolites 2021;11:468.
8. Heinrich P, Kohler C, Ellmann L, Kuerner P, Spang R, Oefner PJ, Dettmer K. Correcting for natural isotope abundance and tracer impurity in MS-, MS/MS- and high-resolution-multiple-tracer-data from stable isotope labeling experiments with IsoCorrectoR. Sci Rep 2018;8:17910.
9. Ermakova YG, Bilan DS, Matlashov ME, Mishina NM, Markvicheva KN, Subach OM, Subach FV, et al. Red fluorescent genetically encoded indicator for intracellular hydrogen peroxide. Nat Commun 2014;5:5222.
10. Amr SS, Funke B. Targeted Hybrid Capture for Inherited Disease Panels. Clinical Genomics 2015:251-269.
11. Dobin A, Davis CA, Schlesinger F, Drenkow J, Zaleski C, Jha S, Batut P, et al. STAR: ultrafast universal RNA-seq aligner. Bioinformatics 2013;29:15-21.
12. Hartley SW, Mullikin JC. QoRTs: a comprehensive toolset for quality control and data processing of RNA-Seq experiments. BMC bioinformatics 2015;16:1-7.
13. Perampalam P, Dick FA. BEAVR: a browser-based tool for the exploration and visualization of RNA-seq data. BMC bioinformatics 2020;21:1-14.
14. Mootha VK, Lindgren CM, Eriksson K-F, Subramanian A, Sihag S, Lehar J, Puigserver P, et al. PGC-1α-responsive genes involved in oxidative phosphorylation are coordinately downregulated in human diabetes. Nature genetics 2003;34:267-273.
15. Subramanian A, Tamayo P, Mootha VK, Mukherjee S, Ebert BL, Gillette MA, Paulovich A, et al. Gene set enrichment analysis: a knowledge-based approach for interpreting genome-wide expression profiles. Proceedings of the National Academy of Sciences 2005;102:15545-15550.
16. Swainston N, Smallbone K, Hefzi H, Dobson PD, Brewer J, Hanscho M, Zielinski DC, et al. Recon 2.2: from reconstruction to model of human metabolism. Metabolomics 2016;12:1-7.
17. Wagner A, Wang C, Fessler J, DeTomaso D, Avila-Pacheco J, Kaminski J, Zaghouani S, et al. Metabolic modeling of single Th17 cells reveals regulators of autoimmunity. Cell 2021;184:4168-4185. e4121.
18. Achreja A, Yu T, Mittal A, Choppara S, Animasahun O, Nenwani M, Wuchu F, et al. Metabolic collateral lethal target identification reveals MTHFD2 paralogue dependency in ovarian cancer. Nature Metabolism 2022;4:1119-1137.
19. Zhu Z, Achreja A, Meurs N, Animasahun O, Owen S, Mittal A, Parikh P, et al. Tumour-reprogrammed stromal BCAT1 fuels branched-chain ketoacid dependency in stromal-rich PDAC tumours. Nature Metabolism 2020;2:775-792.
20. Okita K, Matsumura Y, Sato Y, Okada A, Morizane A, Okamoto S, Hong H, et al. A more efficient method to generate integration-free human iPS cells. Nature methods 2011;8:409-412.

21. Takeishi K, de l'Hortet AC, Wang Y, Handa K, Guzman-Lepe J, Matsubara K, Morita K, et al. Assembly and function of a bioengineered human liver for transplantation generated solely from induced pluripotent stem cells. Cell reports 2020;31.
22. Günther C, Winner B, Neurath MF, Stappenbeck TS. Organoids in gastrointestinal diseases: from experimental models to clinical translation. Gut 2022;71:1892-1908.
23. Florentino RM, Morita K, Haep N, Motomura T, Diaz-Aragon R, Faccioli LA, de l'Hortet AC, et al. Biofabrication of synthetic human liver tissue with advanced programmable functions. Iscience 2022;25.
24. Florentino RM, Li Q, Coard MC, Haep N, Motomura T, Diaz-Aragon R, Faccioli LA, et al. Transmembrane channel activity in human hepatocytes and cholangiocytes derived from induced pluripotent stem cells. Hepatology Communications 2022;6:1561-1573.
25. Faccioli LA, Sun Y, Animasahun O, Motomura T, Liu Z, Kurihara T, Hu Z, et al. Human induced pluripotent stem cell based hepatic-modeling of lipid metabolism associated TM6SF2 E167K variant. Hepatology 2023:10.1097.
26. Coll M, Perea L, Boon R, Leite SB, Vallverdu J, Mannaerts I, Smout A, et al. Generation of Hepatic Stellate Cells from Human Pluripotent Stem Cells Enables In Vitro Modeling of Liver Fibrosis. Cell Stem Cell 2018;23:101-113 e107.

Supplementary Table 1a. Normal human hepatocyte and tissue used in this study.

Hepatocyte ID	AGE	SEX	VIABILITY	PNPLA3 rs738409
HH035	77	female	85%	GG
HH104*	11	male	96%	CG
HH98*	15	male	95%	CG
001	46	male	80%	GG
DEC	53	male	86%	GG
JDL	54	male	80%	GG
EXW	50	male	97%	GG
BRX	45	male	92%	CC
JYN	49	female	86%	CC
HU8339	31	female	91%	CG
HH1072	40	female	89%	GG
HH112	74	male	86%	CG
HH48	33	female	84%	CG
HH46	74	female	90%	CC
HH68	32	male	84%	CC
HH1110	47	male	83%	GG
HH1050	9	male	80%	CC
HH1083	45	female	86%	CG
HH1043	37	male	87%	GG
HH1098	43	female	80%	GG
HH1121	23	female	90%	CG
962	68	female	85%	GG
1081	36	male	80%	GG
HH012	83	male	N/A (tissue)	GG
HH080	16	male	N/A (tissue)	CC
HH133	12	male	N/A (tissue)	GG
HH160	24	male	N/A (tissue)	GG
HH162	60	male	N/A (tissue)	CC

** Cases which we have used isolated primary human hepatocyte and liver tissue.*

Supplementary Table 1b.

Parameter (mean ± SD)	PNPLA3 WT (CC/CG)	PNPLA3 I148M (GG)	p-value
**BMI**	28.7 ± 7.74	31.79 ± 7.51	0.4516
**Age (years)**	38.5 ± 21.9	45.2 ± 20.3	0.4204
**Albumin secretion *in vitro (ng/mL)***	300.4 ± 54.5	285.3 ± 45.4	0.6155
**Urea secretion *in vitro (mg/dL)***	18.7 ± 9.3	19.5 ± 4.5	0.8438

Supplementary table 2a. Taqman® SNP Genotyping Assay IDs used in this study

Target gene	SNP ID	Assay ID	Company
PNPLA3	rs738409	C______7241_10	Life Technologies

Supplementary table 2b. Taqman® Primers used in this study

Target gene	Assay ID	Company
ACTB	Hs01060665_g1	Life Technologies
FADS2	Hs00927433_m1	Life Technologies
GPX4	Hs00989766_g1	Life Technologies
SCD	Hs01682761_m1	Life Technologies
HNF4a	Hs00604431_m1	Life Technologies
FOXA1	Hs04187555_m1	Life Technologies
FOXA2	Hs00232764_m1	Life Technologies
PPARa	Hs00947536_m1	Life Technologies
CEBPa	Hs00269972_s1	Life Technologies
PNPLA3	Hs00228747_m1	Life Technologies
ATF4	Hs00909569_g1	Life Technologies
ERN1	Hs00980095_m1	Life Technologies

Supplementary table 2c. Antibodies and dilutions used for Western blots and Immunohistochemistry.

Target protein	Catalog number	Clone	Company	Dilution	Host
ACOX1	ab184032	EPR19038	Abcam	1:1000	Rabbit
ACSL4	ab124805	EPR2761(2)	Abcam	1:5000	Rabbit
GAPDH	600041IG150UL	1E6D9	Proteintech	1:10000	Mouse
HSPA5	ab21685		Abcam	1:1000	Rabbit
ACAA1	NBP1-55156	N/A	Novus Biologicals	1:200	Rabbit
CHOP	NB600-1335	9C8	Novus Biologicals	1:100	Mouse
ATF6	ab37149	N/A	Abcam	1:100	Rabbit
E-cadherin	NBP2-19051	7H12	Novus Biologicals	1:500	Mouse
FSP1	68049-1-Ig	1A2B2	Proteintech	1:200	Rabbit
TRF1	13-6800	H68.4	Thermo Fisher	1:250	Mouse
Adipophilin 2	610102	AP125	Progen	1:200	Mouse
GPX4	ab125066	EPNCIR144	Abcam	1:1000	Rabbit
PMP70	ab85550	N/A	Abcam	1:1000	Rabbit
Vinculin	700062	42H89L44	Thermo Fisher	1:1000	Rabbit
TFRC	MABC1765	3F3-FMA	Millipore	1:50	Mouse
Nanog	4893	1E6C4	Cell Signalling	1:2000	Mouse
OTC4	sc-9081	N/A	Santa Cruz	1:250	Rabbit
TRA-1-60	560173	N/A	BD PharMingen	1:10	Mouse
SSE4A	560218	N/A	BD PharMingen	1:10	Mouse
SOX17	NL1924R	N/A	R&D Systems	1:50	Goat
HNF4a	ab41898	K9218	Abcam	1:500	Mouse

ALB	A80-229A	N/A	Bethyl	1:100	Goat
AFP	180003	N/A	Invitrogen	1:300	Mouse

Supplementary table 2d. Recombinant DNA

Construction ID	Company	Catalog number
HyPerRed	Addgene	Cat#48249
pLV[Exp]-CMV>EGFP	Vectorbuilder	This paper
pLV[Exp]-CMV>hGPX4	Vectorbuilder	This paper

Supplementary table 2e. SYBER green primers used in this study

Target	Sequence	Company
u/s XBP1 foward	GGTCTGCTGAGTCCGCAGCA	Life Technologies
u/s XBP1 reverse	AAGGGAGGCTGGTAAGGAAC	Life Technologies
t XBP1 foward	TGAAAAACAGAGTAGCAGCTCAGA	Life Technologies
t XBP1 reverse	CCCAAGCGCTGTCTTAACTC	Life Technologies
MT-CO1 foward	CCTACTCCTGCTCGCATCTG	Life Technologies
MT-CO1 reverse	AGAATGGGGTCTCCTCCTCC	Life Technologies
MT-CYB forward	AACTTCGGCTCACTCCTTGG	Life Technologies
MT-CYB reverse	GGAGGTGATTCCTAGGGGGT	Life Technologies
MT-ND1 forward	CACCCAAGAACAGGGTTTGT	Life Technologies
MT-ND1 reverse	TGGCCATGGGATAGTTGTTAA	Life Technologies
ACTB forward	GGCATCCTCACCCTGAAGTA	Life Technologies
ACTB reverse	GAAGGTGTGGTGCCAGATTT	Life Technologies

RX_nmae	wilcox_stat	wilcox_pva	g10(wilcox_p	cohens_d	Abs(cohens	High/low	adjusted_p	metadata_	reaction_name	formula	associated_	subsystem	Subsystem_	EC_number	confidence
G5SADrm_pos	7	0.382733	0.4171041	1.164738	1.164738	1	0.518179	G5SADrm	L-glutamate 5-semialdehyde dehydratase, reversible, mitochondrial	1.00 * L-Glutamate 5-semialdehyde [m] --> 1.00 * proton	1.00 * L-Glu	Arginine and Proline Metabolism	1		4
P5CR_pos	2	0.382733	0.4171041	-0.73416	0.734162	2	0.518179	P5CR	pyrroline-5-carboxylate reductase	2.00 * proton [c] + 1.00 * Nicotinamide adenine dinucleo	PYCR1; PYC	Arginine and Proline Metabolism	1	1.5.1.2	4
r0615_pos	6	0.662521	0.1788004	0.382054	0.382054	1	0.758498	r0615	trans-4-Hydroxy-L-proline:NAD+ 5-oxidoreductase Arginine and pro	1.00 * Nicotinamide adenine dinucleotide [c] + 1.00 * tra	PYCR1; PYC	Arginine and Proline Metabolism	1	1.5.1.2	0
r0617_neg	6	0.662521	0.1788004	0.382054	0.382054	1	0.758498	r0617	trans-4-Hydroxy-L-proline:NADP+ 5-oxidoreductase Arginine and pr	1.00 * Nicotinamide adenine dinucleotide phosphate [c]	PYCR1; PYC	Arginine and Proline Metabolism	1	1.5.1.2	0
r0615_neg	4	1	0	0.001664	0.001664	1	1	r0615	trans-4-Hydroxy-L-proline:NAD+ 5-oxidoreductase Arginine and pro	1.00 * Nicotinamide adenine dinucleotide [c] + 1.00 * tra	PYCR1; PYC	Arginine and Proline Metabolism	1	1.5.1.2	0
r0616_neg	5	1	0	0.002186	0.002186	1	1	r0616	trans-4-Hydroxy-L-proline:NAD+ 5-oxidoreductase Arginine and pro	1.00 * Nicotinamide adenine dinucleotide [m] + 1.00 * tr	PYCR1; PYC	Arginine and Proline Metabolism	1	1.5.1.2	0
r0617_pos	4	1	0	0.001664	0.001664	1	1	r0617	trans-4-Hydroxy-L-proline:NADP+ 5-oxidoreductase Arginine and pr	1.00 * Nicotinamide adenine dinucleotide phosphate [c]	PYCR1; PYC	Arginine and Proline Metabolism	1	1.5.1.2	0
r0618_pos	5	1	0	0.002186	0.002186	1	1	r0618	trans-4-Hydroxy-L-proline:NADP+ 5-oxidoreductase Arginine and pr	1.00 * Nicotinamide adenine dinucleotide phosphate [m]	PYCR1; PYC	Arginine and Proline Metabolism	1	1.5.1.2	0
RE1807C_pos	0	0.080856	1.0922877	-3.14927	3.149265	2	0.240222	RE1807C	RE1807	1.00 * Water [c] + 1.00 * Nicotinamide adenine dinucleot	1.00 * Wat	Bile acid synthesis	2		0
RE2626C_pos	0	0.080856	1.0922877	-2.02292	2.022918	2	0.240222	RE2626C	RE2626	1.00 * Water [c] + 1.00 * Nicotinamide adenine dinucleot	1.00 * Wat	Bile acid synthesis	2		0
RE2626M_pos	0	0.080856	1.0922877	-1.86992	1.86992	2	0.240222	RE2626M	RE2626	1.00 * Water [m] + 1.00 * Nicotinamide adenine dinucleo	CYP27A1	Bile acid synthesis	2		0
RE3346C_pos	0	0.080856	1.0922877	-2.02292	2.022918	2	0.240222	RE3346C	RE3346	2.00 * proton [c] + 1.00 * Nicotinamide adenine dinucleo	ALDH3A1;	Bile acid synthesis	2	1.2.1.3	0
RE3346M_pos	0	0.080856	1.0922877	-1.86992	1.86992	2	0.240222	RE3346M	RE3346	2.00 * proton [m] + 1.00 * Nicotinamide adenine dinucle	ALDH1B1;	Bile acid synthesis	2	1.2.1.3	0
r0688_neg	0	0.080856	1.0922877	-3.14927	3.149265	2	0.240222	r0688	3alpha,7alpha-Dihydroxy-5beta-cholestan-26-al:NAD+ oxidoreduct	1.00 * Water [c] + 1.00 * Nicotinamide adenine dinucleot	ALDH1B1;	Bile acid synthesis	2	1.2.1.3	0
P45027A12m_pos	1	0.19043	0.7202646	-1.61331	1.613311	2	0.347218	P45027A12	5-beta-cholestane-3-alpha,7-alpha,12-alpha-triol 27-hydroxylase	1.00 * Nicotinamide adenine dinucleotide phosphate [m]	CYP27A1	Bile acid synthesis	2	1.14.13.15	4
RE2625M_neg	1	0.19043	0.7202646	-1.61331	1.613311	2	0.347218	RE2625M	RE2625	1.00 * Nicotinamide adenine dinucleotide [m] + 1.00 * 5b	CYP27A1	Bile acid synthesis	2		0
BAAT1x_pos	2	0.382733	0.4171041	-1.30963	1.309628	2	0.518179	BAAT1x	bile acid Coenzyme A: amino acid N-acyltransferase	1.00 * Glycine [x] + 1.00 * choloyl-CoA(4-) [x] --> 1.00 * Co	BAAT	Bile acid synthesis	2	2.3.1.65	4
HSD3B7P_pos	7	0.382733	0.4171041	1.185273	1.185273	1	0.518179	HSD3B7P	hydroxy-delta-5-steroid dehydrogenase, 3 beta- and steroid delta-iso	1.00 * Nicotinamide adenine dinucleotide phosphate [r]	HSD3B7	Bile acid synthesis	2		4
RE1796R_neg	7	0.382733	0.4171041	1.185273	1.185273	1	0.518179	RE1796R	RE1796	1.00 * Nicotinamide adenine dinucleotide [r] + 1.00 * 20-	HSD3B2	Bile acid synthesis	2	5.3.3.1	0
RE1845C_neg	7	0.382733	0.4171041	0.998664	0.998664	1	0.518179	RE1845C	RE1845	1.00 * Taurine [c] + 1.00 * chenodeoxycholoyl coenzyme	BAAT	Bile acid synthesis	2	2.3.1.65	0
RE1845C_pos	2	0.382733	0.4171041	-1.30965	1.309646	2	0.518179	RE1845C	RE1845	1.00 * Taurine [c] + 1.00 * chenodeoxycholoyl coenzyme	BAAT	Bile acid synthesis	2	2.3.1.65	0
r0629_neg	2	0.382733	0.4171041	-1.30963	1.309628	2	0.518179	r0629	Choloyl-CoA:glycine N-choloyltransferase Bile acid biosynthesis / Tau	1.00 * Glycine [c] + 1.00 * choloyl-CoA(4-) [c] --> 1.00 * Co	BAAT	Bile acid synthesis	2	2.3.1.65	0
r0797_neg	2	0.382733	0.4171041	-1.30963	1.309628	2	0.518179	r0797	glycine N-choloyltransferase EC:2.3.1.65	1.00 * Glycine [c] + 1.00 * chenodeoxycholoyl coenzyme	BAAT	Bile acid synthesis	2		0
BAAT2x_pos	6	0.662521	0.1788004	0.939309	0.939309	1	0.758498	BAAT2x	bile acid Coenzyme A: amino acid N-acyltransferase	1.00 * choloyl-CoA(4-) [x] + 1.00 * Taurine [x] --> 1.00 * Co	BAAT	Bile acid synthesis	2	2.3.1.65	4
RE1807C_neg	3	0.662521	0.1788004	-0.9529	0.952905	2	0.758498	RE1807C	RE1807	1.00 * Water [c] + 1.00 * Nicotinamide adenine dinucleot	1.00 * Wat	Bile acid synthesis	2		0
RE2626C_neg	3	0.662521	0.1788004	-0.82231	0.822307	2	0.758498	RE2626C	RE2626	1.00 * Water [c] + 1.00 * Nicotinamide adenine dinucleot	1.00 * Wat	Bile acid synthesis	2		0
RE3346C_neg	3	0.662521	0.1788004	-0.82231	0.822307	2	0.758498	RE3346C	RE3346	2.00 * proton [c] + 1.00 * Nicotinamide adenine dinucleo	ALDH3A1;	Bile acid synthesis	2	1.2.1.3	0
r0630_neg	6	0.662521	0.1788004	0.939309	0.939309	1	0.758498	r0630	Choloyl-CoA:glycine N-choloyltransferase Bile acid biosynthesis / Tau	1.00 * Taurine [c] + 1.00 * choloyl-CoA(4-) [c] --> 1.00 * Co	BAAT	Bile acid synthesis	2	2.3.1.65	0
r0688_pos	3	0.662521	0.1788004	-0.9529	0.952905	2	0.758498	r0688	3alpha,7alpha-Dihydroxy-5beta-cholestan-26-al:NAD+ oxidoreduct	1.00 * Water [c] + 1.00 * Nicotinamide adenine dinucleot	ALDH1B1;	Bile acid synthesis	2	1.2.1.3	0
AKR1C41_pos	4	1	0	-0.35507	0.355066	2	1	AKR1C41	aldo-keto reductase family 1, member C4 (chlordecone reductase; 3-	1.00 * proton [c] + 1.00 * Nicotinamide adenine dinucleo	AKR1C6	Bile acid synthesis	2	1.1.1.50	4
AKR1C42_pos	4	1	0	-0.35507	0.355066	2	1	AKR1C42	aldo-keto reductase family 1, member C4 (chlordecone reductase; 3-	1.00 * proton [c] + 1.00 * Nicotinamide adenine dinucleo	AKR1C6	Bile acid synthesis	2	1.1.1.50	4
r0743_pos	5	1	0	0.038147	0.038147	1	1	r0743	hydroxysteroid (17-beta) dehydrogenase 4 Bile acid biosynthesis EC:	1.00 * Nicotinamide adenine dinucleotide [x] + 1.00 * 3al	HSD17B4	Bile acid synthesis	2	1.1.1.35	0
r0744_pos	5	1	0	0.038147	0.038147	1	1	r0744	(24R,25R)-3alpha,7alpha,12alpha,24-tetrahydroxy-5beta-cholestan	1.00 * Water [x] + 1.00 * 3alpha,7alpha,12alpha-Trihydro	HSD17B4	Bile acid synthesis	2	4.2.1.107	0
r0747_pos	4	1	0	-0.35507	0.355066	2	1	r0747	3alpha,7alpha-Dihydroxy-5beta-cholestane:NADP+ oxidoreductase	1.00 * Nicotinamide adenine dinucleotide phosphate [c]	AKR1C6	Bile acid synthesis	2	1.1.1.50	0
r0750_pos	4	1	0	-0.35507	0.355066	2	1	r0750	3alpha,7alpha,12alpha-Trihydroxy-5beta-cholestane:NADP+ oxidor	1.00 * Nicotinamide adenine dinucleotide phosphate [c]	AKR1C6	Bile acid synthesis	2	1.1.1.50	0
DHCR241r_pos	1	0.19043	0.7202646	-1.58511	1.585111	2	0.347218	DHCR241r	24-dehydrocholesterol reductase [Precursor]	1.00 * zymosterol [r] + 1.00 * Flavin adenine dinucleotide	DHCR24	Cholesterol metabolism	3		4
DHCR242r_pos	1	0.19043	0.7202646	-1.59512	1.595115	2	0.347218	DHCR242r	24-dehydrocholesterol reductase [Precursor]	1.00 * Flavin adenine dinucleotide reduced [r] + 1.00 * 5a	CYP7A1; DH	Cholesterol metabolism	3		4
r0783_pos	1	0.19043	0.7202646	-1.59512	1.595115	2	0.347218	r0783	lanosterol D24-reductase Biosynthesis of steroids EC:1.3.1.72	1.00 * Nicotinamide adenine dinucleotide phosphate [r]	DHCR24	Cholesterol metabolism	3	1.3.1.72	0
r1380_neg	1	0.19043	0.7202646	-1.58511	1.585111	2	0.347218	r1380	delta24-sterol reductase Biosynthesis of steroids EC:1.3.1.72	1.00 * proton [r] + 1.00 * Nicotinamide adenine dinucleo	DHCR24	Cholesterol metabolism	3	1.3.1.72	0
SQLSr_pos	2	0.382733	0.4171041	-0.93048	0.930477	2	0.518179	SQLSr	Squalene synthase	1.00 * proton [r] + 1.00 * Nicotinamide adenine dinucleo	FDFT1	Cholesterol metabolism	3	2.5.1.21	4
r0170_neg	2	0.382733	0.4171041	-0.93048	0.930477	2	0.518179	r0170	Farnesyl-diphosphate:farnesyl-diphosphate farnesyltransferase Biosy	2.00 * Farnesyl diphosphate [r] --> 1.00 * Diphosphate [r]	FDFT1	Cholesterol metabolism	3	2.5.1.21	0
r0575_neg	2	0.382733	0.4171041	-0.93048	0.930477	2	0.518179	r0575	Presqualene diphosphate:farnesyl-diphosphate farnesyltransferase B	1.00 * proton [r] + 1.00 * Nicotinamide adenine dinucleo	FDFT1	Cholesterol metabolism	3	2.5.1.21	0
ICDHyrm_neg	9	0.080856	1.0922877	2.461852	2.461852	1	0.240222	ICDHyrm	Isocitrate dehydrogenase (NADP+)	1.00 * Nicotinamide adenine dinucleotide phosphate [m]	IDH2	Citric acid cycle	4	1.1.1.42	4
SUCOAS1m_neg	1	0.19043	0.7202646	-1.85174	1.851739	2	0.347218	SUCOAS1m	Succinate--CoA ligase (GDP-forming)	1.00 * Coenzyme A [m] + 1.00 * GTP [m] + 1.00 * Succinate	SUCLG1; SU	Citric acid cycle	4	6.2.1.4	0
SUCOAS1m_pos	1	0.19043	0.7202646	-1.85174	1.851739	2	0.347218	SUCOAS1m	Succinate--CoA ligase (GDP-forming)	1.00 * Coenzyme A [m] + 1.00 * GTP [m] + 1.00 * Succinate	SUCLG1; SU	Citric acid cycle	4	6.2.1.4	0
SUCOASm_neg	1	0.19043	0.7202646	-1.85174	1.851739	2	0.347218	SUCOASm	Succinate--CoA ligase (ADP-forming)	1.00 * Coenzyme A [m] + 1.00 * ATP [m] + 1.00 * Succinate	SUCLA2; SU	Citric acid cycle	4	6.2.1.5	0
SUCOASm_pos	1	0.19043	0.7202646	-1.85174	1.851739	2	0.347218	SUCOASm	Succinate--CoA ligase (ADP-forming)	1.00 * Coenzyme A [m] + 1.00 * ATP [m] + 1.00 * Succinate	SUCLA2; SU	Citric acid cycle	4	6.2.1.5	0
FUM_neg	3	0.662521	0.1788004	0.706368	0.706368	1	0.758498	FUM	fumarase	1.00 * Water [c] + 1.00 * Fumarate [c] --> 1.00 * (S)-malate	FH1	Citric acid cycle	4	4.2.1.2	0
FUM_pos	3	0.662521	0.1788004	0.706368	0.706368	1	0.758498	FUM	fumarase	1.00 * Water [c] + 1.00 * Fumarate [c] --> 1.00 * (S)-malate	FH1	Citric acid cycle	4	4.2.1.2	0
FUMm_neg	3	0.662521	0.1788004	0.706368	0.706368	1	0.758498	FUMm	fumarase, mitochondrial	1.00 * Water [m] + 1.00 * Fumarate [m] --> 1.00 * (S)-mala	FH1	Citric acid cycle	4	4.2.1.2	0
FUMm_pos	3	0.662521	0.1788004	0.706368	0.706368	1	0.758498	FUMm	fumarase, mitochondrial	1.00 * Water [m] + 1.00 * Fumarate [m] --> 1.00 * (S)-mala	FH1	Citric acid cycle	4	4.2.1.2	0
r0081_neg	6	0.662521	0.1788004	-0.32478	0.324776	2	0.758498	r0081	L-Alanine:2-oxoglutarate aminotransferase Glutamate metabolism /	1.00 * 2-Oxoglutarate [m] + 1.00 * L-alanine [m] --> 1.00 *	GPT; GPT2	Citric acid cycle	4	2.6.1.2	0
r0081_pos	6	0.662521	0.1788004	-0.32478	0.324776	2	0.758498	r0081	L-Alanine:2-oxoglutarate aminotransferase Glutamate metabolism /	1.00 * 2-Oxoglutarate [m] + 1.00 * L-alanine [m] --> 1.00 *	GPT; GPT2	Citric acid cycle	4	2.6.1.2	0
ACONT_neg	4	1	0	-0.91613	0.916131	2	1	ACONT	aconitase	1.00 * Citrate [c] --> 1.00 * Isocitrate [c]ACO1; IREB2	ACO1; IREB	Citric acid cycle	4	4.2.1.3	0
ACONT_pos	4	1	0	-0.91613	0.916131	2	1	ACONT	aconitase	1.00 * Citrate [c] --> 1.00 * Isocitrate [c]ACO1; IREB2	ACO1; IREB	Citric acid cycle	4	4.2.1.3	0
ACONTm_neg	5	1	0	0.111631	0.111631	1	1	ACONTm	Aconitate hydratase	1.00 * Citrate [m] --> 1.00 * Isocitrate [m]ACO1; ACO2	ACO1; ACO	Citric acid cycle	4	4.2.1.3	0
ACONTm_pos	5	1	0	0.111631	0.111631	1	1	ACONTm	Aconitate hydratase	1.00 * Citrate [m] --> 1.00 * Isocitrate [m]ACO1; ACO2	ACO1; ACO	Citric acid cycle	4	4.2.1.3	0
ICDHxm_pos	5	1	0	0.576024	0.576024	1	1	ICDHxm	Isocitrate dehydrogenase (NAD+)	1.00 * Nicotinamide adenine dinucleotide [m] + 1.00 * Iso	IDH3A; IDH	Citric acid cycle	4	1.1.1.41	0
r0679_pos	3	0.662521	0.1788004	-0.27302	0.273018	2	0.758498	r0679	ATP:pantothenate 4-phosphotransferase Pantothenate and CoA bios	1.00 * ATP [c] + 1.00 * N-[(R)-pantothenoyl]-L-cysteinate [	PANK1; PAI	CoA catabolism	5	2.7.1.33	0
r0680_neg	3	0.662521	0.1788004	-0.27302	0.273018	2	0.758498	r0680	ATP:pantothenate 4-phosphotransferase Pantothenate and CoA bios	1.00 * ATP [m] + 1.00 * N-[(R)-pantothenoyl]-L-cysteinate	PANK1; PAI	CoA catabolism	5	2.7.1.33	0
PPCDC_pos	3	0.662521	0.1788004	-0.27302	0.273018	2	0.758498	PPCDC	phosphopantothenoylcysteine decarboxylase	1.00 * proton [c] + 1.00 * N-((R)-4-Phosphopantothenoyl)	PPCDC	CoA synthesis	5	4.1.1.36	4
r0578_neg	3	0.662521	0.1788004	-0.27302	0.273018	2	0.758498	r0578	ATP:pantothenate 4-phosphotransferase Pantothenate and CoA bios	1.00 * ATP [c] + 1.00 * pantetheine [c] --> 1.00 * ADP [c] + 1	PANK1; PAI	CoA synthesis	5	2.7.1.33	0
r0579_pos	3	0.662521	0.1788004	-0.27302	0.273018	2	0.758498	r0579	ATP:pantothenate 4-phosphotransferase Pantothenate and CoA bios	1.00 * ATP [m] + 1.00 * pantetheine [m] --> 1.00 * proton	PANK1; PAI	CoA synthesis	5	2.7.1.33	0
r0580_pos	3	0.662521	0.1788004	-0.78295	0.782951	2	0.758498	r0580	N-((R)-Pantothenoyl)-L-cysteine carboxy-lyase Pantothenate and CoA	1.00 * proton [c] + 1.00 * N-[(R)-pantothenoyl]-L-cysteina	1.00 * prot	CoA synthesis	5	4.1.1.30	0
r0614_neg	3	0.662521	0.1788004	-0.27302	0.273018	2	0.758498	r0614	N-[(R)-4-Phosphopantothenoyl]-L-cysteine carboxy-lyase Pantothen	1.00 * proton [m] + 1.00 * N-((R)-4-Phosphopantothenoyl	PPCDC	CoA synthesis	5	4.1.1.36	0
PYRt2p_pos	9	0.080856	1.0922877	5.692481	5.692481	1	0.240222	PYRt2p	pyruvate peroxisomal transport via proton symport	1.00 * proton [c] + 1.00 * pyruvate [c] --> 1.00 * proton [x]	SLC16A1; S	D-alanine metabolism	6		4
PYRt2p_neg	7	0.382733	0.4171041	0.858628	0.858628	1	0.518179	PYRt2p	pyruvate peroxisomal transport via proton symport	1.00 * proton [c] + 1.00 * pyruvate [c] --> 1.00 * proton [x]	SLC16A1; S	D-alanine metabolism	6		4
LTD4DP_neg	4	1	0	-0.00857	0.008568	2	1	LTD4DP	Leukotriene D4 dipeptidase	1.00 * Water [c] + 1.00 * Leukotriene D4 [c] --> 1.00 * Glyc	1.00 * Wat	Eicosanoid metabolism	7	2.3.2.2	4
LTD4DP_pos	4	1	0	-0.00857	0.008568	2	1	LTD4DP	Leukotriene D4 dipeptidase	1.00 * Water [c] + 1.00 * Leukotriene D4 [c] --> 1.00 * Glyc	1.00 * Wat	Eicosanoid metabolism	7	2.3.2.2	4
DM_sprm_c__pos	2	0.382733	0.4171041	-1.55903	1.559029	2	0.518179	DM_sprm_	DM sprm(c)	1.00 * spermine(4+) [c] --> Ï•No genes	1.00 * sper	Exchange/demand reaction	8		0
CSNATr_neg	9	0.080856	1.0922877	1.613406	1.613406	1	0.240222	CSNATr	carnitine O-acetyltransferase	1.00 * Acetyl-CoA [c] + 1.00 * L-Carnitine [c] --> 1.00 * Coe	CRAT	Fatty acid oxidation	9	2.3.1.7	4
CSNATr_pos	9	0.080856	1.0922877	1.613406	1.613406	1	0.240222	CSNATr	carnitine O-acetyltransferase	1.00 * Acetyl-CoA [c] + 1.00 * L-Carnitine [c] --> 1.00 * Coe	CRAT	Fatty acid oxidation	9	2.3.1.7	4
C181CPT1_pos	8	0.19043	0.7202646	1.376364	1.376364	1	0.347218	C181CPT1	transport of Octadecenoyl-CoA into mitochondrial matrix	1.00 * L-Carnitine [c] + 1.00 * Octadecenoyl-CoA (n-C18:1	CPT1A; CPT	Fatty acid oxidation	9	2.3.1.21	4
C181CPT2_pos	8	0.19043	0.7202646	1.376364	1.376364	1	0.347218	C181CPT2	transport of Octadecenoyl-CoA into mitochondrial matrix	1.00 * Coenzyme A [m] + 1.00 * octadecenoyl carnitine [m	CPT2	Fatty acid oxidation	9		4
ELAIDCRNt_pos	8	0.19043	0.7202646	1.376364	1.376364	1	0.347218	ELAIDCRNt	transport into the mitochondria (carnitine)	1.00 * Elaidic carnitine [c] --> 1.00 * Elaidic carnitine [m]S	SLC25A20	Fatty acid oxidation	9		4
FACOAL1821_neg	1	0.19043	0.7202646	-1.46238	1.462381	2	0.347218	FACOAL182	fatty-acid--CoA ligase	1.00 * ATP [c] + 1.00 * Coenzyme A [c] + 1.00 * linoleate [c	ACSBG2; AC	Fatty acid oxidation	9	6.2.1.3	4
OCD11COACPT1_ne	8	0.19043	0.7202646	1.381802	1.381802	1	0.347218	OCD11COA	transport of 11-octadecenoyl carnitine into the mitochondrial matr	1.00 * L-Carnitine [c] + 1.00 * 11-octadecenoylcoa [c] --> 1	CPT1A; CPT	Fatty acid oxidation	9		4
OCD11CRNCACT_ne	8	0.19043	0.7202646	1.381802	1.381802	1	0.347218	OCD11CRN	transport of 11-octadecenoyl carnitine into the mitochondrial matr	1.00 * 11-octadecenoyl carnitine [c] --> 1.00 * 11-octade	SLC25A20	Fatty acid oxidation	9		4
OCD11CRNCPT2_ne	8	0.19043	0.7202646	1.381802	1.381802	1	0.347218	OCD11CRN	transport of 11-octadecenoyl carnitine into the mitochondrial matr	1.00 * Coenzyme A [m] + 1.00 * 11-octadecenoyl carnitin	CPT2	Fatty acid oxidation	9		4
OCTDECCACT_neg	8	0.19043	0.7202646	1.376364	1.376364	1	0.347218	OCTDECCA	transport of Octadecenoyl-CoA into mitochondrial matrix	1.00 * octadecenoyl carnitine (hypothetical, position of d	SLC25A20	Fatty acid oxidation	9		4
OCTDECCPT1_neg	8	0.19043	0.7202646	1.376364	1.376364	1	0.347218	OCTDECCP	transport of Octadecenoyl-CoA into mitochondrial matrix	1.00 * L-Carnitine [c] + 1.00 * octadecenoylcoa [c] --> 1.00	CPT1A; CPT	Fatty acid oxidation	9		4
OCTDECCPT2_neg	8	0.19043	0.7202646	1.376364	1.376364	1	0.347218	OCTDECCP	transport of Octadecenoyl-CoA into mitochondrial matrix	1.00 * Coenzyme A [m] + 1.00 * octadecenoyl carnitine (h	CPT2	Fatty acid oxidation	9		4

VACCCPT1_pos	8	0.19043	0.7202646	1.381802	1.381802	1	0.347218	VACCCPT1	carnitine O-palmitoyltransferase	1.00 * L-Carnitine [c] + 1.00 * vaccenyl coenzyme A [c] -->	CPT1A; CPT	Fatty acid oxidation	9	2.3.1.21	4
VACCCPT2_pos	8	0.19043	0.7202646	1.381802	1.381802	1	0.347218	VACCCPT2	carnitine transferase	1.00 * Coenzyme A [m] + 1.00 * Vaccenyl carnitine [m] -->	CPT2	Fatty acid oxidation	9		4
VACCCRNt_pos	8	0.19043	0.7202646	1.381802	1.381802	1	0.347218	VACCCRNt	transport into the mitochondria (carnitine)	1.00 * Vaccenyl carnitine [c] --> 1.00 * Vaccenyl carnitine	SLC25A20	Fatty acid oxidation	9		4
C160CPT1_pos	7	0.382733	0.4171041	1.115609	1.115609	1	0.518179	C160CPT1	production of Palmitoylcarnitine	1.00 * L-Carnitine [c] + 1.00 * Palmitoyl-CoA (n-C16:0CoA	CPT1A; CPT	Fatty acid oxidation	9	2.3.1.21	4
C40CPT1_pos	7	0.382733	0.4171041	0.839865	0.839865	1	0.518179	C40CPT1	production of butyrylcarnitine	1.00 * L-Carnitine [c] + 1.00 * Butanoyl-CoA [c] --> 1.00 * (	CPT1A; CPT	Fatty acid oxidation	9		4
C4CRNCPT2_pos	7	0.382733	0.4171041	0.839865	0.839865	1	0.518179	C4CRNCPT2	transport of butyryl carnitine in the mitochondrial matrix for final h	1.00 * Coenzyme A [m] + 1.00 * butyryl carnitine [m] --> 1.	CPT2	Fatty acid oxidation	9		4
FACOAL1831_neg	7	0.382733	0.4171041	0.957852	0.957852	1	0.518179	FACOAL183	fatty-acid--CoA ligase	1.00 * ATP [c] + 1.00 * Coenzyme A [c] + 1.00 * gamma-linc	ACSBG2; AC	Fatty acid oxidation	9	6.2.1.3	4
RE3076X_neg	7	0.382733	0.4171041	0.858628	0.858628	1	0.518179	RE3076X	RE3076	1.00 * Nicotinamide adenine dinucleotide [x] + 1.00 * Wa	1.00 * Nico	Fatty acid oxidation	9	1.2.1.48	0
r0434_pos	7	0.382733	0.4171041	1.115609	1.115609	1	0.518179	r0434	Palmitoyl-CoA:L-carnitine O-palmitoyltransferase Fatty acid metabo	1.00 * L-Carnitine [r] + 1.00 * Palmitoyl-CoA (n-C16:0CoA	CPT1A; CPT	Fatty acid oxidation	9	2.3.1.21	0
r1257_pos	7	0.382733	0.4171041	0.938989	0.938989	1	0.518179	r1257	EC:6.2.1.3	1.00 * ATP [r] + 1.00 * Coenzyme A [r] + 1.00 * linoleate [r]	ACSBG2; AC	Fatty acid oxidation	9	6.2.1.3	0
r1260_pos	7	0.382733	0.4171041	0.957852	0.957852	1	0.518179	r1260	EC:6.2.1.3	1.00 * ATP [r] + 1.00 * Coenzyme A [r] + 1.00 * gamma-linc	ACSBG2; AC	Fatty acid oxidation	9	6.2.1.3	0
r1401_pos	7	0.382733	0.4171041	0.839865	0.839865	1	0.518179	r1401	Facilitated diffusion	1.00 * Butanoyl-CoA [m] + 1.00 * L-Carnitine [c] --> 1.00 *	1.00 * Buta	Fatty acid oxidation	9		0
C161CPT1_pos	6	0.662521	0.1788004	0.852074	0.852074	1	0.758498	C161CPT1	production of palmitoleoylcarnitine	1.00 * L-Carnitine [c] + 1.00 * Hexadecenoyl-CoA (n-C16:1	CPT1A; CPT	Fatty acid oxidation	9	2.3.1.21	4
C30CPT1_pos	6	0.662521	0.1788004	0.74055	0.74055	1	0.758498	C30CPT1	production of propionylcarnitine	1.00 * Propanoyl-CoA [c] + 1.00 * L-Carnitine [c] --> 1.00 *	CPT1A; CPT	Fatty acid oxidation	9		4
C60CPT1_pos	6	0.662521	0.1788004	0.518912	0.518912	1	0.758498	C60CPT1	production of hexanoylcarnitine	1.00 * L-Carnitine [c] + 1.00 * Hexanoyl-CoA (n-C6:0CoA) [	CPT1A; CPT	Fatty acid oxidation	9		4
C80CPT1_neg	6	0.662521	0.1788004	0.224924	0.224924	1	0.758498	C80CPT1	production of octanoylcarnitine	1.00 * L-Carnitine [c] + 1.00 * Octanoyl-CoA (n-C8:0CoA) [	CPT1A; CPT	Fatty acid oxidation	9		4
CSNAT2m_pos	6	0.662521	0.1788004	0.74055	0.74055	1	0.758498	CSNAT2m	carnitine O-aceyltransferase, mitochondrial	1.00 * Coenzyme A [m] + 1.00 * propionyl-carnitine [m] --	CRAT	Fatty acid oxidation	9	2.3.1.7	4
CSNAT2x_neg	3	0.662521	0.1788004	-0.29731	0.29731	2	0.758498	CSNAT2x	carnitine dimethyl nonanoyl transferase, revsible, peroxisomal	1.00 * L-Carnitine [x] + 1.00 * 4,8 dimethylnonanoyl-CoA	CRAT; CROT	Fatty acid oxidation	9	2.3.1.7	4
FACOAL161_pos	6	0.662521	0.1788004	0.852074	0.852074	1	0.758498	FACOAL161	fatty-acid--CoA ligase (hexadecenoate)	1.00 * ATP [c] + 1.00 * Coenzyme A [c] + 1.00 * Hexadecen	ACSBG2; AC	Fatty acid oxidation	9		4
PCRNtm_pos	6	0.662521	0.1788004	0.637578	0.637578	1	0.758498	PCRNtm	transport into the mitochondria from cytosol (carnitine)	1.00 * propionyl-carnitine [c] --> 1.00 * propionyl-carniti	SLC25A20	Fatty acid oxidation	9		4
RE2759X_neg	6	0.662521	0.1788004	0.160261	0.160261	1	0.758498	RE2759X	RE2759	1.00 * (2S)-pristanoyl-CoA [x] --> 1.00 * (2R)-pristanoyl-Co	AMACR	Fatty acid oxidation	9	5.1.99.4	0
RE2759X_pos	6	0.662521	0.1788004	0.160261	0.160261	1	0.758498	RE2759X	RE2759	1.00 * (2S)-pristanoyl-CoA [x] --> 1.00 * (2R)-pristanoyl-Co	AMACR	Fatty acid oxidation	9	5.1.99.4	0
RE3074X_neg	6	0.662521	0.1788004	0.160261	0.160261	1	0.758498	RE3074X	RE3074	1.00 * Coenzyme A [x] + 1.00 * ATP [x] + 1.00 * (2R,6S,10S	ACSL1; ACS	Fatty acid oxidation	9	6.2.1.3	0
RE3074X_pos	6	0.662521	0.1788004	0.160261	0.160261	1	0.758498	RE3074X	RE3074	1.00 * Coenzyme A [x] + 1.00 * ATP [x] + 1.00 * (2R,6S,10S	ACSL1; ACS	Fatty acid oxidation	9	6.2.1.3	0
RE3079X_neg	6	0.662521	0.1788004	0.160261	0.160261	1	0.758498	RE3079X	RE3079	1.00 * Coenzyme A [x] + 1.00 * ATP [x] + 1.00 * (2R,6S,10S	SLC27A2	Fatty acid oxidation	9		0
RE3079X_pos	6	0.662521	0.1788004	0.160261	0.160261	1	0.758498	RE3079X	RE3079	1.00 * Coenzyme A [x] + 1.00 * ATP [x] + 1.00 * (2R,6S,10S	SLC27A2	Fatty acid oxidation	9		0
r0444_neg	6	0.662521	0.1788004	0.899335	0.899335	1	0.758498	r0444	Palmitoyl-CoA:L-carnitine O-palmitoyltransferase Fatty acid metabo	1.00 * L-Carnitine [r] + 1.00 * palmitoleyl-CoA(4-) [r] --> 1.	CPT1A; CPT	Fatty acid oxidation	9	2.3.1.21	0
r0445_neg	6	0.662521	0.1788004	0.852074	0.852074	1	0.758498	r0445	Palmitoyl-CoA:L-carnitine O-palmitoyltransferase Fatty acid metabo	1.00 * L-Carnitine [c] + 1.00 * palmitoleyl-CoA(4-) [c] --> 1.	IDH1; IDH2	Fatty acid oxidation	9	2.3.1.21	0
r0445_pos	6	0.662521	0.1788004	0.742638	0.742638	1	0.758498	r0445	Palmitoyl-CoA:L-carnitine O-palmitoyltransferase Fatty acid metabo	1.00 * L-Carnitine [c] + 1.00 * palmitoleyl-CoA(4-) [c] --> 1.	IDH1; IDH2	Fatty acid oxidation	9	2.3.1.21	0
r0637_pos	6	0.662521	0.1788004	0.224924	0.224924	1	0.758498	r0637	Octanoyl-CoA:L-carnitine O-octanoyltransferase EC:2.3.1.137	1.00 * Octanoyl-CoA (n-C8:0CoA) [x] + 1.00 * L-Carnitine [	CROT	Fatty acid oxidation	9	2.3.1.137	0
r1400_pos	6	0.662521	0.1788004	0.564266	0.564266	1	0.758498	r1400	Active transport	1.00 * Propanoyl-CoA [c] + 1.00 * L-Carnitine [m] --> 1.00	1.00 * Prop	Fatty acid oxidation	9		0
r1487_neg	6	0.662521	0.1788004	0.899335	0.899335	1	0.758498	r1487	EC:6.2.1.3	1.00 * ATP [r] + 1.00 * Coenzyme A [r] + 1.00 * Hexadecen	ACSBG2; AC	Fatty acid oxidation	9	6.2.1.3	0
r1488_neg	6	0.662521	0.1788004	0.852074	0.852074	1	0.758498	r1488	EC:6.2.1.3	1.00 * ATP [c] + 1.00 * Coenzyme A [c] + 1.00 * Hexadecen	1.00 * ATP	Fatty acid oxidation	9	6.2.1.3	0
r1488_pos	6	0.662521	0.1788004	0.742638	0.742638	1	0.758498	r1488	EC:6.2.1.3	1.00 * ATP [c] + 1.00 * Coenzyme A [c] + 1.00 * Hexadecen	1.00 * ATP	Fatty acid oxidation	9	6.2.1.3	0
C161CRNt_pos	5	1	0	0.333815	0.333815	1	1	C161CRNt	C161 transport into the mitochondria	1.00 * Hexadecenoyl carnitine [c] --> 1.00 * Hexadecenoyl	SLC25A20	Fatty acid oxidation	9		4
CSNAT2x_pos	4	1	0	-0.07871	0.078708	2	1	CSNAT2x	carnitine dimethyl nonanoyl transferase, revsible, peroxisomal	1.00 * L-Carnitine [x] + 1.00 * 4,8 dimethylnonanoyl-CoA	CRAT; CROT	Fatty acid oxidation	9	2.3.1.7	4
CSNAT3x_pos	4	1	0	-0.11711	0.117112	2	1	CSNAT3x	carnitine O-aceyltransferase, peroxisomal	1.00 * L-Carnitine [x] + 1.00 * Propanoyl-CoA [x] --> 1.00 *	CRAT	Fatty acid oxidation	9	2.3.1.7	4
FACOAL1812_neg	4	1	0	0.355967	0.355967	1	1	FACOAL181	fatty-acid--CoA ligase	1.00 * ATP [c] + 1.00 * Coenzyme A [c] + 1.00 * trans-vacce	ACSL1	Fatty acid oxidation	9	6.2.1.3	4
FAOXC101C8m_pos	4	1	0	-0.48616	0.486157	2	1	FAOXC101(	fatty acid beta oxidation(C10:1-->C8)m	1.00 * Water [m] + 1.00 * Coenzyme A [m] + 1.00 * Nicotir	ACAA2; ECH	Fatty acid oxidation	9		4
PCRNtc_pos	4	1	0	0.04023	0.04023	1	1	PCRNtc	transport into the cytosol from peroxisome (carnitine)	1.00 * propionyl-carnitine [x] --> 1.00 * propionyl-carniti	1.00 * prop	Fatty acid oxidation	9		4
RE3248X_neg	5	1	0	0.038147	0.038147	1	1	RE3248X	RE3248	1.00 * Water [x] + 1.00 * 3alpha,7alpha,12alpha-Trihydro	EHHADH	Fatty acid oxidation	9	4.2.1.17	0
RE3250X_neg	5	1	0	0.038147	0.038147	1	1	RE3250X	RE3250	1.00 * Nicotinamide adenine dinucleotide [x] + 1.00 * 24(	EHHADH; H	Fatty acid oxidation	9	1.1.1.35	0
r0634_pos	4	1	0	-0.48616	0.486157	2	1	r0634	Octanoyl-CoA:acetyl-CoA C-acyltransferase Fatty acid elongation in r	1.00 * Acetyl-CoA [m] + 1.00 * Octanoyl-CoA (n-C8:0CoA)	ACAA1B; AC	Fatty acid oxidation	9	2.3.1.16	0
r0641_neg	4	1	0	-0.00857	0.008568	2	1	r0641	(5-Glutamyl)-peptide:amino-acid 5-glutamyltransferase Arachidonic	1.00 * Water [c] + 1.00 * leukotriene C4(2-) [c] --> 1.00 * L	GGT1; GGT	Fatty acid oxidation	9	2.3.2.2	0
r0641_pos	4	1	0	-0.00857	0.008568	2	1	r0641	(5-Glutamyl)-peptide:amino-acid 5-glutamyltransferase Arachidonic	1.00 * Water [c] + 1.00 * leukotriene C4(2-) [c] --> 1.00 * L	GGT1; GGT	Fatty acid oxidation	9	2.3.2.2	0
r0726_neg	4	1	0	-0.48616	0.486157	2	1	r0726	(S)-Hydroxydecanoyl-CoA:NAD+ oxidoreductase Fatty acid elongatio	1.00 * Nicotinamide adenine dinucleotide [m] + 1.00 * (S	EHHADH; H	Fatty acid oxidation	9	1.1.1.35	0
r0728_pos	4	1	0	-0.48616	0.486157	2	1	r0728	(S)-Hydroxydecanoyl-CoA hydro-lyase Fatty acid elongation in mitoc	1.00 * (S)-Hydroxydecanoyl-CoA [m] --> 1.00 * Water [m] -	ECHS1; EHH	Fatty acid oxidation	9	4.2.1.17	0
r0224_pos	1	0.19043	0.7202646	-1.44404	1.444038	2	0.347218	r0224	5,6,7,8-Tetrahydrofolate:NAD+ oxidoreductase One carbon pool by	1.00 * proton [c] + 1.00 * Nicotinamide adenine dinucleo	DHFR	Folate metabolism	10	1.5.1.3	0
r0512_pos	1	0.19043	0.7202646	-0.99725	0.997247	2	0.347218	r0512	Dihydrofolate:NAD+ oxidoreductase Folate biosynthesis EC:1.5.1.3	1.00 * Nicotinamide adenine dinucleotide [c] + 1.00 * 7,8	DHFR	Folate metabolism	10	1.5.1.3	0
MTHFDm_neg	7	0.382733	0.4171041	1.18664	1.18664	1	0.518179	MTHFDm	methylenetetrahydrofolate dehydrogenase (NADP), mitochondrial	1.00 * Nicotinamide adenine dinucleotide phosphate [m]	MTHFD1; M	Folate metabolism	10	1.5.1.5	4
MTHFDm_pos	7	0.382733	0.4171041	1.18664	1.18664	1	0.518179	MTHFDm	methylenetetrahydrofolate dehydrogenase (NADP), mitochondrial	1.00 * Nicotinamide adenine dinucleotide phosphate [m]	MTHFD1; M	Folate metabolism	10	1.5.1.5	4
MTHFD_neg	6	0.662521	0.1788004	-0.28968	0.289683	2	0.758498	MTHFD	methylenetetrahydrofolate dehydrogenase (NADP)	1.00 * Nicotinamide adenine dinucleotide phosphate [c]	MTHFD1; M	Folate metabolism	10	1.5.1.5	4
MTHFD_pos	3	0.662521	0.1788004	-0.75694	0.756944	2	0.758498	MTHFD	methylenetetrahydrofolate dehydrogenase (NADP)	1.00 * Nicotinamide adenine dinucleotide phosphate [c]	MTHFD1; M	Folate metabolism	10	1.5.1.5	4
r0792_pos	3	0.662521	0.1788004	-0.79078	0.790781	2	0.758498	r0792	5-methyltetrahydrofolate:NAD+ oxidoreductase One carbon pool by	1.00 * Nicotinamide adenine dinucleotide [c] + 1.00 * 5-M	MTHFR	Folate metabolism	10	1.5.1.20	0
r0191_pos	1	0.19043	0.7202646	-1.28899	1.288988	2	0.347218	r0191	UTP:D-fructose-6-phosphate 1-phosphotransferase EC:2.7.1.11	1.00 * UTP [c] + 1.00 * D-Fructose 6-phosphate [c] --> 1.00	PFKL	Fructose and mannose metabolism	11	2.7.1.11	0
GALT_neg	1	0.19043	0.7202646	-1.98565	1.985646	2	0.347218	GALT	galactose-1-phosphate uridylyltransferase	1.00 * proton [c] + 1.00 * UTP [c] + 1.00 * (2R,3R,4S,5R,6R	GALT	Galactose metabolism	12	2.7.7.12	0
GALT_pos	1	0.19043	0.7202646	-1.98565	1.985646	2	0.347218	GALT	galactose-1-phosphate uridylyltransferase	1.00 * proton [c] + 1.00 * UTP [c] + 1.00 * (2R,3R,4S,5R,6R	GALT	Galactose metabolism	12	2.7.7.12	0
GALU_neg	1	0.19043	0.7202646	-1.98565	1.985646	2	0.347218	GALU	UTP-glucose-1-phosphate uridylyltransferase	1.00 * proton [c] + 1.00 * D-Glucose 1-phosphate [c] + 1.0	UGP2	Galactose metabolism	12	2.7.7.9	0
GALU_pos	1	0.19043	0.7202646	-1.98565	1.985646	2	0.347218	GALU	UTP-glucose-1-phosphate uridylyltransferase	1.00 * proton [c] + 1.00 * D-Glucose 1-phosphate [c] + 1.0	UGP2	Galactose metabolism	12	2.7.7.9	0
UGLT_neg	1	0.19043	0.7202646	-1.98565	1.985646	2	0.347218	UGLT	UDPglucose--hexose-1-phosphate uridylyltransferase	1.00 * (2R,3R,4S,5R,6R)-3,4,5-trihydroxy-6-(hydroxymet	GALT	Galactose metabolism	12	2.7.7.12	0
UGLT_pos	1	0.19043	0.7202646	-1.98565	1.985646	2	0.347218	UGLT	UDPglucose--hexose-1-phosphate uridylyltransferase	1.00 * (2R,3R,4S,5R,6R)-3,4,5-trihydroxy-6-(hydroxymet	GALT	Galactose metabolism	12	2.7.7.12	0
GLUDxm_neg	0	0.080856	1.0922877	-1.79286	1.792864	2	0.240222	GLUDxm	glutamate dehydrogenase (NAD) (mitochondrial)	1.00 * Water [m] + 1.00 * Nicotinamide adenine dinucleo	GLUD1	Glutamate metabolism	13	1.4.1.3	4
GLUDxm_pos	0	0.080856	1.0922877	-1.79286	1.792864	2	0.240222	GLUDxm	glutamate dehydrogenase (NAD) (mitochondrial)	1.00 * Water [m] + 1.00 * Nicotinamide adenine dinucleo	GLUD1	Glutamate metabolism	13	1.4.1.3	4
GLUDym_neg	0	0.080856	1.0922877	-1.79286	1.792864	2	0.240222	GLUDym	glutamate dehydrogenase (NADP), mitochondrial	1.00 * Water [m] + 1.00 * Nicotinamide adenine dinucleo	GLUD1	Glutamate metabolism	13	1.4.1.4	4
GLUDym_pos	0	0.080856	1.0922877	-1.79286	1.792864	2	0.240222	GLUDym	glutamate dehydrogenase (NADP), mitochondrial	1.00 * Water [m] + 1.00 * Nicotinamide adenine dinucleo	GLUD1	Glutamate metabolism	13	1.4.1.4	4
r0178_neg	0	0.080856	1.0922877	-1.46179	1.461791	2	0.240222	r0178	Succinate-semialdehyde:NAD+ oxidoreductase Glutamate metabolis	1.00 * Water [m] + 1.00 * Nicotinamide adenine dinucleo	ALDH5A1	Glutamate metabolism	13	1.2.1.24	0
r0179_pos	0	0.080856	1.0922877	-1.46179	1.461791	2	0.240222	r0179	Succinate-semialdehyde:NADP+ oxidoreductase Glutamate metabol	1.00 * Water [m] + 1.00 * Nicotinamide adenine dinucleo	1.00 * Wat	Glutamate metabolism	13	1.2.1.16	0
r0464_neg	0	0.080856	1.0922877	-1.79286	1.792864	2	0.240222	r0464	4-Aminobutyraldehyde:NAD+ oxidoreductase Urea cycle and metab	1.00 * Water [m] + 1.00 * Nicotinamide adenine dinucleo	ALDH1B1; A	Glutamate metabolism	13	1.2.1.3	0
r0464_pos	0	0.080856	1.0922877	-1.79286	1.792864	2	0.240222	r0464	4-Aminobutyraldehyde:NAD+ oxidoreductase Urea cycle and metab	1.00 * Water [m] + 1.00 * Nicotinamide adenine dinucleo	ALDH1B1; A	Glutamate metabolism	13	1.2.1.3	0
r0549_neg	0	0.080856	1.0922877	-1.79286	1.792864	2	0.240222	r0549	4-aminobutanal:NAD+ 1-oxidoreductase; 4-aminobutyraldehyde:NA	1.00 * Water [m] + 1.00 * Nicotinamide adenine dinucleo	ALDH1B1; A	Glutamate metabolism	13	1.2.1.3	0
r0549_pos	0	0.080856	1.0922877	-1.79286	1.792864	2	0.240222	r0549	4-aminobutanal:NAD+ 1-oxidoreductase; 4-aminobutyraldehyde:NA	1.00 * Water [m] + 1.00 * Nicotinamide adenine dinucleo	ALDH1B1; A	Glutamate metabolism	13	1.2.1.3	0
ALATA_L_pos	7	0.382733	0.4171041	0.954814	0.954814	1	0.518179	ALATA_L	L-alanine transaminase	1.00 * 2-Oxoglutarate [c] + 1.00 * L-alanine [c] --> 1.00 * p	GPT; GPT2	Glutamate metabolism	13	2.6.1.2	4
P5CDm_pos	7	0.382733	0.4171041	1.164738	1.164738	1	0.518179	P5CDm	1-pyrroline-5-carboxylate dehydrogenase, mitochondrial	2.00 * Water [m] + 1.00 * Nicotinamide adenine dinucleo	ALDH4A1	Glutamate metabolism	13	1.5.1.12	4
r0129_neg	7	0.382733	0.4171041	1.422001	1.422001	1	0.518179	r0129	gamma-glutamyltranspeptidase Glutathione metabolism EC:3.4.11.	1.00 * Water [c] + 1.00 * Reduced glutathione [c] --> 1.00	GGT1; GGT	Glutathione metabolism	14	3.4.11.4	0
r0130_pos	7	0.382733	0.4171041	1.422001	1.422001	1	0.518179	r0130	gamma-glutamyltranspeptidase Glutathione metabolism EC:3.4.11.	1.00 * Water [e] + 1.00 * Reduced glutathione [e] --> 1.00	GGT1; GGT	Glutathione metabolism	14	3.4.11.4	0
r0021_pos	6	0.662521	0.1788004	0.787793	0.787793	1	0.758498	r0021	glutathione:NAD+ oxidoreductase Glutamate metabolism EC:1.8.1.	1.00 * Nicotinamide adenine dinucleotide [c] + 2.00 * Re	GSR	Glutathione metabolism	14	1.8.1.7	0
r0021_neg	5	1	0	0.170212	0.170212	1	1	r0021	glutathione:NAD+ oxidoreductase Glutamate metabolism EC:1.8.1.	1.00 * Nicotinamide adenine dinucleotide [c] + 2.00 * Re	GSR	Glutathione metabolism	14	1.8.1.7	0
r0022_pos	5	1	0	0.088133	0.088133	1	1	r0022	glutathione:NAD+ oxidoreductase Glutamate metabolism EC:1.8.1.	1.00 * Nicotinamide adenine dinucleotide [m] + 2.00 * Re	GSR	Glutathione metabolism	14	1.8.1.7	0
G3PD1_pos	7	0.382733	0.4171041	0.954814	0.954814	1	0.518179	G3PD1	glycerol-3-phosphate dehydrogenase (NAD)	1.00 * Nicotinamide adenine dinucleotide [c] + 1.00 * Gly	GPD1	Glycerophospholipid metabolism	15	1.1.1.94	4
GLYK_pos	7	0.382733	0.4171041	0.954814	0.954814	1	0.518179	GLYK	glycerol kinase	1.00 * ATP [c] + 1.00 * Glycerol [c] --> 1.00 * ADP [c] + 1.00	GK; GK2	Glycerophospholipid metabolism	15		4
r0245_neg	7	0.382733	0.4171041	0.954814	0.954814	1	0.518179	r0245	Glycerol:NADP+ oxidoreductase Glycerolipid metabolism EC:1.1.1.2	1.00 * Nicotinamide adenine dinucleotide phosphate [c]	AKR1A1; AK	Glycerophospholipid metabolism	15	1.1.1.72	0
PCHOLP_hs_pos	3	0.662521	0.1788004	-0.96585	0.965851	2	0.758498	PCHOLP_hs	choline phosphatase	1.00 * Water [c] + 1.00 * Phosphatidylcholine [c] --> 1.00	PLD1; PLD2	Glycerophospholipid metabolism	15	3.1.4.4	4
PCHOLPg_hs_pos	3	0.662521	0.1788004	-1.04961	1.049612	2	0.758498	PCHOLPg_h	choline phosphatase	1.00 * Water [g] + 1.00 * Phosphatidylcholine [g] --> 1.00	PLD1	Glycerophospholipid metabolism	15	3.1.4.4	4

PCHOLPr_hs_pos	3	0.662521	0.1788004	-1.04961	1.049612	2	0.758498	PCHOLPr_h	choline phosphatase	1.00 * Water [r] + 1.00 * Phosphatidylcholine [r] --> 1.00	PLD1	Glycerophospholipid metabolism	15	3.1.4.4	4
PSSA1_hs_neg	3	0.662521	0.1788004	-0.96585	0.965851	2	0.758498	PSSA1_hs	Phosphatidylserine synthase homo sapiens	1.00 * L-serine [c] + 1.00 * Phosphatidylcholine [c] --> 1.0	PTDSS1	Glycerophospholipid metabolism	15	2.7.8.8	4
RE3301C_neg	3	0.662521	0.1788004	-0.96585	0.965851	2	0.758498	RE3301C	RE3301	1.00 * Water [c] + 1.00 * phosphatidylserine [c] --> 1.00 *	PLD2	Glycerophospholipid metabolism	15	3.1.4.4	0
r0552_neg	0	0.080856	1.0922877	-2.51343	2.51343	2	0.240222	r0552	p-cumic alcohol:NAD+ oxidoreductase Glycine, serine and threonine	1.00 * Water [c] + 1.00 * Nicotinamide adenine dinucleot	ALDH7A1	Glycine, serine, alanine and threonine metabolism	16	1.2.1.8	0
r0552_pos	0	0.080856	1.0922877	-1.56317	1.563172	2	0.240222	r0552	p-cumic alcohol:NAD+ oxidoreductase Glycine, serine and threonine	1.00 * Water [c] + 1.00 * Nicotinamide adenine dinucleot	ALDH7A1	Glycine, serine, alanine and threonine metabolism	16	1.2.1.8	0
r0553_neg	0	0.080856	1.0922877	-1.56317	1.563172	2	0.240222	r0553	p-Cumic alcohol:NADP+ oxidoreductase Glycine, serine and threoni	1.00 * Water [c] + 1.00 * Nicotinamide adenine dinucleot	ALDH7A1	Glycine, serine, alanine and threonine metabolism	16	1.2.1.8	0
r0553_pos	0	0.080856	1.0922877	-2.51343	2.51343	2	0.240222	r0553	p-Cumic alcohol:NADP+ oxidoreductase Glycine, serine and threoni	1.00 * Water [c] + 1.00 * Nicotinamide adenine dinucleot	ALDH7A1	Glycine, serine, alanine and threonine metabolism	16	1.2.1.8	0
GCC2am_pos	1	0.19043	0.7202646	-1.73098	1.730978	2	0.347218	GCC2am	glycine-cleavage complex (lipoamide), mitochondrial	1.00 * proton [m] + 1.00 * Glycine [m] + 1.00 * lipoamide	AMT; DLD;	Glycine, serine, alanine and threonine metabolism	16	2.1.2.10	4
GCC2bim_pos	1	0.19043	0.7202646	-1.73098	1.730978	2	0.347218	GCC2bim	glycine-cleavage system (lipoamide) irreversible, mitochondrial	1.00 * 5,6,7,8-Tetrahydrofolate [m] + 1.00 * S-aminomet	AMT; DLD;	Glycine, serine, alanine and threonine metabolism	16	1.8.1.4	4
GCC2cm_pos	1	0.19043	0.7202646	-1.73098	1.730978	2	0.347218	GCC2cm	glycine-cleavage complex (lipoamide), mitochondrial	1.00 * Nicotinamide adenine dinucleotide [m] + 1.00 * di	AMT; DLD;	Glycine, serine, alanine and threonine metabolism	16	2.1.2.10	4
GCCam_pos	1	0.19043	0.7202646	-1.73098	1.730978	2	0.347218	GCCam	glycine-cleavage complex (lipoylprotein), mitochondrial	1.00 * proton [m] + 1.00 * Glycine [m] + 1.00 * Lipoylprot	AMT; DLD;	Glycine, serine, alanine and threonine metabolism	16	2.1.2.10	4
GCCbim_pos	1	0.19043	0.7202646	-1.73098	1.730978	2	0.347218	GCCbim	glycine-cleavage complex (lipoylprotein) irreversible, mitochondria	1.00 * 5,6,7,8-Tetrahydrofolate [m] + 1.00 * S-Aminomet	AMT; DLD;	Glycine, serine, alanine and threonine metabolism	16	1.8.1.4	4
GCCcm_pos	1	0.19043	0.7202646	-1.73098	1.730978	2	0.347218	GCCcm	glycine-cleavage complex (lipoylprotein), mitochondrial	1.00 * Nicotinamide adenine dinucleotide [m] + 1.00 * Di	AMT; DLD;	Glycine, serine, alanine and threonine metabolism	16	2.1.2.10	4
r0295_pos	1	0.19043	0.7202646	-1.73098	1.730978	2	0.347218	r0295	glycine synthase Nitrogen metabolism EC:2.1.2.10	1.00 * carbon dioxide [m] + 1.00 * Nicotinamide adenine	AMT	Glycine, serine, alanine and threonine metabolism	16	2.1.2.10	0
PGCD_pos	7	0.382733	0.4171041	0.954814	0.954814	1	0.518179	PGCD	phosphoglycerate dehydrogenase	1.00 * Nicotinamide adenine dinucleotide [c] + 1.00 * 3-P	PHGDH	Glycine, serine, alanine and threonine metabolism	16	1.1.1.95	4
PSERT_pos	7	0.382733	0.4171041	0.954814	0.954814	1	0.518179	PSERT	phosphoserine transaminase	1.00 * L-glutamate(1-) [c] + 1.00 * 3-phosphonatooxypyru	PSAT1	Glycine, serine, alanine and threonine metabolism	16	2.6.1.52	4
PSP_L_pos	7	0.382733	0.4171041	0.954814	0.954814	1	0.518179	PSP_L	phosphoserine phosphatase (L-serine)	1.00 * Water [c] + 1.00 * proton [c] + 1.00 * O-Phospho-L-	PSPH	Glycine, serine, alanine and threonine metabolism	16	3.1.3.3	4
r0160_pos	7	0.382733	0.4171041	0.954814	0.954814	1	0.518179	r0160	L-Serine:pyruvate aminotransferase Glycine, serine and threonine m	1.00 * pyruvate [c] + 1.00 * L-serine [c] --> 1.00 * L-alanine	AGXT	Glycine, serine, alanine and threonine metabolism	16	2.6.1.51	0
r0173_neg	9	0.080856	1.0922877	5.692481	5.692481	1	0.240222	r0173	(S)-Lactate:NAD+ oxidoreductase Glycolysis / Gluconeogenesis / Pyru	1.00 * Nicotinamide adenine dinucleotide [x] + 1.00 * (S)-	LDHA; LDH	Glycolysis/gluconeogenesis	17	1.1.1.27	0
FBA_pos	1	0.19043	0.7202646	-1.46042	1.460419	2	0.347218	FBA	fructose-bisphosphate aldolase	1.00 * D-Fructose 1,6-bisphosphate [c] --> 1.00 * Dihydro	ALDOART2	Glycolysis/gluconeogenesis	17	4.1.2.13	0
GAPD_pos	7	0.382733	0.4171041	0.954814	0.954814	1	0.518179	GAPD	glyceraldehyde-3-phosphate dehydrogenase	1.00 * hydrogenphosphate [c] + 1.00 * Nicotinamide ade	GAPDHS; G	Glycolysis/gluconeogenesis	17	1.2.1.12	0
LDH_L_neg	7	0.382733	0.4171041	0.858628	0.858628	1	0.518179	LDH_L	L-lactate dehydrogenase	1.00 * Nicotinamide adenine dinucleotide [c] + 1.00 * (S)-	LDHA; LDH	Glycolysis/gluconeogenesis	17	1.1.1.27	0
PGK_neg	7	0.382733	0.4171041	0.954814	0.954814	1	0.518179	PGK	phosphoglycerate kinase	1.00 * ATP [c] + 1.00 * 3-Phospho-D-glycerate [c] --> 1.00	MIA3; PGK	Glycolysis/gluconeogenesis	17		0
TPI_pos	7	0.382733	0.4171041	0.954814	0.954814	1	0.518179	TPI	triose-phosphate isomerase	1.00 * Dihydroxyacetone phosphate [c] --> 1.00 * Glycera	TPI1	Glycolysis/gluconeogenesis	17	5.3.1.1	0
r0173_pos	7	0.382733	0.4171041	0.858628	0.858628	1	0.518179	r0173	(S)-Lactate:NAD+ oxidoreductase Glycolysis / Gluconeogenesis / Pyru	1.00 * Nicotinamide adenine dinucleotide [x] + 1.00 * (S)-	LDHA; LDH	Glycolysis/gluconeogenesis	17	1.1.1.27	0
LDH_L_pos	3	0.662521	0.1788004	-0.61458	0.61458	2	0.758498	LDH_L	L-lactate dehydrogenase	1.00 * Nicotinamide adenine dinucleotide [c] + 1.00 * (S)-	LDHA; LDH	Glycolysis/gluconeogenesis	17	1.1.1.27	0
PYK_pos	4	1	0	0.406305	0.406305	1	1	PYK	pyruvate kinase	1.00 * ADP [c] + 1.00 * proton [c] + 1.00 * Phosphoenolpy	PKLR; PKM	Glycolysis/gluconeogenesis	17		0
r0122_pos	4	1	0	0.331588	0.331588	1	1	r0122	GTP:pyruvate O2-phosphotransferase Purine metabolism EC:2.7.1.4	1.00 * proton [c] + 1.00 * Phosphoenolpyruvate [c] + 1.00	PKLR	Glycolysis/gluconeogenesis	17	2.7.1.40	0
r0153_pos	4	1	0	0.331588	0.331588	1	1	r0153	CTP:pyruvate O2-phosphotransferase EC:2.7.1.40	1.00 * proton [c] + 1.00 * Phosphoenolpyruvate [c] + 1.00	PKLR	Glycolysis/gluconeogenesis	17	2.7.1.40	0
r0165_pos	4	1	0	0.331588	0.331588	1	1	r0165	UTP:pyruvate O2-phosphotransferase EC:2.7.1.40	1.00 * proton [c] + 1.00 * UDP [c] + 1.00 * Phosphoenolpy	PKLR	Glycolysis/gluconeogenesis	17	2.7.1.40	0
r0280_pos	4	1	0	0.331588	0.331588	1	1	r0280	dATP:pyruvate O2-phosphotransferase Purine metabolism EC:2.7.1.	1.00 * proton [c] + 1.00 * Phosphoenolpyruvate [c] + 1.00	PKLR	Glycolysis/gluconeogenesis	17	2.7.1.40	0
r0413_pos	4	1	0	0.331588	0.331588	1	1	r0413	dATP:pyruvate O2-phosphotransferase Purine metabolism EC:2.7.1.	1.00 * proton [c] + 1.00 * Phosphoenolpyruvate [c] + 1.00	PKLR	Glycolysis/gluconeogenesis	17	2.7.1.40	0
HPYRRy_pos	7	0.382733	0.4171041	0.954814	0.954814	1	0.518179	HPYRRy	Hydroxypyruvate reductase (NADPH)	1.00 * proton [c] + 1.00 * Nicotinamide adenine dinucleo	GRHPR	Glyoxylate and dicarboxylate metabolism	18	1.1.1.79	4
SACCD4m_pos	1	0.19043	0.7202646	-1.40385	1.403852	2	0.347218	SACCD4m	saccharopine dehydrogenase (NAD, L-glutamate forming), mitochon	1.00 * Water [m] + 1.00 * Nicotinamide adenine dinucleo	AASS	Lysine metabolism	19	1.5.1.10	4
r0525_neg	1	0.19043	0.7202646	-1.40385	1.403852	2	0.347218	r0525	N6-(L-1,3-Dicarboxypropyl)-L-lysine:NAD+ oxidoreductase; N6-(L-1,	1.00 * Water [m] + 1.00 * Nicotinamide adenine dinucleo	AASS	Lysine metabolism	19	1.5.1.9	0
2AMADPTm_pos	3	0.662521	0.1788004	-0.85086	0.850863	2	0.758498	2AMADPTm	L-2-aminoadipate shuttle (cytosol/mitochondria)	1.00 * L-2-aminoadipate(1-) [c] + 1.00 * 2-Oxoglutarate [n	SLC25A21	Lysine metabolism	19		4
AATAi_neg	3	0.662521	0.1788004	-0.85086	0.850863	2	0.758498	AATAi	2-aminoadipate transaminase, irreversible	1.00 * L-2-aminoadipate(1-) [c] + 1.00 * 2-Oxoglutarate [c	AADAT	Lysine metabolism	19	2.6.1.39	4
r0450_pos	3	0.662521	0.1788004	-0.85086	0.850863	2	0.758498	r0450	L-2-Aminoadipate:2-oxoglutarate aminotransferase Lysine biosynth	1.00 * 2-Oxoglutarate [m] + 1.00 * L-2-aminoadipate(1-) [	AADAT	Lysine metabolism	19	2.6.1.39	0
CYSGLTH_pos	8	0.19043	0.7202646	1.095385	1.095385	1	0.347218	CYSGLTH	Glutathione:cystine oxidoreductase	1.00 * L-cystine [c] + 2.00 * Reduced glutathione [c] --> 2.0	1.00 * L-cys	Methionine and cysteine metabolism	20	1.8.4.4	4
r0027_pos	8	0.19043	0.7202646	1.095385	1.095385	1	0.347218	r0027	NADPH:CoA-glutathione oxidoreductase EC:1.8.1.9	1.00 * Nicotinamide adenine dinucleotide phosphate [c]	TXNRD1; T	Methionine and cysteine metabolism	20	1.8.1.9	0
CYSGLTH_neg	7	0.382733	0.4171041	1.076682	1.076682	1	0.518179	CYSGLTH	Glutathione:cystine oxidoreductase	1.00 * L-cystine [c] + 2.00 * Reduced glutathione [c] --> 2.0	1.00 * L-cys	Methionine and cysteine metabolism	20	1.8.4.4	4
r0027_neg	7	0.382733	0.4171041	1.076682	1.076682	1	0.518179	r0027	NADPH:CoA-glutathione oxidoreductase EC:1.8.1.9	1.00 * Nicotinamide adenine dinucleotide phosphate [c]	TXNRD1; T	Methionine and cysteine metabolism	20	1.8.1.9	0
H2CO3Dm_neg	0	0.080856	1.0922877	-2.31899	2.318987	2	0.240222	H2CO3Dm	carboxylic acid dissociation	1.00 * Water [m] + 1.00 * carbon dioxide [m] --> 1.00 * pr	CAR5A; CA	Miscellaneous		4.2.1.1	4
H2CO3Dm_pos	0	0.080856	1.0922877	-2.94107	2.941075	2	0.240222	H2CO3Dm	carboxylic acid dissociation	1.00 * Water [m] + 1.00 * carbon dioxide [m] --> 1.00 * pr	CAR5A; CA	Miscellaneous		4.2.1.1	4
H2CO3D_pos	0	0.080856	1.0922877	-1.79567	1.795669	2	0.240222	H2CO3D	carboxylic acid dissociation	1.00 * Water [c] + 1.00 * carbon dioxide [c] --> 1.00 * prot	CAR1; CAR	Miscellaneous		4.2.1.1	0
H2CO3D_neg	1	0.19043	0.7202646	-1.43187	1.431873	2	0.347218	H2CO3D	carboxylic acid dissociation	1.00 * Water [c] + 1.00 * carbon dioxide [c] --> 1.00 * prot	CAR1; CAR	Miscellaneous		4.2.1.1	0
RE0383C_pos	3	0.662521	0.1788004	-0.79031	0.790314	2	0.758498	RE0383C	RE0383	1.00 * Nicotinamide adenine dinucleotide phosphate [c]	AKR1A1	Miscellaneous		1.1.1.2	0
THD1m_pos	0	0.080856	1.0922877	-2.43874	2.43874	2	0.240222	THD1m	NAD(P) transhydrogenase	1.00 * Nicotinamide adenine dinucleotide phosphate [m	NNT	NAD metabolism	21	1.6.1.2	4
CYTK10_neg	9	0.080856	1.0922877	2.263577	2.263577	1	0.240222	CYTK10	cytidylate kinase (CMP,dGTP)	1.00 * CMP [c] + 1.00 * dGTP [c] --> 1.00 * CDP [c] + 1.00 *	CMPK1	Nucleotide interconversion	22	2.7.4.14	4
CYTK10_pos	9	0.080856	1.0922877	2.263577	2.263577	1	0.240222	CYTK10	cytidylate kinase (CMP,dGTP)	1.00 * CMP [c] + 1.00 * dGTP [c] --> 1.00 * CDP [c] + 1.00 *	CMPK1	Nucleotide interconversion	22	2.7.4.14	4
CYTK11_neg	9	0.080856	1.0922877	2.263577	2.263577	1	0.240222	CYTK11	cytidylate kinase (dCMP,dGTP)	1.00 * dGTP [c] + 1.00 * dCMP [c] --> 1.00 * dGDP [c] + 1.0	CMPK1	Nucleotide interconversion	22	2.7.4.14	4
CYTK11_pos	9	0.080856	1.0922877	2.263577	2.263577	1	0.240222	CYTK11	cytidylate kinase (dCMP,dGTP)	1.00 * dGTP [c] + 1.00 * dCMP [c] --> 1.00 * dGDP [c] + 1.0	CMPK1	Nucleotide interconversion	22	2.7.4.14	4
CYTK12_neg	9	0.080856	1.0922877	2.263577	2.263577	1	0.240222	CYTK12	cytidylate kinase (dCMP,dCTP)	1.00 * dCMP [c] + 1.00 * dCTP [c] --> 2.00 * dCDP [c]CMPK	CMPK1	Nucleotide interconversion	22	2.7.4.14	4
CYTK12_pos	9	0.080856	1.0922877	2.263577	2.263577	1	0.240222	CYTK12	cytidylate kinase (dCMP,dCTP)	1.00 * dCMP [c] + 1.00 * dCTP [c] --> 2.00 * dCDP [c]CMPK	CMPK1	Nucleotide interconversion	22	2.7.4.14	4
CYTK13_neg	9	0.080856	1.0922877	2.263577	2.263577	1	0.240222	CYTK13	cytidylate kinase (dCMP,dATP)	1.00 * dATP [c] + 1.00 * dCMP [c] --> 1.00 * dADP [c] + 1.00	CMPK1	Nucleotide interconversion	22	2.7.4.14	4
CYTK13_pos	9	0.080856	1.0922877	2.263577	2.263577	1	0.240222	CYTK13	cytidylate kinase (dCMP,dATP)	1.00 * dATP [c] + 1.00 * dCMP [c] --> 1.00 * dADP [c] + 1.00	CMPK1	Nucleotide interconversion	22	2.7.4.14	4
CYTK14_neg	9	0.080856	1.0922877	2.263577	2.263577	1	0.240222	CYTK14	cytidylate kinase (dCMP,UTP)	1.00 * UTP [c] + 1.00 * dCMP [c] --> 1.00 * UDP [c] + 1.00 *	CMPK1	Nucleotide interconversion	22	2.7.4.14	4
CYTK14_pos	9	0.080856	1.0922877	2.263577	2.263577	1	0.240222	CYTK14	cytidylate kinase (dCMP,UTP)	1.00 * UTP [c] + 1.00 * dCMP [c] --> 1.00 * UDP [c] + 1.00 *	CMPK1	Nucleotide interconversion	22	2.7.4.14	4
CYTK1_neg	9	0.080856	1.0922877	2.295253	2.295253	1	0.240222	CYTK1	cytidylate kinase (CMP)	1.00 * ATP [c] + 1.00 * CMP [c] --> 1.00 * ADP [c] + 1.00 * C	CMPK1	Nucleotide interconversion	22		4
CYTK1_pos	9	0.080856	1.0922877	2.295253	2.295253	1	0.240222	CYTK1	cytidylate kinase (CMP)	1.00 * ATP [c] + 1.00 * CMP [c] --> 1.00 * ADP [c] + 1.00 * C	CMPK1	Nucleotide interconversion	22		4
CYTK2_neg	9	0.080856	1.0922877	2.295253	2.295253	1	0.240222	CYTK2	cytidylate kinase (dCMP)	1.00 * ATP [c] + 1.00 * dCMP [c] --> 1.00 * ADP [c] + 1.00 *	CMPK1	Nucleotide interconversion	22		4
CYTK2_pos	9	0.080856	1.0922877	2.295253	2.295253	1	0.240222	CYTK2	cytidylate kinase (dCMP)	1.00 * ATP [c] + 1.00 * dCMP [c] --> 1.00 * ADP [c] + 1.00 *	CMPK1	Nucleotide interconversion	22		4
CYTK3_neg	9	0.080856	1.0922877	2.295253	2.295253	1	0.240222	CYTK3	cytidylate kinase (CMP)(GTP)	1.00 * GTP [c] + 1.00 * CMP [c] --> 1.00 * GDP [c] + 1.00 * C	AK5	Nucleotide interconversion	22		4
CYTK3_pos	9	0.080856	1.0922877	2.295253	2.295253	1	0.240222	CYTK3	cytidylate kinase (CMP)(GTP)	1.00 * GTP [c] + 1.00 * CMP [c] --> 1.00 * GDP [c] + 1.00 * C	AK5	Nucleotide interconversion	22		4
CYTK4_neg	9	0.080856	1.0922877	2.295253	2.295253	1	0.240222	CYTK4	cytidylate kinase (dCMP)(GTP)	1.00 * GTP [c] + 1.00 * dCMP [c] --> 1.00 * GDP [c] + 1.00 *	AK5	Nucleotide interconversion	22		4
CYTK4_pos	9	0.080856	1.0922877	2.295253	2.295253	1	0.240222	CYTK4	cytidylate kinase (dCMP)(GTP)	1.00 * GTP [c] + 1.00 * dCMP [c] --> 1.00 * GDP [c] + 1.00 *	AK5	Nucleotide interconversion	22		4
CYTK5_neg	9	0.080856	1.0922877	2.263577	2.263577	1	0.240222	CYTK5	cytidylate kinase (dCMP)	1.00 * CTP [c] + 1.00 * dCMP [c] --> 1.00 * CDP [c] + 1.00 *	CMPK1	Nucleotide interconversion	22	2.7.4.14	4
CYTK5_pos	9	0.080856	1.0922877	2.263577	2.263577	1	0.240222	CYTK5	cytidylate kinase (dCMP)	1.00 * CTP [c] + 1.00 * dCMP [c] --> 1.00 * CDP [c] + 1.00 *	CMPK1	Nucleotide interconversion	22	2.7.4.14	4
CYTK6_neg	9	0.080856	1.0922877	2.263577	2.263577	1	0.240222	CYTK6	cytidylate kinase (CMP,CTP)	1.00 * CMP [c] + 1.00 * CTP [c] --> 2.00 * CDP [c]CMPK1	CMPK1	Nucleotide interconversion	22	2.7.4.14	4
CYTK6_pos	9	0.080856	1.0922877	2.263577	2.263577	1	0.240222	CYTK6	cytidylate kinase (CMP,CTP)	1.00 * CMP [c] + 1.00 * CTP [c] --> 2.00 * CDP [c]CMPK1	CMPK1	Nucleotide interconversion	22	2.7.4.14	4
CYTK7_neg	9	0.080856	1.0922877	2.263577	2.263577	1	0.240222	CYTK7	cytidylate kinase (CMP,UTP)	1.00 * CMP [c] + 1.00 * UTP [c] --> 1.00 * UDP [c] + 1.00 * C	CMPK1	Nucleotide interconversion	22	2.7.4.14	4
CYTK7_pos	9	0.080856	1.0922877	2.263577	2.263577	1	0.240222	CYTK7	cytidylate kinase (CMP,UTP)	1.00 * CMP [c] + 1.00 * UTP [c] --> 1.00 * UDP [c] + 1.00 * C	CMPK1	Nucleotide interconversion	22	2.7.4.14	4
CYTK8_neg	9	0.080856	1.0922877	2.263577	2.263577	1	0.240222	CYTK8	cytidylate kinase (CMP,dATP)	1.00 * dATP [c] + 1.00 * CMP [c] --> 1.00 * dADP [c] + 1.00	CMPK1	Nucleotide interconversion	22	2.7.4.14	4
CYTK8_pos	9	0.080856	1.0922877	2.263577	2.263577	1	0.240222	CYTK8	cytidylate kinase (CMP,dATP)	1.00 * dATP [c] + 1.00 * CMP [c] --> 1.00 * dADP [c] + 1.00	CMPK1	Nucleotide interconversion	22	2.7.4.14	4
CYTK9_neg	9	0.080856	1.0922877	2.263577	2.263577	1	0.240222	CYTK9	cytidylate kinase (CMP,dCTP)	1.00 * CMP [c] + 1.00 * dCTP [c] --> 1.00 * CDP [c] + 1.00 *	CMPK1	Nucleotide interconversion	22	2.7.4.14	4
CYTK9_pos	9	0.080856	1.0922877	2.263577	2.263577	1	0.240222	CYTK9	cytidylate kinase (CMP,dCTP)	1.00 * CMP [c] + 1.00 * dCTP [c] --> 1.00 * CDP [c] + 1.00 *	CMPK1	Nucleotide interconversion	22	2.7.4.14	4
DCK1n_neg	9	0.080856	1.0922877	1.541946	1.541946	1	0.240222	DCK1n	Deoxycytidine kinase, nuclear (ATP)	1.00 * ATP [n] + 1.00 * Deoxycytidine [n] --> 1.00 * proton	DCK	Nucleotide interconversion	22	2.7.1.74	4
DCK1n_pos	9	0.080856	1.0922877	1.541946	1.541946	1	0.240222	DCK1n	Deoxycytidine kinase, nuclear (ATP)	1.00 * ATP [n] + 1.00 * Deoxycytidine [n] --> 1.00 * proton	DCK	Nucleotide interconversion	22	2.7.1.74	4
DCK2n_neg	9	0.080856	1.0922877	1.541946	1.541946	1	0.240222	DCK2n	Deoxycytidine kinase, nuclear (UTP)	1.00 * UTP [n] + 1.00 * Deoxycytidine [n] --> 1.00 * proton	DCK	Nucleotide interconversion	22	2.7.1.74	4
DCK2n_pos	9	0.080856	1.0922877	1.541946	1.541946	1	0.240222	DCK2n	Deoxycytidine kinase, nuclear (UTP)	1.00 * UTP [n] + 1.00 * Deoxycytidine [n] --> 1.00 * proton	DCK	Nucleotide interconversion	22	2.7.1.74	4
NDPK1_neg	9	0.080856	1.0922877	2.295253	2.295253	1	0.240222	NDPK1	nucleoside-diphosphate kinase (ATP:GDP)	1.00 * ATP [c] + 1.00 * GDP [c] --> 1.00 * ADP [c] + 1.00 * G	GM20390;	Nucleotide interconversion	22		4
NDPK1_pos	9	0.080856	1.0922877	2.295253	2.295253	1	0.240222	NDPK1	nucleoside-diphosphate kinase (ATP:GDP)	1.00 * ATP [c] + 1.00 * GDP [c] --> 1.00 * ADP [c] + 1.00 * G	GM20390;	Nucleotide interconversion	22		4
NDPK2_neg	9	0.080856	1.0922877	2.263577	2.263577	1	0.240222	NDPK2	nucleoside-diphosphate kinase (ATP:UDP)	1.00 * ATP [c] + 1.00 * UDP [c] --> 1.00 * ADP [c] + 1.00 * U	GM20390;	Nucleotide interconversion	22		4

NDPK2_pos	9	0.080856	1.0922877	2.263577	2.263577	1	0.240222	NDPK2	nucleoside-diphosphate kinase (ATP:UDP)	1.00 * ATP [c] + 1.00 * UDP [c] --> 1.00 * ADP [c] + 1.00 * U	GM20390;	Nucleotide interconversion	22		4
NDPK3_neg	9	0.080856	1.0922877	2.263577	2.263577	1	0.240222	NDPK3	nucleoside-diphosphate kinase (ATP:CDP)	1.00 * ATP [c] + 1.00 * CDP [c] --> 1.00 * ADP [c] + 1.00 * C	GM20390;	Nucleotide interconversion	22		4
NDPK3_pos	9	0.080856	1.0922877	2.263577	2.263577	1	0.240222	NDPK3	nucleoside-diphosphate kinase (ATP:CDP)	1.00 * ATP [c] + 1.00 * CDP [c] --> 1.00 * ADP [c] + 1.00 * C	GM20390;	Nucleotide interconversion	22		4
NDPK5_neg	9	0.080856	1.0922877	2.263577	2.263577	1	0.240222	NDPK5	Nucleoside-diphosphate kinase (ATP:dGDP)	1.00 * ATP [c] + 1.00 * dGDP [c] --> 1.00 * ADP [c] + 1.00 *	GM20390;	Nucleotide interconversion	22		4
NDPK5_pos	9	0.080856	1.0922877	2.263577	2.263577	1	0.240222	NDPK5	Nucleoside-diphosphate kinase (ATP:dGDP)	1.00 * ATP [c] + 1.00 * dGDP [c] --> 1.00 * ADP [c] + 1.00 *	GM20390;	Nucleotide interconversion	22		4
NDPK6_pos	9	0.080856	1.0922877	2.013967	2.013967	1	0.240222	NDPK6	Nucleoside-diphosphate kinase (ATP:dUDP)	1.00 * ATP [c] + 1.00 * dUDP [c] --> 1.00 * ADP [c] + 1.00 *	GM20390;	Nucleotide interconversion	22		4
NDPK7_neg	9	0.080856	1.0922877	2.263577	2.263577	1	0.240222	NDPK7	nucleoside-diphosphate kinase (ATP:dCDP)	1.00 * ATP [c] + 1.00 * dCDP [c] --> 1.00 * ADP [c] + 1.00 * d	GM20390;	Nucleotide interconversion	22		4
NDPK7_pos	9	0.080856	1.0922877	2.263577	2.263577	1	0.240222	NDPK7	nucleoside-diphosphate kinase (ATP:dCDP)	1.00 * ATP [c] + 1.00 * dCDP [c] --> 1.00 * ADP [c] + 1.00 * d	GM20390;	Nucleotide interconversion	22		4
NDPK8_neg	9	0.080856	1.0922877	2.263577	2.263577	1	0.240222	NDPK8	Nucleoside-diphosphate kinase (ATP:dADP)	1.00 * ATP [c] + 1.00 * dADP [c] --> 1.00 * ADP [c] + 1.00 * d	GM20390;	Nucleotide interconversion	22		4
NDPK8_pos	9	0.080856	1.0922877	2.263577	2.263577	1	0.240222	NDPK8	Nucleoside-diphosphate kinase (ATP:dADP)	1.00 * ATP [c] + 1.00 * dADP [c] --> 1.00 * ADP [c] + 1.00 * d	GM20390;	Nucleotide interconversion	22		4
PUNP7_neg	0	0.080856	1.0922877	-5.40841	5.408411	2	0.240222	PUNP7	purine-nucleoside phosphorylase (Xanthosine)	1.00 * hydrogenphosphate [c] + 1.00 * xanthosine [c] --> 1	PNP2	Nucleotide interconversion	22	3.4.2.1	4
RNDR3_pos	9	0.080856	1.0922877	1.801074	1.801074	1	0.240222	RNDR3	ribonucleoside-diphosphate reductase (CDP)	1.00 * CDP [c] + 1.00 * Reduced thioredoxin [c] --> 1.00 *	RRM1; RRM	Nucleotide interconversion	22	1.7.14.1	4
UMPK2_neg	9	0.080856	1.0922877	2.263577	2.263577	1	0.240222	UMPK2	UMP kinase (CTP)	1.00 * CTP [c] + 1.00 * UMP [c] --> 1.00 * UDP [c] + 1.00 * C	CMPK1	Nucleotide interconversion	22	2.7.4.14	4
UMPK2_pos	9	0.080856	1.0922877	2.263577	2.263577	1	0.240222	UMPK2	UMP kinase (CTP)	1.00 * CTP [c] + 1.00 * UMP [c] --> 1.00 * UDP [c] + 1.00 * C	CMPK1	Nucleotide interconversion	22	2.7.4.14	4
UMPK3_neg	9	0.080856	1.0922877	2.263577	2.263577	1	0.240222	UMPK3	UMP kinase (UTP)	1.00 * UTP [c] + 1.00 * UMP [c] --> 2.00 * UDP [c]CMPK1	CMPK1	Nucleotide interconversion	22	2.7.4.14	4
UMPK3_pos	9	0.080856	1.0922877	2.263577	2.263577	1	0.240222	UMPK3	UMP kinase (UTP)	1.00 * UTP [c] + 1.00 * UMP [c] --> 2.00 * UDP [c]CMPK1	CMPK1	Nucleotide interconversion	22	2.7.4.14	4
UMPK4_neg	9	0.080856	1.0922877	2.263577	2.263577	1	0.240222	UMPK4	UMP kinase (GTP)	1.00 * GTP [c] + 1.00 * UMP [c] --> 1.00 * UDP [c] + 1.00 * C	CMPK1	Nucleotide interconversion	22	2.7.4.14	4
UMPK4_pos	9	0.080856	1.0922877	2.263577	2.263577	1	0.240222	UMPK4	UMP kinase (GTP)	1.00 * GTP [c] + 1.00 * UMP [c] --> 1.00 * UDP [c] + 1.00 * C	CMPK1	Nucleotide interconversion	22	2.7.4.14	4
UMPK5_neg	9	0.080856	1.0922877	2.263577	2.263577	1	0.240222	UMPK5	UMP kinase (dATP)	1.00 * dATP [c] + 1.00 * UMP [c] --> 1.00 * UDP [c] + 1.00 *	CMPK1	Nucleotide interconversion	22	2.7.4.14	4
UMPK5_pos	9	0.080856	1.0922877	2.263577	2.263577	1	0.240222	UMPK5	UMP kinase (dATP)	1.00 * dATP [c] + 1.00 * UMP [c] --> 1.00 * UDP [c] + 1.00 *	CMPK1	Nucleotide interconversion	22	2.7.4.14	4
UMPK6_neg	9	0.080856	1.0922877	2.263577	2.263577	1	0.240222	UMPK6	UMP kinase (dCTP)	1.00 * dCTP [c] + 1.00 * UMP [c] --> 1.00 * UDP [c] + 1.00 *	CMPK1	Nucleotide interconversion	22	2.7.4.14	4
UMPK6_pos	9	0.080856	1.0922877	2.263577	2.263577	1	0.240222	UMPK6	UMP kinase (dCTP)	1.00 * dCTP [c] + 1.00 * UMP [c] --> 1.00 * UDP [c] + 1.00 *	CMPK1	Nucleotide interconversion	22	2.7.4.14	4
UMPK7_neg	9	0.080856	1.0922877	2.263577	2.263577	1	0.240222	UMPK7	UMP kinase (dGTP)	1.00 * dGTP [c] + 1.00 * UMP [c] --> 1.00 * UDP [c] + 1.00 *	CMPK1	Nucleotide interconversion	22	2.7.4.14	4
UMPK7_pos	9	0.080856	1.0922877	2.263577	2.263577	1	0.240222	UMPK7	UMP kinase (dGTP)	1.00 * dGTP [c] + 1.00 * UMP [c] --> 1.00 * UDP [c] + 1.00 *	CMPK1	Nucleotide interconversion	22	2.7.4.14	4
UMPK_neg	9	0.080856	1.0922877	2.263577	2.263577	1	0.240222	UMPK	UMP kinase	1.00 * ATP [c] + 1.00 * UMP [c] --> 1.00 * ADP [c] + 1.00 * U	CMPK1	Nucleotide interconversion	22		4
UMPK_pos	9	0.080856	1.0922877	2.263577	2.263577	1	0.240222	UMPK	UMP kinase	1.00 * ATP [c] + 1.00 * UMP [c] --> 1.00 * ADP [c] + 1.00 * U	CMPK1	Nucleotide interconversion	22		4
r0472_pos	9	0.080856	1.0922877	1.835454	1.835454	1	0.240222	r0472	2-Deoxyadenosine 5-diphosphate:oxidized-thioredoxin 2-oxidoredu	1.00 * Water [c] + 1.00 * dGTP [c] + 1.00 * Oxidized thiore	RRM1; RRM	Nucleotide interconversion	22	1.17.4.1	0
r0474_pos	9	0.080856	1.0922877	1.801074	1.801074	1	0.240222	r0474	2-Deoxyadenosine 5-diphosphate:oxidized-thioredoxin 2-oxidoredu	1.00 * Water [c] + 1.00 * dCTP [c] + 1.00 * Oxidized thiore	RRM1; RRM	Nucleotide interconversion	22	1.17.4.1	0
r0475_pos	9	0.080856	1.0922877	2.013967	2.013967	1	0.240222	r0475	2-Deoxyadenosine 5-diphosphate:oxidized-thioredoxin 2-oxidoredu	1.00 * Water [c] + 1.00 * dUTP [c] + 1.00 * Oxidized thiore	RRM1; RRM	Nucleotide interconversion	22	1.17.4.1	0
r0531_neg	9	0.080856	1.0922877	1.563427	1.563427	1	0.240222	r0531	dUTP:cytidine 5-phosphotransferase Pyrimidine metabolism EC:2.7	1.00 * Cytidine [c] + 1.00 * dUTP [c] --> 1.00 * proton [c] +	UCK1; UCK	Nucleotide interconversion	22	2.7.1.48	0
r1384_neg	0	0.080856	1.0922877	-5.40841	5.408411	2	0.240222	r1384	Guanosine aminohydrolase EC:3.5.4.15	1.00 * Water [c] + 1.00 * proton [c] + 1.00 * Guanosine [c]	1.00 * Wat	Nucleotide interconversion	22	3.5.4.15	0
ADK1m_neg	1	0.19043	0.7202646	-1.45529	1.455287	2	0.347218	ADK1m	adenylate kinase, mitochondrial	1.00 * ATP [m] + 1.00 * AMP [m] --> 2.00 * ADP [m]AK2; AK	AK2; AK4	Nucleotide interconversion	22	2.7.4.3	4
ADK1m_pos	1	0.19043	0.7202646	-1.45529	1.455287	2	0.347218	ADK1m	adenylate kinase, mitochondrial	1.00 * ATP [m] + 1.00 * AMP [m] --> 2.00 * ADP [m]AK2; AK	AK2; AK4	Nucleotide interconversion	22	2.7.4.3	4
ADK3m_neg	1	0.19043	0.7202646	-1.45529	1.455287	2	0.347218	ADK3m	adentylate kinase (GTP)	1.00 * AMP [m] + 1.00 * GTP [m] --> 1.00 * ADP [m] + 1.00	AK3	Nucleotide interconversion	22		4
ADK3m_pos	1	0.19043	0.7202646	-1.45529	1.455287	2	0.347218	ADK3m	adentylate kinase (GTP)	1.00 * AMP [m] + 1.00 * GTP [m] --> 1.00 * ADP [m] + 1.00	AK3	Nucleotide interconversion	22		4
r0470_pos	8	0.19043	0.7202646	1.709019	1.709019	1	0.347218	r0470	2-Deoxyadenosine 5-diphosphate:oxidized-thioredoxin 2-oxidoredu	1.00 * Water [c] + 1.00 * dATP [c] + 1.00 * Oxidized thiore	RRM1; RRM	Nucleotide interconversion	22	1.17.4.1	0
CYTK10n_neg	7	0.382733	0.4171041	1.077892	1.077892	1	0.518179	CYTK10n	cytidylate kinase (CMP,dGTP),nuclear	1.00 * CMP [n] + 1.00 * dGTP [n] --> 1.00 * CDP [n] + 1.00 *	CMPK1	Nucleotide interconversion	22	2.7.4.14	4
CYTK10n_pos	7	0.382733	0.4171041	1.077892	1.077892	1	0.518179	CYTK10n	cytidylate kinase (CMP,dGTP),nuclear	1.00 * CMP [n] + 1.00 * dGTP [n] --> 1.00 * CDP [n] + 1.00 *	CMPK1	Nucleotide interconversion	22	2.7.4.14	4
CYTK11n_neg	7	0.382733	0.4171041	1.077892	1.077892	1	0.518179	CYTK11n	cytidylate kinase (dCMP,dGTP),nuclear	1.00 * dGTP [n] + 1.00 * dCMP [n] --> 1.00 * dGDP [n] + 1.0	CMPK1	Nucleotide interconversion	22	2.7.4.14	4
CYTK11n_pos	7	0.382733	0.4171041	1.077892	1.077892	1	0.518179	CYTK11n	cytidylate kinase (dCMP,dGTP),nuclear	1.00 * dGTP [n] + 1.00 * dCMP [n] --> 1.00 * dGDP [n] + 1.0	CMPK1	Nucleotide interconversion	22	2.7.4.14	4
CYTK12n_neg	7	0.382733	0.4171041	1.077892	1.077892	1	0.518179	CYTK12n	cytidylate kinase (dCMP,dCTP),nuclear	1.00 * dCMP [n] + 1.00 * dCTP [n] --> 2.00 * dCDP [n]CMPK	CMPK1	Nucleotide interconversion	22	2.7.4.14	4
CYTK12n_pos	7	0.382733	0.4171041	1.077892	1.077892	1	0.518179	CYTK12n	cytidylate kinase (dCMP,dCTP),nuclear	1.00 * dCMP [n] + 1.00 * dCTP [n] --> 2.00 * dCDP [n]CMPK	CMPK1	Nucleotide interconversion	22	2.7.4.14	4
CYTK13n_neg	7	0.382733	0.4171041	1.077892	1.077892	1	0.518179	CYTK13n	cytidylate kinase (dCMP,dATP),nuclear	1.00 * dCMP [n] + 1.00 * dATP [n] --> 1.00 * dCDP [n] + 1.0	CMPK1	Nucleotide interconversion	22	2.7.4.14	4
CYTK13n_pos	7	0.382733	0.4171041	1.077892	1.077892	1	0.518179	CYTK13n	cytidylate kinase (dCMP,dATP),nuclear	1.00 * dCMP [n] + 1.00 * dATP [n] --> 1.00 * dCDP [n] + 1.0	CMPK1	Nucleotide interconversion	22	2.7.4.14	4
CYTK14n_neg	7	0.382733	0.4171041	1.077892	1.077892	1	0.518179	CYTK14n	cytidylate kinase (dCMP,CTP),nuclear	1.00 * dCMP [n] + 1.00 * UTP [n] --> 1.00 * dCDP [n] + 1.00	CMPK1	Nucleotide interconversion	22	2.7.4.14	4
CYTK14n_pos	7	0.382733	0.4171041	1.077892	1.077892	1	0.518179	CYTK14n	cytidylate kinase (dCMP,CTP),nuclear	1.00 * dCMP [n] + 1.00 * UTP [n] --> 1.00 * dCDP [n] + 1.00	CMPK1	Nucleotide interconversion	22	2.7.4.14	4
CYTK1n_neg	7	0.382733	0.4171041	1.077892	1.077892	1	0.518179	CYTK1n	cytidylate kinase (CMP),nuclear	1.00 * ATP [n] + 1.00 * CMP [n] --> 1.00 * CDP [n] + 1.00 * A	CMPK1	Nucleotide interconversion	22	2.7.4.14	4
CYTK1n_pos	7	0.382733	0.4171041	1.077892	1.077892	1	0.518179	CYTK1n	cytidylate kinase (CMP),nuclear	1.00 * ATP [n] + 1.00 * CMP [n] --> 1.00 * CDP [n] + 1.00 * A	CMPK1	Nucleotide interconversion	22	2.7.4.14	4
CYTK2n_neg	7	0.382733	0.4171041	1.077892	1.077892	1	0.518179	CYTK2n	cytidylate kinase (dCMP),nuclear	1.00 * ATP [n] + 1.00 * dCMP [n] --> 1.00 * dCDP [n] + 1.00	CMPK1	Nucleotide interconversion	22	2.7.4.14	4
CYTK2n_pos	7	0.382733	0.4171041	1.077892	1.077892	1	0.518179	CYTK2n	cytidylate kinase (dCMP),nuclear	1.00 * ATP [n] + 1.00 * dCMP [n] --> 1.00 * dCDP [n] + 1.00	CMPK1	Nucleotide interconversion	22	2.7.4.14	4
CYTK3n_neg	7	0.382733	0.4171041	1.077892	1.077892	1	0.518179	CYTK3n	cytidylate kinase (dCMP,CTP),nuclear	1.00 * CTP [n] + 1.00 * dCMP [n] --> 1.00 * CDP [n] + 1.00 *	CMPK1	Nucleotide interconversion	22	2.7.4.14	4
CYTK3n_pos	7	0.382733	0.4171041	1.077892	1.077892	1	0.518179	CYTK3n	cytidylate kinase (dCMP,CTP),nuclear	1.00 * CTP [n] + 1.00 * dCMP [n] --> 1.00 * CDP [n] + 1.00 *	CMPK1	Nucleotide interconversion	22	2.7.4.14	4
CYTK4n_neg	7	0.382733	0.4171041	1.077892	1.077892	1	0.518179	CYTK4n	cytidylate kinase (dCMP,GTP),nuclear	1.00 * dCMP [n] + 1.00 * GTP [n] --> 1.00 * dCDP [n] + 1.00	CMPK1	Nucleotide interconversion	22	2.7.4.14	4
CYTK4n_pos	7	0.382733	0.4171041	1.077892	1.077892	1	0.518179	CYTK4n	cytidylate kinase (dCMP,GTP),nuclear	1.00 * dCMP [n] + 1.00 * GTP [n] --> 1.00 * dCDP [n] + 1.00	CMPK1	Nucleotide interconversion	22	2.7.4.14	4
CYTK5n_neg	7	0.382733	0.4171041	1.077892	1.077892	1	0.518179	CYTK5n	cytidylate kinase (CMP),nuclear	1.00 * CMP [n] + 1.00 * GTP [n] --> 1.00 * CDP [n] + 1.00 * C	CMPK1	Nucleotide interconversion	22	2.7.4.14	4
CYTK5n_pos	7	0.382733	0.4171041	1.077892	1.077892	1	0.518179	CYTK5n	cytidylate kinase (CMP),nuclear	1.00 * CMP [n] + 1.00 * GTP [n] --> 1.00 * CDP [n] + 1.00 * C	CMPK1	Nucleotide interconversion	22	2.7.4.14	4
CYTK6n_neg	7	0.382733	0.4171041	1.077892	1.077892	1	0.518179	CYTK6n	cytidylate kinase (CMP,CTP),nuclear	1.00 * CTP [n] + 1.00 * CMP [n] --> 2.00 * CDP [n]CMPK1	CMPK1	Nucleotide interconversion	22	2.7.4.14	4
CYTK6n_pos	7	0.382733	0.4171041	1.077892	1.077892	1	0.518179	CYTK6n	cytidylate kinase (CMP,CTP),nuclear	1.00 * CTP [n] + 1.00 * CMP [n] --> 2.00 * CDP [n]CMPK1	CMPK1	Nucleotide interconversion	22	2.7.4.14	4
CYTK7n_neg	7	0.382733	0.4171041	1.077892	1.077892	1	0.518179	CYTK7n	cytidylate kinase (CMP,UTP),nuclear	1.00 * CMP [n] + 1.00 * UTP [n] --> 1.00 * CDP [n] + 1.00 * U	CMPK1	Nucleotide interconversion	22	2.7.4.14	4
CYTK7n_pos	7	0.382733	0.4171041	1.077892	1.077892	1	0.518179	CYTK7n	cytidylate kinase (CMP,UTP),nuclear	1.00 * CMP [n] + 1.00 * UTP [n] --> 1.00 * CDP [n] + 1.00 * U	CMPK1	Nucleotide interconversion	22	2.7.4.14	4
CYTK8n_neg	7	0.382733	0.4171041	1.077892	1.077892	1	0.518179	CYTK8n	cytidylate kinase (CMP,dATP),nuclear	1.00 * CMP [n] + 1.00 * dATP [n] --> 1.00 * CDP [n] + 1.00 *	CMPK1	Nucleotide interconversion	22	2.7.4.14	4
CYTK8n_pos	7	0.382733	0.4171041	1.077892	1.077892	1	0.518179	CYTK8n	cytidylate kinase (CMP,dATP),nuclear	1.00 * CMP [n] + 1.00 * dATP [n] --> 1.00 * CDP [n] + 1.00 *	CMPK1	Nucleotide interconversion	22	2.7.4.14	4
CYTK9n_neg	7	0.382733	0.4171041	1.077892	1.077892	1	0.518179	CYTK9n	cytidylate kinase (CMP,dCTP),nuclear	1.00 * CMP [n] + 1.00 * dCTP [n] --> 1.00 * CDP [n] + 1.00 *	CMPK1	Nucleotide interconversion	22	2.7.4.14	4
CYTK9n_pos	7	0.382733	0.4171041	1.077892	1.077892	1	0.518179	CYTK9n	cytidylate kinase (CMP,dCTP),nuclear	1.00 * CMP [n] + 1.00 * dCTP [n] --> 1.00 * CDP [n] + 1.00 *	CMPK1	Nucleotide interconversion	22	2.7.4.14	4
NDPK4_neg	7	0.382733	0.4171041	1.039213	1.039213	1	0.518179	NDPK4	nucleoside-diphosphate kinase (ATP:dTDP)	1.00 * ATP [c] + 1.00 * dTDP [c] --> 1.00 * ADP [c] + 1.00 * d	GM20390;	Nucleotide interconversion	22		4
UMPK2n_neg	7	0.382733	0.4171041	1.077892	1.077892	1	0.518179	UMPK2n	UMP kinase (CTP),nuclear	1.00 * CTP [n] + 1.00 * UMP [n] --> 1.00 * CDP [n] + 1.00 * U	CMPK1	Nucleotide interconversion	22	2.7.4.14	4
UMPK2n_pos	7	0.382733	0.4171041	1.077892	1.077892	1	0.518179	UMPK2n	UMP kinase (CTP),nuclear	1.00 * CTP [n] + 1.00 * UMP [n] --> 1.00 * CDP [n] + 1.00 * U	CMPK1	Nucleotide interconversion	22	2.7.4.14	4
UMPK3n_neg	7	0.382733	0.4171041	1.077892	1.077892	1	0.518179	UMPK3n	UMP kinase (UTP),nuclear	1.00 * UTP [n] + 1.00 * UMP [n] --> 2.00 * UDP [n]CMPK1	CMPK1	Nucleotide interconversion	22	2.7.4.14	4
UMPK3n_pos	7	0.382733	0.4171041	1.077892	1.077892	1	0.518179	UMPK3n	UMP kinase (UTP),nuclear	1.00 * UTP [n] + 1.00 * UMP [n] --> 2.00 * UDP [n]CMPK1	CMPK1	Nucleotide interconversion	22	2.7.4.14	4
UMPK4n_neg	7	0.382733	0.4171041	1.077892	1.077892	1	0.518179	UMPK4n	UMP kinase (GTP),nuclear	1.00 * GTP [n] + 1.00 * UMP [n] --> 1.00 * UDP [n] + 1.00 *	CMPK1	Nucleotide interconversion	22	2.7.4.14	4
UMPK4n_pos	7	0.382733	0.4171041	1.077892	1.077892	1	0.518179	UMPK4n	UMP kinase (GTP),nuclear	1.00 * GTP [n] + 1.00 * UMP [n] --> 1.00 * UDP [n] + 1.00 *	CMPK1	Nucleotide interconversion	22	2.7.4.14	4
UMPK5n_neg	7	0.382733	0.4171041	1.077892	1.077892	1	0.518179	UMPK5n	UMP kinase (dATP),nuclear	1.00 * dATP [n] + 1.00 * UMP [n] --> 1.00 * dADP [n] + 1.00	CMPK1	Nucleotide interconversion	22	2.7.4.14	4
UMPK5n_pos	7	0.382733	0.4171041	1.077892	1.077892	1	0.518179	UMPK5n	UMP kinase (dATP),nuclear	1.00 * dATP [n] + 1.00 * UMP [n] --> 1.00 * dADP [n] + 1.00	CMPK1	Nucleotide interconversion	22	2.7.4.14	4
UMPK6n_neg	7	0.382733	0.4171041	1.077892	1.077892	1	0.518179	UMPK6n	UMP kinase (dCTP),nuclear	1.00 * dCTP [n] + 1.00 * UMP [n] --> 1.00 * dCDP [n] + 1.00	CMPK1	Nucleotide interconversion	22	2.7.4.14	4
UMPK6n_pos	7	0.382733	0.4171041	1.077892	1.077892	1	0.518179	UMPK6n	UMP kinase (dCTP),nuclear	1.00 * dCTP [n] + 1.00 * UMP [n] --> 1.00 * dCDP [n] + 1.00	CMPK1	Nucleotide interconversion	22	2.7.4.14	4
UMPK7n_neg	7	0.382733	0.4171041	1.077892	1.077892	1	0.518179	UMPK7n	UMP kinase (dGTP),nuclear	1.00 * dGTP [n] + 1.00 * UMP [n] --> 1.00 * dGDP [n] + 1.0	CMPK1	Nucleotide interconversion	22	2.7.4.14	4
UMPK7n_pos	7	0.382733	0.4171041	1.077892	1.077892	1	0.518179	UMPK7n	UMP kinase (dGTP),nuclear	1.00 * dGTP [n] + 1.00 * UMP [n] --> 1.00 * dGDP [n] + 1.0	CMPK1	Nucleotide interconversion	22	2.7.4.14	4
UMPKn_neg	7	0.382733	0.4171041	1.077892	1.077892	1	0.518179	UMPKn	UMP kinase, nuclear	1.00 * ATP [n] + 1.00 * UMP [n] --> 1.00 * UDP [n] + 1.00 * A	CMPK1	Nucleotide interconversion	22	2.7.4.14	4
UMPKn_pos	7	0.382733	0.4171041	1.077892	1.077892	1	0.518179	UMPKn	UMP kinase, nuclear	1.00 * ATP [n] + 1.00 * UMP [n] --> 1.00 * UDP [n] + 1.00 * A	CMPK1	Nucleotide interconversion	22	2.7.4.14	4
ADK1_neg	6	0.662521	0.1788004	1.624234	1.624234	1	0.758498	ADK1	adenylate kinase	1.00 * ATP [c] + 1.00 * AMP [c] --> 2.00 * ADP [c]AK1; AK2;	AK1; AK2; A	Nucleotide interconversion	22		4
ADK1_pos	6	0.662521	0.1788004	1.624234	1.624234	1	0.758498	ADK1	adenylate kinase	1.00 * ATP [c] + 1.00 * AMP [c] --> 2.00 * ADP [c]AK1; AK2;	AK1; AK2; A	Nucleotide interconversion	22		4
ADK3_neg	6	0.662521	0.1788004	1.624234	1.624234	1	0.758498	ADK3	adentylate kinase (GTP)	1.00 * AMP [c] + 1.00 * GTP [c] --> 1.00 * ADP [c] + 1.00 * G	AK5	Nucleotide interconversion	22		4
ADK3_pos	6	0.662521	0.1788004	1.624234	1.624234	1	0.758498	ADK3	adentylate kinase (GTP)	1.00 * AMP [c] + 1.00 * GTP [c] --> 1.00 * ADP [c] + 1.00 * G	AK5	Nucleotide interconversion	22		4

ADKd_neg	6	0.662521	0.1788004	1.628283	1.628283	1	0.758498	ADKd	adenylate kinase (d form)	1.00 * dAMP(2-) [c] + 1.00 * dATP [c] --> 2.00 * dADP [c]AK	AK5	Nucleotide interconversion	22	2.7.4.3	4
ADKd_pos	6	0.662521	0.1788004	1.628283	1.628283	1	0.758498	ADKd	adenylate kinase (d form)	1.00 * dAMP(2-) [c] + 1.00 * dATP [c] --> 2.00 * dADP [c]AK	AK5	Nucleotide interconversion	22	2.7.4.3	4
NDPK10_neg	3	0.662521	0.1788004	0.123933	0.123933	1	0.758498	NDPK10	nucleoside-diphosphate kinase (ATP:dIDP)	1.00 * ATP [c] + 1.00 * dIDP [c] --> 1.00 * ADP [c] + 1.00 * d	GM20390;	Nucleotide interconversion	22	2.7.4.6	4
NDPK10_pos	3	0.662521	0.1788004	0.123933	0.123933	1	0.758498	NDPK10	nucleoside-diphosphate kinase (ATP:dIDP)	1.00 * ATP [c] + 1.00 * dIDP [c] --> 1.00 * ADP [c] + 1.00 * d	GM20390;	Nucleotide interconversion	22	2.7.4.6	4
NDPK10n_neg	3	0.662521	0.1788004	0.123933	0.123933	1	0.758498	NDPK10n	nucleoside-diphosphate kinase (ATP:dIDP), nuclear	1.00 * ATP [n] + 1.00 * dIDP [n] --> 1.00 * ADP [n] + 1.00 * d	GM20390;	Nucleotide interconversion	22	2.7.4.6	4
NDPK10n_pos	3	0.662521	0.1788004	0.123933	0.123933	1	0.758498	NDPK10n	nucleoside-diphosphate kinase (ATP:dIDP), nuclear	1.00 * ATP [n] + 1.00 * dIDP [n] --> 1.00 * ADP [n] + 1.00 * d	GM20390;	Nucleotide interconversion	22	2.7.4.6	4
NDPK9n_neg	3	0.662521	0.1788004	0.123933	0.123933	1	0.758498	NDPK9n	nucleoside-diphosphate kinase (ATP:IDP), nuclear	1.00 * ATP [n] + 1.00 * IDP(3-) [n] --> 1.00 * ADP [n] + 1.00	GM20390;	Nucleotide interconversion	22	2.7.4.6	4
NDPK9n_pos	3	0.662521	0.1788004	0.123933	0.123933	1	0.758498	NDPK9n	nucleoside-diphosphate kinase (ATP:IDP), nuclear	1.00 * ATP [n] + 1.00 * IDP(3-) [n] --> 1.00 * ADP [n] + 1.00	GM20390;	Nucleotide interconversion	22	2.7.4.6	4
r0345_neg	6	0.662521	0.1788004	1.628283	1.628283	1	0.758498	r0345	ATP:AMP phosphotransferase Purine metabolism EC:2.7.4.11	1.00 * ATP [c] + 1.00 * dAMP(2-) [c] --> 1.00 * ADP [c] + 1.0	AK1; AK2; A	Nucleotide interconversion	22	2.7.4.11	0
r0345_pos	6	0.662521	0.1788004	1.628283	1.628283	1	0.758498	r0345	ATP:AMP phosphotransferase Purine metabolism EC:2.7.4.11	1.00 * ATP [c] + 1.00 * dAMP(2-) [c] --> 1.00 * ADP [c] + 1.0	AK1; AK2; A	Nucleotide interconversion	22	2.7.4.11	0
NDPK1n_neg	4	1	0	0.341896	0.341896	1	1	NDPK1n	nucleoside-diphosphate kinase (ATP:GDP), nuclear	1.00 * ATP [n] + 1.00 * GDP [n] --> 1.00 * ADP [n] + 1.00 * G	GM20390;	Nucleotide interconversion	22	2.7.4.6	4
NDPK1n_pos	4	1	0	0.341896	0.341896	1	1	NDPK1n	nucleoside-diphosphate kinase (ATP:GDP), nuclear	1.00 * ATP [n] + 1.00 * GDP [n] --> 1.00 * ADP [n] + 1.00 * G	GM20390;	Nucleotide interconversion	22	2.7.4.6	4
NDPK2n_neg	4	1	0	0.341896	0.341896	1	1	NDPK2n	nucleoside-diphosphate kinase (ATP:UDP), nuclear	1.00 * ATP [n] + 1.00 * UDP [n] --> 1.00 * UTP [n] + 1.00 * A	GM20390;	Nucleotide interconversion	22	2.7.4.6	4
NDPK2n_pos	4	1	0	0.341896	0.341896	1	1	NDPK2n	nucleoside-diphosphate kinase (ATP:UDP), nuclear	1.00 * ATP [n] + 1.00 * UDP [n] --> 1.00 * UTP [n] + 1.00 * A	GM20390;	Nucleotide interconversion	22	2.7.4.6	4
NDPK3n_neg	4	1	0	0.341896	0.341896	1	1	NDPK3n	nucleoside-diphosphate kinase (ATP:CDP),nuclear	1.00 * ATP [n] + 1.00 * CDP [n] --> 1.00 * CTP [n] + 1.00 * A	GM20390;	Nucleotide interconversion	22	2.7.4.6	4
NDPK3n_pos	4	1	0	0.341896	0.341896	1	1	NDPK3n	nucleoside-diphosphate kinase (ATP:CDP),nuclear	1.00 * ATP [n] + 1.00 * CDP [n] --> 1.00 * CTP [n] + 1.00 * A	GM20390;	Nucleotide interconversion	22	2.7.4.6	4
NDPK4_pos	4	1	0	0.406305	0.406305	1	1	NDPK4	nucleoside-diphosphate kinase (ATP:dTDP)	1.00 * ATP [c] + 1.00 * dTDP [c] --> 1.00 * ADP [c] + 1.00 * d	GM20390;	Nucleotide interconversion	22		4
NDPK4n_neg	4	1	0	0.312921	0.312921	1	1	NDPK4n	nucleoside-diphosphate kinase (ATP:dTDP), nuclear	1.00 * ATP [n] + 1.00 * dTDP [n] --> 1.00 * ADP [n] + 1.00 *	GM20390;	Nucleotide interconversion	22	2.7.4.6	4
NDPK4n_pos	4	1	0	0.312921	0.312921	1	1	NDPK4n	nucleoside-diphosphate kinase (ATP:dTDP), nuclear	1.00 * ATP [n] + 1.00 * dTDP [n] --> 1.00 * ADP [n] + 1.00 *	GM20390;	Nucleotide interconversion	22	2.7.4.6	4
NDPK5n_neg	4	1	0	0.341896	0.341896	1	1	NDPK5n	nucleoside-diphosphate kinase (ATP:dGDP), nuclear	1.00 * ATP [n] + 1.00 * dGDP [n] --> 1.00 * dGTP [n] + 1.00	GM20390;	Nucleotide interconversion	22	2.7.4.6	4
NDPK5n_pos	4	1	0	0.341896	0.341896	1	1	NDPK5n	nucleoside-diphosphate kinase (ATP:dGDP), nuclear	1.00 * ATP [n] + 1.00 * dGDP [n] --> 1.00 * dGTP [n] + 1.00	GM20390;	Nucleotide interconversion	22	2.7.4.6	4
NDPK6_neg	4	1	0	0.306167	0.306167	1	1	NDPK6	Nucleoside-diphosphate kinase (ATP:dUDP)	1.00 * ATP [c] + 1.00 * dUDP [c] --> 1.00 * ADP [c] + 1.00 *	GM20390;	Nucleotide interconversion	22		4
NDPK7n_neg	4	1	0	0.341896	0.341896	1	1	NDPK7n	nucleoside-diphosphate kinase (ATP:dCDP), nuclear	1.00 * ATP [n] + 1.00 * dCDP [n] --> 1.00 * dCTP [n] + 1.00	GM20390;	Nucleotide interconversion	22	2.7.4.6	4
NDPK7n_pos	4	1	0	0.341896	0.341896	1	1	NDPK7n	nucleoside-diphosphate kinase (ATP:dCDP), nuclear	1.00 * ATP [n] + 1.00 * dCDP [n] --> 1.00 * dCTP [n] + 1.00	GM20390;	Nucleotide interconversion	22	2.7.4.6	4
NDPK8n_neg	4	1	0	0.341896	0.341896	1	1	NDPK8n	nucleoside-diphosphate kinase (ATP:dADP), nuclear	1.00 * ATP [n] + 1.00 * dADP [n] --> 1.00 * dATP [n] + 1.00	GM20390;	Nucleotide interconversion	22	2.7.4.6	4
NDPK8n_pos	4	1	0	0.341896	0.341896	1	1	NDPK8n	nucleoside-diphosphate kinase (ATP:dADP), nuclear	1.00 * ATP [n] + 1.00 * dADP [n] --> 1.00 * dATP [n] + 1.00	GM20390;	Nucleotide interconversion	22	2.7.4.6	4
NDPK9_neg	4	1	0	0.480189	0.480189	1	1	NDPK9	nucleoside-diphosphate kinase (ATP:IDP)	1.00 * ATP [c] + 1.00 * IDP(3-) [c] --> 1.00 * ADP [c] + 1.00 *	GM20390;	Nucleotide interconversion	22	2.7.4.6	4
NDPK9_pos	4	1	0	0.480189	0.480189	1	1	NDPK9	nucleoside-diphosphate kinase (ATP:IDP)	1.00 * ATP [c] + 1.00 * IDP(3-) [c] --> 1.00 * ADP [c] + 1.00 *	GM20390;	Nucleotide interconversion	22	2.7.4.6	4
r1156_neg	4	1	0	0.306167	0.306167	1	1	r1156	dUTP:uridine 5-phosphotransferase Pyrimidine metabolism EC:2.7.1	1.00 * Uridine [c] + 1.00 * dUTP [c] --> 1.00 * proton [c] + 1	UCK1; UCK	Nucleotide interconversion	22	2.7.1.48	0
XYLUR_neg	0	0.080856	1.0922877	-6.95104	6.951038	2	0.240222	XYLUR	xylulose reductase	1.00 * proton [c] + 1.00 * Nicotinamide adenine dinucleo	DCXR	Pentose phosphate pathway	23	1.1.1.10	4
XYLUR_pos	0	0.080856	1.0922877	-1.98662	1.986624	2	0.240222	XYLUR	xylulose reductase	1.00 * proton [c] + 1.00 * Nicotinamide adenine dinucleo	DCXR	Pentose phosphate pathway	23	1.1.1.10	4
r0784_neg	0	0.080856	1.0922877	-6.95104	6.951038	2	0.240222	r0784	xylitol:NAD oxidoreductase Pentose and glucuronate interconversio	1.00 * Nicotinamide adenine dinucleotide [c] + 1.00 * xyl	1.00 * Nico	Pentose phosphate pathway	23	1.1.1.15	0
r0784_pos	0	0.080856	1.0922877	-1.98662	1.986624	2	0.240222	r0784	xylitol:NAD oxidoreductase Pentose and glucuronate interconversio	1.00 * Nicotinamide adenine dinucleotide [c] + 1.00 * xyl	1.00 * Nico	Pentose phosphate pathway	23	1.1.1.15	0
RPI_neg	8	0.19043	0.7202646	1.414492	1.414492	1	0.347218	RPI	ribose-5-phosphate isomerase	1.00 * alpha-D-Ribose 5-phosphate [c] --> 1.00 * D-ribulos	RPIA	Pentose phosphate pathway	23	5.3.1.6	0
RPI_pos	8	0.19043	0.7202646	1.414492	1.414492	1	0.347218	RPI	ribose-5-phosphate isomerase	1.00 * alpha-D-Ribose 5-phosphate [c] --> 1.00 * D-ribulos	RPIA	Pentose phosphate pathway	23	5.3.1.6	0
TALA_pos	1	0.19043	0.7202646	-1.46042	1.460419	2	0.347218	TALA	transaldolase	1.00 * Glyceraldehyde 3-phosphate [c] + 1.00 * Sedohept	TALDO1	Pentose phosphate pathway	23	2.2.1.2	0
r0249_neg	8	0.19043	0.7202646	1.414492	1.414492	1	0.347218	r0249	D-Ribose-5-phosphate ketol-isomerase Pentose phosphate pathway	1.00 * alpha-D-Ribose 5-phosphate [r] --> 1.00 * D-ribulos	RPIA	Pentose phosphate pathway	23	5.3.1.6	0
r0249_pos	8	0.19043	0.7202646	1.414492	1.414492	1	0.347218	r0249	D-Ribose-5-phosphate ketol-isomerase Pentose phosphate pathway	1.00 * alpha-D-Ribose 5-phosphate [r] --> 1.00 * D-ribulos	RPIA	Pentose phosphate pathway	23	5.3.1.6	0
G6PDH1rer_neg	6	0.662521	0.1788004	0.722007	0.722007	1	0.758498	G6PDH1rer	glucose-6-phosphate dehydrogenase, endoplasmic reticulum	1.00 * Nicotinamide adenine dinucleotide [r] + 1.00 * D-G	H6PD	Pentose phosphate pathway	23	1.1.1.47	4
G6PDH1rer_pos	6	0.662521	0.1788004	0.722007	0.722007	1	0.758498	G6PDH1rer	glucose-6-phosphate dehydrogenase, endoplasmic reticulum	1.00 * Nicotinamide adenine dinucleotide [r] + 1.00 * D-G	H6PD	Pentose phosphate pathway	23	1.1.1.47	4
G6PDH2rer_neg	6	0.662521	0.1788004	0.722007	0.722007	1	0.758498	G6PDH2rer	glucose 6-phosphate dehydrogenase, endoplasmic reticulum	1.00 * Nicotinamide adenine dinucleotide phosphate [r]	G6PD2; H6	Pentose phosphate pathway	23	1.1.1.47	4
G6PDH2rer_pos	6	0.662521	0.1788004	0.722007	0.722007	1	0.758498	G6PDH2rer	glucose 6-phosphate dehydrogenase, endoplasmic reticulum	1.00 * Nicotinamide adenine dinucleotide phosphate [r]	G6PD2; H6	Pentose phosphate pathway	23	1.1.1.47	4
PHETHPTOX2_pos	2	0.382733	0.4171041	-0.86474	0.864735	2	0.518179	PHETHPTOX	L-Phenylalanine,tetrahydrobiopterin:oxygen oxidoreductase (4-hyd	1.00 * O2 [c] + 1.00 * 5,6,7,8-tetrahydrobiopterin [c] + 1.0	PAH	Phenylalanine metabolism	24	1.14.16.1	4
r0545_pos	6	0.662521	0.1788004	1.000342	1.000342	1	0.758498	r0545	Phenylacetaldehyde:NAD+ oxidoreductase Phenylalanine metabolis	1.00 * Water [c] + 1.00 * Nicotinamide adenine dinucleot	ALDH1A3; A	Phenylalanine metabolism	24	1.2.1.5	0
r0547_neg	6	0.662521	0.1788004	1.000342	1.000342	1	0.758498	r0547	Aldehyde:NADP+ oxidoreductase Phenylalanine metabolism EC:1.2.	1.00 * Water [c] + 1.00 * Nicotinamide adenine dinucleot	ALDH1A3; A	Phenylalanine metabolism	24	1.2.1.5	0
GUAD_pos	0	0.080856	1.0922877	-5.40841	5.408411	2	0.240222	GUAD	guanine deaminase	1.00 * Water [c] + 1.00 * proton [c] + 1.00 * Guanine [c] -->	GDA	Purine catabolism	25	3.5.4.3	4
PUNP3_pos	0	0.080856	1.0922877	-5.40841	5.408411	2	0.240222	PUNP3	purine-nucleoside phosphorylase (Guanosine)	1.00 * hydrogenphosphate [c] + 1.00 * Guanosine [c] --> 1	PNP2	Purine catabolism	25	3.4.2.1	4
r0809_neg	2	0.382733	0.4171041	-1.29337	1.293368	2	0.518179	r0809	Facilitated diffusion	1.00 * Adenosine [c] + 1.00 * Inosine [e] --> 1.00 * Inosine	1.00 * Aden	Purine catabolism	25		0
r0809_pos	2	0.382733	0.4171041	-1.29337	1.293368	2	0.518179	r0809	Facilitated diffusion	1.00 * Adenosine [c] + 1.00 * Inosine [e] --> 1.00 * Inosine	1.00 * Aden	Purine catabolism	25		0
DURAD2_pos	0	0.080856	1.0922877	-4.19166	4.191658	2	0.240222	DURAD2	dihydrothymin dehydrogenase (NADP)	1.00 * Nicotinamide adenine dinucleotide phosphate [c]	DPYD	Pyrimidine catabolism	26	1.3.1.2	4
r0330_pos	0	0.080856	1.0922877	-4.19166	4.191658	2	0.240222	r0330	5,6-Dihydrothymine:NAD+ oxidoreductase Pyrimidine metabolism	1.00 * proton [c] + 1.00 * Nicotinamide adenine dinucleo	1.00 * prot	Pyrimidine catabolism	26	1.3.1.1	0
DURAD_neg	1	0.19043	0.7202646	-1.15486	1.154858	2	0.347218	DURAD	dihydrouracil dehydrogenase (NADP)	1.00 * Nicotinamide adenine dinucleotide phosphate [c]	DPYD	Pyrimidine catabolism	26	1.3.1.2	4
r0236_pos	1	0.19043	0.7202646	-1.15486	1.154858	2	0.347218	r0236	5,6-Dihydrouracil:NAD+ oxidoreductase Pyrimidine metabolism EC:	1.00 * Nicotinamide adenine dinucleotide [c] + 1.00 * 5,6	1.00 * Nico	Pyrimidine catabolism	26	1.3.1.1	0
D3AIBTm_neg	6	0.662521	0.1788004	-0.32478	0.324776	2	0.758498	D3AIBTm	D-3-Amino-isobutanoate:pyruvate aminotransferase, mitochondrial	1.00 * pyruvate [m] + 1.00 * D-3-Amino-isobutanoate [m]	AGXT2	Pyrimidine catabolism	26	2.6.1.40	4
D3AIBTm_pos	6	0.662521	0.1788004	-0.32478	0.324776	2	0.758498	D3AIBTm	D-3-Amino-isobutanoate:pyruvate aminotransferase, mitochondrial	1.00 * pyruvate [m] + 1.00 * D-3-Amino-isobutanoate [m]	AGXT2	Pyrimidine catabolism	26	2.6.1.40	4
r0483_neg	6	0.662521	0.1788004	-0.32478	0.324776	2	0.758498	r0483	(R)-3-Amino-2-methylpropanoate:2-oxoglutarate aminotransferase	1.00 * 2-Oxoglutarate [m] + 1.00 * D-3-Amino-isobutanoa	ABAT	Pyrimidine catabolism	26	2.6.1.22	0
r0483_pos	6	0.662521	0.1788004	-0.32478	0.324776	2	0.758498	r0483	(R)-3-Amino-2-methylpropanoate:2-oxoglutarate aminotransferase	1.00 * 2-Oxoglutarate [m] + 1.00 * D-3-Amino-isobutanoa	ABAT	Pyrimidine catabolism	26	2.6.1.22	0
RE1530C_neg	9	0.080856	1.0922877	4.748812	4.748812	1	0.240222	RE1530C	RE1530	1.00 * dGTP [c] + 1.00 * Deoxyuridine [c] --> 1.00 * proton [c] + 1.00 * dGDP [c] + 1.00 * dUMP [c] No genes	1.00 * dGT	Pyrimidine synthesis	27	2.7.1.21	0
RE0453C_neg	7	0.382733	0.4171041	1.039213	1.039213	1	0.518179	RE0453C	RE0453	1.00 * dATP [c] + 1.00 * dTDP [c] --> 1.00 * dADP [c] + 1.00	1.00 * dATP	Pyrimidine synthesis	27	2.7.4.6	0
RE0453C_pos	7	0.382733	0.4171041	1.039213	1.039213	1	0.518179	RE0453C	RE0453	1.00 * dATP [c] + 1.00 * dTDP [c] --> 1.00 * dADP [c] + 1.00	1.00 * dATP	Pyrimidine synthesis	27	2.7.4.6	0
URIK1_pos	4	1	0	0.306167	0.306167	1	1	URIK1	uridine kinase (ATP:Uridine)	1.00 * ATP [c] + 1.00 * Uridine [c] --> 1.00 * ADP [c] + 1.00	UCK1; UCK	Pyrimidine synthesis	27	2.7.1.48	4
RE0453N_neg	4	1	0	0.312921	0.312921	1	1	RE0453N	RE0453	1.00 * dATP [n] + 1.00 * dTDP [n] --> 1.00 * dADP [n] + 1.00	GM20390;	Pyrimidine synthesis	27	2.7.4.6	0
RE0453N_pos	4	1	0	0.312921	0.312921	1	1	RE0453N	RE0453	1.00 * dATP [n] + 1.00 * dTDP [n] --> 1.00 * dADP [n] + 1.00	GM20390;	Pyrimidine synthesis	27	2.7.4.6	0
RE2954C_neg	4	1	0	0.406305	0.406305	1	1	RE2954C	RE2954	1.00 * proton [c] + 1.00 * Phosphoenolpyruvate [c] + 1.00	PKM	Pyrimidine synthesis	27	2.7.1.40	0
ALR2_pos	1	0.19043	0.7202646	-1.40736	1.407358	2	0.347218	ALR2	aldose reductase (methylglyoxal)	1.00 * proton [c] + 1.00 * Nicotinamide adenine dinucleo	AKR1A1; AK	Pyruvate metabolism	28	1.1.1.21	4
ALR3_pos	1	0.19043	0.7202646	-1.40736	1.407358	2	0.347218	ALR3	aldose reductase (acetol)	1.00 * proton [c] + 1.00 * Nicotinamide adenine dinucleo	AKR1A1; AK	Pyruvate metabolism	28	1.1.1.21	4
LALDO_neg	1	0.19043	0.7202646	-1.09901	1.099014	2	0.347218	LALDO	D-Lactaldehyde:NAD+ oxidoreductase (glutathione-formylating)	1.00 * Nicotinamide adenine dinucleotide [c] + 1.00 * (R)-	ADH5	Pyruvate metabolism	28	1.1.1.1	0
GLYOX_pos	2	0.382733	0.4171041	-0.97891	0.978909	2	0.518179	GLYOX	hydroxyacylglutathione hydrolase	1.00 * Water [c] + 1.00 * (R)-S-lactoylglutathionate(1-) [c]	HAGH; HAG	Pyruvate metabolism	28	3.1.2.6	4
LGTHL_pos	2	0.382733	0.4171041	-0.97891	0.978909	2	0.518179	LGTHL	lactoylglutathione lyase	1.00 * methylglyoxal [c] + 1.00 * Reduced glutathione [c]	GLO1	Pyruvate metabolism	28	4.4.1.5	4
r0055_neg	2	0.382733	0.4171041	-0.97891	0.978909	2	0.518179	r0055	2-Oxopropanal:NADP+ oxidoreductase Pyruvate metabolism EC:1.2	1.00 * Water [c] + 1.00 * Nicotinamide adenine dinucleot	1.00 * Wat	Pyruvate metabolism	28	1.2.1.49	0
LALDO2x_pos	6	0.662521	0.1788004	0.909997	0.909997	1	0.758498	LALDO2x	D-Lactaldehyde:NAD+ 1-oxidoreductase	1.00 * proton [c] + 1.00 * Nicotinamide adenine dinucleo	AKR7A5	Pyruvate metabolism	28	1.1.1.21	4
LALDO_pos	6	0.662521	0.1788004	0.869126	0.869126	1	0.758498	LALDO	D-Lactaldehyde:NAD+ oxidoreductase (glutathione-formylating)	1.00 * Nicotinamide adenine dinucleotide [c] + 1.00 * (R)-	ADH5	Pyruvate metabolism	28	1.1.1.1	0
HSD17B1_neg	1	0.19043	0.7202646	-1.59512	1.595115	2	0.347218	HSD17B1	testicular 17-beta-hydroxysteroid dehydrogenase	1.00 * proton [c] + 1.00 * Nicotinamide adenine dinucleo	H2-KE6; HS	Steroid metabolism	29	1.1.1.62	4
HSD17B7r_pos	1	0.19043	0.7202646	-1.59512	1.595115	2	0.347218	HSD17B7r	testicular 17-beta-hydroxysteroid dehydrogenase	1.00 * proton [r] + 1.00 * Nicotinamide adenine dinucleo	HSD17B7	Steroid metabolism	29	1.1.1.62	4
HSD17B8r_pos	1	0.19043	0.7202646	-2.02049	2.020486	2	0.347218	HSD17B8r	17-beta-hydroxysteroid dehydrogenase	1.00 * proton [r] + 1.00 * Nicotinamide adenine dinucleo	HSD17B2	Steroid metabolism	29	1.1.1.62	4
HSD3A1r_neg	6	0.662521	0.1788004	0.722007	0.722007	1	0.758498	HSD3A1r	3 alpha-hydroxysteroid dehydrogenase (type 3)	1.00 * proton [r] + 1.00 * Nicotinamide adenine dinucleo	AKR1C6	Steroid metabolism	29	1.1.1.225	4
HSD3A1r_pos	6	0.662521	0.1788004	0.722007	0.722007	1	0.758498	HSD3A1r	3 alpha-hydroxysteroid dehydrogenase (type 3)	1.00 * proton [r] + 1.00 * Nicotinamide adenine dinucleo	AKR1C6	Steroid metabolism	29	1.1.1.225	4
HSD3A2r_neg	6	0.662521	0.1788004	0.722007	0.722007	1	0.758498	HSD3A2r	3 alpha-hydroxysteroid dehydrogenase (type 3)	1.00 * proton [r] + 1.00 * Nicotinamide adenine dinucleo	AKR1C6	Steroid metabolism	29	1.1.1.225	0
HSD3A2r_pos	6	0.662521	0.1788004	0.722007	0.722007	1	0.758498	HSD3A2r	3 alpha-hydroxysteroid dehydrogenase (type 3)	1.00 * proton [r] + 1.00 * Nicotinamide adenine dinucleo	AKR1C6	Steroid metabolism	29	1.1.1.225	0
HSD11B1r_neg	4	1	0	-0.0199	0.019897	2	1	HSD11B1r	11-beta-hydroxysteroid dehydrogenase type 1	1.00 * proton [r] + 1.00 * Nicotinamide adenine dinucleo	HSD11B1	Steroid metabolism	29	1.1.1.146	4
HSD11B1r_pos	4	1	0	-0.0199	0.019897	2	1	HSD11B1r	11-beta-hydroxysteroid dehydrogenase type 1	1.00 * proton [r] + 1.00 * Nicotinamide adenine dinucleo	HSD11B1	Steroid metabolism	29	1.1.1.146	4

HSD11B2r_neg	4	1	0	-0.0199	0.019897	2	1	HSD11B2r	11-beta-hydroxysteroid dehydrogenase type 2	1.00 * proton [r] + 1.00 * Nicotinamide adenine dinucleo	HSD11B2	Steroid metabolism	29		4
HSD11B2r_pos	4	1	0	-0.0199	0.019897	2	1	HSD11B2r	11-beta-hydroxysteroid dehydrogenase type 2	1.00 * proton [r] + 1.00 * Nicotinamide adenine dinucleo	HSD11B2	Steroid metabolism	29		4
THBPT4ACAMDASE_	2	0.382733	0.4171041	-0.86474	0.864735	2	0.518179	THBPT4ACA	Tetrahydrobiopterin-4a-carbinolamine dehydratase	1.00 * 4a-hydroxytetrahydrobiopterin [c] --> 1.00 * Wate	PCBD1	Tetrahydrobiopterin metabolism	30	4.2.1.96	4
r0399_neg	2	0.382733	0.4171041	-0.86474	0.864735	2	0.518179	r0399	L-Phenylalanine,tetrahydrobiopterin:oxygen oxidoreductase (4-hyd	1.00 * O2 [c] + 1.00 * 5,6,7,8-tetrahydrobiopterin [c] + 1.	PAH	Tetrahydrobiopterin metabolism	30	1.14.16.1	0
GTPCI_pos	6	0.662521	0.1788004	0.206319	0.206319	1	0.758498	GTPCI	GTP cyclohydrolase I	1.00 * Water [c] + 1.00 * GTP [c] --> 1.00 * proton [c] + 1.0	GCH1	Tetrahydrobiopterin metabolism	30	3.5.4.16	4
GTPCIn_pos	6	0.662521	0.1788004	0.206319	0.206319	1	0.758498	GTPCIn	GTP cyclohydrolase I, nuclear	1.00 * Water [n] + 1.00 * GTP [n] --> 1.00 * proton [n] + 1.0	GCH1	Tetrahydrobiopterin metabolism	30	3.5.4.16	4
RE0830N_neg	3	0.662521	0.1788004	-0.69243	0.692431	2	0.758498	RE0830N	RE0830	1.00 * 4a-hydroxytetrahydrobiopterin [n] --> 1.00 * Wate	PCBD1; PC	Tetrahydrobiopterin metabolism	30	4.2.1.96	0
RE0830N_pos	3	0.662521	0.1788004	-0.69243	0.692431	2	0.758498	RE0830N	RE0830	1.00 * 4a-hydroxytetrahydrobiopterin [n] --> 1.00 * Wate	PCBD1; PC	Tetrahydrobiopterin metabolism	30	4.2.1.96	0
RE1709N_neg	3	0.662521	0.1788004	-0.69243	0.692431	2	0.758498	RE1709N	RE1709	1.00 * 4a-hydroxytetrahydrobiopterin [n] --> 1.00 * Wate	PCBD1; PC	Tetrahydrobiopterin metabolism	30	4.2.1.96	0
RE1709N_pos	3	0.662521	0.1788004	-0.69243	0.692431	2	0.758498	RE1709N	RE1709	1.00 * 4a-hydroxytetrahydrobiopterin [n] --> 1.00 * Wate	PCBD1; PC	Tetrahydrobiopterin metabolism	30	4.2.1.96	0
RE2660N_neg	3	0.662521	0.1788004	-0.69243	0.692431	2	0.758498	RE2660N	RE2660	1.00 * O2'-4a-cyclic-tetrahydrobiopterin [n] --> 1.00 * qu	PCBD1; PC	Tetrahydrobiopterin metabolism	30	4.2.1.96	0
RE2660N_pos	3	0.662521	0.1788004	-0.69243	0.692431	2	0.758498	RE2660N	RE2660	1.00 * O2'-4a-cyclic-tetrahydrobiopterin [n] --> 1.00 * qu	PCBD1; PC	Tetrahydrobiopterin metabolism	30	4.2.1.96	0
r0120_neg	6	0.662521	0.1788004	0.206319	0.206319	1	0.758498	r0120	GTP 7,8-8,9-dihydrolase Folate biosynthesis EC:3.5.4.16	1.00 * Water [c] + 4.00 * proton [c] + 1.00 * GTP [c] --> 1.0	GCH1	Tetrahydrobiopterin metabolism	30	3.5.4.16	0
r0121_neg	6	0.662521	0.1788004	0.206319	0.206319	1	0.758498	r0121	r0121	1.00 * Water [n] + 4.00 * proton [n] + 1.00 * GTP [n] --> 1.0	GCH1	Tetrahydrobiopterin metabolism	30	3.5.4.16	0
r0403_neg	6	0.662521	0.1788004	0.007222	0.007222	1	0.758498	r0403	L-Tryptophan,tetrahydrobiopterin:oxygen oxidoreductase (5-hydro	1.00 * O2 [c] + 1.00 * 5,6,7,8-tetrahydrobiopterin [c] + 1.	TPH1; TPH2	Tetrahydrobiopterin metabolism	30	1.14.16.4	0
r0708_pos	6	0.662521	0.1788004	0.206319	0.206319	1	0.758498	r0708	2-Amino-4-hydroxy-6-(erythro-1,2,3-trihydroxypropyl) dihydropter	1.00 * Water [c] + 4.00 * proton [c] + 1.00 * 7,8-dihydron	GCH1	Tetrahydrobiopterin metabolism	30	3.5.4.16	0
r0709_pos	6	0.662521	0.1788004	0.206319	0.206319	1	0.758498	r0709	r0709	1.00 * Water [n] + 4.00 * proton [n] + 1.00 * 7,8-dihydron	ECHS1; EHH	Tetrahydrobiopterin metabolism	30	3.5.4.16	0
r0776_neg	6	0.662521	0.1788004	0.206319	0.206319	1	0.758498	r0776	r0776	1.00 * Water [n] + 1.00 * Formamidopyrimidine nucleosi	ECHS1; EHH	Tetrahydrobiopterin metabolism	30	3.5.4.16	0
r0777_neg	6	0.662521	0.1788004	0.206319	0.206319	1	0.758498	r0777	GTP 7,8-8,9-dihydrolase Folate biosynthesis EC:3.5.4.16	4.00 * proton [c] + 1.00 * 2,5-Diaminopyrimidine nucleos	GCH1	Tetrahydrobiopterin metabolism	30	3.5.4.16	0
r0778_neg	6	0.662521	0.1788004	0.206319	0.206319	1	0.758498	r0778	r0778	4.00 * proton [n] + 1.00 * 2,5-Diaminopyrimidine nucleo	EHHADH; H	Tetrahydrobiopterin metabolism	30	3.5.4.16	0
ACCOAtr_neg	9	0.080856	1.0922877	1.613406	1.613406	1	0.240222	ACCOAtr	acetyl-coa transport	1.00 * Acetyl-CoA [c] --> 1.00 * Acetyl-CoA [r]SLC33A1	SLC33A1	Transport, endoplasmic reticular			0
ACCOAtr_pos	9	0.080856	1.0922877	1.613406	1.613406	1	0.240222	ACCOAtr	acetyl-coa transport	1.00 * Acetyl-CoA [c] --> 1.00 * Acetyl-CoA [r]SLC33A1	SLC33A1	Transport, endoplasmic reticular			0
r0995_neg	9	0.080856	1.0922877	1.613406	1.613406	1	0.240222	r0995	Mitochondrial Carrier (MC) TCDB:2.A.29.8.3	1.00 * L-Carnitine [c] + 1.00 * O-acetylcarnitine [r] --> 1.00	SLC25A20	Transport, endoplasmic reticular			0
r0995_pos	9	0.080856	1.0922877	1.613406	1.613406	1	0.240222	r0995	Mitochondrial Carrier (MC) TCDB:2.A.29.8.3	1.00 * L-Carnitine [c] + 1.00 * O-acetylcarnitine [r] --> 1.00	SLC25A20	Transport, endoplasmic reticular			0
r0840_neg	8	0.19043	0.7202646	1.414492	1.414492	1	0.347218	r0840	Free diffusion	1.00 * alpha-D-Ribose 5-phosphate [c] --> 1.00 * alpha-D-	1.00 * alph	Transport, endoplasmic reticular			0
r0840_pos	8	0.19043	0.7202646	1.414492	1.414492	1	0.347218	r0840	Free diffusion	1.00 * alpha-D-Ribose 5-phosphate [c] --> 1.00 * alpha-D-	1.00 * alph	Transport, endoplasmic reticular			0
r0841_neg	8	0.19043	0.7202646	1.414492	1.414492	1	0.347218	r0841	Free diffusion	1.00 * D-ribulose 5-phosphate(2-) [c] --> 1.00 * D-ribulose	1.00 * D-rib	Transport, endoplasmic reticular			0
r0841_pos	8	0.19043	0.7202646	1.414492	1.414492	1	0.347218	r0841	Free diffusion	1.00 * D-ribulose 5-phosphate(2-) [c] --> 1.00 * D-ribulose	1.00 * D-rib	Transport, endoplasmic reticular			0
G6Pter_neg	2	0.382733	0.4171041	-1.13714	1.137136	2	0.518179	G6Pter	glucose 6-phosphate endoplasmic reticular transport via diffusion	1.00 * D-Glucose 6-phosphate [c] --> 1.00 * D-Glucose 6-p	SLC37A4	Transport, endoplasmic reticular			4
HPETFABP1tc_pos	2	0.382733	0.4171041	-1.2911	1.291104	2	0.518179	HPETFABP1	transport of 5-HPETE into the enterocytes	2.00 * 5(S)-HPETE(1-) [c] --> 2.00 * 5(S)-HPETE(1-) [r]FABP1	FABP1	Transport, endoplasmic reticular			4
OCDEAFABP1tc_neg	2	0.382733	0.4171041	-1.2911	1.291104	2	0.518179	OCDEAFABI	Transport of octadecenoyl coA into the enterocytes	2.00 * octadecenoate (n-C18:1) [c] --> 2.00 * octadecenoa	FABP1	Transport, endoplasmic reticular			4
OCDEAFABP1tc_pos	2	0.382733	0.4171041	-1.2911	1.291104	2	0.518179	OCDEAFABI	Transport of octadecenoyl coA into the enterocytes	2.00 * octadecenoate (n-C18:1) [c] --> 2.00 * octadecenoa	FABP1	Transport, endoplasmic reticular			4
PMTCOAFABP1tc_ne	7	0.382733	0.4171041	1.115609	1.115609	1	0.518179	PMTCOAFA	transport of palmitoyl coA into the enterocytes	2.00 * Palmitoyl-CoA (n-C16:0CoA) [c] --> 2.00 * Palmitoy	FABP1	Transport, endoplasmic reticular			4
r0002_neg	2	0.382733	0.4171041	-1.2911	1.291104	2	0.518179	r0002	Active transport	1.00 * L-Carnitine [c] --> 1.00 * L-Carnitine [r]No genes	1.00 * L-Ca	Transport, endoplasmic reticular			0
r0002_pos	2	0.382733	0.4171041	-1.2911	1.291104	2	0.518179	r0002	Active transport	1.00 * L-Carnitine [c] --> 1.00 * L-Carnitine [r]No genes	1.00 * L-Ca	Transport, endoplasmic reticular			0
r0998_neg	7	0.382733	0.4171041	1.115609	1.115609	1	0.518179	r0998	Facilitated diffusion	1.00 * L-Carnitine [c] + 1.00 * L-palmitoylcarnitine [r] --> 1	1.00 * L-Ca	Transport, endoplasmic reticular			0
r1051_neg	7	0.382733	0.4171041	1.272298	1.272298	1	0.518179	r1051	Vesicular transport	1.00 * cholesterol [l] --> 1.00 * cholesterol [r]No genes	1.00 * chol	Transport, endoplasmic reticular			0
r1051_pos	7	0.382733	0.4171041	1.272298	1.272298	1	0.518179	r1051	Vesicular transport	1.00 * cholesterol [l] --> 1.00 * cholesterol [r]No genes	1.00 * chol	Transport, endoplasmic reticular			0
r1318_neg	7	0.382733	0.4171041	0.938989	0.938989	1	0.518179	r1318	Transport reaction	1.00 * linoleic coenzyme A [c] --> 1.00 * linoleic coenzyme	1.00 * linol	Transport, endoplasmic reticular			0
r2473_pos	2	0.382733	0.4171041	-1.13714	1.137136	2	0.518179	r2473	Major Facilitator(MFS) TCDB:2.A.1.4.5	1.00 * hydrogenphosphate [r] + 1.00 * D-Glucose 6-phosp	SLC37A4	Transport, endoplasmic reticular			0
r2509_neg	2	0.382733	0.4171041	-1.2911	1.291104	2	0.518179	r2509	Utilized transport	1.00 * L-Carnitine [r] + 1.00 * octadecenoate (n-C18:1) [c]	1.00 * L-Ca	Transport, endoplasmic reticular			0
r2509_pos	2	0.382733	0.4171041	-1.2911	1.291104	2	0.518179	r2509	Utilized transport	1.00 * L-Carnitine [r] + 1.00 * octadecenoate (n-C18:1) [c]	1.00 * L-Ca	Transport, endoplasmic reticular			0
r2511_pos	7	0.382733	0.4171041	0.938989	0.938989	1	0.518179	r2511	Utilized transport	1.00 * L-Carnitine [r] + 1.00 * linoleate [c] --> 1.00 * L-Carn	1.00 * L-Ca	Transport, endoplasmic reticular			0
r2521_neg	2	0.382733	0.4171041	-1.14825	1.148251	2	0.518179	r2521	Utilized transport	1.00 * Diphosphate [c] + 1.00 * hydrogenphosphate [r] -->	1.00 * Diph	Transport, endoplasmic reticular			0
r2521_pos	2	0.382733	0.4171041	-1.14825	1.148251	2	0.518179	r2521	Utilized transport	1.00 * Diphosphate [c] + 1.00 * hydrogenphosphate [r] -->	1.00 * Diph	Transport, endoplasmic reticular			0
r2537_neg	7	0.382733	0.4171041	0.957852	0.957852	1	0.518179	r2537	Utilized transport	1.00 * gamma-linolenoyl-CoA(4-) [c] --> 1.00 * gamma-lin	1.00 * gam	Transport, endoplasmic reticular			0
r0960_neg	3	0.662521	0.1788004	-0.39029	0.390288	2	0.758498	r0960	Resistance-Nodulation-Cell Division (RND) TCDB:2.A.65.1.1	1.00 * Sodium [c] + 1.00 * bilirubin(2-) [c] --> 1.00 * biliru	CP	Transport, endoplasmic reticular			0
r0960_pos	3	0.662521	0.1788004	-0.39029	0.390288	2	0.758498	r0960	Resistance-Nodulation-Cell Division (RND) TCDB:2.A.65.1.1	1.00 * Sodium [c] + 1.00 * bilirubin(2-) [c] --> 1.00 * biliru	CP	Transport, endoplasmic reticular			0
r1000_neg	6	0.662521	0.1788004	0.899335	0.899335	1	0.758498	r1000	Facilitated diffusion	1.00 * L-Carnitine [c] + 1.00 * palmitoleoyl-Carnitine [r] --	1.00 * L-Ca	Transport, endoplasmic reticular			0
r1010_neg	3	0.662521	0.1788004	-0.27302	0.273018	2	0.758498	r1010	Postulated transport reaction	1.00 * N-[(R)-pantothenoyl]-L-cysteinate [c] --> 1.00 * N-[(	1.00 * N-[(R	Transport, endoplasmic reticular			0
r2506_neg	3	0.662521	0.1788004	-0.39029	0.390288	2	0.758498	r2506	Utilized transport	1.00 * hydrogenphosphate [c] + 1.00 * Sodium [c] --> 1.00	1.00 * hydr	Transport, endoplasmic reticular			0
r2506_pos	3	0.662521	0.1788004	-0.39029	0.390288	2	0.758498	r2506	Utilized transport	1.00 * hydrogenphosphate [c] + 1.00 * Sodium [c] --> 1.00	1.00 * hydr	Transport, endoplasmic reticular			0
UDPGLCAter_neg	5	1	0	0.130644	0.130644	1	1	UDPGLCAte	UDPGlcA endoplasmic reticulum transport via UMP antiport	1.00 * UMP [c] + 1.00 * UDP-D-glucuronate [r] --> 1.00 * U	SLC35D1	Transport, endoplasmic reticular			4
UDPGLCAter_pos	5	1	0	0.130644	0.130644	1	1	UDPGLCAte	UDPGlcA endoplasmic reticulum transport via UMP antiport	1.00 * UMP [c] + 1.00 * UDP-D-glucuronate [r] --> 1.00 * U	SLC35D1	Transport, endoplasmic reticular			4
r2519_neg	5	1	0	0.130644	0.130644	1	1	r2519	Utilized transport	1.00 * UDP-D-glucuronate [c] --> 1.00 * UDP-D-glucurona	1.00 * UDP	Transport, endoplasmic reticular			0
r2519_pos	5	1	0	0.130644	0.130644	1	1	r2519	Utilized transport	1.00 * UDP-D-glucuronate [c] --> 1.00 * UDP-D-glucurona	1.00 * UDP	Transport, endoplasmic reticular			0
5MTHFt2_pos	0	0.080856	1.0922877	-1.93455	1.934552	2	0.240222	5MTHFt2	5-methyltetrahydrofolate transport via anion exchange	1.00 * Water [c] + 1.00 * 5-Methyltetrahydrofolate [e] -->	SLC19A1	Transport, extracellular			4
ABUTt4_2_r_neg	0	0.080856	1.0922877	-1.62626	1.626257	2	0.240222	ABUTt4_2_	4-aminobutyrate reversible transport in via sodium symport (1:2)	2.00 * Sodium [e] + 1.00 * 4-Aminobutanoate [e] --> 2.00	SLC6A1; SL	Transport, extracellular			4
ALACYSNaEx_pos	9	0.080856	1.0922877	3.071466	3.071466	1	0.240222	ALACYSNaE	L-alanine/L-cysteine Na-dependent exchange (Ala-L in)	1.00 * Sodium [e] + 1.00 * L-alanine [e] + 1.00 * L-cysteine	SLC1A4; SL	Transport, extracellular			4
BTNt2_neg	0	0.080856	1.0922877	-2.15646	2.156456	2	0.240222	BTNt2	Biotin reversible transport via proton symport	1.00 * proton [e] + 1.00 * Biotin [e] --> 1.00 * proton [c] +	SLC16A1	Transport, extracellular			4
BTNt2_pos	0	0.080856	1.0922877	-2.07298	2.072982	2	0.240222	BTNt2	Biotin reversible transport via proton symport	1.00 * proton [e] + 1.00 * Biotin [e] --> 1.00 * proton [c] +	SLC16A1	Transport, extracellular			4
BTNt4i_pos	0	0.080856	1.0922877	-2.15646	2.156456	2	0.240222	BTNt4i	Biotin uptake (antiport)	1.00 * proton [c] + 1.00 * Biotin [e] --> 1.00 * proton [e] +	SLC19A3	Transport, extracellular			4
CYSSERNaEx_pos	9	0.080856	1.0922877	2.266923	2.266923	1	0.240222	CYSSERNaE	L-serine/L-cysteine Na-dependent exchange (Cys-L in)	1.00 * Sodium [e] + 1.00 * L-cysteine [e] + 1.00 * L-serine [	SLC1A4; SL	Transport, extracellular			4
CYSTHRNaEx_pos	9	0.080856	1.0922877	2.266923	2.266923	1	0.240222	CYSTHRNaE	L-cysteine/L-threonine Na-dependent exchange (Cys-L in)	1.00 * Sodium [e] + 1.00 * L-cysteine [e] + 1.00 * L-threoni	SLC1A4; SL	Transport, extracellular			4
ESTRONESt2_pos	0	0.080856	1.0922877	-1.52674	1.526737	2	0.240222	ESTRONESt	estrone-3-sulfate transport via sodium cotransport	2.00 * Sodium [e] + 1.00 * estrone 3-sulfate(1-) [e] --> 2.00	SLC10A1	Transport, extracellular			4
FOLt2_neg	0	0.080856	1.0922877	-2.04859	2.048588	2	0.240222	FOLt2	folate transport via anion exchange	1.00 * Water [c] + 1.00 * Folate [e] --> 1.00 * Water [e] + 1.	SLC19A1	Transport, extracellular			4
FOLt2_pos	0	0.080856	1.0922877	-2.13615	2.136146	2	0.240222	FOLt2	folate transport via anion exchange	1.00 * Water [c] + 1.00 * Folate [e] --> 1.00 * Water [e] + 1.	SLC19A1	Transport, extracellular			4
GALt2_2_pos	0	0.080856	1.0922877	-1.86967	1.869669	2	0.240222	GALt2_2	D-galactose transportvia proton symport	2.00 * proton [e] + 1.00 * D-Galactose [e] --> 2.00 * protor	SLC5A1	Transport, extracellular			4
GCHOLAt2_pos	0	0.080856	1.0922877	-1.71311	1.71311	2	0.240222	GCHOLAt2	glycocholate transport via sodium cotransport	2.00 * Sodium [e] + 1.00 * glycocholate [e] --> 2.00 * Sodiu	SLC10A1; S	Transport, extracellular			4
GLCt2_2_pos	0	0.080856	1.0922877	-1.90551	1.905507	2	0.240222	GLCt2_2	D-glucose transport in via proton symport	2.00 * proton [e] + 1.00 * D-glucose [e] --> 2.00 * proton [	SLC5A1	Transport, extracellular			4
GLYBt4_2_r_neg	0	0.080856	1.0922877	-1.93799	1.937994	2	0.240222	GLYBt4_2_	Betaine transport (sodium symport) (2:1)	2.00 * Sodium [e] + 1.00 * glycine betaine [e] --> 2.00 * So	SLC6A12	Transport, extracellular			4
GLYBt4_2_r_pos	0	0.080856	1.0922877	-1.93799	1.937994	2	0.240222	GLYBt4_2_	Betaine transport (sodium symport) (2:1)	2.00 * Sodium [e] + 1.00 * glycine betaine [e] --> 2.00 * So	SLC6A12	Transport, extracellular			4
GLYt7_211_r_neg	9	0.080856	1.0922877	1.708507	1.708507	1	0.240222	GLYt7_211	glycine reversible transport via sodium and chloride symport (2:1:1)	2.00 * Sodium [e] + 1.00 * Glycine [e] + 1.00 * Chloride [e]	SLC6A9	Transport, extracellular			4
GLYt7_211_r_pos	9	0.080856	1.0922877	1.788393	1.788393	1	0.240222	GLYt7_211	glycine reversible transport via sodium and chloride symport (2:1:1)	2.00 * Sodium [e] + 1.00 * Glycine [e] + 1.00 * Chloride [e]	SLC6A9	Transport, extracellular			4
H2Ot_neg	0	0.080856	1.0922877	-2.13912	2.139124	2	0.240222	H2Ot	H2O transport via diffusion	1.00 * Water [e] --> 1.00 * Water [c]AQP1; AQP2; AQP4; A	AQP1; AQP	Transport, extracellular			4
H2Ot_pos	0	0.080856	1.0922877	-2.13615	2.136146	2	0.240222	H2Ot	H2O transport via diffusion	1.00 * Water [e] --> 1.00 * Water [c]AQP1; AQP2; AQP4; A	AQP1; AQP	Transport, extracellular			4
HCO3_CLt_pos	9	0.080856	1.0922877	1.973584	1.973584	1	0.240222	HCO3_CLt	bicarbonate transport (Cl-/HCO3- exchange)	1.00 * Bicarbonate [e] + 1.00 * Chloride [c] --> 1.00 * Bicar	SLC4A1; SL	Transport, extracellular			4
HIStN1_pos	9	0.080856	1.0922877	2.355038	2.355038	1	0.240222	HIStN1	Histidine transport (Na, H coupled)	1.00 * proton [c] + 2.00 * Sodium [e] + 1.00 * L-histidine [	SLC38A3	Transport, extracellular			4
HIStiDF_pos	9	0.080856	1.0922877	3.074572	3.074572	1	0.240222	HIStiDF	L-histidine transport via diffusion (extracellular to cytosol)	1.00 * L-histidine [e] --> 1.00 * L-histidine [c]SLC38A4	SLC38A4	Transport, extracellular			4
HISyLATthc_pos	9	0.080856	1.0922877	2.493042	2.493042	1	0.240222	HISyLATthc	Transport of Histidine by y+LAT1 or y+LAT2 with co-transporter of h	1.00 * proton [e] + 1.00 * L-argininium(1+) [c] + 1.00 * L-h	SLC3A2; SL	Transport, extracellular			4
HOMt4_pos	9	0.080856	1.0922877	2.459132	2.459132	1	0.240222	HOMt4	L-homoserine via sodium symport	1.00 * Sodium [e] + 1.00 * L-homoserine [e] --> 1.00 * Sodi	SLC7A1; SL	Transport, extracellular			4
ILEtec_neg	9	0.080856	1.0922877	2.477097	2.477097	1	0.240222	ILEtec	L-isoleucine transport via diffusion (extracellular to cytosol)	1.00 * L-isoleucine [e] --> 1.00 * L-isoleucine [c]SLC43A1;	SLC43A1; S	Transport, extracellular			4
ILEtec_pos	9	0.080856	1.0922877	2.477097	2.477097	1	0.240222	ILEtec	L-isoleucine transport via diffusion (extracellular to cytosol)	1.00 * L-isoleucine [e] --> 1.00 * L-isoleucine [c]SLC43A1;	SLC43A1; S	Transport, extracellular			4

INSTt4_2_neg	0	0.080856	1.0922877	-1.86623	1.866225	2	0.240222	INSTt4_2	inositol transport via sodium symport	2.00 * Sodium [e] + 1.00 * myo-inositol [e] --> 2.00 * Sodiu	SLC5A11	Transport, extracellular			4
INSTt4_2_pos	0	0.080856	1.0922877	-1.77669	1.776693	2	0.240222	INSTt4_2	inositol transport via sodium symport	2.00 * Sodium [e] + 1.00 * myo-inositol [e] --> 2.00 * Sodiu	SLC5A11	Transport, extracellular			4
LEUtec_neg	9	0.080856	1.0922877	2.025473	2.025473	1	0.240222	LEUtec	L-leucine transport via diffusion (extracellular to cytosol)	1.00 * L-leucine [e] --> 1.00 * L-leucine [c]SLC43A1; SLC43	SLC43A1; S	Transport, extracellular			4
LYSATB0tc_pos	9	0.080856	1.0922877	2.025314	2.025314	1	0.240222	LYSATB0tc	transport of L-Lysine into the intestinal cells by ATB0 transporter	2.00 * Sodium [e] + 1.00 * L-lysinium(1+) [e] + 1.00 * Chlo	SLC6A14	Transport, extracellular			4
LYStiDF_pos	9	0.080856	1.0922877	3.880678	3.880678	1	0.240222	LYStiDF	L-lysine transport via diffusion (extracellular to cytosol)	1.00 * L-lysinium(1+) [e] --> 1.00 * L-lysinium(1+) [c]SLC38	SLC38A4; S	Transport, extracellular			4
METLEUex_pos	9	0.080856	1.0922877	2.497908	2.497908	1	0.240222	METLEUex	Methionine/Leucine exchange (Met in)	1.00 * L-methionine [e] + 1.00 * L-leucine [c] --> 1.00 * L-m	SLC3A2; SL	Transport, extracellular			4
ORNALArBATtc_pos	9	0.080856	1.0922877	2.660891	2.660891	1	0.240222	ORNALArBA	transport of L-Ornithine into the cell in exchange for L-Alanine by b0	1.00 * L-alanine [c] + 1.00 * Ornithine [e] --> 1.00 * Ornith	SLC3A1; SL	Transport, extracellular			4
ORNLEUrBATtc_pos	9	0.080856	1.0922877	2.581164	2.581164	1	0.240222	ORNLEUrBA	transport of L-Ornithineinto the cell in exchange for L-Leucine by b0	1.00 * Ornithine [e] + 1.00 * L-leucine [c] --> 1.00 * Ornith	SLC3A1; SL	Transport, extracellular			4
ORNtiDF_pos	9	0.080856	1.0922877	2.854084	2.854084	1	0.240222	ORNtiDF	ornithine transport via diffusion (extracellular to cytosol)	1.00 * Ornithine [e] --> 1.00 * Ornithine [c]SLC7A1; SLC7A	SLC7A1; SL	Transport, extracellular			4
PIt7_neg	0	0.080856	1.0922877	-2.77478	2.774783	2	0.240222	PIt7	phosphate transport in/out via three Na+ symporter	3.00 * Sodium [e] + 1.00 * hydrogenphosphate [e] --> 1.00	SLC17A1; S	Transport, extracellular			4
PIt7_pos	0	0.080856	1.0922877	-2.44786	2.447855	2	0.240222	PIt7	phosphate transport in/out via three Na+ symporter	3.00 * Sodium [e] + 1.00 * hydrogenphosphate [e] --> 1.00	SLC17A1; S	Transport, extracellular			4
PIt8_neg	0	0.080856	1.0922877	-2.86625	2.866254	2	0.240222	PIt8	phosphate transport in/out via Na+ symporter	1.50 * Sodium [e] + 1.00 * hydrogenphosphate [e] --> 1.00	SLC20A1; S	Transport, extracellular			4
PIt8_pos	0	0.080856	1.0922877	-2.77478	2.774783	2	0.240222	PIt8	phosphate transport in/out via Na+ symporter	1.50 * Sodium [e] + 1.00 * hydrogenphosphate [e] --> 1.00	SLC20A1; S	Transport, extracellular			4
PIt9_neg	0	0.080856	1.0922877	-6.81309	6.813093	2	0.240222	PIt9	phosphate transport in/out via two Na+ symporter	2.00 * Sodium [e] + 1.00 * hydrogenphosphate [e] --> 1.00	SLC34A3	Transport, extracellular			4
PIt9_pos	0	0.080856	1.0922877	-6.81309	6.813093	2	0.240222	PIt9	phosphate transport in/out via two Na+ symporter	2.00 * Sodium [e] + 1.00 * hydrogenphosphate [e] --> 1.00	SLC34A3	Transport, extracellular			4
PROSTGF2t_neg	0	0.080856	1.0922877	-2.17032	2.170324	2	0.240222	PROSTGF2t	prostaglandin uniport	1.00 * prostaglandin F2alpha(1-) [e] --> 1.00 * prostagland	SLC22A1; S	Transport, extracellular			4
PROSTGF2t_pos	0	0.080856	1.0922877	-2.17032	2.170324	2	0.240222	PROSTGF2t	prostaglandin uniport	1.00 * prostaglandin F2alpha(1-) [e] --> 1.00 * prostagland	SLC22A1; S	Transport, extracellular			4
SERALANaEx_pos	9	0.080856	1.0922877	3.151917	3.151917	1	0.240222	SERALANaE	L-alanine/L-serine Na-dependent exchange (Ser-L in)	1.00 * Sodium [e] + 1.00 * L-alanine [c] + 1.00 * L-serine [e	SLC1A4; SL	Transport, extracellular			4
SERASNNaEx_pos	9	0.080856	1.0922877	2.402062	2.402062	1	0.240222	SERASNNaE	L-serine/L-asparagine Na-dependent exchange (Ser-L in)	1.00 * Sodium [e] + 1.00 * L-asparagine [c] + 1.00 * L-serin	SLC1A5	Transport, extracellular			4
SERCYSNaEx_pos	9	0.080856	1.0922877	3.151917	3.151917	1	0.240222	SERCYSNaE	L-serine/L-cysteine Na-dependent exchange (Ser-L in)	1.00 * Sodium [e] + 1.00 * L-cysteine [c] + 1.00 * L-serine [	SLC1A4; SL	Transport, extracellular			4
SERTHRNaEx_pos	9	0.080856	1.0922877	3.151917	3.151917	1	0.240222	SERTHRNaE	L-serine/L-threonine Na-dependent exchange (Ser-L in)	1.00 * Sodium [e] + 1.00 * L-serine [e] + 1.00 * L-threonine	SLC1A4; SL	Transport, extracellular			4
SERt4_pos	9	0.080856	1.0922877	1.969249	1.969249	1	0.240222	SERt4	L-serine via sodium symport	1.00 * Sodium [e] + 1.00 * L-serine [e] --> 1.00 * Sodium [c	ACE2; SLC3	Transport, extracellular			4
TCHOLAt2_pos	0	0.080856	1.0922877	-1.71208	1.712078	2	0.240222	TCHOLAt2	taurocholate transport via sodium cotransport	2.00 * Sodium [e] + 1.00 * taurocholate [e] --> 2.00 * Sodi	SLC10A1; S	Transport, extracellular			4
THRALANaEx_pos	9	0.080856	1.0922877	3.151917	3.151917	1	0.240222	THRALANaE	L-alanine/L-threonine Na-dependent exchange (Thr-L in)	1.00 * Sodium [e] + 1.00 * L-alanine [c] + 1.00 * L-threonir	SLC1A4; SL	Transport, extracellular			4
THRASNNaEx_pos	9	0.080856	1.0922877	2.402062	2.402062	1	0.240222	THRASNNaI	L-threonine/L-asparagine Na-dependent exchange (Thr-L in)	1.00 * Sodium [e] + 1.00 * L-asparagine [c] + 1.00 * L-threo	SLC1A5	Transport, extracellular			4
THRCYSNaEx_pos	9	0.080856	1.0922877	3.151917	3.151917	1	0.240222	THRCYSNaE	L-cysteine/L-threonine Na-dependent exchange (Thr-L in)	1.00 * Sodium [e] + 1.00 * L-cysteine [c] + 1.00 * L-threoni	SLC1A4; SL	Transport, extracellular			4
THRSERNaEx_pos	9	0.080856	1.0922877	3.151917	3.151917	1	0.240222	THRSERNaE	L-serine/L-threonine Na-dependent exchange (Thr-L in)	1.00 * Sodium [e] + 1.00 * L-serine [c] + 1.00 * L-threonine	SLC1A4; SL	Transport, extracellular			4
THRt4_pos	9	0.080856	1.0922877	2.195997	2.195997	1	0.240222	THRt4	L-threoninevia sodium symport	1.00 * Sodium [e] + 1.00 * L-threonine [e] --> 1.00 * Sodiu	ACE2; SLC3	Transport, extracellular			4
THYOXt2_neg	0	0.080856	1.0922877	-1.89558	1.895578	2	0.240222	THYOXt2	T4 transport via facilitated diffusion	1.00 * L-thyroxine [e] --> 1.00 * L-thyroxine [c]SLC16A2	SLC16A2	Transport, extracellular			4
THYOXt2_pos	0	0.080856	1.0922877	-1.95293	1.952935	2	0.240222	THYOXt2	T4 transport via facilitated diffusion	1.00 * L-thyroxine [e] --> 1.00 * L-thyroxine [c]SLC16A2	SLC16A2	Transport, extracellular			4
THYOXt_neg	0	0.080856	1.0922877	-1.95293	1.952935	2	0.240222	THYOXt	T4 transport via bicarbonate countertransport	1.00 * Bicarbonate [c] + 1.00 * L-thyroxine [e] --> 1.00 * Bi	SLCO1A1; S	Transport, extracellular			4
THYOXt_pos	0	0.080856	1.0922877	-1.89558	1.895578	2	0.240222	THYOXt	T4 transport via bicarbonate countertransport	1.00 * Bicarbonate [c] + 1.00 * L-thyroxine [e] --> 1.00 * Bi	SLCO1A1; S	Transport, extracellular			4
TRIODTHYt2_neg	0	0.080856	1.0922877	-1.94365	1.943647	2	0.240222	TRIODTHYt	T3 transport via facilitated diffusion	1.00 * 3,3',5-triiodo-L-thyronine [e] --> 1.00 * 3,3',5-triio	SLC16A2	Transport, extracellular			4
TRIODTHYt2_pos	0	0.080856	1.0922877	-2.00456	2.004563	2	0.240222	TRIODTHYt	T3 transport via facilitated diffusion	1.00 * 3,3',5-triiodo-L-thyronine [e] --> 1.00 * 3,3',5-triio	SLC16A2	Transport, extracellular			4
TRIODTHYt_neg	0	0.080856	1.0922877	-2.00456	2.004563	2	0.240222	TRIODTHYt	T3 transport via bicarbonate countertransport	1.00 * Bicarbonate [c] + 1.00 * 3,3',5-triiodo-L-thyronine	SLCO1A1; S	Transport, extracellular			4
TRIODTHYt_pos	0	0.080856	1.0922877	-1.94365	1.943647	2	0.240222	TRIODTHYt	T3 transport via bicarbonate countertransport	1.00 * Bicarbonate [c] + 1.00 * 3,3',5-triiodo-L-thyronine	SLCO1A1; S	Transport, extracellular			4
TRPt_neg	9	0.080856	1.0922877	2.352854	2.352854	1	0.240222	TRPt	L-tryptophan transport	1.00 * L-tryptophan [e] --> 1.00 * L-tryptophan [c]SLC16A	SLC16A10;	Transport, extracellular			4
TRPt_pos	9	0.080856	1.0922877	2.352854	2.352854	1	0.240222	TRPt	L-tryptophan transport	1.00 * L-tryptophan [e] --> 1.00 * L-tryptophan [c]SLC16A	SLC16A10;	Transport, extracellular			4
VALtec_neg	9	0.080856	1.0922877	2.477097	2.477097	1	0.240222	VALtec	L-valine transport via diffusion (extracellular to cytosol)	1.00 * L-valine [e] --> 1.00 * L-valine [c]SLC43A1; SLC43A2	SLC43A1; S	Transport, extracellular			4
VALtec_pos	9	0.080856	1.0922877	2.477097	2.477097	1	0.240222	VALtec	L-valine transport via diffusion (extracellular to cytosol)	1.00 * L-valine [e] --> 1.00 * L-valine [c]SLC43A1; SLC43A2	SLC43A1; S	Transport, extracellular			4
r0817_neg	9	0.080856	1.0922877	2.739064	2.739064	1	0.240222	r0817	Amino Acid-Polyamine-Organocation (APC) TCDB:2.A.3.3.2	1.00 * L-citrulline [c] --> 1.00 * L-citrulline [e]SLC7A2	SLC7A2	Transport, extracellular			0
r0817_pos	0	0.080856	1.0922877	-2.12943	2.129425	2	0.240222	r0817	Amino Acid-Polyamine-Organocation (APC) TCDB:2.A.3.3.2	1.00 * L-citrulline [c] --> 1.00 * L-citrulline [e]SLC7A2	SLC7A2	Transport, extracellular			0
r0946_neg	9	0.080856	1.0922877	2.610324	2.610324	1	0.240222	r0946	Amino Acid-Polyamine-Organocation (APC) TCDB:2.A.3.8.15	1.00 * L-citrulline [c] + 1.00 * Ornithine [e] --> 1.00 * Orni	SLC7A9	Transport, extracellular			0
r0946_pos	9	0.080856	1.0922877	2.937499	2.937499	1	0.240222	r0946	Amino Acid-Polyamine-Organocation (APC) TCDB:2.A.3.8.15	1.00 * L-citrulline [c] + 1.00 * Ornithine [e] --> 1.00 * Orni	SLC7A9	Transport, extracellular			0
r0992_neg	0	0.080856	1.0922877	-1.29975	1.299745	2	0.240222	r0992	Na(+)/bile acid cotransporter Active transport	1.00 * glycocholate [e] + 1.00 * Lepidimoide [e] --> 1.00 *	SLC10A1	Transport, extracellular			0
r0992_pos	0	0.080856	1.0922877	-1.43197	1.431969	2	0.240222	r0992	Na(+)/bile acid cotransporter Active transport	1.00 * glycocholate [e] + 1.00 * Lepidimoide [e] --> 1.00 *	SLC10A1	Transport, extracellular			0
r1027_neg	0	0.080856	1.0922877	-1.43197	1.431969	2	0.240222	r1027	Active transport	1.00 * glycochenodeoxycholate [e] + 1.00 * Lepidimoide	1.00 * glyc	Transport, extracellular			0
r1027_pos	0	0.080856	1.0922877	-1.29975	1.299745	2	0.240222	r1027	Active transport	1.00 * glycochenodeoxycholate [e] + 1.00 * Lepidimoide	1.00 * glyc	Transport, extracellular			0
r1043_pos	0	0.080856	1.0922877	-2.81968	2.819676	2	0.240222	r1043	Concentrative Nucleoside Transporter (CNT) TCDB:2.A.41.2.3	1.00 * Sodium [e] + 1.00 * 2-deoxyadenosine [e] --> 1.00 *	SLC28A1; S	Transport, extracellular			0
r1044_pos	0	0.080856	1.0922877	-2.81968	2.819676	2	0.240222	r1044	Concentrative Nucleoside Transporter (CNT) TCDB:2.A.41.2.3	1.00 * Sodium [e] + 1.00 * Deoxycytidine [e] --> 1.00 * Soc	SLC28A1; S	Transport, extracellular			0
r1498_pos	0	0.080856	1.0922877	-2.26359	2.263594	2	0.240222	r1498	Glycoside-Pentoside-Hexuronide (GPH):Cation Symporter TCDB:2.A	2.00 * Sodium [e] + 1.00 * glycochenodeoxycholate [e] -->	SLC10A1	Transport, extracellular			0
r1544_neg	9	0.080856	1.0922877	2.191991	2.191991	1	0.240222	r1544	Amino Acid-Polyamine-Organocation (APC) TCDB:2.A.3.8.1	1.00 * L-glutamine [c] + 1.00 * Glycine [e] --> 1.00 * L-glut	SLC7A5	Transport, extracellular			0
r1546_pos	9	0.080856	1.0922877	2.605207	2.605207	1	0.240222	r1546	Amino Acid-Polyamine-Organocation (APC) TCDB:2.A.3.8.1	1.00 * Glycine [e] + 1.00 * L-methionine [c] --> 1.00 * Glyc	SLC7A5	Transport, extracellular			0
r1547_neg	9	0.080856	1.0922877	2.067733	2.067733	1	0.240222	r1547	Amino Acid-Polyamine-Organocation (APC) TCDB:2.A.3.8.1	1.00 * Glycine [e] + 1.00 * L-tryptophan [c] --> 1.00 * Glyc	SLC7A5	Transport, extracellular			0
r1547_pos	9	0.080856	1.0922877	2.27118	2.27118	1	0.240222	r1547	Amino Acid-Polyamine-Organocation (APC) TCDB:2.A.3.8.1	1.00 * Glycine [e] + 1.00 * L-tryptophan [c] --> 1.00 * Glyc	SLC7A5	Transport, extracellular			0
r1548_neg	9	0.080856	1.0922877	2.08185	2.08185	1	0.240222	r1548	Amino Acid-Polyamine-Organocation (APC) TCDB:2.A.3.8.1	1.00 * Glycine [e] + 1.00 * L-phenylalanine [c] --> 1.00 * Gl	SLC7A5	Transport, extracellular			0
r1548_pos	9	0.080856	1.0922877	3.055923	3.055923	1	0.240222	r1548	Amino Acid-Polyamine-Organocation (APC) TCDB:2.A.3.8.1	1.00 * Glycine [e] + 1.00 * L-phenylalanine [c] --> 1.00 * Gl	SLC7A5	Transport, extracellular			0
r1549_neg	9	0.080856	1.0922877	2.067733	2.067733	1	0.240222	r1549	Amino Acid-Polyamine-Organocation (APC) TCDB:2.A.3.8.1	1.00 * Glycine [e] + 1.00 * L-tyrosine [c] --> 1.00 * Glycine	SLC7A5	Transport, extracellular			0
r1549_pos	9	0.080856	1.0922877	2.090819	2.090819	1	0.240222	r1549	Amino Acid-Polyamine-Organocation (APC) TCDB:2.A.3.8.1	1.00 * Glycine [e] + 1.00 * L-tyrosine [c] --> 1.00 * Glycine	SLC7A5	Transport, extracellular			0
r1551_neg	9	0.080856	1.0922877	2.022618	2.022618	1	0.240222	r1551	Amino Acid-Polyamine-Organocation (APC) TCDB:2.A.3.8.1	1.00 * Glycine [e] + 1.00 * L-leucine [c] --> 1.00 * Glycine [	SLC7A5	Transport, extracellular			0
r1551_pos	9	0.080856	1.0922877	2.605207	2.605207	1	0.240222	r1551	Amino Acid-Polyamine-Organocation (APC) TCDB:2.A.3.8.1	1.00 * Glycine [e] + 1.00 * L-leucine [c] --> 1.00 * Glycine [	SLC7A5	Transport, extracellular			0
r1552_neg	9	0.080856	1.0922877	2.156029	2.156029	1	0.240222	r1552	Amino Acid-Polyamine-Organocation (APC) TCDB:2.A.3.8.1	1.00 * Glycine [e] + 1.00 * L-proline [c] --> 1.00 * Glycine [	SLC7A5	Transport, extracellular			0
r1552_pos	9	0.080856	1.0922877	2.133601	2.133601	1	0.240222	r1552	Amino Acid-Polyamine-Organocation (APC) TCDB:2.A.3.8.1	1.00 * Glycine [e] + 1.00 * L-proline [c] --> 1.00 * Glycine [	SLC7A5	Transport, extracellular			0
r1553_neg	9	0.080856	1.0922877	2.072464	2.072464	1	0.240222	r1553	Amino Acid-Polyamine-Organocation (APC) TCDB:2.A.3.8.1	1.00 * L-asparagine [c] + 1.00 * Glycine [e] --> 1.00 * L-aspa	SLC7A5	Transport, extracellular			0
r1553_pos	9	0.080856	1.0922877	2.590316	2.590316	1	0.240222	r1553	Amino Acid-Polyamine-Organocation (APC) TCDB:2.A.3.8.1	1.00 * L-asparagine [c] + 1.00 * Glycine [e] --> 1.00 * L-aspa	SLC7A5	Transport, extracellular			0
r1554_neg	9	0.080856	1.0922877	2.022618	2.022618	1	0.240222	r1554	Amino Acid-Polyamine-Organocation (APC) TCDB:2.A.3.8.1	1.00 * Glycine [e] + 1.00 * L-valine [c] --> 1.00 * Glycine [c]	SLC7A5	Transport, extracellular			0
r1554_pos	9	0.080856	1.0922877	2.360613	2.360613	1	0.240222	r1554	Amino Acid-Polyamine-Organocation (APC) TCDB:2.A.3.8.1	1.00 * Glycine [e] + 1.00 * L-valine [c] --> 1.00 * Glycine [c]	SLC7A5	Transport, extracellular			0
r1556_neg	9	0.080856	1.0922877	2.020381	2.020381	1	0.240222	r1556	Amino Acid-Polyamine-Organocation (APC) TCDB:2.A.3.8.1	1.00 * Glycine [e] + 1.00 * L-homoserine [c] --> 1.00 * Glyc	SLC7A5	Transport, extracellular			0
r1556_pos	9	0.080856	1.0922877	2.532356	2.532356	1	0.240222	r1556	Amino Acid-Polyamine-Organocation (APC) TCDB:2.A.3.8.1	1.00 * Glycine [e] + 1.00 * L-homoserine [c] --> 1.00 * Glyc	SLC7A5	Transport, extracellular			0
r1557_neg	9	0.080856	1.0922877	2.022618	2.022618	1	0.240222	r1557	Amino Acid-Polyamine-Organocation (APC) TCDB:2.A.3.8.1	1.00 * Glycine [e] + 1.00 * L-isoleucine [c] --> 1.00 * Glycin	SLC7A5	Transport, extracellular			0
r1557_pos	9	0.080856	1.0922877	2.360613	2.360613	1	0.240222	r1557	Amino Acid-Polyamine-Organocation (APC) TCDB:2.A.3.8.1	1.00 * Glycine [e] + 1.00 * L-isoleucine [c] --> 1.00 * Glycin	SLC7A5	Transport, extracellular			0
r1559_neg	9	0.080856	1.0922877	2.691027	2.691027	1	0.240222	r1559	Amino Acid-Polyamine-Organocation (APC) TCDB:2.A.3.8.1	1.00 * L-alanine [e] + 1.00 * L-serine [c] --> 1.00 * L-alanine	SLC7A5	Transport, extracellular			0
r1559_pos	9	0.080856	1.0922877	2.763417	2.763417	1	0.240222	r1559	Amino Acid-Polyamine-Organocation (APC) TCDB:2.A.3.8.1	1.00 * L-alanine [e] + 1.00 * L-serine [c] --> 1.00 * L-alanine	SLC7A5	Transport, extracellular			0
r1560_pos	9	0.080856	1.0922877	2.741148	2.741148	1	0.240222	r1560	Amino Acid-Polyamine-Organocation (APC) TCDB:2.A.3.8.1	1.00 * L-alanine [e] + 1.00 * L-methionine [c] --> 1.00 * L-a	SLC7A5	Transport, extracellular			0
r1561_neg	9	0.080856	1.0922877	1.996924	1.996924	1	0.240222	r1561	Amino Acid-Polyamine-Organocation (APC) TCDB:2.A.3.8.1	1.00 * L-alanine [e] + 1.00 * L-tryptophan [c] --> 1.00 * L-a	SLC7A5	Transport, extracellular			0
r1561_pos	9	0.080856	1.0922877	4.374749	4.374749	1	0.240222	r1561	Amino Acid-Polyamine-Organocation (APC) TCDB:2.A.3.8.1	1.00 * L-alanine [e] + 1.00 * L-tryptophan [c] --> 1.00 * L-a	SLC7A5	Transport, extracellular			0
r1562_neg	9	0.080856	1.0922877	2.179097	2.179097	1	0.240222	r1562	Amino Acid-Polyamine-Organocation (APC) TCDB:2.A.3.8.1	1.00 * L-alanine [e] + 1.00 * L-phenylalanine [c] --> 1.00 * L	SLC7A5	Transport, extracellular			0
r1562_pos	9	0.080856	1.0922877	2.521254	2.521254	1	0.240222	r1562	Amino Acid-Polyamine-Organocation (APC) TCDB:2.A.3.8.1	1.00 * L-alanine [e] + 1.00 * L-phenylalanine [c] --> 1.00 * L	SLC7A5	Transport, extracellular			0
r1563_neg	9	0.080856	1.0922877	2.157995	2.157995	1	0.240222	r1563	Amino Acid-Polyamine-Organocation (APC) TCDB:2.A.3.8.1	1.00 * L-alanine [e] + 1.00 * L-tyrosine [c] --> 1.00 * L-alani	SLC7A5	Transport, extracellular			0
r1563_pos	9	0.080856	1.0922877	4.403818	4.403818	1	0.240222	r1563	Amino Acid-Polyamine-Organocation (APC) TCDB:2.A.3.8.1	1.00 * L-alanine [e] + 1.00 * L-tyrosine [c] --> 1.00 * L-alani	SLC7A5	Transport, extracellular			0
r1564_pos	9	0.080856	1.0922877	2.356737	2.356737	1	0.240222	r1564	Amino Acid-Polyamine-Organocation (APC) TCDB:2.A.3.8.1	1.00 * L-alanine [e] + 1.00 * L-cysteine [c] --> 1.00 * L-alani	SLC7A5	Transport, extracellular			0
r1565_neg	9	0.080856	1.0922877	2.30644	2.30644	1	0.240222	r1565	Amino Acid-Polyamine-Organocation (APC) TCDB:2.A.3.8.1	1.00 * L-alanine [e] + 1.00 * L-leucine [c] --> 1.00 * L-alanir	SLC7A5	Transport, extracellular			0
r1565_pos	9	0.080856	1.0922877	3.821305	3.821305	1	0.240222	r1565	Amino Acid-Polyamine-Organocation (APC) TCDB:2.A.3.8.1	1.00 * L-alanine [e] + 1.00 * L-leucine [c] --> 1.00 * L-alanir	SLC7A5	Transport, extracellular			0

r1566_neg	9	0.080856	1.0922877	2.666195	2.666195	1	0.240222	r1566	Amino Acid-Polyamine-Organocation (APC) TCDB:2.A.3.8.1	1.00 * L-alanine [e] + 1.00 * L-proline [c] --> 1.00 * L-alanir	SLC7A5	Transport, extracellular			0
r1566_pos	9	0.080856	1.0922877	2.958855	2.958855	1	0.240222	r1566	Amino Acid-Polyamine-Organocation (APC) TCDB:2.A.3.8.1	1.00 * L-alanine [e] + 1.00 * L-proline [c] --> 1.00 * L-alanir	SLC7A5	Transport, extracellular			0
r1568_neg	9	0.080856	1.0922877	2.30644	2.30644	1	0.240222	r1568	Amino Acid-Polyamine-Organocation (APC) TCDB:2.A.3.8.1	1.00 * L-alanine [e] + 1.00 * L-valine [c] --> 1.00 * L-alanine	SLC7A5	Transport, extracellular			0
r1568_pos	9	0.080856	1.0922877	3.821305	3.821305	1	0.240222	r1568	Amino Acid-Polyamine-Organocation (APC) TCDB:2.A.3.8.1	1.00 * L-alanine [e] + 1.00 * L-valine [c] --> 1.00 * L-alanine	SLC7A5	Transport, extracellular			0
r1569_neg	9	0.080856	1.0922877	2.691027	2.691027	1	0.240222	r1569	Amino Acid-Polyamine-Organocation (APC) TCDB:2.A.3.8.1	1.00 * L-alanine [e] + 1.00 * L-threonine [c] --> 1.00 * L-ala	SLC7A5	Transport, extracellular			0
r1569_pos	9	0.080856	1.0922877	2.763417	2.763417	1	0.240222	r1569	Amino Acid-Polyamine-Organocation (APC) TCDB:2.A.3.8.1	1.00 * L-alanine [e] + 1.00 * L-threonine [c] --> 1.00 * L-ala	SLC7A5	Transport, extracellular			0
r1570_neg	9	0.080856	1.0922877	2.306891	2.306891	1	0.240222	r1570	Amino Acid-Polyamine-Organocation (APC) TCDB:2.A.3.8.1	1.00 * L-alanine [e] + 1.00 * L-homoserine [c] --> 1.00 * L-a	SLC7A5	Transport, extracellular			0
r1570_pos	9	0.080856	1.0922877	2.695255	2.695255	1	0.240222	r1570	Amino Acid-Polyamine-Organocation (APC) TCDB:2.A.3.8.1	1.00 * L-alanine [e] + 1.00 * L-homoserine [c] --> 1.00 * L-a	SLC7A5	Transport, extracellular			0
r1571_neg	9	0.080856	1.0922877	2.30644	2.30644	1	0.240222	r1571	Amino Acid-Polyamine-Organocation (APC) TCDB:2.A.3.8.1	1.00 * L-alanine [e] + 1.00 * L-isoleucine [c] --> 1.00 * L-ala	SLC7A5	Transport, extracellular			0
r1571_pos	9	0.080856	1.0922877	3.821305	3.821305	1	0.240222	r1571	Amino Acid-Polyamine-Organocation (APC) TCDB:2.A.3.8.1	1.00 * L-alanine [e] + 1.00 * L-isoleucine [c] --> 1.00 * L-ala	SLC7A5	Transport, extracellular			0
r1573_pos	9	0.080856	1.0922877	2.025473	2.025473	1	0.240222	r1573	Amino Acid-Polyamine-Organocation (APC) TCDB:2.A.3.8.1	1.00 * L-glutamine [e] + 1.00 * L-methionine [c] --> 1.00 *	SLC7A5	Transport, extracellular			0
r1575_neg	9	0.080856	1.0922877	1.968055	1.968055	1	0.240222	r1575	Amino Acid-Polyamine-Organocation (APC) TCDB:2.A.3.8.1	1.00 * L-glutamine [e] + 1.00 * L-phenylalanine [c] --> 1.00	SLC7A5	Transport, extracellular			0
r1575_pos	9	0.080856	1.0922877	1.968055	1.968055	1	0.240222	r1575	Amino Acid-Polyamine-Organocation (APC) TCDB:2.A.3.8.1	1.00 * L-glutamine [e] + 1.00 * L-phenylalanine [c] --> 1.00	SLC7A5	Transport, extracellular			0
r1578_neg	9	0.080856	1.0922877	2.025473	2.025473	1	0.240222	r1578	Amino Acid-Polyamine-Organocation (APC) TCDB:2.A.3.8.1	1.00 * L-glutamine [e] + 1.00 * L-leucine [c] --> 1.00 * L-glu	SLC7A5	Transport, extracellular			0
r1578_pos	9	0.080856	1.0922877	2.025473	2.025473	1	0.240222	r1578	Amino Acid-Polyamine-Organocation (APC) TCDB:2.A.3.8.1	1.00 * L-glutamine [e] + 1.00 * L-leucine [c] --> 1.00 * L-glu	SLC7A5	Transport, extracellular			0
r1579_neg	9	0.080856	1.0922877	2.40533	2.40533	1	0.240222	r1579	Amino Acid-Polyamine-Organocation (APC) TCDB:2.A.3.8.1	1.00 * L-glutamine [e] + 1.00 * L-proline [c] --> 1.00 * L-glu	SLC7A5	Transport, extracellular			0
r1579_pos	9	0.080856	1.0922877	2.404344	2.404344	1	0.240222	r1579	Amino Acid-Polyamine-Organocation (APC) TCDB:2.A.3.8.1	1.00 * L-glutamine [e] + 1.00 * L-proline [c] --> 1.00 * L-glu	SLC7A5	Transport, extracellular			0
r1581_neg	9	0.080856	1.0922877	2.025473	2.025473	1	0.240222	r1581	Amino Acid-Polyamine-Organocation (APC) TCDB:2.A.3.8.1	1.00 * L-glutamine [e] + 1.00 * L-valine [c] --> 1.00 * L-glut	SLC7A5	Transport, extracellular			0
r1581_pos	9	0.080856	1.0922877	2.025473	2.025473	1	0.240222	r1581	Amino Acid-Polyamine-Organocation (APC) TCDB:2.A.3.8.1	1.00 * L-glutamine [e] + 1.00 * L-valine [c] --> 1.00 * L-glut	SLC7A5	Transport, extracellular			0
r1583_neg	9	0.080856	1.0922877	2.004175	2.004175	1	0.240222	r1583	Amino Acid-Polyamine-Organocation (APC) TCDB:2.A.3.8.1	1.00 * L-glutamine [e] + 1.00 * L-homoserine [c] --> 1.00 *	SLC7A5	Transport, extracellular			0
r1583_pos	9	0.080856	1.0922877	2.004175	2.004175	1	0.240222	r1583	Amino Acid-Polyamine-Organocation (APC) TCDB:2.A.3.8.1	1.00 * L-glutamine [e] + 1.00 * L-homoserine [c] --> 1.00 *	SLC7A5	Transport, extracellular			0
r1584_neg	9	0.080856	1.0922877	2.025473	2.025473	1	0.240222	r1584	Amino Acid-Polyamine-Organocation (APC) TCDB:2.A.3.8.1	1.00 * L-glutamine [e] + 1.00 * L-isoleucine [c] --> 1.00 * L-	SLC7A5	Transport, extracellular			0
r1584_pos	9	0.080856	1.0922877	2.025473	2.025473	1	0.240222	r1584	Amino Acid-Polyamine-Organocation (APC) TCDB:2.A.3.8.1	1.00 * L-glutamine [e] + 1.00 * L-isoleucine [c] --> 1.00 * L-	SLC7A5	Transport, extracellular			0
r1585_pos	9	0.080856	1.0922877	2.025473	2.025473	1	0.240222	r1585	Amino Acid-Polyamine-Organocation (APC) TCDB:2.A.3.8.1	1.00 * L-serine [e] + 1.00 * L-methionine [c] --> 1.00 * L-ser	SLC7A5	Transport, extracellular			0
r1587_neg	9	0.080856	1.0922877	1.968055	1.968055	1	0.240222	r1587	Amino Acid-Polyamine-Organocation (APC) TCDB:2.A.3.8.1	1.00 * L-serine [e] + 1.00 * L-phenylalanine [c] --> 1.00 * L-	SLC7A5	Transport, extracellular			0
r1587_pos	9	0.080856	1.0922877	1.968055	1.968055	1	0.240222	r1587	Amino Acid-Polyamine-Organocation (APC) TCDB:2.A.3.8.1	1.00 * L-serine [e] + 1.00 * L-phenylalanine [c] --> 1.00 * L-	SLC7A5	Transport, extracellular			0
r1589_neg	9	0.080856	1.0922877	2.047343	2.047343	1	0.240222	r1589	Amino Acid-Polyamine-Organocation (APC) TCDB:2.A.3.8.1	1.00 * L-cysteine [c] + 1.00 * L-serine [e] --> 1.00 * L-cystei	SLC7A5	Transport, extracellular			0
r1589_pos	9	0.080856	1.0922877	2.993375	2.993375	1	0.240222	r1589	Amino Acid-Polyamine-Organocation (APC) TCDB:2.A.3.8.1	1.00 * L-cysteine [c] + 1.00 * L-serine [e] --> 1.00 * L-cystei	SLC7A5	Transport, extracellular			0
r1590_neg	9	0.080856	1.0922877	2.025473	2.025473	1	0.240222	r1590	Amino Acid-Polyamine-Organocation (APC) TCDB:2.A.3.8.1	1.00 * L-serine [e] + 1.00 * L-leucine [c] --> 1.00 * L-serine [	SLC7A5	Transport, extracellular			0
r1590_pos	9	0.080856	1.0922877	2.025473	2.025473	1	0.240222	r1590	Amino Acid-Polyamine-Organocation (APC) TCDB:2.A.3.8.1	1.00 * L-serine [e] + 1.00 * L-leucine [c] --> 1.00 * L-serine [	SLC7A5	Transport, extracellular			0
r1591_neg	9	0.080856	1.0922877	2.40533	2.40533	1	0.240222	r1591	Amino Acid-Polyamine-Organocation (APC) TCDB:2.A.3.8.1	1.00 * L-serine [e] + 1.00 * L-proline [c] --> 1.00 * L-serine [	SLC7A5	Transport, extracellular			0
r1591_pos	9	0.080856	1.0922877	2.306891	2.306891	1	0.240222	r1591	Amino Acid-Polyamine-Organocation (APC) TCDB:2.A.3.8.1	1.00 * L-serine [e] + 1.00 * L-proline [c] --> 1.00 * L-serine [	SLC7A5	Transport, extracellular			0
r1592_neg	9	0.080856	1.0922877	2.004175	2.004175	1	0.240222	r1592	Amino Acid-Polyamine-Organocation (APC) TCDB:2.A.3.8.1	1.00 * L-asparagine [c] + 1.00 * L-serine [e] --> 1.00 * L-asp	SLC7A5	Transport, extracellular			0
r1592_pos	9	0.080856	1.0922877	2.004175	2.004175	1	0.240222	r1592	Amino Acid-Polyamine-Organocation (APC) TCDB:2.A.3.8.1	1.00 * L-asparagine [c] + 1.00 * L-serine [e] --> 1.00 * L-asp	SLC7A5	Transport, extracellular			0
r1593_neg	9	0.080856	1.0922877	2.025473	2.025473	1	0.240222	r1593	Amino Acid-Polyamine-Organocation (APC) TCDB:2.A.3.8.1	1.00 * L-serine [e] + 1.00 * L-valine [c] --> 1.00 * L-serine [c	SLC7A5	Transport, extracellular			0
r1593_pos	9	0.080856	1.0922877	2.025473	2.025473	1	0.240222	r1593	Amino Acid-Polyamine-Organocation (APC) TCDB:2.A.3.8.1	1.00 * L-serine [e] + 1.00 * L-valine [c] --> 1.00 * L-serine [c	SLC7A5	Transport, extracellular			0
r1594_neg	9	0.080856	1.0922877	2.123095	2.123095	1	0.240222	r1594	Amino Acid-Polyamine-Organocation (APC) TCDB:2.A.3.8.1	1.00 * L-serine [e] + 1.00 * L-threonine [c] --> 1.00 * L-serir	SLC7A5	Transport, extracellular			0
r1594_pos	9	0.080856	1.0922877	2.123095	2.123095	1	0.240222	r1594	Amino Acid-Polyamine-Organocation (APC) TCDB:2.A.3.8.1	1.00 * L-serine [e] + 1.00 * L-threonine [c] --> 1.00 * L-serir	SLC7A5	Transport, extracellular			0
r1595_neg	9	0.080856	1.0922877	2.004175	2.004175	1	0.240222	r1595	Amino Acid-Polyamine-Organocation (APC) TCDB:2.A.3.8.1	1.00 * L-serine [e] + 1.00 * L-homoserine [c] --> 1.00 * L-se	SLC7A5	Transport, extracellular			0
r1595_pos	9	0.080856	1.0922877	2.004175	2.004175	1	0.240222	r1595	Amino Acid-Polyamine-Organocation (APC) TCDB:2.A.3.8.1	1.00 * L-serine [e] + 1.00 * L-homoserine [c] --> 1.00 * L-se	SLC7A5	Transport, extracellular			0
r1596_neg	9	0.080856	1.0922877	2.025473	2.025473	1	0.240222	r1596	Amino Acid-Polyamine-Organocation (APC) TCDB:2.A.3.8.1	1.00 * L-serine [e] + 1.00 * L-isoleucine [c] --> 1.00 * L-serir	SLC7A5	Transport, extracellular			0
r1596_pos	9	0.080856	1.0922877	2.025473	2.025473	1	0.240222	r1596	Amino Acid-Polyamine-Organocation (APC) TCDB:2.A.3.8.1	1.00 * L-serine [e] + 1.00 * L-isoleucine [c] --> 1.00 * L-serir	SLC7A5	Transport, extracellular			0
r1597_pos	9	0.080856	1.0922877	3.132677	3.132677	1	0.240222	r1597	Amino Acid-Polyamine-Organocation (APC) TCDB:2.A.3.8.1	1.00 * L-methionine [e] + 1.00 * L-tryptophan [c] --> 1.00	SLC7A5	Transport, extracellular			0
r1598_neg	9	0.080856	1.0922877	1.988084	1.988084	1	0.240222	r1598	Amino Acid-Polyamine-Organocation (APC) TCDB:2.A.3.8.1	1.00 * L-methionine [e] + 1.00 * L-phenylalanine [c] --> 1.0	SLC7A5	Transport, extracellular			0
r1599_pos	9	0.080856	1.0922877	2.961327	2.961327	1	0.240222	r1599	Amino Acid-Polyamine-Organocation (APC) TCDB:2.A.3.8.1	1.00 * L-methionine [e] + 1.00 * L-tyrosine [c] --> 1.00 * L-	SLC7A5	Transport, extracellular			0
r1602_neg	9	0.080856	1.0922877	2.411045	2.411045	1	0.240222	r1602	Amino Acid-Polyamine-Organocation (APC) TCDB:2.A.3.8.1	1.00 * L-methionine [e] + 1.00 * L-proline [c] --> 1.00 * L-n	SLC7A5	Transport, extracellular			0
r1602_pos	9	0.080856	1.0922877	4.02755	4.02755	1	0.240222	r1602	Amino Acid-Polyamine-Organocation (APC) TCDB:2.A.3.8.1	1.00 * L-methionine [e] + 1.00 * L-proline [c] --> 1.00 * L-n	SLC7A5	Transport, extracellular			0
r1603_neg	9	0.080856	1.0922877	2.025473	2.025473	1	0.240222	r1603	Amino Acid-Polyamine-Organocation (APC) TCDB:2.A.3.8.1	1.00 * L-asparagine [c] + 1.00 * L-methionine [e] --> 1.00 *	SLC7A5	Transport, extracellular			0
r1604_neg	9	0.080856	1.0922877	2.046774	2.046774	1	0.240222	r1604	Amino Acid-Polyamine-Organocation (APC) TCDB:2.A.3.8.1	1.00 * L-methionine [e] + 1.00 * L-valine [c] --> 1.00 * L-me	SLC7A5	Transport, extracellular			0
r1604_pos	9	0.080856	1.0922877	3.104513	3.104513	1	0.240222	r1604	Amino Acid-Polyamine-Organocation (APC) TCDB:2.A.3.8.1	1.00 * L-methionine [e] + 1.00 * L-valine [c] --> 1.00 * L-me	SLC7A5	Transport, extracellular			0
r1605_neg	9	0.080856	1.0922877	2.025473	2.025473	1	0.240222	r1605	Amino Acid-Polyamine-Organocation (APC) TCDB:2.A.3.8.1	1.00 * L-threonine [c] + 1.00 * L-methionine [e] --> 1.00 *	SLC7A5	Transport, extracellular			0
r1606_neg	9	0.080856	1.0922877	2.025473	2.025473	1	0.240222	r1606	Amino Acid-Polyamine-Organocation (APC) TCDB:2.A.3.8.1	1.00 * L-methionine [e] + 1.00 * L-homoserine [c] --> 1.00	SLC7A5	Transport, extracellular			0
r1607_neg	9	0.080856	1.0922877	2.046774	2.046774	1	0.240222	r1607	Amino Acid-Polyamine-Organocation (APC) TCDB:2.A.3.8.1	1.00 * L-methionine [e] + 1.00 * L-isoleucine [c] --> 1.00 *	SLC7A5	Transport, extracellular			0
r1607_pos	9	0.080856	1.0922877	3.104513	3.104513	1	0.240222	r1607	Amino Acid-Polyamine-Organocation (APC) TCDB:2.A.3.8.1	1.00 * L-methionine [e] + 1.00 * L-isoleucine [c] --> 1.00 *	SLC7A5	Transport, extracellular			0
r1610_neg	9	0.080856	1.0922877	4.381375	4.381375	1	0.240222	r1610	Amino Acid-Polyamine-Organocation (APC) TCDB:2.A.3.8.1	1.00 * L-cysteine [c] + 1.00 * L-tryptophan [e] --> 1.00 * L-c	SLC7A5	Transport, extracellular			0
r1610_pos	9	0.080856	1.0922877	1.886905	1.886905	1	0.240222	r1610	Amino Acid-Polyamine-Organocation (APC) TCDB:2.A.3.8.1	1.00 * L-cysteine [c] + 1.00 * L-tryptophan [e] --> 1.00 * L-c	SLC7A5	Transport, extracellular			0
r1627_neg	9	0.080856	1.0922877	4.036714	4.036714	1	0.240222	r1627	Amino Acid-Polyamine-Organocation (APC) TCDB:2.A.3.8.1	1.00 * L-cysteine [c] + 1.00 * L-tyrosine [e] --> 1.00 * L-cyst	SLC7A5	Transport, extracellular			0
r1627_pos	9	0.080856	1.0922877	1.879925	1.879925	1	0.240222	r1627	Amino Acid-Polyamine-Organocation (APC) TCDB:2.A.3.8.1	1.00 * L-cysteine [c] + 1.00 * L-tyrosine [e] --> 1.00 * L-cyst	SLC7A5	Transport, extracellular			0
r1629_neg	9	0.080856	1.0922877	2.339703	2.339703	1	0.240222	r1629	Amino Acid-Polyamine-Organocation (APC) TCDB:2.A.3.8.1	1.00 * L-tyrosine [e] + 1.00 * L-proline [c] --> 1.00 * L-proli	SLC7A5	Transport, extracellular			0
r1629_pos	9	0.080856	1.0922877	2.157995	2.157995	1	0.240222	r1629	Amino Acid-Polyamine-Organocation (APC) TCDB:2.A.3.8.1	1.00 * L-tyrosine [e] + 1.00 * L-proline [c] --> 1.00 * L-proli	SLC7A5	Transport, extracellular			0
r1635_neg	9	0.080856	1.0922877	1.921724	1.921724	1	0.240222	r1635	Amino Acid-Polyamine-Organocation (APC) TCDB:2.A.3.8.1	1.00 * L-cysteine [e] + 1.00 * L-leucine [c] --> 1.00 * L-cyste	SLC7A5	Transport, extracellular			0
r1635_pos	9	0.080856	1.0922877	4.155919	4.155919	1	0.240222	r1635	Amino Acid-Polyamine-Organocation (APC) TCDB:2.A.3.8.1	1.00 * L-cysteine [e] + 1.00 * L-leucine [c] --> 1.00 * L-cyste	SLC7A5	Transport, extracellular			0
r1636_neg	9	0.080856	1.0922877	2.019342	2.019342	1	0.240222	r1636	Amino Acid-Polyamine-Organocation (APC) TCDB:2.A.3.8.1	1.00 * L-cysteine [e] + 1.00 * L-proline [c] --> 1.00 * L-cyste	SLC7A5	Transport, extracellular			0
r1636_pos	9	0.080856	1.0922877	3.884135	3.884135	1	0.240222	r1636	Amino Acid-Polyamine-Organocation (APC) TCDB:2.A.3.8.1	1.00 * L-cysteine [e] + 1.00 * L-proline [c] --> 1.00 * L-cyste	SLC7A5	Transport, extracellular			0
r1638_neg	9	0.080856	1.0922877	1.921724	1.921724	1	0.240222	r1638	Amino Acid-Polyamine-Organocation (APC) TCDB:2.A.3.8.1	1.00 * L-cysteine [e] + 1.00 * L-valine [c] --> 1.00 * L-cystei	SLC7A5	Transport, extracellular			0
r1638_pos	9	0.080856	1.0922877	4.155919	4.155919	1	0.240222	r1638	Amino Acid-Polyamine-Organocation (APC) TCDB:2.A.3.8.1	1.00 * L-cysteine [e] + 1.00 * L-valine [c] --> 1.00 * L-cystei	SLC7A5	Transport, extracellular			0
r1639_neg	9	0.080856	1.0922877	2.993375	2.993375	1	0.240222	r1639	Amino Acid-Polyamine-Organocation (APC) TCDB:2.A.3.8.1	1.00 * L-cysteine [e] + 1.00 * L-threonine [c] --> 1.00 * L-cy	SLC7A5	Transport, extracellular			0
r1639_pos	9	0.080856	1.0922877	2.351951	2.351951	1	0.240222	r1639	Amino Acid-Polyamine-Organocation (APC) TCDB:2.A.3.8.1	1.00 * L-cysteine [e] + 1.00 * L-threonine [c] --> 1.00 * L-cy	SLC7A5	Transport, extracellular			0
r1640_neg	9	0.080856	1.0922877	1.919999	1.919999	1	0.240222	r1640	Amino Acid-Polyamine-Organocation (APC) TCDB:2.A.3.8.1	1.00 * L-cysteine [e] + 1.00 * L-homoserine [c] --> 1.00 * L-	SLC7A5	Transport, extracellular			0
r1641_neg	9	0.080856	1.0922877	1.921724	1.921724	1	0.240222	r1641	Amino Acid-Polyamine-Organocation (APC) TCDB:2.A.3.8.1	1.00 * L-cysteine [e] + 1.00 * L-isoleucine [c] --> 1.00 * L-cy	SLC7A5	Transport, extracellular			0
r1641_pos	9	0.080856	1.0922877	4.155919	4.155919	1	0.240222	r1641	Amino Acid-Polyamine-Organocation (APC) TCDB:2.A.3.8.1	1.00 * L-cysteine [e] + 1.00 * L-isoleucine [c] --> 1.00 * L-cy	SLC7A5	Transport, extracellular			0
r1642_neg	9	0.080856	1.0922877	2.411045	2.411045	1	0.240222	r1642	Amino Acid-Polyamine-Organocation (APC) TCDB:2.A.3.8.1	1.00 * L-leucine [e] + 1.00 * L-proline [c] --> 1.00 * L-prolir	SLC7A5	Transport, extracellular			0
r1642_pos	9	0.080856	1.0922877	2.30644	2.30644	1	0.240222	r1642	Amino Acid-Polyamine-Organocation (APC) TCDB:2.A.3.8.1	1.00 * L-leucine [e] + 1.00 * L-proline [c] --> 1.00 * L-prolir	SLC7A5	Transport, extracellular			0
r1643_neg	9	0.080856	1.0922877	2.025473	2.025473	1	0.240222	r1643	Amino Acid-Polyamine-Organocation (APC) TCDB:2.A.3.8.1	1.00 * L-asparagine [c] + 1.00 * L-leucine [e] --> 1.00 * L-as	SLC7A5	Transport, extracellular			0
r1643_pos	9	0.080856	1.0922877	2.025473	2.025473	1	0.240222	r1643	Amino Acid-Polyamine-Organocation (APC) TCDB:2.A.3.8.1	1.00 * L-asparagine [c] + 1.00 * L-leucine [e] --> 1.00 * L-as	SLC7A5	Transport, extracellular			0
r1645_neg	9	0.080856	1.0922877	2.025473	2.025473	1	0.240222	r1645	Amino Acid-Polyamine-Organocation (APC) TCDB:2.A.3.8.1	1.00 * L-threonine [c] + 1.00 * L-leucine [e] --> 1.00 * L-thr	SLC7A5	Transport, extracellular			0
r1645_pos	9	0.080856	1.0922877	2.025473	2.025473	1	0.240222	r1645	Amino Acid-Polyamine-Organocation (APC) TCDB:2.A.3.8.1	1.00 * L-threonine [c] + 1.00 * L-leucine [e] --> 1.00 * L-thr	SLC7A5	Transport, extracellular			0
r1646_neg	9	0.080856	1.0922877	2.025473	2.025473	1	0.240222	r1646	Amino Acid-Polyamine-Organocation (APC) TCDB:2.A.3.8.1	1.00 * L-leucine [e] + 1.00 * L-homoserine [c] --> 1.00 * L-h	SLC7A5	Transport, extracellular			0
r1646_pos	9	0.080856	1.0922877	2.025473	2.025473	1	0.240222	r1646	Amino Acid-Polyamine-Organocation (APC) TCDB:2.A.3.8.1	1.00 * L-leucine [e] + 1.00 * L-homoserine [c] --> 1.00 * L-h	SLC7A5	Transport, extracellular			0
r1648_neg	9	0.080856	1.0922877	2.404344	2.404344	1	0.240222	r1648	Amino Acid-Polyamine-Organocation (APC) TCDB:2.A.3.8.1	1.00 * L-asparagine [c] + 1.00 * L-proline [e] --> 1.00 * L-as	SLC7A5	Transport, extracellular			0
r1648_pos	9	0.080856	1.0922877	2.40533	2.40533	1	0.240222	r1648	Amino Acid-Polyamine-Organocation (APC) TCDB:2.A.3.8.1	1.00 * L-asparagine [c] + 1.00 * L-proline [e] --> 1.00 * L-as	SLC7A5	Transport, extracellular			0
r1649_neg	9	0.080856	1.0922877	2.30644	2.30644	1	0.240222	r1649	Amino Acid-Polyamine-Organocation (APC) TCDB:2.A.3.8.1	1.00 * L-proline [e] + 1.00 * L-valine [c] --> 1.00 * L-valine [	SLC7A5	Transport, extracellular			0
r1649_pos	9	0.080856	1.0922877	2.411045	2.411045	1	0.240222	r1649	Amino Acid-Polyamine-Organocation (APC) TCDB:2.A.3.8.1	1.00 * L-proline [e] + 1.00 * L-valine [c] --> 1.00 * L-valine [	SLC7A5	Transport, extracellular			0

r1650_neg	9	0.080856	1.0922877	2.306891	2.306891	1	0.240222	r1650	Amino Acid-Polyamine-Organocation (APC) TCDB:2.A.3.8.1	1.00 * L-threonine [c] + 1.00 * L-proline [e] --> 1.00 * L-thr	SLC7A5	Transport, extracellular			0
r1650_pos	9	0.080856	1.0922877	2.40533	2.40533	1	0.240222	r1650	Amino Acid-Polyamine-Organocation (APC) TCDB:2.A.3.8.1	1.00 * L-threonine [c] + 1.00 * L-proline [e] --> 1.00 * L-thr	SLC7A5	Transport, extracellular			0
r1651_neg	9	0.080856	1.0922877	2.306891	2.306891	1	0.240222	r1651	Amino Acid-Polyamine-Organocation (APC) TCDB:2.A.3.8.1	1.00 * L-proline [e] + 1.00 * L-homoserine [c] --> 1.00 * L-h	SLC7A5	Transport, extracellular			0
r1651_pos	9	0.080856	1.0922877	2.40533	2.40533	1	0.240222	r1651	Amino Acid-Polyamine-Organocation (APC) TCDB:2.A.3.8.1	1.00 * L-proline [e] + 1.00 * L-homoserine [c] --> 1.00 * L-h	SLC7A5	Transport, extracellular			0
r1652_neg	9	0.080856	1.0922877	2.30644	2.30644	1	0.240222	r1652	Amino Acid-Polyamine-Organocation (APC) TCDB:2.A.3.8.1	1.00 * L-proline [e] + 1.00 * L-isoleucine [c] --> 1.00 * L-iso	SLC7A5	Transport, extracellular			0
r1652_pos	9	0.080856	1.0922877	2.411045	2.411045	1	0.240222	r1652	Amino Acid-Polyamine-Organocation (APC) TCDB:2.A.3.8.1	1.00 * L-proline [e] + 1.00 * L-isoleucine [c] --> 1.00 * L-iso	SLC7A5	Transport, extracellular			0
r1653_neg	9	0.080856	1.0922877	2.025473	2.025473	1	0.240222	r1653	Amino Acid-Polyamine-Organocation (APC) TCDB:2.A.3.8.1	1.00 * L-asparagine [e] + 1.00 * L-valine [c] --> 1.00 * L-asp	SLC7A5	Transport, extracellular			0
r1653_pos	9	0.080856	1.0922877	2.025473	2.025473	1	0.240222	r1653	Amino Acid-Polyamine-Organocation (APC) TCDB:2.A.3.8.1	1.00 * L-asparagine [e] + 1.00 * L-valine [c] --> 1.00 * L-asp	SLC7A5	Transport, extracellular			0
r1654_neg	9	0.080856	1.0922877	2.004175	2.004175	1	0.240222	r1654	Amino Acid-Polyamine-Organocation (APC) TCDB:2.A.3.8.1	1.00 * L-asparagine [e] + 1.00 * L-threonine [c] --> 1.00 * L-	SLC7A5	Transport, extracellular			0
r1654_pos	9	0.080856	1.0922877	2.004175	2.004175	1	0.240222	r1654	Amino Acid-Polyamine-Organocation (APC) TCDB:2.A.3.8.1	1.00 * L-asparagine [e] + 1.00 * L-threonine [c] --> 1.00 * L-	SLC7A5	Transport, extracellular			0
r1655_neg	9	0.080856	1.0922877	2.004175	2.004175	1	0.240222	r1655	Amino Acid-Polyamine-Organocation (APC) TCDB:2.A.3.8.1	1.00 * L-asparagine [e] + 1.00 * L-homoserine [c] --> 1.00 *	SLC7A5	Transport, extracellular			0
r1655_pos	9	0.080856	1.0922877	2.004175	2.004175	1	0.240222	r1655	Amino Acid-Polyamine-Organocation (APC) TCDB:2.A.3.8.1	1.00 * L-asparagine [e] + 1.00 * L-homoserine [c] --> 1.00 *	SLC7A5	Transport, extracellular			0
r1656_neg	9	0.080856	1.0922877	2.025473	2.025473	1	0.240222	r1656	Amino Acid-Polyamine-Organocation (APC) TCDB:2.A.3.8.1	1.00 * L-asparagine [e] + 1.00 * L-isoleucine [c] --> 1.00 * L	SLC7A5	Transport, extracellular			0
r1656_pos	9	0.080856	1.0922877	2.025473	2.025473	1	0.240222	r1656	Amino Acid-Polyamine-Organocation (APC) TCDB:2.A.3.8.1	1.00 * L-asparagine [e] + 1.00 * L-isoleucine [c] --> 1.00 * L	SLC7A5	Transport, extracellular			0
r1657_neg	9	0.080856	1.0922877	2.025473	2.025473	1	0.240222	r1657	Amino Acid-Polyamine-Organocation (APC) TCDB:2.A.3.8.1	1.00 * L-threonine [c] + 1.00 * L-valine [e] --> 1.00 * L-thre	SLC7A5	Transport, extracellular			0
r1657_pos	9	0.080856	1.0922877	2.025473	2.025473	1	0.240222	r1657	Amino Acid-Polyamine-Organocation (APC) TCDB:2.A.3.8.1	1.00 * L-threonine [c] + 1.00 * L-valine [e] --> 1.00 * L-thre	SLC7A5	Transport, extracellular			0
r1658_neg	9	0.080856	1.0922877	2.025473	2.025473	1	0.240222	r1658	Amino Acid-Polyamine-Organocation (APC) TCDB:2.A.3.8.1	1.00 * L-valine [e] + 1.00 * L-homoserine [c] --> 1.00 * L-ho	SLC7A5	Transport, extracellular			0
r1658_pos	9	0.080856	1.0922877	2.025473	2.025473	1	0.240222	r1658	Amino Acid-Polyamine-Organocation (APC) TCDB:2.A.3.8.1	1.00 * L-valine [e] + 1.00 * L-homoserine [c] --> 1.00 * L-ho	SLC7A5	Transport, extracellular			0
r1659_neg	9	0.080856	1.0922877	2.046774	2.046774	1	0.240222	r1659	Amino Acid-Polyamine-Organocation (APC) TCDB:2.A.3.8.1	1.00 * L-valine [e] + 1.00 * L-isoleucine [c] --> 1.00 * L-isol	SLC7A5	Transport, extracellular			0
r1659_pos	9	0.080856	1.0922877	2.046774	2.046774	1	0.240222	r1659	Amino Acid-Polyamine-Organocation (APC) TCDB:2.A.3.8.1	1.00 * L-valine [e] + 1.00 * L-isoleucine [c] --> 1.00 * L-isol	SLC7A5	Transport, extracellular			0
r1660_neg	9	0.080856	1.0922877	2.004175	2.004175	1	0.240222	r1660	Amino Acid-Polyamine-Organocation (APC) TCDB:2.A.3.8.1	1.00 * L-threonine [e] + 1.00 * L-homoserine [c] --> 1.00 *	SLC7A5	Transport, extracellular			0
r1660_pos	9	0.080856	1.0922877	2.004175	2.004175	1	0.240222	r1660	Amino Acid-Polyamine-Organocation (APC) TCDB:2.A.3.8.1	1.00 * L-threonine [e] + 1.00 * L-homoserine [c] --> 1.00 *	SLC7A5	Transport, extracellular			0
r1661_neg	9	0.080856	1.0922877	2.025473	2.025473	1	0.240222	r1661	Amino Acid-Polyamine-Organocation (APC) TCDB:2.A.3.8.1	1.00 * L-threonine [e] + 1.00 * L-isoleucine [c] --> 1.00 * L-	SLC7A5	Transport, extracellular			0
r1661_pos	9	0.080856	1.0922877	2.025473	2.025473	1	0.240222	r1661	Amino Acid-Polyamine-Organocation (APC) TCDB:2.A.3.8.1	1.00 * L-threonine [e] + 1.00 * L-isoleucine [c] --> 1.00 * L-	SLC7A5	Transport, extracellular			0
r1662_neg	9	0.080856	1.0922877	2.025473	2.025473	1	0.240222	r1662	Amino Acid-Polyamine-Organocation (APC) TCDB:2.A.3.8.1	1.00 * L-homoserine [e] + 1.00 * L-isoleucine [c] --> 1.00 *	SLC7A5	Transport, extracellular			0
r1662_pos	9	0.080856	1.0922877	2.025473	2.025473	1	0.240222	r1662	Amino Acid-Polyamine-Organocation (APC) TCDB:2.A.3.8.1	1.00 * L-homoserine [e] + 1.00 * L-isoleucine [c] --> 1.00 *	SLC7A5	Transport, extracellular			0
r1664_neg	9	0.080856	1.0922877	2.814368	2.814368	1	0.240222	r1664	Y+LAT2 Utilized transport	1.00 * Ornithine [c] + 1.00 * L-lysinium(1+) [e] --> 1.00 * L-	SLC7A6	Transport, extracellular			0
r1664_pos	9	0.080856	1.0922877	3.031531	3.031531	1	0.240222	r1664	Y+LAT2 Utilized transport	1.00 * Ornithine [c] + 1.00 * L-lysinium(1+) [e] --> 1.00 * L-	SLC7A6	Transport, extracellular			0
r1665_neg	9	0.080856	1.0922877	2.465985	2.465985	1	0.240222	r1665	Y+LAT2 Utilized transport	1.00 * L-lysinium(1+) [e] + 1.00 * L-histidine [c] --> 1.00 * L	SLC7A6	Transport, extracellular			0
r1665_pos	9	0.080856	1.0922877	2.577006	2.577006	1	0.240222	r1665	Y+LAT2 Utilized transport	1.00 * L-lysinium(1+) [e] + 1.00 * L-histidine [c] --> 1.00 * L	SLC7A6	Transport, extracellular			0
r1666_neg	9	0.080856	1.0922877	2.814368	2.814368	1	0.240222	r1666	Y+LAT2 Utilized transport	1.00 * L-lysinium(1+) [e] + 1.00 * L-citrulline [c] --> 1.00 *	SLC7A6	Transport, extracellular			0
r1666_pos	9	0.080856	1.0922877	3.024584	3.024584	1	0.240222	r1666	Y+LAT2 Utilized transport	1.00 * L-lysinium(1+) [e] + 1.00 * L-citrulline [c] --> 1.00 *	SLC7A6	Transport, extracellular			0
r1667_neg	9	0.080856	1.0922877	2.773087	2.773087	1	0.240222	r1667	Y+LAT2 Utilized transport	1.00 * Ornithine [c] + 1.00 * L-argininium(1+) [e] --> 1.00 *	SLC7A6	Transport, extracellular			0
r1667_pos	9	0.080856	1.0922877	2.604285	2.604285	1	0.240222	r1667	Y+LAT2 Utilized transport	1.00 * Ornithine [c] + 1.00 * L-argininium(1+) [e] --> 1.00 *	SLC7A6	Transport, extracellular			0
r1668_neg	9	0.080856	1.0922877	2.465985	2.465985	1	0.240222	r1668	Y+LAT2 Utilized transport	1.00 * L-argininium(1+) [e] + 1.00 * L-histidine [c] --> 1.00	SLC7A6	Transport, extracellular			0
r1668_pos	9	0.080856	1.0922877	2.508715	2.508715	1	0.240222	r1668	Y+LAT2 Utilized transport	1.00 * L-argininium(1+) [e] + 1.00 * L-histidine [c] --> 1.00	SLC7A6	Transport, extracellular			0
r1669_neg	9	0.080856	1.0922877	2.773087	2.773087	1	0.240222	r1669	Y+LAT2 Utilized transport	1.00 * L-argininium(1+) [e] + 1.00 * L-citrulline [c] --> 1.00	SLC7A6	Transport, extracellular			0
r1669_pos	9	0.080856	1.0922877	2.880757	2.880757	1	0.240222	r1669	Y+LAT2 Utilized transport	1.00 * L-argininium(1+) [e] + 1.00 * L-citrulline [c] --> 1.00	SLC7A6	Transport, extracellular			0
r1670_neg	9	0.080856	1.0922877	2.39885	2.39885	1	0.240222	r1670	Y+LAT2 Utilized transport	1.00 * Ornithine [e] + 1.00 * L-histidine [c] --> 1.00 * Ornit	SLC7A6	Transport, extracellular			0
r1670_pos	9	0.080856	1.0922877	2.812316	2.812316	1	0.240222	r1670	Y+LAT2 Utilized transport	1.00 * Ornithine [e] + 1.00 * L-histidine [c] --> 1.00 * Ornit	SLC7A6	Transport, extracellular			0
r1671_neg	9	0.080856	1.0922877	2.812316	2.812316	1	0.240222	r1671	Y+LAT2 Utilized transport	1.00 * L-citrulline [c] + 1.00 * L-histidine [e] --> 1.00 * L-hi	SLC7A6	Transport, extracellular			0
r1671_pos	9	0.080856	1.0922877	2.227251	2.227251	1	0.240222	r1671	Y+LAT2 Utilized transport	1.00 * L-citrulline [c] + 1.00 * L-histidine [e] --> 1.00 * L-hi	SLC7A6	Transport, extracellular			0
r1672_pos	9	0.080856	1.0922877	2.3904	2.3904	1	0.240222	r1672	Y+LAT2 Utilized transport	1.00 * Sodium [e] + 1.00 * L-alanine [c] + 1.00 * Glycine [e]	SLC7A6	Transport, extracellular			0
r1673_pos	9	0.080856	1.0922877	4.144266	4.144266	1	0.240222	r1673	Y+LAT2 Utilized transport	1.00 * Sodium [e] + 1.00 * L-glutamine [c] + 1.00 * Glycine	SLC7A6	Transport, extracellular			0
r1674_pos	9	0.080856	1.0922877	2.3904	2.3904	1	0.240222	r1674	Y+LAT2 Utilized transport	1.00 * Sodium [e] + 1.00 * Glycine [e] + 1.00 * L-serine [c] -	SLC7A6	Transport, extracellular			0
r1675_pos	9	0.080856	1.0922877	4.144266	4.144266	1	0.240222	r1675	Y+LAT2 Utilized transport	1.00 * Sodium [e] + 1.00 * Glycine [e] + 1.00 * L-methionin	SLC7A6	Transport, extracellular			0
r1676_pos	9	0.080856	1.0922877	4.144266	4.144266	1	0.240222	r1676	Y+LAT2 Utilized transport	1.00 * Sodium [e] + 1.00 * Glycine [e] + 1.00 * L-tryptopha	SLC7A6	Transport, extracellular			0
r1677_pos	9	0.080856	1.0922877	4.144266	4.144266	1	0.240222	r1677	Y+LAT2 Utilized transport	1.00 * Sodium [e] + 1.00 * Glycine [e] + 1.00 * L-phenylala	SLC7A6	Transport, extracellular			0
r1678_pos	9	0.080856	1.0922877	4.144266	4.144266	1	0.240222	r1678	Y+LAT2 Utilized transport	1.00 * Sodium [e] + 1.00 * Glycine [e] + 1.00 * L-tyrosine [	SLC7A6	Transport, extracellular			0
r1679_pos	9	0.080856	1.0922877	2.459877	2.459877	1	0.240222	r1679	Y+LAT2 Utilized transport	1.00 * Sodium [e] + 1.00 * L-cysteine [c] + 1.00 * Glycine [	SLC7A6	Transport, extracellular			0
r1680_pos	9	0.080856	1.0922877	4.144266	4.144266	1	0.240222	r1680	Y+LAT2 Utilized transport	1.00 * Sodium [e] + 1.00 * Glycine [e] + 1.00 * L-leucine [c]	SLC7A6	Transport, extracellular			0
r1681_pos	9	0.080856	1.0922877	4.144266	4.144266	1	0.240222	r1681	Y+LAT2 Utilized transport	1.00 * Sodium [e] + 1.00 * Glycine [e] + 1.00 * L-proline [c]	SLC7A6	Transport, extracellular			0
r1682_pos	9	0.080856	1.0922877	4.144266	4.144266	1	0.240222	r1682	Y+LAT2 Utilized transport	1.00 * Sodium [e] + 1.00 * L-asparagine [c] + 1.00 * Glycin	SLC7A6	Transport, extracellular			0
r1683_pos	9	0.080856	1.0922877	4.144266	4.144266	1	0.240222	r1683	Y+LAT2 Utilized transport	1.00 * Sodium [e] + 1.00 * Glycine [e] + 1.00 * L-valine [c] -	SLC7A6	Transport, extracellular			0
r1684_pos	9	0.080856	1.0922877	2.3904	2.3904	1	0.240222	r1684	Y+LAT2 Utilized transport	1.00 * Sodium [e] + 1.00 * Glycine [e] + 1.00 * L-threonine	SLC7A6	Transport, extracellular			0
r1685_pos	9	0.080856	1.0922877	4.144266	4.144266	1	0.240222	r1685	Y+LAT2 Utilized transport	1.00 * Sodium [e] + 1.00 * Glycine [e] + 1.00 * L-homoseri	SLC7A6	Transport, extracellular			0
r1686_pos	9	0.080856	1.0922877	4.144266	4.144266	1	0.240222	r1686	Y+LAT2 Utilized transport	1.00 * Sodium [e] + 1.00 * Glycine [e] + 1.00 * L-isoleucine	SLC7A6	Transport, extracellular			0
r1687_pos	9	0.080856	1.0922877	2.3904	2.3904	1	0.240222	r1687	Y+LAT2 Utilized transport	1.00 * Sodium [e] + 1.00 * L-alanine [e] + 1.00 * Glycine [c]	SLC7A6	Transport, extracellular			0
r1690_pos	9	0.080856	1.0922877	2.834972	2.834972	1	0.240222	r1690	Y+LAT2 Utilized transport	1.00 * Sodium [e] + 1.00 * L-alanine [e] + 1.00 * L-methion	SLC7A6	Transport, extracellular			0
r1691_pos	9	0.080856	1.0922877	3.009969	3.009969	1	0.240222	r1691	Y+LAT2 Utilized transport	1.00 * Sodium [e] + 1.00 * L-alanine [e] + 1.00 * L-tryptoph	SLC7A6	Transport, extracellular			0
r1692_pos	9	0.080856	1.0922877	2.822181	2.822181	1	0.240222	r1692	Y+LAT2 Utilized transport	1.00 * Sodium [e] + 1.00 * L-alanine [e] + 1.00 * L-phenylal	SLC7A6	Transport, extracellular			0
r1693_pos	9	0.080856	1.0922877	3.131513	3.131513	1	0.240222	r1693	Y+LAT2 Utilized transport	1.00 * Sodium [e] + 1.00 * L-alanine [e] + 1.00 * L-tyrosine	SLC7A6	Transport, extracellular			0
r1695_pos	9	0.080856	1.0922877	2.834972	2.834972	1	0.240222	r1695	Y+LAT2 Utilized transport	1.00 * Sodium [e] + 1.00 * L-alanine [e] + 1.00 * L-leucine [	SLC7A6	Transport, extracellular			0
r1696_pos	9	0.080856	1.0922877	3.361208	3.361208	1	0.240222	r1696	Y+LAT2 Utilized transport	1.00 * Sodium [e] + 1.00 * L-alanine [e] + 1.00 * L-proline [	SLC7A6	Transport, extracellular			0
r1698_pos	9	0.080856	1.0922877	2.834972	2.834972	1	0.240222	r1698	Y+LAT2 Utilized transport	1.00 * Sodium [e] + 1.00 * L-alanine [e] + 1.00 * L-valine [c	SLC7A6	Transport, extracellular			0
r1700_pos	9	0.080856	1.0922877	2.843614	2.843614	1	0.240222	r1700	Y+LAT2 Utilized transport	1.00 * Sodium [e] + 1.00 * L-alanine [e] + 1.00 * L-homose	SLC7A6	Transport, extracellular			0
r1701_pos	9	0.080856	1.0922877	2.834972	2.834972	1	0.240222	r1701	Y+LAT2 Utilized transport	1.00 * Sodium [e] + 1.00 * L-alanine [e] + 1.00 * L-isoleuci	SLC7A6	Transport, extracellular			0
r1702_pos	9	0.080856	1.0922877	3.61083	3.61083	1	0.240222	r1702	Y+LAT2 Utilized transport	1.00 * Sodium [e] + 1.00 * L-glutamine [e] + 1.00 * Glycine	SLC7A6	Transport, extracellular			0
r1705_pos	9	0.080856	1.0922877	3.272606	3.272606	1	0.240222	r1705	Y+LAT2 Utilized transport	1.00 * Sodium [e] + 1.00 * L-glutamine [e] + 1.00 * L-meth	SLC7A6	Transport, extracellular			0
r1706_pos	9	0.080856	1.0922877	3.329088	3.329088	1	0.240222	r1706	Y+LAT2 Utilized transport	1.00 * Sodium [e] + 1.00 * L-glutamine [e] + 1.00 * L-trypt	SLC7A6	Transport, extracellular			0
r1707_pos	9	0.080856	1.0922877	3.296926	3.296926	1	0.240222	r1707	Y+LAT2 Utilized transport	1.00 * Sodium [e] + 1.00 * L-glutamine [e] + 1.00 * L-phen	SLC7A6	Transport, extracellular			0
r1708_pos	9	0.080856	1.0922877	3.374619	3.374619	1	0.240222	r1708	Y+LAT2 Utilized transport	1.00 * Sodium [e] + 1.00 * L-glutamine [e] + 1.00 * L-tyros	SLC7A6	Transport, extracellular			0
r1710_pos	9	0.080856	1.0922877	3.272606	3.272606	1	0.240222	r1710	Y+LAT2 Utilized transport	1.00 * Sodium [e] + 1.00 * L-glutamine [e] + 1.00 * L-leuci	SLC7A6	Transport, extracellular			0
r1711_pos	9	0.080856	1.0922877	3.361208	3.361208	1	0.240222	r1711	Y+LAT2 Utilized transport	1.00 * Sodium [e] + 1.00 * L-glutamine [e] + 1.00 * L-proli	SLC7A6	Transport, extracellular			0
r1713_pos	9	0.080856	1.0922877	3.272606	3.272606	1	0.240222	r1713	Y+LAT2 Utilized transport	1.00 * Sodium [e] + 1.00 * L-glutamine [e] + 1.00 * L-valine	SLC7A6	Transport, extracellular			0
r1715_pos	9	0.080856	1.0922877	3.276495	3.276495	1	0.240222	r1715	Y+LAT2 Utilized transport	1.00 * Sodium [e] + 1.00 * L-glutamine [e] + 1.00 * L-homo	SLC7A6	Transport, extracellular			0
r1716_pos	9	0.080856	1.0922877	3.272606	3.272606	1	0.240222	r1716	Y+LAT2 Utilized transport	1.00 * Sodium [e] + 1.00 * L-glutamine [e] + 1.00 * L-isoleu	SLC7A6	Transport, extracellular			0
r1717_pos	9	0.080856	1.0922877	2.3904	2.3904	1	0.240222	r1717	Y+LAT2 Utilized transport	1.00 * Sodium [e] + 1.00 * Glycine [c] + 1.00 * L-serine [e] -	SLC7A6	Transport, extracellular			0
r1720_pos	9	0.080856	1.0922877	4.144266	4.144266	1	0.240222	r1720	Y+LAT2 Utilized transport	1.00 * Sodium [e] + 1.00 * L-serine [e] + 1.00 * L-methioni	SLC7A6	Transport, extracellular			0
r1721_pos	9	0.080856	1.0922877	4.144266	4.144266	1	0.240222	r1721	Y+LAT2 Utilized transport	1.00 * Sodium [e] + 1.00 * L-serine [e] + 1.00 * L-tryptopha	SLC7A6	Transport, extracellular			0
r1722_pos	9	0.080856	1.0922877	4.144266	4.144266	1	0.240222	r1722	Y+LAT2 Utilized transport	1.00 * Sodium [e] + 1.00 * L-serine [e] + 1.00 * L-phenylala	SLC7A6	Transport, extracellular			0
r1723_pos	9	0.080856	1.0922877	4.144266	4.144266	1	0.240222	r1723	Y+LAT2 Utilized transport	1.00 * Sodium [e] + 1.00 * L-serine [e] + 1.00 * L-tyrosine [	SLC7A6	Transport, extracellular			0
r1725_pos	9	0.080856	1.0922877	4.144266	4.144266	1	0.240222	r1725	Y+LAT2 Utilized transport	1.00 * Sodium [e] + 1.00 * L-serine [e] + 1.00 * L-leucine [c	SLC7A6	Transport, extracellular			0

r1726_pos	9	0.080856	1.0922877	4.144266	4.144266	1	0.240222	r1726	Y+LAT2 Utilized transport	1.00 * Sodium [e] + 1.00 * L-serine [e] + 1.00 * L-proline [c	SLC7A6	Transport, extracellular			0
r1728_pos	9	0.080856	1.0922877	4.144266	4.144266	1	0.240222	r1728	Y+LAT2 Utilized transport	1.00 * Sodium [e] + 1.00 * L-serine [e] + 1.00 * L-valine [c]	SLC7A6	Transport, extracellular			0
r1730_pos	9	0.080856	1.0922877	4.144266	4.144266	1	0.240222	r1730	Y+LAT2 Utilized transport	1.00 * Sodium [e] + 1.00 * L-serine [e] + 1.00 * L-homoseri	SLC7A6	Transport, extracellular			0
r1731_pos	9	0.080856	1.0922877	4.144266	4.144266	1	0.240222	r1731	Y+LAT2 Utilized transport	1.00 * Sodium [e] + 1.00 * L-serine [e] + 1.00 * L-isoleucine	SLC7A6	Transport, extracellular			0
r1732_pos	9	0.080856	1.0922877	3.37221	3.37221	1	0.240222	r1732	Y+LAT2 Utilized transport	1.00 * Sodium [e] + 1.00 * Glycine [c] + 1.00 * L-methionin	SLC7A6	Transport, extracellular			0
r1733_pos	9	0.080856	1.0922877	2.743461	2.743461	1	0.240222	r1733	Y+LAT2 Utilized transport	1.00 * Sodium [e] + 1.00 * L-alanine [c] + 1.00 * L-methion	SLC7A6	Transport, extracellular			0
r1734_pos	9	0.080856	1.0922877	2.912398	2.912398	1	0.240222	r1734	Y+LAT2 Utilized transport	1.00 * Sodium [e] + 1.00 * L-glutamine [c] + 1.00 * L-meth	SLC7A6	Transport, extracellular			0
r1735_pos	9	0.080856	1.0922877	2.912398	2.912398	1	0.240222	r1735	Y+LAT2 Utilized transport	1.00 * Sodium [e] + 1.00 * L-serine [c] + 1.00 * L-methioni	SLC7A6	Transport, extracellular			0
r1736_pos	9	0.080856	1.0922877	2.99593	2.99593	1	0.240222	r1736	Y+LAT2 Utilized transport	1.00 * Sodium [e] + 1.00 * L-methionine [e] + 1.00 * L-tryp	SLC7A6	Transport, extracellular			0
r1737_pos	9	0.080856	1.0922877	2.949743	2.949743	1	0.240222	r1737	Y+LAT2 Utilized transport	1.00 * Sodium [e] + 1.00 * L-methionine [e] + 1.00 * L-phe	SLC7A6	Transport, extracellular			0
r1738_pos	9	0.080856	1.0922877	2.97248	2.97248	1	0.240222	r1738	Y+LAT2 Utilized transport	1.00 * Sodium [e] + 1.00 * L-methionine [e] + 1.00 * L-tyro	SLC7A6	Transport, extracellular			0
r1739_pos	9	0.080856	1.0922877	3.442199	3.442199	1	0.240222	r1739	Y+LAT2 Utilized transport	1.00 * Sodium [e] + 1.00 * L-cysteine [c] + 1.00 * L-methio	SLC7A6	Transport, extracellular			0
r1740_pos	9	0.080856	1.0922877	2.907355	2.907355	1	0.240222	r1740	Y+LAT2 Utilized transport	1.00 * Sodium [e] + 1.00 * L-methionine [e] + 1.00 * L-leuc	SLC7A6	Transport, extracellular			0
r1741_pos	9	0.080856	1.0922877	2.743461	2.743461	1	0.240222	r1741	Y+LAT2 Utilized transport	1.00 * Sodium [e] + 1.00 * L-methionine [e] + 1.00 * L-prol	SLC7A6	Transport, extracellular			0
r1742_pos	9	0.080856	1.0922877	2.912398	2.912398	1	0.240222	r1742	Y+LAT2 Utilized transport	1.00 * Sodium [e] + 1.00 * L-asparagine [c] + 1.00 * L-meth	SLC7A6	Transport, extracellular			0
r1743_pos	9	0.080856	1.0922877	2.907355	2.907355	1	0.240222	r1743	Y+LAT2 Utilized transport	1.00 * Sodium [e] + 1.00 * L-methionine [e] + 1.00 * L-vali	SLC7A6	Transport, extracellular			0
r1744_pos	9	0.080856	1.0922877	2.912398	2.912398	1	0.240222	r1744	Y+LAT2 Utilized transport	1.00 * Sodium [e] + 1.00 * L-threonine [c] + 1.00 * L-meth	SLC7A6	Transport, extracellular			0
r1745_pos	9	0.080856	1.0922877	2.912398	2.912398	1	0.240222	r1745	Y+LAT2 Utilized transport	1.00 * Sodium [e] + 1.00 * L-methionine [e] + 1.00 * L-hom	SLC7A6	Transport, extracellular			0
r1746_pos	9	0.080856	1.0922877	2.907355	2.907355	1	0.240222	r1746	Y+LAT2 Utilized transport	1.00 * Sodium [e] + 1.00 * L-methionine [e] + 1.00 * L-isol	SLC7A6	Transport, extracellular			0
r1747_pos	9	0.080856	1.0922877	4.144266	4.144266	1	0.240222	r1747	Y+LAT2 Utilized transport	1.00 * Sodium [e] + 1.00 * Glycine [c] + 1.00 * L-tryptopha	SLC7A6	Transport, extracellular			0
r1748_pos	9	0.080856	1.0922877	4.144266	4.144266	1	0.240222	r1748	Y+LAT2 Utilized transport	1.00 * Sodium [e] + 1.00 * L-alanine [c] + 1.00 * L-tryptoph	SLC7A6	Transport, extracellular			0
r1749_pos	9	0.080856	1.0922877	4.144266	4.144266	1	0.240222	r1749	Y+LAT2 Utilized transport	1.00 * Sodium [e] + 1.00 * L-glutamine [c] + 1.00 * L-trypt	SLC7A6	Transport, extracellular			0
r1750_pos	9	0.080856	1.0922877	4.144266	4.144266	1	0.240222	r1750	Y+LAT2 Utilized transport	1.00 * Sodium [e] + 1.00 * L-serine [c] + 1.00 * L-tryptopha	SLC7A6	Transport, extracellular			0
r1751_pos	9	0.080856	1.0922877	4.144266	4.144266	1	0.240222	r1751	Y+LAT2 Utilized transport	1.00 * Sodium [e] + 1.00 * L-methionine [c] + 1.00 * L-tryp	SLC7A6	Transport, extracellular			0
r1752_pos	9	0.080856	1.0922877	4.144266	4.144266	1	0.240222	r1752	Y+LAT2 Utilized transport	1.00 * Sodium [e] + 1.00 * L-tryptophan [e] + 1.00 * L-phe	SLC7A6	Transport, extracellular			0
r1753_pos	9	0.080856	1.0922877	4.144266	4.144266	1	0.240222	r1753	Y+LAT2 Utilized transport	1.00 * Sodium [e] + 1.00 * L-tryptophan [e] + 1.00 * L-tyro	SLC7A6	Transport, extracellular			0
r1754_pos	9	0.080856	1.0922877	4.144266	4.144266	1	0.240222	r1754	Y+LAT2 Utilized transport	1.00 * Sodium [e] + 1.00 * L-cysteine [c] + 1.00 * L-tryptop	SLC7A6	Transport, extracellular			0
r1755_pos	9	0.080856	1.0922877	4.144266	4.144266	1	0.240222	r1755	Y+LAT2 Utilized transport	1.00 * Sodium [e] + 1.00 * L-tryptophan [e] + 1.00 * L-leuc	SLC7A6	Transport, extracellular			0
r1756_pos	9	0.080856	1.0922877	2.854938	2.854938	1	0.240222	r1756	Y+LAT2 Utilized transport	1.00 * Sodium [e] + 1.00 * L-tryptophan [e] + 1.00 * L-prol	SLC7A6	Transport, extracellular			0
r1757_pos	9	0.080856	1.0922877	2.854938	2.854938	1	0.240222	r1757	Y+LAT2 Utilized transport	1.00 * Sodium [e] + 1.00 * L-asparagine [c] + 1.00 * L-trypt	SLC7A6	Transport, extracellular			0
r1758_pos	9	0.080856	1.0922877	2.854938	2.854938	1	0.240222	r1758	Y+LAT2 Utilized transport	1.00 * Sodium [e] + 1.00 * L-tryptophan [e] + 1.00 * L-vali	SLC7A6	Transport, extracellular			0
r1759_pos	9	0.080856	1.0922877	2.854938	2.854938	1	0.240222	r1759	Y+LAT2 Utilized transport	1.00 * Sodium [e] + 1.00 * L-threonine [c] + 1.00 * L-trypto	SLC7A6	Transport, extracellular			0
r1760_pos	9	0.080856	1.0922877	2.854938	2.854938	1	0.240222	r1760	Y+LAT2 Utilized transport	1.00 * Sodium [e] + 1.00 * L-tryptophan [e] + 1.00 * L-hom	SLC7A6	Transport, extracellular			0
r1761_pos	9	0.080856	1.0922877	2.854938	2.854938	1	0.240222	r1761	Y+LAT2 Utilized transport	1.00 * Sodium [e] + 1.00 * L-tryptophan [e] + 1.00 * L-isole	SLC7A6	Transport, extracellular			0
r1762_pos	9	0.080856	1.0922877	4.144266	4.144266	1	0.240222	r1762	Y+LAT2 Utilized transport	1.00 * Sodium [e] + 1.00 * Glycine [c] + 1.00 * L-phenylala	SLC7A6	Transport, extracellular			0
r1763_pos	9	0.080856	1.0922877	4.144266	4.144266	1	0.240222	r1763	Y+LAT2 Utilized transport	1.00 * Sodium [e] + 1.00 * L-alanine [c] + 1.00 * L-phenylal	SLC7A6	Transport, extracellular			0
r1764_pos	9	0.080856	1.0922877	4.144266	4.144266	1	0.240222	r1764	Y+LAT2 Utilized transport	1.00 * Sodium [e] + 1.00 * L-glutamine [c] + 1.00 * L-pheny	SLC7A6	Transport, extracellular			0
r1765_pos	9	0.080856	1.0922877	4.144266	4.144266	1	0.240222	r1765	Y+LAT2 Utilized transport	1.00 * Sodium [e] + 1.00 * L-serine [c] + 1.00 * L-phenylala	SLC7A6	Transport, extracellular			0
r1766_pos	9	0.080856	1.0922877	4.144266	4.144266	1	0.240222	r1766	Y+LAT2 Utilized transport	1.00 * Sodium [e] + 1.00 * L-methionine [c] + 1.00 * L-phe	SLC7A6	Transport, extracellular			0
r1767_pos	9	0.080856	1.0922877	4.144266	4.144266	1	0.240222	r1767	Y+LAT2 Utilized transport	1.00 * Sodium [e] + 1.00 * L-phenylalanine [e] + 1.00 * L-tr	SLC7A6	Transport, extracellular			0
r1768_pos	9	0.080856	1.0922877	3.054027	3.054027	1	0.240222	r1768	Y+LAT2 Utilized transport	1.00 * Sodium [e] + 1.00 * L-phenylalanine [e] + 1.00 * L-ty	SLC7A6	Transport, extracellular			0
r1769_pos	9	0.080856	1.0922877	3.515568	3.515568	1	0.240222	r1769	Y+LAT2 Utilized transport	1.00 * Sodium [e] + 1.00 * L-cysteine [c] + 1.00 * L-phenyla	SLC7A6	Transport, extracellular			0
r1770_pos	9	0.080856	1.0922877	2.97239	2.97239	1	0.240222	r1770	Y+LAT2 Utilized transport	1.00 * Sodium [e] + 1.00 * L-phenylalanine [e] + 1.00 * L-le	SLC7A6	Transport, extracellular			0
r1771_pos	9	0.080856	1.0922877	2.854938	2.854938	1	0.240222	r1771	Y+LAT2 Utilized transport	1.00 * Sodium [e] + 1.00 * L-phenylalanine [e] + 1.00 * L-p	SLC7A6	Transport, extracellular			0
r1772_pos	9	0.080856	1.0922877	2.3704	2.3704	1	0.240222	r1772	Y+LAT2 Utilized transport	1.00 * Sodium [e] + 1.00 * L-asparagine [c] + 1.00 * L-phen	SLC7A6	Transport, extracellular			0
r1773_pos	9	0.080856	1.0922877	2.97239	2.97239	1	0.240222	r1773	Y+LAT2 Utilized transport	1.00 * Sodium [e] + 1.00 * L-phenylalanine [e] + 1.00 * L-va	SLC7A6	Transport, extracellular			0
r1774_pos	9	0.080856	1.0922877	2.978131	2.978131	1	0.240222	r1774	Y+LAT2 Utilized transport	1.00 * Sodium [e] + 1.00 * L-threonine [c] + 1.00 * L-pheny	SLC7A6	Transport, extracellular			0
r1775_pos	9	0.080856	1.0922877	2.854938	2.854938	1	0.240222	r1775	Y+LAT2 Utilized transport	1.00 * Sodium [e] + 1.00 * L-phenylalanine [e] + 1.00 * L-h	SLC7A6	Transport, extracellular			0
r1776_pos	9	0.080856	1.0922877	2.97239	2.97239	1	0.240222	r1776	Y+LAT2 Utilized transport	1.00 * Sodium [e] + 1.00 * L-phenylalanine [e] + 1.00 * L-is	SLC7A6	Transport, extracellular			0
r1777_pos	9	0.080856	1.0922877	4.144266	4.144266	1	0.240222	r1777	Y+LAT2 Utilized transport	1.00 * Sodium [e] + 1.00 * Glycine [c] + 1.00 * L-tyrosine [e	SLC7A6	Transport, extracellular			0
r1778_pos	9	0.080856	1.0922877	4.144266	4.144266	1	0.240222	r1778	Y+LAT2 Utilized transport	1.00 * Sodium [e] + 1.00 * L-alanine [c] + 1.00 * L-tyrosine	SLC7A6	Transport, extracellular			0
r1779_pos	9	0.080856	1.0922877	4.144266	4.144266	1	0.240222	r1779	Y+LAT2 Utilized transport	1.00 * Sodium [e] + 1.00 * L-glutamine [c] + 1.00 * L-tyrosi	SLC7A6	Transport, extracellular			0
r1780_pos	9	0.080856	1.0922877	4.144266	4.144266	1	0.240222	r1780	Y+LAT2 Utilized transport	1.00 * Sodium [e] + 1.00 * L-serine [c] + 1.00 * L-tyrosine [	SLC7A6	Transport, extracellular			0
r1781_pos	9	0.080856	1.0922877	4.144266	4.144266	1	0.240222	r1781	Y+LAT2 Utilized transport	1.00 * Sodium [e] + 1.00 * L-methionine [c] + 1.00 * L-tyro	SLC7A6	Transport, extracellular			0
r1782_pos	9	0.080856	1.0922877	4.144266	4.144266	1	0.240222	r1782	Y+LAT2 Utilized transport	1.00 * Sodium [e] + 1.00 * L-tyrosine [e] + 1.00 * L-tryptop	SLC7A6	Transport, extracellular			0
r1783_pos	9	0.080856	1.0922877	3.546102	3.546102	1	0.240222	r1783	Y+LAT2 Utilized transport	1.00 * Sodium [e] + 1.00 * L-tyrosine [e] + 1.00 * L-phenyla	SLC7A6	Transport, extracellular			0
r1784_pos	9	0.080856	1.0922877	4.144266	4.144266	1	0.240222	r1784	Y+LAT2 Utilized transport	1.00 * Sodium [e] + 1.00 * L-cysteine [c] + 1.00 * L-tyrosine	SLC7A6	Transport, extracellular			0
r1785_pos	9	0.080856	1.0922877	4.144266	4.144266	1	0.240222	r1785	Y+LAT2 Utilized transport	1.00 * Sodium [e] + 1.00 * L-tyrosine [e] + 1.00 * L-leucine	SLC7A6	Transport, extracellular			0
r1786_pos	9	0.080856	1.0922877	4.144266	4.144266	1	0.240222	r1786	Y+LAT2 Utilized transport	1.00 * Sodium [e] + 1.00 * L-tyrosine [e] + 1.00 * L-proline	SLC7A6	Transport, extracellular			0
r1787_pos	9	0.080856	1.0922877	4.144266	4.144266	1	0.240222	r1787	Y+LAT2 Utilized transport	1.00 * Sodium [e] + 1.00 * L-asparagine [c] + 1.00 * L-tyros	SLC7A6	Transport, extracellular			0
r1788_pos	9	0.080856	1.0922877	4.144266	4.144266	1	0.240222	r1788	Y+LAT2 Utilized transport	1.00 * Sodium [e] + 1.00 * L-tyrosine [e] + 1.00 * L-valine [	SLC7A6	Transport, extracellular			0
r1789_pos	9	0.080856	1.0922877	2.854938	2.854938	1	0.240222	r1789	Y+LAT2 Utilized transport	1.00 * Sodium [e] + 1.00 * L-threonine [c] + 1.00 * L-tyrosi	SLC7A6	Transport, extracellular			0
r1790_pos	9	0.080856	1.0922877	4.144266	4.144266	1	0.240222	r1790	Y+LAT2 Utilized transport	1.00 * Sodium [e] + 1.00 * L-tyrosine [e] + 1.00 * L-homose	SLC7A6	Transport, extracellular			0
r1791_pos	9	0.080856	1.0922877	4.144266	4.144266	1	0.240222	r1791	Y+LAT2 Utilized transport	1.00 * Sodium [e] + 1.00 * L-tyrosine [e] + 1.00 * L-isoleuc	SLC7A6	Transport, extracellular			0
r1792_pos	9	0.080856	1.0922877	2.459877	2.459877	1	0.240222	r1792	Y+LAT2 Utilized transport	1.00 * Sodium [e] + 1.00 * L-cysteine [e] + 1.00 * Glycine [	SLC7A6	Transport, extracellular			0
r1796_pos	9	0.080856	1.0922877	2.907355	2.907355	1	0.240222	r1796	Y+LAT2 Utilized transport	1.00 * Sodium [e] + 1.00 * L-cysteine [e] + 1.00 * L-methio	SLC7A6	Transport, extracellular			0
r1797_pos	9	0.080856	1.0922877	2.99593	2.99593	1	0.240222	r1797	Y+LAT2 Utilized transport	1.00 * Sodium [e] + 1.00 * L-cysteine [e] + 1.00 * L-tryptop	SLC7A6	Transport, extracellular			0
r1798_pos	9	0.080856	1.0922877	2.949743	2.949743	1	0.240222	r1798	Y+LAT2 Utilized transport	1.00 * Sodium [e] + 1.00 * L-cysteine [e] + 1.00 * L-phenyla	SLC7A6	Transport, extracellular			0
r1799_pos	9	0.080856	1.0922877	2.97248	2.97248	1	0.240222	r1799	Y+LAT2 Utilized transport	1.00 * Sodium [e] + 1.00 * L-cysteine [e] + 1.00 * L-tyrosine	SLC7A6	Transport, extracellular			0
r1800_pos	9	0.080856	1.0922877	2.907355	2.907355	1	0.240222	r1800	Y+LAT2 Utilized transport	1.00 * Sodium [e] + 1.00 * L-cysteine [e] + 1.00 * L-leucine	SLC7A6	Transport, extracellular			0
r1801_pos	9	0.080856	1.0922877	2.743461	2.743461	1	0.240222	r1801	Y+LAT2 Utilized transport	1.00 * Sodium [e] + 1.00 * L-cysteine [e] + 1.00 * L-proline	SLC7A6	Transport, extracellular			0
r1803_pos	9	0.080856	1.0922877	2.907355	2.907355	1	0.240222	r1803	Y+LAT2 Utilized transport	1.00 * Sodium [e] + 1.00 * L-cysteine [e] + 1.00 * L-valine [	SLC7A6	Transport, extracellular			0
r1805_pos	9	0.080856	1.0922877	2.912398	2.912398	1	0.240222	r1805	Y+LAT2 Utilized transport	1.00 * Sodium [e] + 1.00 * L-cysteine [e] + 1.00 * L-homose	SLC7A6	Transport, extracellular			0
r1806_pos	9	0.080856	1.0922877	2.907355	2.907355	1	0.240222	r1806	Y+LAT2 Utilized transport	1.00 * Sodium [e] + 1.00 * L-cysteine [e] + 1.00 * L-isoleuc	SLC7A6	Transport, extracellular			0
r1807_pos	9	0.080856	1.0922877	4.144266	4.144266	1	0.240222	r1807	Y+LAT2 Utilized transport	1.00 * Sodium [e] + 1.00 * Glycine [c] + 1.00 * L-leucine [e	SLC7A6	Transport, extracellular			0
r1808_pos	9	0.080856	1.0922877	4.144266	4.144266	1	0.240222	r1808	Y+LAT2 Utilized transport	1.00 * Sodium [e] + 1.00 * L-alanine [c] + 1.00 * L-leucine [	SLC7A6	Transport, extracellular			0
r1809_pos	9	0.080856	1.0922877	4.144266	4.144266	1	0.240222	r1809	Y+LAT2 Utilized transport	1.00 * Sodium [e] + 1.00 * L-glutamine [c] + 1.00 * L-leucin	SLC7A6	Transport, extracellular			0
r1810_pos	9	0.080856	1.0922877	4.144266	4.144266	1	0.240222	r1810	Y+LAT2 Utilized transport	1.00 * Sodium [e] + 1.00 * L-serine [c] + 1.00 * L-leucine [e	SLC7A6	Transport, extracellular			0
r1811_pos	9	0.080856	1.0922877	2.854938	2.854938	1	0.240222	r1811	Y+LAT2 Utilized transport	1.00 * Sodium [e] + 1.00 * L-methionine [c] + 1.00 * L-leuc	SLC7A6	Transport, extracellular			0
r1812_pos	9	0.080856	1.0922877	4.144266	4.144266	1	0.240222	r1812	Y+LAT2 Utilized transport	1.00 * Sodium [e] + 1.00 * L-leucine [e] + 1.00 * L-tryptoph	SLC7A6	Transport, extracellular			0
r1813_pos	9	0.080856	1.0922877	3.078795	3.078795	1	0.240222	r1813	Y+LAT2 Utilized transport	1.00 * Sodium [e] + 1.00 * L-leucine [e] + 1.00 * L-phenylal	SLC7A6	Transport, extracellular			0
r1814_pos	9	0.080856	1.0922877	4.144266	4.144266	1	0.240222	r1814	Y+LAT2 Utilized transport	1.00 * Sodium [e] + 1.00 * L-leucine [e] + 1.00 * L-tyrosine	SLC7A6	Transport, extracellular			0
r1815_pos	9	0.080856	1.0922877	4.144266	4.144266	1	0.240222	r1815	Y+LAT2 Utilized transport	1.00 * Sodium [e] + 1.00 * L-cysteine [c] + 1.00 * L-leucine	SLC7A6	Transport, extracellular			0

r1816_pos	9	0.080856	1.0922877	4.144266	4.144266	1	0.240222	r1816	Y+LAT2 Utilized transport	1.00 * Sodium [e] + 1.00 * L-leucine [e] + 1.00 * L-proline [	SLC7A6	Transport, extracellular			0
r1817_pos	9	0.080856	1.0922877	4.144266	4.144266	1	0.240222	r1817	Y+LAT2 Utilized transport	1.00 * Sodium [e] + 1.00 * L-asparagine [c] + 1.00 * L-leuci	SLC7A6	Transport, extracellular			0
r1818_pos	9	0.080856	1.0922877	3.032817	3.032817	1	0.240222	r1818	Y+LAT2 Utilized transport	1.00 * Sodium [e] + 1.00 * L-leucine [e] + 1.00 * L-valine [c	SLC7A6	Transport, extracellular			0
r1819_pos	9	0.080856	1.0922877	4.144266	4.144266	1	0.240222	r1819	Y+LAT2 Utilized transport	1.00 * Sodium [e] + 1.00 * L-threonine [c] + 1.00 * L-leucir	SLC7A6	Transport, extracellular			0
r1820_pos	9	0.080856	1.0922877	4.144266	4.144266	1	0.240222	r1820	Y+LAT2 Utilized transport	1.00 * Sodium [e] + 1.00 * L-leucine [e] + 1.00 * L-homose	SLC7A6	Transport, extracellular			0
r1821_pos	9	0.080856	1.0922877	3.032817	3.032817	1	0.240222	r1821	Y+LAT2 Utilized transport	1.00 * Sodium [e] + 1.00 * L-leucine [e] + 1.00 * L-isoleucir	SLC7A6	Transport, extracellular			0
r1822_pos	9	0.080856	1.0922877	4.144266	4.144266	1	0.240222	r1822	Y+LAT2 Utilized transport	1.00 * Sodium [e] + 1.00 * Glycine [c] + 1.00 * L-proline [e]	SLC7A6	Transport, extracellular			0
r1823_pos	9	0.080856	1.0922877	4.144266	4.144266	1	0.240222	r1823	Y+LAT2 Utilized transport	1.00 * Sodium [e] + 1.00 * L-alanine [c] + 1.00 * L-proline [	SLC7A6	Transport, extracellular			0
r1824_pos	9	0.080856	1.0922877	4.144266	4.144266	1	0.240222	r1824	Y+LAT2 Utilized transport	1.00 * Sodium [e] + 1.00 * L-glutamine [c] + 1.00 * L-prolir	SLC7A6	Transport, extracellular			0
r1825_pos	9	0.080856	1.0922877	4.144266	4.144266	1	0.240222	r1825	Y+LAT2 Utilized transport	1.00 * Sodium [e] + 1.00 * L-serine [c] + 1.00 * L-proline [e	SLC7A6	Transport, extracellular			0
r1826_pos	9	0.080856	1.0922877	4.144266	4.144266	1	0.240222	r1826	Y+LAT2 Utilized transport	1.00 * Sodium [e] + 1.00 * L-methionine [c] + 1.00 * L-prol	SLC7A6	Transport, extracellular			0
r1827_pos	9	0.080856	1.0922877	2.854938	2.854938	1	0.240222	r1827	Y+LAT2 Utilized transport	1.00 * Sodium [e] + 1.00 * L-proline [e] + 1.00 * L-tryptoph	SLC7A6	Transport, extracellular			0
r1828_pos	9	0.080856	1.0922877	2.854938	2.854938	1	0.240222	r1828	Y+LAT2 Utilized transport	1.00 * Sodium [e] + 1.00 * L-proline [e] + 1.00 * L-phenylal	SLC7A6	Transport, extracellular			0
r1829_pos	9	0.080856	1.0922877	4.144266	4.144266	1	0.240222	r1829	Y+LAT2 Utilized transport	1.00 * Sodium [e] + 1.00 * L-proline [e] + 1.00 * L-tyrosine	SLC7A6	Transport, extracellular			0
r1830_pos	9	0.080856	1.0922877	4.144266	4.144266	1	0.240222	r1830	Y+LAT2 Utilized transport	1.00 * Sodium [e] + 1.00 * L-cysteine [c] + 1.00 * L-proline	SLC7A6	Transport, extracellular			0
r1831_pos	9	0.080856	1.0922877	4.144266	4.144266	1	0.240222	r1831	Y+LAT2 Utilized transport	1.00 * Sodium [e] + 1.00 * L-proline [e] + 1.00 * L-leucine [	SLC7A6	Transport, extracellular			0
r1832_pos	9	0.080856	1.0922877	4.144266	4.144266	1	0.240222	r1832	Y+LAT2 Utilized transport	1.00 * Sodium [e] + 1.00 * L-asparagine [c] + 1.00 * L-proli	SLC7A6	Transport, extracellular			0
r1833_pos	9	0.080856	1.0922877	4.144266	4.144266	1	0.240222	r1833	Y+LAT2 Utilized transport	1.00 * Sodium [e] + 1.00 * L-proline [e] + 1.00 * L-valine [c	SLC7A6	Transport, extracellular			0
r1834_pos	9	0.080856	1.0922877	4.144266	4.144266	1	0.240222	r1834	Y+LAT2 Utilized transport	1.00 * Sodium [e] + 1.00 * L-threonine [c] + 1.00 * L-prolir	SLC7A6	Transport, extracellular			0
r1835_pos	9	0.080856	1.0922877	4.144266	4.144266	1	0.240222	r1835	Y+LAT2 Utilized transport	1.00 * Sodium [e] + 1.00 * L-proline [e] + 1.00 * L-homose	SLC7A6	Transport, extracellular			0
r1836_pos	9	0.080856	1.0922877	4.144266	4.144266	1	0.240222	r1836	Y+LAT2 Utilized transport	1.00 * Sodium [e] + 1.00 * L-proline [e] + 1.00 * L-isoleucir	SLC7A6	Transport, extracellular			0
r1837_pos	9	0.080856	1.0922877	3.61083	3.61083	1	0.240222	r1837	Y+LAT2 Utilized transport	1.00 * Sodium [e] + 1.00 * L-asparagine [e] + 1.00 * Glycin	SLC7A6	Transport, extracellular			0
r1841_pos	9	0.080856	1.0922877	3.272606	3.272606	1	0.240222	r1841	Y+LAT2 Utilized transport	1.00 * Sodium [e] + 1.00 * L-asparagine [e] + 1.00 * L-meth	SLC7A6	Transport, extracellular			0
r1842_pos	9	0.080856	1.0922877	2.724286	2.724286	1	0.240222	r1842	Y+LAT2 Utilized transport	1.00 * Sodium [e] + 1.00 * L-asparagine [e] + 1.00 * L-trypt	SLC7A6	Transport, extracellular			0
r1843_pos	9	0.080856	1.0922877	2.704224	2.704224	1	0.240222	r1843	Y+LAT2 Utilized transport	1.00 * Sodium [e] + 1.00 * L-asparagine [e] + 1.00 * L-phen	SLC7A6	Transport, extracellular			0
r1844_pos	9	0.080856	1.0922877	3.374619	3.374619	1	0.240222	r1844	Y+LAT2 Utilized transport	1.00 * Sodium [e] + 1.00 * L-asparagine [e] + 1.00 * L-tyros	SLC7A6	Transport, extracellular			0
r1846_pos	9	0.080856	1.0922877	3.272606	3.272606	1	0.240222	r1846	Y+LAT2 Utilized transport	1.00 * Sodium [e] + 1.00 * L-asparagine [e] + 1.00 * L-leuci	SLC7A6	Transport, extracellular			0
r1847_pos	9	0.080856	1.0922877	3.361208	3.361208	1	0.240222	r1847	Y+LAT2 Utilized transport	1.00 * Sodium [e] + 1.00 * L-asparagine [e] + 1.00 * L-proli	SLC7A6	Transport, extracellular			0
r1848_pos	9	0.080856	1.0922877	3.272606	3.272606	1	0.240222	r1848	Y+LAT2 Utilized transport	1.00 * Sodium [e] + 1.00 * L-asparagine [e] + 1.00 * L-valin	SLC7A6	Transport, extracellular			0
r1850_pos	9	0.080856	1.0922877	3.276495	3.276495	1	0.240222	r1850	Y+LAT2 Utilized transport	1.00 * Sodium [e] + 1.00 * L-asparagine [e] + 1.00 * L-hom	SLC7A6	Transport, extracellular			0
r1851_pos	9	0.080856	1.0922877	3.272606	3.272606	1	0.240222	r1851	Y+LAT2 Utilized transport	1.00 * Sodium [e] + 1.00 * L-asparagine [e] + 1.00 * L-isole	SLC7A6	Transport, extracellular			0
r1852_pos	9	0.080856	1.0922877	4.144266	4.144266	1	0.240222	r1852	Y+LAT2 Utilized transport	1.00 * Sodium [e] + 1.00 * Glycine [c] + 1.00 * L-valine [e] -	SLC7A6	Transport, extracellular			0
r1853_pos	9	0.080856	1.0922877	4.144266	4.144266	1	0.240222	r1853	Y+LAT2 Utilized transport	1.00 * Sodium [e] + 1.00 * L-alanine [c] + 1.00 * L-valine [e	SLC7A6	Transport, extracellular			0
r1854_pos	9	0.080856	1.0922877	4.144266	4.144266	1	0.240222	r1854	Y+LAT2 Utilized transport	1.00 * Sodium [e] + 1.00 * L-glutamine [c] + 1.00 * L-valine	SLC7A6	Transport, extracellular			0
r1855_pos	9	0.080856	1.0922877	4.144266	4.144266	1	0.240222	r1855	Y+LAT2 Utilized transport	1.00 * Sodium [e] + 1.00 * L-serine [c] + 1.00 * L-valine [e] -	SLC7A6	Transport, extracellular			0
r1856_pos	9	0.080856	1.0922877	4.144266	4.144266	1	0.240222	r1856	Y+LAT2 Utilized transport	1.00 * Sodium [e] + 1.00 * L-methionine [c] + 1.00 * L-vali	SLC7A6	Transport, extracellular			0
r1857_pos	9	0.080856	1.0922877	2.854938	2.854938	1	0.240222	r1857	Y+LAT2 Utilized transport	1.00 * Sodium [e] + 1.00 * L-valine [e] + 1.00 * L-tryptopha	SLC7A6	Transport, extracellular			0
r1858_pos	9	0.080856	1.0922877	3.080632	3.080632	1	0.240222	r1858	Y+LAT2 Utilized transport	1.00 * Sodium [e] + 1.00 * L-valine [e] + 1.00 * L-phenylala	SLC7A6	Transport, extracellular			0
r1859_pos	9	0.080856	1.0922877	4.144266	4.144266	1	0.240222	r1859	Y+LAT2 Utilized transport	1.00 * Sodium [e] + 1.00 * L-valine [e] + 1.00 * L-tyrosine [	SLC7A6	Transport, extracellular			0
r1860_pos	9	0.080856	1.0922877	4.144266	4.144266	1	0.240222	r1860	Y+LAT2 Utilized transport	1.00 * Sodium [e] + 1.00 * L-cysteine [c] + 1.00 * L-valine [	SLC7A6	Transport, extracellular			0
r1861_pos	9	0.080856	1.0922877	3.037857	3.037857	1	0.240222	r1861	Y+LAT2 Utilized transport	1.00 * Sodium [e] + 1.00 * L-valine [e] + 1.00 * L-leucine [c	SLC7A6	Transport, extracellular			0
r1862_pos	9	0.080856	1.0922877	4.144266	4.144266	1	0.240222	r1862	Y+LAT2 Utilized transport	1.00 * Sodium [e] + 1.00 * L-valine [e] + 1.00 * L-proline [c	SLC7A6	Transport, extracellular			0
r1863_pos	9	0.080856	1.0922877	4.144266	4.144266	1	0.240222	r1863	Y+LAT2 Utilized transport	1.00 * Sodium [e] + 1.00 * L-asparagine [c] + 1.00 * L-valin	SLC7A6	Transport, extracellular			0
r1864_pos	9	0.080856	1.0922877	4.144266	4.144266	1	0.240222	r1864	Y+LAT2 Utilized transport	1.00 * Sodium [e] + 1.00 * L-threonine [c] + 1.00 * L-valine	SLC7A6	Transport, extracellular			0
r1865_pos	9	0.080856	1.0922877	4.144266	4.144266	1	0.240222	r1865	Y+LAT2 Utilized transport	1.00 * Sodium [e] + 1.00 * L-valine [e] + 1.00 * L-homoseri	SLC7A6	Transport, extracellular			0
r1866_pos	9	0.080856	1.0922877	4.144266	4.144266	1	0.240222	r1866	Y+LAT2 Utilized transport	1.00 * Sodium [e] + 1.00 * L-valine [e] + 1.00 * L-isoleucine	SLC7A6	Transport, extracellular			0
r1867_pos	9	0.080856	1.0922877	2.3904	2.3904	1	0.240222	r1867	Y+LAT2 Utilized transport	1.00 * Sodium [e] + 1.00 * Glycine [c] + 1.00 * L-threonine	SLC7A6	Transport, extracellular			0
r1871_pos	9	0.080856	1.0922877	4.144266	4.144266	1	0.240222	r1871	Y+LAT2 Utilized transport	1.00 * Sodium [e] + 1.00 * L-threonine [e] + 1.00 * L-methi	SLC7A6	Transport, extracellular			0
r1872_pos	9	0.080856	1.0922877	2.854938	2.854938	1	0.240222	r1872	Y+LAT2 Utilized transport	1.00 * Sodium [e] + 1.00 * L-threonine [e] + 1.00 * L-trypto	SLC7A6	Transport, extracellular			0
r1873_pos	9	0.080856	1.0922877	3.094535	3.094535	1	0.240222	r1873	Y+LAT2 Utilized transport	1.00 * Sodium [e] + 1.00 * L-threonine [e] + 1.00 * L-pheny	SLC7A6	Transport, extracellular			0
r1874_pos	9	0.080856	1.0922877	2.854938	2.854938	1	0.240222	r1874	Y+LAT2 Utilized transport	1.00 * Sodium [e] + 1.00 * L-threonine [e] + 1.00 * L-tyrosi	SLC7A6	Transport, extracellular			0
r1876_pos	9	0.080856	1.0922877	4.144266	4.144266	1	0.240222	r1876	Y+LAT2 Utilized transport	1.00 * Sodium [e] + 1.00 * L-threonine [e] + 1.00 * L-leucir	SLC7A6	Transport, extracellular			0
r1877_pos	9	0.080856	1.0922877	4.144266	4.144266	1	0.240222	r1877	Y+LAT2 Utilized transport	1.00 * Sodium [e] + 1.00 * L-threonine [e] + 1.00 * L-prolir	SLC7A6	Transport, extracellular			0
r1879_pos	9	0.080856	1.0922877	4.144266	4.144266	1	0.240222	r1879	Y+LAT2 Utilized transport	1.00 * Sodium [e] + 1.00 * L-threonine [e] + 1.00 * L-valine	SLC7A6	Transport, extracellular			0
r1880_pos	9	0.080856	1.0922877	4.144266	4.144266	1	0.240222	r1880	Y+LAT2 Utilized transport	1.00 * Sodium [e] + 1.00 * L-threonine [e] + 1.00 * L-homo	SLC7A6	Transport, extracellular			0
r1881_pos	9	0.080856	1.0922877	4.144266	4.144266	1	0.240222	r1881	Y+LAT2 Utilized transport	1.00 * Sodium [e] + 1.00 * L-threonine [e] + 1.00 * L-isoleu	SLC7A6	Transport, extracellular			0
r1882_pos	9	0.080856	1.0922877	4.144266	4.144266	1	0.240222	r1882	Y+LAT2 Utilized transport	1.00 * Sodium [e] + 1.00 * Glycine [c] + 1.00 * L-homoseri	SLC7A6	Transport, extracellular			0
r1883_pos	9	0.080856	1.0922877	4.144266	4.144266	1	0.240222	r1883	Y+LAT2 Utilized transport	1.00 * Sodium [e] + 1.00 * L-alanine [c] + 1.00 * L-homose	SLC7A6	Transport, extracellular			0
r1884_pos	9	0.080856	1.0922877	4.144266	4.144266	1	0.240222	r1884	Y+LAT2 Utilized transport	1.00 * Sodium [e] + 1.00 * L-glutamine [c] + 1.00 * L-homo	SLC7A6	Transport, extracellular			0
r1885_pos	9	0.080856	1.0922877	4.144266	4.144266	1	0.240222	r1885	Y+LAT2 Utilized transport	1.00 * Sodium [e] + 1.00 * L-serine [c] + 1.00 * L-homoseri	SLC7A6	Transport, extracellular			0
r1886_pos	9	0.080856	1.0922877	4.144266	4.144266	1	0.240222	r1886	Y+LAT2 Utilized transport	1.00 * Sodium [e] + 1.00 * L-methionine [c] + 1.00 * L-hon	SLC7A6	Transport, extracellular			0
r1887_pos	9	0.080856	1.0922877	2.854938	2.854938	1	0.240222	r1887	Y+LAT2 Utilized transport	1.00 * Sodium [e] + 1.00 * L-homoserine [e] + 1.00 * L-tryp	SLC7A6	Transport, extracellular			0
r1888_pos	9	0.080856	1.0922877	2.854938	2.854938	1	0.240222	r1888	Y+LAT2 Utilized transport	1.00 * Sodium [e] + 1.00 * L-homoserine [e] + 1.00 * L-phe	SLC7A6	Transport, extracellular			0
r1889_pos	9	0.080856	1.0922877	4.144266	4.144266	1	0.240222	r1889	Y+LAT2 Utilized transport	1.00 * Sodium [e] + 1.00 * L-homoserine [e] + 1.00 * L-tyro	SLC7A6	Transport, extracellular			0
r1890_pos	9	0.080856	1.0922877	4.144266	4.144266	1	0.240222	r1890	Y+LAT2 Utilized transport	1.00 * Sodium [e] + 1.00 * L-cysteine [c] + 1.00 * L-homose	SLC7A6	Transport, extracellular			0
r1891_pos	9	0.080856	1.0922877	4.144266	4.144266	1	0.240222	r1891	Y+LAT2 Utilized transport	1.00 * Sodium [e] + 1.00 * L-homoserine [e] + 1.00 * L-leu	SLC7A6	Transport, extracellular			0
r1892_pos	9	0.080856	1.0922877	4.144266	4.144266	1	0.240222	r1892	Y+LAT2 Utilized transport	1.00 * Sodium [e] + 1.00 * L-homoserine [e] + 1.00 * L-pro	SLC7A6	Transport, extracellular			0
r1893_pos	9	0.080856	1.0922877	4.144266	4.144266	1	0.240222	r1893	Y+LAT2 Utilized transport	1.00 * Sodium [e] + 1.00 * L-asparagine [c] + 1.00 * L-hom	SLC7A6	Transport, extracellular			0
r1894_pos	9	0.080856	1.0922877	4.144266	4.144266	1	0.240222	r1894	Y+LAT2 Utilized transport	1.00 * Sodium [e] + 1.00 * L-homoserine [e] + 1.00 * L-vali	SLC7A6	Transport, extracellular			0
r1895_pos	9	0.080856	1.0922877	4.144266	4.144266	1	0.240222	r1895	Y+LAT2 Utilized transport	1.00 * Sodium [e] + 1.00 * L-threonine [c] + 1.00 * L-homo	SLC7A6	Transport, extracellular			0
r1896_pos	9	0.080856	1.0922877	4.144266	4.144266	1	0.240222	r1896	Y+LAT2 Utilized transport	1.00 * Sodium [e] + 1.00 * L-homoserine [e] + 1.00 * L-isol	SLC7A6	Transport, extracellular			0
r1897_pos	9	0.080856	1.0922877	4.144266	4.144266	1	0.240222	r1897	Y+LAT2 Utilized transport	1.00 * Sodium [e] + 1.00 * Glycine [c] + 1.00 * L-isoleucine	SLC7A6	Transport, extracellular			0
r1898_pos	9	0.080856	1.0922877	4.144266	4.144266	1	0.240222	r1898	Y+LAT2 Utilized transport	1.00 * Sodium [e] + 1.00 * L-alanine [c] + 1.00 * L-isoleucir	SLC7A6	Transport, extracellular			0
r1899_pos	9	0.080856	1.0922877	4.144266	4.144266	1	0.240222	r1899	Y+LAT2 Utilized transport	1.00 * Sodium [e] + 1.00 * L-glutamine [c] + 1.00 * L-isoleu	SLC7A6	Transport, extracellular			0
r1900_pos	9	0.080856	1.0922877	4.144266	4.144266	1	0.240222	r1900	Y+LAT2 Utilized transport	1.00 * Sodium [e] + 1.00 * L-serine [c] + 1.00 * L-isoleucine	SLC7A6	Transport, extracellular			0
r1901_pos	9	0.080856	1.0922877	4.144266	4.144266	1	0.240222	r1901	Y+LAT2 Utilized transport	1.00 * Sodium [e] + 1.00 * L-methionine [c] + 1.00 * L-isol	SLC7A6	Transport, extracellular			0
r1902_pos	9	0.080856	1.0922877	2.854938	2.854938	1	0.240222	r1902	Y+LAT2 Utilized transport	1.00 * Sodium [e] + 1.00 * L-isoleucine [e] + 1.00 * L-trypt	SLC7A6	Transport, extracellular			0
r1903_pos	9	0.080856	1.0922877	3.080632	3.080632	1	0.240222	r1903	Y+LAT2 Utilized transport	1.00 * Sodium [e] + 1.00 * L-isoleucine [e] + 1.00 * L-phen	SLC7A6	Transport, extracellular			0
r1904_pos	9	0.080856	1.0922877	4.144266	4.144266	1	0.240222	r1904	Y+LAT2 Utilized transport	1.00 * Sodium [e] + 1.00 * L-isoleucine [e] + 1.00 * L-tyrosi	SLC7A6	Transport, extracellular			0
r1905_pos	9	0.080856	1.0922877	4.144266	4.144266	1	0.240222	r1905	Y+LAT2 Utilized transport	1.00 * Sodium [e] + 1.00 * L-cysteine [c] + 1.00 * L-isoleuci	SLC7A6	Transport, extracellular			0
r1906_pos	9	0.080856	1.0922877	3.037857	3.037857	1	0.240222	r1906	Y+LAT2 Utilized transport	1.00 * Sodium [e] + 1.00 * L-isoleucine [e] + 1.00 * L-leucir	SLC7A6	Transport, extracellular			0
r1907_pos	9	0.080856	1.0922877	4.144266	4.144266	1	0.240222	r1907	Y+LAT2 Utilized transport	1.00 * Sodium [e] + 1.00 * L-isoleucine [e] + 1.00 * L-prolir	SLC7A6	Transport, extracellular			0
r1908_pos	9	0.080856	1.0922877	4.144266	4.144266	1	0.240222	r1908	Y+LAT2 Utilized transport	1.00 * Sodium [e] + 1.00 * L-asparagine [c] + 1.00 * L-isole	SLC7A6	Transport, extracellular			0

r1909_pos	9	0.080856	1.0922877	4.144266	4.144266	1	0.240222	r1909	Y+LAT2 Utilized transport	1.00 * Sodium [e] + 1.00 * L-isoleucine [e] + 1.00 * L-valin	SLC7A6	Transport, extracellular			0
r1910_pos	9	0.080856	1.0922877	4.144266	4.144266	1	0.240222	r1910	Y+LAT2 Utilized transport	1.00 * Sodium [e] + 1.00 * L-threonine [c] + 1.00 * L-isoleu	SLC7A6	Transport, extracellular			0
r1911_pos	9	0.080856	1.0922877	4.144266	4.144266	1	0.240222	r1911	Y+LAT2 Utilized transport	1.00 * Sodium [e] + 1.00 * L-isoleucine [e] + 1.00 * L-homo	SLC7A6	Transport, extracellular			0
r1912_pos	9	0.080856	1.0922877	2.59361	2.59361	1	0.240222	r1912	Y+LAT2 Utilized transport	1.00 * Sodium [e] + 1.00 * Glycine [e] + 1.00 * L-lysinium(1	SLC7A6	Transport, extracellular			0
r1913_pos	9	0.080856	1.0922877	2.59361	2.59361	1	0.240222	r1913	Y+LAT2 Utilized transport	1.00 * Sodium [e] + 1.00 * Glycine [e] + 1.00 * L-argininiur	SLC7A6	Transport, extracellular			0
r1914_pos	9	0.080856	1.0922877	2.59361	2.59361	1	0.240222	r1914	Y+LAT2 Utilized transport	1.00 * Sodium [e] + 1.00 * Ornithine [c] + 1.00 * Glycine [e	SLC7A6	Transport, extracellular			0
r1915_pos	9	0.080856	1.0922877	2.59361	2.59361	1	0.240222	r1915	Y+LAT2 Utilized transport	1.00 * Sodium [e] + 1.00 * Glycine [e] + 1.00 * L-histidine [	SLC7A6	Transport, extracellular			0
r1916_pos	9	0.080856	1.0922877	2.59361	2.59361	1	0.240222	r1916	Y+LAT2 Utilized transport	1.00 * Sodium [e] + 1.00 * Glycine [e] + 1.00 * L-citrulline	SLC7A6	Transport, extracellular			0
r1917_pos	9	0.080856	1.0922877	2.679384	2.679384	1	0.240222	r1917	Y+LAT2 Utilized transport	1.00 * Sodium [e] + 1.00 * L-alanine [e] + 1.00 * L-lysinium	SLC7A6	Transport, extracellular			0
r1918_pos	9	0.080856	1.0922877	2.679384	2.679384	1	0.240222	r1918	Y+LAT2 Utilized transport	1.00 * Sodium [e] + 1.00 * L-alanine [e] + 1.00 * L-argininiu	SLC7A6	Transport, extracellular			0
r1919_pos	9	0.080856	1.0922877	2.65646	2.65646	1	0.240222	r1919	Y+LAT2 Utilized transport	1.00 * Sodium [e] + 1.00 * Ornithine [c] + 1.00 * L-alanine	SLC7A6	Transport, extracellular			0
r1920_pos	9	0.080856	1.0922877	2.695763	2.695763	1	0.240222	r1920	Y+LAT2 Utilized transport	1.00 * Sodium [e] + 1.00 * L-alanine [e] + 1.00 * L-histidine	SLC7A6	Transport, extracellular			0
r1921_pos	9	0.080856	1.0922877	2.59361	2.59361	1	0.240222	r1921	Y+LAT2 Utilized transport	1.00 * Sodium [e] + 1.00 * L-alanine [e] + 1.00 * L-citrullin	SLC7A6	Transport, extracellular			0
r1922_pos	9	0.080856	1.0922877	2.538532	2.538532	1	0.240222	r1922	Y+LAT2 Utilized transport	1.00 * Sodium [e] + 1.00 * L-glutamine [e] + 1.00 * L-lysini	SLC7A6	Transport, extracellular			0
r1923_pos	9	0.080856	1.0922877	2.538532	2.538532	1	0.240222	r1923	Y+LAT2 Utilized transport	1.00 * Sodium [e] + 1.00 * L-glutamine [e] + 1.00 * L-argini	SLC7A6	Transport, extracellular			0
r1924_pos	9	0.080856	1.0922877	2.525797	2.525797	1	0.240222	r1924	Y+LAT2 Utilized transport	1.00 * Sodium [e] + 1.00 * Ornithine [c] + 1.00 * L-glutami	SLC7A6	Transport, extracellular			0
r1925_pos	9	0.080856	1.0922877	2.545382	2.545382	1	0.240222	r1925	Y+LAT2 Utilized transport	1.00 * Sodium [e] + 1.00 * L-glutamine [e] + 1.00 * L-histid	SLC7A6	Transport, extracellular			0
r1926_pos	9	0.080856	1.0922877	2.59361	2.59361	1	0.240222	r1926	Y+LAT2 Utilized transport	1.00 * Sodium [e] + 1.00 * L-glutamine [e] + 1.00 * L-citrul	SLC7A6	Transport, extracellular			0
r1928_pos	9	0.080856	1.0922877	2.59361	2.59361	1	0.240222	r1928	Y+LAT2 Utilized transport	1.00 * Sodium [e] + 1.00 * L-serine [e] + 1.00 * L-argininiur	SLC7A6	Transport, extracellular			0
r1929_pos	9	0.080856	1.0922877	2.59361	2.59361	1	0.240222	r1929	Y+LAT2 Utilized transport	1.00 * Sodium [e] + 1.00 * Ornithine [c] + 1.00 * L-serine [e	SLC7A6	Transport, extracellular			0
r1930_pos	9	0.080856	1.0922877	2.59361	2.59361	1	0.240222	r1930	Y+LAT2 Utilized transport	1.00 * Sodium [e] + 1.00 * L-serine [e] + 1.00 * L-histidine [	SLC7A6	Transport, extracellular			0
r1931_pos	9	0.080856	1.0922877	2.59361	2.59361	1	0.240222	r1931	Y+LAT2 Utilized transport	1.00 * Sodium [e] + 1.00 * L-serine [e] + 1.00 * L-citrulline	SLC7A6	Transport, extracellular			0
r1932_pos	9	0.080856	1.0922877	2.885731	2.885731	1	0.240222	r1932	Y+LAT2 Utilized transport	1.00 * Sodium [e] + 1.00 * L-lysinium(1+) [c] + 1.00 * L-me	SLC7A6	Transport, extracellular			0
r1933_pos	9	0.080856	1.0922877	2.885731	2.885731	1	0.240222	r1933	Y+LAT2 Utilized transport	1.00 * Sodium [e] + 1.00 * L-argininium(1+) [c] + 1.00 * L-r	SLC7A6	Transport, extracellular			0
r1934_pos	9	0.080856	1.0922877	2.854084	2.854084	1	0.240222	r1934	Y+LAT2 Utilized transport	1.00 * Sodium [e] + 1.00 * Ornithine [c] + 1.00 * L-methio	SLC7A6	Transport, extracellular			0
r1935_pos	9	0.080856	1.0922877	2.90081	2.90081	1	0.240222	r1935	Y+LAT2 Utilized transport	1.00 * Sodium [e] + 1.00 * L-methionine [e] + 1.00 * L-hist	SLC7A6	Transport, extracellular			0
r1936_pos	9	0.080856	1.0922877	2.59361	2.59361	1	0.240222	r1936	Y+LAT2 Utilized transport	1.00 * Sodium [e] + 1.00 * L-citrulline [c] + 1.00 * L-methi	SLC7A6	Transport, extracellular			0
r1937_pos	9	0.080856	1.0922877	2.59361	2.59361	1	0.240222	r1937	Y+LAT2 Utilized transport	1.00 * Sodium [e] + 1.00 * L-lysinium(1+) [c] + 1.00 * L-try	SLC7A6	Transport, extracellular			0
r1938_pos	9	0.080856	1.0922877	2.59361	2.59361	1	0.240222	r1938	Y+LAT2 Utilized transport	1.00 * Sodium [e] + 1.00 * L-argininium(1+) [c] + 1.00 * L-t	SLC7A6	Transport, extracellular			0
r1939_pos	9	0.080856	1.0922877	2.59361	2.59361	1	0.240222	r1939	Y+LAT2 Utilized transport	1.00 * Sodium [e] + 1.00 * Ornithine [c] + 1.00 * L-tryptop	SLC7A6	Transport, extracellular			0
r1940_pos	9	0.080856	1.0922877	2.59361	2.59361	1	0.240222	r1940	Y+LAT2 Utilized transport	1.00 * Sodium [e] + 1.00 * L-tryptophan [e] + 1.00 * L-hist	SLC7A6	Transport, extracellular			0
r1941_pos	9	0.080856	1.0922877	2.59361	2.59361	1	0.240222	r1941	Y+LAT2 Utilized transport	1.00 * Sodium [e] + 1.00 * L-citrulline [c] + 1.00 * L-trypto	SLC7A6	Transport, extracellular			0
r1942_pos	9	0.080856	1.0922877	2.59361	2.59361	1	0.240222	r1942	Y+LAT2 Utilized transport	1.00 * Sodium [e] + 1.00 * L-lysinium(1+) [c] + 1.00 * L-phe	SLC7A6	Transport, extracellular			0
r1943_pos	9	0.080856	1.0922877	2.59361	2.59361	1	0.240222	r1943	Y+LAT2 Utilized transport	1.00 * Sodium [e] + 1.00 * L-argininium(1+) [c] + 1.00 * L-p	SLC7A6	Transport, extracellular			0
r1944_pos	9	0.080856	1.0922877	2.59361	2.59361	1	0.240222	r1944	Y+LAT2 Utilized transport	1.00 * Sodium [e] + 1.00 * Ornithine [c] + 1.00 * L-phenyla	SLC7A6	Transport, extracellular			0
r1945_pos	9	0.080856	1.0922877	2.59361	2.59361	1	0.240222	r1945	Y+LAT2 Utilized transport	1.00 * Sodium [e] + 1.00 * L-phenylalanine [e] + 1.00 * L-h	SLC7A6	Transport, extracellular			0
r1946_pos	9	0.080856	1.0922877	2.59361	2.59361	1	0.240222	r1946	Y+LAT2 Utilized transport	1.00 * Sodium [e] + 1.00 * L-citrulline [c] + 1.00 * L-pheny	SLC7A6	Transport, extracellular			0
r1947_pos	9	0.080856	1.0922877	2.59361	2.59361	1	0.240222	r1947	Y+LAT2 Utilized transport	1.00 * Sodium [e] + 1.00 * L-lysinium(1+) [c] + 1.00 * L-tyr	SLC7A6	Transport, extracellular			0
r1948_pos	9	0.080856	1.0922877	2.59361	2.59361	1	0.240222	r1948	Y+LAT2 Utilized transport	1.00 * Sodium [e] + 1.00 * L-argininium(1+) [c] + 1.00 * L-t	SLC7A6	Transport, extracellular			0
r1949_pos	9	0.080856	1.0922877	2.59361	2.59361	1	0.240222	r1949	Y+LAT2 Utilized transport	1.00 * Sodium [e] + 1.00 * Ornithine [c] + 1.00 * L-tyrosine	SLC7A6	Transport, extracellular			0
r1950_pos	9	0.080856	1.0922877	2.59361	2.59361	1	0.240222	r1950	Y+LAT2 Utilized transport	1.00 * Sodium [e] + 1.00 * L-tyrosine [e] + 1.00 * L-histidir	SLC7A6	Transport, extracellular			0
r1951_pos	9	0.080856	1.0922877	2.59361	2.59361	1	0.240222	r1951	Y+LAT2 Utilized transport	1.00 * Sodium [e] + 1.00 * L-citrulline [c] + 1.00 * L-tyrosir	SLC7A6	Transport, extracellular			0
r1952_pos	9	0.080856	1.0922877	2.885731	2.885731	1	0.240222	r1952	Y+LAT2 Utilized transport	1.00 * Sodium [e] + 1.00 * L-cysteine [e] + 1.00 * L-lysiniur	SLC7A6	Transport, extracellular			0
r1953_pos	9	0.080856	1.0922877	2.885731	2.885731	1	0.240222	r1953	Y+LAT2 Utilized transport	1.00 * Sodium [e] + 1.00 * L-cysteine [e] + 1.00 * L-arginini	SLC7A6	Transport, extracellular			0
r1954_pos	9	0.080856	1.0922877	2.854084	2.854084	1	0.240222	r1954	Y+LAT2 Utilized transport	1.00 * Sodium [e] + 1.00 * Ornithine [c] + 1.00 * L-cysteine	SLC7A6	Transport, extracellular			0
r1955_pos	9	0.080856	1.0922877	2.90081	2.90081	1	0.240222	r1955	Y+LAT2 Utilized transport	1.00 * Sodium [e] + 1.00 * L-cysteine [e] + 1.00 * L-histidir	SLC7A6	Transport, extracellular			0
r1956_pos	9	0.080856	1.0922877	2.59361	2.59361	1	0.240222	r1956	Y+LAT2 Utilized transport	1.00 * Sodium [e] + 1.00 * L-cysteine [e] + 1.00 * L-citrullir	SLC7A6	Transport, extracellular			0
r1957_pos	9	0.080856	1.0922877	2.59361	2.59361	1	0.240222	r1957	Y+LAT2 Utilized transport	1.00 * Sodium [e] + 1.00 * L-lysinium(1+) [c] + 1.00 * L-leu	SLC7A6	Transport, extracellular			0
r1958_pos	9	0.080856	1.0922877	2.59361	2.59361	1	0.240222	r1958	Y+LAT2 Utilized transport	1.00 * Sodium [e] + 1.00 * L-argininium(1+) [c] + 1.00 * L-l	SLC7A6	Transport, extracellular			0
r1959_pos	9	0.080856	1.0922877	2.660891	2.660891	1	0.240222	r1959	Y+LAT2 Utilized transport	1.00 * Sodium [e] + 1.00 * Ornithine [c] + 1.00 * L-leucine	SLC7A6	Transport, extracellular			0
r1960_pos	9	0.080856	1.0922877	2.59361	2.59361	1	0.240222	r1960	Y+LAT2 Utilized transport	1.00 * Sodium [e] + 1.00 * L-leucine [e] + 1.00 * L-histidine	SLC7A6	Transport, extracellular			0
r1961_pos	9	0.080856	1.0922877	2.59361	2.59361	1	0.240222	r1961	Y+LAT2 Utilized transport	1.00 * Sodium [e] + 1.00 * L-citrulline [c] + 1.00 * L-leucin	SLC7A6	Transport, extracellular			0
r1962_pos	9	0.080856	1.0922877	2.59361	2.59361	1	0.240222	r1962	Y+LAT2 Utilized transport	1.00 * Sodium [e] + 1.00 * L-lysinium(1+) [c] + 1.00 * L-pro	SLC7A6	Transport, extracellular			0
r1963_pos	9	0.080856	1.0922877	2.59361	2.59361	1	0.240222	r1963	Y+LAT2 Utilized transport	1.00 * Sodium [e] + 1.00 * L-argininium(1+) [c] + 1.00 * L-p	SLC7A6	Transport, extracellular			0
r1964_pos	9	0.080856	1.0922877	2.59361	2.59361	1	0.240222	r1964	Y+LAT2 Utilized transport	1.00 * Sodium [e] + 1.00 * Ornithine [c] + 1.00 * L-proline	SLC7A6	Transport, extracellular			0
r1965_pos	9	0.080856	1.0922877	2.59361	2.59361	1	0.240222	r1965	Y+LAT2 Utilized transport	1.00 * Sodium [e] + 1.00 * L-proline [e] + 1.00 * L-histidine	SLC7A6	Transport, extracellular			0
r1966_pos	9	0.080856	1.0922877	2.59361	2.59361	1	0.240222	r1966	Y+LAT2 Utilized transport	1.00 * Sodium [e] + 1.00 * L-citrulline [c] + 1.00 * L-prolin	SLC7A6	Transport, extracellular			0
r1967_pos	9	0.080856	1.0922877	2.538532	2.538532	1	0.240222	r1967	Y+LAT2 Utilized transport	1.00 * Sodium [e] + 1.00 * L-asparagine [e] + 1.00 * L-lysini	SLC7A6	Transport, extracellular			0
r1968_pos	9	0.080856	1.0922877	2.538532	2.538532	1	0.240222	r1968	Y+LAT2 Utilized transport	1.00 * Sodium [e] + 1.00 * L-asparagine [e] + 1.00 * L-argin	SLC7A6	Transport, extracellular			0
r1969_pos	9	0.080856	1.0922877	2.525797	2.525797	1	0.240222	r1969	Y+LAT2 Utilized transport	1.00 * Sodium [e] + 1.00 * Ornithine [c] + 1.00 * L-asparag	SLC7A6	Transport, extracellular			0
r1970_pos	9	0.080856	1.0922877	2.545382	2.545382	1	0.240222	r1970	Y+LAT2 Utilized transport	1.00 * Sodium [e] + 1.00 * L-asparagine [e] + 1.00 * L-histid	SLC7A6	Transport, extracellular			0
r1971_pos	9	0.080856	1.0922877	2.59361	2.59361	1	0.240222	r1971	Y+LAT2 Utilized transport	1.00 * Sodium [e] + 1.00 * L-asparagine [e] + 1.00 * L-citru	SLC7A6	Transport, extracellular			0
r1972_pos	9	0.080856	1.0922877	2.59361	2.59361	1	0.240222	r1972	Y+LAT2 Utilized transport	1.00 * Sodium [e] + 1.00 * L-lysinium(1+) [c] + 1.00 * L-vali	SLC7A6	Transport, extracellular			0
r1973_pos	9	0.080856	1.0922877	2.59361	2.59361	1	0.240222	r1973	Y+LAT2 Utilized transport	1.00 * Sodium [e] + 1.00 * L-argininium(1+) [c] + 1.00 * L-v	SLC7A6	Transport, extracellular			0
r1974_pos	9	0.080856	1.0922877	2.59361	2.59361	1	0.240222	r1974	Y+LAT2 Utilized transport	1.00 * Sodium [e] + 1.00 * Ornithine [c] + 1.00 * L-valine [e	SLC7A6	Transport, extracellular			0
r1975_pos	9	0.080856	1.0922877	2.59361	2.59361	1	0.240222	r1975	Y+LAT2 Utilized transport	1.00 * Sodium [e] + 1.00 * L-valine [e] + 1.00 * L-histidine [	SLC7A6	Transport, extracellular			0
r1976_pos	9	0.080856	1.0922877	2.59361	2.59361	1	0.240222	r1976	Y+LAT2 Utilized transport	1.00 * Sodium [e] + 1.00 * L-citrulline [c] + 1.00 * L-valine	SLC7A6	Transport, extracellular			0
r1977_pos	9	0.080856	1.0922877	2.59361	2.59361	1	0.240222	r1977	Y+LAT2 Utilized transport	1.00 * Sodium [e] + 1.00 * L-threonine [e] + 1.00 * L-lysini	SLC7A6	Transport, extracellular			0
r1978_pos	9	0.080856	1.0922877	2.59361	2.59361	1	0.240222	r1978	Y+LAT2 Utilized transport	1.00 * Sodium [e] + 1.00 * L-threonine [e] + 1.00 * L-argini	SLC7A6	Transport, extracellular			0
r1979_pos	9	0.080856	1.0922877	2.59361	2.59361	1	0.240222	r1979	Y+LAT2 Utilized transport	1.00 * Sodium [e] + 1.00 * Ornithine [c] + 1.00 * L-threoni	SLC7A6	Transport, extracellular			0
r1980_pos	9	0.080856	1.0922877	2.59361	2.59361	1	0.240222	r1980	Y+LAT2 Utilized transport	1.00 * Sodium [e] + 1.00 * L-threonine [e] + 1.00 * L-histid	SLC7A6	Transport, extracellular			0
r1981_pos	9	0.080856	1.0922877	2.59361	2.59361	1	0.240222	r1981	Y+LAT2 Utilized transport	1.00 * Sodium [e] + 1.00 * L-threonine [e] + 1.00 * L-citrul	SLC7A6	Transport, extracellular			0
r1982_pos	9	0.080856	1.0922877	2.59361	2.59361	1	0.240222	r1982	Y+LAT2 Utilized transport	1.00 * Sodium [e] + 1.00 * L-lysinium(1+) [c] + 1.00 * L-hor	SLC7A6	Transport, extracellular			0
r1983_pos	9	0.080856	1.0922877	2.59361	2.59361	1	0.240222	r1983	Y+LAT2 Utilized transport	1.00 * Sodium [e] + 1.00 * L-argininium(1+) [c] + 1.00 * L-h	SLC7A6	Transport, extracellular			0
r1984_pos	9	0.080856	1.0922877	2.59361	2.59361	1	0.240222	r1984	Y+LAT2 Utilized transport	1.00 * Sodium [e] + 1.00 * Ornithine [c] + 1.00 * L-homose	SLC7A6	Transport, extracellular			0
r1985_pos	9	0.080856	1.0922877	2.59361	2.59361	1	0.240222	r1985	Y+LAT2 Utilized transport	1.00 * Sodium [e] + 1.00 * L-homoserine [e] + 1.00 * L-hist	SLC7A6	Transport, extracellular			0
r1986_pos	9	0.080856	1.0922877	2.59361	2.59361	1	0.240222	r1986	Y+LAT2 Utilized transport	1.00 * Sodium [e] + 1.00 * L-citrulline [c] + 1.00 * L-homos	SLC7A6	Transport, extracellular			0
r1987_pos	9	0.080856	1.0922877	2.59361	2.59361	1	0.240222	r1987	Y+LAT2 Utilized transport	1.00 * Sodium [e] + 1.00 * L-lysinium(1+) [c] + 1.00 * L-isol	SLC7A6	Transport, extracellular			0
r1988_pos	9	0.080856	1.0922877	2.59361	2.59361	1	0.240222	r1988	Y+LAT2 Utilized transport	1.00 * Sodium [e] + 1.00 * L-argininium(1+) [c] + 1.00 * L-i	SLC7A6	Transport, extracellular			0
r1989_pos	9	0.080856	1.0922877	2.59361	2.59361	1	0.240222	r1989	Y+LAT2 Utilized transport	1.00 * Sodium [e] + 1.00 * Ornithine [c] + 1.00 * L-isoleuci	SLC7A6	Transport, extracellular			0
r1990_pos	9	0.080856	1.0922877	2.59361	2.59361	1	0.240222	r1990	Y+LAT2 Utilized transport	1.00 * Sodium [e] + 1.00 * L-isoleucine [e] + 1.00 * L-histid	SLC7A6	Transport, extracellular			0
r1991_pos	9	0.080856	1.0922877	2.59361	2.59361	1	0.240222	r1991	Y+LAT2 Utilized transport	1.00 * Sodium [e] + 1.00 * L-citrulline [c] + 1.00 * L-isoleu	SLC7A6	Transport, extracellular			0
r1992_pos	9	0.080856	1.0922877	2.815705	2.815705	1	0.240222	r1992	Amino Acid-Polyamine-Organocation (APC) TCDB:2.A.3.8.15	1.00 * Glycine [c] + 1.00 * L-lysinium(1+) [e] --> 1.00 * Gly	SLC7A9	Transport, extracellular			0

r1993_pos	9	0.080856	1.0922877	3.224989	3.224989	1	0.240222	r1993	Amino Acid-Polyamine-Organocation (APC) TCDB:2.A.3.8.15	1.00 * L-alanine [c] + 1.00 * L-lysinium(1+) [e] --> 1.00 * L-a	SLC7A9	Transport, extracellular			0
r1994_pos	9	0.080856	1.0922877	3.039758	3.039758	1	0.240222	r1994	Amino Acid-Polyamine-Organocation (APC) TCDB:2.A.3.8.15	1.00 * L-glutamine [c] + 1.00 * L-lysinium(1+) [e] --> 1.00 *	SLC7A9	Transport, extracellular			0
r1996_pos	9	0.080856	1.0922877	3.039849	3.039849	1	0.240222	r1996	Amino Acid-Polyamine-Organocation (APC) TCDB:2.A.3.8.15	1.00 * L-lysinium(1+) [e] + 1.00 * L-methionine [c] --> 1.00	SLC7A9	Transport, extracellular			0
r1997_pos	9	0.080856	1.0922877	2.957988	2.957988	1	0.240222	r1997	Amino Acid-Polyamine-Organocation (APC) TCDB:2.A.3.8.15	1.00 * L-lysinium(1+) [e] + 1.00 * L-tryptophan [c] --> 1.00	SLC7A9	Transport, extracellular			0
r1998_pos	9	0.080856	1.0922877	3.091586	3.091586	1	0.240222	r1998	Amino Acid-Polyamine-Organocation (APC) TCDB:2.A.3.8.15	1.00 * L-lysinium(1+) [e] + 1.00 * L-phenylalanine [c] --> 1.	SLC7A9	Transport, extracellular			0
r1999_pos	9	0.080856	1.0922877	2.969658	2.969658	1	0.240222	r1999	Amino Acid-Polyamine-Organocation (APC) TCDB:2.A.3.8.15	1.00 * L-lysinium(1+) [e] + 1.00 * L-tyrosine [c] --> 1.00 * L-	SLC7A9	Transport, extracellular			0
r2000_pos	9	0.080856	1.0922877	2.969658	2.969658	1	0.240222	r2000	Amino Acid-Polyamine-Organocation (APC) TCDB:2.A.3.8.15	1.00 * L-cysteine [c] + 1.00 * L-lysinium(1+) [e] --> 1.00 * L-	SLC7A9	Transport, extracellular			0
r2001_pos	9	0.080856	1.0922877	3.039849	3.039849	1	0.240222	r2001	Amino Acid-Polyamine-Organocation (APC) TCDB:2.A.3.8.15	1.00 * L-lysinium(1+) [e] + 1.00 * L-leucine [c] --> 1.00 * L-l	SLC7A9	Transport, extracellular			0
r2002_pos	9	0.080856	1.0922877	2.969658	2.969658	1	0.240222	r2002	Amino Acid-Polyamine-Organocation (APC) TCDB:2.A.3.8.15	1.00 * L-lysinium(1+) [e] + 1.00 * L-proline [c] --> 1.00 * L-l	SLC7A9	Transport, extracellular			0
r2003_pos	9	0.080856	1.0922877	2.834945	2.834945	1	0.240222	r2003	Amino Acid-Polyamine-Organocation (APC) TCDB:2.A.3.8.15	1.00 * L-asparagine [c] + 1.00 * L-lysinium(1+) [e] --> 1.00 *	SLC7A9	Transport, extracellular			0
r2004_pos	9	0.080856	1.0922877	2.832987	2.832987	1	0.240222	r2004	Amino Acid-Polyamine-Organocation (APC) TCDB:2.A.3.8.15	1.00 * L-lysinium(1+) [e] + 1.00 * L-valine [c] --> 1.00 * L-ly	SLC7A9	Transport, extracellular			0
r2005_pos	9	0.080856	1.0922877	2.834945	2.834945	1	0.240222	r2005	Amino Acid-Polyamine-Organocation (APC) TCDB:2.A.3.8.15	1.00 * L-threonine [c] + 1.00 * L-lysinium(1+) [e] --> 1.00 *	SLC7A9	Transport, extracellular			0
r2006_pos	9	0.080856	1.0922877	2.834945	2.834945	1	0.240222	r2006	Amino Acid-Polyamine-Organocation (APC) TCDB:2.A.3.8.15	1.00 * L-lysinium(1+) [e] + 1.00 * L-homoserine [c] --> 1.00	SLC7A9	Transport, extracellular			0
r2007_pos	9	0.080856	1.0922877	2.832987	2.832987	1	0.240222	r2007	Amino Acid-Polyamine-Organocation (APC) TCDB:2.A.3.8.15	1.00 * L-lysinium(1+) [e] + 1.00 * L-isoleucine [c] --> 1.00 *	SLC7A9	Transport, extracellular			0
r2008_pos	9	0.080856	1.0922877	3.203779	3.203779	1	0.240222	r2008	Amino Acid-Polyamine-Organocation (APC) TCDB:2.A.3.8.15	1.00 * Glycine [c] + 1.00 * L-argininium(1+) [e] --> 1.00 * G	SLC7A9	Transport, extracellular			0
r2011_pos	9	0.080856	1.0922877	2.33227	2.33227	1	0.240222	r2011	Amino Acid-Polyamine-Organocation (APC) TCDB:2.A.3.8.15	1.00 * L-serine [c] + 1.00 * L-argininium(1+) [e] --> 1.00 * L	SLC7A9	Transport, extracellular			0
r2013_pos	9	0.080856	1.0922877	2.417374	2.417374	1	0.240222	r2013	Amino Acid-Polyamine-Organocation (APC) TCDB:2.A.3.8.15	1.00 * L-argininium(1+) [e] + 1.00 * L-tryptophan [c] --> 1.	SLC7A9	Transport, extracellular			0
r2015_pos	9	0.080856	1.0922877	2.508021	2.508021	1	0.240222	r2015	Amino Acid-Polyamine-Organocation (APC) TCDB:2.A.3.8.15	1.00 * L-argininium(1+) [e] + 1.00 * L-tyrosine [c] --> 1.00 *	SLC7A9	Transport, extracellular			0
r2016_pos	9	0.080856	1.0922877	2.902113	2.902113	1	0.240222	r2016	Amino Acid-Polyamine-Organocation (APC) TCDB:2.A.3.8.15	1.00 * L-cysteine [c] + 1.00 * L-argininium(1+) [e] --> 1.00 *	SLC7A9	Transport, extracellular			0
r2018_pos	9	0.080856	1.0922877	2.949726	2.949726	1	0.240222	r2018	Amino Acid-Polyamine-Organocation (APC) TCDB:2.A.3.8.15	1.00 * L-argininium(1+) [e] + 1.00 * L-proline [c] --> 1.00 *	SLC7A9	Transport, extracellular			0
r2019_pos	9	0.080856	1.0922877	2.222485	2.222485	1	0.240222	r2019	Amino Acid-Polyamine-Organocation (APC) TCDB:2.A.3.8.15	1.00 * L-asparagine [c] + 1.00 * L-argininium(1+) [e] --> 1.0	SLC7A9	Transport, extracellular			0
r2020_pos	9	0.080856	1.0922877	2.262072	2.262072	1	0.240222	r2020	Amino Acid-Polyamine-Organocation (APC) TCDB:2.A.3.8.15	1.00 * L-argininium(1+) [e] + 1.00 * L-valine [c] --> 1.00 * L	SLC7A9	Transport, extracellular			0
r2021_pos	9	0.080856	1.0922877	2.33227	2.33227	1	0.240222	r2021	Amino Acid-Polyamine-Organocation (APC) TCDB:2.A.3.8.15	1.00 * L-threonine [c] + 1.00 * L-argininium(1+) [e] --> 1.00	SLC7A9	Transport, extracellular			0
r2022_pos	9	0.080856	1.0922877	2.321032	2.321032	1	0.240222	r2022	Amino Acid-Polyamine-Organocation (APC) TCDB:2.A.3.8.15	1.00 * L-argininium(1+) [e] + 1.00 * L-homoserine [c] --> 1.	SLC7A9	Transport, extracellular			0
r2023_pos	9	0.080856	1.0922877	2.262072	2.262072	1	0.240222	r2023	Amino Acid-Polyamine-Organocation (APC) TCDB:2.A.3.8.15	1.00 * L-argininium(1+) [e] + 1.00 * L-isoleucine [c] --> 1.0	SLC7A9	Transport, extracellular			0
r2024_pos	9	0.080856	1.0922877	2.59361	2.59361	1	0.240222	r2024	Amino Acid-Polyamine-Organocation (APC) TCDB:2.A.3.8.15	1.00 * Glycine [c] + 1.00 * Ornithine [e] --> 1.00 * Ornithin	SLC7A9	Transport, extracellular			0
r2026_pos	9	0.080856	1.0922877	2.518744	2.518744	1	0.240222	r2026	Amino Acid-Polyamine-Organocation (APC) TCDB:2.A.3.8.15	1.00 * L-glutamine [c] + 1.00 * Ornithine [e] --> 1.00 * Orn	SLC7A9	Transport, extracellular			0
r2027_pos	9	0.080856	1.0922877	2.518744	2.518744	1	0.240222	r2027	Amino Acid-Polyamine-Organocation (APC) TCDB:2.A.3.8.15	1.00 * L-serine [c] + 1.00 * Ornithine [e] --> 1.00 * Ornithin	SLC7A9	Transport, extracellular			0
r2028_pos	9	0.080856	1.0922877	2.517151	2.517151	1	0.240222	r2028	Amino Acid-Polyamine-Organocation (APC) TCDB:2.A.3.8.15	1.00 * L-methionine [c] + 1.00 * Ornithine [e] --> 1.00 * Or	SLC7A9	Transport, extracellular			0
r2029_pos	9	0.080856	1.0922877	2.588227	2.588227	1	0.240222	r2029	Amino Acid-Polyamine-Organocation (APC) TCDB:2.A.3.8.15	1.00 * Ornithine [e] + 1.00 * L-tryptophan [c] --> 1.00 * Or	SLC7A9	Transport, extracellular			0
r2030_pos	9	0.080856	1.0922877	2.526388	2.526388	1	0.240222	r2030	Amino Acid-Polyamine-Organocation (APC) TCDB:2.A.3.8.15	1.00 * Ornithine [e] + 1.00 * L-phenylalanine [c] --> 1.00 *	SLC7A9	Transport, extracellular			0
r2031_pos	9	0.080856	1.0922877	2.594964	2.594964	1	0.240222	r2031	Amino Acid-Polyamine-Organocation (APC) TCDB:2.A.3.8.15	1.00 * Ornithine [e] + 1.00 * L-tyrosine [c] --> 1.00 * Ornith	SLC7A9	Transport, extracellular			0
r2032_pos	9	0.080856	1.0922877	2.59361	2.59361	1	0.240222	r2032	Amino Acid-Polyamine-Organocation (APC) TCDB:2.A.3.8.15	1.00 * L-cysteine [c] + 1.00 * Ornithine [e] --> 1.00 * Ornith	SLC7A9	Transport, extracellular			0
r2034_pos	9	0.080856	1.0922877	2.59361	2.59361	1	0.240222	r2034	Amino Acid-Polyamine-Organocation (APC) TCDB:2.A.3.8.15	1.00 * Ornithine [e] + 1.00 * L-proline [c] --> 1.00 * Ornith	SLC7A9	Transport, extracellular			0
r2035_pos	9	0.080856	1.0922877	2.518744	2.518744	1	0.240222	r2035	Amino Acid-Polyamine-Organocation (APC) TCDB:2.A.3.8.15	1.00 * L-asparagine [c] + 1.00 * Ornithine [e] --> 1.00 * Orn	SLC7A9	Transport, extracellular			0
r2036_pos	9	0.080856	1.0922877	2.517151	2.517151	1	0.240222	r2036	Amino Acid-Polyamine-Organocation (APC) TCDB:2.A.3.8.15	1.00 * Ornithine [e] + 1.00 * L-valine [c] --> 1.00 * Ornithin	SLC7A9	Transport, extracellular			0
r2037_pos	9	0.080856	1.0922877	2.518744	2.518744	1	0.240222	r2037	Amino Acid-Polyamine-Organocation (APC) TCDB:2.A.3.8.15	1.00 * L-threonine [c] + 1.00 * Ornithine [e] --> 1.00 * Orni	SLC7A9	Transport, extracellular			0
r2038_pos	9	0.080856	1.0922877	2.518744	2.518744	1	0.240222	r2038	Amino Acid-Polyamine-Organocation (APC) TCDB:2.A.3.8.15	1.00 * Ornithine [e] + 1.00 * L-homoserine [c] --> 1.00 * Or	SLC7A9	Transport, extracellular			0
r2039_pos	9	0.080856	1.0922877	2.517151	2.517151	1	0.240222	r2039	Amino Acid-Polyamine-Organocation (APC) TCDB:2.A.3.8.15	1.00 * Ornithine [e] + 1.00 * L-isoleucine [c] --> 1.00 * Orn	SLC7A9	Transport, extracellular			0
r2040_pos	9	0.080856	1.0922877	2.176635	2.176635	1	0.240222	r2040	Amino Acid-Polyamine-Organocation (APC) TCDB:2.A.3.8.15	1.00 * Glycine [c] + 1.00 * L-histidine [e] --> 1.00 * Glycine	SLC7A9	Transport, extracellular			0
r2041_pos	0	0.080856	1.0922877	-3.22542	3.225423	2	0.240222	r2041	Amino Acid-Polyamine-Organocation (APC) TCDB:2.A.3.8.15	1.00 * L-alanine [c] + 1.00 * L-histidine [e] --> 1.00 * L-alan	SLC7A9	Transport, extracellular			0
r2042_pos	0	0.080856	1.0922877	-3.22542	3.225423	2	0.240222	r2042	Amino Acid-Polyamine-Organocation (APC) TCDB:2.A.3.8.15	1.00 * L-glutamine [c] + 1.00 * L-histidine [e] --> 1.00 * L-g	SLC7A9	Transport, extracellular			0
r2043_pos	9	0.080856	1.0922877	2.219019	2.219019	1	0.240222	r2043	Amino Acid-Polyamine-Organocation (APC) TCDB:2.A.3.8.15	1.00 * L-serine [c] + 1.00 * L-histidine [e] --> 1.00 * L-serine	SLC7A9	Transport, extracellular			0
r2044_pos	9	0.080856	1.0922877	2.294707	2.294707	1	0.240222	r2044	Amino Acid-Polyamine-Organocation (APC) TCDB:2.A.3.8.15	1.00 * L-methionine [c] + 1.00 * L-histidine [e] --> 1.00 * L-	SLC7A9	Transport, extracellular			0
r2045_pos	9	0.080856	1.0922877	2.832089	2.832089	1	0.240222	r2045	Amino Acid-Polyamine-Organocation (APC) TCDB:2.A.3.8.15	1.00 * L-histidine [e] + 1.00 * L-tryptophan [c] --> 1.00 * L-	SLC7A9	Transport, extracellular			0
r2046_pos	9	0.080856	1.0922877	2.257394	2.257394	1	0.240222	r2046	Amino Acid-Polyamine-Organocation (APC) TCDB:2.A.3.8.15	1.00 * L-histidine [e] + 1.00 * L-phenylalanine [c] --> 1.00 *	SLC7A9	Transport, extracellular			0
r2047_pos	9	0.080856	1.0922877	2.58432	2.58432	1	0.240222	r2047	Amino Acid-Polyamine-Organocation (APC) TCDB:2.A.3.8.15	1.00 * L-histidine [e] + 1.00 * L-tyrosine [c] --> 1.00 * L-tyr	SLC7A9	Transport, extracellular			0
r2048_pos	9	0.080856	1.0922877	2.198259	2.198259	1	0.240222	r2048	Amino Acid-Polyamine-Organocation (APC) TCDB:2.A.3.8.15	1.00 * L-cysteine [c] + 1.00 * L-histidine [e] --> 1.00 * L-cys	SLC7A9	Transport, extracellular			0
r2049_pos	9	0.080856	1.0922877	2.444748	2.444748	1	0.240222	r2049	Amino Acid-Polyamine-Organocation (APC) TCDB:2.A.3.8.15	1.00 * L-histidine [e] + 1.00 * L-leucine [c] --> 1.00 * L-leuc	SLC7A9	Transport, extracellular			0
r2050_pos	9	0.080856	1.0922877	2.425681	2.425681	1	0.240222	r2050	Amino Acid-Polyamine-Organocation (APC) TCDB:2.A.3.8.15	1.00 * L-histidine [e] + 1.00 * L-proline [c] --> 1.00 * L-prol	SLC7A9	Transport, extracellular			0
r2051_pos	0	0.080856	1.0922877	-3.22542	3.225423	2	0.240222	r2051	Amino Acid-Polyamine-Organocation (APC) TCDB:2.A.3.8.15	1.00 * L-asparagine [c] + 1.00 * L-histidine [e] --> 1.00 * L-a	SLC7A9	Transport, extracellular			0
r2052_pos	9	0.080856	1.0922877	2.444748	2.444748	1	0.240222	r2052	Amino Acid-Polyamine-Organocation (APC) TCDB:2.A.3.8.15	1.00 * L-histidine [e] + 1.00 * L-valine [c] --> 1.00 * L-valine	SLC7A9	Transport, extracellular			0
r2053_pos	9	0.080856	1.0922877	2.452797	2.452797	1	0.240222	r2053	Amino Acid-Polyamine-Organocation (APC) TCDB:2.A.3.8.15	1.00 * L-threonine [c] + 1.00 * L-histidine [e] --> 1.00 * L-th	SLC7A9	Transport, extracellular			0
r2054_pos	9	0.080856	1.0922877	2.335223	2.335223	1	0.240222	r2054	Amino Acid-Polyamine-Organocation (APC) TCDB:2.A.3.8.15	1.00 * L-histidine [e] + 1.00 * L-homoserine [c] --> 1.00 * L	SLC7A9	Transport, extracellular			0
r2055_pos	9	0.080856	1.0922877	2.444748	2.444748	1	0.240222	r2055	Amino Acid-Polyamine-Organocation (APC) TCDB:2.A.3.8.15	1.00 * L-histidine [e] + 1.00 * L-isoleucine [c] --> 1.00 * L-is	SLC7A9	Transport, extracellular			0
r2056_pos	9	0.080856	1.0922877	2.59361	2.59361	1	0.240222	r2056	Amino Acid-Polyamine-Organocation (APC) TCDB:2.A.3.8.15	1.00 * Glycine [c] + 1.00 * L-citrulline [e] --> 1.00 * Glycine	SLC7A9	Transport, extracellular			0
r2057_pos	9	0.080856	1.0922877	2.59361	2.59361	1	0.240222	r2057	Amino Acid-Polyamine-Organocation (APC) TCDB:2.A.3.8.15	1.00 * L-alanine [c] + 1.00 * L-citrulline [e] --> 1.00 * L-alar	SLC7A9	Transport, extracellular			0
r2058_pos	9	0.080856	1.0922877	2.518744	2.518744	1	0.240222	r2058	Amino Acid-Polyamine-Organocation (APC) TCDB:2.A.3.8.15	1.00 * L-glutamine [c] + 1.00 * L-citrulline [e] --> 1.00 * L-g	SLC7A9	Transport, extracellular			0
r2059_pos	9	0.080856	1.0922877	2.518744	2.518744	1	0.240222	r2059	Amino Acid-Polyamine-Organocation (APC) TCDB:2.A.3.8.15	1.00 * L-serine [c] + 1.00 * L-citrulline [e] --> 1.00 * L-serin	SLC7A9	Transport, extracellular			0
r2060_pos	9	0.080856	1.0922877	2.517151	2.517151	1	0.240222	r2060	Amino Acid-Polyamine-Organocation (APC) TCDB:2.A.3.8.15	1.00 * L-methionine [c] + 1.00 * L-citrulline [e] --> 1.00 * L	SLC7A9	Transport, extracellular			0
r2061_pos	9	0.080856	1.0922877	2.588227	2.588227	1	0.240222	r2061	Amino Acid-Polyamine-Organocation (APC) TCDB:2.A.3.8.15	1.00 * L-tryptophan [c] + 1.00 * L-citrulline [e] --> 1.00 * L	SLC7A9	Transport, extracellular			0
r2062_pos	9	0.080856	1.0922877	2.526388	2.526388	1	0.240222	r2062	Amino Acid-Polyamine-Organocation (APC) TCDB:2.A.3.8.15	1.00 * L-phenylalanine [c] + 1.00 * L-citrulline [e] --> 1.00	SLC7A9	Transport, extracellular			0
r2063_pos	9	0.080856	1.0922877	2.594964	2.594964	1	0.240222	r2063	Amino Acid-Polyamine-Organocation (APC) TCDB:2.A.3.8.15	1.00 * L-tyrosine [c] + 1.00 * L-citrulline [e] --> 1.00 * L-cit	SLC7A9	Transport, extracellular			0
r2064_pos	9	0.080856	1.0922877	2.59361	2.59361	1	0.240222	r2064	Amino Acid-Polyamine-Organocation (APC) TCDB:2.A.3.8.15	1.00 * L-cysteine [c] + 1.00 * L-citrulline [e] --> 1.00 * L-cys	SLC7A9	Transport, extracellular			0
r2065_pos	9	0.080856	1.0922877	2.517151	2.517151	1	0.240222	r2065	Amino Acid-Polyamine-Organocation (APC) TCDB:2.A.3.8.15	1.00 * L-leucine [c] + 1.00 * L-citrulline [e] --> 1.00 * L-citr	SLC7A9	Transport, extracellular			0
r2066_pos	9	0.080856	1.0922877	2.59361	2.59361	1	0.240222	r2066	Amino Acid-Polyamine-Organocation (APC) TCDB:2.A.3.8.15	1.00 * L-proline [c] + 1.00 * L-citrulline [e] --> 1.00 * L-citr	SLC7A9	Transport, extracellular			0
r2067_pos	9	0.080856	1.0922877	2.518744	2.518744	1	0.240222	r2067	Amino Acid-Polyamine-Organocation (APC) TCDB:2.A.3.8.15	1.00 * L-asparagine [c] + 1.00 * L-citrulline [e] --> 1.00 * L-	SLC7A9	Transport, extracellular			0
r2068_pos	9	0.080856	1.0922877	2.517151	2.517151	1	0.240222	r2068	Amino Acid-Polyamine-Organocation (APC) TCDB:2.A.3.8.15	1.00 * L-citrulline [e] + 1.00 * L-valine [c] --> 1.00 * L-citru	SLC7A9	Transport, extracellular			0
r2069_pos	9	0.080856	1.0922877	2.518744	2.518744	1	0.240222	r2069	Amino Acid-Polyamine-Organocation (APC) TCDB:2.A.3.8.15	1.00 * L-threonine [c] + 1.00 * L-citrulline [e] --> 1.00 * L-t	SLC7A9	Transport, extracellular			0
r2070_pos	9	0.080856	1.0922877	2.518744	2.518744	1	0.240222	r2070	Amino Acid-Polyamine-Organocation (APC) TCDB:2.A.3.8.15	1.00 * L-homoserine [c] + 1.00 * L-citrulline [e] --> 1.00 * L	SLC7A9	Transport, extracellular			0
r2071_pos	9	0.080856	1.0922877	2.517151	2.517151	1	0.240222	r2071	Amino Acid-Polyamine-Organocation (APC) TCDB:2.A.3.8.15	1.00 * L-isoleucine [c] + 1.00 * L-citrulline [e] --> 1.00 * L-c	SLC7A9	Transport, extracellular			0
r2136_pos	0	0.080856	1.0922877	-2.66434	2.664337	2	0.240222	r2136	Major Facilitator(MFS) TCDB:2.A.1.14.6	1.00 * Sodium [e] + 1.00 * hydrogenphosphate [e] --> 1.00	SLC17A1	Transport, extracellular			0
r2218_neg	0	0.080856	1.0922877	-2.21417	2.21417	2	0.240222	r2218	Resistance-Nodulation-Cell Division (RND) TCDB:2.A.60.1.5	1.00 * Bicarbonate [c] + 1.00 * arachidonate [e] + 1.00 * g	SLCO1B2	Transport, extracellular			0
r2218_pos	0	0.080856	1.0922877	-2.21417	2.21417	2	0.240222	r2218	Resistance-Nodulation-Cell Division (RND) TCDB:2.A.60.1.5	1.00 * Bicarbonate [c] + 1.00 * arachidonate [e] + 1.00 * g	SLCO1B2	Transport, extracellular			0
r2219_neg	0	0.080856	1.0922877	-2.21417	2.21417	2	0.240222	r2219	Resistance-Nodulation-Cell Division (RND) TCDB:2.A.60.1.5	1.00 * Bicarbonate [c] + 1.00 * arachidonate [e] + 1.00 * S-	SLCO1B2	Transport, extracellular			0
r2219_pos	0	0.080856	1.0922877	-2.21417	2.21417	2	0.240222	r2219	Resistance-Nodulation-Cell Division (RND) TCDB:2.A.60.1.5	1.00 * Bicarbonate [c] + 1.00 * arachidonate [e] + 1.00 * S-	SLCO1B2	Transport, extracellular			0
r2220_neg	0	0.080856	1.0922877	-2.21417	2.21417	2	0.240222	r2220	Resistance-Nodulation-Cell Division (RND) TCDB:2.A.60.1.5	1.00 * Bicarbonate [c] + 1.00 * arachidonate [e] + 1.00 * S-	SLCO1B2	Transport, extracellular			0
r2220_pos	0	0.080856	1.0922877	-2.21417	2.21417	2	0.240222	r2220	Resistance-Nodulation-Cell Division (RND) TCDB:2.A.60.1.5	1.00 * Bicarbonate [c] + 1.00 * arachidonate [e] + 1.00 * S-	SLCO1B2	Transport, extracellular			0
r2293_neg	0	0.080856	1.0922877	-2.21417	2.21417	2	0.240222	r2293	Resistance-Nodulation-Cell Division (RND) TCDB:2.A.60.1.5	1.00 * Bicarbonate [c] + 1.00 * prostaglandin F2alpha(1-)	SLCO1B2	Transport, extracellular			0
r2293_pos	0	0.080856	1.0922877	-2.21417	2.21417	2	0.240222	r2293	Resistance-Nodulation-Cell Division (RND) TCDB:2.A.60.1.5	1.00 * Bicarbonate [c] + 1.00 * prostaglandin F2alpha(1-)	SLCO1B2	Transport, extracellular			0
r2294_neg	0	0.080856	1.0922877	-2.21417	2.21417	2	0.240222	r2294	Resistance-Nodulation-Cell Division (RND) TCDB:2.A.60.1.5	1.00 * Bicarbonate [c] + 1.00 * prostaglandin F2alpha(1-)	SLCO1B2	Transport, extracellular			0
r2294_pos	0	0.080856	1.0922877	-2.21417	2.21417	2	0.240222	r2294	Resistance-Nodulation-Cell Division (RND) TCDB:2.A.60.1.5	1.00 * Bicarbonate [c] + 1.00 * prostaglandin F2alpha(1-)	SLCO1B2	Transport, extracellular			0
r2295_neg	0	0.080856	1.0922877	-2.21417	2.21417	2	0.240222	r2295	Resistance-Nodulation-Cell Division (RND) TCDB:2.A.60.1.5	1.00 * Bicarbonate [c] + 1.00 * prostaglandin F2alpha(1-)	SLCO1B2	Transport, extracellular			0

r2295_pos	0	0.080856	1.0922877	-2.21417	2.21417	2	0.240222	r2295	Resistance-Nodulation-Cell Division (RND) TCDB:2.A.60.1.5	1.00 * Bicarbonate [c] + 1.00 * prostaglandin F2alpha(1-)	SLCO1B2	Transport, extracellular			0
r2447_pos	0	0.080856	1.0922877	-2.81968	2.819676	2	0.240222	r2447	Concentrative Nucleoside Transporter (CNT) TCDB:2.A.41.2.3	1.00 * Sodium [e] + 1.00 * Deoxyuridine [e] --> 1.00 * Sodi	SLC28A1; S	Transport, extracellular			0
r2525_pos	9	0.080856	1.0922877	2.131356	2.131356	1	0.240222	r2525	Major Facilitator(MFS) TCDB:2.A.1.44.1	1.00 * L-glutamine [e] --> 1.00 * L-glutamine [c]SLC43A1	SLC43A1	Transport, extracellular			0
r2526_pos	9	0.080856	1.0922877	2.266923	2.266923	1	0.240222	r2526	Major Facilitator(MFS) TCDB:2.A.1.44.1	1.00 * L-serine [e] --> 1.00 * L-serine [c]SLC43A1	SLC43A1	Transport, extracellular			0
r2532_pos	9	0.080856	1.0922877	2.092413	2.092413	1	0.240222	r2532	Major Facilitator(MFS) TCDB:2.A.1.44.1	1.00 * L-asparagine [e] --> 1.00 * L-asparagine [c]SLC43A1	SLC43A1	Transport, extracellular			0
r2534_pos	9	0.080856	1.0922877	2.266923	2.266923	1	0.240222	r2534	Major Facilitator(MFS) TCDB:2.A.1.44.1	1.00 * L-threonine [e] --> 1.00 * L-threonine [c]SLC43A1	SLC43A1	Transport, extracellular			0
3HCO3_NAt_neg	1	0.19043	0.7202646	-1.45591	1.455908	2	0.347218	3HCO3_NA	3HCO3_NAt	3.00 * Bicarbonate [e] + 1.00 * Sodium [e] --> 3.00 * Bicar	SLC4A4; SL	Transport, extracellular			4
3HCO3_NAt_pos	1	0.19043	0.7202646	-1.46297	1.46297	2	0.347218	3HCO3_NA	3HCO3_NAt	3.00 * Bicarbonate [e] + 1.00 * Sodium [e] --> 3.00 * Bicar	SLC4A4; SL	Transport, extracellular			4
ACETONEt2_pos	1	0.19043	0.7202646	-1.89059	1.890593	2	0.347218	ACETONEt2	acetone transport via proton symport	1.00 * proton [e] + 1.00 * acetone [e] --> 1.00 * proton [c]	SLC16A1; S	Transport, extracellular			4
ADNt4_pos	1	0.19043	0.7202646	-1.80803	1.808029	2	0.347218	ADNt4	adenosine transport (Na/Adn cotransport)	1.00 * Sodium [e] + 1.00 * Adenosine [e] --> 1.00 * Sodium	SLC28A1; S	Transport, extracellular			4
ADNt_neg	1	0.19043	0.7202646	-1.80803	1.808029	2	0.347218	ADNt	adenosine facilated transport in cytosol	1.00 * Adenosine [e] --> 1.00 * Adenosine [c]SLC29A1; SLC	SLC29A1; S	Transport, extracellular			4
ADNt_pos	1	0.19043	0.7202646	-1.46943	1.469431	2	0.347218	ADNt	adenosine facilated transport in cytosol	1.00 * Adenosine [e] --> 1.00 * Adenosine [c]SLC29A1; SLC	SLC29A1; S	Transport, extracellular			4
ALAASNNaEx_pos	8	0.19043	0.7202646	1.240088	1.240088	1	0.347218	ALAASNNaE	L-alanine/L-asparagine Na-dependent exchange (Ala-L in)	1.00 * Sodium [e] + 1.00 * L-alanine [e] + 1.00 * L-asparagi	SLC1A5	Transport, extracellular			4
ALASERNaEx_pos	8	0.19043	0.7202646	1.908201	1.908201	1	0.347218	ALASERNaE	L-alanine/L-serine Na-dependent exchange (Ala-L in)	1.00 * Sodium [e] + 1.00 * L-alanine [e] + 1.00 * L-serine [c	SLC1A4; SL	Transport, extracellular			4
ALATHRNaEx_pos	8	0.19043	0.7202646	1.908201	1.908201	1	0.347218	ALATHRNaE	L-alanine/L-threonine Na-dependent exchange (Ala-L in)	1.00 * Sodium [e] + 1.00 * L-alanine [e] + 1.00 * L-threonir	SLC1A4; SL	Transport, extracellular			4
ARGtiDF_pos	8	0.19043	0.7202646	1.4084	1.4084	1	0.347218	ARGtiDF	L-arginine transport via diffusion (extracellular to cytosol)	1.00 * L-argininium(1+) [e] --> 1.00 * L-argininium(1+) [c]S	SLC38A4; S	Transport, extracellular			4
ASNCYSNaEx_pos	8	0.19043	0.7202646	1.993983	1.993983	1	0.347218	ASNCYSNaE	L-cysteine/L-asparagine Na-dependent exchange (Asn-L in)	1.00 * Sodium [e] + 1.00 * L-asparagine [e] + 1.00 * L-cyste	SLC1A5	Transport, extracellular			4
ASNSERNaEx_pos	8	0.19043	0.7202646	1.515545	1.515545	1	0.347218	ASNSERNaE	L-serine/L-asparagine Na-dependent exchange (Asn-L in)	1.00 * Sodium [e] + 1.00 * L-asparagine [e] + 1.00 * L-serin	SLC1A5	Transport, extracellular			4
ASNTHRNaEx_pos	8	0.19043	0.7202646	1.515545	1.515545	1	0.347218	ASNTHRNaI	L-threonine/L-asparagine Na-dependent exchange (Asn-L in)	1.00 * Sodium [e] + 1.00 * L-asparagine [e] + 1.00 * L-thre	SLC1A5	Transport, extracellular			4
ASNt4_pos	1	0.19043	0.7202646	-1.66853	1.668526	2	0.347218	ASNt4	L-asparagine transport in via sodium symport	1.00 * Sodium [e] + 1.00 * L-asparagine [e] --> 1.00 * Sodiu	ACE2; SLC3	Transport, extracellular			4
BILDGLCURt_neg	1	0.19043	0.7202646	-1.78936	1.789358	2	0.347218	BILDGLCUR	bilirubin beta-diglucuronide transport via bicarbonate countertran	1.00 * Bicarbonate [c] + 1.00 * bis(beta-glucosyluronate)b	SLCO1B2	Transport, extracellular			4
BILDGLCURt_pos	1	0.19043	0.7202646	-1.61251	1.612512	2	0.347218	BILDGLCUR	bilirubin beta-diglucuronide transport via bicarbonate countertran	1.00 * Bicarbonate [c] + 1.00 * bis(beta-glucosyluronate)b	SLCO1B2	Transport, extracellular			4
BILGLCURt_neg	1	0.19043	0.7202646	-1.80401	1.804011	2	0.347218	BILGLCURt	bilirubin monoglucuronide transport via bicarbonate countertrans	1.00 * Bicarbonate [c] + 1.00 * mono(glucosyluronic acid	SLCO1B2	Transport, extracellular			4
BILGLCURt_pos	1	0.19043	0.7202646	-1.6198	1.619795	2	0.347218	BILGLCURt	bilirubin monoglucuronide transport via bicarbonate countertrans	1.00 * Bicarbonate [c] + 1.00 * mono(glucosyluronic acid	SLCO1B2	Transport, extracellular			4
CHOLATEt2_pos	1	0.19043	0.7202646	-1.69187	1.691867	2	0.347218	CHOLATEt2	cholate transport via sodium cotransport	2.00 * Sodium [e] + 1.00 * Cholate [e] --> 2.00 * Sodium [c	SLC10A1; S	Transport, extracellular			4
CHOLATEt_neg	1	0.19043	0.7202646	-1.44763	1.447631	2	0.347218	CHOLATEt	cholate transport via bicarbonate countertransport	1.00 * Bicarbonate [c] + 1.00 * Cholate [e] --> 1.00 * Bicar	SLCO1A1; S	Transport, extracellular			4
CITt4_2_neg	1	0.19043	0.7202646	-1.21355	1.213555	2	0.347218	CITt4_2	citrate transport via sodium symport	2.00 * Sodium [e] + 1.00 * Citrate [e] --> 2.00 * Sodium [c]	SLC13A2	Transport, extracellular			4
CITt4_2_pos	1	0.19043	0.7202646	-1.25788	1.257882	2	0.347218	CITt4_2	citrate transport via sodium symport	2.00 * Sodium [e] + 1.00 * Citrate [e] --> 2.00 * Sodium [c]	SLC13A2	Transport, extracellular			4
CITt4_4_neg	1	0.19043	0.7202646	-1.25788	1.257882	2	0.347218	CITt4_4	citrate transport via sodium symport	4.00 * Sodium [e] + 1.00 * Citrate [e] --> 4.00 * Sodium [c]	SLC13A5	Transport, extracellular			4
CITt4_4_pos	1	0.19043	0.7202646	-1.21355	1.213555	2	0.347218	CITt4_4	citrate transport via sodium symport	4.00 * Sodium [e] + 1.00 * Citrate [e] --> 4.00 * Sodium [c]	SLC13A5	Transport, extracellular			4
CYSALANaEx_pos	8	0.19043	0.7202646	1.626676	1.626676	1	0.347218	CYSALANaE	L-alanine/L-cysteine Na-dependent exchange (Cys-L in)	1.00 * Sodium [e] + 1.00 * L-alanine [c] + 1.00 * L-cysteine	SLC1A4; SL	Transport, extracellular			4
CYTDt4_pos	1	0.19043	0.7202646	-1.81321	1.813214	2	0.347218	CYTDt4	cytidine transport in via sodium symport	1.00 * Sodium [e] + 1.00 * Cytidine [e] --> 1.00 * Sodium [	SLC28A1; S	Transport, extracellular			4
CYTDt_neg	1	0.19043	0.7202646	-1.81321	1.813214	2	0.347218	CYTDt	cytidine facilated transport in cytosol	1.00 * Cytidine [e] --> 1.00 * Cytidine [c]SLC29A1; SLC29A	SLC29A1; S	Transport, extracellular			4
DHEASt_neg	1	0.19043	0.7202646	-1.79879	1.798793	2	0.347218	DHEASt	dehydroepiandrosterone sulfate transport via bicarbonate countert	1.00 * Bicarbonate [c] + 1.00 * 3beta-hydroxyandrost-5-e	SLCO1A1; S	Transport, extracellular			4
DHEASt_pos	1	0.19043	0.7202646	-1.63257	1.632568	2	0.347218	DHEASt	dehydroepiandrosterone sulfate transport via bicarbonate countert	1.00 * Bicarbonate [c] + 1.00 * 3beta-hydroxyandrost-5-e	SLCO1A1; S	Transport, extracellular			4
ESTRONESt_neg	1	0.19043	0.7202646	-1.79202	1.792024	2	0.347218	ESTRONESt	estrone 3-sulfate transport via bicarbonate countertransport	1.00 * Bicarbonate [c] + 1.00 * estrone 3-sulfate(1-) [e] -->	SLCO1A1; S	Transport, extracellular			4
ESTRONESt_pos	1	0.19043	0.7202646	-1.6324	1.632396	2	0.347218	ESTRONESt	estrone 3-sulfate transport via bicarbonate countertransport	1.00 * Bicarbonate [c] + 1.00 * estrone 3-sulfate(1-) [e] -->	SLCO1A1; S	Transport, extracellular			4
FATP5t_neg	1	0.19043	0.7202646	-1.43663	1.436632	2	0.347218	FATP5t	fatty acid electroneutral transport	1.00 * Sodium [c] + 1.00 * arachidonate [c] --> 1.00 * Sodi	SLC27A5	Transport, extracellular			4
FATP5t_pos	1	0.19043	0.7202646	-1.65516	1.655156	2	0.347218	FATP5t	fatty acid electroneutral transport	1.00 * Sodium [c] + 1.00 * arachidonate [c] --> 1.00 * Sodi	SLC27A5	Transport, extracellular			4
FATP8t_pos	1	0.19043	0.7202646	-1.24228	1.24228	2	0.347218	FATP8t	fatty acid electroneutral transport	1.00 * Sodium [c] + 1.00 * lignocerate [c] --> 1.00 * Sodiur	SLC27A2; S	Transport, extracellular			4
FRUt1r_neg	1	0.19043	0.7202646	-1.56102	1.561021	2	0.347218	FRUt1r	D-fructose transport in via uniport	1.00 * D-Fructose [e] --> 1.00 * D-Fructose [c]SLC2A2; SLC	SLC2A2; SL	Transport, extracellular			4
FRUt1r_pos	1	0.19043	0.7202646	-1.66491	1.664907	2	0.347218	FRUt1r	D-fructose transport in via uniport	1.00 * D-Fructose [e] --> 1.00 * D-Fructose [c]SLC2A2; SLC	SLC2A2; SL	Transport, extracellular			4
FRUt4_neg	1	0.19043	0.7202646	-1.66491	1.664907	2	0.347218	FRUt4	D-fructose transportvia sodium cotransport	1.00 * Sodium [e] + 1.00 * D-Fructose [e] --> 1.00 * Sodiun	SLC5A10; S	Transport, extracellular			4
FRUt4_pos	1	0.19043	0.7202646	-1.56102	1.561021	2	0.347218	FRUt4	D-fructose transportvia sodium cotransport	1.00 * Sodium [e] + 1.00 * D-Fructose [e] --> 1.00 * Sodiun	SLC5A10; S	Transport, extracellular			4
GALt1r_neg	1	0.19043	0.7202646	-1.49646	1.496462	2	0.347218	GALt1r	galactose transport (uniport)	1.00 * D-Galactose [e] --> 1.00 * D-Galactose [c]SLC2A1; SI	SLC2A1; SL	Transport, extracellular			4
GALt1r_pos	1	0.19043	0.7202646	-1.61356	1.613559	2	0.347218	GALt1r	galactose transport (uniport)	1.00 * D-Galactose [e] --> 1.00 * D-Galactose [c]SLC2A1; SI	SLC2A1; SL	Transport, extracellular			4
GALt2_2_neg	1	0.19043	0.7202646	-1.87488	1.874881	2	0.347218	GALt2_2	D-galactose transportvia proton symport	2.00 * proton [e] + 1.00 * D-Galactose [e] --> 2.00 * protor	SLC5A1	Transport, extracellular			4
GALt4_neg	1	0.19043	0.7202646	-1.61356	1.613559	2	0.347218	GALt4	galactose transport via sodium symport	1.00 * Sodium [e] + 1.00 * D-Galactose [e] --> 1.00 * Sodiu	SLC5A10; S	Transport, extracellular			4
GALt4_pos	1	0.19043	0.7202646	-1.49646	1.496462	2	0.347218	GALt4	galactose transport via sodium symport	1.00 * Sodium [e] + 1.00 * D-Galactose [e] --> 1.00 * Sodiu	SLC5A10; S	Transport, extracellular			4
GCHOLAt_neg	1	0.19043	0.7202646	-1.46197	1.461972	2	0.347218	GCHOLAt	glycocholate transport via bicarbonate countertransport	1.00 * Bicarbonate [c] + 1.00 * glycocholate [e] --> 1.00 *	SLCO1A1; S	Transport, extracellular			4
GLCt2_2_neg	1	0.19043	0.7202646	-1.72302	1.723023	2	0.347218	GLCt2_2	D-glucose transport in via proton symport	2.00 * proton [e] + 1.00 * D-glucose [e] --> 2.00 * proton [	SLC5A1	Transport, extracellular			4
GLCt4_neg	1	0.19043	0.7202646	-1.70077	1.700774	2	0.347218	GLCt4	glucose transport via sodium symport	1.00 * Sodium [e] + 1.00 * D-glucose [e] --> 1.00 * Sodium	SLC5A10; S	Transport, extracellular			4
GLNt4_pos	1	0.19043	0.7202646	-1.66853	1.668526	2	0.347218	GLNt4	L-glutamine reversible transport via sodium symport	1.00 * Sodium [e] + 1.00 * L-glutamine [e] --> 1.00 * Sodiu	ACE2; SLC3	Transport, extracellular			4
GLNyLATthc_pos	1	0.19043	0.7202646	-1.45585	1.455852	2	0.347218	GLNyLATth	transport of Glutamine by y+LAT1 or y+LAT2 with co-transporter of h	1.00 * proton [e] + 1.00 * L-glutamine [e] + 1.00 * L-argini	SLC3A2; SL	Transport, extracellular			4
GLYt7_311_r_pos	8	0.19043	0.7202646	1.318925	1.318925	1	0.347218	GLYt7_311	glycine reversible transport via sodium and chloride symport (3:1:1)	3.00 * Sodium [e] + 1.00 * Glycine [e] + 1.00 * Chloride [e]	SLC6A5	Transport, extracellular			4
HCO3_NAt_pos	8	0.19043	0.7202646	0.87311	0.87311	1	0.347218	HCO3_NAt	bicarbonate transport (Na/HCO3 cotransport)	1.00 * Bicarbonate [e] + 1.00 * Sodium [e] --> 1.00 * Bicar	SLC4A7	Transport, extracellular			4
HIStN1_neg	1	0.19043	0.7202646	-1.2026	1.202596	2	0.347218	HIStN1	Histidine transport (Na, H coupled)	1.00 * proton [c] + 2.00 * Sodium [e] + 1.00 * L-histidine [	SLC38A3	Transport, extracellular			4
HISyLATtc_pos	8	0.19043	0.7202646	1.95066	1.95066	1	0.347218	HISyLATtc	Transport of L-Histidine by y+LAT1 or y+LAT2 transporters in small in	1.00 * Sodium [e] + 1.00 * L-argininium(1+) [c] + 1.00 * L-H	SLC3A2; SL	Transport, extracellular			4
LEUKTRC4t_neg	1	0.19043	0.7202646	-1.80401	1.804011	2	0.347218	LEUKTRC4t	leukotriene C4 transport via bicarbonate countertransport	1.00 * Bicarbonate [c] + 1.00 * leukotriene C4(2-) [e] --> 1.	SLCO1B2	Transport, extracellular			4
LEUKTRC4t_pos	1	0.19043	0.7202646	-1.6198	1.619795	2	0.347218	LEUKTRC4t	leukotriene C4 transport via bicarbonate countertransport	1.00 * Bicarbonate [c] + 1.00 * leukotriene C4(2-) [e] --> 1.	SLCO1B2	Transport, extracellular			4
LEUtec_pos	8	0.19043	0.7202646	1.562783	1.562783	1	0.347218	LEUtec	L-leucine transport via diffusion (extracellular to cytosol)	1.00 * L-leucine [e] --> 1.00 * L-leucine [c]SLC43A1; SLC43	SLC43A1; S	Transport, extracellular			4
LEUyLAThtc_pos	1	0.19043	0.7202646	-1.45585	1.455852	2	0.347218	LEUyLAThtc	transport of Leucine by y+LAT1 or y+LAT2 with co-transporter of h in	1.00 * proton [e] + 1.00 * L-argininium(1+) [c] + 1.00 * L-le	SLC3A2; SL	Transport, extracellular			4
LINOFATPtc_pos	1	0.19043	0.7202646	-1.46238	1.462381	2	0.347218	LINOFATPtc	uptake of linoleic acid by the enterocytes	1.00 * ATP [c] + 1.00 * Coenzyme A [c] + 1.00 * linoleate [e	SLC27A4	Transport, extracellular			4
MANt1r_neg	1	0.19043	0.7202646	-1.54813	1.548133	2	0.347218	MANt1r	mannose transport (uniport)	1.00 * D-Mannose [e] --> 1.00 * D-Mannose [c]SLC2A1; SLC	SLC2A1; SL	Transport, extracellular			4
MANt1r_pos	1	0.19043	0.7202646	-1.65385	1.653849	2	0.347218	MANt1r	mannose transport (uniport)	1.00 * D-Mannose [e] --> 1.00 * D-Mannose [c]SLC2A1; SLC	SLC2A1; SL	Transport, extracellular			4
MANt4_neg	1	0.19043	0.7202646	-1.65385	1.653849	2	0.347218	MANt4	D-mannose transport via sodium cotransport	1.00 * Sodium [e] + 1.00 * D-Mannose [e] --> 1.00 * Sodiur	SLC5A10; S	Transport, extracellular			4
MANt4_pos	1	0.19043	0.7202646	-1.54813	1.548133	2	0.347218	MANt4	D-mannose transport via sodium cotransport	1.00 * Sodium [e] + 1.00 * D-Mannose [e] --> 1.00 * Sodiur	SLC5A10; S	Transport, extracellular			4
METyLATthc_pos	1	0.19043	0.7202646	-1.45585	1.455852	2	0.347218	METyLATth	transport of Methionine by y+LAT1 or y+LAT2 with co-transporter of	1.00 * proton [e] + 1.00 * L-argininium(1+) [c] + 1.00 * L-m	SLC3A2; SL	Transport, extracellular			4
NAHCO3_HCLt_neg	8	0.19043	0.7202646	1.577459	1.577459	1	0.347218	NAHCO3_H	bicarbonate transport (HCl/NaHCO3 exchange)	1.00 * proton [c] + 1.00 * Bicarbonate [e] + 1.00 * Sodium	SLC4A10; S	Transport, extracellular			4
NAHCO3_HCLt_pos	8	0.19043	0.7202646	1.606226	1.606226	1	0.347218	NAHCO3_H	bicarbonate transport (HCl/NaHCO3 exchange)	1.00 * proton [c] + 1.00 * Bicarbonate [e] + 1.00 * Sodium	SLC4A10; S	Transport, extracellular			4
NAt_neg	1	0.19043	0.7202646	-1.53876	1.538759	2	0.347218	NAt	sodium transport (uniport)	1.00 * Sodium [e] --> 1.00 * Sodium [c]SLC5A1; SLC5A3; S	SLC5A1; SL	Transport, extracellular			4
NAt_pos	1	0.19043	0.7202646	-1.49541	1.495412	2	0.347218	NAt	sodium transport (uniport)	1.00 * Sodium [e] --> 1.00 * Sodium [c]SLC5A1; SLC5A3; S	SLC5A1; SL	Transport, extracellular			4
PHEtec_pos	8	0.19043	0.7202646	1.686415	1.686415	1	0.347218	PHEtec	L-phenylalanine transport via diffusion (extracellular to cytosol)	1.00 * L-phenylalanine [e] --> 1.00 * L-phenylalanine [c]SL	SLC16A10;	Transport, extracellular			4
PHEyLATthc_pos	1	0.19043	0.7202646	-1.45585	1.455852	2	0.347218	PHEyLATth	transport of Phenylalanine by y+LAT1 or y+LAT2 with co-transporter	1.00 * proton [e] + 1.00 * L-argininium(1+) [c] + 1.00 * L-p	SLC3A2; SL	Transport, extracellular			4
PROSTGD2t_neg	1	0.19043	0.7202646	-1.03616	1.036162	2	0.347218	PROSTGD2	prostaglandin transport via bicarbonate countertransport	1.00 * Bicarbonate [c] + 1.00 * prostaglandin D2(1-) [e] -->	SLCO2A1	Transport, extracellular			4
PROSTGD2t_pos	1	0.19043	0.7202646	-1.02815	1.028152	2	0.347218	PROSTGD2	prostaglandin transport via bicarbonate countertransport	1.00 * Bicarbonate [c] + 1.00 * prostaglandin D2(1-) [e] -->	SLCO2A1	Transport, extracellular			4
PROSTGE1t_neg	1	0.19043	0.7202646	-1.03616	1.036162	2	0.347218	PROSTGE1t	prostaglandin transport via bicarbonate countertransport	1.00 * Bicarbonate [c] + 1.00 * prostaglandin E1(1-) [e] -->	SLCO2A1	Transport, extracellular			4
PROSTGE1t_pos	1	0.19043	0.7202646	-1.02815	1.028152	2	0.347218	PROSTGE1t	prostaglandin transport via bicarbonate countertransport	1.00 * Bicarbonate [c] + 1.00 * prostaglandin E1(1-) [e] -->	SLCO2A1	Transport, extracellular			4
SRTNtu_pos	1	0.19043	0.7202646	-1.29464	1.294635	2	0.347218	SRTNtu	Serotonin uniport	1.00 * serotonin(1+) [e] --> 1.00 * serotonin(1+) [c]SLC22A	SLC22A1; S	Transport, extracellular			4
TCHOLAt_neg	1	0.19043	0.7202646	-1.46104	1.461038	2	0.347218	TCHOLAt	taurocholate transport via bicarbonate countertransport	1.00 * Bicarbonate [c] + 1.00 * taurocholate [e] --> 1.00 *	SLCO1A1; S	Transport, extracellular			4
TYRATB0tc_pos	8	0.19043	0.7202646	1.173188	1.173188	1	0.347218	TYRATB0tc	transport of L-Tyrosine into the intestinal cells by ATB0 transporter	2.00 * Sodium [e] + 1.00 * Chloride [e] + 1.00 * L-tyrosine	SLC6A14	Transport, extracellular			4
UREAt5_neg	1	0.19043	0.7202646	-1.59449	1.594489	2	0.347218	UREAt5	urea, water cotransport	1.00 * Water [e] + 1.00 * Urea [e] --> 1.00 * Water [c] + 1.0	SLC5A1	Transport, extracellular			4
UREAt5_pos	1	0.19043	0.7202646	-1.59449	1.594489	2	0.347218	UREAt5	urea, water cotransport	1.00 * Water [e] + 1.00 * Urea [e] --> 1.00 * Water [c] + 1.0	SLC5A1	Transport, extracellular			4

UREAt_neg	1	0.19043	0.7202646	-1.59449	1.594489	2	0.347218	UREAt	Urea transport via facilitate diffusion	1.00 * Urea [e] --> 1.00 * Urea [c]SLC14A1; SLC14A2; SLC5	SLC14A1; S	Transport, extracellular			4
UREAt_pos	1	0.19043	0.7202646	-1.59449	1.594489	2	0.347218	UREAt	Urea transport via facilitate diffusion	1.00 * Urea [e] --> 1.00 * Urea [c]SLC14A1; SLC14A2; SLC5	SLC14A1; S	Transport, extracellular			4
URIt4_pos	1	0.19043	0.7202646	-1.80803	1.808029	2	0.347218	URIt4	uridine transport in via sodium symport	1.00 * Sodium [e] + 1.00 * Uridine [e] --> 1.00 * Sodium [c	SLC28A1; S	Transport, extracellular			4
URIt_neg	1	0.19043	0.7202646	-1.80803	1.808029	2	0.347218	URIt	uridine facilated transport in cytosol	1.00 * Uridine [e] --> 1.00 * Uridine [c]SLC29A1; SLC29A2	SLC29A1; S	Transport, extracellular			4
r1088_neg	1	0.19043	0.7202646	-1.23609	1.236089	2	0.347218	r1088	Active transport	1.00 * proton [e] + 1.00 * Citrate [e] --> 1.00 * proton [c] +	1.00 * prot	Transport, extracellular			0
r1088_pos	1	0.19043	0.7202646	-1.40635	1.406346	2	0.347218	r1088	Active transport	1.00 * proton [e] + 1.00 * Citrate [e] --> 1.00 * proton [c] +	1.00 * prot	Transport, extracellular			0
r1423_pos	1	0.19043	0.7202646	-1.42433	1.424332	2	0.347218	r1423	Facilitated diffusion	1.00 * hydrogenphosphate [c] --> 1.00 * hydrogenphosph	1.00 * hydr	Transport, extracellular		4.2.1.1	0
r1493_pos	1	0.19043	0.7202646	-1.87454	1.874543	2	0.347218	r1493	Utilized transport	1.00 * Reduced glutathione [c] --> 1.00 * Reduced glutath	1.00 * Redu	Transport, extracellular			0
r1512_pos	1	0.19043	0.7202646	-1.44583	1.445826	2	0.347218	r1512	Utilized transport	1.00 * bilirubin(2-) [e] --> 1.00 * bilirubin(2-) [c]No genes	1.00 * bilir	Transport, extracellular			0
r1544_pos	8	0.19043	0.7202646	1.939651	1.939651	1	0.347218	r1544	Amino Acid-Polyamine-Organocation (APC) TCDB:2.A.3.8.1	1.00 * L-glutamine [c] + 1.00 * Glycine [e] --> 1.00 * L-glut	SLC7A5	Transport, extracellular			0
r1546_neg	8	0.19043	0.7202646	1.776681	1.776681	1	0.347218	r1546	Amino Acid-Polyamine-Organocation (APC) TCDB:2.A.3.8.1	1.00 * Glycine [e] + 1.00 * L-methionine [c] --> 1.00 * Glyc	SLC7A5	Transport, extracellular			0
r1560_neg	8	0.19043	0.7202646	1.810288	1.810288	1	0.347218	r1560	Amino Acid-Polyamine-Organocation (APC) TCDB:2.A.3.8.1	1.00 * L-alanine [e] + 1.00 * L-methionine [c] --> 1.00 * L-a	SLC7A5	Transport, extracellular			0
r1564_neg	8	0.19043	0.7202646	1.810288	1.810288	1	0.347218	r1564	Amino Acid-Polyamine-Organocation (APC) TCDB:2.A.3.8.1	1.00 * L-alanine [e] + 1.00 * L-cysteine [c] --> 1.00 * L-alani	SLC7A5	Transport, extracellular			0
r1567_neg	8	0.19043	0.7202646	1.022377	1.022377	1	0.347218	r1567	Amino Acid-Polyamine-Organocation (APC) TCDB:2.A.3.8.1	1.00 * L-alanine [e] + 1.00 * L-asparagine [c] --> 1.00 * L-ala	SLC7A5	Transport, extracellular			0
r1567_pos	8	0.19043	0.7202646	1.120517	1.120517	1	0.347218	r1567	Amino Acid-Polyamine-Organocation (APC) TCDB:2.A.3.8.1	1.00 * L-alanine [e] + 1.00 * L-asparagine [c] --> 1.00 * L-ala	SLC7A5	Transport, extracellular			0
r1573_neg	8	0.19043	0.7202646	1.776681	1.776681	1	0.347218	r1573	Amino Acid-Polyamine-Organocation (APC) TCDB:2.A.3.8.1	1.00 * L-glutamine [e] + 1.00 * L-methionine [c] --> 1.00 *	SLC7A5	Transport, extracellular			0
r1574_neg	8	0.19043	0.7202646	1.63147	1.63147	1	0.347218	r1574	Amino Acid-Polyamine-Organocation (APC) TCDB:2.A.3.8.1	1.00 * L-glutamine [e] + 1.00 * L-tryptophan [c] --> 1.00 *	SLC7A5	Transport, extracellular			0
r1574_pos	8	0.19043	0.7202646	1.63147	1.63147	1	0.347218	r1574	Amino Acid-Polyamine-Organocation (APC) TCDB:2.A.3.8.1	1.00 * L-glutamine [e] + 1.00 * L-tryptophan [c] --> 1.00 *	SLC7A5	Transport, extracellular			0
r1576_neg	8	0.19043	0.7202646	1.2716	1.2716	1	0.347218	r1576	Amino Acid-Polyamine-Organocation (APC) TCDB:2.A.3.8.1	1.00 * L-glutamine [e] + 1.00 * L-tyrosine [c] --> 1.00 * L-gl	SLC7A5	Transport, extracellular			0
r1576_pos	8	0.19043	0.7202646	1.445123	1.445123	1	0.347218	r1576	Amino Acid-Polyamine-Organocation (APC) TCDB:2.A.3.8.1	1.00 * L-glutamine [e] + 1.00 * L-tyrosine [c] --> 1.00 * L-gl	SLC7A5	Transport, extracellular			0
r1580_neg	8	0.19043	0.7202646	1.173168	1.173168	1	0.347218	r1580	Amino Acid-Polyamine-Organocation (APC) TCDB:2.A.3.8.1	1.00 * L-asparagine [c] + 1.00 * L-glutamine [e] --> 1.00 * L	SLC7A5	Transport, extracellular			0
r1580_pos	8	0.19043	0.7202646	1.173168	1.173168	1	0.347218	r1580	Amino Acid-Polyamine-Organocation (APC) TCDB:2.A.3.8.1	1.00 * L-asparagine [c] + 1.00 * L-glutamine [e] --> 1.00 * L	SLC7A5	Transport, extracellular			0
r1585_neg	8	0.19043	0.7202646	1.776681	1.776681	1	0.347218	r1585	Amino Acid-Polyamine-Organocation (APC) TCDB:2.A.3.8.1	1.00 * L-serine [e] + 1.00 * L-methionine [c] --> 1.00 * L-ser	SLC7A5	Transport, extracellular			0
r1586_neg	8	0.19043	0.7202646	1.63147	1.63147	1	0.347218	r1586	Amino Acid-Polyamine-Organocation (APC) TCDB:2.A.3.8.1	1.00 * L-serine [e] + 1.00 * L-tryptophan [c] --> 1.00 * L-ser	SLC7A5	Transport, extracellular			0
r1586_pos	8	0.19043	0.7202646	1.63147	1.63147	1	0.347218	r1586	Amino Acid-Polyamine-Organocation (APC) TCDB:2.A.3.8.1	1.00 * L-serine [e] + 1.00 * L-tryptophan [c] --> 1.00 * L-ser	SLC7A5	Transport, extracellular			0
r1588_neg	8	0.19043	0.7202646	1.2716	1.2716	1	0.347218	r1588	Amino Acid-Polyamine-Organocation (APC) TCDB:2.A.3.8.1	1.00 * L-serine [e] + 1.00 * L-tyrosine [c] --> 1.00 * L-serine	SLC7A5	Transport, extracellular			0
r1588_pos	8	0.19043	0.7202646	1.032076	1.032076	1	0.347218	r1588	Amino Acid-Polyamine-Organocation (APC) TCDB:2.A.3.8.1	1.00 * L-serine [e] + 1.00 * L-tyrosine [c] --> 1.00 * L-serine	SLC7A5	Transport, extracellular			0
r1597_neg	8	0.19043	0.7202646	1.643382	1.643382	1	0.347218	r1597	Amino Acid-Polyamine-Organocation (APC) TCDB:2.A.3.8.1	1.00 * L-methionine [e] + 1.00 * L-tryptophan [c] --> 1.00	SLC7A5	Transport, extracellular			0
r1598_pos	8	0.19043	0.7202646	1.810288	1.810288	1	0.347218	r1598	Amino Acid-Polyamine-Organocation (APC) TCDB:2.A.3.8.1	1.00 * L-methionine [e] + 1.00 * L-phenylalanine [c] --> 1.0	SLC7A5	Transport, extracellular			0
r1599_neg	8	0.19043	0.7202646	1.278018	1.278018	1	0.347218	r1599	Amino Acid-Polyamine-Organocation (APC) TCDB:2.A.3.8.1	1.00 * L-methionine [e] + 1.00 * L-tyrosine [c] --> 1.00 * L-	SLC7A5	Transport, extracellular			0
r1600_neg	8	0.19043	0.7202646	1.774119	1.774119	1	0.347218	r1600	Amino Acid-Polyamine-Organocation (APC) TCDB:2.A.3.8.1	1.00 * L-cysteine [c] + 1.00 * L-methionine [e] --> 1.00 * L-	SLC7A5	Transport, extracellular			0
r1600_pos	8	0.19043	0.7202646	1.774119	1.774119	1	0.347218	r1600	Amino Acid-Polyamine-Organocation (APC) TCDB:2.A.3.8.1	1.00 * L-cysteine [c] + 1.00 * L-methionine [e] --> 1.00 * L-	SLC7A5	Transport, extracellular			0
r1603_pos	8	0.19043	0.7202646	1.776681	1.776681	1	0.347218	r1603	Amino Acid-Polyamine-Organocation (APC) TCDB:2.A.3.8.1	1.00 * L-asparagine [c] + 1.00 * L-methionine [e] --> 1.00 *	SLC7A5	Transport, extracellular			0
r1605_pos	8	0.19043	0.7202646	1.728661	1.728661	1	0.347218	r1605	Amino Acid-Polyamine-Organocation (APC) TCDB:2.A.3.8.1	1.00 * L-threonine [c] + 1.00 * L-methionine [e] --> 1.00 *	SLC7A5	Transport, extracellular			0
r1606_pos	8	0.19043	0.7202646	1.867029	1.867029	1	0.347218	r1606	Amino Acid-Polyamine-Organocation (APC) TCDB:2.A.3.8.1	1.00 * L-methionine [e] + 1.00 * L-homoserine [c] --> 1.00	SLC7A5	Transport, extracellular			0
r1608_neg	8	0.19043	0.7202646	1.49946	1.49946	1	0.347218	r1608	Amino Acid-Polyamine-Organocation (APC) TCDB:2.A.3.8.1	1.00 * L-tryptophan [e] + 1.00 * L-phenylalanine [c] --> 1.0	SLC7A5	Transport, extracellular			0
r1608_pos	8	0.19043	0.7202646	1.49946	1.49946	1	0.347218	r1608	Amino Acid-Polyamine-Organocation (APC) TCDB:2.A.3.8.1	1.00 * L-tryptophan [e] + 1.00 * L-phenylalanine [c] --> 1.0	SLC7A5	Transport, extracellular			0
r1609_neg	8	0.19043	0.7202646	1.276776	1.276776	1	0.347218	r1609	Amino Acid-Polyamine-Organocation (APC) TCDB:2.A.3.8.1	1.00 * L-tryptophan [e] + 1.00 * L-tyrosine [c] --> 1.00 * L-t	SLC7A5	Transport, extracellular			0
r1609_pos	8	0.19043	0.7202646	1.276776	1.276776	1	0.347218	r1609	Amino Acid-Polyamine-Organocation (APC) TCDB:2.A.3.8.1	1.00 * L-tryptophan [e] + 1.00 * L-tyrosine [c] --> 1.00 * L-t	SLC7A5	Transport, extracellular			0
r1611_neg	8	0.19043	0.7202646	1.643382	1.643382	1	0.347218	r1611	Amino Acid-Polyamine-Organocation (APC) TCDB:2.A.3.8.1	1.00 * L-tryptophan [e] + 1.00 * L-leucine [c] --> 1.00 * L-le	SLC7A5	Transport, extracellular			0
r1611_pos	8	0.19043	0.7202646	1.643382	1.643382	1	0.347218	r1611	Amino Acid-Polyamine-Organocation (APC) TCDB:2.A.3.8.1	1.00 * L-tryptophan [e] + 1.00 * L-leucine [c] --> 1.00 * L-le	SLC7A5	Transport, extracellular			0
r1612_neg	8	0.19043	0.7202646	1.797795	1.797795	1	0.347218	r1612	Amino Acid-Polyamine-Organocation (APC) TCDB:2.A.3.8.1	1.00 * L-tryptophan [e] + 1.00 * L-proline [c] --> 1.00 * L-p	SLC7A5	Transport, extracellular			0
r1612_pos	8	0.19043	0.7202646	1.760981	1.760981	1	0.347218	r1612	Amino Acid-Polyamine-Organocation (APC) TCDB:2.A.3.8.1	1.00 * L-tryptophan [e] + 1.00 * L-proline [c] --> 1.00 * L-p	SLC7A5	Transport, extracellular			0
r1621_neg	8	0.19043	0.7202646	1.487099	1.487099	1	0.347218	r1621	Amino Acid-Polyamine-Organocation (APC) TCDB:2.A.3.8.1	1.00 * L-phenylalanine [e] + 1.00 * L-proline [c] --> 1.00 * L	SLC7A5	Transport, extracellular			0
r1625_neg	8	0.19043	0.7202646	1.170708	1.170708	1	0.347218	r1625	Amino Acid-Polyamine-Organocation (APC) TCDB:2.A.3.8.1	1.00 * L-phenylalanine [e] + 1.00 * L-homoserine [c] --> 1.0	SLC7A5	Transport, extracellular			0
r1625_pos	8	0.19043	0.7202646	1.170708	1.170708	1	0.347218	r1625	Amino Acid-Polyamine-Organocation (APC) TCDB:2.A.3.8.1	1.00 * L-phenylalanine [e] + 1.00 * L-homoserine [c] --> 1.0	SLC7A5	Transport, extracellular			0
r1628_neg	8	0.19043	0.7202646	1.037064	1.037064	1	0.347218	r1628	Amino Acid-Polyamine-Organocation (APC) TCDB:2.A.3.8.1	1.00 * L-tyrosine [e] + 1.00 * L-leucine [c] --> 1.00 * L-leuci	SLC7A5	Transport, extracellular			0
r1628_pos	8	0.19043	0.7202646	1.278018	1.278018	1	0.347218	r1628	Amino Acid-Polyamine-Organocation (APC) TCDB:2.A.3.8.1	1.00 * L-tyrosine [e] + 1.00 * L-leucine [c] --> 1.00 * L-leuci	SLC7A5	Transport, extracellular			0
r1630_neg	8	0.19043	0.7202646	1.445123	1.445123	1	0.347218	r1630	Amino Acid-Polyamine-Organocation (APC) TCDB:2.A.3.8.1	1.00 * L-asparagine [c] + 1.00 * L-tyrosine [e] --> 1.00 * L-a	SLC7A5	Transport, extracellular			0
r1630_pos	8	0.19043	0.7202646	1.2716	1.2716	1	0.347218	r1630	Amino Acid-Polyamine-Organocation (APC) TCDB:2.A.3.8.1	1.00 * L-asparagine [c] + 1.00 * L-tyrosine [e] --> 1.00 * L-a	SLC7A5	Transport, extracellular			0
r1631_neg	8	0.19043	0.7202646	1.037064	1.037064	1	0.347218	r1631	Amino Acid-Polyamine-Organocation (APC) TCDB:2.A.3.8.1	1.00 * L-tyrosine [e] + 1.00 * L-valine [c] --> 1.00 * L-valine	SLC7A5	Transport, extracellular			0
r1631_pos	8	0.19043	0.7202646	1.278018	1.278018	1	0.347218	r1631	Amino Acid-Polyamine-Organocation (APC) TCDB:2.A.3.8.1	1.00 * L-tyrosine [e] + 1.00 * L-valine [c] --> 1.00 * L-valine	SLC7A5	Transport, extracellular			0
r1633_neg	8	0.19043	0.7202646	1.032076	1.032076	1	0.347218	r1633	Amino Acid-Polyamine-Organocation (APC) TCDB:2.A.3.8.1	1.00 * L-tyrosine [e] + 1.00 * L-homoserine [c] --> 1.00 * L-	SLC7A5	Transport, extracellular			0
r1633_pos	8	0.19043	0.7202646	1.2716	1.2716	1	0.347218	r1633	Amino Acid-Polyamine-Organocation (APC) TCDB:2.A.3.8.1	1.00 * L-tyrosine [e] + 1.00 * L-homoserine [c] --> 1.00 * L-	SLC7A5	Transport, extracellular			0
r1634_neg	8	0.19043	0.7202646	1.037064	1.037064	1	0.347218	r1634	Amino Acid-Polyamine-Organocation (APC) TCDB:2.A.3.8.1	1.00 * L-tyrosine [e] + 1.00 * L-isoleucine [c] --> 1.00 * L-is	SLC7A5	Transport, extracellular			0
r1634_pos	8	0.19043	0.7202646	1.278018	1.278018	1	0.347218	r1634	Amino Acid-Polyamine-Organocation (APC) TCDB:2.A.3.8.1	1.00 * L-tyrosine [e] + 1.00 * L-isoleucine [c] --> 1.00 * L-is	SLC7A5	Transport, extracellular			0
r1637_neg	8	0.19043	0.7202646	1.787212	1.787212	1	0.347218	r1637	Amino Acid-Polyamine-Organocation (APC) TCDB:2.A.3.8.1	1.00 * L-asparagine [c] + 1.00 * L-cysteine [e] --> 1.00 * L-a	SLC7A5	Transport, extracellular			0
r1637_pos	8	0.19043	0.7202646	1.776681	1.776681	1	0.347218	r1637	Amino Acid-Polyamine-Organocation (APC) TCDB:2.A.3.8.1	1.00 * L-asparagine [c] + 1.00 * L-cysteine [e] --> 1.00 * L-a	SLC7A5	Transport, extracellular			0
r1640_pos	8	0.19043	0.7202646	1.867029	1.867029	1	0.347218	r1640	Amino Acid-Polyamine-Organocation (APC) TCDB:2.A.3.8.1	1.00 * L-cysteine [e] + 1.00 * L-homoserine [c] --> 1.00 * L-	SLC7A5	Transport, extracellular			0
r2009_pos	1	0.19043	0.7202646	-1.45585	1.455852	2	0.347218	r2009	Amino Acid-Polyamine-Organocation (APC) TCDB:2.A.3.8.15	1.00 * L-alanine [c] + 1.00 * L-argininium(1+) [e] --> 1.00 *	SLC7A9	Transport, extracellular			0
r2010_pos	1	0.19043	0.7202646	-1.45585	1.455852	2	0.347218	r2010	Amino Acid-Polyamine-Organocation (APC) TCDB:2.A.3.8.15	1.00 * L-glutamine [c] + 1.00 * L-argininium(1+) [e] --> 1.0	SLC7A9	Transport, extracellular			0
r2012_pos	1	0.19043	0.7202646	-1.45585	1.455852	2	0.347218	r2012	Amino Acid-Polyamine-Organocation (APC) TCDB:2.A.3.8.15	1.00 * L-argininium(1+) [e] + 1.00 * L-methionine [c] --> 1.	SLC7A9	Transport, extracellular			0
r2014_pos	1	0.19043	0.7202646	-1.45585	1.455852	2	0.347218	r2014	Amino Acid-Polyamine-Organocation (APC) TCDB:2.A.3.8.15	1.00 * L-argininium(1+) [e] + 1.00 * L-phenylalanine [c] -->	SLC7A9	Transport, extracellular			0
r2017_pos	1	0.19043	0.7202646	-1.45585	1.455852	2	0.347218	r2017	Amino Acid-Polyamine-Organocation (APC) TCDB:2.A.3.8.15	1.00 * L-argininium(1+) [e] + 1.00 * L-leucine [c] --> 1.00 *	SLC7A9	Transport, extracellular			0
r2073_pos	8	0.19043	0.7202646	1.686657	1.686657	1	0.347218	r2073	Zinc (Zn2+)-Iron (Fe2+) Permease (ZIP), TCDB:2.A.55.2.3	1.00 * proton [e] + 1.00 * Zinc [e] --> 1.00 * proton [c] + 1.	SLC11A1	Transport, extracellular			0
r2093_pos	8	0.19043	0.7202646	1.401522	1.401522	1	0.347218	r2093	Major Facilitator(MFS) TCDB:2.A.1.13.1	1.00 * 2-Oxoglutarate [c] + 1.00 * pyruvate [e] --> 1.00 * p	SLC16A1	Transport, extracellular			0
r2104_neg	8	0.19043	0.7202646	1.387863	1.387863	1	0.347218	r2104	Major Facilitator(MFS) TCDB:2.A.1.13.1	1.00 * acetoacetate [c] + 1.00 * 2-Oxoglutarate [e] --> 1.00	SLC16A1	Transport, extracellular			0
r2105_neg	8	0.19043	0.7202646	1.401522	1.401522	1	0.347218	r2105	Major Facilitator(MFS) TCDB:2.A.1.13.1	1.00 * 2-Oxoglutarate [e] + 1.00 * (S)-lactate [c] --> 1.00 *	SLC16A1	Transport, extracellular			0
r2107_neg	8	0.19043	0.7202646	1.748452	1.748452	1	0.347218	r2107	Major Facilitator(MFS) TCDB:2.A.1.13.1	1.00 * 2-Oxoglutarate [e] + 1.00 * all-trans-retinoate [c] --	SLC16A1	Transport, extracellular			0
r2109_neg	8	0.19043	0.7202646	1.401522	1.401522	1	0.347218	r2109	Major Facilitator(MFS) TCDB:2.A.1.13.1	1.00 * 2-Hydroxybutyrate [c] + 1.00 * 2-Oxoglutarate [e] -	SLC16A1	Transport, extracellular			0
r2198_neg	1	0.19043	0.7202646	-1.87376	1.873757	2	0.347218	r2198	Resistance-Nodulation-Cell Division (RND) TCDB:2.A.60.1.5	1.00 * Bicarbonate [c] + 1.00 * Reduced glutathione [c] +	SLCO1B2	Transport, extracellular			0
r2198_pos	1	0.19043	0.7202646	-1.87376	1.873757	2	0.347218	r2198	Resistance-Nodulation-Cell Division (RND) TCDB:2.A.60.1.5	1.00 * Bicarbonate [c] + 1.00 * Reduced glutathione [c] +	SLCO1B2	Transport, extracellular			0
r2199_neg	1	0.19043	0.7202646	-1.86342	1.863421	2	0.347218	r2199	Resistance-Nodulation-Cell Division (RND) TCDB:2.A.60.1.5	1.00 * Bicarbonate [c] + 1.00 * bis(beta-glucosyluronate)b	SLCO1B2	Transport, extracellular			0
r2199_pos	1	0.19043	0.7202646	-1.86342	1.863421	2	0.347218	r2199	Resistance-Nodulation-Cell Division (RND) TCDB:2.A.60.1.5	1.00 * Bicarbonate [c] + 1.00 * bis(beta-glucosyluronate)b	SLCO1B2	Transport, extracellular			0
r2200_neg	1	0.19043	0.7202646	-1.85065	1.850655	2	0.347218	r2200	Resistance-Nodulation-Cell Division (RND) TCDB:2.A.60.1.5	1.00 * Bicarbonate [c] + 1.00 * Reduced glutathione [c] +	SLCO1B2	Transport, extracellular			0
r2200_pos	1	0.19043	0.7202646	-1.85065	1.850655	2	0.347218	r2200	Resistance-Nodulation-Cell Division (RND) TCDB:2.A.60.1.5	1.00 * Bicarbonate [c] + 1.00 * Reduced glutathione [c] +	SLCO1B2	Transport, extracellular			0
r2201_neg	1	0.19043	0.7202646	-1.85065	1.850655	2	0.347218	r2201	Resistance-Nodulation-Cell Division (RND) TCDB:2.A.60.1.5	1.00 * Bicarbonate [c] + 1.00 * Reduced glutathione [c] +	SLCO1B2	Transport, extracellular			0
r2201_pos	1	0.19043	0.7202646	-1.85065	1.850655	2	0.347218	r2201	Resistance-Nodulation-Cell Division (RND) TCDB:2.A.60.1.5	1.00 * Bicarbonate [c] + 1.00 * Reduced glutathione [c] +	SLCO1B2	Transport, extracellular			0
r2202_neg	1	0.19043	0.7202646	-1.87376	1.873757	2	0.347218	r2202	Resistance-Nodulation-Cell Division (RND) TCDB:2.A.60.1.5	1.00 * Bicarbonate [c] + 1.00 * mono(glucosyluronic acid	SLCO1B2	Transport, extracellular			0
r2202_pos	1	0.19043	0.7202646	-1.87376	1.873757	2	0.347218	r2202	Resistance-Nodulation-Cell Division (RND) TCDB:2.A.60.1.5	1.00 * Bicarbonate [c] + 1.00 * mono(glucosyluronic acid	SLCO1B2	Transport, extracellular			0
r2221_neg	1	0.19043	0.7202646	-1.91086	1.910864	2	0.347218	r2221	Resistance-Nodulation-Cell Division (RND) TCDB:2.A.60.1.5	1.00 * Bicarbonate [c] + 1.00 * leukotriene C4(2-) [e] + 1.0	SLCO1B2	Transport, extracellular			0
r2221_pos	1	0.19043	0.7202646	-1.91086	1.910864	2	0.347218	r2221	Resistance-Nodulation-Cell Division (RND) TCDB:2.A.60.1.5	1.00 * Bicarbonate [c] + 1.00 * leukotriene C4(2-) [e] + 1.0	SLCO1B2	Transport, extracellular			0
r2222_neg	1	0.19043	0.7202646	-1.91086	1.910864	2	0.347218	r2222	Resistance-Nodulation-Cell Division (RND) TCDB:2.A.60.1.5	1.00 * Bicarbonate [c] + 1.00 * leukotriene C4(2-) [e] + 1.0	SLCO1B2	Transport, extracellular			0

r2222_pos	1	0.19043	0.7202646	-1.91086	1.910864	2	0.347218	r2222	Resistance-Nodulation-Cell Division (RND) TCDB:2.A.60.1.5	1.00 * Bicarbonate [c] + 1.00 * leukotriene C4(2-) [e] + 1.0	SLCO1B2	Transport, extracellular			0
r2223_neg	1	0.19043	0.7202646	-1.91086	1.910864	2	0.347218	r2223	Resistance-Nodulation-Cell Division (RND) TCDB:2.A.60.1.5	1.00 * Bicarbonate [c] + 1.00 * leukotriene C4(2-) [e] + 1.0	SLCO1B2	Transport, extracellular			0
r2223_pos	1	0.19043	0.7202646	-1.91086	1.910864	2	0.347218	r2223	Resistance-Nodulation-Cell Division (RND) TCDB:2.A.60.1.5	1.00 * Bicarbonate [c] + 1.00 * leukotriene C4(2-) [e] + 1.0	SLCO1B2	Transport, extracellular			0
r2224_neg	1	0.19043	0.7202646	-1.85338	1.853379	2	0.347218	r2224	Resistance-Nodulation-Cell Division (RND) TCDB:2.A.60.1.5	1.00 * Bicarbonate [c] + 1.00 * estrone 3-sulfate(1-) [e] + 1	SLCO1B2	Transport, extracellular			0
r2224_pos	1	0.19043	0.7202646	-1.85338	1.853379	2	0.347218	r2224	Resistance-Nodulation-Cell Division (RND) TCDB:2.A.60.1.5	1.00 * Bicarbonate [c] + 1.00 * estrone 3-sulfate(1-) [e] + 1	SLCO1B2	Transport, extracellular			0
r2225_neg	1	0.19043	0.7202646	-1.85338	1.853379	2	0.347218	r2225	Resistance-Nodulation-Cell Division (RND) TCDB:2.A.60.1.5	1.00 * Bicarbonate [c] + 1.00 * estrone 3-sulfate(1-) [e] + 1	SLCO1B2	Transport, extracellular			0
r2225_pos	1	0.19043	0.7202646	-1.85338	1.853379	2	0.347218	r2225	Resistance-Nodulation-Cell Division (RND) TCDB:2.A.60.1.5	1.00 * Bicarbonate [c] + 1.00 * estrone 3-sulfate(1-) [e] + 1	SLCO1B2	Transport, extracellular			0
r2226_neg	1	0.19043	0.7202646	-1.85338	1.853379	2	0.347218	r2226	Resistance-Nodulation-Cell Division (RND) TCDB:2.A.60.1.5	1.00 * Bicarbonate [c] + 1.00 * estrone 3-sulfate(1-) [e] + 1	SLCO1B2	Transport, extracellular			0
r2226_pos	1	0.19043	0.7202646	-1.85338	1.853379	2	0.347218	r2226	Resistance-Nodulation-Cell Division (RND) TCDB:2.A.60.1.5	1.00 * Bicarbonate [c] + 1.00 * estrone 3-sulfate(1-) [e] + 1	SLCO1B2	Transport, extracellular			0
r2227_neg	1	0.19043	0.7202646	-1.85338	1.853379	2	0.347218	r2227	Resistance-Nodulation-Cell Division (RND) TCDB:2.A.60.1.5	1.00 * Bicarbonate [c] + 1.00 * 3beta-hydroxyandrost-5-e	SLCO1B2	Transport, extracellular			0
r2227_pos	1	0.19043	0.7202646	-1.85338	1.853379	2	0.347218	r2227	Resistance-Nodulation-Cell Division (RND) TCDB:2.A.60.1.5	1.00 * Bicarbonate [c] + 1.00 * 3beta-hydroxyandrost-5-e	SLCO1B2	Transport, extracellular			0
r2228_neg	1	0.19043	0.7202646	-1.85338	1.853379	2	0.347218	r2228	Resistance-Nodulation-Cell Division (RND) TCDB:2.A.60.1.5	1.00 * Bicarbonate [c] + 1.00 * 3beta-hydroxyandrost-5-e	SLCO1B2	Transport, extracellular			0
r2228_pos	1	0.19043	0.7202646	-1.85338	1.853379	2	0.347218	r2228	Resistance-Nodulation-Cell Division (RND) TCDB:2.A.60.1.5	1.00 * Bicarbonate [c] + 1.00 * 3beta-hydroxyandrost-5-e	SLCO1B2	Transport, extracellular			0
r2229_neg	1	0.19043	0.7202646	-1.85338	1.853379	2	0.347218	r2229	Resistance-Nodulation-Cell Division (RND) TCDB:2.A.60.1.5	1.00 * Bicarbonate [c] + 1.00 * 3beta-hydroxyandrost-5-e	SLCO1B2	Transport, extracellular			0
r2229_pos	1	0.19043	0.7202646	-1.85338	1.853379	2	0.347218	r2229	Resistance-Nodulation-Cell Division (RND) TCDB:2.A.60.1.5	1.00 * Bicarbonate [c] + 1.00 * 3beta-hydroxyandrost-5-e	SLCO1B2	Transport, extracellular			0
r2230_neg	1	0.19043	0.7202646	-1.88783	1.887828	2	0.347218	r2230	Resistance-Nodulation-Cell Division (RND) TCDB:2.A.60.1.5	1.00 * Bicarbonate [c] + 1.00 * bis(beta-glucosyluronate)	SLCO1B2	Transport, extracellular			0
r2230_pos	1	0.19043	0.7202646	-1.88783	1.887828	2	0.347218	r2230	Resistance-Nodulation-Cell Division (RND) TCDB:2.A.60.1.5	1.00 * Bicarbonate [c] + 1.00 * bis(beta-glucosyluronate)	SLCO1B2	Transport, extracellular			0
r2231_neg	1	0.19043	0.7202646	-1.88783	1.887828	2	0.347218	r2231	Resistance-Nodulation-Cell Division (RND) TCDB:2.A.60.1.5	1.00 * Bicarbonate [c] + 1.00 * bis(beta-glucosyluronate)	SLCO1B2	Transport, extracellular			0
r2231_pos	1	0.19043	0.7202646	-1.88783	1.887828	2	0.347218	r2231	Resistance-Nodulation-Cell Division (RND) TCDB:2.A.60.1.5	1.00 * Bicarbonate [c] + 1.00 * bis(beta-glucosyluronate)	SLCO1B2	Transport, extracellular			0
r2232_neg	1	0.19043	0.7202646	-1.88783	1.887828	2	0.347218	r2232	Resistance-Nodulation-Cell Division (RND) TCDB:2.A.60.1.5	1.00 * Bicarbonate [c] + 1.00 * bis(beta-glucosyluronate)	SLCO1B2	Transport, extracellular			0
r2232_pos	1	0.19043	0.7202646	-1.88783	1.887828	2	0.347218	r2232	Resistance-Nodulation-Cell Division (RND) TCDB:2.A.60.1.5	1.00 * Bicarbonate [c] + 1.00 * bis(beta-glucosyluronate)	SLCO1B2	Transport, extracellular			0
r2233_neg	1	0.19043	0.7202646	-1.85065	1.850655	2	0.347218	r2233	Resistance-Nodulation-Cell Division (RND) TCDB:2.A.60.1.5	1.00 * Bicarbonate [c] + 1.00 * Leukotriene D4 [e] + 1.00 *	SLCO1B2	Transport, extracellular			0
r2233_pos	1	0.19043	0.7202646	-1.85065	1.850655	2	0.347218	r2233	Resistance-Nodulation-Cell Division (RND) TCDB:2.A.60.1.5	1.00 * Bicarbonate [c] + 1.00 * Leukotriene D4 [e] + 1.00 *	SLCO1B2	Transport, extracellular			0
r2234_neg	1	0.19043	0.7202646	-1.85065	1.850655	2	0.347218	r2234	Resistance-Nodulation-Cell Division (RND) TCDB:2.A.60.1.5	1.00 * Bicarbonate [c] + 1.00 * Leukotriene D4 [e] + 1.00 *	SLCO1B2	Transport, extracellular			0
r2234_pos	1	0.19043	0.7202646	-1.85065	1.850655	2	0.347218	r2234	Resistance-Nodulation-Cell Division (RND) TCDB:2.A.60.1.5	1.00 * Bicarbonate [c] + 1.00 * Leukotriene D4 [e] + 1.00 *	SLCO1B2	Transport, extracellular			0
r2235_neg	1	0.19043	0.7202646	-1.85065	1.850655	2	0.347218	r2235	Resistance-Nodulation-Cell Division (RND) TCDB:2.A.60.1.5	1.00 * Bicarbonate [c] + 1.00 * Leukotriene D4 [e] + 1.00 *	SLCO1B2	Transport, extracellular			0
r2235_pos	1	0.19043	0.7202646	-1.85065	1.850655	2	0.347218	r2235	Resistance-Nodulation-Cell Division (RND) TCDB:2.A.60.1.5	1.00 * Bicarbonate [c] + 1.00 * Leukotriene D4 [e] + 1.00 *	SLCO1B2	Transport, extracellular			0
r2236_neg	1	0.19043	0.7202646	-1.85065	1.850655	2	0.347218	r2236	Resistance-Nodulation-Cell Division (RND) TCDB:2.A.60.1.5	1.00 * Bicarbonate [c] + 1.00 * glutathionyl-leuc4 [c] + 1.0	SLCO1B2	Transport, extracellular			0
r2236_pos	1	0.19043	0.7202646	-1.85065	1.850655	2	0.347218	r2236	Resistance-Nodulation-Cell Division (RND) TCDB:2.A.60.1.5	1.00 * Bicarbonate [c] + 1.00 * glutathionyl-leuc4 [c] + 1.0	SLCO1B2	Transport, extracellular			0
r2237_neg	1	0.19043	0.7202646	-1.85065	1.850655	2	0.347218	r2237	Resistance-Nodulation-Cell Division (RND) TCDB:2.A.60.1.5	1.00 * Bicarbonate [c] + 1.00 * S-glutathionyl-2-4-dinitro	SLCO1B2	Transport, extracellular			0
r2237_pos	1	0.19043	0.7202646	-1.85065	1.850655	2	0.347218	r2237	Resistance-Nodulation-Cell Division (RND) TCDB:2.A.60.1.5	1.00 * Bicarbonate [c] + 1.00 * S-glutathionyl-2-4-dinitro	SLCO1B2	Transport, extracellular			0
r2238_neg	1	0.19043	0.7202646	-1.85065	1.850655	2	0.347218	r2238	Resistance-Nodulation-Cell Division (RND) TCDB:2.A.60.1.5	1.00 * Bicarbonate [c] + 1.00 * S-glutathionyl-ethacrynic-	SLCO1B2	Transport, extracellular			0
r2238_pos	1	0.19043	0.7202646	-1.85065	1.850655	2	0.347218	r2238	Resistance-Nodulation-Cell Division (RND) TCDB:2.A.60.1.5	1.00 * Bicarbonate [c] + 1.00 * S-glutathionyl-ethacrynic-	SLCO1B2	Transport, extracellular			0
r2239_neg	1	0.19043	0.7202646	-1.85065	1.850655	2	0.347218	r2239	Resistance-Nodulation-Cell Division (RND) TCDB:2.A.60.1.5	1.00 * Bicarbonate [c] + 1.00 * glutathionyl-leuc4 [c] + 1.0	SLCO1B2	Transport, extracellular			0
r2239_pos	1	0.19043	0.7202646	-1.85065	1.850655	2	0.347218	r2239	Resistance-Nodulation-Cell Division (RND) TCDB:2.A.60.1.5	1.00 * Bicarbonate [c] + 1.00 * glutathionyl-leuc4 [c] + 1.0	SLCO1B2	Transport, extracellular			0
r2240_neg	1	0.19043	0.7202646	-1.85065	1.850655	2	0.347218	r2240	Resistance-Nodulation-Cell Division (RND) TCDB:2.A.60.1.5	1.00 * Bicarbonate [c] + 1.00 * S-glutathionyl-2-4-dinitro	SLCO1B2	Transport, extracellular			0
r2240_pos	1	0.19043	0.7202646	-1.85065	1.850655	2	0.347218	r2240	Resistance-Nodulation-Cell Division (RND) TCDB:2.A.60.1.5	1.00 * Bicarbonate [c] + 1.00 * S-glutathionyl-2-4-dinitro	SLCO1B2	Transport, extracellular			0
r2241_neg	1	0.19043	0.7202646	-1.85065	1.850655	2	0.347218	r2241	Resistance-Nodulation-Cell Division (RND) TCDB:2.A.60.1.5	1.00 * Bicarbonate [c] + 1.00 * S-glutathionyl-ethacrynic-	SLCO1B2	Transport, extracellular			0
r2241_pos	1	0.19043	0.7202646	-1.85065	1.850655	2	0.347218	r2241	Resistance-Nodulation-Cell Division (RND) TCDB:2.A.60.1.5	1.00 * Bicarbonate [c] + 1.00 * S-glutathionyl-ethacrynic-	SLCO1B2	Transport, extracellular			0
r2242_neg	1	0.19043	0.7202646	-1.85065	1.850655	2	0.347218	r2242	Resistance-Nodulation-Cell Division (RND) TCDB:2.A.60.1.5	1.00 * Bicarbonate [c] + 1.00 * leukotriene A4(1-) [e] + 1.0	SLCO1B2	Transport, extracellular			0
r2242_pos	1	0.19043	0.7202646	-1.85065	1.850655	2	0.347218	r2242	Resistance-Nodulation-Cell Division (RND) TCDB:2.A.60.1.5	1.00 * Bicarbonate [c] + 1.00 * leukotriene A4(1-) [e] + 1.0	SLCO1B2	Transport, extracellular			0
r2243_neg	1	0.19043	0.7202646	-1.85065	1.850655	2	0.347218	r2243	Resistance-Nodulation-Cell Division (RND) TCDB:2.A.60.1.5	1.00 * Bicarbonate [c] + 1.00 * leukotriene A4(1-) [e] + 1.0	SLCO1B2	Transport, extracellular			0
r2243_pos	1	0.19043	0.7202646	-1.85065	1.850655	2	0.347218	r2243	Resistance-Nodulation-Cell Division (RND) TCDB:2.A.60.1.5	1.00 * Bicarbonate [c] + 1.00 * leukotriene A4(1-) [e] + 1.0	SLCO1B2	Transport, extracellular			0
r2244_neg	1	0.19043	0.7202646	-1.85065	1.850655	2	0.347218	r2244	Resistance-Nodulation-Cell Division (RND) TCDB:2.A.60.1.5	1.00 * Bicarbonate [c] + 1.00 * leukotriene A4(1-) [e] + 1.0	SLCO1B2	Transport, extracellular			0
r2244_pos	1	0.19043	0.7202646	-1.85065	1.850655	2	0.347218	r2244	Resistance-Nodulation-Cell Division (RND) TCDB:2.A.60.1.5	1.00 * Bicarbonate [c] + 1.00 * leukotriene A4(1-) [e] + 1.0	SLCO1B2	Transport, extracellular			0
r2245_neg	1	0.19043	0.7202646	-1.85065	1.850655	2	0.347218	r2245	Resistance-Nodulation-Cell Division (RND) TCDB:2.A.60.1.5	1.00 * Bicarbonate [c] + 1.00 * leukotriene B4(1-) [e] + 1.0	SLCO1B2	Transport, extracellular			0
r2245_pos	1	0.19043	0.7202646	-1.85065	1.850655	2	0.347218	r2245	Resistance-Nodulation-Cell Division (RND) TCDB:2.A.60.1.5	1.00 * Bicarbonate [c] + 1.00 * leukotriene B4(1-) [e] + 1.0	SLCO1B2	Transport, extracellular			0
r2246_neg	1	0.19043	0.7202646	-1.85065	1.850655	2	0.347218	r2246	Resistance-Nodulation-Cell Division (RND) TCDB:2.A.60.1.5	1.00 * Bicarbonate [c] + 1.00 * leukotriene B4(1-) [e] + 1.0	SLCO1B2	Transport, extracellular			0
r2246_pos	1	0.19043	0.7202646	-1.85065	1.850655	2	0.347218	r2246	Resistance-Nodulation-Cell Division (RND) TCDB:2.A.60.1.5	1.00 * Bicarbonate [c] + 1.00 * leukotriene B4(1-) [e] + 1.0	SLCO1B2	Transport, extracellular			0
r2247_neg	1	0.19043	0.7202646	-1.85065	1.850655	2	0.347218	r2247	Resistance-Nodulation-Cell Division (RND) TCDB:2.A.60.1.5	1.00 * Bicarbonate [c] + 1.00 * leukotriene B4(1-) [e] + 1.0	SLCO1B2	Transport, extracellular			0
r2247_pos	1	0.19043	0.7202646	-1.85065	1.850655	2	0.347218	r2247	Resistance-Nodulation-Cell Division (RND) TCDB:2.A.60.1.5	1.00 * Bicarbonate [c] + 1.00 * leukotriene B4(1-) [e] + 1.0	SLCO1B2	Transport, extracellular			0
r2248_neg	1	0.19043	0.7202646	-1.85065	1.850655	2	0.347218	r2248	Resistance-Nodulation-Cell Division (RND) TCDB:2.A.60.1.5	1.00 * Bicarbonate [c] + 1.00 * leukotriene E4(1-) [e] + 1.0	SLCO1B2	Transport, extracellular			0
r2248_pos	1	0.19043	0.7202646	-1.85065	1.850655	2	0.347218	r2248	Resistance-Nodulation-Cell Division (RND) TCDB:2.A.60.1.5	1.00 * Bicarbonate [c] + 1.00 * leukotriene E4(1-) [e] + 1.0	SLCO1B2	Transport, extracellular			0
r2249_neg	1	0.19043	0.7202646	-1.85065	1.850655	2	0.347218	r2249	Resistance-Nodulation-Cell Division (RND) TCDB:2.A.60.1.5	1.00 * Bicarbonate [c] + 1.00 * leukotriene E4(1-) [e] + 1.0	SLCO1B2	Transport, extracellular			0
r2249_pos	1	0.19043	0.7202646	-1.85065	1.850655	2	0.347218	r2249	Resistance-Nodulation-Cell Division (RND) TCDB:2.A.60.1.5	1.00 * Bicarbonate [c] + 1.00 * leukotriene E4(1-) [e] + 1.0	SLCO1B2	Transport, extracellular			0
r2250_neg	1	0.19043	0.7202646	-1.85065	1.850655	2	0.347218	r2250	Resistance-Nodulation-Cell Division (RND) TCDB:2.A.60.1.5	1.00 * Bicarbonate [c] + 1.00 * leukotriene E4(1-) [e] + 1.0	SLCO1B2	Transport, extracellular			0
r2250_pos	1	0.19043	0.7202646	-1.85065	1.850655	2	0.347218	r2250	Resistance-Nodulation-Cell Division (RND) TCDB:2.A.60.1.5	1.00 * Bicarbonate [c] + 1.00 * leukotriene E4(1-) [e] + 1.0	SLCO1B2	Transport, extracellular			0
r2251_neg	1	0.19043	0.7202646	-1.85065	1.850655	2	0.347218	r2251	Resistance-Nodulation-Cell Division (RND) TCDB:2.A.60.1.5	1.00 * Bicarbonate [c] + 1.00 * leukotriene F4 [e] + 1.00 *	SLCO1B2	Transport, extracellular			0
r2251_pos	1	0.19043	0.7202646	-1.85065	1.850655	2	0.347218	r2251	Resistance-Nodulation-Cell Division (RND) TCDB:2.A.60.1.5	1.00 * Bicarbonate [c] + 1.00 * leukotriene F4 [e] + 1.00 *	SLCO1B2	Transport, extracellular			0
r2252_neg	1	0.19043	0.7202646	-1.85065	1.850655	2	0.347218	r2252	Resistance-Nodulation-Cell Division (RND) TCDB:2.A.60.1.5	1.00 * Bicarbonate [c] + 1.00 * leukotriene F4 [e] + 1.00 *	SLCO1B2	Transport, extracellular			0
r2252_pos	1	0.19043	0.7202646	-1.85065	1.850655	2	0.347218	r2252	Resistance-Nodulation-Cell Division (RND) TCDB:2.A.60.1.5	1.00 * Bicarbonate [c] + 1.00 * leukotriene F4 [e] + 1.00 *	SLCO1B2	Transport, extracellular			0
r2253_neg	1	0.19043	0.7202646	-1.85065	1.850655	2	0.347218	r2253	Resistance-Nodulation-Cell Division (RND) TCDB:2.A.60.1.5	1.00 * Bicarbonate [c] + 1.00 * leukotriene F4 [e] + 1.00 *	SLCO1B2	Transport, extracellular			0
r2253_pos	1	0.19043	0.7202646	-1.85065	1.850655	2	0.347218	r2253	Resistance-Nodulation-Cell Division (RND) TCDB:2.A.60.1.5	1.00 * Bicarbonate [c] + 1.00 * leukotriene F4 [e] + 1.00 *	SLCO1B2	Transport, extracellular			0
r2254_neg	1	0.19043	0.7202646	-1.91086	1.910864	2	0.347218	r2254	Resistance-Nodulation-Cell Division (RND) TCDB:2.A.60.1.5	1.00 * Bicarbonate [c] + 1.00 * mono(glucosyluronic acid	SLCO1B2	Transport, extracellular			0
r2254_pos	1	0.19043	0.7202646	-1.91086	1.910864	2	0.347218	r2254	Resistance-Nodulation-Cell Division (RND) TCDB:2.A.60.1.5	1.00 * Bicarbonate [c] + 1.00 * mono(glucosyluronic acid	SLCO1B2	Transport, extracellular			0
r2255_neg	1	0.19043	0.7202646	-1.91086	1.910864	2	0.347218	r2255	Resistance-Nodulation-Cell Division (RND) TCDB:2.A.60.1.5	1.00 * Bicarbonate [c] + 1.00 * mono(glucosyluronic acid	SLCO1B2	Transport, extracellular			0
r2255_pos	1	0.19043	0.7202646	-1.91086	1.910864	2	0.347218	r2255	Resistance-Nodulation-Cell Division (RND) TCDB:2.A.60.1.5	1.00 * Bicarbonate [c] + 1.00 * mono(glucosyluronic acid	SLCO1B2	Transport, extracellular			0
r2256_neg	1	0.19043	0.7202646	-1.91086	1.910864	2	0.347218	r2256	Resistance-Nodulation-Cell Division (RND) TCDB:2.A.60.1.5	1.00 * Bicarbonate [c] + 1.00 * mono(glucosyluronic acid	SLCO1B2	Transport, extracellular			0
r2256_pos	1	0.19043	0.7202646	-1.91086	1.910864	2	0.347218	r2256	Resistance-Nodulation-Cell Division (RND) TCDB:2.A.60.1.5	1.00 * Bicarbonate [c] + 1.00 * mono(glucosyluronic acid	SLCO1B2	Transport, extracellular			0
r2257_neg	1	0.19043	0.7202646	-1.85065	1.850655	2	0.347218	r2257	Resistance-Nodulation-Cell Division (RND) TCDB:2.A.60.1.5	1.00 * Bicarbonate [c] + 1.00 * glutathionyl-leuc4 [c] + 1.0	SLCO1B2	Transport, extracellular			0
r2257_pos	1	0.19043	0.7202646	-1.85065	1.850655	2	0.347218	r2257	Resistance-Nodulation-Cell Division (RND) TCDB:2.A.60.1.5	1.00 * Bicarbonate [c] + 1.00 * glutathionyl-leuc4 [c] + 1.0	SLCO1B2	Transport, extracellular			0
r2258_neg	1	0.19043	0.7202646	-1.85065	1.850655	2	0.347218	r2258	Resistance-Nodulation-Cell Division (RND) TCDB:2.A.60.1.5	1.00 * Bicarbonate [c] + 1.00 * S-glutathionyl-2-4-dinitro	SLCO1B2	Transport, extracellular			0
r2258_pos	1	0.19043	0.7202646	-1.85065	1.850655	2	0.347218	r2258	Resistance-Nodulation-Cell Division (RND) TCDB:2.A.60.1.5	1.00 * Bicarbonate [c] + 1.00 * S-glutathionyl-2-4-dinitro	SLCO1B2	Transport, extracellular			0
r2259_neg	1	0.19043	0.7202646	-1.85065	1.850655	2	0.347218	r2259	Resistance-Nodulation-Cell Division (RND) TCDB:2.A.60.1.5	1.00 * Bicarbonate [c] + 1.00 * S-glutathionyl-ethacrynic-	SLCO1B2	Transport, extracellular			0
r2259_pos	1	0.19043	0.7202646	-1.85065	1.850655	2	0.347218	r2259	Resistance-Nodulation-Cell Division (RND) TCDB:2.A.60.1.5	1.00 * Bicarbonate [c] + 1.00 * S-glutathionyl-ethacrynic-	SLCO1B2	Transport, extracellular			0
r2260_neg	1	0.19043	0.7202646	-1.85065	1.850655	2	0.347218	r2260	Resistance-Nodulation-Cell Division (RND) TCDB:2.A.60.1.5	1.00 * Bicarbonate [c] + 1.00 * glutathionyl-leuc4 [c] + 1.0	SLCO1B2	Transport, extracellular			0
r2260_pos	1	0.19043	0.7202646	-1.85065	1.850655	2	0.347218	r2260	Resistance-Nodulation-Cell Division (RND) TCDB:2.A.60.1.5	1.00 * Bicarbonate [c] + 1.00 * glutathionyl-leuc4 [c] + 1.0	SLCO1B2	Transport, extracellular			0
r2261_neg	1	0.19043	0.7202646	-1.85065	1.850655	2	0.347218	r2261	Resistance-Nodulation-Cell Division (RND) TCDB:2.A.60.1.5	1.00 * Bicarbonate [c] + 1.00 * S-glutathionyl-2-4-dinitro	SLCO1B2	Transport, extracellular			0
r2261_pos	1	0.19043	0.7202646	-1.85065	1.850655	2	0.347218	r2261	Resistance-Nodulation-Cell Division (RND) TCDB:2.A.60.1.5	1.00 * Bicarbonate [c] + 1.00 * S-glutathionyl-2-4-dinitro	SLCO1B2	Transport, extracellular			0
r2262_neg	1	0.19043	0.7202646	-1.85065	1.850655	2	0.347218	r2262	Resistance-Nodulation-Cell Division (RND) TCDB:2.A.60.1.5	1.00 * Bicarbonate [c] + 1.00 * S-glutathionyl-ethacrynic-	SLCO1B2	Transport, extracellular			0
r2262_pos	1	0.19043	0.7202646	-1.85065	1.850655	2	0.347218	r2262	Resistance-Nodulation-Cell Division (RND) TCDB:2.A.60.1.5	1.00 * Bicarbonate [c] + 1.00 * S-glutathionyl-ethacrynic-	SLCO1B2	Transport, extracellular			0
r2263_neg	1	0.19043	0.7202646	-1.85065	1.850655	2	0.347218	r2263	Resistance-Nodulation-Cell Division (RND) TCDB:2.A.60.1.5	1.00 * Bicarbonate [c] + 1.00 * glutathionyl-leuc4 [c] + 1.0	SLCO1B2	Transport, extracellular			0
r2263_pos	1	0.19043	0.7202646	-1.85065	1.850655	2	0.347218	r2263	Resistance-Nodulation-Cell Division (RND) TCDB:2.A.60.1.5	1.00 * Bicarbonate [c] + 1.00 * glutathionyl-leuc4 [c] + 1.0	SLCO1B2	Transport, extracellular			0

r2264_neg	1	0.19043	0.7202646	-1.85065	1.850655	2	0.347218	r2264	Resistance-Nodulation-Cell Division (RND) TCDB:2.A.60.1.5	1.00 * Bicarbonate [c] + 1.00 * S-glutathionyl-2-4-dinitro	SLCO1B2	Transport, extracellular			0
r2264_pos	1	0.19043	0.7202646	-1.85065	1.850655	2	0.347218	r2264	Resistance-Nodulation-Cell Division (RND) TCDB:2.A.60.1.5	1.00 * Bicarbonate [c] + 1.00 * S-glutathionyl-2-4-dinitro	SLCO1B2	Transport, extracellular			0
r2265_neg	1	0.19043	0.7202646	-1.85065	1.850655	2	0.347218	r2265	Resistance-Nodulation-Cell Division (RND) TCDB:2.A.60.1.5	1.00 * Bicarbonate [c] + 1.00 * S-glutathionyl-ethacrynic-	SLCO1B2	Transport, extracellular			0
r2265_pos	1	0.19043	0.7202646	-1.85065	1.850655	2	0.347218	r2265	Resistance-Nodulation-Cell Division (RND) TCDB:2.A.60.1.5	1.00 * Bicarbonate [c] + 1.00 * S-glutathionyl-ethacrynic-	SLCO1B2	Transport, extracellular			0
r2266_neg	1	0.19043	0.7202646	-1.85065	1.850655	2	0.347218	r2266	Resistance-Nodulation-Cell Division (RND) TCDB:2.A.60.1.5	1.00 * Bicarbonate [c] + 1.00 * glutathionyl-leuc4 [c] + 1.0	SLCO1B2	Transport, extracellular			0
r2266_pos	1	0.19043	0.7202646	-1.85065	1.850655	2	0.347218	r2266	Resistance-Nodulation-Cell Division (RND) TCDB:2.A.60.1.5	1.00 * Bicarbonate [c] + 1.00 * glutathionyl-leuc4 [c] + 1.0	SLCO1B2	Transport, extracellular			0
r2267_neg	1	0.19043	0.7202646	-1.85065	1.850655	2	0.347218	r2267	Resistance-Nodulation-Cell Division (RND) TCDB:2.A.60.1.5	1.00 * Bicarbonate [c] + 1.00 * S-glutathionyl-2-4-dinitro	SLCO1B2	Transport, extracellular			0
r2267_pos	1	0.19043	0.7202646	-1.85065	1.850655	2	0.347218	r2267	Resistance-Nodulation-Cell Division (RND) TCDB:2.A.60.1.5	1.00 * Bicarbonate [c] + 1.00 * S-glutathionyl-2-4-dinitro	SLCO1B2	Transport, extracellular			0
r2268_neg	1	0.19043	0.7202646	-1.85065	1.850655	2	0.347218	r2268	Resistance-Nodulation-Cell Division (RND) TCDB:2.A.60.1.5	1.00 * Bicarbonate [c] + 1.00 * S-glutathionyl-ethacrynic-	SLCO1B2	Transport, extracellular			0
r2268_pos	1	0.19043	0.7202646	-1.85065	1.850655	2	0.347218	r2268	Resistance-Nodulation-Cell Division (RND) TCDB:2.A.60.1.5	1.00 * Bicarbonate [c] + 1.00 * S-glutathionyl-ethacrynic-	SLCO1B2	Transport, extracellular			0
r2269_neg	1	0.19043	0.7202646	-1.85065	1.850655	2	0.347218	r2269	Resistance-Nodulation-Cell Division (RND) TCDB:2.A.60.1.5	1.00 * Bicarbonate [c] + 1.00 * glutathionyl-leuc4 [c] + 1.0	SLCO1B2	Transport, extracellular			0
r2269_pos	1	0.19043	0.7202646	-1.85065	1.850655	2	0.347218	r2269	Resistance-Nodulation-Cell Division (RND) TCDB:2.A.60.1.5	1.00 * Bicarbonate [c] + 1.00 * glutathionyl-leuc4 [c] + 1.0	SLCO1B2	Transport, extracellular			0
r2270_neg	1	0.19043	0.7202646	-1.85065	1.850655	2	0.347218	r2270	Resistance-Nodulation-Cell Division (RND) TCDB:2.A.60.1.5	1.00 * Bicarbonate [c] + 1.00 * S-glutathionyl-2-4-dinitro	SLCO1B2	Transport, extracellular			0
r2270_pos	1	0.19043	0.7202646	-1.85065	1.850655	2	0.347218	r2270	Resistance-Nodulation-Cell Division (RND) TCDB:2.A.60.1.5	1.00 * Bicarbonate [c] + 1.00 * S-glutathionyl-2-4-dinitro	SLCO1B2	Transport, extracellular			0
r2271_neg	1	0.19043	0.7202646	-1.85065	1.850655	2	0.347218	r2271	Resistance-Nodulation-Cell Division (RND) TCDB:2.A.60.1.5	1.00 * Bicarbonate [c] + 1.00 * S-glutathionyl-ethacrynic-	SLCO1B2	Transport, extracellular			0
r2271_pos	1	0.19043	0.7202646	-1.85065	1.850655	2	0.347218	r2271	Resistance-Nodulation-Cell Division (RND) TCDB:2.A.60.1.5	1.00 * Bicarbonate [c] + 1.00 * S-glutathionyl-ethacrynic-	SLCO1B2	Transport, extracellular			0
r2272_neg	1	0.19043	0.7202646	-1.85065	1.850655	2	0.347218	r2272	Resistance-Nodulation-Cell Division (RND) TCDB:2.A.60.1.5	1.00 * Bicarbonate [c] + 1.00 * glutathionyl-leuc4 [c] + 1.0	SLCO1B2	Transport, extracellular			0
r2272_pos	1	0.19043	0.7202646	-1.85065	1.850655	2	0.347218	r2272	Resistance-Nodulation-Cell Division (RND) TCDB:2.A.60.1.5	1.00 * Bicarbonate [c] + 1.00 * glutathionyl-leuc4 [c] + 1.0	SLCO1B2	Transport, extracellular			0
r2273_neg	1	0.19043	0.7202646	-1.85065	1.850655	2	0.347218	r2273	Resistance-Nodulation-Cell Division (RND) TCDB:2.A.60.1.5	1.00 * Bicarbonate [c] + 1.00 * S-glutathionyl-2-4-dinitro	SLCO1B2	Transport, extracellular			0
r2273_pos	1	0.19043	0.7202646	-1.85065	1.850655	2	0.347218	r2273	Resistance-Nodulation-Cell Division (RND) TCDB:2.A.60.1.5	1.00 * Bicarbonate [c] + 1.00 * S-glutathionyl-2-4-dinitro	SLCO1B2	Transport, extracellular			0
r2274_neg	1	0.19043	0.7202646	-1.85065	1.850655	2	0.347218	r2274	Resistance-Nodulation-Cell Division (RND) TCDB:2.A.60.1.5	1.00 * Bicarbonate [c] + 1.00 * S-glutathionyl-ethacrynic-	SLCO1B2	Transport, extracellular			0
r2274_pos	1	0.19043	0.7202646	-1.85065	1.850655	2	0.347218	r2274	Resistance-Nodulation-Cell Division (RND) TCDB:2.A.60.1.5	1.00 * Bicarbonate [c] + 1.00 * S-glutathionyl-ethacrynic-	SLCO1B2	Transport, extracellular			0
r2275_neg	1	0.19043	0.7202646	-1.85065	1.850655	2	0.347218	r2275	Resistance-Nodulation-Cell Division (RND) TCDB:2.A.60.1.5	1.00 * Bicarbonate [c] + 1.00 * glutathionyl-leuc4 [c] + 1.0	SLCO1B2	Transport, extracellular			0
r2275_pos	1	0.19043	0.7202646	-1.85065	1.850655	2	0.347218	r2275	Resistance-Nodulation-Cell Division (RND) TCDB:2.A.60.1.5	1.00 * Bicarbonate [c] + 1.00 * glutathionyl-leuc4 [c] + 1.0	SLCO1B2	Transport, extracellular			0
r2276_neg	1	0.19043	0.7202646	-1.85065	1.850655	2	0.347218	r2276	Resistance-Nodulation-Cell Division (RND) TCDB:2.A.60.1.5	1.00 * Bicarbonate [c] + 1.00 * S-glutathionyl-2-4-dinitro	SLCO1B2	Transport, extracellular			0
r2276_pos	1	0.19043	0.7202646	-1.85065	1.850655	2	0.347218	r2276	Resistance-Nodulation-Cell Division (RND) TCDB:2.A.60.1.5	1.00 * Bicarbonate [c] + 1.00 * S-glutathionyl-2-4-dinitro	SLCO1B2	Transport, extracellular			0
r2277_neg	1	0.19043	0.7202646	-1.85065	1.850655	2	0.347218	r2277	Resistance-Nodulation-Cell Division (RND) TCDB:2.A.60.1.5	1.00 * Bicarbonate [c] + 1.00 * S-glutathionyl-ethacrynic-	SLCO1B2	Transport, extracellular			0
r2277_pos	1	0.19043	0.7202646	-1.85065	1.850655	2	0.347218	r2277	Resistance-Nodulation-Cell Division (RND) TCDB:2.A.60.1.5	1.00 * Bicarbonate [c] + 1.00 * S-glutathionyl-ethacrynic-	SLCO1B2	Transport, extracellular			0
r2278_neg	1	0.19043	0.7202646	-1.85065	1.850655	2	0.347218	r2278	Resistance-Nodulation-Cell Division (RND) TCDB:2.A.60.1.5	1.00 * Bicarbonate [c] + 1.00 * prostaglandin D2(1-) [e] + 1	SLCO1B2	Transport, extracellular			0
r2278_pos	1	0.19043	0.7202646	-1.85065	1.850655	2	0.347218	r2278	Resistance-Nodulation-Cell Division (RND) TCDB:2.A.60.1.5	1.00 * Bicarbonate [c] + 1.00 * prostaglandin D2(1-) [e] + 1	SLCO1B2	Transport, extracellular			0
r2279_neg	1	0.19043	0.7202646	-1.85065	1.850655	2	0.347218	r2279	Resistance-Nodulation-Cell Division (RND) TCDB:2.A.60.1.5	1.00 * Bicarbonate [c] + 1.00 * prostaglandin D2(1-) [e] + 1	SLCO1B2	Transport, extracellular			0
r2279_pos	1	0.19043	0.7202646	-1.85065	1.850655	2	0.347218	r2279	Resistance-Nodulation-Cell Division (RND) TCDB:2.A.60.1.5	1.00 * Bicarbonate [c] + 1.00 * prostaglandin D2(1-) [e] + 1	SLCO1B2	Transport, extracellular			0
r2280_neg	1	0.19043	0.7202646	-1.85065	1.850655	2	0.347218	r2280	Resistance-Nodulation-Cell Division (RND) TCDB:2.A.60.1.5	1.00 * Bicarbonate [c] + 1.00 * prostaglandin D2(1-) [e] + 1	SLCO1B2	Transport, extracellular			0
r2280_pos	1	0.19043	0.7202646	-1.85065	1.850655	2	0.347218	r2280	Resistance-Nodulation-Cell Division (RND) TCDB:2.A.60.1.5	1.00 * Bicarbonate [c] + 1.00 * prostaglandin D2(1-) [e] + 1	SLCO1B2	Transport, extracellular			0
r2281_neg	1	0.19043	0.7202646	-1.85065	1.850655	2	0.347218	r2281	Resistance-Nodulation-Cell Division (RND) TCDB:2.A.60.1.5	1.00 * Bicarbonate [c] + 1.00 * glutathionyl-leuc4 [c] + 1.0	SLCO1B2	Transport, extracellular			0
r2281_pos	1	0.19043	0.7202646	-1.85065	1.850655	2	0.347218	r2281	Resistance-Nodulation-Cell Division (RND) TCDB:2.A.60.1.5	1.00 * Bicarbonate [c] + 1.00 * glutathionyl-leuc4 [c] + 1.0	SLCO1B2	Transport, extracellular			0
r2282_neg	1	0.19043	0.7202646	-1.85065	1.850655	2	0.347218	r2282	Resistance-Nodulation-Cell Division (RND) TCDB:2.A.60.1.5	1.00 * Bicarbonate [c] + 1.00 * S-glutathionyl-2-4-dinitro	SLCO1B2	Transport, extracellular			0
r2282_pos	1	0.19043	0.7202646	-1.85065	1.850655	2	0.347218	r2282	Resistance-Nodulation-Cell Division (RND) TCDB:2.A.60.1.5	1.00 * Bicarbonate [c] + 1.00 * S-glutathionyl-2-4-dinitro	SLCO1B2	Transport, extracellular			0
r2283_neg	1	0.19043	0.7202646	-1.85065	1.850655	2	0.347218	r2283	Resistance-Nodulation-Cell Division (RND) TCDB:2.A.60.1.5	1.00 * Bicarbonate [c] + 1.00 * S-glutathionyl-ethacrynic-	SLCO1B2	Transport, extracellular			0
r2283_pos	1	0.19043	0.7202646	-1.85065	1.850655	2	0.347218	r2283	Resistance-Nodulation-Cell Division (RND) TCDB:2.A.60.1.5	1.00 * Bicarbonate [c] + 1.00 * S-glutathionyl-ethacrynic-	SLCO1B2	Transport, extracellular			0
r2284_neg	1	0.19043	0.7202646	-1.85065	1.850655	2	0.347218	r2284	Resistance-Nodulation-Cell Division (RND) TCDB:2.A.60.1.5	1.00 * Bicarbonate [c] + 1.00 * prostaglandin E1(1-) [e] + 1	SLCO1B2	Transport, extracellular			0
r2284_pos	1	0.19043	0.7202646	-1.85065	1.850655	2	0.347218	r2284	Resistance-Nodulation-Cell Division (RND) TCDB:2.A.60.1.5	1.00 * Bicarbonate [c] + 1.00 * prostaglandin E1(1-) [e] + 1	SLCO1B2	Transport, extracellular			0
r2285_neg	1	0.19043	0.7202646	-1.85065	1.850655	2	0.347218	r2285	Resistance-Nodulation-Cell Division (RND) TCDB:2.A.60.1.5	1.00 * Bicarbonate [c] + 1.00 * prostaglandin E1(1-) [e] + 1	SLCO1B2	Transport, extracellular			0
r2285_pos	1	0.19043	0.7202646	-1.85065	1.850655	2	0.347218	r2285	Resistance-Nodulation-Cell Division (RND) TCDB:2.A.60.1.5	1.00 * Bicarbonate [c] + 1.00 * prostaglandin E1(1-) [e] + 1	SLCO1B2	Transport, extracellular			0
r2286_neg	1	0.19043	0.7202646	-1.85065	1.850655	2	0.347218	r2286	Resistance-Nodulation-Cell Division (RND) TCDB:2.A.60.1.5	1.00 * Bicarbonate [c] + 1.00 * prostaglandin E1(1-) [e] + 1	SLCO1B2	Transport, extracellular			0
r2286_pos	1	0.19043	0.7202646	-1.85065	1.850655	2	0.347218	r2286	Resistance-Nodulation-Cell Division (RND) TCDB:2.A.60.1.5	1.00 * Bicarbonate [c] + 1.00 * prostaglandin E1(1-) [e] + 1	SLCO1B2	Transport, extracellular			0
r2287_neg	1	0.19043	0.7202646	-1.85065	1.850655	2	0.347218	r2287	Resistance-Nodulation-Cell Division (RND) TCDB:2.A.60.1.5	1.00 * Bicarbonate [c] + 1.00 * glutathionyl-leuc4 [c] + 1.0	SLCO1B2	Transport, extracellular			0
r2287_pos	1	0.19043	0.7202646	-1.85065	1.850655	2	0.347218	r2287	Resistance-Nodulation-Cell Division (RND) TCDB:2.A.60.1.5	1.00 * Bicarbonate [c] + 1.00 * glutathionyl-leuc4 [c] + 1.0	SLCO1B2	Transport, extracellular			0
r2288_neg	1	0.19043	0.7202646	-1.85065	1.850655	2	0.347218	r2288	Resistance-Nodulation-Cell Division (RND) TCDB:2.A.60.1.5	1.00 * Bicarbonate [c] + 1.00 * S-glutathionyl-2-4-dinitro	SLCO1B2	Transport, extracellular			0
r2288_pos	1	0.19043	0.7202646	-1.85065	1.850655	2	0.347218	r2288	Resistance-Nodulation-Cell Division (RND) TCDB:2.A.60.1.5	1.00 * Bicarbonate [c] + 1.00 * S-glutathionyl-2-4-dinitro	SLCO1B2	Transport, extracellular			0
r2289_neg	1	0.19043	0.7202646	-1.85065	1.850655	2	0.347218	r2289	Resistance-Nodulation-Cell Division (RND) TCDB:2.A.60.1.5	1.00 * Bicarbonate [c] + 1.00 * S-glutathionyl-ethacrynic-	SLCO1B2	Transport, extracellular			0
r2289_pos	1	0.19043	0.7202646	-1.85065	1.850655	2	0.347218	r2289	Resistance-Nodulation-Cell Division (RND) TCDB:2.A.60.1.5	1.00 * Bicarbonate [c] + 1.00 * S-glutathionyl-ethacrynic-	SLCO1B2	Transport, extracellular			0
r2290_neg	1	0.19043	0.7202646	-1.85065	1.850655	2	0.347218	r2290	Resistance-Nodulation-Cell Division (RND) TCDB:2.A.60.1.5	1.00 * Bicarbonate [c] + 1.00 * glutathionyl-leuc4 [c] + 1.0	SLCO1B2	Transport, extracellular			0
r2290_pos	1	0.19043	0.7202646	-1.85065	1.850655	2	0.347218	r2290	Resistance-Nodulation-Cell Division (RND) TCDB:2.A.60.1.5	1.00 * Bicarbonate [c] + 1.00 * glutathionyl-leuc4 [c] + 1.0	SLCO1B2	Transport, extracellular			0
r2291_neg	1	0.19043	0.7202646	-1.85065	1.850655	2	0.347218	r2291	Resistance-Nodulation-Cell Division (RND) TCDB:2.A.60.1.5	1.00 * Bicarbonate [c] + 1.00 * S-glutathionyl-2-4-dinitro	SLCO1B2	Transport, extracellular			0
r2291_pos	1	0.19043	0.7202646	-1.85065	1.850655	2	0.347218	r2291	Resistance-Nodulation-Cell Division (RND) TCDB:2.A.60.1.5	1.00 * Bicarbonate [c] + 1.00 * S-glutathionyl-2-4-dinitro	SLCO1B2	Transport, extracellular			0
r2292_neg	1	0.19043	0.7202646	-1.85065	1.850655	2	0.347218	r2292	Resistance-Nodulation-Cell Division (RND) TCDB:2.A.60.1.5	1.00 * Bicarbonate [c] + 1.00 * S-glutathionyl-ethacrynic-	SLCO1B2	Transport, extracellular			0
r2292_pos	1	0.19043	0.7202646	-1.85065	1.850655	2	0.347218	r2292	Resistance-Nodulation-Cell Division (RND) TCDB:2.A.60.1.5	1.00 * Bicarbonate [c] + 1.00 * S-glutathionyl-ethacrynic-	SLCO1B2	Transport, extracellular			0
r2296_neg	1	0.19043	0.7202646	-1.85065	1.850655	2	0.347218	r2296	Resistance-Nodulation-Cell Division (RND) TCDB:2.A.60.1.5	1.00 * Bicarbonate [c] + 1.00 * glutathionyl-leuc4 [c] + 1.0	SLCO1B2	Transport, extracellular			0
r2296_pos	1	0.19043	0.7202646	-1.85065	1.850655	2	0.347218	r2296	Resistance-Nodulation-Cell Division (RND) TCDB:2.A.60.1.5	1.00 * Bicarbonate [c] + 1.00 * glutathionyl-leuc4 [c] + 1.0	SLCO1B2	Transport, extracellular			0
r2297_neg	1	0.19043	0.7202646	-1.85065	1.850655	2	0.347218	r2297	Resistance-Nodulation-Cell Division (RND) TCDB:2.A.60.1.5	1.00 * Bicarbonate [c] + 1.00 * S-glutathionyl-2-4-dinitro	SLCO1B2	Transport, extracellular			0
r2297_pos	1	0.19043	0.7202646	-1.85065	1.850655	2	0.347218	r2297	Resistance-Nodulation-Cell Division (RND) TCDB:2.A.60.1.5	1.00 * Bicarbonate [c] + 1.00 * S-glutathionyl-2-4-dinitro	SLCO1B2	Transport, extracellular			0
r2298_neg	1	0.19043	0.7202646	-1.85065	1.850655	2	0.347218	r2298	Resistance-Nodulation-Cell Division (RND) TCDB:2.A.60.1.5	1.00 * Bicarbonate [c] + 1.00 * S-glutathionyl-ethacrynic-	SLCO1B2	Transport, extracellular			0
r2298_pos	1	0.19043	0.7202646	-1.85065	1.850655	2	0.347218	r2298	Resistance-Nodulation-Cell Division (RND) TCDB:2.A.60.1.5	1.00 * Bicarbonate [c] + 1.00 * S-glutathionyl-ethacrynic-	SLCO1B2	Transport, extracellular			0
r2299_neg	1	0.19043	0.7202646	-1.85065	1.850655	2	0.347218	r2299	Resistance-Nodulation-Cell Division (RND) TCDB:2.A.60.1.5	1.00 * Bicarbonate [c] + 1.00 * glutathionyl-leuc4 [c] + 1.0	SLCO1B2	Transport, extracellular			0
r2299_pos	1	0.19043	0.7202646	-1.85065	1.850655	2	0.347218	r2299	Resistance-Nodulation-Cell Division (RND) TCDB:2.A.60.1.5	1.00 * Bicarbonate [c] + 1.00 * glutathionyl-leuc4 [c] + 1.0	SLCO1B2	Transport, extracellular			0
r2300_neg	1	0.19043	0.7202646	-1.85065	1.850655	2	0.347218	r2300	Resistance-Nodulation-Cell Division (RND) TCDB:2.A.60.1.5	1.00 * Bicarbonate [c] + 1.00 * S-glutathionyl-2-4-dinitro	SLCO1B2	Transport, extracellular			0
r2300_pos	1	0.19043	0.7202646	-1.85065	1.850655	2	0.347218	r2300	Resistance-Nodulation-Cell Division (RND) TCDB:2.A.60.1.5	1.00 * Bicarbonate [c] + 1.00 * S-glutathionyl-2-4-dinitro	SLCO1B2	Transport, extracellular			0
r2301_neg	1	0.19043	0.7202646	-1.85065	1.850655	2	0.347218	r2301	Resistance-Nodulation-Cell Division (RND) TCDB:2.A.60.1.5	1.00 * Bicarbonate [c] + 1.00 * S-glutathionyl-ethacrynic-	SLCO1B2	Transport, extracellular			0
r2301_pos	1	0.19043	0.7202646	-1.85065	1.850655	2	0.347218	r2301	Resistance-Nodulation-Cell Division (RND) TCDB:2.A.60.1.5	1.00 * Bicarbonate [c] + 1.00 * S-glutathionyl-ethacrynic-	SLCO1B2	Transport, extracellular			0
r2302_neg	1	0.19043	0.7202646	-1.85065	1.850655	2	0.347218	r2302	Resistance-Nodulation-Cell Division (RND) TCDB:2.A.60.1.5	1.00 * Bicarbonate [c] + 1.00 * prostaglandin H2(1-) [e] + 1	SLCO1B2	Transport, extracellular			0
r2302_pos	1	0.19043	0.7202646	-1.85065	1.850655	2	0.347218	r2302	Resistance-Nodulation-Cell Division (RND) TCDB:2.A.60.1.5	1.00 * Bicarbonate [c] + 1.00 * prostaglandin H2(1-) [e] + 1	SLCO1B2	Transport, extracellular			0
r2303_neg	1	0.19043	0.7202646	-1.85065	1.850655	2	0.347218	r2303	Resistance-Nodulation-Cell Division (RND) TCDB:2.A.60.1.5	1.00 * Bicarbonate [c] + 1.00 * prostaglandin H2(1-) [e] + 1	SLCO1B2	Transport, extracellular			0
r2303_pos	1	0.19043	0.7202646	-1.85065	1.850655	2	0.347218	r2303	Resistance-Nodulation-Cell Division (RND) TCDB:2.A.60.1.5	1.00 * Bicarbonate [c] + 1.00 * prostaglandin H2(1-) [e] + 1	SLCO1B2	Transport, extracellular			0
r2304_neg	1	0.19043	0.7202646	-1.85065	1.850655	2	0.347218	r2304	Resistance-Nodulation-Cell Division (RND) TCDB:2.A.60.1.5	1.00 * Bicarbonate [c] + 1.00 * prostaglandin H2(1-) [e] + 1	SLCO1B2	Transport, extracellular			0
r2304_pos	1	0.19043	0.7202646	-1.85065	1.850655	2	0.347218	r2304	Resistance-Nodulation-Cell Division (RND) TCDB:2.A.60.1.5	1.00 * Bicarbonate [c] + 1.00 * prostaglandin H2(1-) [e] + 1	SLCO1B2	Transport, extracellular			0
r2305_neg	1	0.19043	0.7202646	-1.85065	1.850655	2	0.347218	r2305	Resistance-Nodulation-Cell Division (RND) TCDB:2.A.60.1.5	1.00 * Bicarbonate [c] + 1.00 * prostaglandin I2(1-) [e] + 1	SLCO1B2	Transport, extracellular			0
r2305_pos	1	0.19043	0.7202646	-1.85065	1.850655	2	0.347218	r2305	Resistance-Nodulation-Cell Division (RND) TCDB:2.A.60.1.5	1.00 * Bicarbonate [c] + 1.00 * prostaglandin I2(1-) [e] + 1	SLCO1B2	Transport, extracellular			0
r2306_neg	1	0.19043	0.7202646	-1.85065	1.850655	2	0.347218	r2306	Resistance-Nodulation-Cell Division (RND) TCDB:2.A.60.1.5	1.00 * Bicarbonate [c] + 1.00 * prostaglandin I2(1-) [e] + 1	SLCO1B2	Transport, extracellular			0
r2306_pos	1	0.19043	0.7202646	-1.85065	1.850655	2	0.347218	r2306	Resistance-Nodulation-Cell Division (RND) TCDB:2.A.60.1.5	1.00 * Bicarbonate [c] + 1.00 * prostaglandin I2(1-) [e] + 1	SLCO1B2	Transport, extracellular			0
r2307_neg	1	0.19043	0.7202646	-1.85065	1.850655	2	0.347218	r2307	Resistance-Nodulation-Cell Division (RND) TCDB:2.A.60.1.5	1.00 * Bicarbonate [c] + 1.00 * prostaglandin I2(1-) [e] + 1	SLCO1B2	Transport, extracellular			0
r2307_pos	1	0.19043	0.7202646	-1.85065	1.850655	2	0.347218	r2307	Resistance-Nodulation-Cell Division (RND) TCDB:2.A.60.1.5	1.00 * Bicarbonate [c] + 1.00 * prostaglandin I2(1-) [e] + 1	SLCO1B2	Transport, extracellular			0
r2313_neg	1	0.19043	0.7202646	-0.98541	0.985411	2	0.347218	r2313	Resistance-Nodulation-Cell Division (RND) TCDB:2.A.60.1.2	1.00 * Bicarbonate [c] + 1.00 * arachidonate [e] + 1.00 * R	SLCO2A1	Transport, extracellular			0

r2313_pos	1	0.19043	0.7202646	-0.98541	0.985411	2	0.347218	r2313	Resistance-Nodulation-Cell Division (RND) TCDB:2.A.60.1.2	1.00 * Bicarbonate [c] + 1.00 * arachidonate [e] + 1.00 * R	SLCO2A1	Transport, extracellular			0
r2314_neg	1	0.19043	0.7202646	-0.98541	0.985411	2	0.347218	r2314	Resistance-Nodulation-Cell Division (RND) TCDB:2.A.60.1.2	1.00 * Bicarbonate [c] + 1.00 * Reduced glutathione [c] +	SLCO2A1	Transport, extracellular			0
r2314_pos	1	0.19043	0.7202646	-0.98541	0.985411	2	0.347218	r2314	Resistance-Nodulation-Cell Division (RND) TCDB:2.A.60.1.2	1.00 * Bicarbonate [c] + 1.00 * Reduced glutathione [c] +	SLCO2A1	Transport, extracellular			0
r2315_neg	1	0.19043	0.7202646	-0.98541	0.985411	2	0.347218	r2315	Resistance-Nodulation-Cell Division (RND) TCDB:2.A.60.1.2	1.00 * Bicarbonate [c] + 1.00 * Reduced glutathione [c] +	SLCO2A1	Transport, extracellular			0
r2315_pos	1	0.19043	0.7202646	-0.98541	0.985411	2	0.347218	r2315	Resistance-Nodulation-Cell Division (RND) TCDB:2.A.60.1.2	1.00 * Bicarbonate [c] + 1.00 * Reduced glutathione [c] +	SLCO2A1	Transport, extracellular			0
r2316_neg	1	0.19043	0.7202646	-0.98541	0.985411	2	0.347218	r2316	Resistance-Nodulation-Cell Division (RND) TCDB:2.A.60.1.2	1.00 * Bicarbonate [c] + 1.00 * Reduced glutathione [c] +	SLCO2A1	Transport, extracellular			0
r2316_pos	1	0.19043	0.7202646	-0.98541	0.985411	2	0.347218	r2316	Resistance-Nodulation-Cell Division (RND) TCDB:2.A.60.1.2	1.00 * Bicarbonate [c] + 1.00 * Reduced glutathione [c] +	SLCO2A1	Transport, extracellular			0
r2317_neg	1	0.19043	0.7202646	-0.98541	0.985411	2	0.347218	r2317	Resistance-Nodulation-Cell Division (RND) TCDB:2.A.60.1.2	1.00 * Bicarbonate [c] + 1.00 * Reduced glutathione [c] +	SLCO2A1	Transport, extracellular			0
r2317_pos	1	0.19043	0.7202646	-0.98541	0.985411	2	0.347218	r2317	Resistance-Nodulation-Cell Division (RND) TCDB:2.A.60.1.2	1.00 * Bicarbonate [c] + 1.00 * Reduced glutathione [c] +	SLCO2A1	Transport, extracellular			0
r2318_neg	1	0.19043	0.7202646	-0.98541	0.985411	2	0.347218	r2318	Resistance-Nodulation-Cell Division (RND) TCDB:2.A.60.1.2	1.00 * Bicarbonate [c] + 1.00 * Reduced glutathione [c] +	SLCO2A1	Transport, extracellular			0
r2318_pos	1	0.19043	0.7202646	-0.98541	0.985411	2	0.347218	r2318	Resistance-Nodulation-Cell Division (RND) TCDB:2.A.60.1.2	1.00 * Bicarbonate [c] + 1.00 * Reduced glutathione [c] +	SLCO2A1	Transport, extracellular			0
r2319_neg	1	0.19043	0.7202646	-0.98541	0.985411	2	0.347218	r2319	Resistance-Nodulation-Cell Division (RND) TCDB:2.A.60.1.2	1.00 * Bicarbonate [c] + 1.00 * Reduced glutathione [c] +	SLCO2A1	Transport, extracellular			0
r2319_pos	1	0.19043	0.7202646	-0.98541	0.985411	2	0.347218	r2319	Resistance-Nodulation-Cell Division (RND) TCDB:2.A.60.1.2	1.00 * Bicarbonate [c] + 1.00 * Reduced glutathione [c] +	SLCO2A1	Transport, extracellular			0
r2320_neg	1	0.19043	0.7202646	-0.98541	0.985411	2	0.347218	r2320	Resistance-Nodulation-Cell Division (RND) TCDB:2.A.60.1.2	1.00 * Bicarbonate [c] + 1.00 * Reduced glutathione [c] +	SLCO2A1	Transport, extracellular			0
r2320_pos	1	0.19043	0.7202646	-0.98541	0.985411	2	0.347218	r2320	Resistance-Nodulation-Cell Division (RND) TCDB:2.A.60.1.2	1.00 * Bicarbonate [c] + 1.00 * Reduced glutathione [c] +	SLCO2A1	Transport, extracellular			0
r2321_neg	1	0.19043	0.7202646	-0.98541	0.985411	2	0.347218	r2321	Resistance-Nodulation-Cell Division (RND) TCDB:2.A.60.1.2	1.00 * Bicarbonate [c] + 1.00 * Reduced glutathione [c] +	SLCO2A1	Transport, extracellular			0
r2321_pos	1	0.19043	0.7202646	-0.98541	0.985411	2	0.347218	r2321	Resistance-Nodulation-Cell Division (RND) TCDB:2.A.60.1.2	1.00 * Bicarbonate [c] + 1.00 * Reduced glutathione [c] +	SLCO2A1	Transport, extracellular			0
r2322_neg	1	0.19043	0.7202646	-0.98541	0.985411	2	0.347218	r2322	Resistance-Nodulation-Cell Division (RND) TCDB:2.A.60.1.2	1.00 * Bicarbonate [c] + 1.00 * Reduced glutathione [c] +	SLCO2A1	Transport, extracellular			0
r2322_pos	1	0.19043	0.7202646	-0.98541	0.985411	2	0.347218	r2322	Resistance-Nodulation-Cell Division (RND) TCDB:2.A.60.1.2	1.00 * Bicarbonate [c] + 1.00 * Reduced glutathione [c] +	SLCO2A1	Transport, extracellular			0
r2323_neg	1	0.19043	0.7202646	-0.98541	0.985411	2	0.347218	r2323	Resistance-Nodulation-Cell Division (RND) TCDB:2.A.60.1.2	1.00 * Bicarbonate [c] + 1.00 * Reduced glutathione [c] +	SLCO2A1	Transport, extracellular			0
r2323_pos	1	0.19043	0.7202646	-0.98541	0.985411	2	0.347218	r2323	Resistance-Nodulation-Cell Division (RND) TCDB:2.A.60.1.2	1.00 * Bicarbonate [c] + 1.00 * Reduced glutathione [c] +	SLCO2A1	Transport, extracellular			0
r2324_neg	1	0.19043	0.7202646	-0.98541	0.985411	2	0.347218	r2324	Resistance-Nodulation-Cell Division (RND) TCDB:2.A.60.1.2	1.00 * Bicarbonate [c] + 1.00 * Reduced glutathione [c] +	SLCO2A1	Transport, extracellular			0
r2324_pos	1	0.19043	0.7202646	-0.98541	0.985411	2	0.347218	r2324	Resistance-Nodulation-Cell Division (RND) TCDB:2.A.60.1.2	1.00 * Bicarbonate [c] + 1.00 * Reduced glutathione [c] +	SLCO2A1	Transport, extracellular			0
r2325_neg	1	0.19043	0.7202646	-0.98541	0.985411	2	0.347218	r2325	Resistance-Nodulation-Cell Division (RND) TCDB:2.A.60.1.2	1.00 * Bicarbonate [c] + 1.00 * Reduced glutathione [c] +	SLCO2A1	Transport, extracellular			0
r2325_pos	1	0.19043	0.7202646	-0.98541	0.985411	2	0.347218	r2325	Resistance-Nodulation-Cell Division (RND) TCDB:2.A.60.1.2	1.00 * Bicarbonate [c] + 1.00 * Reduced glutathione [c] +	SLCO2A1	Transport, extracellular			0
r2326_neg	1	0.19043	0.7202646	-0.98541	0.985411	2	0.347218	r2326	Resistance-Nodulation-Cell Division (RND) TCDB:2.A.60.1.2	1.00 * Bicarbonate [c] + 1.00 * Reduced glutathione [c] +	SLCO2A1	Transport, extracellular			0
r2326_pos	1	0.19043	0.7202646	-0.98541	0.985411	2	0.347218	r2326	Resistance-Nodulation-Cell Division (RND) TCDB:2.A.60.1.2	1.00 * Bicarbonate [c] + 1.00 * Reduced glutathione [c] +	SLCO2A1	Transport, extracellular			0
r2327_neg	1	0.19043	0.7202646	-0.98541	0.985411	2	0.347218	r2327	Resistance-Nodulation-Cell Division (RND) TCDB:2.A.60.1.2	1.00 * Bicarbonate [c] + 1.00 * Reduced glutathione [c] +	SLCO2A1	Transport, extracellular			0
r2327_pos	1	0.19043	0.7202646	-0.98541	0.985411	2	0.347218	r2327	Resistance-Nodulation-Cell Division (RND) TCDB:2.A.60.1.2	1.00 * Bicarbonate [c] + 1.00 * Reduced glutathione [c] +	SLCO2A1	Transport, extracellular			0
r2328_neg	1	0.19043	0.7202646	-0.98541	0.985411	2	0.347218	r2328	Resistance-Nodulation-Cell Division (RND) TCDB:2.A.60.1.2	1.00 * Bicarbonate [c] + 1.00 * Reduced glutathione [c] +	SLCO2A1	Transport, extracellular			0
r2328_pos	1	0.19043	0.7202646	-0.98541	0.985411	2	0.347218	r2328	Resistance-Nodulation-Cell Division (RND) TCDB:2.A.60.1.2	1.00 * Bicarbonate [c] + 1.00 * Reduced glutathione [c] +	SLCO2A1	Transport, extracellular			0
r2329_neg	1	0.19043	0.7202646	-0.98541	0.985411	2	0.347218	r2329	Resistance-Nodulation-Cell Division (RND) TCDB:2.A.60.1.2	1.00 * Bicarbonate [c] + 1.00 * Reduced glutathione [c] +	SLCO2A1	Transport, extracellular			0
r2329_pos	1	0.19043	0.7202646	-0.98541	0.985411	2	0.347218	r2329	Resistance-Nodulation-Cell Division (RND) TCDB:2.A.60.1.2	1.00 * Bicarbonate [c] + 1.00 * Reduced glutathione [c] +	SLCO2A1	Transport, extracellular			0
r2330_neg	1	0.19043	0.7202646	-0.98541	0.985411	2	0.347218	r2330	Resistance-Nodulation-Cell Division (RND) TCDB:2.A.60.1.2	1.00 * Bicarbonate [c] + 1.00 * Reduced glutathione [c] +	SLCO2A1	Transport, extracellular			0
r2330_pos	1	0.19043	0.7202646	-0.98541	0.985411	2	0.347218	r2330	Resistance-Nodulation-Cell Division (RND) TCDB:2.A.60.1.2	1.00 * Bicarbonate [c] + 1.00 * Reduced glutathione [c] +	SLCO2A1	Transport, extracellular			0
r2331_neg	1	0.19043	0.7202646	-0.98541	0.985411	2	0.347218	r2331	Resistance-Nodulation-Cell Division (RND) TCDB:2.A.60.1.2	1.00 * Bicarbonate [c] + 1.00 * Reduced glutathione [c] +	SLCO2A1	Transport, extracellular			0
r2331_pos	1	0.19043	0.7202646	-0.98541	0.985411	2	0.347218	r2331	Resistance-Nodulation-Cell Division (RND) TCDB:2.A.60.1.2	1.00 * Bicarbonate [c] + 1.00 * Reduced glutathione [c] +	SLCO2A1	Transport, extracellular			0
r2332_neg	1	0.19043	0.7202646	-0.98541	0.985411	2	0.347218	r2332	Resistance-Nodulation-Cell Division (RND) TCDB:2.A.60.1.2	1.00 * Bicarbonate [c] + 1.00 * Reduced glutathione [c] +	SLCO2A1	Transport, extracellular			0
r2332_pos	1	0.19043	0.7202646	-0.98541	0.985411	2	0.347218	r2332	Resistance-Nodulation-Cell Division (RND) TCDB:2.A.60.1.2	1.00 * Bicarbonate [c] + 1.00 * Reduced glutathione [c] +	SLCO2A1	Transport, extracellular			0
r2333_neg	1	0.19043	0.7202646	-0.98541	0.985411	2	0.347218	r2333	Resistance-Nodulation-Cell Division (RND) TCDB:2.A.60.1.2	1.00 * Bicarbonate [c] + 1.00 * Reduced glutathione [c] +	SLCO2A1	Transport, extracellular			0
r2333_pos	1	0.19043	0.7202646	-0.98541	0.985411	2	0.347218	r2333	Resistance-Nodulation-Cell Division (RND) TCDB:2.A.60.1.2	1.00 * Bicarbonate [c] + 1.00 * Reduced glutathione [c] +	SLCO2A1	Transport, extracellular			0
r2334_neg	1	0.19043	0.7202646	-0.98541	0.985411	2	0.347218	r2334	Resistance-Nodulation-Cell Division (RND) TCDB:2.A.60.1.2	1.00 * Bicarbonate [c] + 1.00 * Reduced glutathione [c] +	SLCO2A1	Transport, extracellular			0
r2334_pos	1	0.19043	0.7202646	-0.98541	0.985411	2	0.347218	r2334	Resistance-Nodulation-Cell Division (RND) TCDB:2.A.60.1.2	1.00 * Bicarbonate [c] + 1.00 * Reduced glutathione [c] +	SLCO2A1	Transport, extracellular			0
r2335_neg	1	0.19043	0.7202646	-0.98541	0.985411	2	0.347218	r2335	Resistance-Nodulation-Cell Division (RND) TCDB:2.A.60.1.2	1.00 * Bicarbonate [c] + 1.00 * Reduced glutathione [c] +	SLCO2A1	Transport, extracellular			0
r2335_pos	1	0.19043	0.7202646	-0.98541	0.985411	2	0.347218	r2335	Resistance-Nodulation-Cell Division (RND) TCDB:2.A.60.1.2	1.00 * Bicarbonate [c] + 1.00 * Reduced glutathione [c] +	SLCO2A1	Transport, extracellular			0
r2341_neg	1	0.19043	0.7202646	-1.6198	1.619795	2	0.347218	r2341	Organic anion transporter 5 Utilized transport	1.00 * leukotriene C4(2-) [e] --> 1.00 * leukotriene C4(2-) [	1.00 * leuk	Transport, extracellular			0
r2341_pos	1	0.19043	0.7202646	-1.64712	1.647117	2	0.347218	r2341	Organic anion transporter 5 Utilized transport	1.00 * leukotriene C4(2-) [e] --> 1.00 * leukotriene C4(2-) [	1.00 * leuk	Transport, extracellular			0
r2342_neg	1	0.19043	0.7202646	-1.6324	1.632396	2	0.347218	r2342	Organic anion transporter 5 Utilized transport	1.00 * estrone 3-sulfate(1-) [e] --> 1.00 * estrone 3-sulfate(	1.00 * estro	Transport, extracellular			0
r2342_pos	1	0.19043	0.7202646	-1.59651	1.596512	2	0.347218	r2342	Organic anion transporter 5 Utilized transport	1.00 * estrone 3-sulfate(1-) [e] --> 1.00 * estrone 3-sulfate(	1.00 * estro	Transport, extracellular			0
r2343_neg	1	0.19043	0.7202646	-1.63257	1.632568	2	0.347218	r2343	Organic anion transporter 5 Utilized transport	1.00 * 3beta-hydroxyandrost-5-en-17-one 3-sulfate(1-) [e	1.00 * 3bet	Transport, extracellular			0
r2343_pos	1	0.19043	0.7202646	-1.65665	1.656655	2	0.347218	r2343	Organic anion transporter 5 Utilized transport	1.00 * 3beta-hydroxyandrost-5-en-17-one 3-sulfate(1-) [e	1.00 * 3bet	Transport, extracellular			0
r2344_neg	1	0.19043	0.7202646	-1.61251	1.612512	2	0.347218	r2344	Organic anion transporter 5 Utilized transport	1.00 * bis(beta-glucosyluronate)bilirubin [e] --> 1.00 * bis	1.00 * bis(b	Transport, extracellular			0
r2344_pos	1	0.19043	0.7202646	-1.63886	1.638857	2	0.347218	r2344	Organic anion transporter 5 Utilized transport	1.00 * bis(beta-glucosyluronate)bilirubin [e] --> 1.00 * bis	1.00 * bis(b	Transport, extracellular			0
r2346_neg	1	0.19043	0.7202646	-1.97541	1.975412	2	0.347218	r2346	Organic anion transporter 5 Utilized transport	1.00 * 20-hydroxy-arachidonate [e] --> 1.00 * 20-hydroxy	1.00 * 20-h	Transport, extracellular			0
r2346_pos	1	0.19043	0.7202646	-1.97541	1.975412	2	0.347218	r2346	Organic anion transporter 5 Utilized transport	1.00 * 20-hydroxy-arachidonate [e] --> 1.00 * 20-hydroxy	1.00 * 20-h	Transport, extracellular			0
r2347_neg	1	0.19043	0.7202646	-1.97541	1.975412	2	0.347218	r2347	Organic anion transporter 5 Utilized transport	1.00 * thromboxane-b2 [e] --> 1.00 * thromboxane-b2 [c]	1.00 * thro	Transport, extracellular			0
r2347_pos	1	0.19043	0.7202646	-1.97541	1.975412	2	0.347218	r2347	Organic anion transporter 5 Utilized transport	1.00 * thromboxane-b2 [e] --> 1.00 * thromboxane-b2 [c]	1.00 * thro	Transport, extracellular			0
r2353_neg	1	0.19043	0.7202646	-1.6198	1.619795	2	0.347218	r2353	Organic anion transporter 5 Utilized transport	1.00 * mono(glucosyluronic acid)bilirubin(2-) [e] --> 1.00	1.00 * mon	Transport, extracellular			0
r2353_pos	1	0.19043	0.7202646	-1.64712	1.647117	2	0.347218	r2353	Organic anion transporter 5 Utilized transport	1.00 * mono(glucosyluronic acid)bilirubin(2-) [e] --> 1.00	1.00 * mon	Transport, extracellular			0
r2354_neg	1	0.19043	0.7202646	-1.97541	1.975412	2	0.347218	r2354	Organic anion transporter 5 Utilized transport	1.00 * prostaglandin A1(1-) [e] --> 1.00 * prostaglandin A1	1.00 * pros	Transport, extracellular			0
r2354_pos	1	0.19043	0.7202646	-1.97541	1.975412	2	0.347218	r2354	Organic anion transporter 5 Utilized transport	1.00 * prostaglandin A1(1-) [e] --> 1.00 * prostaglandin A1	1.00 * pros	Transport, extracellular			0
r2355_neg	1	0.19043	0.7202646	-1.97541	1.975412	2	0.347218	r2355	Organic anion transporter 5 Utilized transport	1.00 * prostaglandin-a2 [e] --> 1.00 * prostaglandin-a2 [c]	1.00 * pros	Transport, extracellular			0
r2355_pos	1	0.19043	0.7202646	-1.97541	1.975412	2	0.347218	r2355	Organic anion transporter 5 Utilized transport	1.00 * prostaglandin-a2 [e] --> 1.00 * prostaglandin-a2 [c]	1.00 * pros	Transport, extracellular			0
r2356_neg	1	0.19043	0.7202646	-1.97541	1.975412	2	0.347218	r2356	Organic anion transporter 5 Utilized transport	1.00 * prostaglandin-b1 [e] --> 1.00 * prostaglandin-b1 [c	1.00 * pros	Transport, extracellular			0
r2356_pos	1	0.19043	0.7202646	-1.97541	1.975412	2	0.347218	r2356	Organic anion transporter 5 Utilized transport	1.00 * prostaglandin-b1 [e] --> 1.00 * prostaglandin-b1 [c	1.00 * pros	Transport, extracellular			0
r2357_neg	1	0.19043	0.7202646	-1.97541	1.975412	2	0.347218	r2357	Organic anion transporter 5 Utilized transport	1.00 * prostaglandin-b2 [e] --> 1.00 * prostaglandin-b2 [c	1.00 * pros	Transport, extracellular			0
r2357_pos	1	0.19043	0.7202646	-1.97541	1.975412	2	0.347218	r2357	Organic anion transporter 5 Utilized transport	1.00 * prostaglandin-b2 [e] --> 1.00 * prostaglandin-b2 [c	1.00 * pros	Transport, extracellular			0
r2358_neg	1	0.19043	0.7202646	-1.97541	1.975412	2	0.347218	r2358	Organic anion transporter 5 Utilized transport	1.00 * prostaglandin C1(1-) [e] --> 1.00 * prostaglandin C1	1.00 * pros	Transport, extracellular			0
r2358_pos	1	0.19043	0.7202646	-1.97541	1.975412	2	0.347218	r2358	Organic anion transporter 5 Utilized transport	1.00 * prostaglandin C1(1-) [e] --> 1.00 * prostaglandin C1	1.00 * pros	Transport, extracellular			0
r2359_neg	1	0.19043	0.7202646	-1.97541	1.975412	2	0.347218	r2359	Organic anion transporter 5 Utilized transport	1.00 * prostaglandin-c2 [e] --> 1.00 * prostaglandin-c2 [c]	1.00 * pros	Transport, extracellular			0
r2359_pos	1	0.19043	0.7202646	-1.97541	1.975412	2	0.347218	r2359	Organic anion transporter 5 Utilized transport	1.00 * prostaglandin-c2 [e] --> 1.00 * prostaglandin-c2 [c]	1.00 * pros	Transport, extracellular			0
r2360_neg	1	0.19043	0.7202646	-1.97541	1.975412	2	0.347218	r2360	Organic anion transporter 5 Utilized transport	1.00 * prostaglandin-d1 [e] --> 1.00 * prostaglandin-d1 [c	1.00 * pros	Transport, extracellular			0
r2360_pos	1	0.19043	0.7202646	-1.97541	1.975412	2	0.347218	r2360	Organic anion transporter 5 Utilized transport	1.00 * prostaglandin-d1 [e] --> 1.00 * prostaglandin-d1 [c	1.00 * pros	Transport, extracellular			0
r2361_neg	1	0.19043	0.7202646	-1.97541	1.975412	2	0.347218	r2361	Organic anion transporter 5 Utilized transport	1.00 * prostaglandin D2(1-) [e] --> 1.00 * prostaglandin D	1.00 * pros	Transport, extracellular			0
r2361_pos	1	0.19043	0.7202646	-1.97541	1.975412	2	0.347218	r2361	Organic anion transporter 5 Utilized transport	1.00 * prostaglandin D2(1-) [e] --> 1.00 * prostaglandin D	1.00 * pros	Transport, extracellular			0
r2362_neg	1	0.19043	0.7202646	-1.97541	1.975412	2	0.347218	r2362	Organic anion transporter 5 Utilized transport	1.00 * prostaglandin-d3 [e] --> 1.00 * prostaglandin-d3 [c	1.00 * pros	Transport, extracellular			0
r2362_pos	1	0.19043	0.7202646	-1.97541	1.975412	2	0.347218	r2362	Organic anion transporter 5 Utilized transport	1.00 * prostaglandin-d3 [e] --> 1.00 * prostaglandin-d3 [c	1.00 * pros	Transport, extracellular			0
r2363_neg	1	0.19043	0.7202646	-1.97541	1.975412	2	0.347218	r2363	Organic anion transporter 5 Utilized transport	1.00 * prostaglandin E1(1-) [e] --> 1.00 * prostaglandin E1	1.00 * pros	Transport, extracellular			0
r2363_pos	1	0.19043	0.7202646	-1.97541	1.975412	2	0.347218	r2363	Organic anion transporter 5 Utilized transport	1.00 * prostaglandin E1(1-) [e] --> 1.00 * prostaglandin E1	1.00 * pros	Transport, extracellular			0
r2364_neg	1	0.19043	0.7202646	-1.97541	1.975412	2	0.347218	r2364	Organic anion transporter 5 Utilized transport	1.00 * prostaglandin-e3 [e] --> 1.00 * prostaglandin-e3 [c]	1.00 * pros	Transport, extracellular			0
r2364_pos	1	0.19043	0.7202646	-1.97541	1.975412	2	0.347218	r2364	Organic anion transporter 5 Utilized transport	1.00 * prostaglandin-e3 [e] --> 1.00 * prostaglandin-e3 [c]	1.00 * pros	Transport, extracellular			0
r2365_neg	1	0.19043	0.7202646	-1.97541	1.975412	2	0.347218	r2365	Organic anion transporter 5 Utilized transport	1.00 * prostaglandin-f1alpha [e] --> 1.00 * prostaglandin-	1.00 * pros	Transport, extracellular			0
r2365_pos	1	0.19043	0.7202646	-1.97541	1.975412	2	0.347218	r2365	Organic anion transporter 5 Utilized transport	1.00 * prostaglandin-f1alpha [e] --> 1.00 * prostaglandin-	1.00 * pros	Transport, extracellular			0

r2366_neg	1	0.19043	0.7202646	-1.97541	1.975412	2	0.347218	r2366	Organic anion transporter 5 Utilized transport	1.00 * prostaglandin-f2beta [e] --> 1.00 * prostaglandin-f2	1.00 * pros	Transport, extracellular			0
r2366_pos	1	0.19043	0.7202646	-1.97541	1.975412	2	0.347218	r2366	Organic anion transporter 5 Utilized transport	1.00 * prostaglandin-f2beta [e] --> 1.00 * prostaglandin-f2	1.00 * pros	Transport, extracellular			0
r2367_neg	1	0.19043	0.7202646	-1.97541	1.975412	2	0.347218	r2367	Organic anion transporter 5 Utilized transport	1.00 * prostaglandin-g2 [e] --> 1.00 * prostaglandin-g2 [c]	1.00 * pros	Transport, extracellular			0
r2367_pos	1	0.19043	0.7202646	-1.97541	1.975412	2	0.347218	r2367	Organic anion transporter 5 Utilized transport	1.00 * prostaglandin-g2 [e] --> 1.00 * prostaglandin-g2 [c]	1.00 * pros	Transport, extracellular			0
r2368_neg	1	0.19043	0.7202646	-1.97541	1.975412	2	0.347218	r2368	Organic anion transporter 5 Utilized transport	1.00 * prostaglandin H2(1-) [e] --> 1.00 * prostaglandin H2	1.00 * pros	Transport, extracellular			0
r2368_pos	1	0.19043	0.7202646	-1.97541	1.975412	2	0.347218	r2368	Organic anion transporter 5 Utilized transport	1.00 * prostaglandin H2(1-) [e] --> 1.00 * prostaglandin H2	1.00 * pros	Transport, extracellular			0
r2369_neg	1	0.19043	0.7202646	-1.97541	1.975412	2	0.347218	r2369	Organic anion transporter 5 Utilized transport	1.00 * prostaglandin I2(1-) [e] --> 1.00 * prostaglandin I2(	1.00 * pros	Transport, extracellular			0
r2369_pos	1	0.19043	0.7202646	-1.97541	1.975412	2	0.347218	r2369	Organic anion transporter 5 Utilized transport	1.00 * prostaglandin I2(1-) [e] --> 1.00 * prostaglandin I2(	1.00 * pros	Transport, extracellular			0
r2465_pos	8	0.19043	0.7202646	1.686657	1.686657	1	0.347218	r2465	Cation Diffusion Facilitator (CDF) TCDB:2.A.4.2.3	1.00 * Zinc [c] --> 1.00 * Zinc [e]SLC30A1	SLC30A1	Transport, extracellular			0
r2535_pos	8	0.19043	0.7202646	1.197693	1.197693	1	0.347218	r2535	Major Facilitator(MFS) TCDB:2.A.1.44.1	1.00 * L-homoserine [e] --> 1.00 * L-homoserine [c]SLC43A	SLC43A1	Transport, extracellular			0
2HBt2_neg	2	0.382733	0.4171041	-0.82228	0.82228	2	0.518179	2HBt2	2-hydroxybutyrate cotransport with proton	1.00 * 2-Hydroxybutyrate [e] + 1.00 * proton [e] --> 1.00 *	SLC16A1; S	Transport, extracellular			4
2HBt2_pos	2	0.382733	0.4171041	-0.82228	0.82228	2	0.518179	2HBt2	2-hydroxybutyrate cotransport with proton	1.00 * 2-Hydroxybutyrate [e] + 1.00 * proton [e] --> 1.00 *	SLC16A1; S	Transport, extracellular			4
2HCO3_NAt_pos	2	0.382733	0.4171041	-0.51537	0.515372	2	0.518179	2HCO3_NA	bicarbonate transport (Na/HCO3 1:2 cotransport)	2.00 * Bicarbonate [e] + 1.00 * Sodium [e] --> 2.00 * Bicarl	SLC4A4; SL	Transport, extracellular			4
ABUTt4_2_r_pos	2	0.382733	0.4171041	-1.34621	1.346206	2	0.518179	ABUTt4_2_	4-aminobutyrate reversible transport in via sodium symport (1:2)	2.00 * Sodium [e] + 1.00 * 4-Aminobutanoate [e] --> 2.00	SLC6A1; SL	Transport, extracellular			4
ACACt2_neg	2	0.382733	0.4171041	-0.83341	0.833415	2	0.518179	ACACt2	acetoacetate transport via proton symport	1.00 * proton [e] + 1.00 * acetoacetate [e] --> 1.00 * proto	SLC16A1; S	Transport, extracellular			4
ACACt2_pos	2	0.382733	0.4171041	-0.83341	0.833415	2	0.518179	ACACt2	acetoacetate transport via proton symport	1.00 * proton [e] + 1.00 * acetoacetate [e] --> 1.00 * proto	SLC16A1; S	Transport, extracellular			4
ACt2r_neg	2	0.382733	0.4171041	-1.10346	1.103457	2	0.518179	ACt2r	acetate reversible transport via proton symport	1.00 * proton [e] + 1.00 * acetate [e] --> 1.00 * proton [c] +	1.00 * prot	Transport, extracellular			4
ACt2r_pos	2	0.382733	0.4171041	-1.10346	1.103457	2	0.518179	ACt2r	acetate reversible transport via proton symport	1.00 * proton [e] + 1.00 * acetate [e] --> 1.00 * proton [c] +	1.00 * prot	Transport, extracellular			4
ASCBt4_pos	2	0.382733	0.4171041	-0.86601	0.866007	2	0.518179	ASCBt4	L-ascorbate transport via proton symport	1.00 * Sodium [e] + 1.00 * L-ascorbate [e] --> 1.00 * Sodiu	SLC23A1; S	Transport, extracellular			4
ASNALANaEx_pos	7	0.382733	0.4171041	1.479592	1.479592	1	0.518179	ASNALANaE	L-alanine/L-asparagine Na-dependent exchange (Asn-L in)	1.00 * Sodium [e] + 1.00 * L-alanine [c] + 1.00 * L-asparagi	SLC1A5	Transport, extracellular			4
ASNGLNNaEx_pos	7	0.382733	0.4171041	1.356376	1.356376	1	0.518179	ASNGLNNa	L-glutamine/L-asparagine Na-dependent exchange (Asn-L in)	1.00 * Sodium [e] + 1.00 * L-asparagine [e] + 1.00 * L-gluta	SLC1A5	Transport, extracellular			4
ASNtN1_neg	2	0.382733	0.4171041	-1.05893	1.058928	2	0.518179	ASNtN1	Asparagine transport (Na, H coupled)	1.00 * proton [c] + 2.00 * Sodium [e] + 1.00 * L-asparagine	SLC38A3; S	Transport, extracellular			4
BUTt2r_neg	2	0.382733	0.4171041	-1.3338	1.333799	2	0.518179	BUTt2r	Butyrate transport via proton symport, reversible	1.00 * proton [e] + 1.00 * butyrate [e] --> 1.00 * proton [c]	SLC16A1	Transport, extracellular			4
BUTt2r_pos	2	0.382733	0.4171041	-1.3338	1.333799	2	0.518179	BUTt2r	Butyrate transport via proton symport, reversible	1.00 * proton [e] + 1.00 * butyrate [e] --> 1.00 * proton [c]	SLC16A1	Transport, extracellular			4
CREATt4_2_r_pos	7	0.382733	0.4171041	0.688468	0.688468	1	0.518179	CREATt4_2	Creatine transport (sodium symport) (2:1)	2.00 * Sodium [e] + 1.00 * creatine [e] --> 2.00 * Sodium [c	SLC6A8	Transport, extracellular			4
CYSASNNaEx_pos	7	0.382733	0.4171041	1.197484	1.197484	1	0.518179	CYSASNNaE	L-cysteine/L-asparagine Na-dependent exchange (Cys-L in)	1.00 * Sodium [e] + 1.00 * L-asparagine [c] + 1.00 * L-cyste	SLC1A5	Transport, extracellular			4
CYSGLNNaEx_pos	7	0.382733	0.4171041	1.197484	1.197484	1	0.518179	CYSGLNNaE	L-cysteine/L-glutamine Na-dependent exchange (Cys-L in)	1.00 * Sodium [e] + 1.00 * L-cysteine [e] + 1.00 * L-glutami	SLC1A5	Transport, extracellular			4
CYSTLEUrBATtc_pos	2	0.382733	0.4171041	-1.07129	1.071291	2	0.518179	CYSTLEUrBA	transport of L-Cystine into the cell in exchange for L-Leucine by b0,+	1.00 * L-cystine [e] + 1.00 * L-leucine [c] --> 1.00 * L-cystin	SLC3A1; SL	Transport, extracellular			4
CYSTSERex_pos	2	0.382733	0.4171041	-1.04745	1.04745	2	0.518179	CYSTSERex	L-cystine/glycine exchanger (cystine in)	1.00 * L-serine [c] + 1.00 * L-cystine [e] --> 1.00 * L-serine [	SLC3A1; SL	Transport, extracellular			4
CYStec_neg	7	0.382733	0.4171041	0.389589	0.389589	1	0.518179	CYStec	L-cysteine transport via diffusion (extracellular to cytosol)	1.00 * L-cysteine [e] --> 1.00 * L-cysteine [c]SLC43A2	SLC43A2	Transport, extracellular			4
CYStec_pos	7	0.382733	0.4171041	0.632644	0.632644	1	0.518179	CYStec	L-cysteine transport via diffusion (extracellular to cytosol)	1.00 * L-cysteine [e] --> 1.00 * L-cysteine [c]SLC43A2	SLC43A2	Transport, extracellular			4
CYTDt_pos	2	0.382733	0.4171041	-1.43165	1.431651	2	0.518179	CYTDt	cytidine facilated transport in cytosol	1.00 * Cytidine [e] --> 1.00 * Cytidine [c]SLC29A1; SLC29A	SLC29A1; S	Transport, extracellular			4
DOPAtu_neg	2	0.382733	0.4171041	-0.3751	0.375097	2	0.518179	DOPAtu	Dopamine uniport	1.00 * dopaminium(1+) [e] --> 1.00 * dopaminium(1+) [c]S	SLC22A2; S	Transport, extracellular			4
DOPAtu_pos	2	0.382733	0.4171041	-0.99699	0.996991	2	0.518179	DOPAtu	Dopamine uniport	1.00 * dopaminium(1+) [e] --> 1.00 * dopaminium(1+) [c]S	SLC22A2; S	Transport, extracellular			4
FATP1t_pos	2	0.382733	0.4171041	-0.72451	0.724511	2	0.518179	FATP1t	fatty acid electroneutral transport	1.00 * Sodium [c] + 1.00 * Hexadecanoate (n-C16:0) [c] -->	SLC27A1; S	Transport, extracellular			4
FATP2t_neg	2	0.382733	0.4171041	-0.76468	0.764685	2	0.518179	FATP2t	fatty acid electroneutral transport	1.00 * Sodium [c] + 1.00 * octadecenoate (n-C18:1) [c] -->	SLC27A1	Transport, extracellular			4
FATP2t_pos	2	0.382733	0.4171041	-1.00027	1.000272	2	0.518179	FATP2t	fatty acid electroneutral transport	1.00 * Sodium [c] + 1.00 * octadecenoate (n-C18:1) [c] -->	SLC27A1	Transport, extracellular			4
FATP3t_pos	2	0.382733	0.4171041	-0.89073	0.89073	2	0.518179	FATP3t	fatty acid electroneutral transport	1.00 * Sodium [c] + 1.00 * octadecanoate (n-C18:0) [c] -->	SLC27A1	Transport, extracellular			4
FATP4t_pos	2	0.382733	0.4171041	-1.47086	1.470858	2	0.518179	FATP4t	fatty acid electroneutral transport	1.00 * Sodium [c] + 1.00 * arachidate [c] --> 1.00 * Sodium	SLC27A5	Transport, extracellular			4
FATP6t_pos	2	0.382733	0.4171041	-1.11579	1.115788	2	0.518179	FATP6t	fatty acid electroneutral transport	1.00 * Sodium [c] + 1.00 * adrenic acid [c] --> 1.00 * Sodiu	SLC27A2; S	Transport, extracellular			4
FATP7t_pos	2	0.382733	0.4171041	-1.11579	1.115788	2	0.518179	FATP7t	fatty acid electroneutral transport	1.00 * Sodium [c] + 1.00 * cervonic acid, C22:6 n-3 [c] -->	SLC27A2; S	Transport, extracellular			4
FATP9t_pos	2	0.382733	0.4171041	-1.11579	1.115788	2	0.518179	FATP9t	fatty acid electroneutral transport	1.00 * Sodium [c] + 1.00 * nervonate [c] --> 1.00 * Sodium	SLC27A2; S	Transport, extracellular			4
GLCt4_pos	2	0.382733	0.4171041	-1.01431	1.014308	2	0.518179	GLCt4	glucose transport via sodium symport	1.00 * Sodium [e] + 1.00 * D-glucose [e] --> 1.00 * Sodium	SLC5A10; S	Transport, extracellular			4
GLNASNNaEx_pos	7	0.382733	0.4171041	1.356376	1.356376	1	0.518179	GLNASNNa	L-glutamine/L-asparagine Na-dependent exchange (Gln-L in)	1.00 * Sodium [e] + 1.00 * L-asparagine [c] + 1.00 * L-gluta	SLC1A5	Transport, extracellular			4
GLNCYSNaEx_pos	7	0.382733	0.4171041	1.088228	1.088228	1	0.518179	GLNCYSNaE	L-cysteine/L-glutamine Na-dependent exchange (Gln-L in)	1.00 * Sodium [e] + 1.00 * L-cysteine [c] + 1.00 * L-glutami	SLC1A5	Transport, extracellular			4
GLNtN1_neg	2	0.382733	0.4171041	-1.05893	1.058928	2	0.518179	GLNtN1	Glutamine transport (Na, H coupled)	1.00 * proton [c] + 2.00 * Sodium [e] + 1.00 * L-glutamine	SLC38A3; S	Transport, extracellular			4
GLYSNAT5tc_pos	7	0.382733	0.4171041	0.974459	0.974459	1	0.518179	GLYSNAT5t	transport of Glycine into the cell coupled with co-transport with So	1.00 * proton [c] + 1.00 * Sodium [e] + 1.00 * Glycine [e] -	SLC38A5	Transport, extracellular			4
GSNt_neg	2	0.382733	0.4171041	-1.39802	1.398016	2	0.518179	GSNt	guanosine facilated transport in cytosol	1.00 * Guanosine [e] --> 1.00 * Guanosine [c]SLC29A1; SL	SLC29A1; S	Transport, extracellular			4
GSNt_pos	2	0.382733	0.4171041	-1.43165	1.431651	2	0.518179	GSNt	guanosine facilated transport in cytosol	1.00 * Guanosine [e] --> 1.00 * Guanosine [c]SLC29A1; SL	SLC29A1; S	Transport, extracellular			4
ILEATB0tc_pos	7	0.382733	0.4171041	1.004822	1.004822	1	0.518179	ILEATB0tc	Transport of L-Isoleucine into the intestinal cells by ATB0 transporte	2.00 * Sodium [e] + 1.00 * Chloride [e] + 1.00 * L-isoleucin	SLC6A14	Transport, extracellular			4
INSt_neg	2	0.382733	0.4171041	-1.39802	1.398016	2	0.518179	INSt	Inosine transport (diffusion)	1.00 * Inosine [e] --> 1.00 * Inosine [c]SLC29A1; SLC29A2	SLC29A1; S	Transport, extracellular			4
INSt_pos	2	0.382733	0.4171041	-1.39884	1.398841	2	0.518179	INSt	Inosine transport (diffusion)	1.00 * Inosine [e] --> 1.00 * Inosine [c]SLC29A1; SLC29A2	SLC29A1; S	Transport, extracellular			4
KCC2t_neg	7	0.382733	0.4171041	0.882528	0.882528	1	0.518179	KCC2t	K+-Cl- cotransporter (NH4+)	1.00 * Ammonium [e] + 1.00 * Chloride [e] --> 1.00 * Amm	SLC12A4; S	Transport, extracellular			4
KCC2t_pos	7	0.382733	0.4171041	0.882528	0.882528	1	0.518179	KCC2t	K+-Cl- cotransporter (NH4+)	1.00 * Ammonium [e] + 1.00 * Chloride [e] --> 1.00 * Amm	SLC12A4; S	Transport, extracellular			4
LEUATB0tc_pos	7	0.382733	0.4171041	0.973951	0.973951	1	0.518179	LEUATB0tc	Transport of L-Leucine into the intestinal cells by ATB0 transporter	2.00 * Sodium [e] + 1.00 * Chloride [e] + 1.00 * L-leucine [	SLC6A14	Transport, extracellular			4
L_LACt2r_neg	2	0.382733	0.4171041	-0.82228	0.82228	2	0.518179	L_LACt2r	L-lactate reversible transport via proton symport	1.00 * proton [e] + 1.00 * (S)-lactate [e] --> 1.00 * proton [	BSG; SLC16	Transport, extracellular			4
L_LACt2r_pos	2	0.382733	0.4171041	-0.82228	0.82228	2	0.518179	L_LACt2r	L-lactate reversible transport via proton symport	1.00 * proton [e] + 1.00 * (S)-lactate [e] --> 1.00 * proton [	BSG; SLC16	Transport, extracellular			4
PHEt4_pos	7	0.382733	0.4171041	0.866035	0.866035	1	0.518179	PHEt4	L-phenylalanine transport in via sodium symport	1.00 * Sodium [e] + 1.00 * L-phenylalanine [e] --> 1.00 * So	ACE2; SLC3	Transport, extracellular			4
PROt4_2_r_pos	2	0.382733	0.4171041	-1.60504	1.60504	2	0.518179	PROt4_2_r	Proline transport (sodium symport) (2:1)	2.00 * Sodium [e] + 1.00 * L-proline [e] --> 2.00 * Sodium [	SLC6A7	Transport, extracellular			4
PYRt2r_neg	2	0.382733	0.4171041	-0.82228	0.82228	2	0.518179	PYRt2r	pyruvate reversible transport via proton symport	1.00 * proton [e] + 1.00 * pyruvate [e] --> 1.00 * proton [c]	SLC16A1; S	Transport, extracellular			4
PYRt2r_pos	2	0.382733	0.4171041	-0.82228	0.82228	2	0.518179	PYRt2r	pyruvate reversible transport via proton symport	1.00 * proton [e] + 1.00 * pyruvate [e] --> 1.00 * proton [c]	SLC16A1; S	Transport, extracellular			4
SERATB0tc_pos	7	0.382733	0.4171041	1.003441	1.003441	1	0.518179	SERATB0tc	transport of L-Serine into the intestinal cells by ATB0 transporter	2.00 * Sodium [e] + 1.00 * L-serine [e] + 1.00 * Chloride [e]	SLC6A14	Transport, extracellular			4
SRTNtu_neg	2	0.382733	0.4171041	-0.88333	0.883326	2	0.518179	SRTNtu	Serotonin uniport	1.00 * serotonin(1+) [e] --> 1.00 * serotonin(1+) [c]SLC22A	SLC22A1; S	Transport, extracellular			4
TAUBETAtc_pos	7	0.382733	0.4171041	0.962828	0.962828	1	0.518179	TAUBETAtc	transport of taurine into the intestinal cells by beta transport system	2.00 * Sodium [e] + 1.00 * Chloride [e] + 1.00 * Taurine [e]	SLC6A6	Transport, extracellular			4
TAURt4_2_r_neg	7	0.382733	0.4171041	0.95223	0.95223	1	0.518179	TAURt4_2_	taurine transport (sodium symport) (2:1)	2.00 * Sodium [e] + 1.00 * Taurine [e] --> 2.00 * Sodium [c]	SLC6A6	Transport, extracellular			4
THMDt4_pos	2	0.382733	0.4171041	-1.36096	1.36096	2	0.518179	THMDt4	thymidine transport in via sodium symport	1.00 * Sodium [e] + 1.00 * Thymidine [e] --> 1.00 * Sodium	SLC28A1; S	Transport, extracellular			4
THRATB0tc_pos	7	0.382733	0.4171041	1.003441	1.003441	1	0.518179	THRATB0tc	Transport of L-Threonine into the intestinal cells by ATB0 transporte	2.00 * Sodium [e] + 1.00 * L-threonine [e] + 1.00 * Chlorid	SLC6A14	Transport, extracellular			4
THYMDt1_pos	2	0.382733	0.4171041	-1.43165	1.431651	2	0.518179	THYMDt1	thymd transport	1.00 * Thymidine [e] --> 1.00 * Thymidine [c]SLC29A1; SL	SLC29A1; S	Transport, extracellular			4
TRPATB0tc_pos	7	0.382733	0.4171041	1.09908	1.09908	1	0.518179	TRPATB0tc	transport of L-Tryptophan into the intestinal cells by ATB0 transport	2.00 * Sodium [e] + 1.00 * Chloride [e] + 1.00 * L-tryptoph	SLC6A14	Transport, extracellular			4
URIt_pos	2	0.382733	0.4171041	-1.43165	1.431651	2	0.518179	URIt	uridine facilated transport in cytosol	1.00 * Uridine [e] --> 1.00 * Uridine [c]SLC29A1; SLC29A2	SLC29A1; S	Transport, extracellular			4
VALATB0tc_pos	7	0.382733	0.4171041	1.004822	1.004822	1	0.518179	VALATB0tc	transport of L-Valine into the intestinal cells by ATB0 transporter	2.00 * Sodium [e] + 1.00 * Chloride [e] + 1.00 * L-valine [e]	SLC6A14	Transport, extracellular			4
r0839_neg	7	0.382733	0.4171041	0.935747	0.935747	1	0.518179	r0839	Postulated transport reaction	1.00 * Orotate [e] --> 1.00 * Orotate [c]No genes	1.00 * Orot	Transport, extracellular			0
r0839_pos	7	0.382733	0.4171041	0.754536	0.754536	1	0.518179	r0839	Postulated transport reaction	1.00 * Orotate [e] --> 1.00 * Orotate [c]No genes	1.00 * Orot	Transport, extracellular			0
r0942_pos	7	0.382733	0.4171041	0.688468	0.688468	1	0.518179	r0942	Neurotransmitter:Sodium Symporter (NSS) TCDB:2.A.22.3.4	1.00 * creatine [c] --> 1.00 * creatine [e]SLC6A8	SLC6A8	Transport, extracellular			0
r1144_pos	7	0.382733	0.4171041	1.033906	1.033906	1	0.518179	r1144	Major Facilitator(MFS) TCDB:2.A.18.6.3	1.00 * Sodium [e] + 1.00 * L-glutamate(1-) [e] --> 1.00 * So	SLC38A3	Transport, extracellular			0
r1613_neg	7	0.382733	0.4171041	1.142433	1.142433	1	0.518179	r1613	Amino Acid-Polyamine-Organocation (APC) TCDB:2.A.3.8.1	1.00 * L-asparagine [c] + 1.00 * L-tryptophan [e] --> 1.00 *	SLC7A5	Transport, extracellular			0
r1613_pos	7	0.382733	0.4171041	1.142433	1.142433	1	0.518179	r1613	Amino Acid-Polyamine-Organocation (APC) TCDB:2.A.3.8.1	1.00 * L-asparagine [c] + 1.00 * L-tryptophan [e] --> 1.00 *	SLC7A5	Transport, extracellular			0
r1614_neg	7	0.382733	0.4171041	1.185461	1.185461	1	0.518179	r1614	Amino Acid-Polyamine-Organocation (APC) TCDB:2.A.3.8.1	1.00 * L-tryptophan [e] + 1.00 * L-valine [c] --> 1.00 * L-val	SLC7A5	Transport, extracellular			0
r1614_pos	7	0.382733	0.4171041	1.185461	1.185461	1	0.518179	r1614	Amino Acid-Polyamine-Organocation (APC) TCDB:2.A.3.8.1	1.00 * L-tryptophan [e] + 1.00 * L-valine [c] --> 1.00 * L-val	SLC7A5	Transport, extracellular			0
r1615_neg	7	0.382733	0.4171041	1.142433	1.142433	1	0.518179	r1615	Amino Acid-Polyamine-Organocation (APC) TCDB:2.A.3.8.1	1.00 * L-threonine [c] + 1.00 * L-tryptophan [e] --> 1.00 *	SLC7A5	Transport, extracellular			0
r1615_pos	7	0.382733	0.4171041	1.142433	1.142433	1	0.518179	r1615	Amino Acid-Polyamine-Organocation (APC) TCDB:2.A.3.8.1	1.00 * L-threonine [c] + 1.00 * L-tryptophan [e] --> 1.00 *	SLC7A5	Transport, extracellular			0
r1616_neg	7	0.382733	0.4171041	1.142433	1.142433	1	0.518179	r1616	Amino Acid-Polyamine-Organocation (APC) TCDB:2.A.3.8.1	1.00 * L-tryptophan [e] + 1.00 * L-homoserine [c] --> 1.00	SLC7A5	Transport, extracellular			0
r1616_pos	7	0.382733	0.4171041	1.142433	1.142433	1	0.518179	r1616	Amino Acid-Polyamine-Organocation (APC) TCDB:2.A.3.8.1	1.00 * L-tryptophan [e] + 1.00 * L-homoserine [c] --> 1.00	SLC7A5	Transport, extracellular			0

r1617_neg	7	0.382733	0.4171041	1.185461	1.185461	1	0.518179	r1617	Amino Acid-Polyamine-Organocation (APC) TCDB:2.A.3.8.1	1.00 * L-tryptophan [e] + 1.00 * L-isoleucine [c] --> 1.00 *	SLC7A5	Transport, extracellular			0
r1617_pos	7	0.382733	0.4171041	1.185461	1.185461	1	0.518179	r1617	Amino Acid-Polyamine-Organocation (APC) TCDB:2.A.3.8.1	1.00 * L-tryptophan [e] + 1.00 * L-isoleucine [c] --> 1.00 *	SLC7A5	Transport, extracellular			0
r1621_pos	7	0.382733	0.4171041	1.328521	1.328521	1	0.518179	r1621	Amino Acid-Polyamine-Organocation (APC) TCDB:2.A.3.8.1	1.00 * L-phenylalanine [e] + 1.00 * L-proline [c] --> 1.00 *	SLC7A5	Transport, extracellular			0
r2079_neg	2	0.382733	0.4171041	-0.83341	0.833415	2	0.518179	r2079	Major Facilitator(MFS) TCDB:2.A.1.13.1	1.00 * acetoacetate [c] + 1.00 * pyruvate [e] --> 1.00 * pyru	SLC16A1	Transport, extracellular			0
r2079_pos	2	0.382733	0.4171041	-0.83341	0.833415	2	0.518179	r2079	Major Facilitator(MFS) TCDB:2.A.1.13.1	1.00 * acetoacetate [c] + 1.00 * pyruvate [e] --> 1.00 * pyru	SLC16A1	Transport, extracellular			0
r2080_neg	2	0.382733	0.4171041	-1.64322	1.643222	2	0.518179	r2080	Major Facilitator(MFS) TCDB:2.A.1.13.1	1.00 * acetoacetate [c] + 1.00 * acetate [e] --> 1.00 * aceto	SLC16A1	Transport, extracellular			0
r2080_pos	2	0.382733	0.4171041	-1.64322	1.643222	2	0.518179	r2080	Major Facilitator(MFS) TCDB:2.A.1.13.1	1.00 * acetoacetate [c] + 1.00 * acetate [e] --> 1.00 * aceto	SLC16A1	Transport, extracellular			0
r2081_neg	2	0.382733	0.4171041	-1.64322	1.643222	2	0.518179	r2081	Major Facilitator(MFS) TCDB:2.A.1.13.1	1.00 * acetoacetate [c] + 1.00 * Formate [e] --> 1.00 * acet	SLC16A1	Transport, extracellular			0
r2081_pos	2	0.382733	0.4171041	-1.64322	1.643222	2	0.518179	r2081	Major Facilitator(MFS) TCDB:2.A.1.13.1	1.00 * acetoacetate [c] + 1.00 * Formate [e] --> 1.00 * acet	SLC16A1	Transport, extracellular			0
r2082_neg	2	0.382733	0.4171041	-1.64322	1.643222	2	0.518179	r2082	Major Facilitator(MFS) TCDB:2.A.1.13.1	1.00 * acetoacetate [c] + 1.00 * propionate [e] --> 1.00 * a	SLC16A1	Transport, extracellular			0
r2082_pos	2	0.382733	0.4171041	-1.64322	1.643222	2	0.518179	r2082	Major Facilitator(MFS) TCDB:2.A.1.13.1	1.00 * acetoacetate [c] + 1.00 * propionate [e] --> 1.00 * a	SLC16A1	Transport, extracellular			0
r2083_neg	2	0.382733	0.4171041	-0.83341	0.833415	2	0.518179	r2083	Major Facilitator(MFS) TCDB:2.A.1.13.1	1.00 * acetoacetate [c] + 1.00 * (S)-lactate [e] --> 1.00 * ace	SLC16A1	Transport, extracellular			0
r2083_pos	2	0.382733	0.4171041	-0.83341	0.833415	2	0.518179	r2083	Major Facilitator(MFS) TCDB:2.A.1.13.1	1.00 * acetoacetate [c] + 1.00 * (S)-lactate [e] --> 1.00 * ace	SLC16A1	Transport, extracellular			0
r2084_neg	2	0.382733	0.4171041	-1.6311	1.631095	2	0.518179	r2084	Major Facilitator(MFS) TCDB:2.A.1.13.1	1.00 * acetoacetate [c] + 1.00 * all-trans-retinoate [e] --> 1	SLC16A1	Transport, extracellular			0
r2084_pos	2	0.382733	0.4171041	-1.6311	1.631095	2	0.518179	r2084	Major Facilitator(MFS) TCDB:2.A.1.13.1	1.00 * acetoacetate [c] + 1.00 * all-trans-retinoate [e] --> 1	SLC16A1	Transport, extracellular			0
r2085_neg	2	0.382733	0.4171041	-0.83341	0.833415	2	0.518179	r2085	Major Facilitator(MFS) TCDB:2.A.1.13.1	1.00 * 2-Hydroxybutyrate [e] + 1.00 * acetoacetate [c] -->	SLC16A1	Transport, extracellular			0
r2085_pos	2	0.382733	0.4171041	-0.83341	0.833415	2	0.518179	r2085	Major Facilitator(MFS) TCDB:2.A.1.13.1	1.00 * 2-Hydroxybutyrate [e] + 1.00 * acetoacetate [c] -->	SLC16A1	Transport, extracellular			0
r2086_neg	2	0.382733	0.4171041	-0.82228	0.82228	2	0.518179	r2086	Major Facilitator(MFS) TCDB:2.A.1.13.1	1.00 * 2-Hydroxybutyrate [c] + 1.00 * pyruvate [e] --> 1.00	SLC16A1	Transport, extracellular			0
r2086_pos	2	0.382733	0.4171041	-0.82228	0.82228	2	0.518179	r2086	Major Facilitator(MFS) TCDB:2.A.1.13.1	1.00 * 2-Hydroxybutyrate [c] + 1.00 * pyruvate [e] --> 1.00	SLC16A1	Transport, extracellular			0
r2087_neg	2	0.382733	0.4171041	-1.64322	1.643222	2	0.518179	r2087	Major Facilitator(MFS) TCDB:2.A.1.13.1	1.00 * 2-Hydroxybutyrate [c] + 1.00 * acetate [e] --> 1.00	SLC16A1	Transport, extracellular			0
r2087_pos	2	0.382733	0.4171041	-1.64322	1.643222	2	0.518179	r2087	Major Facilitator(MFS) TCDB:2.A.1.13.1	1.00 * 2-Hydroxybutyrate [c] + 1.00 * acetate [e] --> 1.00	SLC16A1	Transport, extracellular			0
r2088_neg	2	0.382733	0.4171041	-1.64322	1.643222	2	0.518179	r2088	Major Facilitator(MFS) TCDB:2.A.1.13.1	1.00 * 2-Hydroxybutyrate [c] + 1.00 * Formate [e] --> 1.00	SLC16A1	Transport, extracellular			0
r2088_pos	2	0.382733	0.4171041	-1.64322	1.643222	2	0.518179	r2088	Major Facilitator(MFS) TCDB:2.A.1.13.1	1.00 * 2-Hydroxybutyrate [c] + 1.00 * Formate [e] --> 1.00	SLC16A1	Transport, extracellular			0
r2089_neg	2	0.382733	0.4171041	-1.64322	1.643222	2	0.518179	r2089	Major Facilitator(MFS) TCDB:2.A.1.13.1	1.00 * 2-Hydroxybutyrate [c] + 1.00 * propionate [e] --> 1.	SLC16A1	Transport, extracellular			0
r2089_pos	2	0.382733	0.4171041	-1.64322	1.643222	2	0.518179	r2089	Major Facilitator(MFS) TCDB:2.A.1.13.1	1.00 * 2-Hydroxybutyrate [c] + 1.00 * propionate [e] --> 1.	SLC16A1	Transport, extracellular			0
r2090_neg	2	0.382733	0.4171041	-0.82228	0.82228	2	0.518179	r2090	Major Facilitator(MFS) TCDB:2.A.1.13.1	1.00 * 2-Hydroxybutyrate [c] + 1.00 * (S)-lactate [e] --> 1.0	SLC16A1	Transport, extracellular			0
r2090_pos	2	0.382733	0.4171041	-0.82228	0.82228	2	0.518179	r2090	Major Facilitator(MFS) TCDB:2.A.1.13.1	1.00 * 2-Hydroxybutyrate [c] + 1.00 * (S)-lactate [e] --> 1.0	SLC16A1	Transport, extracellular			0
r2091_neg	2	0.382733	0.4171041	-1.3338	1.333799	2	0.518179	r2091	Major Facilitator(MFS) TCDB:2.A.1.13.1	1.00 * 2-Hydroxybutyrate [c] + 1.00 * butyrate [e] --> 1.00	SLC16A1	Transport, extracellular			0
r2091_pos	2	0.382733	0.4171041	-1.3338	1.333799	2	0.518179	r2091	Major Facilitator(MFS) TCDB:2.A.1.13.1	1.00 * 2-Hydroxybutyrate [c] + 1.00 * butyrate [e] --> 1.00	SLC16A1	Transport, extracellular			0
r2092_neg	2	0.382733	0.4171041	-1.6311	1.631095	2	0.518179	r2092	Major Facilitator(MFS) TCDB:2.A.1.13.1	1.00 * 2-Hydroxybutyrate [c] + 1.00 * all-trans-retinoate [	SLC16A1	Transport, extracellular			0
r2092_pos	2	0.382733	0.4171041	-1.6311	1.631095	2	0.518179	r2092	Major Facilitator(MFS) TCDB:2.A.1.13.1	1.00 * 2-Hydroxybutyrate [c] + 1.00 * all-trans-retinoate [	SLC16A1	Transport, extracellular			0
r2094_neg	2	0.382733	0.4171041	-1.64322	1.643222	2	0.518179	r2094	Major Facilitator(MFS) TCDB:2.A.1.13.1	1.00 * acetate [c] + 1.00 * pyruvate [e] --> 1.00 * pyruvate	SLC16A1	Transport, extracellular			0
r2094_pos	2	0.382733	0.4171041	-1.64322	1.643222	2	0.518179	r2094	Major Facilitator(MFS) TCDB:2.A.1.13.1	1.00 * acetate [c] + 1.00 * pyruvate [e] --> 1.00 * pyruvate	SLC16A1	Transport, extracellular			0
r2095_neg	2	0.382733	0.4171041	-1.64322	1.643222	2	0.518179	r2095	Major Facilitator(MFS) TCDB:2.A.1.13.1	1.00 * Formate [c] + 1.00 * pyruvate [e] --> 1.00 * pyruvate	SLC16A1	Transport, extracellular			0
r2095_pos	2	0.382733	0.4171041	-1.64322	1.643222	2	0.518179	r2095	Major Facilitator(MFS) TCDB:2.A.1.13.1	1.00 * Formate [c] + 1.00 * pyruvate [e] --> 1.00 * pyruvate	SLC16A1	Transport, extracellular			0
r2096_neg	2	0.382733	0.4171041	-1.64322	1.643222	2	0.518179	r2096	Major Facilitator(MFS) TCDB:2.A.1.13.1	1.00 * propionate [c] + 1.00 * pyruvate [e] --> 1.00 * pyru	SLC16A1	Transport, extracellular			0
r2096_pos	2	0.382733	0.4171041	-1.64322	1.643222	2	0.518179	r2096	Major Facilitator(MFS) TCDB:2.A.1.13.1	1.00 * propionate [c] + 1.00 * pyruvate [e] --> 1.00 * pyru	SLC16A1	Transport, extracellular			0
r2097_neg	2	0.382733	0.4171041	-0.82228	0.82228	2	0.518179	r2097	Major Facilitator(MFS) TCDB:2.A.1.13.1	1.00 * pyruvate [e] + 1.00 * (S)-lactate [c] --> 1.00 * pyruva	SLC16A1	Transport, extracellular			0
r2097_pos	2	0.382733	0.4171041	-0.82228	0.82228	2	0.518179	r2097	Major Facilitator(MFS) TCDB:2.A.1.13.1	1.00 * pyruvate [e] + 1.00 * (S)-lactate [c] --> 1.00 * pyruva	SLC16A1	Transport, extracellular			0
r2098_neg	2	0.382733	0.4171041	-1.3338	1.333799	2	0.518179	r2098	Major Facilitator(MFS) TCDB:2.A.1.13.1	1.00 * butyrate [c] + 1.00 * pyruvate [e] --> 1.00 * pyruvat	SLC16A1	Transport, extracellular			0
r2098_pos	2	0.382733	0.4171041	-1.3338	1.333799	2	0.518179	r2098	Major Facilitator(MFS) TCDB:2.A.1.13.1	1.00 * butyrate [c] + 1.00 * pyruvate [e] --> 1.00 * pyruvat	SLC16A1	Transport, extracellular			0
r2099_neg	2	0.382733	0.4171041	-1.6311	1.631095	2	0.518179	r2099	Major Facilitator(MFS) TCDB:2.A.1.13.1	1.00 * pyruvate [e] + 1.00 * all-trans-retinoate [c] --> 1.00	SLC16A1	Transport, extracellular			0
r2099_pos	2	0.382733	0.4171041	-1.6311	1.631095	2	0.518179	r2099	Major Facilitator(MFS) TCDB:2.A.1.13.1	1.00 * pyruvate [e] + 1.00 * all-trans-retinoate [c] --> 1.00	SLC16A1	Transport, extracellular			0
r2100_neg	2	0.382733	0.4171041	-1.64322	1.643222	2	0.518179	r2100	Major Facilitator(MFS) TCDB:2.A.1.13.1	1.00 * (R)-3-hydroxybutyrate [c] + 1.00 * pyruvate [e] --> 1	SLC16A1	Transport, extracellular			0
r2100_pos	2	0.382733	0.4171041	-1.64322	1.643222	2	0.518179	r2100	Major Facilitator(MFS) TCDB:2.A.1.13.1	1.00 * (R)-3-hydroxybutyrate [c] + 1.00 * pyruvate [e] --> 1	SLC16A1	Transport, extracellular			0
r2101_neg	7	0.382733	0.4171041	0.449832	0.449832	1	0.518179	r2101	Major Facilitator(MFS) TCDB:2.A.1.13.1	1.00 * acetate [c] + 1.00 * 2-Oxoglutarate [e] --> 1.00 * 2-O	SLC16A1	Transport, extracellular			0
r2103_neg	7	0.382733	0.4171041	0.449832	0.449832	1	0.518179	r2103	Major Facilitator(MFS) TCDB:2.A.1.13.1	1.00 * propionate [c] + 1.00 * 2-Oxoglutarate [e] --> 1.00 *	SLC16A1	Transport, extracellular			0
r2106_neg	7	0.382733	0.4171041	0.504348	0.504348	1	0.518179	r2106	Major Facilitator(MFS) TCDB:2.A.1.13.1	1.00 * 2-Oxoglutarate [e] + 1.00 * butyrate [c] --> 1.00 * 2-	SLC16A1	Transport, extracellular			0
r2107_pos	2	0.382733	0.4171041	-0.60529	0.605294	2	0.518179	r2107	Major Facilitator(MFS) TCDB:2.A.1.13.1	1.00 * 2-Oxoglutarate [e] + 1.00 * all-trans-retinoate [c] --	SLC16A1	Transport, extracellular			0
r2108_neg	7	0.382733	0.4171041	0.449832	0.449832	1	0.518179	r2108	Major Facilitator(MFS) TCDB:2.A.1.13.1	1.00 * 2-Oxoglutarate [e] + 1.00 * (R)-3-hydroxybutyrate [	SLC16A1	Transport, extracellular			0
r2110_neg	2	0.382733	0.4171041	-1.64322	1.643222	2	0.518179	r2110	Major Facilitator(MFS) TCDB:2.A.1.13.1	1.00 * acetate [e] + 1.00 * Formate [c] --> 1.00 * acetate [c	SLC16A1	Transport, extracellular			0
r2110_pos	2	0.382733	0.4171041	-1.64322	1.643222	2	0.518179	r2110	Major Facilitator(MFS) TCDB:2.A.1.13.1	1.00 * acetate [e] + 1.00 * Formate [c] --> 1.00 * acetate [c	SLC16A1	Transport, extracellular			0
r2111_neg	2	0.382733	0.4171041	-1.64322	1.643222	2	0.518179	r2111	Major Facilitator(MFS) TCDB:2.A.1.13.1	1.00 * propionate [c] + 1.00 * acetate [e] --> 1.00 * acetate	SLC16A1	Transport, extracellular			0
r2111_pos	2	0.382733	0.4171041	-1.64322	1.643222	2	0.518179	r2111	Major Facilitator(MFS) TCDB:2.A.1.13.1	1.00 * propionate [c] + 1.00 * acetate [e] --> 1.00 * acetate	SLC16A1	Transport, extracellular			0
r2112_neg	2	0.382733	0.4171041	-1.64322	1.643222	2	0.518179	r2112	Major Facilitator(MFS) TCDB:2.A.1.13.1	1.00 * acetate [e] + 1.00 * (S)-lactate [c] --> 1.00 * acetate [	SLC16A1	Transport, extracellular			0
r2112_pos	2	0.382733	0.4171041	-1.64322	1.643222	2	0.518179	r2112	Major Facilitator(MFS) TCDB:2.A.1.13.1	1.00 * acetate [e] + 1.00 * (S)-lactate [c] --> 1.00 * acetate [	SLC16A1	Transport, extracellular			0
r2113_neg	2	0.382733	0.4171041	-1.64322	1.643222	2	0.518179	r2113	Major Facilitator(MFS) TCDB:2.A.1.13.1	1.00 * acetate [e] + 1.00 * butyrate [c] --> 1.00 * acetate [c	SLC16A1	Transport, extracellular			0
r2113_pos	2	0.382733	0.4171041	-1.64322	1.643222	2	0.518179	r2113	Major Facilitator(MFS) TCDB:2.A.1.13.1	1.00 * acetate [e] + 1.00 * butyrate [c] --> 1.00 * acetate [c	SLC16A1	Transport, extracellular			0
r2114_neg	2	0.382733	0.4171041	-1.6311	1.631095	2	0.518179	r2114	Major Facilitator(MFS) TCDB:2.A.1.13.1	1.00 * acetate [e] + 1.00 * all-trans-retinoate [c] --> 1.00 *	SLC16A1	Transport, extracellular			0
r2114_pos	2	0.382733	0.4171041	-1.6311	1.631095	2	0.518179	r2114	Major Facilitator(MFS) TCDB:2.A.1.13.1	1.00 * acetate [e] + 1.00 * all-trans-retinoate [c] --> 1.00 *	SLC16A1	Transport, extracellular			0
r2115_neg	2	0.382733	0.4171041	-1.64322	1.643222	2	0.518179	r2115	Major Facilitator(MFS) TCDB:2.A.1.13.1	1.00 * acetate [e] + 1.00 * (R)-3-hydroxybutyrate [c] --> 1.0	SLC16A1	Transport, extracellular			0
r2115_pos	2	0.382733	0.4171041	-1.64322	1.643222	2	0.518179	r2115	Major Facilitator(MFS) TCDB:2.A.1.13.1	1.00 * acetate [e] + 1.00 * (R)-3-hydroxybutyrate [c] --> 1.0	SLC16A1	Transport, extracellular			0
r2116_neg	2	0.382733	0.4171041	-1.64322	1.643222	2	0.518179	r2116	Major Facilitator(MFS) TCDB:2.A.1.13.1	1.00 * propionate [c] + 1.00 * Formate [e] --> 1.00 * Forma	SLC16A1	Transport, extracellular			0
r2116_pos	2	0.382733	0.4171041	-1.64322	1.643222	2	0.518179	r2116	Major Facilitator(MFS) TCDB:2.A.1.13.1	1.00 * propionate [c] + 1.00 * Formate [e] --> 1.00 * Forma	SLC16A1	Transport, extracellular			0
r2117_neg	2	0.382733	0.4171041	-1.64322	1.643222	2	0.518179	r2117	Major Facilitator(MFS) TCDB:2.A.1.13.1	1.00 * Formate [e] + 1.00 * (S)-lactate [c] --> 1.00 * Format	SLC16A1	Transport, extracellular			0
r2117_pos	2	0.382733	0.4171041	-1.64322	1.643222	2	0.518179	r2117	Major Facilitator(MFS) TCDB:2.A.1.13.1	1.00 * Formate [e] + 1.00 * (S)-lactate [c] --> 1.00 * Format	SLC16A1	Transport, extracellular			0
r2118_neg	2	0.382733	0.4171041	-1.64322	1.643222	2	0.518179	r2118	Major Facilitator(MFS) TCDB:2.A.1.13.1	1.00 * butyrate [c] + 1.00 * Formate [e] --> 1.00 * butyrate	SLC16A1	Transport, extracellular			0
r2118_pos	2	0.382733	0.4171041	-1.64322	1.643222	2	0.518179	r2118	Major Facilitator(MFS) TCDB:2.A.1.13.1	1.00 * butyrate [c] + 1.00 * Formate [e] --> 1.00 * butyrate	SLC16A1	Transport, extracellular			0
r2119_neg	2	0.382733	0.4171041	-1.6311	1.631095	2	0.518179	r2119	Major Facilitator(MFS) TCDB:2.A.1.13.1	1.00 * Formate [e] + 1.00 * all-trans-retinoate [c] --> 1.00	SLC16A1	Transport, extracellular			0
r2119_pos	2	0.382733	0.4171041	-1.6311	1.631095	2	0.518179	r2119	Major Facilitator(MFS) TCDB:2.A.1.13.1	1.00 * Formate [e] + 1.00 * all-trans-retinoate [c] --> 1.00	SLC16A1	Transport, extracellular			0
r2120_neg	2	0.382733	0.4171041	-1.64322	1.643222	2	0.518179	r2120	Major Facilitator(MFS) TCDB:2.A.1.13.1	1.00 * (R)-3-hydroxybutyrate [c] + 1.00 * Formate [e] --> 1	SLC16A1	Transport, extracellular			0
r2120_pos	2	0.382733	0.4171041	-1.64322	1.643222	2	0.518179	r2120	Major Facilitator(MFS) TCDB:2.A.1.13.1	1.00 * (R)-3-hydroxybutyrate [c] + 1.00 * Formate [e] --> 1	SLC16A1	Transport, extracellular			0
r2121_neg	2	0.382733	0.4171041	-1.64322	1.643222	2	0.518179	r2121	Major Facilitator(MFS) TCDB:2.A.1.13.1	1.00 * propionate [e] + 1.00 * (S)-lactate [c] --> 1.00 * prop	SLC16A1	Transport, extracellular			0
r2121_pos	2	0.382733	0.4171041	-1.64322	1.643222	2	0.518179	r2121	Major Facilitator(MFS) TCDB:2.A.1.13.1	1.00 * propionate [e] + 1.00 * (S)-lactate [c] --> 1.00 * prop	SLC16A1	Transport, extracellular			0
r2122_neg	2	0.382733	0.4171041	-1.64322	1.643222	2	0.518179	r2122	Major Facilitator(MFS) TCDB:2.A.1.13.1	1.00 * butyrate [c] + 1.00 * propionate [e] --> 1.00 * propi	SLC16A1	Transport, extracellular			0
r2122_pos	2	0.382733	0.4171041	-1.64322	1.643222	2	0.518179	r2122	Major Facilitator(MFS) TCDB:2.A.1.13.1	1.00 * butyrate [c] + 1.00 * propionate [e] --> 1.00 * propi	SLC16A1	Transport, extracellular			0
r2123_neg	2	0.382733	0.4171041	-1.6311	1.631095	2	0.518179	r2123	Major Facilitator(MFS) TCDB:2.A.1.13.1	1.00 * propionate [e] + 1.00 * all-trans-retinoate [c] --> 1.0	SLC16A1	Transport, extracellular			0
r2123_pos	2	0.382733	0.4171041	-1.6311	1.631095	2	0.518179	r2123	Major Facilitator(MFS) TCDB:2.A.1.13.1	1.00 * propionate [e] + 1.00 * all-trans-retinoate [c] --> 1.0	SLC16A1	Transport, extracellular			0
r2124_neg	2	0.382733	0.4171041	-1.64322	1.643222	2	0.518179	r2124	Major Facilitator(MFS) TCDB:2.A.1.13.1	1.00 * (R)-3-hydroxybutyrate [c] + 1.00 * propionate [e] --	SLC16A1	Transport, extracellular			0
r2124_pos	2	0.382733	0.4171041	-1.64322	1.643222	2	0.518179	r2124	Major Facilitator(MFS) TCDB:2.A.1.13.1	1.00 * (R)-3-hydroxybutyrate [c] + 1.00 * propionate [e] --	SLC16A1	Transport, extracellular			0
r2125_neg	2	0.382733	0.4171041	-1.3398	1.339804	2	0.518179	r2125	Major Facilitator(MFS) TCDB:2.A.1.13.1	1.00 * acetoacetate [e] + 1.00 * butyrate [c] --> 1.00 * acet	SLC16A1	Transport, extracellular			0
r2125_pos	2	0.382733	0.4171041	-1.3398	1.339804	2	0.518179	r2125	Major Facilitator(MFS) TCDB:2.A.1.13.1	1.00 * acetoacetate [e] + 1.00 * butyrate [c] --> 1.00 * acet	SLC16A1	Transport, extracellular			0
r2126_neg	2	0.382733	0.4171041	-1.64322	1.643222	2	0.518179	r2126	Major Facilitator(MFS) TCDB:2.A.1.13.1	1.00 * acetoacetate [e] + 1.00 * (R)-3-hydroxybutyrate [c]	SLC16A1	Transport, extracellular			0

r2126_pos	2	0.382733	0.4171041	-1.64322	1.643222	2	0.518179	r2126	Major Facilitator(MFS) TCDB:2.A.1.13.1	1.00 * acetoacetate [e] + 1.00 * (R)-3-hydroxybutyrate [c]	SLC16A1	Transport, extracellular			0
r2127_neg	2	0.382733	0.4171041	-1.3338	1.333799	2	0.518179	r2127	Major Facilitator(MFS) TCDB:2.A.1.13.1	1.00 * butyrate [c] + 1.00 * (S)-lactate [e] --> 1.00 * butyra	SLC16A1	Transport, extracellular			0
r2127_pos	2	0.382733	0.4171041	-1.3338	1.333799	2	0.518179	r2127	Major Facilitator(MFS) TCDB:2.A.1.13.1	1.00 * butyrate [c] + 1.00 * (S)-lactate [e] --> 1.00 * butyra	SLC16A1	Transport, extracellular			0
r2128_neg	2	0.382733	0.4171041	-1.6311	1.631095	2	0.518179	r2128	Major Facilitator(MFS) TCDB:2.A.1.13.1	1.00 * (S)-lactate [e] + 1.00 * all-trans-retinoate [c] --> 1.0	SLC16A1	Transport, extracellular			0
r2128_pos	2	0.382733	0.4171041	-1.6311	1.631095	2	0.518179	r2128	Major Facilitator(MFS) TCDB:2.A.1.13.1	1.00 * (S)-lactate [e] + 1.00 * all-trans-retinoate [c] --> 1.0	SLC16A1	Transport, extracellular			0
r2129_neg	2	0.382733	0.4171041	-1.64322	1.643222	2	0.518179	r2129	Major Facilitator(MFS) TCDB:2.A.1.13.1	1.00 * (R)-3-hydroxybutyrate [c] + 1.00 * (S)-lactate [e] -->	SLC16A1	Transport, extracellular			0
r2129_pos	2	0.382733	0.4171041	-1.64322	1.643222	2	0.518179	r2129	Major Facilitator(MFS) TCDB:2.A.1.13.1	1.00 * (R)-3-hydroxybutyrate [c] + 1.00 * (S)-lactate [e] -->	SLC16A1	Transport, extracellular			0
r2130_neg	2	0.382733	0.4171041	-1.6311	1.631095	2	0.518179	r2130	Major Facilitator(MFS) TCDB:2.A.1.13.1	1.00 * butyrate [e] + 1.00 * all-trans-retinoate [c] --> 1.00	SLC16A1	Transport, extracellular			0
r2130_pos	2	0.382733	0.4171041	-1.6311	1.631095	2	0.518179	r2130	Major Facilitator(MFS) TCDB:2.A.1.13.1	1.00 * butyrate [e] + 1.00 * all-trans-retinoate [c] --> 1.00	SLC16A1	Transport, extracellular			0
r2131_neg	2	0.382733	0.4171041	-1.64322	1.643222	2	0.518179	r2131	Major Facilitator(MFS) TCDB:2.A.1.13.1	1.00 * (R)-3-hydroxybutyrate [c] + 1.00 * butyrate [e] --> 1	SLC16A1	Transport, extracellular			0
r2131_pos	2	0.382733	0.4171041	-1.64322	1.643222	2	0.518179	r2131	Major Facilitator(MFS) TCDB:2.A.1.13.1	1.00 * (R)-3-hydroxybutyrate [c] + 1.00 * butyrate [e] --> 1	SLC16A1	Transport, extracellular			0
r2132_neg	2	0.382733	0.4171041	-1.6311	1.631095	2	0.518179	r2132	Major Facilitator(MFS) TCDB:2.A.1.13.1	1.00 * (R)-3-hydroxybutyrate [c] + 1.00 * all-trans-retinoa	SLC16A1	Transport, extracellular			0
r2132_pos	2	0.382733	0.4171041	-1.6311	1.631095	2	0.518179	r2132	Major Facilitator(MFS) TCDB:2.A.1.13.1	1.00 * (R)-3-hydroxybutyrate [c] + 1.00 * all-trans-retinoa	SLC16A1	Transport, extracellular			0
r2133_neg	2	0.382733	0.4171041	-1.64322	1.643222	2	0.518179	r2133	Major Facilitator(MFS) TCDB:2.A.1.13.1	1.00 * 2-Hydroxybutyrate [c] + 1.00 * (R)-3-hydroxybutyr	SLC16A1	Transport, extracellular			0
r2133_pos	2	0.382733	0.4171041	-1.64322	1.643222	2	0.518179	r2133	Major Facilitator(MFS) TCDB:2.A.1.13.1	1.00 * 2-Hydroxybutyrate [c] + 1.00 * (R)-3-hydroxybutyr	SLC16A1	Transport, extracellular			0
r2196_neg	2	0.382733	0.4171041	-0.68538	0.685377	2	0.518179	r2196	Resistance-Nodulation-Cell Division (RND) TCDB:2.A.60.1.14	1.00 * Bicarbonate [c] + 1.00 * Reduced glutathione [c] +	SLCO1A1	Transport, extracellular			0
r2196_pos	2	0.382733	0.4171041	-0.68538	0.685377	2	0.518179	r2196	Resistance-Nodulation-Cell Division (RND) TCDB:2.A.60.1.14	1.00 * Bicarbonate [c] + 1.00 * Reduced glutathione [c] +	SLCO1A1	Transport, extracellular			0
r2525_neg	7	0.382733	0.4171041	0.291953	0.291953	1	0.518179	r2525	Major Facilitator(MFS) TCDB:2.A.1.44.1	1.00 * L-glutamine [e] --> 1.00 * L-glutamine [c]SLC43A1	SLC43A1	Transport, extracellular			0
r2526_neg	7	0.382733	0.4171041	0.291953	0.291953	1	0.518179	r2526	Major Facilitator(MFS) TCDB:2.A.1.44.1	1.00 * L-serine [e] --> 1.00 * L-serine [c]SLC43A1	SLC43A1	Transport, extracellular			0
r2532_neg	7	0.382733	0.4171041	0.291953	0.291953	1	0.518179	r2532	Major Facilitator(MFS) TCDB:2.A.1.44.1	1.00 * L-asparagine [e] --> 1.00 * L-asparagine [c]SLC43A1	SLC43A1	Transport, extracellular			0
r2535_neg	7	0.382733	0.4171041	0.487157	0.487157	1	0.518179	r2535	Major Facilitator(MFS) TCDB:2.A.1.44.1	1.00 * L-homoserine [e] --> 1.00 * L-homoserine [c]SLC43	SLC43A1	Transport, extracellular			0
2HCO3_NAt_neg	3	0.662521	0.1788004	-0.76786	0.767865	2	0.758498	2HCO3_NA	bicarbonate transport (Na/HCO3 1:2 cotransport)	2.00 * Bicarbonate [e] + 1.00 * Sodium [e] --> 2.00 * Bicar	SLC4A4; SL	Transport, extracellular			4
5MTHFt2_neg	3	0.662521	0.1788004	-1.10744	1.10744	2	0.758498	5MTHFt2	5-methyltetrahydrofolate transport via anion exchange	1.00 * Water [c] + 1.00 * 5-Methyltetrahydrofolate [e] -->	SLC19A1	Transport, extracellular			4
ALAATB0tc_pos	3	0.662521	0.1788004	-0.64698	0.646976	2	0.758498	ALAATB0tc	transport of L-Alanine into the intestinal cells by ATB0 transporter	2.00 * Sodium [e] + 1.00 * L-alanine [e] + 1.00 * Chloride [	SLC6A14	Transport, extracellular			4
ALAGLNNaEx_pos	6	0.662521	0.1788004	0.986437	0.986437	1	0.758498	ALAGLNNa	L-alanine/L-glutamine Na-dependent exchange (Ala-L in)	1.00 * Sodium [e] + 1.00 * L-alanine [e] + 1.00 * L-glutami	SLC1A5	Transport, extracellular			4
ALAt4_pos	6	0.662521	0.1788004	0.151522	0.151522	1	0.758498	ALAt4	Alanine-Sodium symporter	1.00 * Sodium [e] + 1.00 * L-alanine [e] --> 1.00 * Sodium [	ACE2; SLC3	Transport, extracellular			4
ALAtN1_pos	3	0.662521	0.1788004	-0.86973	0.869725	2	0.758498	ALAtN1	Alanine transport (Na, H coupled)	1.00 * proton [c] + 2.00 * Sodium [e] + 1.00 * L-alanine [e]	SLC38A3; S	Transport, extracellular			4
ALAyLATthc_pos	6	0.662521	0.1788004	0.515453	0.515453	1	0.758498	ALAyLATth	transport of Alanine by y+LAT1 or y+LAT2 with co-transporter of h in	1.00 * proton [e] + 1.00 * L-alanine [e] + 1.00 * L-argininiu	SLC3A2; SL	Transport, extracellular			4
ARGLYSex_pos	6	0.662521	0.1788004	0.822236	0.822236	1	0.758498	ARGLYSex	Arginine/Lysine exchanger (Arg in)	1.00 * L-lysinium(1+) [c] + 1.00 * L-argininium(1+) [e] --> 1	SLC3A2; SL	Transport, extracellular			4
ASNATB0tc_pos	3	0.662521	0.1788004	-0.88955	0.889548	2	0.758498	ASNATB0tc	transport of L-Asparagine into the intestinal cells by ATB0 transporte	2.00 * Sodium [e] + 1.00 * L-asparagine [e] + 1.00 * Chlori	SLC6A14	Transport, extracellular			4
ASNtN1_pos	3	0.662521	0.1788004	-0.88552	0.88552	2	0.758498	ASNtN1	Asparagine transport (Na, H coupled)	1.00 * proton [c] + 2.00 * Sodium [e] + 1.00 * L-asparagine	SLC38A3; S	Transport, extracellular			4
ASPDt6_pos	3	0.662521	0.1788004	-0.37691	0.376908	2	0.758498	ASPDt6	D-aspartate transport via Na, H symport and K antiport	1.00 * proton [e] + 3.00 * Sodium [e] + 1.00 * D-aspartate	SLC1A1; SL	Transport, extracellular			4
ASPt6_pos	3	0.662521	0.1788004	-0.37691	0.376908	2	0.758498	ASPt6	L-aspartate transport via Na, H symport and K antiport	1.00 * proton [e] + 3.00 * Sodium [e] + 1.00 * potassium [	SLC1A1; SL	Transport, extracellular			4
BALABETAtc_pos	6	0.662521	0.1788004	0.964986	0.964986	1	0.758498	BALABETAt	transport of beta alanine into the intestinal cells by beta transport s	2.00 * Sodium [e] + 1.00 * beta-alanine [e] + 1.00 * Chlori	SLC6A6	Transport, extracellular			4
BILIRUBt2_neg	3	0.662521	0.1788004	-0.46888	0.468877	2	0.758498	BILIRUBt2	bilirubin transport via bicarbonate countertransport	1.00 * Bicarbonate [c] + 1.00 * bilirubin(2-) [e] --> 1.00 * B	SLCO1B2	Transport, extracellular			4
CAt7r_neg	6	0.662521	0.1788004	0.579153	0.579153	1	0.758498	CAt7r	calcium / sodium antiporter (1:3), reversible	3.00 * Sodium [e] + 1.00 * calcium(2+) [c] --> 3.00 * Sodiu	SLC8A1; SL	Transport, extracellular			4
CAt7r_pos	6	0.662521	0.1788004	0.504285	0.504285	1	0.758498	CAt7r	calcium / sodium antiporter (1:3), reversible	3.00 * Sodium [e] + 1.00 * calcium(2+) [c] --> 3.00 * Sodiu	SLC8A1; SL	Transport, extracellular			4
CHOLt4_neg	3	0.662521	0.1788004	-0.44559	0.445591	2	0.758498	CHOLt4	choline, sodium cotransport	1.00 * Sodium [e] + 1.00 * Choline [e] --> 1.00 * Sodium [c	SLC5A7	Transport, extracellular			4
CHOLtu_pos	3	0.662521	0.1788004	-0.44559	0.445591	2	0.758498	CHOLtu	Choline uniport	1.00 * Choline [e] --> 1.00 * Choline [c]SLC22A2; SLC22A5	SLC22A2; S	Transport, extracellular			4
CREATt4_2_r_neg	6	0.662521	0.1788004	0.475077	0.475077	1	0.758498	CREATt4_2	Creatine transport (sodium symport) (2:1)	2.00 * Sodium [e] + 1.00 * creatine [e] --> 2.00 * Sodium [	SLC6A8	Transport, extracellular			4
CRNtHa_pos	6	0.662521	0.1788004	0.266364	0.266364	1	0.758498	CRNtHa	L-carnitine outward transport (H+ antiport)	1.00 * proton [e] + 1.00 * L-Carnitine [c] --> 1.00 * proton	SLC22A4	Transport, extracellular			4
CRNt_neg	6	0.662521	0.1788004	0.610815	0.610815	1	0.758498	CRNt	L-carnitine reversible transport	1.00 * L-Carnitine [e] --> 1.00 * L-Carnitine [c]SLC22A5	SLC22A5	Transport, extracellular			4
CRNt_pos	6	0.662521	0.1788004	0.266364	0.266364	1	0.758498	CRNt	L-carnitine reversible transport	1.00 * L-Carnitine [e] --> 1.00 * L-Carnitine [c]SLC22A5	SLC22A5	Transport, extracellular			4
CRNtuNa_pos	6	0.662521	0.1788004	0.610815	0.610815	1	0.758498	CRNtuNa	L-carnitine inward transport by Na+ symport	1.00 * Sodium [e] + 1.00 * L-Carnitine [e] --> 1.00 * Sodiu	SLC22A5	Transport, extracellular			4
CYSATB0tc_pos	6	0.662521	0.1788004	0.670927	0.670927	1	0.758498	CYSATB0tc	transport of L-Cysteine into the intestinal cells by ATB0 transporter	2.00 * Sodium [e] + 1.00 * L-cysteine [e] + 1.00 * Chloride	SLC6A14	Transport, extracellular			4
CYSSNAT5tc_neg	6	0.662521	0.1788004	0.896923	0.896923	1	0.758498	CYSSNAT5t	transport of L-Cysteine into the cell coupled with co-transport with	1.00 * proton [c] + 1.00 * Sodium [e] + 1.00 * L-cysteine [e	SLC38A5	Transport, extracellular			4
CYSSNAT5tc_pos	6	0.662521	0.1788004	0.877626	0.877626	1	0.758498	CYSSNAT5t	transport of L-Cysteine into the cell coupled with co-transport with	1.00 * proton [c] + 1.00 * Sodium [e] + 1.00 * L-cysteine [e	SLC38A5	Transport, extracellular			4
CYSTALArBATtc_pos	3	0.662521	0.1788004	-0.86028	0.86028	2	0.758498	CYSTALArB	transport of L-Cystine into the cell in exchange for L-Alanine by b0,+	1.00 * L-alanine [c] + 1.00 * L-cystine [e] --> 1.00 * L-alani	SLC3A1; SL	Transport, extracellular			4
CYSTGLUex_pos	6	0.662521	0.1788004	0.772541	0.772541	1	0.758498	CYSTGLUex	L-cystine/L-glutamate exchanger	1.00 * L-glutamate(1-) [c] + 1.00 * L-cystine [e] --> 1.00 * L	SLC3A2; SL	Transport, extracellular			4
FATP6t_neg	3	0.662521	0.1788004	-0.63376	0.633759	2	0.758498	FATP6t	fatty acid electroneutral transport	1.00 * Sodium [c] + 1.00 * adrenic acid [c] --> 1.00 * Sodiu	SLC27A2; S	Transport, extracellular			4
FATP7t_neg	3	0.662521	0.1788004	-0.63376	0.633759	2	0.758498	FATP7t	fatty acid electroneutral transport	1.00 * Sodium [c] + 1.00 * cervonic acid, C22:6 n-3 [c] -->	SLC27A2; S	Transport, extracellular			4
FATP9t_neg	3	0.662521	0.1788004	-0.63376	0.633759	2	0.758498	FATP9t	fatty acid electroneutral transport	1.00 * Sodium [c] + 1.00 * nervonate [c] --> 1.00 * Sodium	SLC27A2; S	Transport, extracellular			4
GLNALANaEx_pos	6	0.662521	0.1788004	0.859427	0.859427	1	0.758498	GLNALANa	L-alanine/L-glutamine Na-dependent exchange (Gln-L in)	1.00 * Sodium [e] + 1.00 * L-alanine [c] + 1.00 * L-glutami	SLC1A5	Transport, extracellular			4
GLNATB0tc_pos	3	0.662521	0.1788004	-0.88955	0.889548	2	0.758498	GLNATB0tc	Transport of L-Glutamine into the intestinal cells by ATB0 transporte	2.00 * Sodium [e] + 1.00 * L-glutamine [e] + 1.00 * Chlori	SLC6A14	Transport, extracellular			4
GLNSERNaEx_pos	6	0.662521	0.1788004	0.832336	0.832336	1	0.758498	GLNSERNa	L-serine/L-glutamine Na-dependent exchange (Gln-L in)	1.00 * Sodium [e] + 1.00 * L-glutamine [e] + 1.00 * L-serin	SLC1A5	Transport, extracellular			4
GLNTHRNaEx_pos	6	0.662521	0.1788004	0.832336	0.832336	1	0.758498	GLNTHRNa	L-threonine/L-glutamine Na-dependent exchange (Gln-L in)	1.00 * Sodium [e] + 1.00 * L-glutamine [e] + 1.00 * L-threo	SLC1A5	Transport, extracellular			4
GLNtN1_pos	3	0.662521	0.1788004	-0.86973	0.869725	2	0.758498	GLNtN1	Glutamine transport (Na, H coupled)	1.00 * proton [c] + 2.00 * Sodium [e] + 1.00 * L-glutamine	SLC38A3; S	Transport, extracellular			4
GLYSNAT5tc_neg	6	0.662521	0.1788004	0.895213	0.895213	1	0.758498	GLYSNAT5t	transport of Glycine into the cell coupled with co-transport with So	1.00 * proton [c] + 1.00 * Sodium [e] + 1.00 * Glycine [e] -	SLC38A5	Transport, extracellular			4
GLYt4_pos	6	0.662521	0.1788004	1.244595	1.244595	1	0.758498	GLYt4	glycine transport via sodium symport	1.00 * Sodium [e] + 1.00 * Glycine [e] --> 1.00 * Sodium [c]	ACE2; SLC3	Transport, extracellular			4
HCO3_NAt_neg	6	0.662521	0.1788004	0.292216	0.292216	1	0.758498	HCO3_NAt	bicarbonate transport (Na/HCO3 cotransport)	1.00 * Bicarbonate [e] + 1.00 * Sodium [e] --> 1.00 * Bicar	SLC4A7	Transport, extracellular			4
HISt4_pos	3	0.662521	0.1788004	-0.53967	0.539673	2	0.758498	HISt4	L-histidine transport in via sodium symport	1.00 * Sodium [e] + 1.00 * L-histidine [e] --> 1.00 * Sodium	SLC38A2; S	Transport, extracellular			4
ILELAT1tc_pos	6	0.662521	0.1788004	0.889707	0.889707	1	0.758498	ILELAT1tc	transport of L-Isoleucine by LAT1 in association with 4F2hc, across t	1.00 * L-isoleucine [e] + 1.00 * L-leucine [c] --> 1.00 * L-leu	SLC3A2; SL	Transport, extracellular			4
ILEPHELAT2tc_pos	6	0.662521	0.1788004	0.832843	0.832843	1	0.758498	ILEPHELAT2	transport of L-Isoleucine into the cell and efflux of L-Phenylalanine o	1.00 * L-isoleucine [e] + 1.00 * L-phenylalanine [c] --> 1.00	SLC3A2; SL	Transport, extracellular			4
KCCt_neg	6	0.662521	0.1788004	0.794038	0.794038	1	0.758498	KCCt	K+-Cl- cotransport	1.00 * potassium [e] + 1.00 * Chloride [e] --> 1.00 * potass	SLC12A4; S	Transport, extracellular			4
KCCt_pos	6	0.662521	0.1788004	0.570174	0.570174	1	0.758498	KCCt	K+-Cl- cotransport	1.00 * potassium [e] + 1.00 * Chloride [e] --> 1.00 * potass	SLC12A4; S	Transport, extracellular			4
LEUPHELAT2tc_neg	6	0.662521	0.1788004	0.832843	0.832843	1	0.758498	LEUPHELAT	Transport of L-Leucine into the cell and efflux of L-Phenylalanine out	1.00 * L-leucine [e] + 1.00 * L-phenylalanine [c] --> 1.00 *	SLC3A2; SL	Transport, extracellular			4
LEUPHELAT2tc_pos	6	0.662521	0.1788004	0.832843	0.832843	1	0.758498	LEUPHELAT	Transport of L-Leucine into the cell and efflux of L-Phenylalanine out	1.00 * L-leucine [e] + 1.00 * L-phenylalanine [c] --> 1.00 *	SLC3A2; SL	Transport, extracellular			4
METATB0tc_pos	6	0.662521	0.1788004	0.670927	0.670927	1	0.758498	METATB0tc	transport of L-Methionine into the intestinal cells by ATB0 transport	2.00 * Sodium [e] + 1.00 * Chloride [e] + 1.00 * L-methion	SLC6A14	Transport, extracellular			4
METtec_neg	6	0.662521	0.1788004	0.327553	0.327553	1	0.758498	METtec	L-methionine transport via diffusion (extracellular to cytosol)	1.00 * L-methionine [e] --> 1.00 * L-methionine [c]SLC43A	SLC43A1; S	Transport, extracellular			4
NAIt_pos	6	0.662521	0.1788004	0.446851	0.446851	1	0.758498	NAIt	Na+ / iodide cotransport	2.00 * Sodium [e] + 1.00 * iodide [e] --> 2.00 * Sodium [c]	SLC5A5	Transport, extracellular			4
NAt3_1_neg	6	0.662521	0.1788004	0.369768	0.369768	1	0.758498	NAt3_1	sodium proton antiporter (H:NA is 1:1)	1.00 * proton [e] + 1.00 * Sodium [c] --> 1.00 * proton [c]	SLC9A1; SL	Transport, extracellular			4
NAt3_1_pos	6	0.662521	0.1788004	0.369768	0.369768	1	0.758498	NAt3_1	sodium proton antiporter (H:NA is 1:1)	1.00 * proton [e] + 1.00 * Sodium [c] --> 1.00 * proton [c]	SLC9A1; SL	Transport, extracellular			4
NAt5_pos	6	0.662521	0.1788004	0.269849	0.269849	1	0.758498	NAt5	sodium/ammonium proton antiporter	1.00 * Sodium [c] + 1.00 * Ammonium [e] --> 1.00 * Amm	SLC9A1; SL	Transport, extracellular			4
NCCt_pos	6	0.662521	0.1788004	-0.76629	0.766287	2	0.758498	NCCt	Na+-Cl- cotransport	1.00 * Sodium [e] + 1.00 * Chloride [e] --> 1.00 * Sodium [	SLC12A3	Transport, extracellular			4
NH4t3r_neg	3	0.662521	0.1788004	-0.67202	0.672018	2	0.758498	NH4t3r	ammonia transport via proton antiport	1.00 * Ammonium [c] + 1.00 * proton [e] --> 1.00 * proto	RHAG; RHB	Transport, extracellular			4
NH4t3r_pos	3	0.662521	0.1788004	-0.67202	0.672018	2	0.758498	NH4t3r	ammonia transport via proton antiport	1.00 * Ammonium [c] + 1.00 * proton [e] --> 1.00 * proto	RHAG; RHB	Transport, extracellular			4
NKCC2t_neg	6	0.662521	0.1788004	0.681244	0.681244	1	0.758498	NKCC2t	Na+-K+-Cl- cotransport (NH4+)	1.00 * Sodium [e] + 1.00 * Ammonium [e] + 2.00 * Chlori	SLC12A1; S	Transport, extracellular			4
NKCC2t_pos	6	0.662521	0.1788004	0.774092	0.774092	1	0.758498	NKCC2t	Na+-K+-Cl- cotransport (NH4+)	1.00 * Sodium [e] + 1.00 * Ammonium [e] + 2.00 * Chlori	SLC12A1; S	Transport, extracellular			4
NKCCt_neg	6	0.662521	0.1788004	0.800874	0.800874	1	0.758498	NKCCt	Na+-K+-Cl- cotransport	1.00 * Sodium [e] + 1.00 * potassium [e] + 2.00 * Chloride	SLC12A1; S	Transport, extracellular			4
NKCCt_pos	6	0.662521	0.1788004	0.855608	0.855608	1	0.758498	NKCCt	Na+-K+-Cl- cotransport	1.00 * Sodium [e] + 1.00 * potassium [e] + 2.00 * Chloride	SLC12A1; S	Transport, extracellular			4
PHEATB0tc_pos	6	0.662521	0.1788004	0.875723	0.875723	1	0.758498	PHEATB0tc	transport of L-Phenylalanine into the intestinal cells by ATB0 transp	2.00 * Sodium [e] + 1.00 * Chloride [e] + 1.00 * L-phenylal	SLC6A14	Transport, extracellular			4
PROIMINOtc_pos	6	0.662521	0.1788004	-0.29938	0.299377	2	0.758498	PROIMINO	transport of L-Proline by the apical IMINO amino acid transporters i	2.00 * Sodium [e] + 1.00 * Chloride [e] + 1.00 * L-proline [	SLC6A20B;	Transport, extracellular			4
PROSTGE2t2_neg	3	0.662521	0.1788004	-0.61964	0.619645	2	0.758498	PROSTGE2t	prostaglandin uniport	1.00 * prostaglandin E2(1-) [e] --> 1.00 * prostaglandin E2	SLC22A1; S	Transport, extracellular			4

PROSTGE2t2_pos	3	0.662521	0.1788004	-0.67693	0.676932	2	0.758498	PROSTGE2t	prostaglandin uniport	1.00 * prostaglandin E2(1-) [e] --> 1.00 * prostaglandin E2	SLC22A1; S	Transport, extracellular				4
PROSTGE2t_neg	3	0.662521	0.1788004	-0.67693	0.676932	2	0.758498	PROSTGE2t	prostaglandin transport via bicarbonate countertransport	1.00 * Bicarbonate [c] + 1.00 * prostaglandin E2(1-) [e] -->	SLCO1A1; S	Transport, extracellular				4
PROSTGE2t_pos	3	0.662521	0.1788004	-0.61964	0.619645	2	0.758498	PROSTGE2t	prostaglandin transport via bicarbonate countertransport	1.00 * Bicarbonate [c] + 1.00 * prostaglandin E2(1-) [e] -->	SLCO1A1; S	Transport, extracellular				4
PROt4_2_r_neg	3	0.662521	0.1788004	-1.4783	1.478299	2	0.758498	PROt4_2_r	Proline transport (sodium symport) (2:1)	2.00 * Sodium [e] + 1.00 * L-proline [e] --> 2.00 * Sodium [	SLC6A7	Transport, extracellular				4
SERGLNNaEx_pos	6	0.662521	0.1788004	0.853722	0.853722	1	0.758498	SERGLNNaE	L-serine/L-glutamine Na-dependent exchange (Ser-L in)	1.00 * Sodium [e] + 1.00 * L-glutamine [c] + 1.00 * L-serine	SLC1A5	Transport, extracellular				4
SERtN1_neg	6	0.662521	0.1788004	0.921057	0.921057	1	0.758498	SERtN1	Serine transport (Na, H coupled)	1.00 * proton [c] + 2.00 * Sodium [e] + 1.00 * L-serine [e] -	SLC38A5	Transport, extracellular				4
SERtN1_pos	6	0.662521	0.1788004	0.928035	0.928035	1	0.758498	SERtN1	Serine transport (Na, H coupled)	1.00 * proton [c] + 2.00 * Sodium [e] + 1.00 * L-serine [e] -	SLC38A5	Transport, extracellular				4
SO4t4_2_pos	3	0.662521	0.1788004	-0.03739	0.037393	2	0.758498	SO4t4_2	sulfate transport via sodium symport	2.00 * Sodium [e] + 1.00 * sulfate [e] --> 2.00 * Sodium [c]	SLC13A4	Transport, extracellular				4
SRTNt6_2_r_pos	6	0.662521	0.1788004	0.003858	0.003858	1	0.758498	SRTNt6_2_	Seratonin reversible transport in via sodium symport/potassium ant	2.00 * Sodium [e] + 2.00 * potassium [c] + 1.00 * serotoni	SLC6A4	Transport, extracellular				4
TAUPAT1c_pos	6	0.662521	0.1788004	1.013934	1.013934	1	0.758498	TAUPAT1c	transport of taurine into the intestinal cells by PAT1	1.00 * proton [e] + 1.00 * Taurine [e] --> 1.00 * proton [c] -	SLC36A1	Transport, extracellular				4
TAURt4_2_r_pos	6	0.662521	0.1788004	0.798705	0.798705	1	0.758498	TAURt4_2_	taurine transport (sodium symport) (2:1)	2.00 * Sodium [e] + 1.00 * Taurine [e] --> 2.00 * Sodium [c	SLC6A6	Transport, extracellular				4
THRGLNNaEx_pos	6	0.662521	0.1788004	0.853722	0.853722	1	0.758498	THRGLNNa	L-threonine/L-glutamine Na-dependent exchange (Thr-L in)	1.00 * Sodium [e] + 1.00 * L-glutamine [c] + 1.00 * L-threo	SLC1A5	Transport, extracellular				4
THRPHELAT2tc_pos	6	0.662521	0.1788004	0.791739	0.791739	1	0.758498	THRPHELAT	transport of L-Threonine into the cell and efflux of L-Phenylalanine o	1.00 * L-threonine [e] + 1.00 * L-phenylalanine [c] --> 1.00	SLC3A2; SL	Transport, extracellular				4
TYRPHELAT2tc_pos	3	0.662521	0.1788004	-0.68569	0.68569	2	0.758498	TYRPHELAT	transport of L-Tyrosine into the cell and efflux of L-Phenylalanine ou	1.00 * L-tyrosine [e] + 1.00 * L-phenylalanine [c] --> 1.00 *	SLC3A2; SL	Transport, extracellular				4
VALLAT1tc_pos	6	0.662521	0.1788004	0.889707	0.889707	1	0.758498	VALLAT1tc	transport of L-Valine by LAT1 in association with 4F2hc, across the a	1.00 * L-valine [e] + 1.00 * L-leucine [c] --> 1.00 * L-leucine	SLC3A2; SL	Transport, extracellular				4
VALPHELAT2tc_pos	6	0.662521	0.1788004	0.832843	0.832843	1	0.758498	VALPHELAT	transport of L-Valine into the cell and efflux of L-Phenylalanine out o	1.00 * L-valine [e] + 1.00 * L-phenylalanine [c] --> 1.00 * L-	SLC3A2; SL	Transport, extracellular				4
r0942_neg	6	0.662521	0.1788004	0.475077	0.475077	1	0.758498	r0942	Neurotransmitter:Sodium Symporter (NSS) TCDB:2.A.22.3.4	1.00 * creatine [c] --> 1.00 * creatine [e]SLC6A8	SLC6A8	Transport, extracellular				0
r0961_pos	3	0.662521	0.1788004	-0.46888	0.468877	2	0.758498	r0961	Resistance-Nodulation-Cell Division (RND) TCDB:2.A.65.1.1	1.00 * Sodium [e] + 1.00 * bilirubin(2-) [e] --> 1.00 * Sodiu	CP	Transport, extracellular				0
r0974_pos	3	0.662521	0.1788004	-0.57167	0.571673	2	0.758498	r0974	Active transport	1.00 * Sodium [e] + 1.00 * (R)-Pantothenate [e] --> 1.00 * S	1.00 * Sodi	Transport, extracellular				0
r1045_pos	3	0.662521	0.1788004	-0.38014	0.380138	2	0.758498	r1045	Concentrative Nucleoside Transporter (CNT) TCDB:2.A.41.2.4	1.00 * Sodium [e] + 1.00 * Deoxyguanosine [e] --> 1.00 * S	SLC28A2; S	Transport, extracellular				0
r1143_pos	3	0.662521	0.1788004	-0.57167	0.571673	2	0.758498	r1143	Major Facilitator(MFS) TCDB:2.A.18.6.3	1.00 * Sodium [e] + 1.00 * L-aspartate(1-) [e] --> 1.00 * Soc	SLC38A3	Transport, extracellular				0
r1495_pos	3	0.662521	0.1788004	-0.33919	0.339191	2	0.758498	r1495	Glycoside-Pentoside-Hexuronide (GPH):Cation Symporter TCDB:2.A	2.00 * Sodium [e] + 1.00 * chenodeoxycholate [e] --> 2.00	SLC10A1	Transport, extracellular				0
r1497_pos	3	0.662521	0.1788004	-0.33919	0.339191	2	0.758498	r1497	Glycoside-Pentoside-Hexuronide (GPH):Cation Symporter TCDB:2.A	2.00 * Sodium [e] + 1.00 * taurochenodeoxycholate [e] -->	SLC10A1	Transport, extracellular				0
r1499_pos	3	0.662521	0.1788004	-0.33919	0.339191	2	0.758498	r1499	Glycoside-Pentoside-Hexuronide (GPH):Cation Symporter TCDB:2.A	2.00 * Sodium [e] + 1.00 * taurolithocholate [e] --> 2.00 *	SLC10A1	Transport, extracellular				0
r1500_pos	3	0.662521	0.1788004	-0.33919	0.339191	2	0.758498	r1500	Glycoside-Pentoside-Hexuronide (GPH):Cation Symporter TCDB:2.A	2.00 * Sodium [e] + 1.00 * glycolithocholate [e] --> 2.00 *	SLC10A1	Transport, extracellular				0
r1501_pos	3	0.662521	0.1788004	-0.33919	0.339191	2	0.758498	r1501	Glycoside-Pentoside-Hexuronide (GPH):Cation Symporter TCDB:2.A	2.00 * Sodium [e] + 1.00 * tauroursodeoxycholate [e] --> 2	SLC10A1	Transport, extracellular				0
r1502_pos	3	0.662521	0.1788004	-0.33919	0.339191	2	0.758498	r1502	Glycoside-Pentoside-Hexuronide (GPH):Cation Symporter TCDB:2.A	2.00 * Sodium [e] + 1.00 * glycoursodeoxycholate [e] --> 2	SLC10A1	Transport, extracellular				0
r1503_pos	3	0.662521	0.1788004	-0.33919	0.339191	2	0.758498	r1503	Glycoside-Pentoside-Hexuronide (GPH):Cation Symporter TCDB:2.A	2.00 * Sodium [e] + 1.00 * sulfochenodeoxycholate [e] -->	SLC10A1	Transport, extracellular				0
r1538_pos	3	0.662521	0.1788004	-0.39301	0.393012	2	0.758498	r1538	Anion Exchanger (AE) TCDB:2.A.31.2.8	1.00 * Bicarbonate [e] + 2.00 * Sodium [e] --> 1.00 * Bicar	SLC4A5	Transport, extracellular				0
r1632_pos	6	0.662521	0.1788004	0.509084	0.509084	1	0.758498	r1632	Amino Acid-Polyamine-Organocation (APC) TCDB:2.A.3.8.1	1.00 * L-threonine [c] + 1.00 * L-tyrosine [e] --> 1.00 * L-th	SLC7A5	Transport, extracellular				0
r1995_pos	3	0.662521	0.1788004	-0.83331	0.833313	2	0.758498	r1995	Amino Acid-Polyamine-Organocation (APC) TCDB:2.A.3.8.15	1.00 * L-serine [c] + 1.00 * L-lysinium(1+) [e] --> 1.00 * L-se	SLC7A9	Transport, extracellular				0
r2102_neg	6	0.662521	0.1788004	0.636543	0.636543	1	0.758498	r2102	Major Facilitator(MFS) TCDB:2.A.1.13.1	1.00 * 2-Oxoglutarate [e] + 1.00 * Formate [c] --> 1.00 * 2-	SLC16A1	Transport, extracellular				0
r2157_neg	3	0.662521	0.1788004	-0.30634	0.306337	2	0.758498	r2157	Resistance-Nodulation-Cell Division (RND) TCDB:2.A.60.1.14	1.00 * Bicarbonate [c] + 1.00 * chenodeoxycholate [e] + 1	SLCO1A1	Transport, extracellular				0
r2158_neg	3	0.662521	0.1788004	-0.30634	0.306337	2	0.758498	r2158	Resistance-Nodulation-Cell Division (RND) TCDB:2.A.60.1.14	1.00 * Bicarbonate [c] + 1.00 * chenodeoxycholate [e] + 1	SLCO1A1	Transport, extracellular				0
r2159_neg	3	0.662521	0.1788004	-0.30634	0.306337	2	0.758498	r2159	Resistance-Nodulation-Cell Division (RND) TCDB:2.A.60.1.14	1.00 * Bicarbonate [c] + 1.00 * chenodeoxycholate [e] + 1	SLCO1A1	Transport, extracellular				0
r2163_neg	3	0.662521	0.1788004	-0.30634	0.306337	2	0.758498	r2163	Resistance-Nodulation-Cell Division (RND) TCDB:2.A.60.1.14	1.00 * Bicarbonate [c] + 1.00 * taurochenodeoxycholate [	SLCO1A1	Transport, extracellular				0
r2164_neg	3	0.662521	0.1788004	-0.30634	0.306337	2	0.758498	r2164	Resistance-Nodulation-Cell Division (RND) TCDB:2.A.60.1.14	1.00 * Bicarbonate [c] + 1.00 * taurochenodeoxycholate [	SLCO1A1	Transport, extracellular				0
r2165_neg	3	0.662521	0.1788004	-0.30634	0.306337	2	0.758498	r2165	Resistance-Nodulation-Cell Division (RND) TCDB:2.A.60.1.14	1.00 * Bicarbonate [c] + 1.00 * taurochenodeoxycholate [	SLCO1A1	Transport, extracellular				0
r2169_neg	3	0.662521	0.1788004	-0.30634	0.306337	2	0.758498	r2169	Resistance-Nodulation-Cell Division (RND) TCDB:2.A.60.1.14	1.00 * Bicarbonate [c] + 1.00 * taurolithocholate [e] + 1.0	SLCO1A1	Transport, extracellular				0
r2170_neg	3	0.662521	0.1788004	-0.30634	0.306337	2	0.758498	r2170	Resistance-Nodulation-Cell Division (RND) TCDB:2.A.60.1.14	1.00 * Bicarbonate [c] + 1.00 * taurolithocholate [e] + 1.0	SLCO1A1	Transport, extracellular				0
r2171_neg	3	0.662521	0.1788004	-0.30634	0.306337	2	0.758498	r2171	Resistance-Nodulation-Cell Division (RND) TCDB:2.A.60.1.14	1.00 * Bicarbonate [c] + 1.00 * taurolithocholate [e] + 1.0	SLCO1A1	Transport, extracellular				0
r2172_neg	3	0.662521	0.1788004	-0.30634	0.306337	2	0.758498	r2172	Resistance-Nodulation-Cell Division (RND) TCDB:2.A.60.1.14	1.00 * Bicarbonate [c] + 1.00 * glycolithocholate [e] + 1.0	SLCO1A1	Transport, extracellular				0
r2173_neg	3	0.662521	0.1788004	-0.30634	0.306337	2	0.758498	r2173	Resistance-Nodulation-Cell Division (RND) TCDB:2.A.60.1.14	1.00 * Bicarbonate [c] + 1.00 * glycolithocholate [e] + 1.0	SLCO1A1	Transport, extracellular				0
r2174_neg	3	0.662521	0.1788004	-0.30634	0.306337	2	0.758498	r2174	Resistance-Nodulation-Cell Division (RND) TCDB:2.A.60.1.14	1.00 * Bicarbonate [c] + 1.00 * glycolithocholate [e] + 1.0	SLCO1A1	Transport, extracellular				0
r2178_neg	3	0.662521	0.1788004	-0.30634	0.306337	2	0.758498	r2178	Resistance-Nodulation-Cell Division (RND) TCDB:2.A.60.1.14	1.00 * Bicarbonate [c] + 1.00 * tauroursodeoxycholate [e	SLCO1A1	Transport, extracellular				0
r2179_neg	3	0.662521	0.1788004	-0.30634	0.306337	2	0.758498	r2179	Resistance-Nodulation-Cell Division (RND) TCDB:2.A.60.1.14	1.00 * Bicarbonate [c] + 1.00 * tauroursodeoxycholate [e	SLCO1A1	Transport, extracellular				0
r2180_neg	3	0.662521	0.1788004	-0.30634	0.306337	2	0.758498	r2180	Resistance-Nodulation-Cell Division (RND) TCDB:2.A.60.1.14	1.00 * Bicarbonate [c] + 1.00 * tauroursodeoxycholate [e	SLCO1A1	Transport, extracellular				0
r2181_neg	3	0.662521	0.1788004	-0.30634	0.306337	2	0.758498	r2181	Resistance-Nodulation-Cell Division (RND) TCDB:2.A.60.1.14	1.00 * Bicarbonate [c] + 1.00 * glycoursodeoxycholate [e	SLCO1A1	Transport, extracellular				0
r2182_neg	3	0.662521	0.1788004	-0.30634	0.306337	2	0.758498	r2182	Resistance-Nodulation-Cell Division (RND) TCDB:2.A.60.1.14	1.00 * Bicarbonate [c] + 1.00 * glycoursodeoxycholate [e	SLCO1A1	Transport, extracellular				0
r2183_neg	3	0.662521	0.1788004	-0.30634	0.306337	2	0.758498	r2183	Resistance-Nodulation-Cell Division (RND) TCDB:2.A.60.1.14	1.00 * Bicarbonate [c] + 1.00 * glycoursodeoxycholate [e	SLCO1A1	Transport, extracellular				0
r2190_neg	3	0.662521	0.1788004	-0.30634	0.306337	2	0.758498	r2190	Resistance-Nodulation-Cell Division (RND) TCDB:2.A.60.1.14	1.00 * Bicarbonate [c] + 1.00 * sulfochenodeoxycholate [	SLCO1A1	Transport, extracellular				0
r2191_neg	3	0.662521	0.1788004	-0.30634	0.306337	2	0.758498	r2191	Resistance-Nodulation-Cell Division (RND) TCDB:2.A.60.1.14	1.00 * Bicarbonate [c] + 1.00 * sulfochenodeoxycholate [	SLCO1A1	Transport, extracellular				0
r2192_neg	3	0.662521	0.1788004	-0.30634	0.306337	2	0.758498	r2192	Resistance-Nodulation-Cell Division (RND) TCDB:2.A.60.1.14	1.00 * Bicarbonate [c] + 1.00 * sulfochenodeoxycholate [	SLCO1A1	Transport, extracellular				0
r2197_neg	3	0.662521	0.1788004	-0.49385	0.493845	2	0.758498	r2197	Resistance-Nodulation-Cell Division (RND) TCDB:2.A.60.1.14	1.00 * Bicarbonate [c] + 1.00 * Reduced glutathione [c] +	SLCO1A1	Transport, extracellular				0
r2197_pos	3	0.662521	0.1788004	-0.49385	0.493845	2	0.758498	r2197	Resistance-Nodulation-Cell Division (RND) TCDB:2.A.60.1.14	1.00 * Bicarbonate [c] + 1.00 * Reduced glutathione [c] +	SLCO1A1	Transport, extracellular				0
r2212_neg	3	0.662521	0.1788004	-0.49385	0.493845	2	0.758498	r2212	Resistance-Nodulation-Cell Division (RND) TCDB:2.A.60.1.14	1.00 * Bicarbonate [c] + 1.00 * glutathionyl-leuc4 [c] + 1.0	SLCO1A1	Transport, extracellular				0
r2212_pos	3	0.662521	0.1788004	-0.49385	0.493845	2	0.758498	r2212	Resistance-Nodulation-Cell Division (RND) TCDB:2.A.60.1.14	1.00 * Bicarbonate [c] + 1.00 * glutathionyl-leuc4 [c] + 1.0	SLCO1A1	Transport, extracellular				0
r2213_neg	3	0.662521	0.1788004	-0.49385	0.493845	2	0.758498	r2213	Resistance-Nodulation-Cell Division (RND) TCDB:2.A.60.1.14	1.00 * Bicarbonate [c] + 1.00 * S-glutathionyl-2-4-dinitro	SLCO1A1	Transport, extracellular				0
r2213_pos	3	0.662521	0.1788004	-0.49385	0.493845	2	0.758498	r2213	Resistance-Nodulation-Cell Division (RND) TCDB:2.A.60.1.14	1.00 * Bicarbonate [c] + 1.00 * S-glutathionyl-2-4-dinitro	SLCO1A1	Transport, extracellular				0
r2214_neg	3	0.662521	0.1788004	-0.49385	0.493845	2	0.758498	r2214	Resistance-Nodulation-Cell Division (RND) TCDB:2.A.60.1.14	1.00 * Bicarbonate [c] + 1.00 * S-glutathionyl-ethacrynic-	SLCO1A1	Transport, extracellular				0
r2214_pos	3	0.662521	0.1788004	-0.49385	0.493845	2	0.758498	r2214	Resistance-Nodulation-Cell Division (RND) TCDB:2.A.60.1.14	1.00 * Bicarbonate [c] + 1.00 * S-glutathionyl-ethacrynic-	SLCO1A1	Transport, extracellular				0
r2309_neg	3	0.662521	0.1788004	-0.48327	0.483274	2	0.758498	r2309	Resistance-Nodulation-Cell Division (RND) TCDB:2.A.60.1.14	1.00 * Bicarbonate [c] + 1.00 * Reduced glutathione [c] +	SLCO1A1	Transport, extracellular				0
r2309_pos	3	0.662521	0.1788004	-0.48327	0.483274	2	0.758498	r2309	Resistance-Nodulation-Cell Division (RND) TCDB:2.A.60.1.14	1.00 * Bicarbonate [c] + 1.00 * Reduced glutathione [c] +	SLCO1A1	Transport, extracellular				0
r2310_neg	3	0.662521	0.1788004	-0.49384	0.493837	2	0.758498	r2310	Resistance-Nodulation-Cell Division (RND) TCDB:2.A.60.1.14	1.00 * Bicarbonate [c] + 1.00 * Reduced glutathione [c] +	SLCO1A1	Transport, extracellular				0
r2310_pos	3	0.662521	0.1788004	-0.49384	0.493837	2	0.758498	r2310	Resistance-Nodulation-Cell Division (RND) TCDB:2.A.60.1.14	1.00 * Bicarbonate [c] + 1.00 * Reduced glutathione [c] +	SLCO1A1	Transport, extracellular				0
r2311_neg	3	0.662521	0.1788004	-0.49383	0.493829	2	0.758498	r2311	Resistance-Nodulation-Cell Division (RND) TCDB:2.A.60.1.14	1.00 * Bicarbonate [c] + 1.00 * acetylcholine [e] + 1.00 * R	SLCO1A1	Transport, extracellular				0
r2311_pos	3	0.662521	0.1788004	-0.49383	0.493829	2	0.758498	r2311	Resistance-Nodulation-Cell Division (RND) TCDB:2.A.60.1.14	1.00 * Bicarbonate [c] + 1.00 * acetylcholine [e] + 1.00 * R	SLCO1A1	Transport, extracellular				0
r2352_neg	3	0.662521	0.1788004	-0.49385	0.493845	2	0.758498	r2352	Organic anion transporter 5 Utilized transport	1.00 * reverse-triiodthyronine [e] --> 1.00 * reverse-triiod	1.00 * reve	Transport, extracellular				0
r2352_pos	3	0.662521	0.1788004	-0.49385	0.493845	2	0.758498	r2352	Organic anion transporter 5 Utilized transport	1.00 * reverse-triiodthyronine [e] --> 1.00 * reverse-triiod	1.00 * reve	Transport, extracellular				0
r2449_pos	3	0.662521	0.1788004	-0.38014	0.380138	2	0.758498	r2449	Concentrative Nucleoside Transporter (CNT) TCDB:2.A.41.2.4	1.00 * Sodium [e] + 1.00 * Deoxyinosine [e] --> 1.00 * Sodi	SLC28A2; S	Transport, extracellular				0
r2471_pos	6	0.662521	0.1788004	0.501961	0.501961	1	0.758498	r2471	Major Facilitator(MFS) TCDB:2.A.18.8.1	1.00 * proton [e] + 1.00 * L-serine [e] --> 1.00 * proton [c]	SLC36A1	Transport, extracellular				0
34DHPHELAT1tc_po	4	1	0	-0.6815	0.681504	2	1	34DHPHEL	transport of 3,4-Dihydroxy-L-phenylalanine by LAT1 in association w	1.00 * L-dopa [e] + 1.00 * L-leucine [c] --> 1.00 * L-dopa [c]	SLC3A2; SL	Transport, extracellular				4
34DHPHEt_neg	4	1	0	-0.6815	0.681504	2	1	34DHPHEt	3,4-Dihydroxy-L-phenylalanine transport	1.00 * L-dopa [e] --> 1.00 * L-dopa [c]SLC16A10	SLC16A10	Transport, extracellular				4
4OHPROIMINOtc_po	5	1	0	-0.34879	0.348793	2	1	4OHPROIM	transport of L-OH-Proline by the apical IMINO amino acid transporte	2.00 * Sodium [e] + 1.00 * Chloride [e] + 1.00 * trans-4-hy	SLC6A20B;	Transport, extracellular				4
AKGt4_3_neg	5	1	0	0.142464	0.142464	1	1	AKGt4_3	AKG transport via sodium symport	3.00 * Sodium [e] + 1.00 * 2-Oxoglutarate [e] --> 1.00 * 2-(	SLC13A3	Transport, extracellular				4
AKGt4_3_pos	5	1	0	0.258846	0.258846	1	1	AKGt4_3	AKG transport via sodium symport	3.00 * Sodium [e] + 1.00 * 2-Oxoglutarate [e] --> 1.00 * 2-(	SLC13A3	Transport, extracellular				4
ALAtN1_neg	4	1	0	-0.64685	0.646852	2	1	ALAtN1	Alanine transport (Na, H coupled)	1.00 * proton [c] + 2.00 * Sodium [e] + 1.00 * L-alanine [e]	SLC38A3; S	Transport, extracellular				4
ARGATB0tc_pos	5	1	0	0.163705	0.163705	1	1	ARGATB0tc	transport of L-Arginine into the intestinal cells by ATB0 transporter	2.00 * Sodium [e] + 1.00 * L-argininium(1+) [e] + 1.00 * Ch	SLC6A14	Transport, extracellular				4
ASCBt4_neg	4	1	0	-0.52556	0.525562	2	1	ASCBt4	L-ascorbate transport via proton symport	1.00 * Sodium [e] + 1.00 * L-ascorbate [e] --> 1.00 * Sodiur	SLC23A1; S	Transport, extracellular				4
ASNPHELAT2tc_pos	4	1	0	0.136487	0.136487	1	1	ASNPHELAT	transport of L-Asparagine into the cell and efflux of L-Phenylalanine	1.00 * L-asparagine [e] + 1.00 * L-phenylalanine [c] --> 1.0	SLC3A2; SL	Transport, extracellular				4
BALAPAT1tc_pos	5	1	0	0.288107	0.288107	1	1	BALAPAT1t	transport of beta alanine by PAT1 in renal and intestinal cells	1.00 * proton [e] + 1.00 * beta-alanine [e] --> 1.00 * proto	SLC36A1	Transport, extracellular				4
BILIRUBt2_pos	4	1	0	-0.19569	0.195687	2	1	BILIRUBt2	bilirubin transport via bicarbonate countertransport	1.00 * Bicarbonate [c] + 1.00 * bilirubin(2-) [e] --> 1.00 * B	SLCO1B2	Transport, extracellular				4
CHOLATEt_pos	4	1	0	-0.19467	0.194669	2	1	CHOLATEt	cholate transport via bicarbonate countertransport	1.00 * Bicarbonate [c] + 1.00 * Cholate [e] --> 1.00 * Bicar	SLCO1A1; S	Transport, extracellular				4

CHOLt4_pos	5	1	0	0.32394	0.32394	1	1	CHOLt4	choline, sodium cotransport	1.00 * Sodium [e] + 1.00 * Choline [e] --> 1.00 * Sodium [c	SLC5A7	Transport, extracellular			4
CHOLtu_neg	5	1	0	0.32394	0.32394	1	1	CHOLtu	Choline uniport	1.00 * Choline [e] --> 1.00 * Choline [c]SLC22A2; SLC22A5	SLC22A2; S	Transport, extracellular			4
CYSPHELAT2tc_pos	5	1	0	-0.20179	0.201789	2	1	CYSPHELAT	transport of L-Cysteine into the cell and efflux of L-Phenylalanine ou	1.00 * L-cysteine [e] + 1.00 * L-phenylalanine [c] --> 1.00 *	SLC3A2; SL	Transport, extracellular			4
DOPAt4_2_r_neg	4	1	0	0.192035	0.192035	1	1	DOPAt4_2_	Dopamine reversible transport in via sodium symport (1:2)	2.00 * Sodium [e] + 1.00 * dopaminium(1+) [e] --> 2.00 * S	SLC6A2; SL	Transport, extracellular			4
DOPAt4_2_r_pos	4	1	0	0.233182	0.233182	1	1	DOPAt4_2_	Dopamine reversible transport in via sodium symport (1:2)	2.00 * Sodium [e] + 1.00 * dopaminium(1+) [e] --> 2.00 * S	SLC6A2; SL	Transport, extracellular			4
GCHOLAt_pos	4	1	0	-0.19193	0.191931	2	1	GCHOLAt	glycocholate transport via bicarbonate countertransport	1.00 * Bicarbonate [c] + 1.00 * glycocholate [e] --> 1.00 *	SLCO1A1; S	Transport, extracellular			4
GLUt6_pos	5	1	0	-0.01603	0.016032	2	1	GLUt6	Glutamate transport via Na, H symport and K antiport	1.00 * proton [e] + 3.00 * Sodium [e] + 1.00 * potassium [	SLC1A1; SL	Transport, extracellular			4
GLYt7_311_r_neg	5	1	0	0.541023	0.541023	1	1	GLYt7_311	glycine reversible transport via sodium and chloride symport (3:1:1)	3.00 * Sodium [e] + 1.00 * Glycine [e] + 1.00 * Chloride [e]	SLC6A5	Transport, extracellular			4
HCO3_CLt_neg	4	1	0	-0.03983	0.039827	2	1	HCO3_CLt	bicarbonate transport (Cl-/HCO3- exchange)	1.00 * Bicarbonate [e] + 1.00 * Chloride [c] --> 1.00 * Bicar	SLC4A1; SL	Transport, extracellular			4
HISTAtu_neg	4	1	0	-0.10307	0.103066	2	1	HISTAtu	Histamine uniport	1.00 * histamium [e] --> 1.00 * histamium [c]SLC22A2; SL	SLC22A2; S	Transport, extracellular			4
HISTAtu_pos	4	1	0	-0.10307	0.103066	2	1	HISTAtu	Histamine uniport	1.00 * histamium [e] --> 1.00 * histamium [c]SLC22A2; SL	SLC22A2; S	Transport, extracellular			4
INSTt2r_neg	5	1	0	0.33103	0.33103	1	1	INSTt2r	inositol transport in via proton symport	1.00 * proton [e] + 1.00 * myo-inositol [e] --> 1.00 * proto	SLC2A13	Transport, extracellular			4
INSTt2r_pos	5	1	0	0.33103	0.33103	1	1	INSTt2r	inositol transport in via proton symport	1.00 * proton [e] + 1.00 * myo-inositol [e] --> 1.00 * proto	SLC2A13	Transport, extracellular			4
INSTt4_neg	5	1	0	-0.39897	0.398975	2	1	INSTt4	inositol transport via sodium symport	1.00 * Sodium [e] + 1.00 * myo-inositol [e] --> 1.00 * Sodi	SLC5A3	Transport, extracellular			4
INSTt4_pos	5	1	0	0.33103	0.33103	1	1	INSTt4	inositol transport via sodium symport	1.00 * Sodium [e] + 1.00 * myo-inositol [e] --> 1.00 * Sodi	SLC5A3	Transport, extracellular			4
It_neg	4	1	0	0.787431	0.787431	1	1	It	Iodide uniport	1.00 * iodide [e] --> 1.00 * iodide [c]SLC5A8	SLC5A8	Transport, extracellular			4
It_pos	5	1	0	-0.50353	0.50353	2	1	It	Iodide uniport	1.00 * iodide [e] --> 1.00 * iodide [c]SLC5A8	SLC5A8	Transport, extracellular			4
METtec_pos	5	1	0	0.426401	0.426401	1	1	METtec	L-methionine transport via diffusion (extracellular to cytosol)	1.00 * L-methionine [e] --> 1.00 * L-methionine [c]SLC43A	SLC43A1; S	Transport, extracellular			4
NAIt_neg	4	1	0	0.72995	0.72995	1	1	NAIt	Na+ / iodide cotransport	2.00 * Sodium [e] + 1.00 * iodide [e] --> 2.00 * Sodium [c]	SLC5A5	Transport, extracellular			4
NAt5_neg	4	1	0	-0.24311	0.243107	2	1	NAt5	sodium/ammonium proton antiporter	1.00 * Sodium [c] + 1.00 * Ammonium [e] --> 1.00 * Amm	SLC9A1; SL	Transport, extracellular			4
NCKt_neg	4	1	0	-0.20667	0.206673	2	1	NCKt	Na+/Ca2+-K+ exchange	4.00 * Sodium [c] + 1.00 * potassium [e] + 1.00 * calcium(	SLC24A1; S	Transport, extracellular			4
NCKt_pos	4	1	0	-0.20667	0.206673	2	1	NCKt	Na+/Ca2+-K+ exchange	4.00 * Sodium [c] + 1.00 * potassium [e] + 1.00 * calcium(	SLC24A1; S	Transport, extracellular			4
NCNt_neg	4	1	0	-0.25173	0.251729	2	1	NCNt	Na+/Ca2+-NH4+ exchange	4.00 * Sodium [c] + 1.00 * Ammonium [e] + 1.00 * calciun	SLC24A1; S	Transport, extracellular			4
NCNt_pos	4	1	0	-0.25173	0.251729	2	1	NCNt	Na+/Ca2+-NH4+ exchange	4.00 * Sodium [c] + 1.00 * Ammonium [e] + 1.00 * calciun	SLC24A1; S	Transport, extracellular			4
NRPPHRt4_2_r_neg	4	1	0	0.168047	0.168047	1	1	NRPPHRt4_	Norepinephrine reversible transport in via sodium symport (1:2)	2.00 * Sodium [e] + 1.00 * Norepinephrine [e] --> 2.00 * So	SLC6A2; SL	Transport, extracellular			4
NRPPHRt4_2_r_pos	4	1	0	0.210376	0.210376	1	1	NRPPHRt4_	Norepinephrine reversible transport in via sodium symport (1:2)	2.00 * Sodium [e] + 1.00 * Norepinephrine [e] --> 2.00 * So	SLC6A2; SL	Transport, extracellular			4
NRPPHRtu_neg	4	1	0	-0.21047	0.210468	2	1	NRPPHRtu	Norepinephrine uniport	1.00 * Norepinephrine [e] --> 1.00 * Norepinephrine [c]SL	SLC22A1; S	Transport, extracellular			4
NRPPHRtu_pos	4	1	0	-0.21047	0.210468	2	1	NRPPHRtu	Norepinephrine uniport	1.00 * Norepinephrine [e] --> 1.00 * Norepinephrine [c]SL	SLC22A1; S	Transport, extracellular			4
PHEtec_neg	4	1	0	-0.14896	0.148963	2	1	PHEtec	L-phenylalanine transport via diffusion (extracellular to cytosol)	1.00 * L-phenylalanine [e] --> 1.00 * L-phenylalanine [c]SL	SLC16A10;	Transport, extracellular			4
SO4t4_2_neg	4	1	0	-0.00519	0.005193	2	1	SO4t4_2	sulfate transport via sodium symport	2.00 * Sodium [e] + 1.00 * sulfate [e] --> 2.00 * Sodium [c]	SLC13A4	Transport, extracellular			4
SRTNt6_2_r_neg	4	1	0	-0.05534	0.055341	2	1	SRTNt6_2_	Seratonin reversible transport in via sodium symport/potassium ant	2.00 * Sodium [e] + 2.00 * potassium [c] + 1.00 * serotoni	SLC6A4	Transport, extracellular			4
TCHOLAt_pos	4	1	0	-0.1917	0.191698	2	1	TCHOLAt	taurocholate transport via bicarbonate countertransport	1.00 * Bicarbonate [c] + 1.00 * taurocholate [e] --> 1.00 *	SLCO1A1; S	Transport, extracellular			4
TYRt_neg	5	1	0	0.370945	0.370945	1	1	TYRt	L-tyrosine transport	1.00 * L-tyrosine [e] --> 1.00 * L-tyrosine [c]SLC16A10	SLC16A10	Transport, extracellular			4
TYRt_pos	5	1	0	0.370945	0.370945	1	1	TYRt	L-tyrosine transport	1.00 * L-tyrosine [e] --> 1.00 * L-tyrosine [c]SLC16A10	SLC16A10	Transport, extracellular			4
r0879_pos	5	1	0	0.142464	0.142464	1	1	r0879	TCDB:2.A.29.2.4 TCDB:2.A.29.16.1	1.00 * Sodium [e] + 1.00 * 2-Oxoglutarate [e] --> 1.00 * 2-	SLC25A19;	Transport, extracellular			0
r0899_pos	5	1	0	0.288107	0.288107	1	1	r0899	Amino acid transporter ATB0+ Facilitated diffusion	1.00 * beta-alanine [c] --> 1.00 * beta-alanine [e]No genes	1.00 * beta	Transport, extracellular			0
r0963_pos	5	1	0	0.0576	0.0576	1	1	r0963	Facilitated diffusion	1.00 * Folate [e] --> 1.00 * Folate [c]FOLR1; FOLR2	FOLR1; FOL	Transport, extracellular			0
r1492_pos	5	1	0	0.06379	0.06379	1	1	r1492	Utilized transport	1.00 * potassium [c] --> 1.00 * potassium [e]ABCC8; ABCC	ABCC8; ABC	Transport, extracellular		6.2.1.3	0
r1632_neg	4	1	0	0.046539	0.046539	1	1	r1632	Amino Acid-Polyamine-Organocation (APC) TCDB:2.A.3.8.1	1.00 * L-threonine [c] + 1.00 * L-tyrosine [e] --> 1.00 * L-th	SLC7A5	Transport, extracellular			0
r2093_neg	5	1	0	0.238242	0.238242	1	1	r2093	Major Facilitator(MFS) TCDB:2.A.1.13.1	1.00 * 2-Oxoglutarate [c] + 1.00 * pyruvate [e] --> 1.00 * p	SLC16A1	Transport, extracellular			0
r2101_pos	4	1	0	-0.07152	0.071519	2	1	r2101	Major Facilitator(MFS) TCDB:2.A.1.13.1	1.00 * acetate [c] + 1.00 * 2-Oxoglutarate [e] --> 1.00 * 2-O	SLC16A1	Transport, extracellular			0
r2102_pos	4	1	0	-0.07152	0.071519	2	1	r2102	Major Facilitator(MFS) TCDB:2.A.1.13.1	1.00 * 2-Oxoglutarate [e] + 1.00 * Formate [c] --> 1.00 * 2-	SLC16A1	Transport, extracellular			0
r2103_pos	4	1	0	-0.07152	0.071519	2	1	r2103	Major Facilitator(MFS) TCDB:2.A.1.13.1	1.00 * propionate [c] + 1.00 * 2-Oxoglutarate [e] --> 1.00 *	SLC16A1	Transport, extracellular			0
r2104_pos	5	1	0	0.233676	0.233676	1	1	r2104	Major Facilitator(MFS) TCDB:2.A.1.13.1	1.00 * acetoacetate [c] + 1.00 * 2-Oxoglutarate [e] --> 1.00	SLC16A1	Transport, extracellular			0
r2105_pos	5	1	0	0.238242	0.238242	1	1	r2105	Major Facilitator(MFS) TCDB:2.A.1.13.1	1.00 * 2-Oxoglutarate [e] + 1.00 * (S)-lactate [c] --> 1.00 *	SLC16A1	Transport, extracellular			0
r2106_pos	4	1	0	-0.08049	0.080493	2	1	r2106	Major Facilitator(MFS) TCDB:2.A.1.13.1	1.00 * 2-Oxoglutarate [e] + 1.00 * butyrate [c] --> 1.00 * 2-	SLC16A1	Transport, extracellular			0
r2108_pos	4	1	0	-0.07152	0.071519	2	1	r2108	Major Facilitator(MFS) TCDB:2.A.1.13.1	1.00 * 2-Oxoglutarate [e] + 1.00 * (R)-3-hydroxybutyrate [	SLC16A1	Transport, extracellular			0
r2109_pos	5	1	0	0.238242	0.238242	1	1	r2109	Major Facilitator(MFS) TCDB:2.A.1.13.1	1.00 * 2-Hydroxybutyrate [c] + 1.00 * 2-Oxoglutarate [e] -	SLC16A1	Transport, extracellular			0
r2139_neg	4	1	0	-0.21608	0.216076	2	1	r2139	Resistance-Nodulation-Cell Division (RND) TCDB:2.A.60.1.14	1.00 * Bicarbonate [c] + 1.00 * Cholate [e] + 1.00 * Reduce	SLCO1A1	Transport, extracellular			0
r2139_pos	4	1	0	-0.21608	0.216076	2	1	r2139	Resistance-Nodulation-Cell Division (RND) TCDB:2.A.60.1.14	1.00 * Bicarbonate [c] + 1.00 * Cholate [e] + 1.00 * Reduce	SLCO1A1	Transport, extracellular			0
r2140_neg	4	1	0	-0.21328	0.213278	2	1	r2140	Resistance-Nodulation-Cell Division (RND) TCDB:2.A.60.1.14	1.00 * Bicarbonate [c] + 1.00 * Reduced glutathione [c] +	SLCO1A1	Transport, extracellular			0
r2140_pos	4	1	0	-0.21328	0.213278	2	1	r2140	Resistance-Nodulation-Cell Division (RND) TCDB:2.A.60.1.14	1.00 * Bicarbonate [c] + 1.00 * Reduced glutathione [c] +	SLCO1A1	Transport, extracellular			0
r2141_neg	4	1	0	-0.21304	0.213043	2	1	r2141	Resistance-Nodulation-Cell Division (RND) TCDB:2.A.60.1.14	1.00 * Bicarbonate [c] + 1.00 * Reduced glutathione [c] +	SLCO1A1	Transport, extracellular			0
r2141_pos	4	1	0	-0.21304	0.213043	2	1	r2141	Resistance-Nodulation-Cell Division (RND) TCDB:2.A.60.1.14	1.00 * Bicarbonate [c] + 1.00 * Reduced glutathione [c] +	SLCO1A1	Transport, extracellular			0
r2142_neg	4	1	0	-0.10035	0.100347	2	1	r2142	Resistance-Nodulation-Cell Division (RND) TCDB:2.A.60.1.14	1.00 * Bicarbonate [c] + 1.00 * Reduced glutathione [c] +	SLCO1A1	Transport, extracellular			0
r2142_pos	4	1	0	-0.10035	0.100347	2	1	r2142	Resistance-Nodulation-Cell Division (RND) TCDB:2.A.60.1.14	1.00 * Bicarbonate [c] + 1.00 * Reduced glutathione [c] +	SLCO1A1	Transport, extracellular			0
r2143_neg	4	1	0	-0.10035	0.100347	2	1	r2143	Resistance-Nodulation-Cell Division (RND) TCDB:2.A.60.1.14	1.00 * Bicarbonate [c] + 1.00 * Reduced glutathione [c] +	SLCO1A1	Transport, extracellular			0
r2143_pos	4	1	0	-0.10035	0.100347	2	1	r2143	Resistance-Nodulation-Cell Division (RND) TCDB:2.A.60.1.14	1.00 * Bicarbonate [c] + 1.00 * Reduced glutathione [c] +	SLCO1A1	Transport, extracellular			0
r2144_neg	4	1	0	-0.10035	0.100347	2	1	r2144	Resistance-Nodulation-Cell Division (RND) TCDB:2.A.60.1.14	1.00 * Bicarbonate [c] + 1.00 * Reduced glutathione [c] +	SLCO1A1	Transport, extracellular			0
r2144_pos	4	1	0	-0.10035	0.100347	2	1	r2144	Resistance-Nodulation-Cell Division (RND) TCDB:2.A.60.1.14	1.00 * Bicarbonate [c] + 1.00 * Reduced glutathione [c] +	SLCO1A1	Transport, extracellular			0
r2145_neg	4	1	0	-0.10035	0.100347	2	1	r2145	Resistance-Nodulation-Cell Division (RND) TCDB:2.A.60.1.14	1.00 * Bicarbonate [c] + 1.00 * Reduced glutathione [c] +	SLCO1A1	Transport, extracellular			0
r2145_pos	4	1	0	-0.10035	0.100347	2	1	r2145	Resistance-Nodulation-Cell Division (RND) TCDB:2.A.60.1.14	1.00 * Bicarbonate [c] + 1.00 * Reduced glutathione [c] +	SLCO1A1	Transport, extracellular			0
r2146_neg	4	1	0	-0.10035	0.100347	2	1	r2146	Resistance-Nodulation-Cell Division (RND) TCDB:2.A.60.1.14	1.00 * Bicarbonate [c] + 1.00 * Reduced glutathione [c] +	SLCO1A1	Transport, extracellular			0
r2146_pos	4	1	0	-0.10035	0.100347	2	1	r2146	Resistance-Nodulation-Cell Division (RND) TCDB:2.A.60.1.14	1.00 * Bicarbonate [c] + 1.00 * Reduced glutathione [c] +	SLCO1A1	Transport, extracellular			0
r2147_neg	4	1	0	-0.10035	0.100347	2	1	r2147	Resistance-Nodulation-Cell Division (RND) TCDB:2.A.60.1.14	1.00 * Bicarbonate [c] + 1.00 * Reduced glutathione [c] +	SLCO1A1	Transport, extracellular			0
r2147_pos	4	1	0	-0.10035	0.100347	2	1	r2147	Resistance-Nodulation-Cell Division (RND) TCDB:2.A.60.1.14	1.00 * Bicarbonate [c] + 1.00 * Reduced glutathione [c] +	SLCO1A1	Transport, extracellular			0
r2148_neg	4	1	0	-0.10035	0.100347	2	1	r2148	Resistance-Nodulation-Cell Division (RND) TCDB:2.A.60.1.14	1.00 * Bicarbonate [c] + 1.00 * Reduced glutathione [c] +	SLCO1A1	Transport, extracellular			0
r2148_pos	4	1	0	-0.10035	0.100347	2	1	r2148	Resistance-Nodulation-Cell Division (RND) TCDB:2.A.60.1.14	1.00 * Bicarbonate [c] + 1.00 * Reduced glutathione [c] +	SLCO1A1	Transport, extracellular			0
r2149_neg	4	1	0	-0.10035	0.100347	2	1	r2149	Resistance-Nodulation-Cell Division (RND) TCDB:2.A.60.1.14	1.00 * Bicarbonate [c] + 1.00 * Reduced glutathione [c] +	SLCO1A1	Transport, extracellular			0
r2149_pos	4	1	0	-0.10035	0.100347	2	1	r2149	Resistance-Nodulation-Cell Division (RND) TCDB:2.A.60.1.14	1.00 * Bicarbonate [c] + 1.00 * Reduced glutathione [c] +	SLCO1A1	Transport, extracellular			0
r2150_neg	4	1	0	-0.10035	0.100347	2	1	r2150	Resistance-Nodulation-Cell Division (RND) TCDB:2.A.60.1.14	1.00 * Bicarbonate [c] + 1.00 * Reduced glutathione [c] +	SLCO1A1	Transport, extracellular			0
r2150_pos	4	1	0	-0.10035	0.100347	2	1	r2150	Resistance-Nodulation-Cell Division (RND) TCDB:2.A.60.1.14	1.00 * Bicarbonate [c] + 1.00 * Reduced glutathione [c] +	SLCO1A1	Transport, extracellular			0
r2151_neg	4	1	0	-0.19467	0.194669	2	1	r2151	Resistance-Nodulation-Cell Division (RND) TCDB:2.A.60.1.14	1.00 * Bicarbonate [c] + 1.00 * Cholate [e] + 1.00 * glutath	SLCO1A1	Transport, extracellular			0
r2151_pos	4	1	0	-0.19467	0.194669	2	1	r2151	Resistance-Nodulation-Cell Division (RND) TCDB:2.A.60.1.14	1.00 * Bicarbonate [c] + 1.00 * Cholate [e] + 1.00 * glutath	SLCO1A1	Transport, extracellular			0
r2152_neg	4	1	0	-0.19467	0.194669	2	1	r2152	Resistance-Nodulation-Cell Division (RND) TCDB:2.A.60.1.14	1.00 * Bicarbonate [c] + 1.00 * Cholate [e] + 1.00 * S-gluta	SLCO1A1	Transport, extracellular			0
r2152_pos	4	1	0	-0.19467	0.194669	2	1	r2152	Resistance-Nodulation-Cell Division (RND) TCDB:2.A.60.1.14	1.00 * Bicarbonate [c] + 1.00 * Cholate [e] + 1.00 * S-gluta	SLCO1A1	Transport, extracellular			0
r2153_neg	4	1	0	-0.19467	0.194669	2	1	r2153	Resistance-Nodulation-Cell Division (RND) TCDB:2.A.60.1.14	1.00 * Bicarbonate [c] + 1.00 * Cholate [e] + 1.00 * S-gluta	SLCO1A1	Transport, extracellular			0
r2153_pos	4	1	0	-0.19467	0.194669	2	1	r2153	Resistance-Nodulation-Cell Division (RND) TCDB:2.A.60.1.14	1.00 * Bicarbonate [c] + 1.00 * Cholate [e] + 1.00 * S-gluta	SLCO1A1	Transport, extracellular			0
r2154_neg	4	1	0	-0.19193	0.191931	2	1	r2154	Resistance-Nodulation-Cell Division (RND) TCDB:2.A.60.1.14	1.00 * Bicarbonate [c] + 1.00 * glycocholate [e] + 1.00 * gl	SLCO1A1	Transport, extracellular			0
r2154_pos	4	1	0	-0.19193	0.191931	2	1	r2154	Resistance-Nodulation-Cell Division (RND) TCDB:2.A.60.1.14	1.00 * Bicarbonate [c] + 1.00 * glycocholate [e] + 1.00 * gl	SLCO1A1	Transport, extracellular			0
r2155_neg	4	1	0	-0.19193	0.191931	2	1	r2155	Resistance-Nodulation-Cell Division (RND) TCDB:2.A.60.1.14	1.00 * Bicarbonate [c] + 1.00 * glycocholate [e] + 1.00 * S-	SLCO1A1	Transport, extracellular			0
r2155_pos	4	1	0	-0.19193	0.191931	2	1	r2155	Resistance-Nodulation-Cell Division (RND) TCDB:2.A.60.1.14	1.00 * Bicarbonate [c] + 1.00 * glycocholate [e] + 1.00 * S-	SLCO1A1	Transport, extracellular			0
r2156_neg	4	1	0	-0.19193	0.191931	2	1	r2156	Resistance-Nodulation-Cell Division (RND) TCDB:2.A.60.1.14	1.00 * Bicarbonate [c] + 1.00 * glycocholate [e] + 1.00 * S-	SLCO1A1	Transport, extracellular			0

r2156_pos	4	1	0	-0.19193	0.191931	2	1	r2156	Resistance-Nodulation-Cell Division (RND) TCDB:2.A.60.1.14	1.00 * Bicarbonate [c] + 1.00 * glycocholate [e] + 1.00 * S-	SLCO1A1	Transport, extracellular			0
r2157_pos	4	1	0	-0.10035	0.100347	2	1	r2157	Resistance-Nodulation-Cell Division (RND) TCDB:2.A.60.1.14	1.00 * Bicarbonate [c] + 1.00 * chenodeoxycholate [e] + 1.	SLCO1A1	Transport, extracellular			0
r2158_pos	4	1	0	-0.10035	0.100347	2	1	r2158	Resistance-Nodulation-Cell Division (RND) TCDB:2.A.60.1.14	1.00 * Bicarbonate [c] + 1.00 * chenodeoxycholate [e] + 1.	SLCO1A1	Transport, extracellular			0
r2159_pos	4	1	0	-0.10035	0.100347	2	1	r2159	Resistance-Nodulation-Cell Division (RND) TCDB:2.A.60.1.14	1.00 * Bicarbonate [c] + 1.00 * chenodeoxycholate [e] + 1.	SLCO1A1	Transport, extracellular			0
r2160_neg	4	1	0	-0.1917	0.191698	2	1	r2160	Resistance-Nodulation-Cell Division (RND) TCDB:2.A.60.1.14	1.00 * Bicarbonate [c] + 1.00 * taurocholate [e] + 1.00 * gl	SLCO1A1	Transport, extracellular			0
r2160_pos	4	1	0	-0.1917	0.191698	2	1	r2160	Resistance-Nodulation-Cell Division (RND) TCDB:2.A.60.1.14	1.00 * Bicarbonate [c] + 1.00 * taurocholate [e] + 1.00 * gl	SLCO1A1	Transport, extracellular			0
r2161_neg	4	1	0	-0.1917	0.191698	2	1	r2161	Resistance-Nodulation-Cell Division (RND) TCDB:2.A.60.1.14	1.00 * Bicarbonate [c] + 1.00 * taurocholate [e] + 1.00 * S-	SLCO1A1	Transport, extracellular			0
r2161_pos	4	1	0	-0.1917	0.191698	2	1	r2161	Resistance-Nodulation-Cell Division (RND) TCDB:2.A.60.1.14	1.00 * Bicarbonate [c] + 1.00 * taurocholate [e] + 1.00 * S-	SLCO1A1	Transport, extracellular			0
r2162_neg	4	1	0	-0.1917	0.191698	2	1	r2162	Resistance-Nodulation-Cell Division (RND) TCDB:2.A.60.1.14	1.00 * Bicarbonate [c] + 1.00 * taurocholate [e] + 1.00 * S-	SLCO1A1	Transport, extracellular			0
r2162_pos	4	1	0	-0.1917	0.191698	2	1	r2162	Resistance-Nodulation-Cell Division (RND) TCDB:2.A.60.1.14	1.00 * Bicarbonate [c] + 1.00 * taurocholate [e] + 1.00 * S-	SLCO1A1	Transport, extracellular			0
r2163_pos	4	1	0	-0.10035	0.100347	2	1	r2163	Resistance-Nodulation-Cell Division (RND) TCDB:2.A.60.1.14	1.00 * Bicarbonate [c] + 1.00 * taurochenodeoxycholate [	SLCO1A1	Transport, extracellular			0
r2164_pos	4	1	0	-0.10035	0.100347	2	1	r2164	Resistance-Nodulation-Cell Division (RND) TCDB:2.A.60.1.14	1.00 * Bicarbonate [c] + 1.00 * taurochenodeoxycholate [	SLCO1A1	Transport, extracellular			0
r2165_pos	4	1	0	-0.10035	0.100347	2	1	r2165	Resistance-Nodulation-Cell Division (RND) TCDB:2.A.60.1.14	1.00 * Bicarbonate [c] + 1.00 * taurochenodeoxycholate [	SLCO1A1	Transport, extracellular			0
r2166_neg	4	1	0	-0.10035	0.100347	2	1	r2166	Resistance-Nodulation-Cell Division (RND) TCDB:2.A.60.1.14	1.00 * Bicarbonate [c] + 1.00 * glutathionyl-leuc4 [c] + 1.0	SLCO1A1	Transport, extracellular			0
r2166_pos	4	1	0	-0.10035	0.100347	2	1	r2166	Resistance-Nodulation-Cell Division (RND) TCDB:2.A.60.1.14	1.00 * Bicarbonate [c] + 1.00 * glutathionyl-leuc4 [c] + 1.0	SLCO1A1	Transport, extracellular			0
r2167_neg	4	1	0	-0.10035	0.100347	2	1	r2167	Resistance-Nodulation-Cell Division (RND) TCDB:2.A.60.1.14	1.00 * Bicarbonate [c] + 1.00 * S-glutathionyl-2-4-dinitro	SLCO1A1	Transport, extracellular			0
r2167_pos	4	1	0	-0.10035	0.100347	2	1	r2167	Resistance-Nodulation-Cell Division (RND) TCDB:2.A.60.1.14	1.00 * Bicarbonate [c] + 1.00 * S-glutathionyl-2-4-dinitro	SLCO1A1	Transport, extracellular			0
r2168_neg	4	1	0	-0.10035	0.100347	2	1	r2168	Resistance-Nodulation-Cell Division (RND) TCDB:2.A.60.1.14	1.00 * Bicarbonate [c] + 1.00 * S-glutathionyl-ethacrynic-	SLCO1A1	Transport, extracellular			0
r2168_pos	4	1	0	-0.10035	0.100347	2	1	r2168	Resistance-Nodulation-Cell Division (RND) TCDB:2.A.60.1.14	1.00 * Bicarbonate [c] + 1.00 * S-glutathionyl-ethacrynic-	SLCO1A1	Transport, extracellular			0
r2169_pos	4	1	0	-0.10035	0.100347	2	1	r2169	Resistance-Nodulation-Cell Division (RND) TCDB:2.A.60.1.14	1.00 * Bicarbonate [c] + 1.00 * taurolithocholate [e] + 1.0	SLCO1A1	Transport, extracellular			0
r2170_pos	4	1	0	-0.10035	0.100347	2	1	r2170	Resistance-Nodulation-Cell Division (RND) TCDB:2.A.60.1.14	1.00 * Bicarbonate [c] + 1.00 * taurolithocholate [e] + 1.0	SLCO1A1	Transport, extracellular			0
r2171_pos	4	1	0	-0.10035	0.100347	2	1	r2171	Resistance-Nodulation-Cell Division (RND) TCDB:2.A.60.1.14	1.00 * Bicarbonate [c] + 1.00 * taurolithocholate [e] + 1.0	SLCO1A1	Transport, extracellular			0
r2172_pos	4	1	0	-0.10035	0.100347	2	1	r2172	Resistance-Nodulation-Cell Division (RND) TCDB:2.A.60.1.14	1.00 * Bicarbonate [c] + 1.00 * glycolithocholate [e] + 1.0	SLCO1A1	Transport, extracellular			0
r2173_pos	4	1	0	-0.10035	0.100347	2	1	r2173	Resistance-Nodulation-Cell Division (RND) TCDB:2.A.60.1.14	1.00 * Bicarbonate [c] + 1.00 * glycolithocholate [e] + 1.0	SLCO1A1	Transport, extracellular			0
r2174_pos	4	1	0	-0.10035	0.100347	2	1	r2174	Resistance-Nodulation-Cell Division (RND) TCDB:2.A.60.1.14	1.00 * Bicarbonate [c] + 1.00 * glycolithocholate [e] + 1.0	SLCO1A1	Transport, extracellular			0
r2175_neg	4	1	0	-0.10035	0.100347	2	1	r2175	Resistance-Nodulation-Cell Division (RND) TCDB:2.A.60.1.14	1.00 * Bicarbonate [c] + 1.00 * glutathionyl-leuc4 [c] + 1.0	SLCO1A1	Transport, extracellular			0
r2175_pos	4	1	0	-0.10035	0.100347	2	1	r2175	Resistance-Nodulation-Cell Division (RND) TCDB:2.A.60.1.14	1.00 * Bicarbonate [c] + 1.00 * glutathionyl-leuc4 [c] + 1.0	SLCO1A1	Transport, extracellular			0
r2176_neg	4	1	0	-0.10035	0.100347	2	1	r2176	Resistance-Nodulation-Cell Division (RND) TCDB:2.A.60.1.14	1.00 * Bicarbonate [c] + 1.00 * S-glutathionyl-2-4-dinitro	SLCO1A1	Transport, extracellular			0
r2176_pos	4	1	0	-0.10035	0.100347	2	1	r2176	Resistance-Nodulation-Cell Division (RND) TCDB:2.A.60.1.14	1.00 * Bicarbonate [c] + 1.00 * S-glutathionyl-2-4-dinitro	SLCO1A1	Transport, extracellular			0
r2177_neg	4	1	0	-0.10035	0.100347	2	1	r2177	Resistance-Nodulation-Cell Division (RND) TCDB:2.A.60.1.14	1.00 * Bicarbonate [c] + 1.00 * S-glutathionyl-ethacrynic-	SLCO1A1	Transport, extracellular			0
r2177_pos	4	1	0	-0.10035	0.100347	2	1	r2177	Resistance-Nodulation-Cell Division (RND) TCDB:2.A.60.1.14	1.00 * Bicarbonate [c] + 1.00 * S-glutathionyl-ethacrynic-	SLCO1A1	Transport, extracellular			0
r2178_pos	4	1	0	-0.10035	0.100347	2	1	r2178	Resistance-Nodulation-Cell Division (RND) TCDB:2.A.60.1.14	1.00 * Bicarbonate [c] + 1.00 * tauroursodeoxycholate [e]	SLCO1A1	Transport, extracellular			0
r2179_pos	4	1	0	-0.10035	0.100347	2	1	r2179	Resistance-Nodulation-Cell Division (RND) TCDB:2.A.60.1.14	1.00 * Bicarbonate [c] + 1.00 * tauroursodeoxycholate [e]	SLCO1A1	Transport, extracellular			0
r2180_pos	4	1	0	-0.10035	0.100347	2	1	r2180	Resistance-Nodulation-Cell Division (RND) TCDB:2.A.60.1.14	1.00 * Bicarbonate [c] + 1.00 * tauroursodeoxycholate [e]	SLCO1A1	Transport, extracellular			0
r2181_pos	4	1	0	-0.10035	0.100347	2	1	r2181	Resistance-Nodulation-Cell Division (RND) TCDB:2.A.60.1.14	1.00 * Bicarbonate [c] + 1.00 * glycoursodeoxycholate [e]	SLCO1A1	Transport, extracellular			0
r2182_pos	4	1	0	-0.10035	0.100347	2	1	r2182	Resistance-Nodulation-Cell Division (RND) TCDB:2.A.60.1.14	1.00 * Bicarbonate [c] + 1.00 * glycoursodeoxycholate [e]	SLCO1A1	Transport, extracellular			0
r2183_pos	4	1	0	-0.10035	0.100347	2	1	r2183	Resistance-Nodulation-Cell Division (RND) TCDB:2.A.60.1.14	1.00 * Bicarbonate [c] + 1.00 * glycoursodeoxycholate [e]	SLCO1A1	Transport, extracellular			0
r2184_neg	4	1	0	-0.10035	0.100347	2	1	r2184	Resistance-Nodulation-Cell Division (RND) TCDB:2.A.60.1.14	1.00 * Bicarbonate [c] + 1.00 * glutathionyl-leuc4 [c] + 1.0	SLCO1A1	Transport, extracellular			0
r2184_pos	4	1	0	-0.10035	0.100347	2	1	r2184	Resistance-Nodulation-Cell Division (RND) TCDB:2.A.60.1.14	1.00 * Bicarbonate [c] + 1.00 * glutathionyl-leuc4 [c] + 1.0	SLCO1A1	Transport, extracellular			0
r2185_neg	4	1	0	-0.10035	0.100347	2	1	r2185	Resistance-Nodulation-Cell Division (RND) TCDB:2.A.60.1.14	1.00 * Bicarbonate [c] + 1.00 * S-glutathionyl-2-4-dinitro	SLCO1A1	Transport, extracellular			0
r2185_pos	4	1	0	-0.10035	0.100347	2	1	r2185	Resistance-Nodulation-Cell Division (RND) TCDB:2.A.60.1.14	1.00 * Bicarbonate [c] + 1.00 * S-glutathionyl-2-4-dinitro	SLCO1A1	Transport, extracellular			0
r2186_neg	4	1	0	-0.10035	0.100347	2	1	r2186	Resistance-Nodulation-Cell Division (RND) TCDB:2.A.60.1.14	1.00 * Bicarbonate [c] + 1.00 * S-glutathionyl-ethacrynic-	SLCO1A1	Transport, extracellular			0
r2186_pos	4	1	0	-0.10035	0.100347	2	1	r2186	Resistance-Nodulation-Cell Division (RND) TCDB:2.A.60.1.14	1.00 * Bicarbonate [c] + 1.00 * S-glutathionyl-ethacrynic-	SLCO1A1	Transport, extracellular			0
r2187_neg	4	1	0	-0.10035	0.100347	2	1	r2187	Resistance-Nodulation-Cell Division (RND) TCDB:2.A.60.1.14	1.00 * Bicarbonate [c] + 1.00 * glutathionyl-leuc4 [c] + 1.0	SLCO1A1	Transport, extracellular			0
r2187_pos	4	1	0	-0.10035	0.100347	2	1	r2187	Resistance-Nodulation-Cell Division (RND) TCDB:2.A.60.1.14	1.00 * Bicarbonate [c] + 1.00 * glutathionyl-leuc4 [c] + 1.0	SLCO1A1	Transport, extracellular			0
r2188_neg	4	1	0	-0.10035	0.100347	2	1	r2188	Resistance-Nodulation-Cell Division (RND) TCDB:2.A.60.1.14	1.00 * Bicarbonate [c] + 1.00 * S-glutathionyl-2-4-dinitro	SLCO1A1	Transport, extracellular			0
r2188_pos	4	1	0	-0.10035	0.100347	2	1	r2188	Resistance-Nodulation-Cell Division (RND) TCDB:2.A.60.1.14	1.00 * Bicarbonate [c] + 1.00 * S-glutathionyl-2-4-dinitro	SLCO1A1	Transport, extracellular			0
r2189_neg	4	1	0	-0.10035	0.100347	2	1	r2189	Resistance-Nodulation-Cell Division (RND) TCDB:2.A.60.1.14	1.00 * Bicarbonate [c] + 1.00 * S-glutathionyl-ethacrynic-	SLCO1A1	Transport, extracellular			0
r2189_pos	4	1	0	-0.10035	0.100347	2	1	r2189	Resistance-Nodulation-Cell Division (RND) TCDB:2.A.60.1.14	1.00 * Bicarbonate [c] + 1.00 * S-glutathionyl-ethacrynic-	SLCO1A1	Transport, extracellular			0
r2190_pos	4	1	0	-0.10035	0.100347	2	1	r2190	Resistance-Nodulation-Cell Division (RND) TCDB:2.A.60.1.14	1.00 * Bicarbonate [c] + 1.00 * sulfochenodeoxycholate [	SLCO1A1	Transport, extracellular			0
r2191_pos	4	1	0	-0.10035	0.100347	2	1	r2191	Resistance-Nodulation-Cell Division (RND) TCDB:2.A.60.1.14	1.00 * Bicarbonate [c] + 1.00 * sulfochenodeoxycholate [	SLCO1A1	Transport, extracellular			0
r2192_pos	4	1	0	-0.10035	0.100347	2	1	r2192	Resistance-Nodulation-Cell Division (RND) TCDB:2.A.60.1.14	1.00 * Bicarbonate [c] + 1.00 * sulfochenodeoxycholate [	SLCO1A1	Transport, extracellular			0
r2193_neg	4	1	0	-0.21713	0.217134	2	1	r2193	Resistance-Nodulation-Cell Division (RND) TCDB:2.A.60.1.14	1.00 * Bicarbonate [c] + 1.00 * bilirubin(2-) [e] + 1.00 * Re	SLCO1A1	Transport, extracellular			0
r2193_pos	4	1	0	-0.21713	0.217134	2	1	r2193	Resistance-Nodulation-Cell Division (RND) TCDB:2.A.60.1.14	1.00 * Bicarbonate [c] + 1.00 * bilirubin(2-) [e] + 1.00 * Re	SLCO1A1	Transport, extracellular			0
r2194_neg	4	1	0	-0.21579	0.215791	2	1	r2194	Resistance-Nodulation-Cell Division (RND) TCDB:2.A.60.1.14	1.00 * Bicarbonate [c] + 1.00 * Reduced glutathione [c] +	SLCO1A1	Transport, extracellular			0
r2194_pos	4	1	0	-0.21579	0.215791	2	1	r2194	Resistance-Nodulation-Cell Division (RND) TCDB:2.A.60.1.14	1.00 * Bicarbonate [c] + 1.00 * Reduced glutathione [c] +	SLCO1A1	Transport, extracellular			0
r2195_neg	4	1	0	-0.21304	0.213043	2	1	r2195	Resistance-Nodulation-Cell Division (RND) TCDB:2.A.60.1.14	1.00 * Bicarbonate [c] + 1.00 * Reduced glutathione [c] +	SLCO1A1	Transport, extracellular			0
r2195_pos	4	1	0	-0.21304	0.213043	2	1	r2195	Resistance-Nodulation-Cell Division (RND) TCDB:2.A.60.1.14	1.00 * Bicarbonate [c] + 1.00 * Reduced glutathione [c] +	SLCO1A1	Transport, extracellular			0
r2203_neg	4	1	0	-0.10734	0.107335	2	1	r2203	Resistance-Nodulation-Cell Division (RND) TCDB:2.A.60.1.14	1.00 * Bicarbonate [c] + 1.00 * bilirubin(2-) [e] + 1.00 * glu	SLCO1A1	Transport, extracellular			0
r2203_pos	4	1	0	-0.19569	0.195687	2	1	r2203	Resistance-Nodulation-Cell Division (RND) TCDB:2.A.60.1.14	1.00 * Bicarbonate [c] + 1.00 * bilirubin(2-) [e] + 1.00 * glu	SLCO1A1	Transport, extracellular			0
r2204_neg	4	1	0	-0.10734	0.107335	2	1	r2204	Resistance-Nodulation-Cell Division (RND) TCDB:2.A.60.1.14	1.00 * Bicarbonate [c] + 1.00 * bilirubin(2-) [e] + 1.00 * S-g	SLCO1A1	Transport, extracellular			0
r2204_pos	4	1	0	-0.19569	0.195687	2	1	r2204	Resistance-Nodulation-Cell Division (RND) TCDB:2.A.60.1.14	1.00 * Bicarbonate [c] + 1.00 * bilirubin(2-) [e] + 1.00 * S-g	SLCO1A1	Transport, extracellular			0
r2205_neg	4	1	0	-0.10734	0.107335	2	1	r2205	Resistance-Nodulation-Cell Division (RND) TCDB:2.A.60.1.14	1.00 * Bicarbonate [c] + 1.00 * bilirubin(2-) [e] + 1.00 * S-g	SLCO1A1	Transport, extracellular			0
r2205_pos	4	1	0	-0.19569	0.195687	2	1	r2205	Resistance-Nodulation-Cell Division (RND) TCDB:2.A.60.1.14	1.00 * Bicarbonate [c] + 1.00 * bilirubin(2-) [e] + 1.00 * S-g	SLCO1A1	Transport, extracellular			0
r2206_neg	4	1	0	-0.19439	0.194385	2	1	r2206	Resistance-Nodulation-Cell Division (RND) TCDB:2.A.60.1.14	1.00 * Bicarbonate [c] + 1.00 * L-thyroxine [e] + 1.00 * glu	SLCO1A1	Transport, extracellular			0
r2206_pos	4	1	0	-0.19439	0.194385	2	1	r2206	Resistance-Nodulation-Cell Division (RND) TCDB:2.A.60.1.14	1.00 * Bicarbonate [c] + 1.00 * L-thyroxine [e] + 1.00 * glu	SLCO1A1	Transport, extracellular			0
r2207_neg	4	1	0	-0.19439	0.194385	2	1	r2207	Resistance-Nodulation-Cell Division (RND) TCDB:2.A.60.1.14	1.00 * Bicarbonate [c] + 1.00 * L-thyroxine [e] + 1.00 * S-g	SLCO1A1	Transport, extracellular			0
r2207_pos	4	1	0	-0.19439	0.194385	2	1	r2207	Resistance-Nodulation-Cell Division (RND) TCDB:2.A.60.1.14	1.00 * Bicarbonate [c] + 1.00 * L-thyroxine [e] + 1.00 * S-g	SLCO1A1	Transport, extracellular			0
r2208_neg	4	1	0	-0.19439	0.194385	2	1	r2208	Resistance-Nodulation-Cell Division (RND) TCDB:2.A.60.1.14	1.00 * Bicarbonate [c] + 1.00 * L-thyroxine [e] + 1.00 * S-g	SLCO1A1	Transport, extracellular			0
r2208_pos	4	1	0	-0.19439	0.194385	2	1	r2208	Resistance-Nodulation-Cell Division (RND) TCDB:2.A.60.1.14	1.00 * Bicarbonate [c] + 1.00 * L-thyroxine [e] + 1.00 * S-g	SLCO1A1	Transport, extracellular			0
r2209_neg	4	1	0	-0.1917	0.191698	2	1	r2209	Resistance-Nodulation-Cell Division (RND) TCDB:2.A.60.1.14	1.00 * Bicarbonate [c] + 1.00 * 3,3',5-triiodo-L-thyronine	SLCO1A1	Transport, extracellular			0
r2209_pos	4	1	0	-0.1917	0.191698	2	1	r2209	Resistance-Nodulation-Cell Division (RND) TCDB:2.A.60.1.14	1.00 * Bicarbonate [c] + 1.00 * 3,3',5-triiodo-L-thyronine	SLCO1A1	Transport, extracellular			0
r2210_neg	4	1	0	-0.1917	0.191698	2	1	r2210	Resistance-Nodulation-Cell Division (RND) TCDB:2.A.60.1.14	1.00 * Bicarbonate [c] + 1.00 * 3,3',5-triiodo-L-thyronine	SLCO1A1	Transport, extracellular			0
r2210_pos	4	1	0	-0.1917	0.191698	2	1	r2210	Resistance-Nodulation-Cell Division (RND) TCDB:2.A.60.1.14	1.00 * Bicarbonate [c] + 1.00 * 3,3',5-triiodo-L-thyronine	SLCO1A1	Transport, extracellular			0
r2211_neg	4	1	0	-0.1917	0.191698	2	1	r2211	Resistance-Nodulation-Cell Division (RND) TCDB:2.A.60.1.14	1.00 * Bicarbonate [c] + 1.00 * 3,3',5-triiodo-L-thyronine	SLCO1A1	Transport, extracellular			0
r2211_pos	4	1	0	-0.1917	0.191698	2	1	r2211	Resistance-Nodulation-Cell Division (RND) TCDB:2.A.60.1.14	1.00 * Bicarbonate [c] + 1.00 * 3,3',5-triiodo-L-thyronine	SLCO1A1	Transport, extracellular			0
r2215_neg	4	1	0	-0.22234	0.222338	2	1	r2215	Resistance-Nodulation-Cell Division (RND) TCDB:2.A.60.1.14	1.00 * Bicarbonate [c] + 1.00 * prostaglandin E2(1-) [e] + 1	SLCO1A1	Transport, extracellular			0
r2215_pos	4	1	0	-0.22234	0.222338	2	1	r2215	Resistance-Nodulation-Cell Division (RND) TCDB:2.A.60.1.14	1.00 * Bicarbonate [c] + 1.00 * prostaglandin E2(1-) [e] + 1	SLCO1A1	Transport, extracellular			0
r2216_neg	4	1	0	-0.22234	0.222338	2	1	r2216	Resistance-Nodulation-Cell Division (RND) TCDB:2.A.60.1.14	1.00 * Bicarbonate [c] + 1.00 * prostaglandin E2(1-) [e] + 1	SLCO1A1	Transport, extracellular			0
r2216_pos	4	1	0	-0.22234	0.222338	2	1	r2216	Resistance-Nodulation-Cell Division (RND) TCDB:2.A.60.1.14	1.00 * Bicarbonate [c] + 1.00 * prostaglandin E2(1-) [e] + 1	SLCO1A1	Transport, extracellular			0
r2217_neg	4	1	0	-0.22234	0.222338	2	1	r2217	Resistance-Nodulation-Cell Division (RND) TCDB:2.A.60.1.14	1.00 * Bicarbonate [c] + 1.00 * prostaglandin E2(1-) [e] + 1	SLCO1A1	Transport, extracellular			0
r2217_pos	4	1	0	-0.22234	0.222338	2	1	r2217	Resistance-Nodulation-Cell Division (RND) TCDB:2.A.60.1.14	1.00 * Bicarbonate [c] + 1.00 * prostaglandin E2(1-) [e] + 1	SLCO1A1	Transport, extracellular			0
r2308_neg	4	1	0	-0.38204	0.382035	2	1	r2308	Resistance-Nodulation-Cell Division (RND) TCDB:2.A.60.1.14	1.00 * Bicarbonate [c] + 1.00 * Reduced glutathione [c] +	SLCO1A1	Transport, extracellular			0

r2308_pos	4	1	0	-0.38204	0.382035	2	1	r2308	Resistance-Nodulation-Cell Division (RND) TCDB:2.A.60.1.14	1.00 * Bicarbonate [c] + 1.00 * Reduced glutathione [c] + 1	SLCO1A1	Transport, extracellular			0
r2312_neg	4	1	0	-0.38204	0.382035	2	1	r2312	Resistance-Nodulation-Cell Division (RND) TCDB:2.A.60.1.14	1.00 * Bicarbonate [c] + 1.00 * Reduced glutathione [c] + 1	SLCO1A1	Transport, extracellular			0
r2312_pos	4	1	0	-0.38204	0.382035	2	1	r2312	Resistance-Nodulation-Cell Division (RND) TCDB:2.A.60.1.14	1.00 * Bicarbonate [c] + 1.00 * Reduced glutathione [c] + 1	SLCO1A1	Transport, extracellular			0
r2338_neg	4	1	0	-0.22233	0.222328	2	1	r2338	Major Facilitator(MFS) TCDB:2.A.1.19.1	1.00 * acetylcholine [e] --> 1.00 * acetylcholine [c]SLC22A	SLC22A1	Transport, extracellular			0
r2338_pos	4	1	0	-0.22233	0.222328	2	1	r2338	Major Facilitator(MFS) TCDB:2.A.1.19.1	1.00 * acetylcholine [e] --> 1.00 * acetylcholine [c]SLC22A	SLC22A1	Transport, extracellular			0
r2370_pos	4	1	0	-0.52556	0.525562	2	1	r2370	Nucleobase:Cation Symporter-2 (NCS2) TCDB:2.A.40.6.1	2.00 * Sodium [e] + 1.00 * L-ascorbate [e] --> 2.00 * Sodiur	SLC23A1	Transport, extracellular			0
r2482_neg	4	1	0	-0.10307	0.103066	2	1	r2482	Resistance-Nodulation-Cell Division (RND) TCDB:2.A.60.1.14	1.00 * Bicarbonate [e] + 1.00 * histamium [c] + 1.00 * glut	SLCO1A1	Transport, extracellular			0
r2482_pos	4	1	0	-0.10307	0.103066	2	1	r2482	Resistance-Nodulation-Cell Division (RND) TCDB:2.A.60.1.14	1.00 * Bicarbonate [e] + 1.00 * histamium [c] + 1.00 * glut	SLCO1A1	Transport, extracellular			0
r2483_neg	4	1	0	-0.10307	0.103066	2	1	r2483	Resistance-Nodulation-Cell Division (RND) TCDB:2.A.60.1.14	1.00 * Bicarbonate [e] + 1.00 * histamium [c] + 1.00 * S-gl	SLCO1A1	Transport, extracellular			0
r2483_pos	4	1	0	-0.10307	0.103066	2	1	r2483	Resistance-Nodulation-Cell Division (RND) TCDB:2.A.60.1.14	1.00 * Bicarbonate [e] + 1.00 * histamium [c] + 1.00 * S-gl	SLCO1A1	Transport, extracellular			0
r2484_neg	4	1	0	-0.10307	0.103066	2	1	r2484	Resistance-Nodulation-Cell Division (RND) TCDB:2.A.60.1.14	1.00 * Bicarbonate [e] + 1.00 * histamium [c] + 1.00 * S-gl	SLCO1A1	Transport, extracellular			0
r2484_pos	4	1	0	-0.10307	0.103066	2	1	r2484	Resistance-Nodulation-Cell Division (RND) TCDB:2.A.60.1.14	1.00 * Bicarbonate [e] + 1.00 * histamium [c] + 1.00 * S-gl	SLCO1A1	Transport, extracellular			0
r2485_neg	4	1	0	-0.21047	0.210468	2	1	r2485	Resistance-Nodulation-Cell Division (RND) TCDB:2.A.60.1.14	1.00 * Bicarbonate [e] + 1.00 * Norepinephrine [c] + 1.00	SLCO1A1	Transport, extracellular			0
r2485_pos	4	1	0	-0.21047	0.210468	2	1	r2485	Resistance-Nodulation-Cell Division (RND) TCDB:2.A.60.1.14	1.00 * Bicarbonate [e] + 1.00 * Norepinephrine [c] + 1.00	SLCO1A1	Transport, extracellular			0
r2486_neg	4	1	0	-0.21047	0.210468	2	1	r2486	Resistance-Nodulation-Cell Division (RND) TCDB:2.A.60.1.14	1.00 * Bicarbonate [e] + 1.00 * Norepinephrine [c] + 1.00	SLCO1A1	Transport, extracellular			0
r2486_pos	4	1	0	-0.21047	0.210468	2	1	r2486	Resistance-Nodulation-Cell Division (RND) TCDB:2.A.60.1.14	1.00 * Bicarbonate [e] + 1.00 * Norepinephrine [c] + 1.00	SLCO1A1	Transport, extracellular			0
r2487_neg	4	1	0	-0.21047	0.210468	2	1	r2487	Resistance-Nodulation-Cell Division (RND) TCDB:2.A.60.1.14	1.00 * Bicarbonate [e] + 1.00 * Norepinephrine [c] + 1.00	SLCO1A1	Transport, extracellular			0
r2487_pos	4	1	0	-0.21047	0.210468	2	1	r2487	Resistance-Nodulation-Cell Division (RND) TCDB:2.A.60.1.14	1.00 * Bicarbonate [e] + 1.00 * Norepinephrine [c] + 1.00	SLCO1A1	Transport, extracellular			0
r2488_neg	4	1	0	-0.22233	0.222333	2	1	r2488	Resistance-Nodulation-Cell Division (RND) TCDB:2.A.60.1.14	1.00 * Bicarbonate [e] + 1.00 * serotonin(1+) [c] + 1.00 * g	SLCO1A1	Transport, extracellular			0
r2488_pos	4	1	0	-0.22233	0.222333	2	1	r2488	Resistance-Nodulation-Cell Division (RND) TCDB:2.A.60.1.14	1.00 * Bicarbonate [e] + 1.00 * serotonin(1+) [c] + 1.00 * g	SLCO1A1	Transport, extracellular			0
r2489_neg	4	1	0	-0.22233	0.222333	2	1	r2489	Resistance-Nodulation-Cell Division (RND) TCDB:2.A.60.1.14	1.00 * Bicarbonate [e] + 1.00 * serotonin(1+) [c] + 1.00 * S	SLCO1A1	Transport, extracellular			0
r2489_pos	4	1	0	-0.22233	0.222333	2	1	r2489	Resistance-Nodulation-Cell Division (RND) TCDB:2.A.60.1.14	1.00 * Bicarbonate [e] + 1.00 * serotonin(1+) [c] + 1.00 * S	SLCO1A1	Transport, extracellular			0
r2490_neg	4	1	0	-0.22233	0.222333	2	1	r2490	Resistance-Nodulation-Cell Division (RND) TCDB:2.A.60.1.14	1.00 * Bicarbonate [e] + 1.00 * serotonin(1+) [c] + 1.00 * S	SLCO1A1	Transport, extracellular			0
r2490_pos	4	1	0	-0.22233	0.222333	2	1	r2490	Resistance-Nodulation-Cell Division (RND) TCDB:2.A.60.1.14	1.00 * Bicarbonate [e] + 1.00 * serotonin(1+) [c] + 1.00 * S	SLCO1A1	Transport, extracellular			0
r2491_neg	4	1	0	-0.22233	0.222328	2	1	r2491	Resistance-Nodulation-Cell Division (RND) TCDB:2.A.60.1.14	1.00 * Bicarbonate [e] + 1.00 * acetylcholine [c] + 1.00 * g	SLCO1A1	Transport, extracellular			0
r2491_pos	4	1	0	-0.22233	0.222328	2	1	r2491	Resistance-Nodulation-Cell Division (RND) TCDB:2.A.60.1.14	1.00 * Bicarbonate [e] + 1.00 * acetylcholine [c] + 1.00 * g	SLCO1A1	Transport, extracellular			0
r2492_neg	4	1	0	-0.22233	0.222328	2	1	r2492	Resistance-Nodulation-Cell Division (RND) TCDB:2.A.60.1.14	1.00 * Bicarbonate [e] + 1.00 * acetylcholine [c] + 1.00 * S	SLCO1A1	Transport, extracellular			0
r2492_pos	4	1	0	-0.22233	0.222328	2	1	r2492	Resistance-Nodulation-Cell Division (RND) TCDB:2.A.60.1.14	1.00 * Bicarbonate [e] + 1.00 * acetylcholine [c] + 1.00 * S	SLCO1A1	Transport, extracellular			0
r2493_neg	4	1	0	-0.22233	0.222328	2	1	r2493	Resistance-Nodulation-Cell Division (RND) TCDB:2.A.60.1.14	1.00 * Bicarbonate [e] + 1.00 * acetylcholine [c] + 1.00 * S	SLCO1A1	Transport, extracellular			0
r2493_pos	4	1	0	-0.22233	0.222328	2	1	r2493	Resistance-Nodulation-Cell Division (RND) TCDB:2.A.60.1.14	1.00 * Bicarbonate [e] + 1.00 * acetylcholine [c] + 1.00 * S	SLCO1A1	Transport, extracellular			0
r2494_neg	4	1	0	-0.10307	0.103066	2	1	r2494	Resistance-Nodulation-Cell Division (RND) TCDB:2.A.60.1.14	1.00 * Bicarbonate [e] + 1.00 * dopaminium(1+) [c] + 1.00	SLCO1A1	Transport, extracellular			0
r2494_pos	4	1	0	-0.10307	0.103066	2	1	r2494	Resistance-Nodulation-Cell Division (RND) TCDB:2.A.60.1.14	1.00 * Bicarbonate [e] + 1.00 * dopaminium(1+) [c] + 1.00	SLCO1A1	Transport, extracellular			0
r2495_neg	4	1	0	-0.10307	0.103066	2	1	r2495	Resistance-Nodulation-Cell Division (RND) TCDB:2.A.60.1.14	1.00 * Bicarbonate [e] + 1.00 * dopaminium(1+) [c] + 1.00	SLCO1A1	Transport, extracellular			0
r2495_pos	4	1	0	-0.10307	0.103066	2	1	r2495	Resistance-Nodulation-Cell Division (RND) TCDB:2.A.60.1.14	1.00 * Bicarbonate [e] + 1.00 * dopaminium(1+) [c] + 1.00	SLCO1A1	Transport, extracellular			0
r2496_neg	4	1	0	-0.10307	0.103066	2	1	r2496	Resistance-Nodulation-Cell Division (RND) TCDB:2.A.60.1.14	1.00 * Bicarbonate [e] + 1.00 * dopaminium(1+) [c] + 1.00	SLCO1A1	Transport, extracellular			0
r2496_pos	4	1	0	-0.10307	0.103066	2	1	r2496	Resistance-Nodulation-Cell Division (RND) TCDB:2.A.60.1.14	1.00 * Bicarbonate [e] + 1.00 * dopaminium(1+) [c] + 1.00	SLCO1A1	Transport, extracellular			0
r2534_neg	5	1	0	0.128895	0.128895	1	1	r2534	Major Facilitator(MFS) TCDB:2.A.1.44.1	1.00 * L-threonine [e] --> 1.00 * L-threonine [c]SLC43A1	SLC43A1	Transport, extracellular			0
r1052_pos	7	0.382733	0.4171041	1.272298	1.272298	1	0.518179	r1052	Vesicular transport	1.00 * cholesterol [l] --> 1.00 * cholesterol [c]No genes	1.00 * chol	Transport, lysosomal			0
r1301_pos	2	0.382733	0.4171041	-1.2911	1.291104	2	0.518179	r1301	Vesicular transport	1.00 * octadecenoate (n-C18:1) [l] --> 1.00 * octadecenoa	1.00 * octa	Transport, lysosomal			0
r1302_neg	2	0.382733	0.4171041	-1.2911	1.291104	2	0.518179	r1302	Vesicular transport	1.00 * octadecenoate (n-C18:1) [l] --> 1.00 * octadecenoa	1.00 * octa	Transport, lysosomal			0
AHCYStd_neg	0	0.080856	1.0922877	-2.62935	2.629346	2	0.240222	AHCYStd	diffusion of S-Adenosyl-L-homocysteine	1.00 * S-Adenosyl-L-homocysteine [m] --> 1.00 * S-Adenos	1.00 * S-Ad	Transport, mitochondrial			4
AHCYStd_pos	0	0.080856	1.0922877	-2.62935	2.629346	2	0.240222	AHCYStd	diffusion of S-Adenosyl-L-homocysteine	1.00 * S-Adenosyl-L-homocysteine [m] --> 1.00 * S-Adenos	1.00 * S-Ad	Transport, mitochondrial			4
AKGMALtm_neg	0	0.080856	1.0922877	-2.29702	2.297021	2	0.240222	AKGMALtm	alpha-ketoglutarate/malate transporter	1.00 * 2-Oxoglutarate [m] + 1.00 * (S)-malate(2-) [c] --> 1.0	SLC25A10;	Transport, mitochondrial			4
AKGMALtm_pos	0	0.080856	1.0922877	-2.29702	2.297021	2	0.240222	AKGMALtm	alpha-ketoglutarate/malate transporter	1.00 * 2-Oxoglutarate [m] + 1.00 * (S)-malate(2-) [c] --> 1.0	SLC25A10;	Transport, mitochondrial			4
AMETt2m_neg	0	0.080856	1.0922877	-2.62935	2.629346	2	0.240222	AMETt2m	S-Adenosyl-L-methionine reversible transport, mitochondrial	1.00 * S-Adenosyl-L-methionine [c] + 1.00 * S-Adenosyl-L-	SLC25A26	Transport, mitochondrial			4
AMETt2m_pos	0	0.080856	1.0922877	-2.62935	2.629346	2	0.240222	AMETt2m	S-Adenosyl-L-methionine reversible transport, mitochondrial	1.00 * S-Adenosyl-L-methionine [c] + 1.00 * S-Adenosyl-L-	SLC25A26	Transport, mitochondrial			4
ARGtm_neg	0	0.080856	1.0922877	-2.57949	2.579493	2	0.240222	ARGtm	arginine mitochondrial transport via ornithine carrier	1.00 * proton [m] + 1.00 * L-argininium(1+) [c] --> 1.00 * p	SLC25A2	Transport, mitochondrial			4
ARGtm_pos	0	0.080856	1.0922877	-2.57506	2.575055	2	0.240222	ARGtm	arginine mitochondrial transport via ornithine carrier	1.00 * proton [m] + 1.00 * L-argininium(1+) [c] --> 1.00 * p	SLC25A2	Transport, mitochondrial			4
CITRtm_neg	0	0.080856	1.0922877	-2.57949	2.579493	2	0.240222	CITRtm	citrulline mitochondrial transport via proton antiport	1.00 * L-citrulline [m] --> 1.00 * L-citrulline [c]SLC25A2	SLC25A2	Transport, mitochondrial			4
CITRtm_pos	0	0.080856	1.0922877	-2.57506	2.575055	2	0.240222	CITRtm	citrulline mitochondrial transport via proton antiport	1.00 * L-citrulline [m] --> 1.00 * L-citrulline [c]SLC25A2	SLC25A2	Transport, mitochondrial			4
CITtam_neg	0	0.080856	1.0922877	-1.94736	1.94736	2	0.240222	CITtam	citrate transport, mitochondrial	1.00 * Citrate [c] + 1.00 * (S)-malate(2-) [m] --> 1.00 * Citra	SLC25A1	Transport, mitochondrial			4
CITtam_pos	0	0.080856	1.0922877	-1.94736	1.94736	2	0.240222	CITtam	citrate transport, mitochondrial	1.00 * Citrate [c] + 1.00 * (S)-malate(2-) [m] --> 1.00 * Citra	SLC25A1	Transport, mitochondrial			4
CITtbm_pos	0	0.080856	1.0922877	-1.94736	1.94736	2	0.240222	CITtbm	citrate transport, mitochondrial	1.00 * Citrate [c] + 1.00 * Phosphoenolpyruvate [m] --> 1.	SLC25A1	Transport, mitochondrial			4
CO2tm_neg	0	0.080856	1.0922877	-2.31899	2.318987	2	0.240222	CO2tm	CO2 transport (diffusion), mitochondrial	1.00 * carbon dioxide [c] --> 1.00 * carbon dioxide [m]No	1.00 * carb	Transport, mitochondrial			4
CO2tm_pos	0	0.080856	1.0922877	-2.94107	2.941075	2	0.240222	CO2tm	CO2 transport (diffusion), mitochondrial	1.00 * carbon dioxide [c] --> 1.00 * carbon dioxide [m]No	1.00 * carb	Transport, mitochondrial			4
DNDPt29m_pos	9	0.080856	1.0922877	1.685929	1.685929	1	0.240222	DNDPt29m	dCDP transport via dADP antiport	1.00 * dCDP [c] + 1.00 * dADP [m] --> 1.00 * dADP [c] + 1.0	SLC25A19	Transport, mitochondrial			4
DNDPt35m_pos	9	0.080856	1.0922877	1.685929	1.685929	1	0.240222	DNDPt35m	dGDP transport via dADP antiport	1.00 * dGDP [c] + 1.00 * dADP [m] --> 1.00 * dADP [c] + 1.0	SLC25A19	Transport, mitochondrial			4
DNDPt40m_pos	9	0.080856	1.0922877	1.678948	1.678948	1	0.240222	DNDPt40m	dUTP transport via dADP antiport	1.00 * dADP [m] + 1.00 * dUTP [c] --> 1.00 * dADP [c] + 1.0	SLC25A19	Transport, mitochondrial			4
DNDPt8m_pos	9	0.080856	1.0922877	1.678948	1.678948	1	0.240222	DNDPt8m	dADP transport via dUDP antiport	1.00 * dADP [c] + 1.00 * dUDP [m] --> 1.00 * dADP [m] + 1.	SLC25A19	Transport, mitochondrial			4
DNDPt9m_pos	9	0.080856	1.0922877	1.685929	1.685929	1	0.240222	DNDPt9m	dADP transport via dTDP antiport	1.00 * dADP [c] + 1.00 * dTDP [m] --> 1.00 * dADP [m] + 1.	SLC25A19	Transport, mitochondrial			4
FUMSO3tm_neg	0	0.080856	1.0922877	-1.74841	1.748415	2	0.240222	FUMSO3tm	Fumarate:sulfite antiport, mitochondrial	1.00 * Sulfite [m] + 1.00 * Fumarate [c] --> 1.00 * Sulfite [c	SLC25A10	Transport, mitochondrial			4
FUMSO3tm_pos	0	0.080856	1.0922877	-1.74841	1.748415	2	0.240222	FUMSO3tm	Fumarate:sulfite antiport, mitochondrial	1.00 * Sulfite [m] + 1.00 * Fumarate [c] --> 1.00 * Sulfite [c	SLC25A10	Transport, mitochondrial			4
FUMSO4tm_neg	0	0.080856	1.0922877	-1.74841	1.748415	2	0.240222	FUMSO4tm	Fumarate:sulfate antiport, mitochondrial	1.00 * Fumarate [c] + 1.00 * sulfate [m] --> 1.00 * Fumarat	SLC25A10	Transport, mitochondrial			4
FUMSO4tm_pos	0	0.080856	1.0922877	-1.74841	1.748415	2	0.240222	FUMSO4tm	Fumarate:sulfate antiport, mitochondrial	1.00 * Fumarate [c] + 1.00 * sulfate [m] --> 1.00 * Fumarat	SLC25A10	Transport, mitochondrial			4
FUMTSULtm_neg	0	0.080856	1.0922877	-1.74841	1.748415	2	0.240222	FUMTSULt	Fumarate:thiosulfate antiport, mitochondrial	1.00 * Fumarate [c] + 1.00 * thiosulfate(2-) [m] --> 1.00 * F	SLC25A10	Transport, mitochondrial			4
FUMTSULtm_pos	0	0.080856	1.0922877	-1.74841	1.748415	2	0.240222	FUMTSULt	Fumarate:thiosulfate antiport, mitochondrial	1.00 * Fumarate [c] + 1.00 * thiosulfate(2-) [m] --> 1.00 * F	SLC25A10	Transport, mitochondrial			4
FUMtm_neg	0	0.080856	1.0922877	-1.74841	1.748415	2	0.240222	FUMtm	fumarate transport, mitochondrial	1.00 * hydrogenphosphate [m] + 1.00 * Fumarate [c] --> 1.	SLC25A10	Transport, mitochondrial			4
FUMtm_pos	0	0.080856	1.0922877	-1.74841	1.748415	2	0.240222	FUMtm	fumarate transport, mitochondrial	1.00 * hydrogenphosphate [m] + 1.00 * Fumarate [c] --> 1.	SLC25A10	Transport, mitochondrial			4
GLUt2m_pos	0	0.080856	1.0922877	-2.391	2.391	2	0.240222	GLUt2m	L-glutamate reversible transport via proton symport, mitochondrial	1.00 * proton [c] + 1.00 * L-glutamate(1-) [c] --> 1.00 * pro	SLC25A12;	Transport, mitochondrial			4
LYStm_neg	0	0.080856	1.0922877	-2.57949	2.579493	2	0.240222	LYStm	Lysine mitochondrial transport via ornithine carrier	1.00 * proton [m] + 1.00 * L-lysinium(1+) [c] --> 1.00 * pro	SLC25A2	Transport, mitochondrial			4
LYStm_pos	0	0.080856	1.0922877	-2.57506	2.575055	2	0.240222	LYStm	Lysine mitochondrial transport via ornithine carrier	1.00 * proton [m] + 1.00 * L-lysinium(1+) [c] --> 1.00 * pro	SLC25A2	Transport, mitochondrial			4
MALSO3tm_neg	0	0.080856	1.0922877	-2.27787	2.277875	2	0.240222	MALSO3tm	Malate:sulfite antiport, mitochondrial	1.00 * Sulfite [m] + 1.00 * (S)-malate(2-) [c] --> 1.00 * Sulfit	SLC25A10	Transport, mitochondrial			4
MALSO3tm_pos	0	0.080856	1.0922877	-2.27787	2.277875	2	0.240222	MALSO3tm	Malate:sulfite antiport, mitochondrial	1.00 * Sulfite [m] + 1.00 * (S)-malate(2-) [c] --> 1.00 * Sulfit	SLC25A10	Transport, mitochondrial			4
MALSO4tm_neg	0	0.080856	1.0922877	-2.27787	2.277875	2	0.240222	MALSO4tm	Malate:sulfate antiport, mitochondrial	1.00 * (S)-malate(2-) [c] + 1.00 * sulfate [m] --> 1.00 * (S)-m	SLC25A10	Transport, mitochondrial			4
MALSO4tm_pos	0	0.080856	1.0922877	-2.27787	2.277875	2	0.240222	MALSO4tm	Malate:sulfate antiport, mitochondrial	1.00 * (S)-malate(2-) [c] + 1.00 * sulfate [m] --> 1.00 * (S)-m	SLC25A10	Transport, mitochondrial			4
MALTSULtm_neg	0	0.080856	1.0922877	-1.74841	1.748415	2	0.240222	MALTSULt	Malate:thiosulfate antiport, mitochondrial	1.00 * (S)-malate(2-) [c] + 1.00 * thiosulfate(2-) [m] --> 1.0	SLC25A10	Transport, mitochondrial			4
MALTSULtm_pos	0	0.080856	1.0922877	-1.74841	1.748415	2	0.240222	MALTSULt	Malate:thiosulfate antiport, mitochondrial	1.00 * (S)-malate(2-) [c] + 1.00 * thiosulfate(2-) [m] --> 1.0	SLC25A10	Transport, mitochondrial			4
MALtm_pos	0	0.080856	1.0922877	-2.27787	2.277875	2	0.240222	MALtm	malate transport, mitochondrial	1.00 * hydrogenphosphate [m] + 1.00 * (S)-malate(2-) [c] -	SLC25A10	Transport, mitochondrial			4
ORNt3m_pos	0	0.080856	1.0922877	-2.57949	2.579493	2	0.240222	ORNt3m	ornithine mitochondrial transport via proton antiport	1.00 * proton [c] + 1.00 * Ornithine [m] --> 1.00 * proton	SLC25A2	Transport, mitochondrial			4
ORNt4m_pos	0	0.080856	1.0922877	-2.63549	2.635493	2	0.240222	ORNt4m	ornithine mitochondrial transport exchange with citruline	1.00 * proton [c] + 1.00 * Ornithine [m] + 1.00 * L-citrulli	SLC25A2	Transport, mitochondrial			4
SUCCt2m_neg	0	0.080856	1.0922877	-2.27787	2.277875	2	0.240222	SUCCt2m	succinate transport, mitochondrial	1.00 * hydrogenphosphate [m] + 1.00 * Succinate [c] --> 1	SLC25A10	Transport, mitochondrial			4
SUCCt2m_pos	0	0.080856	1.0922877	-2.27787	2.277875	2	0.240222	SUCCt2m	succinate transport, mitochondrial	1.00 * hydrogenphosphate [m] + 1.00 * Succinate [c] --> 1	SLC25A10	Transport, mitochondrial			4
r0819_neg	0	0.080856	1.0922877	-1.95861	1.958608	2	0.240222	r0819	Mitochondrial Carrier (MC) TCDB:2.A.29.2.2	1.00 * 2-Oxoglutarate [c] + 1.00 * Succinate [m] --> 1.00 *	SLC25A10	Transport, mitochondrial			0

r0819_pos	0	0.080856	1.0922877	-1.95861	1.958608	2	0.240222	r0819	Mitochondrial Carrier (MC) TCDB:2.A.29.2.2	1.00 * 2-Oxoglutarate [c] + 1.00 * Succinate [m] --> 1.00 *	SLC25A10	Transport, mitochondrial			0
r0821_neg	0	0.080856	1.0922877	-1.73617	1.736166	2	0.240222	r0821	Mitochondrial Carrier (MC) TCDB:2.A.29.2.2	1.00 * Fumarate [c] + 1.00 * Succinate [m] --> 1.00 * Succi	SLC25A10	Transport, mitochondrial			0
r0821_pos	0	0.080856	1.0922877	-1.73617	1.736166	2	0.240222	r0821	Mitochondrial Carrier (MC) TCDB:2.A.29.2.2	1.00 * Fumarate [c] + 1.00 * Succinate [m] --> 1.00 * Succi	SLC25A10	Transport, mitochondrial			0
r0822_neg	0	0.080856	1.0922877	-1.73617	1.736166	2	0.240222	r0822	Mitochondrial Carrier (MC) TCDB:2.A.29.2.2	1.00 * Fumarate [c] + 1.00 * (S)-malate(2-) [m] --> 1.00 * (S	SLC25A10	Transport, mitochondrial			0
r0822_pos	0	0.080856	1.0922877	-1.73617	1.736166	2	0.240222	r0822	Mitochondrial Carrier (MC) TCDB:2.A.29.2.2	1.00 * Fumarate [c] + 1.00 * (S)-malate(2-) [m] --> 1.00 * (S	SLC25A10	Transport, mitochondrial			0
r0829_neg	0	0.080856	1.0922877	-2.27787	2.277875	2	0.240222	r0829	Mitochondrial Carrier (MC) TCDB:2.A.29.2.2	1.00 * Succinate [c] + 1.00 * sulfate [m] --> 1.00 * sulfate [	SLC25A10	Transport, mitochondrial			0
r0829_pos	0	0.080856	1.0922877	-2.27787	2.277875	2	0.240222	r0829	Mitochondrial Carrier (MC) TCDB:2.A.29.2.2	1.00 * Succinate [c] + 1.00 * sulfate [m] --> 1.00 * sulfate [	SLC25A10	Transport, mitochondrial			0
r0830_neg	0	0.080856	1.0922877	-2.27787	2.277875	2	0.240222	r0830	Mitochondrial Carrier (MC) TCDB:2.A.29.2.2	1.00 * Sulfite [m] + 1.00 * Succinate [c] --> 1.00 * Sulfite [c	SLC25A10	Transport, mitochondrial			0
r0830_pos	0	0.080856	1.0922877	-2.27787	2.277875	2	0.240222	r0830	Mitochondrial Carrier (MC) TCDB:2.A.29.2.2	1.00 * Sulfite [m] + 1.00 * Succinate [c] --> 1.00 * Sulfite [c	SLC25A10	Transport, mitochondrial			0
r0834_neg	0	0.080856	1.0922877	-1.74841	1.748415	2	0.240222	r0834	Mitochondrial Carrier (MC) TCDB:2.A.29.2.2	1.00 * hydrogenphosphate [m] + 1.00 * Malonate [c] --> 1	SLC25A10	Transport, mitochondrial			0
r0834_pos	0	0.080856	1.0922877	-1.74841	1.748415	2	0.240222	r0834	Mitochondrial Carrier (MC) TCDB:2.A.29.2.2	1.00 * hydrogenphosphate [m] + 1.00 * Malonate [c] --> 1	SLC25A10	Transport, mitochondrial			0
r0835_neg	0	0.080856	1.0922877	-1.74841	1.748415	2	0.240222	r0835	Mitochondrial Carrier (MC) TCDB:2.A.29.2.2	1.00 * sulfate [m] + 1.00 * Malonate [c] --> 1.00 * sulfate [c	SLC25A10	Transport, mitochondrial			0
r0835_pos	0	0.080856	1.0922877	-1.74841	1.748415	2	0.240222	r0835	Mitochondrial Carrier (MC) TCDB:2.A.29.2.2	1.00 * sulfate [m] + 1.00 * Malonate [c] --> 1.00 * sulfate [c	SLC25A10	Transport, mitochondrial			0
r0836_neg	0	0.080856	1.0922877	-1.74841	1.748415	2	0.240222	r0836	Mitochondrial Carrier (MC) TCDB:2.A.29.2.2	1.00 * Sulfite [m] + 1.00 * Malonate [c] --> 1.00 * Sulfite [c	SLC25A10	Transport, mitochondrial			0
r0836_pos	0	0.080856	1.0922877	-1.74841	1.748415	2	0.240222	r0836	Mitochondrial Carrier (MC) TCDB:2.A.29.2.2	1.00 * Sulfite [m] + 1.00 * Malonate [c] --> 1.00 * Sulfite [c	SLC25A10	Transport, mitochondrial			0
r0911_neg	9	0.080856	1.0922877	2.494541	2.494541	1	0.240222	r0911	Facilitated diffusion	1.00 * L-glutamate(1-) [m] + 1.00 * L-proline [c] --> 1.00 *	1.00 * L-glu	Transport, mitochondrial			0
r0911_pos	0	0.080856	1.0922877	-2.391	2.391	2	0.240222	r0911	Facilitated diffusion	1.00 * L-glutamate(1-) [m] + 1.00 * L-proline [c] --> 1.00 *	1.00 * L-glu	Transport, mitochondrial			0
r0913_neg	0	0.080856	1.0922877	-1.94736	1.94736	2	0.240222	r0913	Mitochondrial Carrier (MC) TCDB:2.A.29.7.2	1.00 * Isocitrate [m] + 1.00 * (S)-malate(2-) [c] --> 1.00 * Is	SLC25A1	Transport, mitochondrial			0
r0913_pos	0	0.080856	1.0922877	-1.94736	1.94736	2	0.240222	r0913	Mitochondrial Carrier (MC) TCDB:2.A.29.7.2	1.00 * Isocitrate [m] + 1.00 * (S)-malate(2-) [c] --> 1.00 * Is	SLC25A1	Transport, mitochondrial			0
r0915_neg	0	0.080856	1.0922877	-1.94736	1.94736	2	0.240222	r0915	Mitochondrial Carrier (MC) TCDB:2.A.29.7.2	1.00 * Citrate [c] + 1.00 * Succinate [m] --> 1.00 * Citrate [	SLC25A1	Transport, mitochondrial			0
r0915_pos	0	0.080856	1.0922877	-1.94736	1.94736	2	0.240222	r0915	Mitochondrial Carrier (MC) TCDB:2.A.29.7.2	1.00 * Citrate [c] + 1.00 * Succinate [m] --> 1.00 * Citrate [	SLC25A1	Transport, mitochondrial			0
r0941_neg	0	0.080856	1.0922877	-2.94107	2.941075	2	0.240222	r0941	Free diffusion	1.00 * Bicarbonate [c] --> 1.00 * Bicarbonate [m]No genes	1.00 * Bica	Transport, mitochondrial			0
r0941_pos	0	0.080856	1.0922877	-2.31899	2.318987	2	0.240222	r0941	Free diffusion	1.00 * Bicarbonate [c] --> 1.00 * Bicarbonate [m]No genes	1.00 * Bica	Transport, mitochondrial			0
r0947_neg	0	0.080856	1.0922877	-2.63549	2.635493	2	0.240222	r0947	Mitochondrial Carrier (MC) TCDB:2.A.29.19.1	1.00 * Ornithine [m] + 1.00 * L-citrulline [c] --> 1.00 * Orn	SLC25A2	Transport, mitochondrial			0
r0947_pos	0	0.080856	1.0922877	-2.66231	2.662308	2	0.240222	r0947	Mitochondrial Carrier (MC) TCDB:2.A.29.19.1	1.00 * Ornithine [m] + 1.00 * L-citrulline [c] --> 1.00 * Orn	SLC25A2	Transport, mitochondrial			0
r1155_pos	0	0.080856	1.0922877	-3.09389	3.093893	2	0.240222	r1155	TCDB:2.A.1.13.5 TCDB:2.A.1.13.1	1.00 * proton [c] + 1.00 * 2-Oxobutanoate [c] --> 1.00 * pr	SLC16A1; S	Transport, mitochondrial			0
r1427_pos	0	0.080856	1.0922877	-2.42231	2.422306	2	0.240222	r1427	Mitochondrial Carrier (MC) TCDB:2.A.29.19.1	1.00 * proton [c] + 1.00 * L-histidine [c] --> 1.00 * proton	SLC25A2	Transport, mitochondrial		4.2.1.1	0
r1454_pos	0	0.080856	1.0922877	-3.09389	3.093893	2	0.240222	r1454	TCDB:2.A.1.13.5 TCDB:2.A.1.13.1	1.00 * proton [c] + 1.00 * 2-Oxobutanoate [m] --> 1.00 * p	SLC16A1; S	Transport, mitochondrial			0
r1455_pos	0	0.080856	1.0922877	-2.99797	2.997971	2	0.240222	r1455	Transport reaction	1.00 * proton [c] + 1.00 * L-phenylalanine [c] --> 1.00 * pr	1.00 * prot	Transport, mitochondrial			0
r1456_pos	0	0.080856	1.0922877	-2.99797	2.997971	2	0.240222	r1456	Transport reaction	1.00 * proton [c] + 1.00 * L-phenylalanine [m] --> 1.00 * p	1.00 * prot	Transport, mitochondrial			0
r2371_neg	0	0.080856	1.0922877	-1.94736	1.94736	2	0.240222	r2371	Mitochondrial Carrier (MC) TCDB:2.A.29.7.2	1.00 * Citrate [m] + 1.00 * cis-aconitate(3-) [c] --> 1.00 * C	SLC25A1	Transport, mitochondrial			0
r2371_pos	0	0.080856	1.0922877	-1.94736	1.94736	2	0.240222	r2371	Mitochondrial Carrier (MC) TCDB:2.A.29.7.2	1.00 * Citrate [m] + 1.00 * cis-aconitate(3-) [c] --> 1.00 * C	SLC25A1	Transport, mitochondrial			0
r2372_neg	0	0.080856	1.0922877	-1.94736	1.94736	2	0.240222	r2372	Mitochondrial Carrier (MC) TCDB:2.A.29.7.2	1.00 * Isocitrate [m] + 1.00 * cis-aconitate(3-) [c] --> 1.00	SLC25A1	Transport, mitochondrial			0
r2372_pos	0	0.080856	1.0922877	-1.94736	1.94736	2	0.240222	r2372	Mitochondrial Carrier (MC) TCDB:2.A.29.7.2	1.00 * Isocitrate [m] + 1.00 * cis-aconitate(3-) [c] --> 1.00	SLC25A1	Transport, mitochondrial			0
r2373_neg	0	0.080856	1.0922877	-1.94736	1.94736	2	0.240222	r2373	Mitochondrial Carrier (MC) TCDB:2.A.29.7.2	1.00 * 2-Oxoglutarate [c] + 1.00 * Citrate [m] --> 1.00 * 2-	SLC25A1	Transport, mitochondrial			0
r2373_pos	0	0.080856	1.0922877	-1.94736	1.94736	2	0.240222	r2373	Mitochondrial Carrier (MC) TCDB:2.A.29.7.2	1.00 * 2-Oxoglutarate [c] + 1.00 * Citrate [m] --> 1.00 * 2-	SLC25A1	Transport, mitochondrial			0
r2374_neg	0	0.080856	1.0922877	-1.94736	1.94736	2	0.240222	r2374	Mitochondrial Carrier (MC) TCDB:2.A.29.7.2	1.00 * Citrate [m] + 1.00 * oxalate(2-) [c] --> 1.00 * Citrate	SLC25A1	Transport, mitochondrial			0
r2374_pos	0	0.080856	1.0922877	-1.94736	1.94736	2	0.240222	r2374	Mitochondrial Carrier (MC) TCDB:2.A.29.7.2	1.00 * Citrate [m] + 1.00 * oxalate(2-) [c] --> 1.00 * Citrate	SLC25A1	Transport, mitochondrial			0
r2375_neg	0	0.080856	1.0922877	-1.94736	1.94736	2	0.240222	r2375	Mitochondrial Carrier (MC) TCDB:2.A.29.7.2	1.00 * Isocitrate [m] + 1.00 * Succinate [c] --> 1.00 * Isocit	SLC25A1	Transport, mitochondrial			0
r2375_pos	0	0.080856	1.0922877	-1.94736	1.94736	2	0.240222	r2375	Mitochondrial Carrier (MC) TCDB:2.A.29.7.2	1.00 * Isocitrate [m] + 1.00 * Succinate [c] --> 1.00 * Isocit	SLC25A1	Transport, mitochondrial			0
r2376_neg	0	0.080856	1.0922877	-1.94736	1.94736	2	0.240222	r2376	Mitochondrial Carrier (MC) TCDB:2.A.29.7.2	1.00 * Isocitrate [m] + 1.00 * oxalate(2-) [c] --> 1.00 * Isoci	SLC25A1	Transport, mitochondrial			0
r2376_pos	0	0.080856	1.0922877	-1.94736	1.94736	2	0.240222	r2376	Mitochondrial Carrier (MC) TCDB:2.A.29.7.2	1.00 * Isocitrate [m] + 1.00 * oxalate(2-) [c] --> 1.00 * Isoci	SLC25A1	Transport, mitochondrial			0
r2377_neg	0	0.080856	1.0922877	-1.94736	1.94736	2	0.240222	r2377	Mitochondrial Carrier (MC) TCDB:2.A.29.7.2	1.00 * 2-Oxoglutarate [c] + 1.00 * cis-aconitate(3-) [m] -->	SLC25A1	Transport, mitochondrial			0
r2377_pos	0	0.080856	1.0922877	-1.94736	1.94736	2	0.240222	r2377	Mitochondrial Carrier (MC) TCDB:2.A.29.7.2	1.00 * 2-Oxoglutarate [c] + 1.00 * cis-aconitate(3-) [m] -->	SLC25A1	Transport, mitochondrial			0
r2378_neg	0	0.080856	1.0922877	-1.94736	1.94736	2	0.240222	r2378	Mitochondrial Carrier (MC) TCDB:2.A.29.7.2	1.00 * Succinate [c] + 1.00 * cis-aconitate(3-) [m] --> 1.00	SLC25A1	Transport, mitochondrial			0
r2378_pos	0	0.080856	1.0922877	-1.94736	1.94736	2	0.240222	r2378	Mitochondrial Carrier (MC) TCDB:2.A.29.7.2	1.00 * Succinate [c] + 1.00 * cis-aconitate(3-) [m] --> 1.00	SLC25A1	Transport, mitochondrial			0
r2379_neg	0	0.080856	1.0922877	-1.94736	1.94736	2	0.240222	r2379	Mitochondrial Carrier (MC) TCDB:2.A.29.7.2	1.00 * (S)-malate(2-) [c] + 1.00 * cis-aconitate(3-) [m] --> 1.	SLC25A1	Transport, mitochondrial			0
r2379_pos	0	0.080856	1.0922877	-1.94736	1.94736	2	0.240222	r2379	Mitochondrial Carrier (MC) TCDB:2.A.29.7.2	1.00 * (S)-malate(2-) [c] + 1.00 * cis-aconitate(3-) [m] --> 1.	SLC25A1	Transport, mitochondrial			0
r2380_neg	0	0.080856	1.0922877	-1.94736	1.94736	2	0.240222	r2380	Mitochondrial Carrier (MC) TCDB:2.A.29.7.2	1.00 * oxalate(2-) [c] + 1.00 * cis-aconitate(3-) [m] --> 1.00	SLC25A1	Transport, mitochondrial			0
r2380_pos	0	0.080856	1.0922877	-1.94736	1.94736	2	0.240222	r2380	Mitochondrial Carrier (MC) TCDB:2.A.29.7.2	1.00 * oxalate(2-) [c] + 1.00 * cis-aconitate(3-) [m] --> 1.00	SLC25A1	Transport, mitochondrial			0
r2381_pos	0	0.080856	1.0922877	-4.53377	4.533774	2	0.240222	r2381	Mitochondrial Carrier (MC) TCDB:2.A.29.7.2	1.00 * proton [c] + 1.00 * 2-Oxoglutarate [m] + 1.00 * Citr	SLC25A1	Transport, mitochondrial			0
r2382_pos	0	0.080856	1.0922877	-4.53377	4.533774	2	0.240222	r2382	Mitochondrial Carrier (MC) TCDB:2.A.29.7.2	1.00 * proton [c] + 1.00 * Citrate [c] + 1.00 * Succinate [m	SLC25A1	Transport, mitochondrial			0
r2384_pos	0	0.080856	1.0922877	-4.53377	4.533774	2	0.240222	r2384	Mitochondrial Carrier (MC) TCDB:2.A.29.7.2	1.00 * proton [c] + 1.00 * Citrate [c] + 1.00 * oxalate(2-) [n	SLC25A1	Transport, mitochondrial			0
r2385_pos	0	0.080856	1.0922877	-4.53377	4.533774	2	0.240222	r2385	Mitochondrial Carrier (MC) TCDB:2.A.29.7.2	1.00 * proton [c] + 1.00 * 2-Oxoglutarate [m] + 1.00 * Isoc	SLC25A1	Transport, mitochondrial			0
r2386_pos	0	0.080856	1.0922877	-4.53377	4.533774	2	0.240222	r2386	Mitochondrial Carrier (MC) TCDB:2.A.29.7.2	1.00 * proton [c] + 1.00 * Isocitrate [c] + 1.00 * Succinate	SLC25A1	Transport, mitochondrial			0
r2387_pos	0	0.080856	1.0922877	-4.53377	4.533774	2	0.240222	r2387	Mitochondrial Carrier (MC) TCDB:2.A.29.7.2	1.00 * proton [c] + 1.00 * Isocitrate [c] + 1.00 * (S)-malate	SLC25A1	Transport, mitochondrial			0
r2388_pos	0	0.080856	1.0922877	-4.53377	4.533774	2	0.240222	r2388	Mitochondrial Carrier (MC) TCDB:2.A.29.7.2	1.00 * proton [c] + 1.00 * Isocitrate [c] + 1.00 * oxalate(2-	SLC25A1	Transport, mitochondrial			0
r2389_pos	0	0.080856	1.0922877	-4.53377	4.533774	2	0.240222	r2389	Mitochondrial Carrier (MC) TCDB:2.A.29.7.2	1.00 * proton [c] + 1.00 * 2-Oxoglutarate [m] + 1.00 * cis-a	SLC25A1	Transport, mitochondrial			0
r2390_pos	0	0.080856	1.0922877	-4.53377	4.533774	2	0.240222	r2390	Mitochondrial Carrier (MC) TCDB:2.A.29.7.2	1.00 * proton [c] + 1.00 * Succinate [m] + 1.00 * cis-aconit	SLC25A1	Transport, mitochondrial			0
r2391_pos	0	0.080856	1.0922877	-4.53377	4.533774	2	0.240222	r2391	Mitochondrial Carrier (MC) TCDB:2.A.29.7.2	1.00 * proton [c] + 1.00 * (S)-malate(2-) [m] + 1.00 * cis-ac	SLC25A1	Transport, mitochondrial			0
r2392_pos	0	0.080856	1.0922877	-4.53377	4.533774	2	0.240222	r2392	Mitochondrial Carrier (MC) TCDB:2.A.29.7.2	1.00 * proton [c] + 1.00 * cis-aconitate(3-) [c] + 1.00 * oxa	SLC25A1	Transport, mitochondrial			0
r2393_pos	0	0.080856	1.0922877	-1.94736	1.94736	2	0.240222	r2393	Mitochondrial Carrier (MC) TCDB:2.A.29.7.2	1.00 * Phosphoenolpyruvate [c] + 1.00 * Isocitrate [m] -->	SLC25A1	Transport, mitochondrial			0
r2394_pos	0	0.080856	1.0922877	-1.94736	1.94736	2	0.240222	r2394	Mitochondrial Carrier (MC) TCDB:2.A.29.7.2	1.00 * Phosphoenolpyruvate [c] + 1.00 * cis-aconitate(3-)	SLC25A1	Transport, mitochondrial			0
r2395_pos	0	0.080856	1.0922877	-2.57949	2.579493	2	0.240222	r2395	Mitochondrial Carrier (MC) TCDB:2.A.29.19.1	1.00 * proton [c] + 1.00 * L-lysinium(1+) [m] + 1.00 * L-arg	SLC25A2	Transport, mitochondrial			0
r2396_pos	0	0.080856	1.0922877	-2.57949	2.579493	2	0.240222	r2396	Mitochondrial Carrier (MC) TCDB:2.A.29.19.1	1.00 * proton [c] + 1.00 * Ornithine [c] + 1.00 * L-lysinium	SLC25A2	Transport, mitochondrial			0
r2397_pos	0	0.080856	1.0922877	-2.68446	2.684464	2	0.240222	r2397	Mitochondrial Carrier (MC) TCDB:2.A.29.19.1	1.00 * proton [c] + 1.00 * L-lysinium(1+) [m] + 1.00 * L-his	SLC25A2	Transport, mitochondrial			0
r2398_pos	0	0.080856	1.0922877	-2.57506	2.575055	2	0.240222	r2398	Mitochondrial Carrier (MC) TCDB:2.A.29.19.1	1.00 * proton [c] + 1.00 * L-lysinium(1+) [m] + 1.00 * L-cit	SLC25A2	Transport, mitochondrial			0
r2399_pos	0	0.080856	1.0922877	-2.57949	2.579493	2	0.240222	r2399	Mitochondrial Carrier (MC) TCDB:2.A.29.19.1	1.00 * proton [c] + 1.00 * L-lysinium(1+) [c] + 1.00 * L-argi	SLC25A2	Transport, mitochondrial			0
r2400_pos	0	0.080856	1.0922877	-2.57949	2.579493	2	0.240222	r2400	Mitochondrial Carrier (MC) TCDB:2.A.29.19.1	1.00 * proton [c] + 1.00 * Ornithine [c] + 1.00 * L-argininiu	SLC25A2	Transport, mitochondrial			0
r2401_pos	0	0.080856	1.0922877	-2.68446	2.684464	2	0.240222	r2401	Mitochondrial Carrier (MC) TCDB:2.A.29.19.1	1.00 * proton [c] + 1.00 * L-argininium(1+) [m] + 1.00 * L-	SLC25A2	Transport, mitochondrial			0
r2402_pos	0	0.080856	1.0922877	-2.57506	2.575055	2	0.240222	r2402	Mitochondrial Carrier (MC) TCDB:2.A.29.19.1	1.00 * proton [c] + 1.00 * L-argininium(1+) [m] + 1.00 * L-	SLC25A2	Transport, mitochondrial			0
r2403_pos	0	0.080856	1.0922877	-2.42231	2.422306	2	0.240222	r2403	Mitochondrial Carrier (MC) TCDB:2.A.29.19.1	1.00 * proton [c] + 1.00 * L-lysinium(1+) [c] + 1.00 * Ornit	SLC25A2	Transport, mitochondrial			0
r2404_pos	0	0.080856	1.0922877	-2.42231	2.422306	2	0.240222	r2404	Mitochondrial Carrier (MC) TCDB:2.A.29.19.1	1.00 * proton [c] + 1.00 * L-argininium(1+) [c] + 1.00 * Orn	SLC25A2	Transport, mitochondrial			0
r2405_pos	0	0.080856	1.0922877	-2.42231	2.422306	2	0.240222	r2405	Mitochondrial Carrier (MC) TCDB:2.A.29.19.1	1.00 * proton [c] + 1.00 * Ornithine [m] + 1.00 * L-histidin	SLC25A2	Transport, mitochondrial			0
r2406_pos	0	0.080856	1.0922877	-2.42231	2.422306	2	0.240222	r2406	Mitochondrial Carrier (MC) TCDB:2.A.29.19.1	1.00 * proton [c] + 1.00 * L-lysinium(1+) [c] + 1.00 * L-hist	SLC25A2	Transport, mitochondrial			0
r2407_pos	0	0.080856	1.0922877	-2.42231	2.422306	2	0.240222	r2407	Mitochondrial Carrier (MC) TCDB:2.A.29.19.1	1.00 * proton [c] + 1.00 * L-argininium(1+) [c] + 1.00 * L-h	SLC25A2	Transport, mitochondrial			0
r2408_pos	0	0.080856	1.0922877	-2.67832	2.678317	2	0.240222	r2408	Mitochondrial Carrier (MC) TCDB:2.A.29.19.1	1.00 * proton [c] + 1.00 * Ornithine [c] + 1.00 * L-histidine	SLC25A2	Transport, mitochondrial			0
r2409_pos	0	0.080856	1.0922877	-2.64598	2.645984	2	0.240222	r2409	Mitochondrial Carrier (MC) TCDB:2.A.29.19.1	1.00 * proton [c] + 1.00 * L-citrulline [c] + 1.00 * L-histidin	SLC25A2	Transport, mitochondrial			0
r2410_pos	0	0.080856	1.0922877	-2.41771	2.417712	2	0.240222	r2410	Mitochondrial Carrier (MC) TCDB:2.A.29.19.1	1.00 * proton [c] + 1.00 * L-lysinium(1+) [c] + 1.00 * L-citr	SLC25A2	Transport, mitochondrial			0
r2411_pos	0	0.080856	1.0922877	-2.41771	2.417712	2	0.240222	r2411	Mitochondrial Carrier (MC) TCDB:2.A.29.19.1	1.00 * proton [c] + 1.00 * L-argininium(1+) [c] + 1.00 * L-c	SLC25A2	Transport, mitochondrial			0
r2413_pos	0	0.080856	1.0922877	-2.42231	2.422306	2	0.240222	r2413	Mitochondrial Carrier (MC) TCDB:2.A.29.19.1	1.00 * proton [c] + 1.00 * L-citrulline [m] + 1.00 * L-histid	SLC25A2	Transport, mitochondrial			0
r2416_pos	0	0.080856	1.0922877	-2.68446	2.684464	2	0.240222	r2416	Mitochondrial Carrier (MC) TCDB:2.A.29.19.1	1.00 * proton [c] + 1.00 * L-histidine [m] --> 1.00 * proton	SLC25A2	Transport, mitochondrial			0
r2420_neg	0	0.080856	1.0922877	-2.27787	2.277875	2	0.240222	r2420	Mitochondrial Carrier (MC) TCDB:2.A.29.2.7	1.00 * hydrogenphosphate [m] + 1.00 * oxalate(2-) [c] -->	SLC25A10	Transport, mitochondrial			0

r2420_pos	0	0.080856	1.0922877	-2.27787	2.277875	2	0.240222	r2420	Mitochondrial Carrier (MC) TCDB:2.A.29.2.7	1.00 * hydrogenphosphate [m] + 1.00 * oxalate(2-) [c] -->	SLC25A10	Transport, mitochondrial			0
MALtm_neg	1	0.19043	0.7202646	-1.51471	1.51471	2	0.347218	MALtm	malate transport, mitochondrial	1.00 * hydrogenphosphate [m] + 1.00 * (S)-malate(2-) [c] -	SLC25A10	Transport, mitochondrial			4
ACETONEt2m_pos	1	0.19043	0.7202646	-1.89059	1.890593	2	0.347218	ACETONEt2	acetone mitochondrial transport via proton symport	1.00 * proton [c] + 1.00 * acetone [c] --> 1.00 * proton [m]	SLC16A1	Transport, mitochondrial			0
L_LACtm_neg	1	0.19043	0.7202646	-1.91888	1.918884	2	0.347218	L_LACtm	L-lactate transport, mitochondrial	1.00 * proton [c] + 1.00 * (S)-lactate [c] --> 1.00 * proton [	SLC16A1	Transport, mitochondrial			0
r0801_neg	8	0.19043	0.7202646	1.201062	1.201062	1	0.347218	r0801	Mitochondrial Carrier (MC) TCDB:2.A.29.21.1	1.00 * GTP [c] + 1.00 * GDP [m] --> 1.00 * GDP [c] + 1.00 *	1.00 * GTP	Transport, mitochondrial			0
r0885_neg	1	0.19043	0.7202646	-1.39467	1.394674	2	0.347218	r0885	Mitochondrial Carrier (MC) TCDB:2.A.29.2.7	1.00 * hydrogenphosphate [m] + 1.00 * Reduced glutathi	SLC25A10	Transport, mitochondrial			0
r0885_pos	1	0.19043	0.7202646	-1.23647	1.236474	2	0.347218	r0885	Mitochondrial Carrier (MC) TCDB:2.A.29.2.7	1.00 * hydrogenphosphate [m] + 1.00 * Reduced glutathi	SLC25A10	Transport, mitochondrial			0
r0927_pos	1	0.19043	0.7202646	-1.89059	1.890593	2	0.347218	r0927	Free diffusion	1.00 * acetone [m] --> 1.00 * acetone [e]No genes	1.00 * acet	Transport, mitochondrial			0
r2419_pos	1	0.19043	0.7202646	-1.51471	1.51471	2	0.347218	r2419	Mitochondrial Carrier (MC) TCDB:2.A.29.2.7	1.00 * 2-Oxoglutarate [c] + 1.00 * hydrogenphosphate [m	SLC25A10	Transport, mitochondrial			0
r2520_neg	1	0.19043	0.7202646	-1.23647	1.236474	2	0.347218	r2520	Mitochondrial Carrier (MC) TCDB:2.A.29.2.1	1.00 * 2-Oxoglutarate [m] + 1.00 * Reduced glutathione [	SLC25A11	Transport, mitochondrial			0
r2520_pos	1	0.19043	0.7202646	-1.39467	1.394674	2	0.347218	r2520	Mitochondrial Carrier (MC) TCDB:2.A.29.2.1	1.00 * 2-Oxoglutarate [m] + 1.00 * Reduced glutathione [	SLC25A11	Transport, mitochondrial			0
3MOBt2im_pos	7	0.382733	0.4171041	0.681567	0.681567	1	0.518179	3MOBt2im	3-methyl-2-oxobutanoate mitochondrial transport via proton symp	1.00 * proton [c] + 1.00 * 3-methyl-2-oxobutanoate [c] -->	1.00 * prot	Transport, mitochondrial			4
3MOPt2im_pos	7	0.382733	0.4171041	0.681567	0.681567	1	0.518179	3MOPt2im	3-Methyl-2-oxopentanoate mitochondrial transport via proton sym	1.00 * proton [c] + 1.00 * 3-methyl-2-oxopentanoate [c] -	1.00 * prot	Transport, mitochondrial			4
4MOPt2im_pos	7	0.382733	0.4171041	0.681567	0.681567	1	0.518179	4MOPt2im	4-methyl-2-oxopentanoate mitochondrial transportvia proton sym	1.00 * proton [c] + 1.00 * 4-methyl-2-oxopentanoate [c] -	1.00 * prot	Transport, mitochondrial			4
ATPtm_pos	7	0.382733	0.4171041	1.240929	1.240929	1	0.518179	ATPtm	ADP/ATP transporter, mitochondrial	1.00 * ADP [c] + 1.00 * ATP [m] --> 1.00 * ATP [c] + 1.00 * A	SLC25A4; S	Transport, mitochondrial			4
C4tmc_pos	7	0.382733	0.4171041	0.83477	0.83477	1	0.518179	C4tmc	transport of bytyrylcarnitine into mitochondrial matrix	1.00 * butyryl carnitine [c] --> 1.00 * butyryl carnitine [m]	SLC25A20	Transport, mitochondrial			4
DNDPt22m_pos	7	0.382733	0.4171041	1.243093	1.243093	1	0.518179	DNDPt22m	dTDP transport via dUDP antiport	1.00 * dTDP [c] + 1.00 * dUDP [m] --> 1.00 * dTDP [m] + 1.0	SLC25A19	Transport, mitochondrial			4
DNDPt26m_pos	7	0.382733	0.4171041	1.243093	1.243093	1	0.518179	DNDPt26m	dCDP transport via dUDP antiport	1.00 * dCDP [c] + 1.00 * dUDP [m] --> 1.00 * dCDP [m] + 1.	SLC25A19	Transport, mitochondrial			4
DNDPt27m_pos	7	0.382733	0.4171041	1.243483	1.243483	1	0.518179	DNDPt27m	dCDP transport via dTDP antiport	1.00 * dCDP [c] + 1.00 * dTDP [m] --> 1.00 * dCDP [m] + 1.0	SLC25A19	Transport, mitochondrial			4
DNDPt33m_pos	7	0.382733	0.4171041	1.243093	1.243093	1	0.518179	DNDPt33m	dGDP transport via dUDP antiport	1.00 * dGDP [c] + 1.00 * dUDP [m] --> 1.00 * dGDP [m] + 1.	SLC25A19	Transport, mitochondrial			4
DNDPt34m_pos	7	0.382733	0.4171041	1.243483	1.243483	1	0.518179	DNDPt34m	dGDP transport via dTDP antiport	1.00 * dGDP [c] + 1.00 * dTDP [m] --> 1.00 * dGDP [m] + 1.	SLC25A19	Transport, mitochondrial			4
DNDPt36m_pos	7	0.382733	0.4171041	1.243483	1.243483	1	0.518179	DNDPt36m	dGDP transport via dCDP antiport	1.00 * dGDP [c] + 1.00 * dCDP [m] --> 1.00 * dCDP [c] + 1.0	SLC25A19	Transport, mitochondrial			4
DNDPt37m_pos	7	0.382733	0.4171041	1.243093	1.243093	1	0.518179	DNDPt37m	dUTP transport via dTDP antiport	1.00 * dTDP [m] + 1.00 * dUTP [c] --> 1.00 * dTDP [c] + 1.00	SLC25A19	Transport, mitochondrial			4
DNDPt38m_pos	7	0.382733	0.4171041	1.240929	1.240929	1	0.518179	DNDPt38m	dUTP transport via dUDP antiport	1.00 * dUDP [m] + 1.00 * dUTP [c] --> 1.00 * dUDP [c] + 1.0	SLC25A19	Transport, mitochondrial			4
DNDPt39m_pos	7	0.382733	0.4171041	1.243093	1.243093	1	0.518179	DNDPt39m	dUTP transport via dGDP antiport	1.00 * dGDP [m] + 1.00 * dUTP [c] --> 1.00 * dGDP [c] + 1.0	SLC25A19	Transport, mitochondrial			4
DNDPt41m_pos	7	0.382733	0.4171041	1.243093	1.243093	1	0.518179	DNDPt41m	dUTP transport via dCDP antiport	1.00 * dCDP [m] + 1.00 * dUTP [c] --> 1.00 * dCDP [c] + 1.0	SLC25A19	Transport, mitochondrial			4
ILEt5m_neg	7	0.382733	0.4171041	0.681567	0.681567	1	0.518179	ILEt5m	Isoleucine mitochondrial transport	1.00 * L-isoleucine [c] --> 1.00 * L-isoleucine [m]No genes	1.00 * L-iso	Transport, mitochondrial			4
LEUt5m_neg	7	0.382733	0.4171041	0.681567	0.681567	1	0.518179	LEUt5m	leucine mitochondrial transport	1.00 * L-leucine [c] --> 1.00 * L-leucine [m]No genes	1.00 * L-leu	Transport, mitochondrial			4
VALt5m_neg	7	0.382733	0.4171041	0.681567	0.681567	1	0.518179	VALt5m	Valine reversible mitochondrial transport	1.00 * L-valine [c] --> 1.00 * L-valine [m]No genes	1.00 * L-val	Transport, mitochondrial			4
r0801_pos	2	0.382733	0.4171041	-0.86031	0.860315	2	0.518179	r0801	Mitochondrial Carrier (MC) TCDB:2.A.29.21.1	1.00 * GTP [c] + 1.00 * GDP [m] --> 1.00 * GDP [c] + 1.00 *	1.00 * GTP	Transport, mitochondrial			0
r2436_pos	7	0.382733	0.4171041	0.918523	0.918523	1	0.518179	r2436	Mitochondrial Carrier (MC) TCDB:2.A.29.8.3	1.00 * L-Carnitine [m] + 1.00 * L-Oleoylcarnitine [c] --> 1.0	SLC25A20	Transport, mitochondrial			0
C4tmc_neg	6	0.662521	0.1788004	0.487932	0.487932	1	0.758498	C4tmc	transport of bytyrylcarnitine into mitochondrial matrix	1.00 * butyryl carnitine [c] --> 1.00 * butyryl carnitine [m]	SLC25A20	Transport, mitochondrial			4
COAtm_pos	6	0.662521	0.1788004	0.518912	0.518912	1	0.758498	COAtm	CoA transporter	1.00 * Coenzyme A [c] --> 1.00 * Coenzyme A [m]SLC25A1	SLC25A16	Transport, mitochondrial			4
CRNtim_pos	6	0.662521	0.1788004	0.518912	0.518912	1	0.758498	CRNtim	L-carnitine transport out of mitochondria via diffusion	1.00 * L-Carnitine [m] --> 1.00 * L-Carnitine [c]SLC25A20	SLC25A20	Transport, mitochondrial			4
H2Otm_neg	3	0.662521	0.1788004	0.706368	0.706368	1	0.758498	H2Otm	H2O transport, mitochondrial	1.00 * Water [c] --> 1.00 * Water [m]AQP8	AQP8	Transport, mitochondrial			4
H2Otm_pos	3	0.662521	0.1788004	0.706368	0.706368	1	0.758498	H2Otm	H2O transport, mitochondrial	1.00 * Water [c] --> 1.00 * Water [m]AQP8	AQP8	Transport, mitochondrial			4
PIt2m_pos	6	0.662521	0.1788004	0.677144	0.677144	1	0.758498	PIt2m	phosphate transporter, mitochondrial	1.00 * proton [c] + 1.00 * hydrogenphosphate [c] --> 1.00	SLC25A3	Transport, mitochondrial			4
GLYCtm_pos	6	0.662521	0.1788004	0.739151	0.739151	1	0.758498	GLYCtm	glycerol transport	1.00 * Glycerol [c] --> 1.00 * Glycerol [m]No genes	1.00 * Glyc	Transport, mitochondrial			0
r0973_neg	3	0.662521	0.1788004	-0.27302	0.273018	2	0.758498	r0973	Facilitated diffusion	1.00 * pantetheine [m] --> 1.00 * pantetheine [c]No genes	1.00 * pant	Transport, mitochondrial			0
r1291_neg	6	0.662521	0.1788004	0.74055	0.74055	1	0.758498	r1291	Postulated transport reaction	1.00 * Propanoyl-CoA [c] --> 1.00 * Propanoyl-CoA [m]No	1.00 * Prop	Transport, mitochondrial		6.2.1.3	0
r2437_pos	6	0.662521	0.1788004	0.74055	0.74055	1	0.758498	r2437	Mitochondrial Carrier (MC) TCDB:2.A.29.8.3	1.00 * L-Carnitine [m] + 1.00 * O-Propanoylcarnitine [c] --	SLC25A20	Transport, mitochondrial			0
r2438_pos	6	0.662521	0.1788004	0.487932	0.487932	1	0.758498	r2438	Mitochondrial Carrier (MC) TCDB:2.A.29.8.3	1.00 * L-Carnitine [m] + 1.00 * O-Butanoylcarnitine [c] -->	SLC25A20	Transport, mitochondrial			0
r2472_pos	6	0.662521	0.1788004	0.677144	0.677144	1	0.758498	r2472	Major Facilitator(MFS) TCDB:2.A.1.4.7	1.00 * hydrogenphosphate [m] + 1.00 * Glycerol 3-phosp	SLC37A1	Transport, mitochondrial			0
r0917_neg	4	1	0	-0.91613	0.916131	2	1	r0917	Mitochondrial Carrier (MC) TCDB:2.A.29.7.2	1.00 * Citrate [c] + 1.00 * Isocitrate [m] --> 1.00 * Isocitrat	SLC25A1	Transport, mitochondrial			0
r0917_pos	4	1	0	-0.91613	0.916131	2	1	r0917	Mitochondrial Carrier (MC) TCDB:2.A.29.7.2	1.00 * Citrate [c] + 1.00 * Isocitrate [m] --> 1.00 * Isocitrat	SLC25A1	Transport, mitochondrial			0
r1001_pos	5	1	0	0.333815	0.333815	1	1	r1001	Facilitated diffusion	1.00 * L-Carnitine [c] + 1.00 * palmitoleoyl-Carnitine [m]	1.00 * L-Ca	Transport, mitochondrial			0
NADHtpu_pos	9	0.080856	1.0922877	5.692481	5.692481	1	0.240222	NADHtpu	NADH transporter, peroxisome	1.00 * Nicotinamide adenine dinucleotide - reduced [c] --	1.00 * Nico	Transport, peroxisomal			0
r1147_neg	0	0.080856	1.0922877	-1.94736	1.94736	2	0.240222	r1147	Mitochondrial Carrier (MC) TCDB:2.A.29.7.2	1.00 * 2-Oxoglutarate [c] + 1.00 * Isocitrate [m] --> 1.00 *	SLC25A1	Transport, peroxisomal			0
r1147_pos	0	0.080856	1.0922877	-1.94736	1.94736	2	0.240222	r1147	Mitochondrial Carrier (MC) TCDB:2.A.29.7.2	1.00 * 2-Oxoglutarate [c] + 1.00 * Isocitrate [m] --> 1.00 *	SLC25A1	Transport, peroxisomal			0
Htx_neg	2	0.382733	0.4171041	-1.6311	1.631095	2	0.518179	Htx	H transporter, peroxisome	1.00 * proton [c] --> 1.00 * proton [x]No genes	1.00 * prot	Transport, peroxisomal			0
Htx_pos	7	0.382733	0.4171041	1.051667	1.051667	1	0.518179	Htx	H transporter, peroxisome	1.00 * proton [c] --> 1.00 * proton [x]No genes	1.00 * prot	Transport, peroxisomal			0
PRISTtx_neg	7	0.382733	0.4171041	0.858628	0.858628	1	0.518179	PRISTtx	prist peroxisomal transport	1.00 * pristanic acid [x] --> 1.00 * pristanic acid [c]No gen	1.00 * prist	Transport, peroxisomal			0
r0934_pos	7	0.382733	0.4171041	1.035927	1.035927	1	0.518179	r0934	Neurotransmitter:Sodium Symporter (NSS) TCDB:2.A.22.3.3	1.00 * Taurine [c] --> 1.00 * Taurine [x]SLC6A6	SLC6A6	Transport, peroxisomal			0
r1019_neg	2	0.382733	0.4171041	-1.30963	1.309628	2	0.518179	r1019	Postulated transport reaction	1.00 * chenodeoxycholoyl coenzyme a [x] --> 1.00 * chen	1.00 * chen	Transport, peroxisomal			0
r1019_pos	2	0.382733	0.4171041	-1.30965	1.309646	2	0.518179	r1019	Postulated transport reaction	1.00 * chenodeoxycholoyl coenzyme a [x] --> 1.00 * chen	1.00 * chen	Transport, peroxisomal			0
r2516_neg	2	0.382733	0.4171041	-1.6311	1.631095	2	0.518179	r2516	Major Facilitator(MFS) TCDB:2.A.1.13.1	1.00 * proton [x] + 1.00 * (S)-lactate [x] --> 1.00 * proton [	SLC16A1	Transport, peroxisomal			0
r2516_pos	7	0.382733	0.4171041	1.051667	1.051667	1	0.518179	r2516	Major Facilitator(MFS) TCDB:2.A.1.13.1	1.00 * proton [x] + 1.00 * (S)-lactate [x] --> 1.00 * proton [	SLC16A1	Transport, peroxisomal			0
C2tcx_neg	6	0.662521	0.1788004	0.310264	0.310264	1	0.758498	C2tcx	transport of acetylcarnitine from peroxisomes to cytosol	1.00 * O-acetylcarnitine [x] --> 1.00 * O-acetylcarnitine [c]	SLC25A20	Transport, peroxisomal			4
SCP22x_neg	3	0.662521	0.1788004	-0.29731	0.29731	2	0.758498	SCP22x	Sterol carrier protein 2	1.00 * 4,8 dimethylnonanoyl-CoA [c] --> 1.00 * 4,8 dimet	SCP2	Transport, peroxisomal			4
CRNCARtp_pos	6	0.662521	0.1788004	0.310264	0.310264	1	0.758498	CRNCARtp	carnitine-acetylcarnitine carrier, peroxisomal	1.00 * L-Carnitine [c] + 1.00 * O-acetylcarnitine [x] --> 1.00	CRAT	Transport, peroxisomal			0
r0990_neg	6	0.662521	0.1788004	0.939309	0.939309	1	0.758498	r0990	Postulated transport reaction	1.00 * choloyl-CoA(4-) [x] --> 1.00 * choloyl-CoA(4-) [c]No	1.00 * chol	Transport, peroxisomal			0
r0997_pos	6	0.662521	0.1788004	0.224924	0.224924	1	0.758498	r0997	COT Facilitated diffusion	1.00 * L-Carnitine [c] + 1.00 * L-Octanoylcarnitine [x] --> 1	CROT	Transport, peroxisomal			0
C9BRxtc_neg	5	1	0	0.335028	0.335028	1	1	C9BRxtc	transport of dimethylnonanoylcarnitine from peroxisomes to cytos	1.00 * 4,8-dimethylnonanoyl carnitine [x] --> 1.00 * 4,8-	SLC25A20	Transport, peroxisomal			4
C9BRxtc_pos	5	1	0	0.335028	0.335028	1	1	C9BRxtc	transport of dimethylnonanoylcarnitine from peroxisomes to cytos	1.00 * 4,8-dimethylnonanoyl carnitine [x] --> 1.00 * 4,8-	SLC25A20	Transport, peroxisomal			4
SCP22x_pos	4	1	0	-0.07871	0.078708	2	1	SCP22x	Sterol carrier protein 2	1.00 * 4,8 dimethylnonanoyl-CoA [c] --> 1.00 * 4,8 dimet	SCP2	Transport, peroxisomal			4
CRNCAR3tp_pos	4	1	0	-0.11711	0.117112	2	1	CRNCAR3tp	carnitine-propcarnitine carrier, peroxisomal	1.00 * L-Carnitine [c] + 1.00 * propionyl-carnitine [x] --> 1	1.00 * L-Ca	Transport, peroxisomal			0
r1292_pos	4	1	0	-0.11711	0.117112	2	1	r1292	Postulated transport reaction	1.00 * Propanoyl-CoA [c] --> 1.00 * Propanoyl-CoA [x]No g	1.00 * Prop	Transport, peroxisomal		6.2.1.3	0
2OXOADPTm_neg	3	0.662521	0.1788004	-0.85086	0.850863	2	0.758498	2OXOADPT	2-oxoadipate shuttle (cytosol/mitochondria)	1.00 * 2-Oxoglutarate [m] + 1.00 * 2-oxoadipate(2-) [c] -->	SLC25A21	Tryptophan metabolism	31		4
KYNAKGAT_pos	3	0.662521	0.1788004	-0.65341	0.653409	2	0.758498	KYNAKGAT	L-Kynurenine:2-oxoglutarate aminotransferase	1.00 * 2-Oxoglutarate [c] + 1.00 * L-kynurenine [c] --> 1.00	AADAT; KY	Tryptophan metabolism	31	2.6.1.7	4
KYNATESYN_pos	3	0.662521	0.1788004	-0.65341	0.653409	2	0.758498	KYNATESYN	4-(2-Aminophenyl)-2,4-dioxobutanoate dehydratase	1.00 * 4-(2-aminophenyl)-2,4-dioxobutanoate [c] --> 1.00	1.00 * 4-(2-	Tryptophan metabolism	31		4
TRPHYDRO2_pos	6	0.662521	0.1788004	0.007222	0.007222	1	0.758498	TRPHYDRO	L-Tryptophan,tetrahydrobiopterin:oxygen oxidoreductase (5-hydro	1.00 * O2 [c] + 1.00 * 5,6,7,8-tetrahydrobiopterin [c] + 1.	TPH1; TPH2	Tryptophan metabolism	31	1.14.16.4	4
RE1233C_neg	3	0.662521	0.1788004	-0.65341	0.653409	2	0.758498	RE1233C	RE1233	1.00 * 2-Oxoglutarate [c] + 1.00 * L-kynurenine [c] --> 1.00	KYAT1	Tryptophan metabolism	31	2.6.1.7	0
RE2349C_neg	3	0.662521	0.1788004	-0.65341	0.653409	2	0.758498	RE2349C	RE2349	1.00 * 2-Oxoglutarate [c] + 1.00 * 3-hydroxy-L-kynurenin	KYAT1	Tryptophan metabolism	31	2.6.1.7	0
r0647_neg	3	0.662521	0.1788004	-0.65341	0.653409	2	0.758498	r0647	Spontaneous reaction	1.00 * Water [c] + 1.00 * Xanthurenic acid [c] --> 1.00 * 4-	1.00 * Wat	Tryptophan metabolism	31		0
FALDH_neg	1	0.19043	0.7202646	-1.28682	1.286817	2	0.347218	FALDH	formaldehyde dehydrogenase	1.00 * Nicotinamide adenine dinucleotide [c] + 1.00 * for	ADH5	Tyrosine metabolism	32	1.2.1.1	4
FALDH_pos	1	0.19043	0.7202646	-1.28682	1.286817	2	0.347218	FALDH	formaldehyde dehydrogenase	1.00 * Nicotinamide adenine dinucleotide [c] + 1.00 * for	ADH5	Tyrosine metabolism	32	1.2.1.1	4
r0083_pos	9	0.080856	1.0922877	2.461852	2.461852	1	0.240222	r0083	Oxalosuccinate:NADP+ oxidoreductase (decarboxylating) Citrate cyc	1.00 * proton [m] + 1.00 * oxalatosuccinate(3-) [m] --> 1.0	IDH1; IDH2	Unassigned		1.1.1.42	0
r0267_pos	0	0.080856	1.0922877	-3.30002	3.300024	2	0.240222	r0267	CMP-N-acetylneuraminate,ferrocytochrome-b5:oxygen oxidoreduc	1.00 * O2 [c] + 1.00 * proton [c] + 1.00 * Nicotinamide ad	1.00 * O2 [	Unassigned		1.14.18.2	0
r0268_neg	0	0.080856	1.0922877	-3.30002	3.300024	2	0.240222	r0268	cytidine monophospho-N-acetylneuraminic acid hydroxylaseEC:1.1	1.00 * O2 [c] + 1.00 * proton [c] + 1.00 * Nicotinamide ad	1.00 * O2 [	Unassigned		1.14.18.2	0
r0423_pos	9	0.080856	1.0922877	2.461852	2.461852	1	0.240222	r0423	Isocitrate:NADP+ oxidoreductase (decarboxylating) Citrate cycle (TC	1.00 * Nicotinamide adenine dinucleotide phosphate [m]	IDH1; IDH2	Unassigned		1.1.1.42	0
r0752_neg	0	0.080856	1.0922877	-1.96346	1.963461	2	0.240222	r0752	3,4-Dihydroxymandelaldehyde:NAD+ oxidoreductase Tyrosine meta	1.00 * Water [m] + 1.00 * Nicotinamide adenine dinucleo	ALDH1A3;	Unassigned		1.2.1.5	0
r0753_pos	0	0.080856	1.0922877	-1.96346	1.963461	2	0.240222	r0753	3,4-Dihydroxymandelaldehyde:NADP+ oxidoreductase Tyrosine met	1.00 * Water [m] + 1.00 * Nicotinamide adenine dinucleo	ALDH1A3;	Unassigned		1.2.1.5	0
r0754_neg	0	0.080856	1.0922877	-1.96346	1.963461	2	0.240222	r0754	3-Methoxy-4-hydroxyphenylacetaldehyde:NAD+ oxidoreductase Tyr	1.00 * Water [m] + 1.00 * Nicotinamide adenine dinucleo	ALDH1A3;	Unassigned		1.2.1.5	0

r0755_pos	0	0.080856	1.0922877	-1.96346	1.963461	2	0.240222	r0755	3-Methoxy-4-hydroxyphenylacetaldehyde:NADP+ oxidoreductase T	1.00 * Water [m] + 1.00 * Nicotinamide adenine dinucleo	ALDH1A3;	Unassigned		1.2.1.5	0
r0756_neg	0	0.080856	1.0922877	-1.96346	1.963461	2	0.240222	r0756	3-Methoxy-4-hydroxyphenylglycolaldehyde:NAD+ oxidoreductase T	1.00 * Water [m] + 1.00 * Nicotinamide adenine dinucleo	ALDH1A3;	Unassigned		1.2.1.5	0
r0757_pos	0	0.080856	1.0922877	-1.96346	1.963461	2	0.240222	r0757	3-Methoxy-4-hydroxyphenylglycolaldehyde:NADP+ oxidoreductase	1.00 * Water [m] + 1.00 * Nicotinamide adenine dinucleo	ALDH1A3;	Unassigned		1.2.1.5	0
r1418_pos	0	0.080856	1.0922877	-1.79567	1.795669	2	0.240222	r1418	Carbonic acid hydro-lyase Nitrogen metabolism EC:4.2.1.1	1.00 * proton [e] + 1.00 * Bicarbonate [e] --> 1.00 * Water	CAR1; CAR	Unassigned		4.2.1.1	0
r0407_neg	1	0.19043	0.7202646	-1.46042	1.460419	2	0.347218	r0407	Sedoheptulose 1,7-bisphosphate D-glyceraldehyde-3-phosphate-lya	1.00 * sedoheptulose 1,7-bisphosphate(4-) [c] --> 1.00 * D	ALDOART2;	Unassigned		4.1.2.13	0
r0408_neg	1	0.19043	0.7202646	-1.46042	1.460419	2	0.347218	r0408	ATP:Sedoheptulose 7-phosphate 1-phosphotransferase EC:2.7.1.11	1.00 * ATP [c] + 1.00 * Sedoheptulose 7-phosphate [c] -->	PFKL	Unassigned		2.7.1.11	0
r0425_pos	8	0.19043	0.7202646	1.387465	1.387465	1	0.347218	r0425	EC:1.1.1.42	1.00 * Nicotinamide adenine dinucleotide [m] + 1.00 * Is	IDH1; IDH2	Unassigned		1.1.1.42	0
r1377_neg	1	0.19043	0.7202646	-1.28682	1.286817	2	0.347218	r1377	S-(hydroxymethyl)glutathione synthase Methane metabolism EC:4.4	1.00 * formaldehyde [c] + 1.00 * Reduced glutathione [c]	1.00 * form	Unassigned		4.4.1.22	0
r1377_pos	1	0.19043	0.7202646	-1.28682	1.286817	2	0.347218	r1377	S-(hydroxymethyl)glutathione synthase Methane metabolism EC:4.4	1.00 * formaldehyde [c] + 1.00 * Reduced glutathione [c]	1.00 * form	Unassigned		4.4.1.22	0
r1378_neg	1	0.19043	0.7202646	-1.28682	1.286817	2	0.347218	r1378	S-(hydroxymethyl)glutathione dehydrogenase Methane metabolism	1.00 * Nicotinamide adenine dinucleotide [c] + 1.00 * S-(	ADH5	Unassigned		1.1.1.284	0
r1378_pos	1	0.19043	0.7202646	-1.28682	1.286817	2	0.347218	r1378	S-(hydroxymethyl)glutathione dehydrogenase Methane metabolism	1.00 * Nicotinamide adenine dinucleotide [c] + 1.00 * S-(	ADH5	Unassigned		1.1.1.284	0
r1418_neg	1	0.19043	0.7202646	-1.43187	1.431873	2	0.347218	r1418	Carbonic acid hydro-lyase Nitrogen metabolism EC:4.2.1.1	1.00 * proton [e] + 1.00 * Bicarbonate [e] --> 1.00 * Water	CAR1; CAR	Unassigned		4.2.1.1	0
r0392_neg	7	0.382733	0.4171041	0.954814	0.954814	1	0.518179	r0392	D-Glyceraldehyde:NAD+ oxidoreductase Glycerolipid metabolism E	1.00 * Water [c] + 1.00 * Nicotinamide adenine dinucleot	ALDH1B1;	Unassigned		1.2.1.3	0
r0196_neg	3	0.662521	0.1788004	-0.91402	0.914024	2	0.758498	r0196	Succinyl-CoA:glycine C-succinyl-transferase(decarboxylating) EC:2.3	1.00 * Glycine [m] + 1.00 * Succinyl-CoA [m] --> 1.00 * Co	ALAS1; ALA	Unassigned		2.3.1.37	0
r0267_neg	3	0.662521	0.1788004	-0.60479	0.604793	2	0.758498	r0267	CMP-N-acetylneuraminate,ferrocytochrome-b5:oxygen oxidoreduc	1.00 * O2 [c] + 1.00 * proton [c] + 1.00 * Nicotinamide ad	1.00 * O2 [	Unassigned		1.14.18.2	0
r0268_pos	3	0.662521	0.1788004	-0.60479	0.604793	2	0.758498	r0268	cytidine monophospho-N-acetylneuraminic acid hydroxylaseEC:1.1	1.00 * O2 [c] + 1.00 * proton [c] + 1.00 * Nicotinamide ad	1.00 * O2 [	Unassigned		1.14.18.2	0
r0408_pos	3	0.662521	0.1788004	-0.28597	0.285972	2	0.758498	r0408	ATP:Sedoheptulose 7-phosphate 1-phosphotransferase EC:2.7.1.11	1.00 * ATP [c] + 1.00 * Sedoheptulose 7-phosphate [c] -->	PFKL	Unassigned		2.7.1.11	0
r0409_neg	3	0.662521	0.1788004	-0.28597	0.285972	2	0.758498	r0409	UTP:Sedoheptulose 7-phosphate 1-phosphotransferase EC:2.7.1.11	1.00 * UTP [c] + 1.00 * Sedoheptulose 7-phosphate [c] -->	PFKL	Unassigned		2.7.1.11	0
r0409_pos	3	0.662521	0.1788004	-0.28597	0.285972	2	0.758498	r0409	UTP:Sedoheptulose 7-phosphate 1-phosphotransferase EC:2.7.1.11	1.00 * UTP [c] + 1.00 * Sedoheptulose 7-phosphate [c] -->	PFKL	Unassigned		2.7.1.11	0
r0517_neg	3	0.662521	0.1788004	-0.91402	0.914024	2	0.758498	r0517	Succinyl-CoA:glycine C-succinyl-transferase(decarboxylating) EC:2.3	1.00 * proton [m] + 1.00 * 2-Amino-3-oxoadipate [m] -->	ALAS1; ALA	Unassigned		2.3.1.37	0
r0775_neg	6	0.662521	0.1788004	0.206319	0.206319	1	0.758498	r0775	Formamidopyrimidine nucleoside triphosphate 7,8-8,9-dihydrolase	1.00 * Water [c] + 1.00 * Formamidopyrimidine nucleosi	GCH1	Unassigned		3.5.4.16	0
r0317_neg	5	1	0	0.111631	0.111631	1	1	r0317	citrate hydro-lyase Citrate cycle (TCA cycle) / Glyoxylate and dicarbo	1.00 * Citrate [m] --> 1.00 * Water [m] + 1.00 * cis-aconita	ACO1; ACO	Unassigned		4.2.1.4	0
r0317_pos	5	1	0	0.111631	0.111631	1	1	r0317	citrate hydro-lyase Citrate cycle (TCA cycle) / Glyoxylate and dicarbo	1.00 * Citrate [m] --> 1.00 * Water [m] + 1.00 * cis-aconita	ACO1; ACO	Unassigned		4.2.1.4	0
r0425_neg	5	1	0	0.576024	0.576024	1	1	r0425	EC:1.1.1.42	1.00 * Nicotinamide adenine dinucleotide [m] + 1.00 * Is	IDH1; IDH2	Unassigned		1.1.1.42	0
r0426_neg	5	1	0	0.111631	0.111631	1	1	r0426	isocitrate hydro-lyase Citrate cycle (TCA cycle) / Glyoxylate and dicar	1.00 * Isocitrate [m] --> 1.00 * Water [m] + 1.00 * cis-acon	ACO1; ACO	Unassigned		4.2.1.3	0
r0426_pos	5	1	0	0.111631	0.111631	1	1	r0426	isocitrate hydro-lyase Citrate cycle (TCA cycle) / Glyoxylate and dicar	1.00 * Isocitrate [m] --> 1.00 * Water [m] + 1.00 * cis-acon	ACO1; ACO	Unassigned		4.2.1.3	0
r0610_neg	4	1	0	0.480189	0.480189	1	1	r0610	CTP:D-Tagatose 6-phosphate 1-phosphotransferase Galactose metab	1.00 * CTP [c] + 1.00 * D-tagatofuranose 6-phosphate(2-) [	PFKL	Unassigned		2.7.1.11	0
r0610_pos	4	1	0	0.480189	0.480189	1	1	r0610	CTP:D-Tagatose 6-phosphate 1-phosphotransferase Galactose metab	1.00 * CTP [c] + 1.00 * D-tagatofuranose 6-phosphate(2-) [	PFKL	Unassigned		2.7.1.11	0
r0611_neg	4	1	0	0.480189	0.480189	1	1	r0611	ITP:D-Tagatose 6-phosphate 1-phosphotransferase Galactose metab	1.00 * ITP(3-) [c] + 1.00 * D-tagatofuranose 6-phosphate(2	PFKL	Unassigned		2.7.1.11	0
r0611_pos	4	1	0	0.480189	0.480189	1	1	r0611	ITP:D-Tagatose 6-phosphate 1-phosphotransferase Galactose metab	1.00 * ITP(3-) [c] + 1.00 * D-tagatofuranose 6-phosphate(2	PFKL	Unassigned		2.7.1.11	0
CK_pos	1	0.19043	0.7202646	-0.96781	0.967811	2	0.347218	CK	ATP Creatine kinase	1.00 * ATP [m] + 1.00 * creatine [m] --> 1.00 * ADP [m] + 1.	CKMT1; CK	Urea cycle	33	2.7.3.2	4
CKc_neg	1	0.19043	0.7202646	-0.96781	0.967811	2	0.347218	CKc	ATP Creatine kinase (c)	1.00 * ATP [c] + 1.00 * creatine [c] --> 1.00 * ADP [c] + 1.00	CKB; CKM	Urea cycle	33	2.7.3.2	4
r0074_neg	7	0.382733	0.4171041	1.164738	1.164738	1	0.518179	r0074	L-Glutamate 5-semialdehyde:NAD+ oxidoreductase Arginine and pr	1.00 * Water [m] + 1.00 * Nicotinamide adenine dinucleo	ALDH4A1	Urea cycle	33	1.5.1.12	0
ILETA_pos	7	0.382733	0.4171041	0.681567	0.681567	1	0.518179	ILETA	isoleucine transaminase	1.00 * 2-Oxoglutarate [c] + 1.00 * L-isoleucine [c] --> 1.00	BCAT1	Valine, leucine, and isoleucine metabolism	34	2.6.1.42	4
ILETAm_neg	7	0.382733	0.4171041	0.681567	0.681567	1	0.518179	ILETAm	isoleucine transaminase, mitochondrial	1.00 * 2-Oxoglutarate [m] + 1.00 * L-isoleucine [m] --> 1.0	BCAT2	Valine, leucine, and isoleucine metabolism	34	2.6.1.42	4
LEUTA_pos	7	0.382733	0.4171041	0.681567	0.681567	1	0.518179	LEUTA	leucine transaminase	1.00 * 2-Oxoglutarate [c] + 1.00 * L-leucine [c] --> 1.00 * L	BCAT1	Valine, leucine, and isoleucine metabolism	34	2.6.1.6	4
LEUTAm_neg	7	0.382733	0.4171041	0.681567	0.681567	1	0.518179	LEUTAm	leucine transaminase, mitochondrial	1.00 * 2-Oxoglutarate [m] + 1.00 * L-leucine [m] --> 1.00 *	BCAT2	Valine, leucine, and isoleucine metabolism	34	2.6.1.6	4
MMEm_pos	2	0.382733	0.4171041	-0.68412	0.684123	2	0.518179	MMEm	methylmalonyl-CoA epimerase/racemase	1.00 * (R)-methylmalonyl-CoA(5-) [m] --> 1.00 * (S)-methy	MCEE	Valine, leucine, and isoleucine metabolism	34	5.1.99.1	4
VALTA_pos	7	0.382733	0.4171041	0.681567	0.681567	1	0.518179	VALTA	valine transaminase	1.00 * 2-Oxoglutarate [c] + 1.00 * L-valine [c] --> 1.00 * L-g	BCAT1	Valine, leucine, and isoleucine metabolism	34	2.6.1.42	4
VALTAm_neg	7	0.382733	0.4171041	0.681567	0.681567	1	0.518179	VALTAm	valine transaminase, mitochondiral	1.00 * 2-Oxoglutarate [m] + 1.00 * L-valine [m] --> 1.00 * L	BCAT2	Valine, leucine, and isoleucine metabolism	34	2.6.1.42	4
r0571_pos	2	0.382733	0.4171041	-0.68412	0.684123	2	0.518179	r0571	(S)-Methylmalonyl-CoA hydrolase Propanoate metabolism EC:3.1.2.	1.00 * Water [m] + 1.00 * (S)-methylmalonyl-CoA(5-) [m]	1.00 * Wat	Valine, leucine, and isoleucine metabolism	34	3.1.2.17	0
r0643_neg	2	0.382733	0.4171041	-0.68412	0.684123	2	0.518179	r0643	(S)-Methylmalonate semialdehyde:NAD+ oxidoreductase Valine, leu	1.00 * Water [m] + 1.00 * Nicotinamide adenine dinucleo	ALDH1B1;	Valine, leucine, and isoleucine metabolism	34	1.2.1.3	0
RDH1_neg	0	0.080856	1.0922877	-1.45296	1.452961	2	0.240222	RDH1	retinol dehydrogenase (all-trans)	1.00 * Nicotinamide adenine dinucleotide [c] + 1.00 * ret	RDH16F1;	Vitamin A metabolism		1.1.1.105	4
RDH1a_pos	0	0.080856	1.0922877	-1.45296	1.452961	2	0.240222	RDH1a	retinol dehydrogenase (all-trans,NADPH)	1.00 * Nicotinamide adenine dinucleotide phosphate [c]	RDH10; RD	Vitamin A metabolism		1.1.1.105	4
RDH2_neg	1	0.19043	0.7202646	-1.45601	1.456005	2	0.347218	RDH2	retinol dehydrogenase (9-cis,NADH)	1.00 * Nicotinamide adenine dinucleotide [c] + 1.00 * 9-c	RDH5	Vitamin A metabolism		1.1.1.105	4
RDH2a_pos	1	0.19043	0.7202646	-1.45601	1.456005	2	0.347218	RDH2a	retinol dehydrogenase (9-cis,NADPH)	1.00 * Nicotinamide adenine dinucleotide phosphate [c]	RDH11; RD	Vitamin A metabolism		1.1.1.105	4
RDH3_neg	1	0.19043	0.7202646	-1.45601	1.456005	2	0.347218	RDH3	retinol dehydrogenase (11-cis,NADH)	1.00 * Nicotinamide adenine dinucleotide [c] + 1.00 * 11	RDH5	Vitamin A metabolism		1.1.1.105	4
RDH3a_pos	1	0.19043	0.7202646	-1.45601	1.456005	2	0.347218	RDH3a	retinol dehydrogenase (11-cis,NADPH)	1.00 * Nicotinamide adenine dinucleotide phosphate [c]	RDH11; RD	Vitamin A metabolism		1.1.1.105	4
RDH1_pos	2	0.382733	0.4171041	-0.60313	0.603125	2	0.518179	RDH1	retinol dehydrogenase (all-trans)	1.00 * Nicotinamide adenine dinucleotide [c] + 1.00 * ret	RDH16F1;	Vitamin A metabolism		1.1.1.105	4
RDH2_pos	2	0.382733	0.4171041	-0.60313	0.603125	2	0.518179	RDH2	retinol dehydrogenase (9-cis,NADH)	1.00 * Nicotinamide adenine dinucleotide [c] + 1.00 * 9-c	RDH5	Vitamin A metabolism		1.1.1.105	4
RDH3_pos	2	0.382733	0.4171041	-0.60313	0.603125	2	0.518179	RDH3	retinol dehydrogenase (11-cis,NADH)	1.00 * Nicotinamide adenine dinucleotide [c] + 1.00 * 11	RDH5	Vitamin A metabolism		1.1.1.105	4
RE3347C_pos	0	0.080856	1.0922877	-2.27244	2.272442	2	0.240222	RE3347C	RE3347	1.00 * proton [c] + 1.00 * Nicotinamide adenine dinucleo	1.00 * prot	Vitamin B2 metabolism		1.5.1.29	0
RE3347C_neg	2	0.382733	0.4171041	-0.73078	0.730783	2	0.518179	RE3347C	RE3347	1.00 * proton [c] + 1.00 * Nicotinamide adenine dinucleo	1.00 * prot	Vitamin B2 metabolism		1.5.1.29	0

Supplementary Table 4.

Metabolite	Isotopologue	Q1	Q3
Acetyl CoA	Acetyl CoA.1 M0	810.1	303.1
Acetyl CoA	Acetyl CoA.1 M1	811.1	304.1
Acetyl CoA	Acetyl CoA.1 M2	812.1	305.1
Acetyl CoA	Acetyl CoA.1 M3	813.1	306.1
Acetyl CoA	Acetyl CoA.1 M4	814.1	307.1
Acetyl CoA	Acetyl CoA.1 M5	815.1	308.1
Propionyl CoA	Propionyl CoA.1 M0	824.1	317.1
Propionyl CoA	Propionyl CoA.1 M1	825.1	318.1
Propionyl CoA	Propionyl CoA.1 M2	826.1	319.1
Propionyl CoA	Propionyl CoA.1 M3	827.1	320.1
Propionyl CoA	Propionyl CoA.1 M4	828.1	321.1
Propionyl CoA	Propionyl CoA.1 M5	829.1	322.1

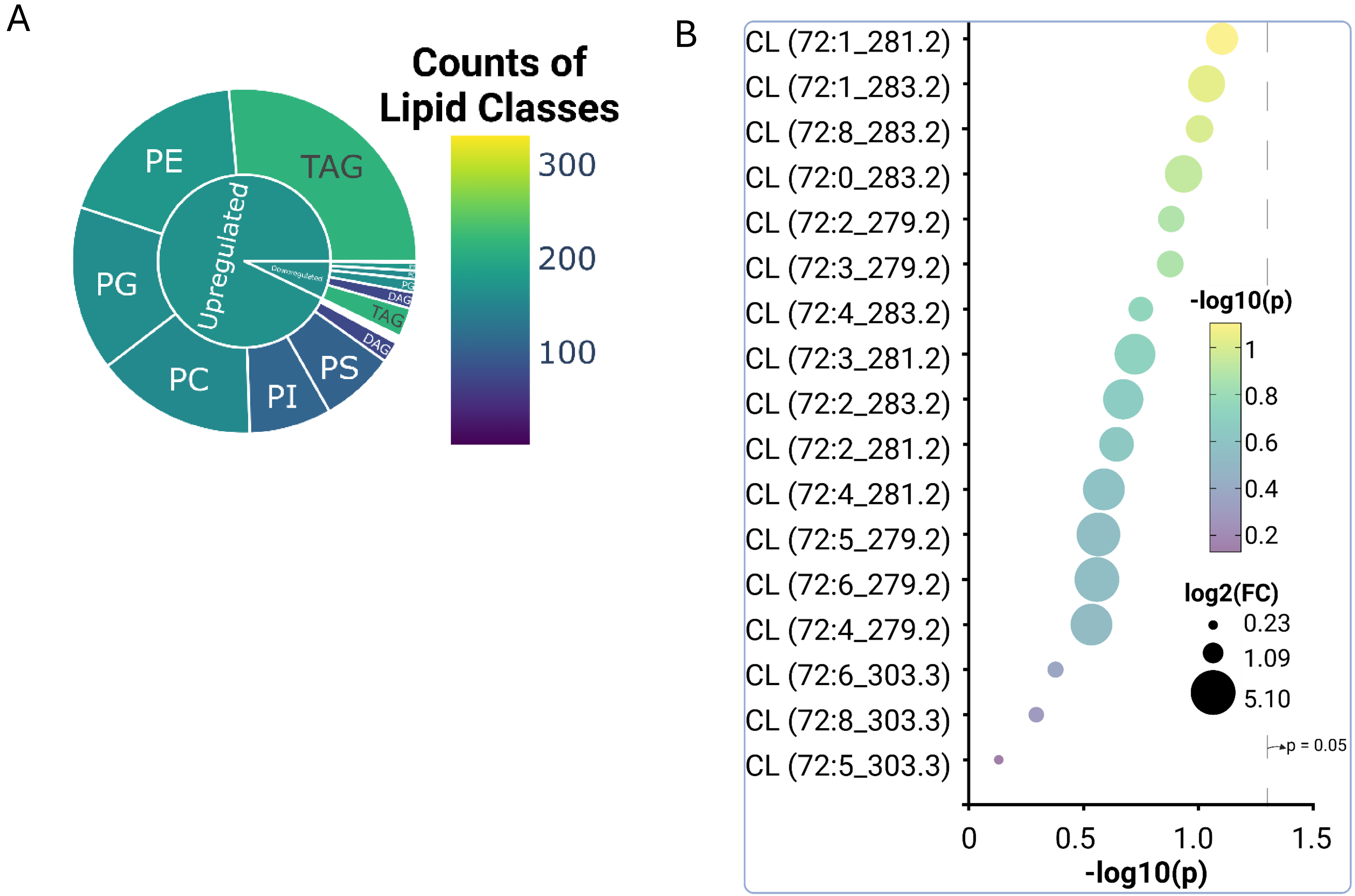

Supplementary Figure 1

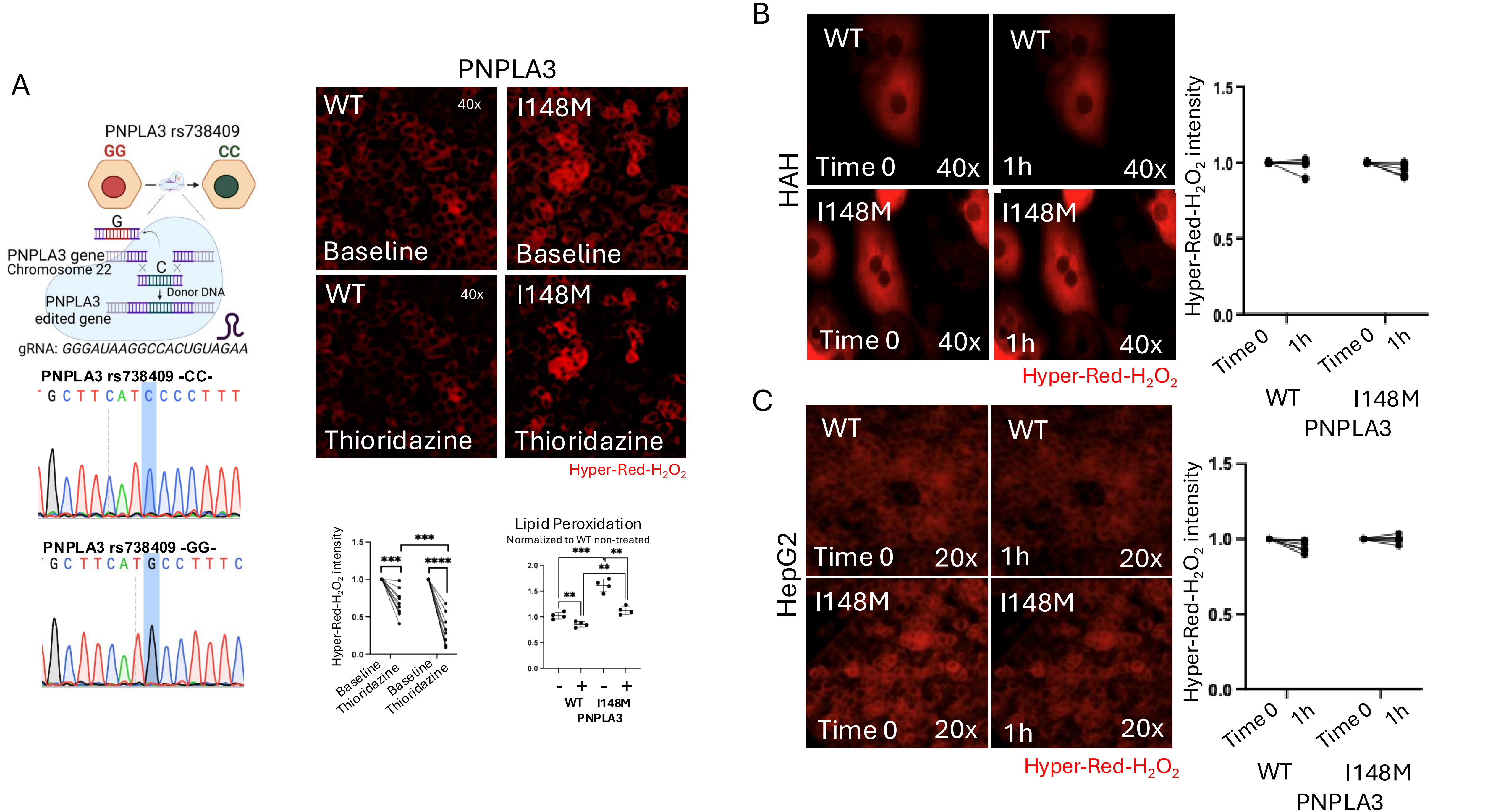

Supplementary Figure 2

A

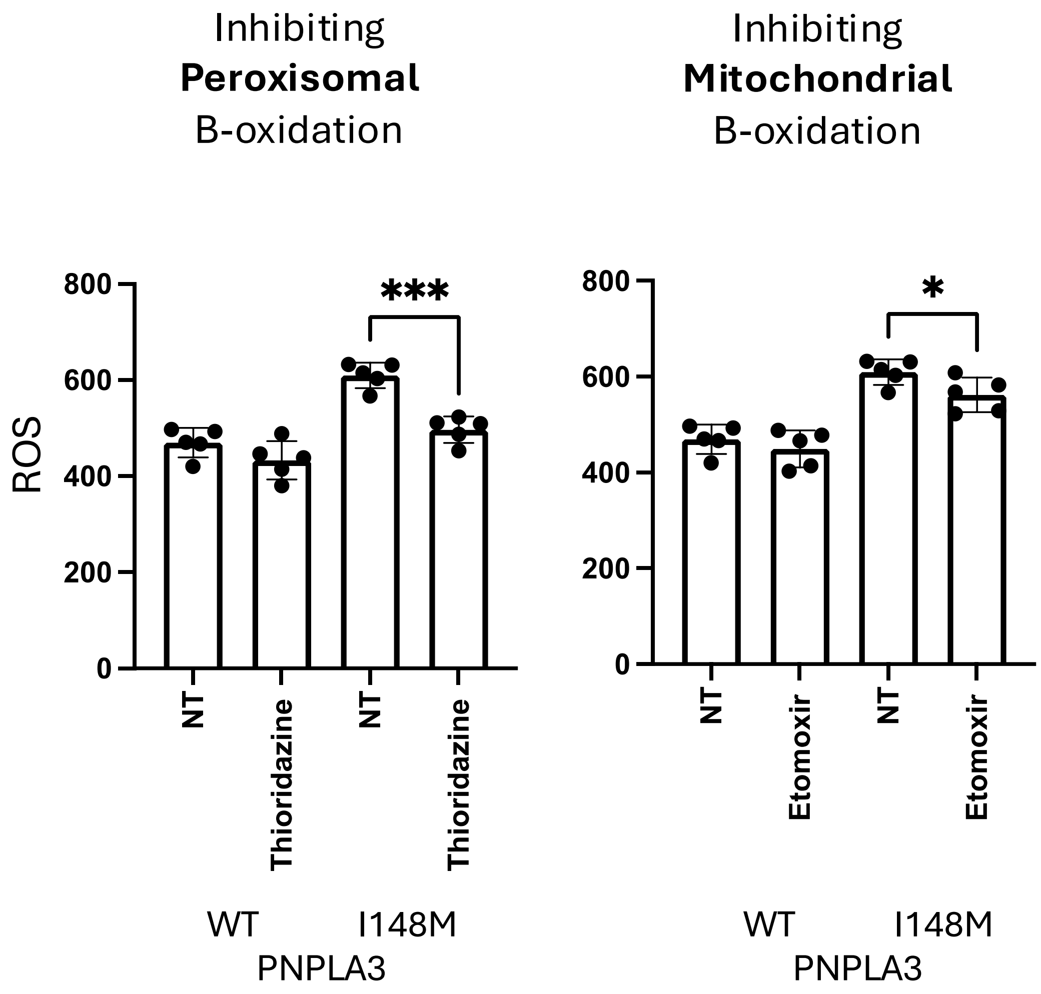

Supplementary Figure 3

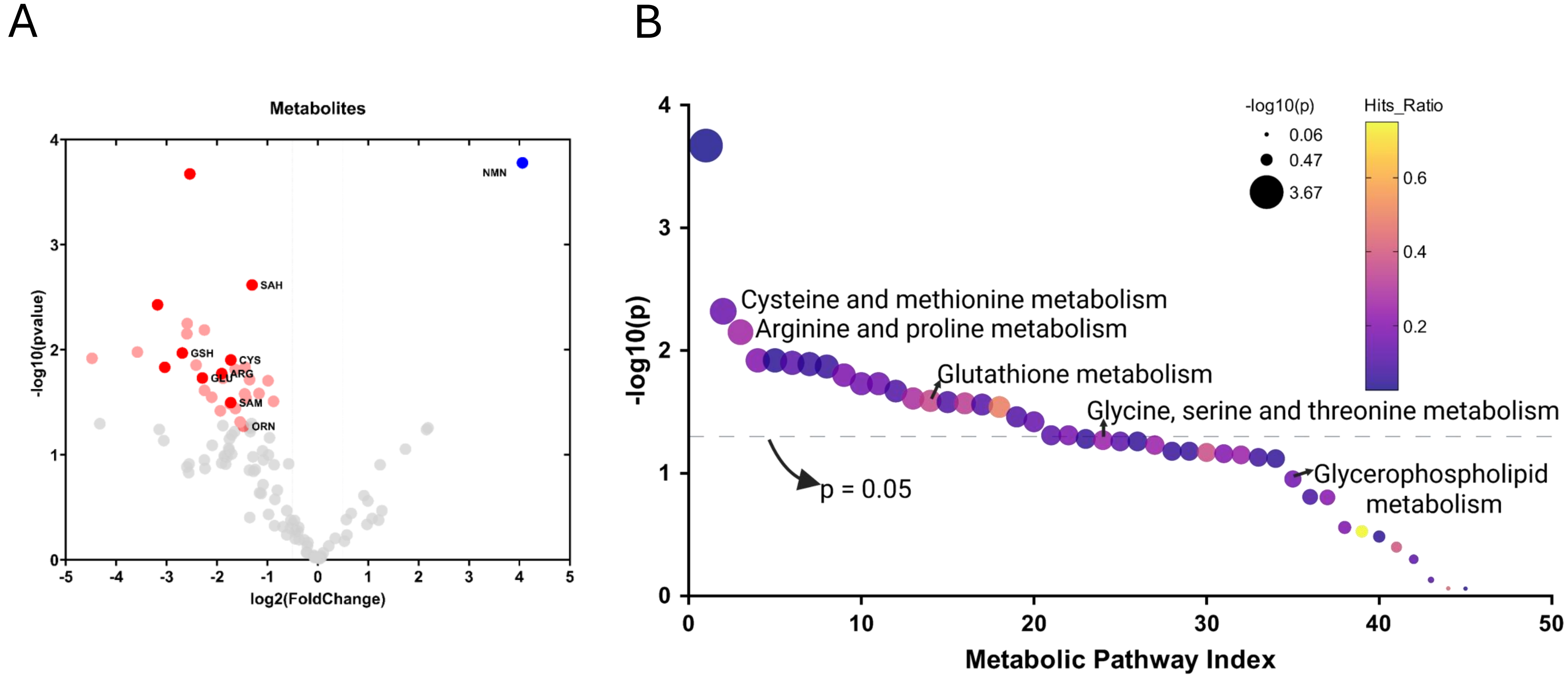

Supplementary Figure 4

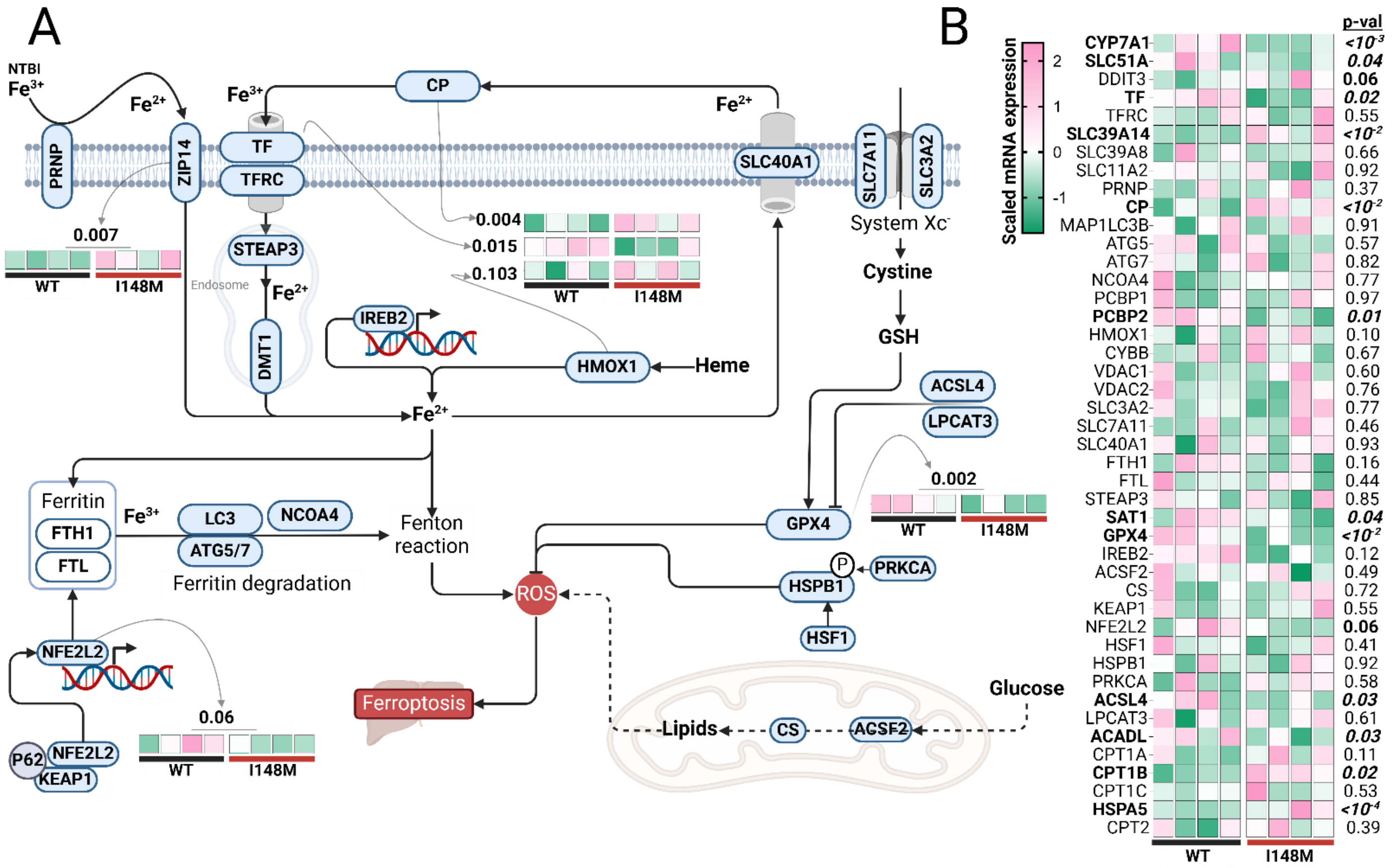

Supplementary Figure 5

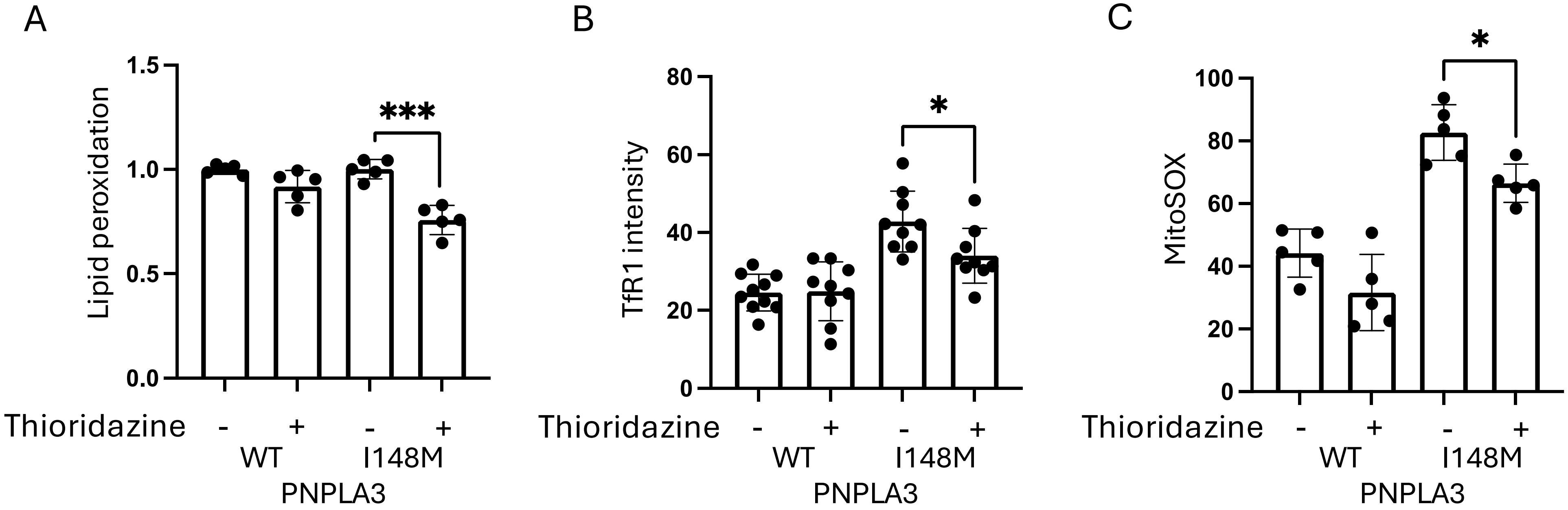

Supplementary Figure 6

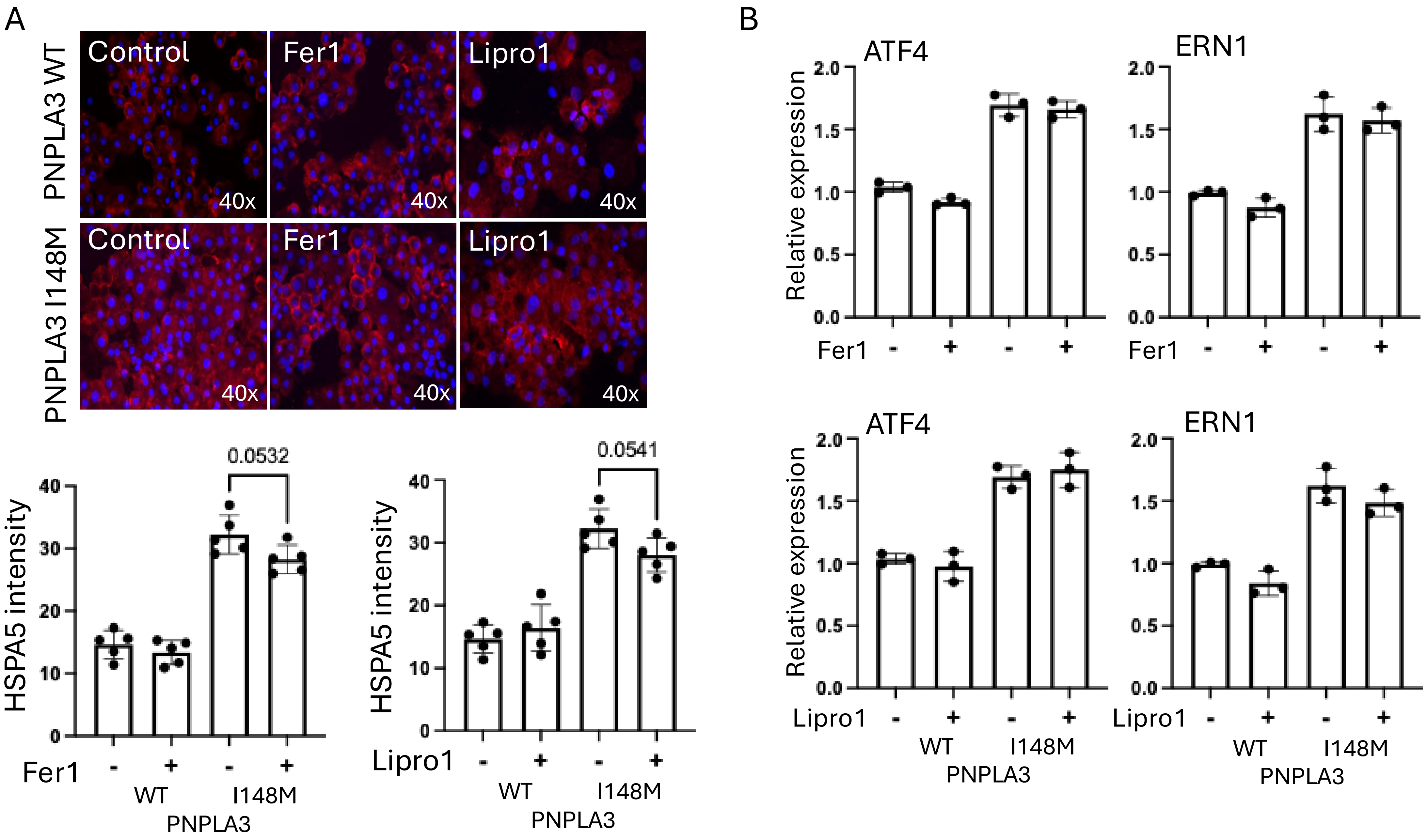

Supplementary Figure 7

(A)

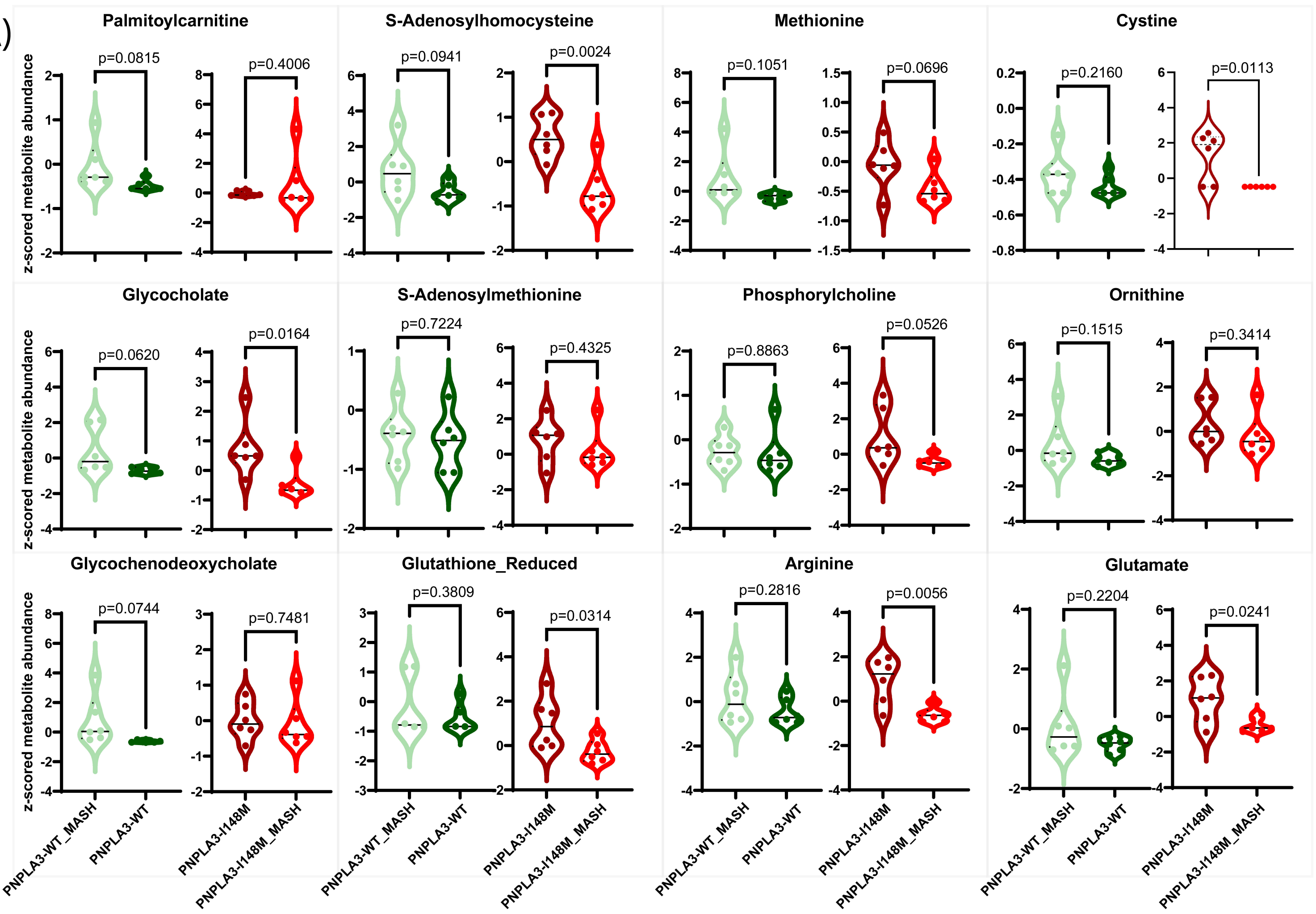

Palmitoylcarnitine
p=0.0815
p=0.4006
S-Adenosylhomocysteine
p=0.0941
p=0.0024
Methionine
p=0.1051
p=0.0696
Cystine
p=0.2160
p=0.0113
Glycocholate
p=0.0620
p=0.0164
S-Adenosylmethionine
p=0.7224
p=0.4325
Phosphorylcholine
p=0.8863
p=0.0526
Ornithine
p=0.1515
p=0.3414
Glycochenodeoxycholate
p=0.0744
p=0.7481
Glutathione_Reduced
p=0.3809
p=0.0314
Arginine
p=0.2816
p=0.0056
Glutamate
p=0.2204
p=0.0241
z-scored metabolite abundance
PNPLA3-WT_MASH
PNPLA3-WT
PNPLA3-I148M
PNPLA3-I148M_MASH

(B)

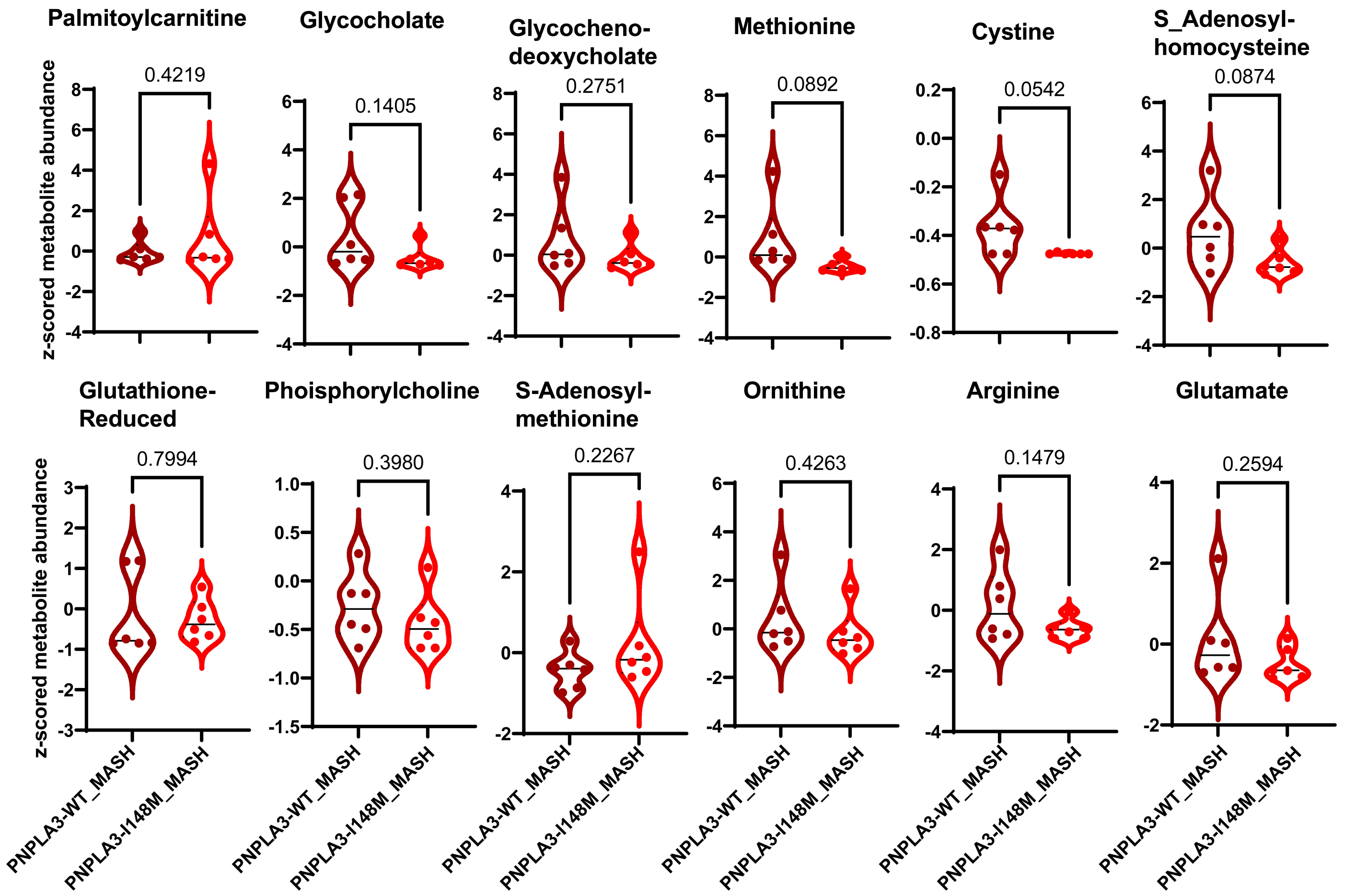

Palmitoylcarnitine
0.4219
Glycocholate
0.1405
Glycocheno-
deoxycholate
0.2751
Methionine
0.0892
Cystine
0.0542
S_Adenosyl-
homocysteine
0.0874
Glutathione-
Reduced
0.7994
Phoisphorylcholine
0.3980
S-Adenosyl-
methionine
0.2267
Ornithine
0.4263
Arginine
0.1479
Glutamate
0.2594
z-scored metabolite abundance
PNPLA3-WT_MASH
PNPLA3-I148M_MASH

A

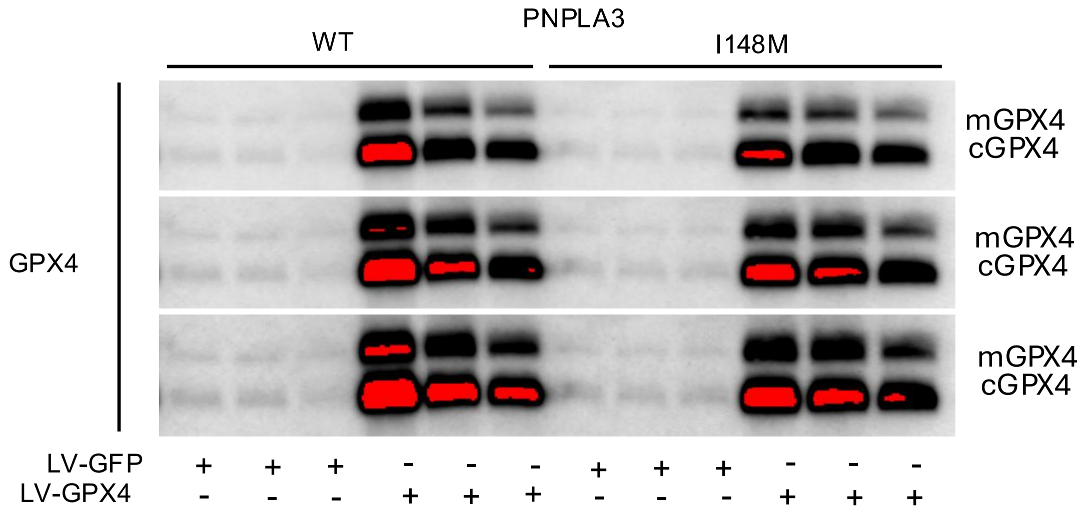

Supplementary Figure 9

A

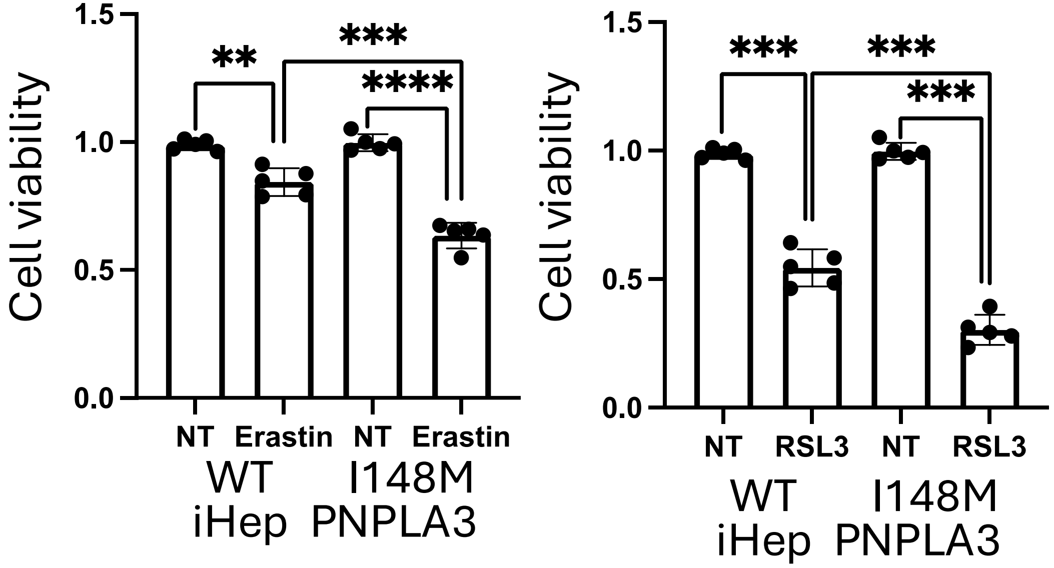

Supplementary Figure 10

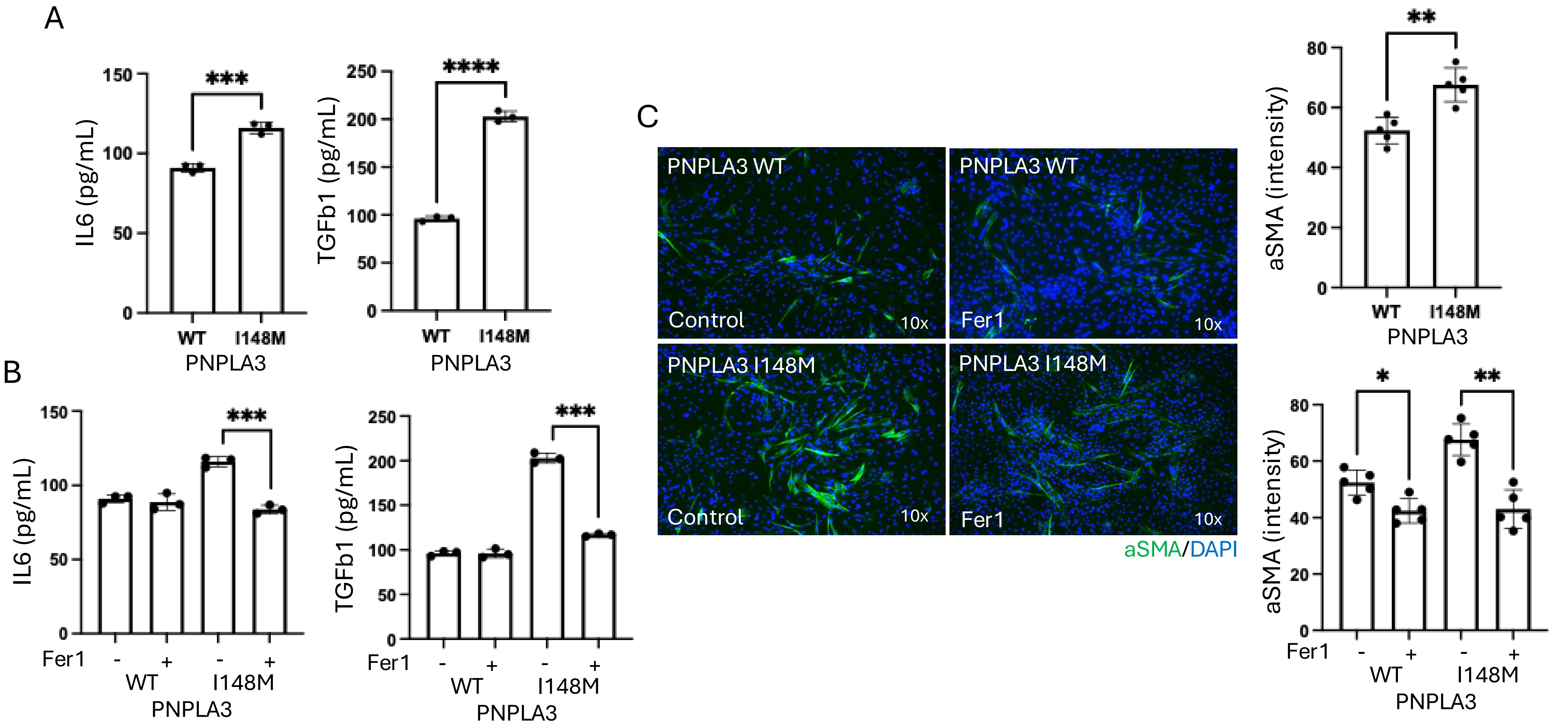

Supplementary Figure 11

Supplementary Figures Footnotes:

Supplementary figure 1: (A) Sunburst plot shows the distribution of upregulated and downregulated lipid classes in Figure 1I. Lipid classes legend; TAG: Triacylglycerols, PS: Phosphatidylserine, PI: Phosphatidylinositol, PG: Phosphatidylglycerol, PE: Phosphatidylethanolamine, PC: Phosphatidylcholine, MAG: Monoacylglycerol, LPS: Lipopolysaccharide , LPI: Lysophosphatidylinositol, LPG: Lysophosphatidylglycerol, LPC: Lysophosphatidylcholine, DAG: Diacylglycerol, CL: Cardiolipin, CE: Cholesteryl Ester. **(B)** Bubble plot showing the level of mitochondria-associated cardiolipins (CL) in Hep-PNPLA3-I148M compared to Hep-PNPLA3-WT. The size of the bubbles indicates the log2 fold change of the lipid cardiolipins upregulated in Hep-PNPLA3-I148M versus WT. The color of the bubbles corresponds to the –log10(p-value). Test statistics were computed with Metaboanalyst® (ver. 6.0). Example of cardiolipin naming convention: CL (72:4_281.2) implies a cardiolipin with 72 carbons (four C18 acyl chains); 4 total double bonds in all the four constituting fatty acids. A daughter ion with molecular weight of 281.2 results from its fragmentation which corresponds to oleic acid (C18:1) in this case.

Supplementary Figure 2: (A) The single-guide RNA (sgRNA) sequence was designed to cut at chr22:43,928,854 to replace the minor allele -G- with the major allele -C- using a donor template. Sanger sequences of the PNPLA3 genotypes. Fluorescent micrographs of HyPer-Red in transfected HepG2-PNPLA3-I148M and HepG2-PNPLA3-WT controls exposed to thioridazine for 1h between micrographs. Quantification of H_2O_2 by HyPer-Red intensity (***$p<0.001$, ****$p<0.0001$, Welch's t-test, WT: n=10, I148M: n=10). Lipid peroxidation suppression on HepG2-PNPLA3-I148M and HepG2-PNPLA3-WT controls exposed to thioridazine (**$p=0.001$, ****$p<0.0001$, Welch's t-test, WT: n=3 I148M: n=4). Hyper-Red-H_2O_2 intensity at time 0 and after 1 hour without exposing **(B)** primary human hepatocytes and **(C)** HepG2 cells to thioridazine. No significant decrease in the fluorescence signal was observed over time.

Supplementary Figure 3: The major ROS source in Hep-PNPLA3-I148M is peroxisomal β-oxidation. Primary human Hep-PNPLA3-WT or Hep-PNPLA3-I148M were exposed to thioridazine or etomoxir, inhibitors of peroxisomal and mitochondrial β-oxidation respectively. About 20% of ROS was decreased in the mutated cells when treated with thioridazine (***$p<0.001$, Welch's t-test, WT: n=6, I148M: n=6) and about 9% was reduced when the cells were exposed to etomoxir (*$p<0.05$, Welch's t-test, WT: n=6, I148M: n=6).

Supplementary Figure 4: (A) Volcano plot indicating various metabolites that are differentially expressed between Hep-PNPLA3-I148M (n=6) and Hep-PNPLA3-WT (n=6). Red circles indicate metabolites significantly upregulated (p value < 0.05, fold change > 1.5) while blue indicates metabolites that are significantly downregulated (p value < 0.05, fold change < 1/1.5) in Hep-PNPLA3-I148M compared to Hep-PNPLA3-WT. Test statistics were computed with Metaboanalyst® (ver. 4.0). Metabolites legend; SAH: s-adenosylhomocysteine, GSH: Glutathione, GLU: Glutamate, ARG: arginine, CYS: cystine, SAM: s-adenosylmethionine, ORN: ornithine, NMN: nicotinamide mononucleotide. (B) Bubble plot showing various metabolic pathways that are significantly different between Hep-PNPLA3-I148M (n=6) and Hep-PNPLA3-WT (n=6). The plot was generated from data obtained from metabolic set enrichment analysis performed in Metaboanalyst ® (ver. 4.0).

Supplementary Figure 5: (A) Simplified Kyoto Encyclopedia of Genes and Genomes (KEGG) ferroptosis pathway overlayed with transcriptomic data of genes involved in hepatocellular ferroptosis and iron metabolism comparing Hep-PNPLA3-I148M (n=4) and Hep-PNPLA3-WT (n=4). **(B)** Heatmap showing differential expression based on z-score of genes related to oxidative stress, ferroptosis, and iron metabolism in Hep-PNPLA3-I148M (n=4) and Hep-PNPLA3-WT (n=4). The genes with bold names are the ones with statistically significant differences between the two groups (p value < 0.05). The p value was computed based on Wald test statistics.

Supplementary Figure 6: Ferroptosis characterization after inhibiting peroxisomal β-oxidation with thioridazine in Hep-PNPLA3-WT and Hep-PNPLA3-I148M. Lipid peroxidation (***p<0.001, Welch's t-test, WT: n=5, I148M: n=5); TfR1 intensity (*p<0.05, Welch's t-test, WT: n=10, I148M: n=10), and MitoSOX (*p<0.05, Welch's t-test, WT: n=5, I148M: n=5).

Supplementary Figure 7: Inhibition of ferroptosis did not reduce ER stress in primary human hepatocytes carrying the PNPLA3-I148M variant. **(A)** HSPA5 immunostaining was performed after exposing primary human hepatocytes to Fer1 and Lipro1. While high signal was observed in non-treated Hep-PNPLA3-I148M in comparison to Hep-PNPLA3-WT, no statistical difference was observed after ferroptosis inhibition **(**Welch's t-test, WT: n=5, I148M: n=5). **(B)** The same phenomenon was observed at the mRNA levels of ATF and ERN1, components of the unfolded protein response (Welch's t-test, WT: n=3, I148M: n=3).

Supplementary Figure 8: Comparing levels of metabolites **(A)** between samples from healthy and MASH patients and **(B)** between PNPLA3-WT MASH patients and PNPLA3-I148M MASH patients

Supplementary Figure 9: Overexposure of the primary human hepatocytes transduced with the LV-GPX to show the basal level of GPX4 expression in the cells transduced with the LV-GFP.

Supplementary Figure 10: iHep-PNPLA3-I148M are more susceptible to cell death after exposure to ferroptosis inducers, such as erastin (**p<0.01, ***p<0.001, ****p<0.0001, Welch's t-test, WT: n=5, I148M: n=5) and RSL3 (***p<0.001, Welch's t-test, WT: n=5, I148M: n=5).

Supplementary Figure 11: PNPLA3-I148M and ferroptosis play a role in the inflammation and fibrosis process. A co-culture of hepatocytes (iHep) and hepatic stellate cells (iHSC) derived from iPSC-PNPLA3-WT and iPSC-PNPLA3-I148M was used to measure the secretion of key cytokines and activation of iHSC through aSMA immunostaining. **(A)** The system carrying the PNPLA3-I148M variant had increased production of IL6 (***p<0.001, Welch's t-test, WT: n=3, I148M: n=3) and TGFb1 (****p<0.0001, Welch's t-test, WT: n=3, I148M: n=3). **(B)** Ferroptosis inhibition with Ferrostatin 1 (Fer1) reduced the production both proinflammatory cytokines: IL6 (***p<0.001, Welch's t-test, WT: n=3, I148M: n=3) and TGFb1 (***p<0.001, Welch's t-test, WT: n=3, I148M: n=3). **(C)** The impact of PNPLA3 mutation in the fibrosis was assessed by aSMA intensity. The system carrying the mutation started with a high basal level of activated iHSC (**p<0.01, Welch's t-test, WT: n=5, I148M: n=5), which was reduced upon Fer1 treatment (*p<0.05, **p<0.01, Welch's t-test, WT: n=5, I148M: n=5).
