# Supplementary figures and images for: PNPLA3-I148M genetic variant rewires lipid metabolism to drive programmed cell death in human hepatocytes
Source: JCI Insight. 2025 Oct 21;10(23):e193805. doi: 10.1172/jci.insight.193805 (PMC12890524; doi:10.1172/jci.insight.193805)

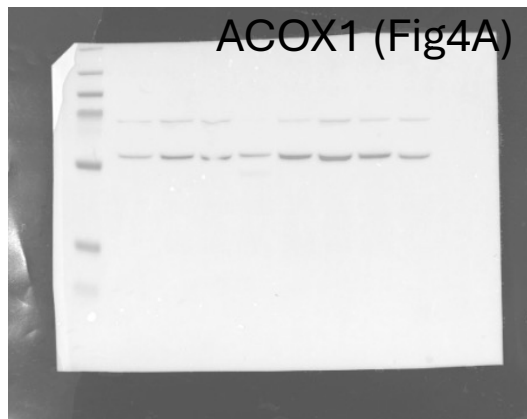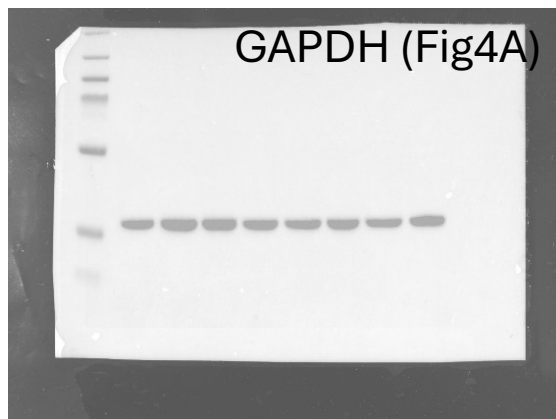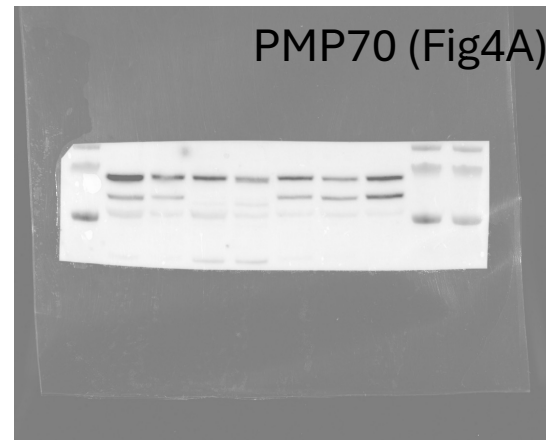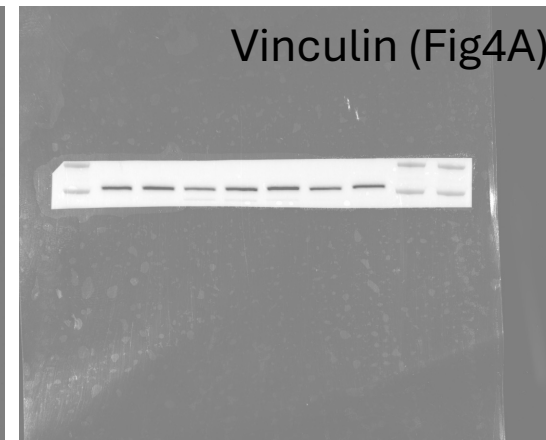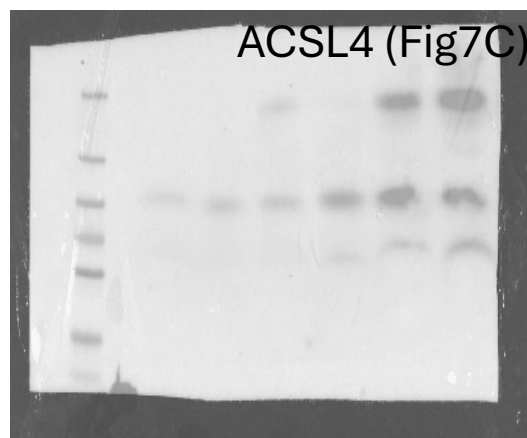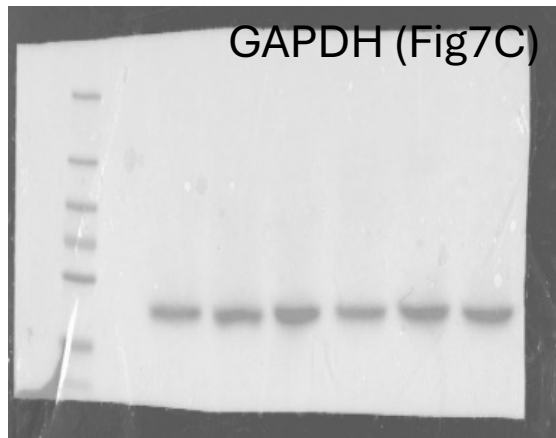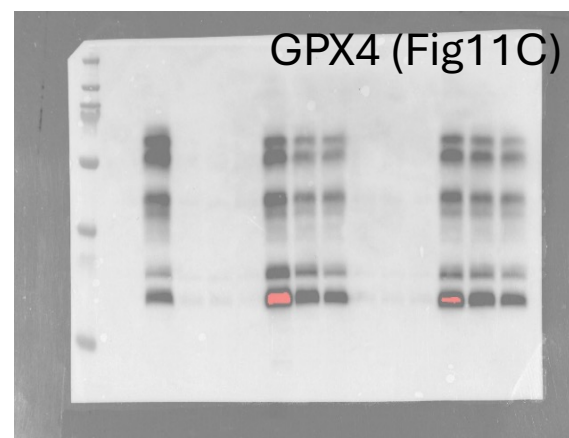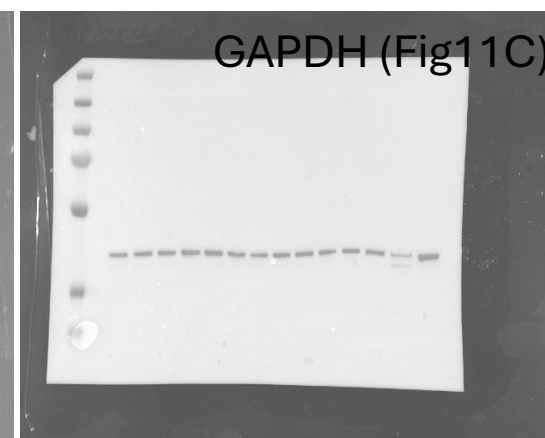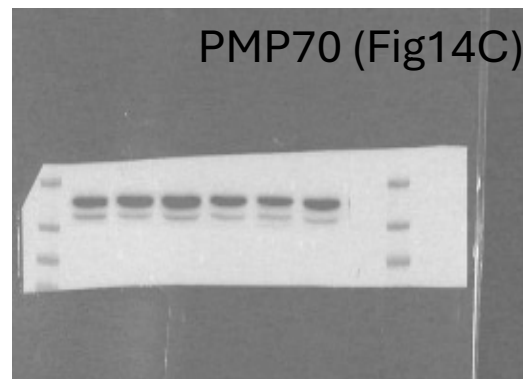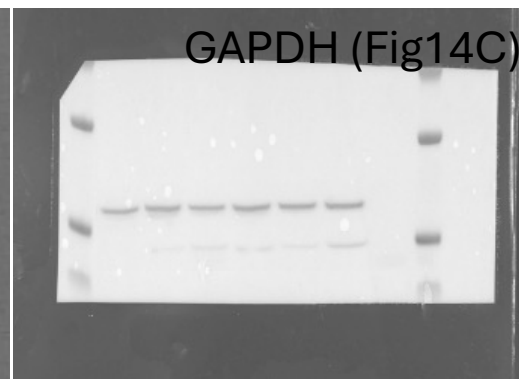

Supplement: Unedited blot and gel images [file jciinsight-10-193805-s058.pdf]
